# Biodegradation of Punicalagin into Ellagic Acid by Selected Probiotic Bacteria: A Study of the Underlying Mechanisms by MS-Based Proteomics

**DOI:** 10.1021/acs.jafc.2c06585

**Published:** 2022-12-15

**Authors:** Víctor Caballero, Mario Estévez, Francisco A. Tomás-Barberán, David Morcuende, Irene Martín, Josué Delgado

**Affiliations:** † Food Technology, IPROCAR Research Institute, Universidad de Extremadura, 10003Cáceres, Spain; ‡ Food Hygiene and Safety, IPROCAR Research Institute, Universidad de Extremadura, 10003Cáceres, Spain; § Research Group on Quality, Safety and Bioactivity of Plant-Derived Foods, CEBAS-CSIC, 30100Murcia, Spain

**Keywords:** lactic acid bacteria, ellagic acid, urolithin, punicalagin, proteomics, metabolomics

## Abstract

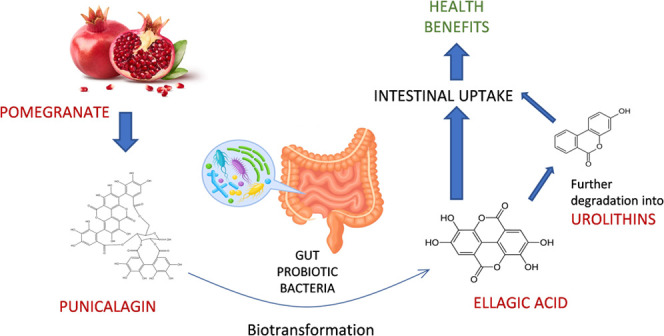

Pomegranate (*Punica granatum* L.) is a well-known source of bioactive phenolic compounds such as ellagitannins, anthocyanins, and flavanols. Punicalagin, one of the main constituents of pomegranate, needs to be biodegraded by bacteria to yield metabolites of medicinal interest. In this work, we tested 30 lactic acid bacteria (LAB) and their capacity to transform punicalagin from a punicalagin-rich pomegranate extract into smaller bioactive molecules, namely, ellagic acid and urolithins. These were identified and quantified by high-performance liquid chromatography-electrospray ionization tandem mass spectrometry (HPLC-ESI-MS^2^). Further, we evaluated the molecular mechanism governing this transformation through label-free comparative MS-based proteomics. All tested LAB strains were capable of transforming punicalagin into ellagic acid, while the biosynthesis of urolithins was not observed. Proteomic analysis revealed an increase of generic transglycosylases that might have a hydrolytic role in the target phenolic molecule, coupled with an increase in the quantity of ATP-binding cassette (ABC) transporters, which might play a relevant role in transporting the resulting byproducts in and out of the cell.

## Introduction

The human gastrointestinal tract (GIT) represents one of the largest interfaces between the host and external pathogens, which impose a threat to human health. The collection of bacteria, archaea, and eukarya colonizing the GIT is known as gut microbiota. In healthy individuals, Gram-negative Proteobacteria and Bacteroidetes and Gram-positive Firmicutes are the most representative ones among eubacteria.^1^ Microbes colonize human hosts immediately after birth. However, the gut microbiota is not a static ecosystem as it can be changed depending on lifestyle, diet, infections, exposure to antibiotics, or surgical interventions.^2^ Alteration of the bowel microbiota composition of prevailing bacterial groups with negative physiological impact is called dysbiosis and could have devastating consequences on human health.^3^ Many diseases have been related to a damaged microbiota status in the gut, including type 2 diabetes (T2D), allergies, nonalcoholic fatty liver disease (NAFLD), obesity, and inflammatory bowel diseases (IBD).^4^

Probiotics are, by definition, “living microorganisms that, when administered in adequate amounts, confer a health benefit on the host”.^4^ Microorganisms with probiotic properties commonly used as dietary supplements belong to the genera *Lactobacillus* and *Bifidobacterium*. The scientific literature is full of examples on how dietary supplementation with probiotic bacteria has a protective effect against the onset of some of the aforementioned diseases. Regarding T2D, probiotics have recently shown their benefits when administered in either animals^5,6^ or humans,^7^ strengthening gut barrier function, reshaping gut microbiota composition, and lowering proinflammatory cytokines, such as interleukin (IL)-6, IL-8, and tumor necrosis factor (TNF)-α, while increasing anti-inflammatory ones like IL-10 and IL-4.

The administration of VSL#3, a probiotic mixture consisting of *Streptococcus salivarius* subsp. *thermophilus*, *Lacticaseibacillus casei*, *Lactiplantibacillus plantarum*, *Lactobacillus acidophilus*, *Lactobacillus delbrueckii* subsp. *bulgaricus*, *Bifidobacterium longum*, *Bifidobacterium infantis*, and *Bifidobacterium breve*, coupled with the current antibiotic 5-aminosalicylates (5-ASA), to individuals suffering from ulcerative colitis (UC), was proven to be effective in both induction and maintenance of the remission of the disease.^8^ Probiotics have also been found to alleviate symptom severity in patients suffering from irritable bowel syndrome (IBS) when compared with placebo groups.^9^

Polyphenols are present in a wide range of plant foods. Their impact on human health is documented through a variety of bioactivities, including their ability to function as prebiotics and reshape gut microbiota into a healthier one.^10,11^ Among those plant foods, pomegranate (*Punica granatum*) has attracted extensive interest due to its phytochemical components (i.e., ellagitannins, gallotannins, and anthocyanins) and their assorted bioactivities.^12^ When compared to control or placebo, research has shown an increase in the population of the genera *Lactobacillus* and *Bifidobacterium* when pomegranate polyphenols were administered,^13^ which emphasizes the effect of dietary polyphenolics as prebiotic compounds. A research study has shown the beneficial effects of pomegranate consumption, either as peel extract or juice, achieving effective results in diminishing oxidation in lipids and proteins.^14^ This antioxidant capacity is highly correlated with the quantity and the type of polyphenols found in pomegranate. The richer the fruit part is in punicalagin, the greater the antioxidative potential it is endowed with.^15^

Punicalagin is a natural component in pomegranate and belongs to the family of ellagitannins. The biological interest of punicalagins arises from being the precursor of ellagic acid (EA) and other smaller bioactive phenolic compounds. Enzymes involved in ellagitannin hydrolysis are known as tannases. After hydrolysis by tannase enzymes, the released intermediate compound undergoes a spontaneous lactonization to form ellagic acid.^16^ In turn, this can be transformed into a variety of smaller molecules called urolithins, which could have a huge positive impact on health as an antioxidant^16^ and as a gut barrier function enhancer.^17^ At this point, the biotransformation of punicalagin into smaller compounds is key. Two bacteria from the *Eggerthellaceae* family named *Gordonibacter urolithinfaciens* and *Gordonibacter pamelaeae* are able to produce urolithins from ellagitannins.^18^ Urolithin produced by these species is an intermediate to other isoforms that have more bioactivity. It has been recently described as a bacteria isolated from human gut belonging to the same family, named as *Ellagibacter isourolithinifaciens*, with the ability to metabolize ellagic acid into isourolithin A.^19^ Research has demonstrated the capacity of a strain of *L. plantarum* to produce ellagic acid from pomegranate juice (PJ) in a 5-day window^20^ and a 24 h window.^21^ In this research, the antioxidant capacity has also been proven. Yet, the current knowledge on the capacity of lactic acid bacteria (LAB) to degrade punicalagin is scarce, and the underlying molecular mechanisms implicated in the interaction between the phytochemical and these probiotic bacteria are unknown.

The purpose of the present study was to assess the ability of selected probiotic bacteria to degrade punicalagin into bioactive compounds and identify the underlying molecular mechanisms by studying the proteome of the bacteria exposed to the phytochemicals present in a nutritional supplement extracted from pomegranate.

## Materials and Methods

### Chemicals and Reagents

De Man, Rogosa and Sharpe broth medium (MRS) was purchased from CondaLab (Spain). All chemicals used in high-performance liquid chromatography (HPLC) were liquid chromatography/mass spectrometry (LC/MS) Grade from Fisher Scientific. Trypsin and ProteaseMAX for proteomic digestion were purchased from PROMEGA. Punicalagin (A + B) mixture was acquired from PhytoLab GmbH & Co. KG (Germany). Reagents were acquired from Scharlab (Spain), ThermoFisher or Acros Organics. Ellagic acid and urolithin standards were purchased from Sigma-Aldrich. Urolithin A has a purity of ≥97%, and urolithin B has a purity of ≥95%. A commercial food supplement of punicalagin-rich extract (Punicalagina granatum plus+) was provided by Antioxidantes del Mediterráneo S.L. (Spain). The pomegranate powder had 300 mg of punicalagin per gram of product.

### Bacterial Cultures

LAB used in this assay were isolated and characterized from ripened cheese and dry-cured fermented sausages.^22^ In a preliminary study, 30 of these strains, listed in Table 1, were tested under physiological conditions (37 °C and 5% CO_2_) in MRS to assess their viability under usual cell culture conditions for further experiments. Bacteria were kept at −80 °C until their use. To revitalize sterilized bacteria, MRS was previously prepared following the manufacturer’s recommended procedure.

**Table 1:** Nomenclature of Bacteria Tested

bacterial strains tested	
*L. plantarum*	*L. plantarum*
*Lacticaseibacillus paracasei*	*Enterococcus faecium*
*Latilactobacillus sakei*	*Leuconostoc mesenteroides*
*Enterococcus hirae*	*Lactococcus lactis*
*L. garvieae* subsp. *garvieae*	*L. casei*
*E. faecium*	*E. faecium*
*L. casei*	*L. sakei*
*L. sakei*	*L. paracasei*
*E. faecium*	*L. lactis* subsp. *cremoris*
*E. faecium*	*E. faecium*
*L. casei*	*Enterococcus durans*
*E. durans*	*L. sakei*
*L. casei*	*L. mesenteroides*
*L. paracasei*	*L. lactis* subsp. *cremoris*

### Experimental Setting

Once bacteria were fully revitalized in MRS broth (after 24 h), each one was exposed to a commercial punicalagin-rich dietary supplement (Punicalagina granatum plus+, Antioxidantes del Mediterráneo S.L., Spain) at a final concentration of punicalagin of 30 μg/mL MRS. To assess the biodegradation of punicalagin by the bacteria, four different types of experimental units were incubated in the same conditions: C_1_: MRS, C_2_: MRS with punicalagin, B + P: MRS with bacteria and punicalagin, and B: MRS with bacteria and without punicalagin. For this preliminary test, two replicates (*n* = 2) of each bacterium were made. While a comparison of B + P vs B would indicate whether bacteria were implicated in the formation of punicalagin byproducts, additional controls and C_2_ were considered to (i) check the occurrence of the bioactive compounds in the MRS (C_1_) and (ii) assess the potential degradation of punicalagin into the compounds of interest in the set conditions by chemical mechanisms (no implication of bacteria). The samples from all experimental units were collected after 24 h incubation to analyze the occurrence and concentration of punicalagin byproducts (ellagic acid and urolithins) by the analytical procedure described in due course.

After screening the 30 initial bacterial strains, 10 of them were selected and retested in triplicate (*n* = 3) based on their bioactivity shown in the first assay. The molecular mechanisms implicated in the biodegradation of punicalagin by label-free MS-based proteomics were investigated in three strains among these last 10 strains. These bacteria were selected among those displaying a more intense activity in the biodegradation of punicalagin. Bacteria eventually selected were *L. plantarum* 89, *L. paracasei* 116, and *E. faecium* 126. For proteomic analyses, each bacterium was incubated in the presence (B + P) and in the absence (B) of the punicalagin product in quintuplicate (*n* = 5).

### Phenolic Content Extraction

At sampling times, bacteria were centrifuged (15 min at room temperature at 7197*g*, Eppendorf 5430 centrifuge), and supernatants were treated for the extraction of phenolic compounds following the procedure described by Delgado et al.^23^ with some modifications. The QuEChERS methodology was applied using a mixture of equal volumes of diethyl ether and ethyl acetate (1:1, v/v). Phase partitioning was conducted using 0.4 g of NaCl and 1.6 g of anhydrous MgSO_4_ (both from Scharlab S.L.). The mixture was shaken vigorously by hand and centrifuged (5 min at room temperature at 2630*g*, Orto Alresa Digtor 21R). After the extraction, the organic phase was collected (1 mL), filtered through 0.22 μm, and allowed to evaporate naturally overnight in a laminar flow cabinet in complete darkness.

Dried extracts of phenolic compounds were resuspended in the same volume of water/acetonitrile 50:50.

### Analysis of Phenolic Compounds

The mixture of phenolics from extractions was separated in a Dionex UltiMate 3000 RSLCnano system (ThermoFisher) with the following program: 0–3 min (10% B, isocratic), 3–3.1 min (35% B, increasing), 3.1–12 min (98% B, increasing), 12–14 min (98% B, isocratic), 14–14.1 min (10% B, decreasing), and 14.1–15 min (10% B, isocratic). The total run time was 15 min. The flow was set at 300 μL/min, and mobile phases (Fisher Scientific) were A, Optima HPLC-MS grade water, and B, Optima HPLC-MS grade acetonitrile, both with 0.1% Optima HPLC-MS grade formic acid. The column used was C18 Accucore Aq (150 mm × 2.1 mm, 2.6 μm, ThermoFisher).

Identification was made on a high-resolution HPLC-MS Q-Exactive Plus. First, an MS scan range set between 200 and 1100 *m*/*z* with a resolution of 70 000 full width at half-maximum (FWHM) in full-scan mode was applied. In addition to full-scan mode, extracted ion chromatogram (EIC) of specific chemical species reported in the literature, to be specific biodegradation products of punicalagin, was specifically searched (Table 2). Standard compounds from punicalagin, EA, and urolithins A and B were run and subjected to MS2 for positive identification (*m*/*z*—fragmentation pattern) of such compounds in the experimental samples. These analyses allowed us to obtain the retention time, as well as the molecular weight, which we confirmed on the database (PubChem). The tentative identification of other urolithins (specifically urolithin C and D), punicalin, and gallagic acid (also punicalagin degradation byproducts) was performed by searching for the ions shown in Table 2. Figure S1 shows the EIC for punicalagin, EA, and both urolithin A and B. Quantification of punicalagin, ellagic acid, and urolithins was made using calibration curves for each compound using standards (Sigma-Aldrich) in the same chromatographic and MS conditions as the experimental samples. Concentrations of species in such curves ranged from 1 to 100 μg/L for urolithins A and B and from 1 to 5000 μg/L for ellagic acid.

**Table 2:**
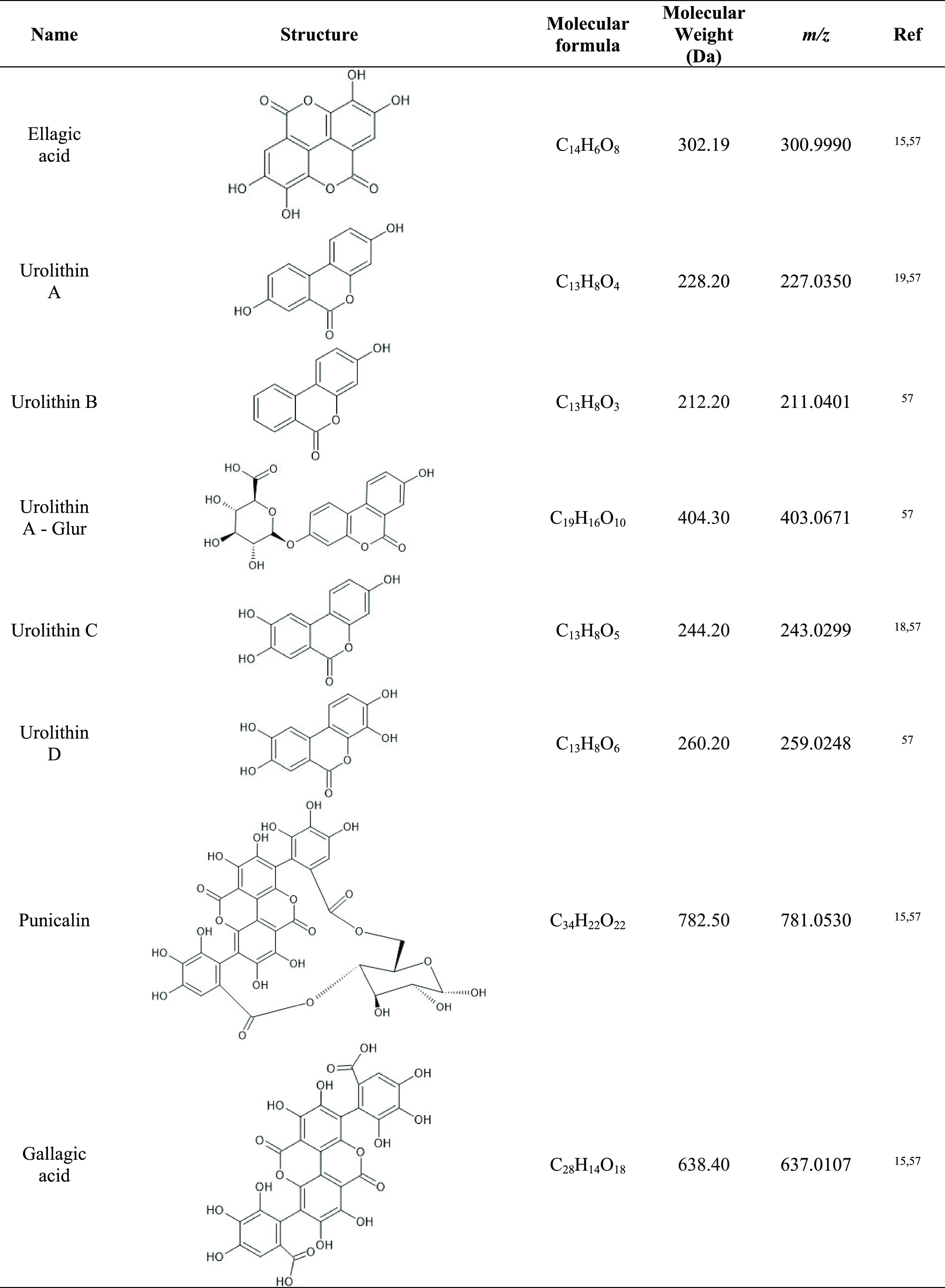
Punicalagin Byproducts Searched in Samples^15,18,19,57^

### Proteomic Analysis

Proteomic analysis was carried out according to the following protocol. Lysates were resuspended in phosphate buffer saline (PBS) and mixed with 5 μL of Laemmli loading dye buffer [Tris HCL 0.125 M, sodium dodecyl sulfate (SDS) 4% (w/v), glycerol 20% (v/v), 2-mercaptoethanol 10% (v/v), and bromophenol blue 0.004% (w/v)] and then were added to each sample. After 1 h of sonication in a Branson sonifier 250 bath (Emerson, Spain) to lysate cells, the samples were run in sodium dodecyl sulfate polyacrylamide gel electrophoresis (SDS-PAGE) 4% stacking/12% separating phases. When the proteins reached the separating phase, the run was stopped, and the gel was left to stain in Coomassie blue overnight. On the next day, destaining and washing procedures were done, and subsequently, the bands were picked up and cut into 1 mm^3^ pieces for in-gel digestion.^24^ After being trimmed, reduction and alkylation steps were performed. The former was carried out by adding a freshly prepared mix of 50 mM ammonium bicarbonate and 1,4-dithiothreitol (DTT) for 20 min at 56 °C. The latter was executed right after and consisted of the addition of a freshly prepared mix of iodoacetamide (IAA) and 50 mM ammonium bicarbonate, allowing the reaction to take place in absolute darkness for 15 min at room temperature. After that, proteins were digested into peptides using a mixture of proteaseMAX (Promega) and sequencing-grade trypsin (Promega). To achieve maximum effectiveness, the samples were left 1 h at 50 °C following the manufacturer’s instructions.

The resultant peptides, resuspended in loading buffer (water/acetonitrile 98:2 (v/v), 0.05% trifluoroacetic acid) for analysis, were sonicated in a water bath for 5 min and centrifuged at 12 700*g* for 15 min at room temperature right before setting them into conic vials for LC-MS/MS Orbitrap. Loading buffer was used to mimic the same conditions as the UHPLC method for proteomic analysis starts.

#### Label-Free Quantitative Proteomic Analyses

Mobile phases (Fisher Scientific) used were A, Optima HPLC-MS grade water, and B, Optima HPLC-MS grade acetonitrile, both with 0.1% Optima HPLC-MS grade formic acid. The column used was HPLC Acclaim PepMap 100 C18 (500 mm × 0.075 mm × 2 μm). Peptides were eluted with the following gradient, starting at 2% AcN: 0–3.1 min (8% B, increasing), 3.1–240 min (30% B, increasing), 240–241 min (90% B, increasing), 241–246 min (90% B, isocratic), 246–247 min (2% B, decreasing), and 247–277 min (2%, isocratic, equilibration phase). The total run time was 277 min. The flow was set at 0.300 μL/min.

Mass spectrometry was accomplished in the data-dependent mode. Data were collected using a Top15 method for MS/MS scans.^25^ Parameters were set as follows: spray voltage of 1.8 kV, capillary temperature of 300 °C, 390–1700 *m*/*z* for a full-scan mass range, and a resolution of 70 000 units.

Spectral normalization and comparative proteome abundance and data analysis were conducted using MaxQuant software.^26^

A Q-Exactive Plus mass spectrometer coupled to a Dionex Ultimate 3000 RSLCnano (Thermo Scientific, Waltham, MA) was used to analyze around 2 μg from each digest. Comparative proteome abundance and data analysis were carried out using MaxQuant software (version 1.6.15.0; https://www.maxquant.org/download_asset/maxquant/latest), and Perseus (v.1.6.15.0) was used to organize the data and perform statistical analysis. Carbamidomethylation of cysteines was set as a fixed modification; oxidation of methionines and acetylation of N-terminals were set as variable modifications. Database searching was performed against *E. faecium*, *L. plantarum*, or *L. paracasei* protein databases (www.uniprot.org). The FASTA files used were as follows: *E. faecium*—Taxon ID 1352 (https://www.uniprot.org/proteomes/UP000005269); *L. plantarum* (strain ATCC BAA-793/NCIMB 8826/WCFS1)—Taxon ID 220668 (https://www.uniprot.org/proteomes/UP000000432); *L. paracasei* (strain ATCC 334/BCRC 17002/CCUG 31169/CIP 107868/KCTC 3260/NRRL B-441)—Taxon ID 3219677 (https://www.uniprot.org/proteomes/UP000001651). The maximum peptide/protein false discovery rates (FDRs) were set to 1% based on a comparison to a reverse database. The LFQ algorithm was used to generate normalized spectral intensities and infer relative protein abundance.^26^ Proteins were identified with at least two peptides, and those proteins that matched to a contaminant database or the reverse database were removed and proteins were only retained in the final analysis if they were detected in at least two replicates from at least one treatment. Quantitative analysis was performed using a *t*-test to compare the B + P group with the B group. The qualitative analysis was also performed to detect proteins that were found in at least three replicates of a given treated group but were undetectable in the comparison B group and vice versa. All proteins satisfying one of the two aforementioned criteria are identified as “discriminating proteins”.

#### Gene Ontology Analysis

For enrichment analysis, the proteins were evaluated through ClueGO v.2.5.7.^27^ To define the term–term interrelations and functional groups based on shared genes between the terms, the Kappa score was established at 0.4. Three GO terms and 4% of genes covered were set as the minimum requirement to be selected. Deep search on the database (NCBI) searching for proteins with the same weight as the ones named by “extracellular glycosylase”, “cell-wall hydrolase”, “peptidoglycan hydrolase”, “uncharacterized protein”, etc., looking for most accurate proteins with a given biological function.

#### Statistical Analysis

When comparing ellagic acid productions by a variety of LAB in the presence of a punicalagin extract, we quantified each replicate and obtained an average with standard deviation. Analysis of variance (ANOVA) was applied to test the ability of bacteria to produce EA and urolithins from punicalagin. We also carried out a *t*-test to assess their significance vs C_2_ (MRS with extract).

The same data treatment was performed with the results of urolithin B since it is the only quantifiable urolithin above 1 μg/L.

For veracity treatment, we filtered out all of the nonsignificant proteins according to the *p*-value set for the software. The *p*-value was corrected by Bonferroni step-down and set as *p* ≤ 0.05.

## Results and Discussion

### Biotransformation of Punicalagin by Selected Probiotic Bacteria

**Figure 1:**
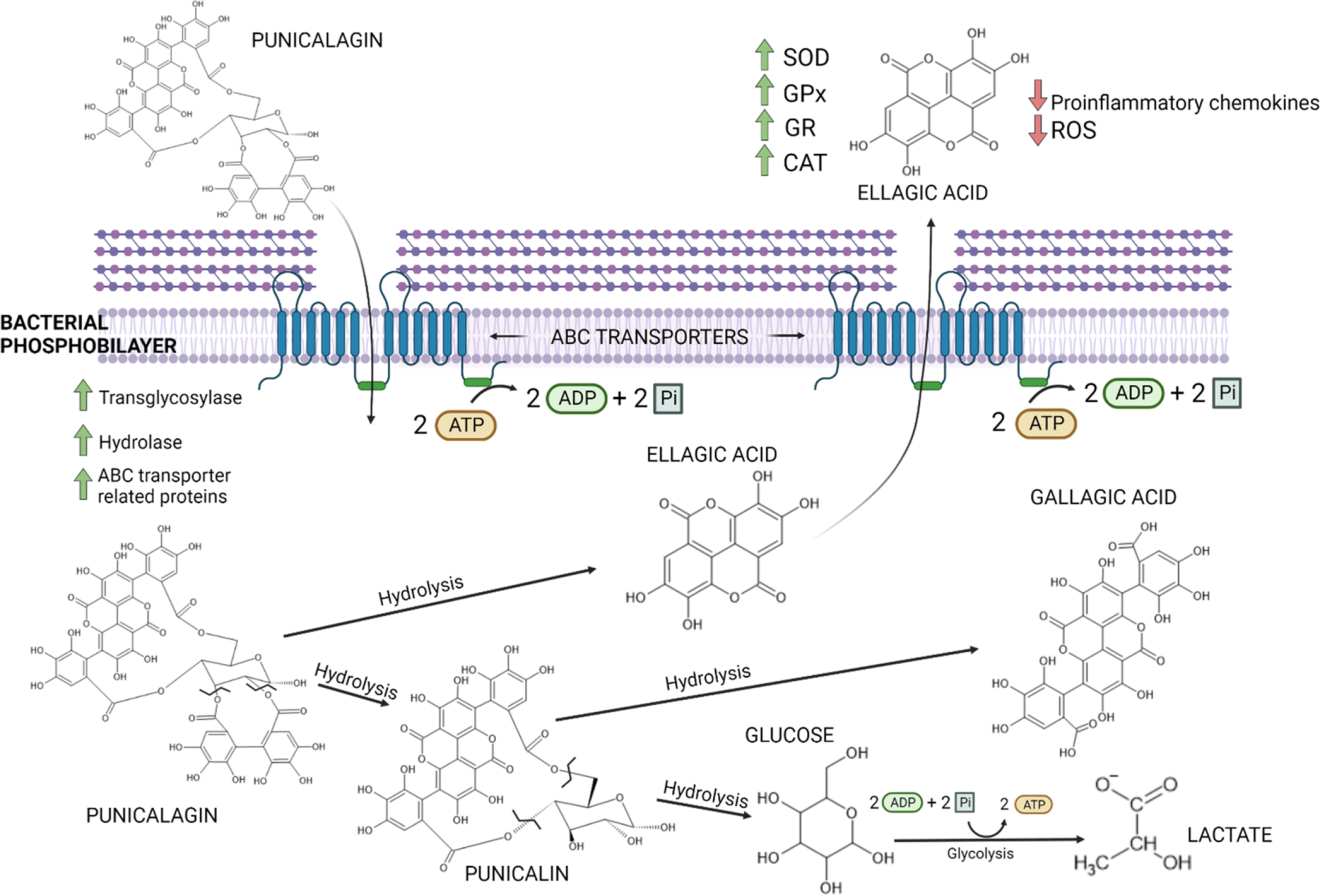
Proposed mechanisms of the bacterial utilization of punicalagin according to the proteomics study and following the fragmentation pattern proposed for this molecule as previously described by other authors.^28^

The culture medium where the LAB were incubated (C_1_ group) had a low quantity of EA (∼4 μg/L) that could plausibly be derived from trace plant ingredients. The addition of the punicalagin-rich pomegranate extract to the medium (C_2_) increased the concentration of EA up to 54.21 μg/L. The incubation of the LAB in the culture medium led to concentrations of EA between 9.11 and 49.38 μg/L. The combination of the punicalagin-rich supplement with the LAB under study (B + P group) led, in all cases, to significantly increased levels of EA compared to experimental units with the medium (C_1_), extract (C_2_), or the bacteria (B groups), alone (*p* < 0.05, Table 3). The accretion of EA in these experimental units was, in fact, highly remarkable, with such concentrations increasing between 25 and 18 times as compared to the concentration of EA found in the pomegranate extract. These results show the clear implication of LAB under study in the production of EA from its precursor, punicalagin. The biodegradation pathway previously reported by other authors^28^ is depicted in Figure 1. According to this mechanism, punicalagin would first be degraded to hexahydroxydiphenic acid (MW: 338.22 Da) and punicalin (MW: 782.5 Da) by hydrolysis. The former would spontaneously lactonize to ellagic acid (MW: 302.19 Da), and the latter would undergo another hydrolysis to form glucose (MW: 180.16 Da) and gallagic acid (MW: 638.39 Da). It is worth highlighting that other species such as complex tannins and gallocatechins could also have contributed to yielding ellagic acid in the present experiment. Yet, a detailed analysis of the extract revealed that punicalagin was, by far, the most abundant species in the pomegranate extract (31.13 mg/100 mg of powder), while other compounds were a minority, such as other ellagitannins (4.53 mg/100 mg powder) and anthocyanidins (0.04 mg/100 mg powder). Catechins were not detected in the present extract, neither by DAD nor by MS. It is therefore reasonable to consider that most of the ellagic acid was produced from punicalagin. The production of ellagic acid from precursors via acid hydrolysis could also have occurred, but the pH of the reaction media was monitored during the entire assay and never dropped below 4, far from the required acidic conditions for the chemical hydrolysis of punicalagin (∼2).

**Table 3:** Influence of Selected Lactic Acid Bacteria on the Concentrations of Ellagic Acid and Urolithins (Means ± Standard Deviation) in MRS Supplemented with 30 μg/mL Punicalagin after 24 h of Incubation at 37 °C^a^

sample	short name	urolithin A content (μg/L)	urolithin B content (μg/L)	ellagic acid content (μg/L)
culture medium (MRS)	*C1*	<1	<1	4.30d ± 5.34
culture medium + extract	*C2*	<1	<1	54.21c ± 17.78
*E. faecium* 37	*37_P*	<1	<1	1000.92b ± 116.74
*L. paracasei* 74	*74_P*	<1	<1	1401.06a ± 324.24
*L. plantarum* 89	*89_P*	<1	<1	1125.01ab ± 315.14
*L. casei* 116	*116_P*	<1	<1	1053.67ab ± 213.54
*E. faecium* 126	*126_P*	<1	<1	1115.34ab ± 164.98
*L. paracasei* 185	*185_P*	<1	<1	1063.33ab ± 374.75
*L. sakei* 195	*195_P*	<1	<1	979.74b ± 153.92
*L. casei* 246	*246_P*	<1	<1	1047.36ab ± 145.42
*L. plantarum* 284	*284_P*	<1	<1	1179.99ab ± 331.22
*L. plantarum* 295	*295_P*	<1	<1	1349.04a ± 385.20

a Culture medium (MRS, C_1_) and MRS plus punicalagin extract (C_2_) are used as controls. Different letters denote significant differences between means.

Although it has been poorly studied, the ability of LAB to produce ellagic acid from plant ellagitannin sources has already been documented. Tannin acyl hydrolase has been proposed to be the enzyme responsible for that biotransformation. The production of this hydrolase has been tested among a wide range of species isolated from grape must and wine, with *L. plantarum* being the only one with the ability to produce this enzyme.^29^ In line with this finding, other authors found that *L. plantarum* was able to produce EA from ellagitannins, enhancing, as a result, the antioxidant action of such phytochemicals in vitro, when recreating gastrointestinal conditions (37 °C, for 3 h)^30^ and alongside MRS (30 °C, 5 days).^20,21^

Pomegranate derivatives have gained a huge interest in the last 20 years. It has been proven that there are other microorganisms, such as *Aspergillus niger*, with the ability to transform ellagitannins into EA.^31^ As far as we are concerned, this is the first time that *L. sakei*, *L. mesenteroides*, *E. hirae*, *L. garvieae*, *L. casei*, and *E. durans* are tested for the in vitro biodegradation of ellagitannins and the first time that *Lacticaseibacillus paracasei*,^32^*E. faecium,*^33^ and *L. lactis*(33) are tested for punicalagin degradation, specifically. Therefore, the results from the present study confirm the ability of certain LAB to facilitate the biodegradation of punicalagin into EA and identify specific strains with particular potential in the formation of bioactive compounds. This finding is of remarkable scientific interest as the occurrence of Lactobacilli as well as *Enterococcus* spp. in the small intestine would contribute to degrading dietary punicalagin into EA that could, in turn, be later absorbed or transformed into urolithins by other bacteria in the colon.

Table 3 shows the concentration of EA in the medium in which all strains were tested. Among all, *L. paracasei* 74 showed a better conversion rate of punicalagin into EA (4.67%), followed by *L. plantarum* 295 (4.50%) and *L. plantarum* 284 (3.93%). Despite achieving a higher transformation ratio than our bacterium selected to conduct proteomic analyses (*L. plantarum* 89, 3.75%; *E. faecium* 126, 3.72%), these bacteria were not selected due to the disparity between the results on ellagic acid content between the samples that were tested in the assay. Bacteria with the lowest conversion rates were *E. faecium* 37 and *L. sakei* 195 (3.34 and 3.27%, respectively), yet their numbers were also highly remarkable. Our results indicate that the ability of LAB to metabolize punicalagin from a pomegranate extract into ellagic acid could be a strain-specific ability, but further research has to be done on that topic. While the production of EA in the experimental units is noticeable, the conversion rates indicate that a relatively small percentage of punicalagin was eventually converted. This could be explained by the imbalance between the concentration of precursor and the bacteria counts and/or the high affinity of punicalagin to bind biomolecules (i.e., proteins), which would hinder its degradation by bacteria.^34^

Other authors conducted similar experiments using pomegranate juice (PJ) as a source of ellagitannins.^20,21^ PJ is made by the extrusion of arils, and it seems that the quantity of punicalagin in arils (4100 ± 200 μg/L) is significantly lower than that in the peel or mesocarp (10 543.4 ± 468.0 and 20 314.8 ± 701.0 mg/kg, respectively).^15^ Yet, the quantity of punicalagin in PJ is highly variable as it depends on the cultivar and on the parts of the fruit employed in the extraction process, either the arils or the whole fruit. The quantity of punicalagin aril pomegranate juice ranges from 4100 to 233 000 μg/L, and in whole fruit pomegranate juice ranges from 166 000 to 800 000 μg/L.^35^ Taking this into account, the production of ellagic acid when fermenting PJ with assorted *L. plantarum* strains (from 6400 ± 200 to 7100 ± 300 μg EA/L) vs control (no bacteria) (4900 ± 200 μg EA/L) was not as impressive as the numbers obtained in this research.^20^ However, the quantity of punicalagin was not measured in that research, so we are not able to calculate the conversion rate from punicalagin to ellagic acid. However, given that the punicalagin concentration in PJ produced from certain varieties of pomegranate arils reaches 233 000 μg/L, an increase of 1500 μg/L in EA is not as noticeable as our average 1131.55 μg/L increase starting from 30 000 μg/L of punicalagin. It also deserves to be highlighted that the fermentation conditions reported in that previous study differ from those in our research in terms of time (120 vs 24 h) and temperature (30 vs 37 °C). Due to this fact, in our experiment where an extract made by pomegranate mesocarp was incubated, the concentration of EA reached up to 20 times more when fermented than that of the extract that was not exposed to a probiotic 1131.55 ± 329.10 vs 54.21 ± 17.78 μg/L.

The pomegranate food supplement used in the present study was produced from mesocarp, and that explains the higher concentration of punicalagin (300 mg/g) in the reaction medium compared to that reported in other studies in which PJ was used. Furthermore, our research was made using a fraction of the pill used as a supplement to avoid possible solubility problems in the final volume used for each batch (15 mL). The whole pill had 183 mg of punicalagin on average, which means that eating this extract would confer the same benefits as drinking a full liter of PJ in terms of antioxidant activity. In addition, other ellagitannins are also present in pomegranate extracts and will add differences in the quantity of precursors of the ellagic acid released.^36^ These results emphasize the relevance of using punicalagin-rich plant sources to perform an efficient and biologically relevant biosynthesis of EA. Furthermore, considering that such materials are typically nonedible tissues from fruits, using such waste materials is of both economic and environmental interest.

While both punicalagin, like other ellagitannins, and EA display antioxidant properties, the low bioavailability and poor absorption of the former make the biotransformation of punicalagin into EA a key biochemical transformation in terms of intestinal uptake and bioactivity.^37^ EA has, in fact, a better absorption in the gut than punicalagin.^38^ EA provides protection against oxidative stress due to its radical scavenging activity and its ability to promote the synthesis and activity of antioxidant enzymes such as superoxide dismutase (SOD), glutathione peroxidase (GPx), glutathione reductase (GR), and catalase (CAT).^39,40^ In addition to this antioxidant property, EA displays anti-inflammatory activity by modulating the formation of proinflammatory cytochemokines (IL-6, TNF-α, IL-8, IL-12, etc.).^40,41^ There is some therapeutical potential attributed to EA when administered in cancer or in combination with cancer treatments,^40^ but more clinical studies have to be carried out.

While the ability of these LAB to produce EA from pomegranate ellagitannins is proven in the present study, the implication of the bacteria in the biotransformation of EA into urolithins was not observed in the present experiment. Neither urolithin A nor urolithin B was found in the conditions of the present experiment. In contrast, in human trials, 10.61 μg/mL urolithins were found in plasma after 6 h from the intake of 1.77 mg/mL EA,^42^ which indicates a 0.6% conversion rate. Hence, it is obvious that even if the LAB under study may facilitate the initial degradation of punicalagin into EA, other common components of human microbiota of the Eggerthellaceae family, such as *Gordonibacter* and *Ellagibacter*, are needed for the subsequent biotransformation of EA into urolithins, as previously reported.^18,19^ The formation of these bioactive species involves an additional relevant step in the biodegradation of ellagitannins as urolithins have been found to be highly bioavailable and display further health-promoting activities.^16^ Under physiological conditions, the EA escaping from ileal absorption continues its path through the GIT where it is metabolized into urolithins, as we already discussed in the Introduction section.^18,19^

According to the present results, the combination of dietary pomegranate with a probiotic bacterium (i.e., *L. plantarum*) could be an efficient means to guarantee the degradation of punicalagin into bioactive compounds with potential benefits on human health. A recent systematic review^43^ gathered all of the data available regarding the antioxidant activity and health benefits provided by pomegranate consumption. Dietary pomegranate enables a significant reduction of plasma malonaldehyde (MDA), a lipid peroxidation biomarker, in patients with any kind of pro-oxidative disease when treated for 8 or more weeks.^43^ Inversely, glutathione peroxidase (GPx) increases when unhealthy people were treated, and it lasted for more than 8 weeks.^43^ An increase in the total antioxidant capacity (TAC) and superoxide dismutase (SOD) activity in plasma was observed.^43^^43^ Oxidative stress has been linked to many diseases such as hypertension, atherosclerosis, cardiovascular disease, or cancer.^44^ The occurrence of the biodegradation described in the present study under physiological conditions as well as the bioactive effects in vivo requires further research.

To shed light on the mechanisms involved in this transformation by LAB, bacteria cocultured in this analysis were analyzed by comparative proteomics to find out which proteins were over- or underexpressed and see if there is any metabolic pathway affected to face the presence of punicalagin in the medium.

### Influence of Punicalagin-Rich Pomegranate Supplement on Lactic Acid Bacteria Proteome

#### General Overview of the Effect of Punicalagin on Bacterial Biological Processes

Spectral data were analyzed by MaxQuant software, enabling the acquisition and comparison of the proteomes between B and B + P groups for the selected bacteria. Among all of the strains assessed, *L. plantarum* 89, *L. paracasei* 126, and *E. faecium* 74 were chosen due to their use as a probiotic in many treatments, especially the first one. Strains from all three of these species have been proven to be a helpful tool in diminishing inflammation in diseases such as ulcerative colitis or irritable bowel syndrome.^45−48^ The proteins, from the three strains evaluated, exhibited higher relative abundance in comparison with their counterparts from the B group, with statistically significant differences (presented in Table 4). After that, data were processed with GeneOntology enrichment software, ClueGo.

**Table 4:** Proteins Found in Higher Relative Quantity among Three Strains with Statistical Significance (*p* < 0.05)

*E. faecium*		*L. paracasei*		*L. plantarum*	
protein name	Log_2_ fold change	protein name	Log_2_ fold change	protein name	Log_2_ fold change
phosphocarrier protein HPr	2.067	uncharacterized protein encoded in the toxicity protection region of plasmid R478 contains the von Willebrand factor (VWF) domain	2.338	extracellular transglycosylase	1.682
50S ribosomal protein L36	1.968	uncharacterized protein	2.168	50S ribosomal protein L35	1.496
ribonucleoside-diphosphate reductase subunit β	1.822	septum formation initiator	2.167	extracellular transglycosylase	1.491
peptidoglycan hydrolase	1.686	cell wall-associated hydrolase	2.042	citrate transport protein	1.397
NlpC/P60 family lipoprotein	1.545	uncharacterized protein	1.993	50S ribosomal protein L33	1.378
carbamoyl-phosphate synthase large chain	1.527	cell wall-associated hydrolase	1.912	extracellular transglycosylase, membrane-bound	1.254
BglG family transcription antiterminator	1.487	protein RecA	1.897	extracellular transglycosylase, with LysM peptidoglycan-binding domain	1.211
DNA-binding response regulator	1.460	WxL domain-containing protein	1.814	glutathione reductase	1.206
*N*-acetylmuramoyl-l-alanine amidase	1.313	surface antigen	1.755	extracellular transglycosylase, with LysM peptidoglycan-binding domain	1.175
GTP diphosphokinase	1.310	predicted outer membrane protein	1.674	serine-type d-Ala-d-Ala carboxypeptidase	1.165
protein RecA	1.275	uncharacterized protein	1.627	30S ribosomal protein S20	1.105
ribosome-recycling factor	1.274	cell wall-associated hydrolase	1.551	extracellular protein, NlpC/P60 family.γ-d-glutamate-*meso*-diaminopimelate muropeptidase	0.948
30S ribosomal protein S21	1.240	surface antigen	1.502	NADH oxidase	0.930
ribonucleoside-diphosphate reductase	1.177	50S ribosomal protein L23	1.475	anaerobic ribonucleoside-triphosphate reductase	0.920
peptidoglycan hydrolase	1.157	energy-coupling factor transporter ATP-binding protein EcfA2	1.429	RNA-binding protein, the YhbY family	0.914
mannosyl-glycoprotein endo-β-*N*-acetylglucosamidase	1.155	NusG_II domain-containing protein	1.313	DNA-entry nuclease	0.845
DNA gyrase subunit A	1.149	predicted membrane protein	1.304	50S ribosomal protein L29	0.844
DNA-binding response regulator	1.134	uncharacterized protein	1.243	deoxyadenosine kinase/deoxyguanosine kinase	0.815
chromosome partitioning protein ParB	1.069	uncharacterized protein	1.208	30S ribosomal protein S16	0.799
leucine–tRNA ligase	0.962	cell surface protein	1.189	nucleoside 2-deoxyribosyltransferase	0.789
50S ribosomal protein L18	0.954	lactocepin I, serine peptidase, MEROPS family S08A	1.153	50S ribosomal protein L36	0.751
ABC superfamily ATP-binding cassette transporter, ABC protein	0.947	β-*N*-acetylhexosaminidase	1.147	pyruvate oxidase	0.749
50S ribosomal protein L29	0.944	uncharacterized protein	1.132	pseudouridine synthase	0.730
ribose-5-phosphate isomerase A	0.940	β-fructosidase (levanase/invertase)	1.124	50S ribosomal protein L32	0.726
50S ribosomal protein L11	0.929	cyclic-di-AMP phosphodiesterase	1.114	large-conductance mechanosensitive channel	0.707
50S ribosomal protein L14	0.929	50S ribosomal protein L33	1.089	tRNA modification GTPase MnmE	0.692
UvrABC system protein A	0.914	DD-transpeptidase	1.086	peroxidase	0.686
ATP-dependent Clp protease ATP-binding subunit ClpX	0.902	*N*-acetylmuramoyl-l-alanine amidase	1.083	ferredoxin–NADP reductase	0.681
DNA topoisomerase 4 subunit B	0.859	β-lactamase class C-related penicillin-binding protein	1.074	uncharacterized protein	0.679
transcription termination factor Rho	0.813	uncharacterized protein	1.067	single-stranded DNA-binding protein	0.677
50S ribosomal protein L19	0.806	30S ribosomal protein S20	1.061	extracellular protein, the DUF1002 family	0.662
glutamine–fructose-6-phosphate aminotransferase [isomerizing]	0.781	50S ribosomal protein L30	1.032	extracellular protein, the DUF2140 family	0.657
KR domain-containing protein	0.775	WxL domain-containing protein	0.997	cell wall hydrolase/muramidase	0.652
Bifunctional oligoribonuclease/PAP phosphatase NrnA	0.762	acyl carrier protein	0.989	sortase A	0.652
UvrABC system protein B	0.732	peptidoglycan transpeptidase; the ErfK-YbiS-YhnG family	0.978	ABC transporter, ATP-binding protein	0.626
DNA-binding protein HU	0.723	ATPase component of ABC transporter with duplicated ATPase domains	0.965	extracellular transglycosylase with the LysM peptidoglycan-binding domain	0.586
redox-sensing transcriptional repressor Rex	0.672	amino acid/polyamine/organocation transporter. APC superfamily	0.942	deoxynucleoside kinase	0.573
peptide chain release factor 3	0.656	predicted secreted protein	0.936	adherence protein, chitin-binding domain	0.571
30S ribosomal protein S15	0.618	transcriptional regulator	0.924	extracellular protein, membrane-anchored	0.570
asparaginase	0.592	uncharacterized protein	0.922	translation initiation factor IF-1	0.568
50S ribosomal protein L13	0.580	phospholipase A2 family enzyme	0.921	extracellular protein, γ-d-glutamate-*meso*-diaminopimelate muropeptidase	0.561
ribosome-binding ATPase YchF	0.578	predicted xylanase/chitin deacetylase	0.921	cell surface protein. membrane-anchored	0.549
valine–tRNA ligase	0.521	DUF4430 domain-containing protein	0.866	kojibiose-like phosphorylase. specific	0.549
FMN-binding domain-containing protein	0.517	acetoin/pyruvate dehydrogenase complex, E3 component, dihydrolipoamide dehydrogenase	0.861	nitroreductase family protein	0.539
ATP-dependent DNA helicase	0.504	predicted membrane protein	0.860	Fe-S_biosyn domain-containing protein	0.533
30S ribosomal protein S20	0.496	uncharacterized protein	0.860	extracellular protein	0.531
DNA gyrase subunit B	0.495	TPR repeats containing protein	0.814	thymidylate synthase	0.524
30S ribosomal protein S12	0.468	β-propeller domains of methanol dehydrogenase type	0.800	phosphoglycerate mutase family protein	0.520
DNA-directed RNA polymerase subunit β	0.454	lyase_8_N domain-containing protein	0.790	oligopeptide ABC transporter, permease protein	0.515
infA	0.435	glycerophosphoryl diester phosphodiesterase	0.788	lipoprotein, FMN-binding protein	0.513
probable succinyl-diaminopimelate desuccinylase	0.409	ABC-type Na^+^ efflux pump, permease component	0.765	acyl-[acyl-carrier protein] thioesterase	0.501
DUF1797 domain-containing protein	0.378	transcriptional regulator	0.741	preprotein translocase, the YajC subunit	0.489
acyl carrier protein	0.364	uncharacterized protein	0.728	rod-shape-determining protein	0.485
glutamate–tRNA ligase	0.359	amino acid ABC transporter membrane protein, PAAT family/amino acid ABC transporter substrate-binding protein; PAAT family	0.712	transcription regulator, the LysR family	0.482
formamidopyrimidine-DNA glycosylase	0.346	trypsin-like serine protease with the PDZ domain	0.702	spermidine/putrescine ABC transporter, substrate-binding protein	0.481
GTPase Obg	0.345	DNA-directed RNA polymerase subunit omega	0.693	ABC transporter, ATP-binding protein	0.475
30S ribosomal protein S10	0.301	uncharacterized protein	0.682	acetaldehyde dehydrogenase	0.469
pyrrolidone-carboxylate peptidase	0.301	α-glucosidase, family 31 of glycosyl hydrolase	0.672	ribonucleoside-diphosphate reductase	0.465
septation ring formation regulator EzrA	0.280	ABC-type uncharacterized transport system, periplasmic component	0.671	ABC transporter, substrate-binding protein	0.461
UDP-glucose 4-epimerase	0.241	ABC-type oligopeptide transport system, periplasmic component	0.663	ABC transporter, substrate-binding protein	0.460
DNA-directed RNA polymerase subunit α	0.199	transcriptional regulator	0.621	RNA-binding protein	0.459
GMP synthase [glutamine-hydrolyzing]	0.196	uncharacterized protein	0.607	probable cell wall amidase lytH	0.458

For *L. plantarum* 89, 1021 proteins were identified, among which 362 showed a significant (*p* < 0.05) increase when exposed to punicalagin (Table S1). Seven of these proteins were only found when bacteria were incubated with the phytochemical (B +P vs B). The distribution of all of these proteins after gene ontology enrichment is shown in Figure S2. Briefly, the most represented group is the non-membrane-bound organelle (48% of the proteins), followed by anion binding (20%) and cellular component organization (14%).

Although there are different locations/functions given by the software, there are some proteins that are settled in two or more of these groups. For instance, serine-type d-Ala-d-Ala carboxypeptidase, which is found to be one with a higher fold change in B + P with respect to B, belongs to the group of non-membrane-bound organelle, cellular component organization, and carbohydrate synthesis process. Glutathione reductase belongs to anion binding and flavin adenine dinucleotide binding. All ribosomal proteins are located in the non-membrane-bound organelle as they should. ATP-binding cassette (ABC) transporters are included mainly in anion binding and intrinsic components of the membrane. Extracellular transglycosylases were the main group that was increased by exposure to punicalagin, but they were not associated with any of the given cellular functions.

Eighty-three out of 1021 were diminished in B + P, while five of them were only present in B (Table S1).

All of these proteins with a significant decrease were grouped mainly into the pyrimidine ribonucleotide metabolic process (85.0%). Metal ion binding, hydrolase activity, uracil phosphoribosyltransferase, and cytoplasm activity-related enzymes were also diminished in this fermentation.

In *L. paracasei* 126, 939 proteins were identified (Table S2), of which 122 were significantly increased in B + P compared to the B counterpart (Table 4). In this case, there were no proteins identified only in the B + P samples. The majority of those 122 proteins were grouped as serine-type peptidase activity (Figure S3). Some of the proteins with the most variation between groups could not be identified by the software and were labeled as “uncharacterized proteins”. By searching on a database by their molecular weight, we found out that they might be associated with cell division or catalytic function (hydrolase, peptidase, hydrogenase, etc.). Interestingly, some cell wall-associated hydrolases were found to be increased in *L. paracasei* when challenged with punicalagin. Their function might be similar to that of the transglycosylases described in *L. plantarum*. As well as in the previous probiotic, the ribosomal proteins in *L. paracasei* were also affected probably due to their association with an increase in the growth rate. A really interesting protein associated with anti-inflammatory properties is lactocepin, which has been found in *Lactobacillus* genus.^49^ The occurrence of punicalagin in the medium enhanced the production of this bioactive protein by *L. paracasei*.

One hundred and seventy-one out of 939 were diminished in B + P, while 37 of them were only present in B samples (Table S2).

Those proteins were classified as nucleotide metabolic process (37.5%), organic acid metabolic process (18.75%), nucleotide binding (15.62%), and organonitrogen compound metabolic process (14.06%) predominantly.

For *E. faecium* 74, 399 proteins were identified, among which 62 had significant increases vs the B group. Only one protein was found in the B + P groups and not found in the B counterpart (Table S3). Those discriminating proteins were classified, as shown in Figure S4. Non-membrane-bound organelle was the group with more proteins affected (52.17%), closely followed by DNA topological change (45.65%) and with a little reminiscent of nucleotide-excision repair (2.17%; Figure S4). Among the ones with increased production in the presence of punicalagin, there are some peptidoglycan/murein hydrolases, showing that even though it belongs to another genus, the proteins in charge of breaking those bonds are still increased.

A protein highlighted in this analysis is phosphocarrier protein HPr, found in many bacteria to be an essential component of the sugar-transporting phosphotransferase system and playing a role in carbohydrate metabolization by interacting with enzymes dedicated to do so.^50^ Those proteins found in higher quantities were classified, as shown in Figure S4.

One hundred and fifty-three out of 399 proteins were diminished in B + P, while 41 of them were only present in B (Table S3).

Those proteins were grouped into the organonitrogen compound metabolic process (29.17%), organic acid metabolic process (24.17%), peptide biosynthetic process (20%), and nucleotide binding (9.17%) mainly. Three of them were also present in *L. paracasei* diminished pool of proteins.

On our proteomic analysis, at first sight, we could confirm that the presence of the extract has promoted the proliferation and faster-growing pace of the bacteria selected in vitro, as other colleagues tested beforehand.^51^ A huge increase in a wide variety of ribosomal proteins, known to translate RNA into proteins, was observed (Table 4).

#### Implication of Specific Proteins in Punicalagin Metabolism

The degradation of punicalagin into bioactive components requires the implication of bacterial enzymes such as the ellagitannin acyl hydrolase, which takes part in the metabolism of ellagitannins.^52^ However, this specific protein was not found in the proteome of the selected bacteria. Hence, it is obvious that other hydrolytic enzymes would be able to degrade punicalagin and hence explain the remarkable increase in EA found in the present study. In fact, some proteins with generic names associated with hydrolyzation might be plausible candidates implicated in this relevant pathway. Proteins potentially involved in the punicalagin hydrolysis are shown in Table 5. As we already showed from our substantial protein database, some transglycosylase/cell wall hydrolases have been found in every genus assessed. Certain lytic transglycosylases are capable of breaking cell walls to expand or reshape this cell structure after a cell division. It is reasonable to consider that these enzymes, in addition to catalyzing the scission of β-1,4 bonds between murein monomers, could have a role in hydrolyzing such bonds between the aromatic rings and the glucose present in punicalagin. This could potentially release hexahydroxydiphenic acid (HHDP) into the medium, a molecule that due to its better stability would be transformed into EA^53^ (Figure 1).

**Table 5:** Proteins Identified Suspects of Being Involved in Punicalagin Hydrolysis^a^

*E. faecium*		*L. paracasei*		*L. plantarum*	
protein name	Log_2_ fold change	protein name	Log_2_ fold change	protein name	Log_2_ fold change
peptidoglycan hydrolase	1.686	cell wall-associated hydrolase	2.042	extracellular transglycosylase	1.682
*N*-acetylmuramoyl-l-alanine amidase	1.313	cell wall-associated hydrolase	1.912	extracellular transglycosylase	1.491
peptidoglycan hydrolase	1.157	cell wall-associated hydrolase	1.551	extracellular transglycosylase membrane-bound	1.254
mannosyl-glycoprotein endo-β-*N*-acetylglucosamidase	1.155	*N*-acetylmuramoyl-l-alanine amidase	1.083	extracellular transglycosylase with LysM peptidoglycan-binding domain	1.211
		peptidoglycan transpeptidase, the ErfK-YbiS-YhnG family	0.978	extracellular transglycosylase with the LysM peptidoglycan-binding domain	1.175
				extracellular protein, the NlpC/P60 family, γ-d-glutamate-*meso*-diaminopimelate muropeptidase	0.948
				cell wall hydrolase/muramidase	0.652
				extracellular transglycosylase with the LysM peptidoglycan-binding domain	0.586
				extracellular protein, γ-d-glutamate-*meso*-diaminopimelate muropeptidase	0.561
				probable cell wall amidase lytH	0.458

a Fold change with respect to their nontreated counterpart groups (*p* < 0.05).

Moreover, in *E. faecium*, we identified mannosyl-glycoprotein endo-β-*N*-acetylglucosamidase, which has been proven to be responsible for breaking oligosaccharides in the cytosol.^54^ Additionally, the enzyme γ-d-glutamate-*meso*-diaminopimelate muropeptidase, associated with the probiotic *L. plantarum*, has been identified as cell wall hydrolase and glutamic acid hydrolase.^55^

To the best of our knowledge, this is the first evaluation of the impact of a potential bioactive compound in LAB proteome, which further transforms it into a more bioavailable compound. The results obtained point to the higher quantity of proteins with glycolytic activity in three quite different species, which are seemingly working by degrading punicalagin into less-complex substances. In this sense and considering the relatively high size of punicalagin, it is not expected to be uptaken without the requirement of any bacterial transporter machinery. We found a higher quantity of ATP-binding cassette (ABC) transporter-related proteins in all three strains when exposed to punicalagin. ABC transporters are ATP-mediated membrane proteins whose role is no other than transferring molecules through the cell wall. The main ABC transporters found are displayed in Table 6.

**Table 6:** ABC Transporter-Related Proteins Found in All Three Bacteria Tested^a^

*E. faecium*		*L. paracasei*		*L. plantarum*	
protein name	Log_2_ fold change	protein name	Log_2_ fold change	protein name	Log_2_ fold change
ABC superfamily ATP-binding cassette transporter ABC protein	0.947	ATPase component of ABC transporter with duplicated ATPase domains	0.965	ABC transporter. ATP-binding protein	0.626
UvrABC system protein A	0.914	ABC-type Na^+^ efflux pump, permease component	0.765	oligopeptide ABC transporter, permease protein	0.515
UvrABC system protein B	0.732	amino acid ABC transporter membrane protein, PAAT family/amino acid ABC transporter substrate-binding protein, the PAAT family	0.712	spermidine/putrescine ABC transporter substrate-binding protein	0.481
		ABC-type uncharacterized transport system, periplasmic component	0.671	ABC transporter. ATP-binding protein	0.475
		ABC-type oligopeptide transport system, periplasmic component	0.663	ABC transporter, substrate-binding protein	0.461
				ABC transporter, substrate-binding protein	0.460

a Fold change with respect to their nontreated counterpart groups (*p* < 0.05).

As research shows,^56^ these transporters import nutrients, export molecules, and play a role in many other cell functions. It could be possible that to get energy from it, they import punicalagin, degrade it, and export ellagic acid to avoid possible antimicrobial activity.

Our hypothesis is that the presence of a new compound that is unable to pass through the membrane by passive diffusion may increase the need for the cells to create new bridges to transfer it into their cytoplasm. Thus, although further studies are required to confirm this asseveration, the higher quantity of this type of protein, with a quite specific function in the three different tested species, suggests a key role of these proteins in the punicalagin biotransformation by LAB, as shown in Figure 1.

In conclusion, this work has demonstrated the ability of 30 different LAB to transform a complex phytochemical with low bioavailability, punicalagin, into simpler molecules, including the bioavailable ellagic acid. This enables a rational procedure to transform punicalagin from a byproduct, pomegranate mesocarp, into a useful molecule. The most efficient way of application, in vivo or in vitro, should be further explored. Additionally, molecular mechanisms underlying this biotransformation by LAB are originally reported through label-free comparative proteomics. This approach has shed light on the way that punicalagin is transformed, pointing to two potential mechanisms involved: degradation by hydrolytic enzymes and intake/export through ABC transporters. The lack of degradation of EA into urolithins emphasizes the necessity of the implication of other bacteria from microbiota to synthesize these bioactive species in vivo.

## Supporting Information Available

Extracted ion chromatogram (EIC) of standard compounds punicalagin (*m*/*z* 1083.0593), ellagic acid (*m*/*z* 300.9990), and both urolithin A (*m*/*z* 227.0350) and B (*m*/*z* 211.0401) (Figure S1); distribution by function of proteins found in higher relative quantity in *L. plantarum* in the presence of 30 μg/mL punicalagin (Figure S2); distribution by function of proteins found in higher relative quantity in *L. paracasei* in the presence of 30 μg/mL punicalagin (Figure S3); distribution by function of proteins found in higher relative quantity in *E. faecium* in the presence of 30 μg/mL punicalagin (Figure S4); proteins identified in this study for *L. plantarum* 89 along with Log_2_ fold change (in blue) and significance values (*p*, in red) of the 30 μg/mL punicalagin (P) treatment in comparison to the nontreated control (B) (Table S1); proteins identified in this study for *L. paracasei* 126 along with Log_2_ fold change (in blue) and significance values (*p*, in red) of the 30 μg/mL punicalagin (P) treatment in comparison to the nontreated control (B) (Table S2); proteins identified in this study for *E. faecium* 74 along with Log_2_ fold change (in blue) and significance values (*p*, in red) of the 30 μg/mL punicalagin (P) treatment in comparison to the nontreated control (B) (Table S3). (PDF)

## Abbreviations

LAB: lactic acid bacteria
ABC transporters: ATP-binding cassette transporters
GIT: gastrointestinal tract
EA: ellagic acid
PJ: pomegranate juice
