# Supplementary material for: Biodegradation of Punicalagin into Ellagic Acid by Selected Probiotic Bacteria: A Study of the Underlying Mechanisms by MS-Based Proteomics
Source: J Agric Food Chem. 2022 Dec 15;70(51):16273–85. doi: 10.1021/acs.jafc.2c06585 (PMC9801417; doi:10.1021/acs.jafc.2c06585)
Supplement: Supplementary file 1 — jf2c06585_si_001.pdf [file jf2c06585_si_001.pdf]

Biodegradation of punicalagin into ellagic acid by selected probiotic bacteria: study of the underlying mechanisms by MS-based proteomics

Víctor Caballero[1,2], Mario Estévez[1]*, Francisco A. Tomás-Barberán[3], David Morcuende[1], Irene Martín[2], Josué Delgado[2]

[1] Food Technology, IPROCAR Research Institute, University of Extremadura, 10003, Cáceres. Spain.

[2] Food Hygiene and Safety, IPROCAR Research Institute, University of Extremadura, 10003, Cáceres. Spain.

[3] Research Group on Quality, Safety and Bioactivity of Plant-derived Foods, CEBAS- CSIC, Murcia, 30100, Spain.

* Corresponding author:
Mario Estévez (mariovet@unex.es)
Tel. +34927257100 (Ext. 51390)
Fax. +34927257110
Department of Animal Production and Food Science, Food Technology,
University of Extremadura, 10003, Spain.

SUPPORTING INFORMATION

Figure S1.Extracted ion chromatogram (EIC) of standard compounds (A) Punicalagin (*m/z* 1083.0593), (B) ellagic acid (*m/z* 300.9990) and (C) both urolithin A (*m/z* 227.0350) and B (*m/z* 211.0401)

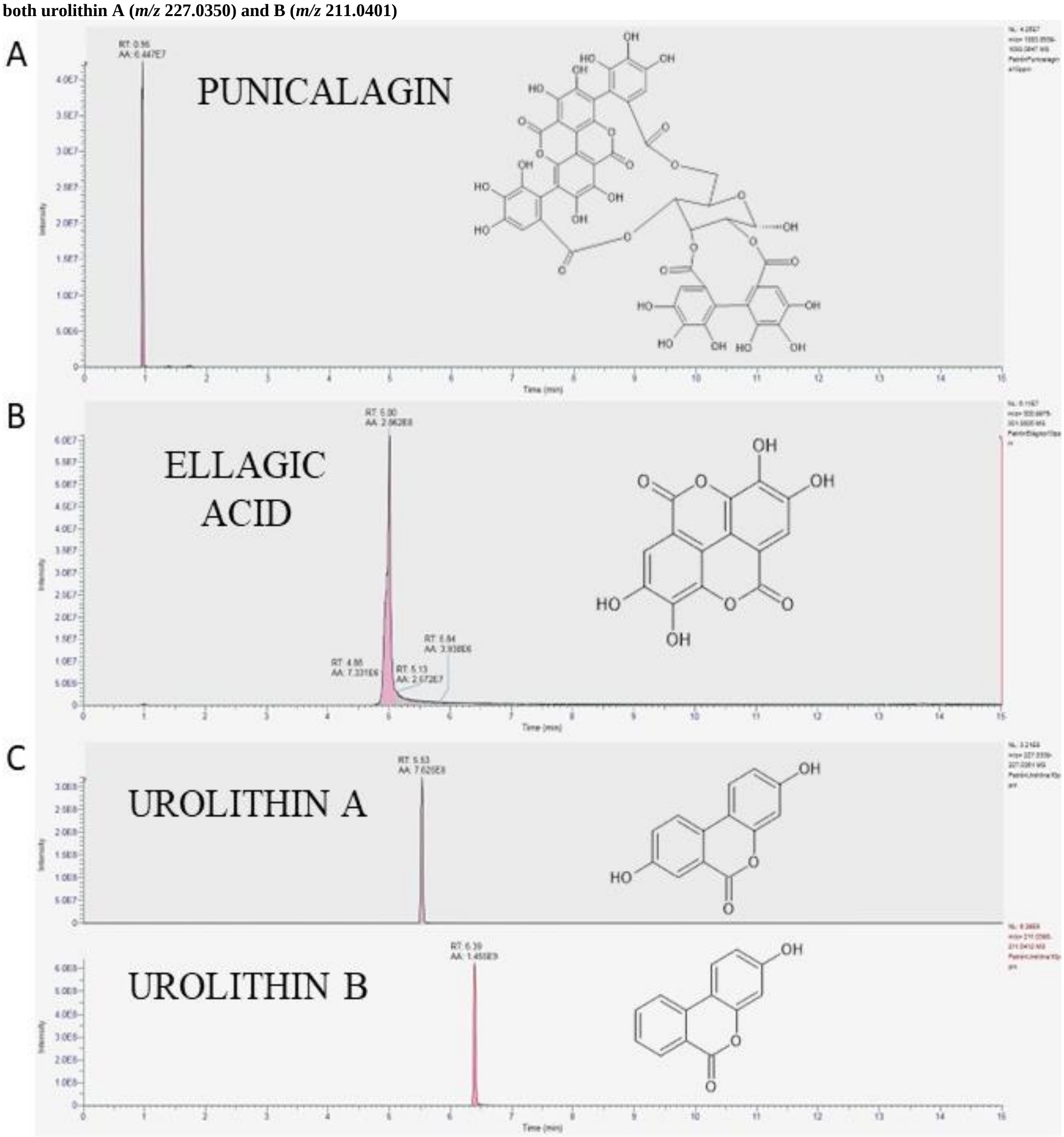

Figure S2. Distribution by function of proteins found in higher relative quantity in *L. plantarum* in presence of 30 µg/mL of punicalagin.

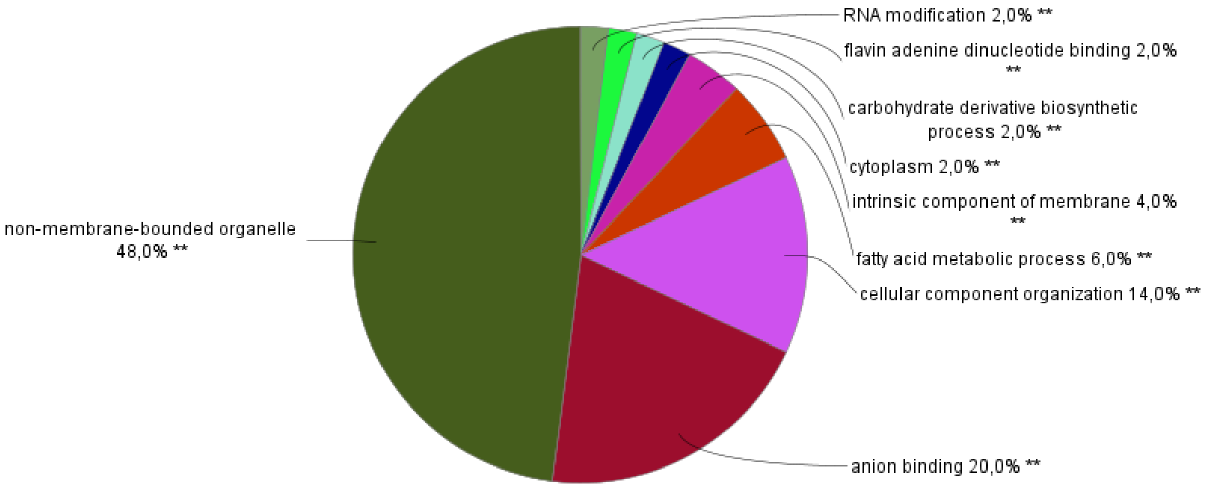

Figure S3. Distribution by function of proteins found in higher relative quantity in *L. paracasei* in presence of 30 µg/mL of punicalagin.

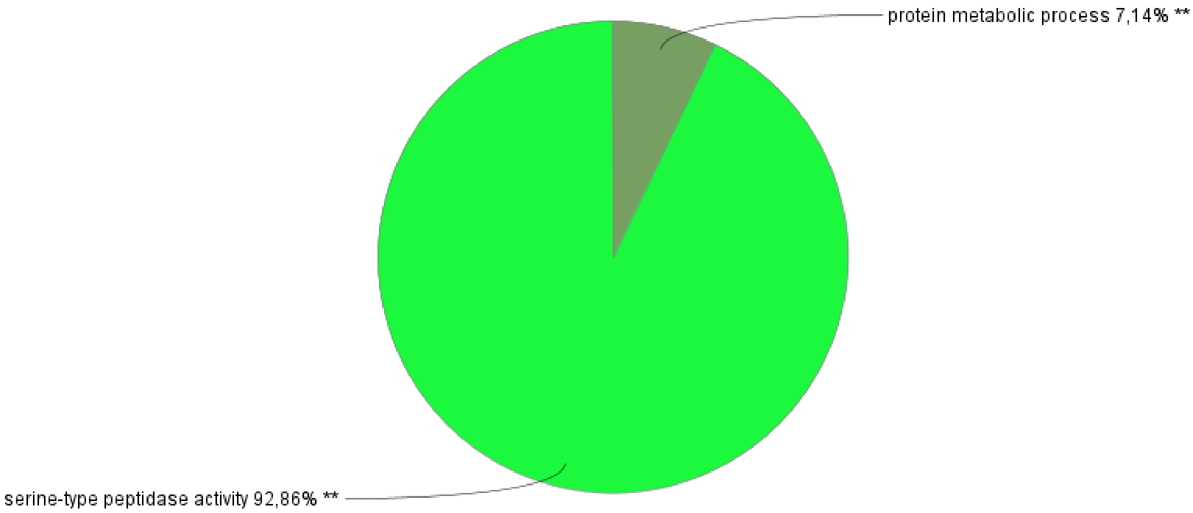

Figure S4. Distribution by function of proteins found in higher relative quantity in *E. faecium* in presence of 30 µg/mL of punicalagin.

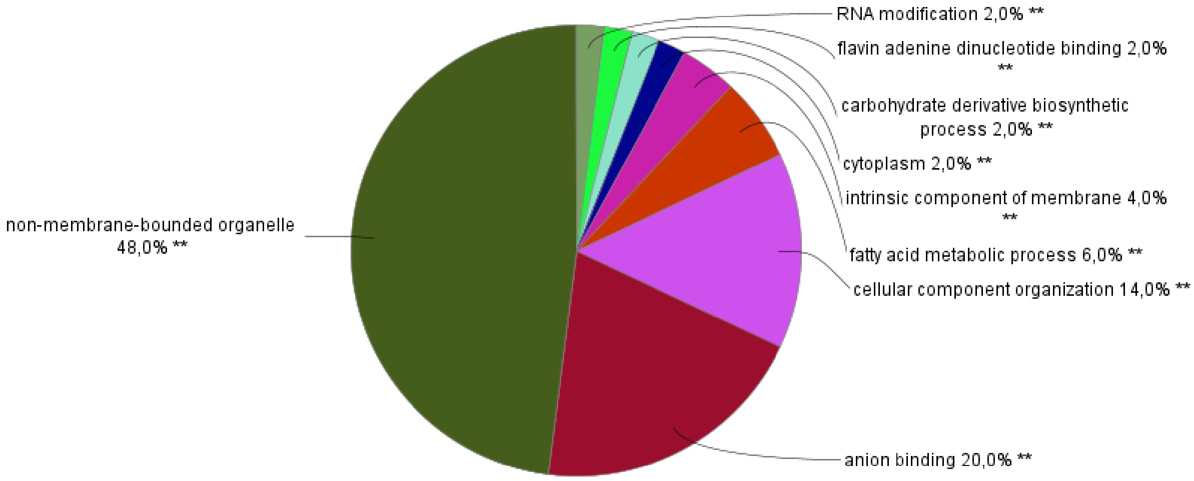

Table S1. Total data of proteins identified in this study for *Lactiplantibacillus plantarum* 89 along with Log2 fold change (in blue) and significance values (p, in red) of the 30 μg/mL punicalagin (P) treatment in comparison to the non-treated control (B). Control samples in yellow, samples treated with P in green

LFQ intensity 89C_1	LFQ intensity 89C_2	LFQ intensity 89C_3	LFQ intensity 89C_4	LFQ intensity 89C_5	LFQ intensity 89P_1	LFQ intensity 89P_2	LFQ intensity 89P_3	LFQ intensity 89P_4	LFQ intensity 89P_5	T-test Significant	Peptides	Razor + unique peptides	Unique peptides	Sequence coverage [%]	Unique + razor sequence coverage [%]	Unique sequence coverage [%]	Mol. weight [kDa]	Intensity	MS/MS count	Mean	Mean Control	Mean Punicalagin	Valid values	Valid values Control	Valid values Punicalagin	T-test p-value	T-test Difference	Protein IDs	Majority protein IDs	Gene name	Protein name
NaN	NaN	NaN	NaN	NaN	25,1186	25,167	25,175	25,1346	25,3364		3	3	3	13,1	13,1	13,1	29,639	3,18E+08	12	25,1863	NaN	25,1863	5	0	5	1	NaN	F9UQL0	F9UQL0	lp_2299	Metallophosphatase (MPP) superfamily protein
NaN	NaN	NaN	NaN	NaN	23,7233	23,891	23,8583	23,7564	23,9389		4	4	4	8,5	8,5	8,5	64,258	1,28E+08	10	23,8336	NaN	23,8336	5	0	5	1	NaN	F9URC8	F9URC8	pox3	Pyruvate oxidase
30,3854	NaN	NaN	NaN	NaN	26,4946	26,4156	26,1258	26,2152	26,3657		3	3	3	24,6	24,6	24,6	7,0011	2,35E+09	12	27,0004	30,3854	26,3234	6	1	5	1	-4,06198	Q88WK6	Q88WK6	rpmB	50S ribosomal protein L28
NaN	NaN	NaN	25,5173	NaN	25,7545	25,8072	25,5884	25,7801	25,6483		4	4	4	22,5	22,5	22,5	20,253	4,46E+08	19	25,6826	25,5173	25,7157	6	1	5	1	0,198445	F9UM30	F9UM30	lp_0811	DNA-directed DNA polymerase III, epsilon chain
NaN	NaN	NaN	23,7689	NaN	24,2069	24,2619	24,1607	23,9357	24,2517		4	4	4	16,3	16,3	16,3	41,304	1,70E+08	14	24,0976	23,7689	24,1634	6	1	5	1	0,394485	F9USZ2	F9USZ2	msmX	Maltodextrin ABC transporter, ATP-binding protein
NaN	25,4755	NaN	NaN	25,5356	25,8754	26,5411	26,4717	25,8782	26,39	+	2	2	2	45,8	45,8	45,8	6,6348	6,01E+08	18	26,0239	25,5056	26,2313	7	2	5	0,0318586	0,725691	Q88WS9	Q88WS9	rpmF	50S ribosomal protein L32
25,0218	NaN	24,6808	NaN	NaN	26,2192	26,2176	25,8597	25,958	26,0595	+	2	2	2	9,2	9,2	9,2	30,707	4,59E+08	18	25,7167	24,8513	26,0628	7	2	5	0,0004586	1,21146	F9URU8	F9URU8	lp_2845	Extracellular transglycosylase, with LysM peptidoglycan binding domain
NaN	24,1565	NaN	24,4654	24,3152	24,3142	24,7737	24,8257	24,9029	24,7087	+	2	2	2	2,8	2,8	2,8	83,876	2,36E+08	9	24,5578	24,3124	24,705	8	3	5	0,0413226	0,392621	F9UQ36	F9UQ36	recJ	Single-stranded-DNA-specific exonuclease RecJ
23,6319	23,9711	23,6528	NaN	NaN	24,3191	24,3814	24,3621	24,6252	24,5002	+	3	3	3	12,6	12,6	12,6	35,96	2,09E+08	16	24,1805	23,752	24,4376	8	3	5	0,00076107	0,685634	F9UUA8	F9UUA8	lp_3430	Peroxidase
23,7081	23,5342	NaN	NaN	24,1786	24,4899	24,7013	24,3292	24,7268	24,7312	+	2	2	2	26	26	26	16,608	2,09E+08	8	24,2999	23,807	24,5957	8	3	5	0,0042638	0,788728	F9UNH5	F9UNH5	lp_1427	Nucleoside 2-deoxyribosyltransferase
24,0487	NaN	NaN	24,1803	24,3189	24,2105	23,6794	24,2712	24,2049	24,2269		2	2	2	8,3	8,3	8,3	34,814	2,60E+08	10	24,1426	24,1826	24,1186	8	3	5	0,699126	-0,0640209	F9UM06	F9UM06	lp_0785	2-hydroxyacid dehydrogenase
24,6707	23,8852	NaN	NaN	23,9382	24,8403	24,297	24,7175	24,7174	24,442		3	3	3	10,6	10,6	10,6	21,879	2,75E+08	23	24,4385	24,1647	24,6028	8	3	5	0,103662	0,438132	Q88YP8	Q88YP8	recR	Recombination protein RecR
NaN	28,6948	28,6639	28,8871	28,8352	28,8183	29,0503	28,9363	29,1176	28,9783	+	3	3	3	15,5	15,5	15,5	31,659	3,84E+09	19	28,8869	28,7702	28,9802	9	4	5	0,0261454	0,209905	F9UR78	F9UR78	hisK	Histidinol-phosphatase
NaN	25,5976	25,5578	25,55	25,4214	25,7857	25,9018	25,7064	25,8259	25,6599	+	2	2	2	10,4	10,4	10,4	22,899	5,75E+08	11	25,6674	25,5317	25,7759	9	4	5	0,00437322	0,244244	F9URW9	F9URW9	pgm8	Phosphoglycerate mutase family protein
26,1576	NaN	26,2691	26,3198	26,3221	26,4215	26,5345	26,7789	26,5074	26,4183	+	3	3	3	5,3	5,3	5,3	83,631	7,90E+08	8	26,4144	26,2671	26,5321	9	4	5	0,0143827	0,265034	F9UUF1	F9UUF1	melA	Alpha-galactosidase
24,0132	24,0592	23,8617	NaN	23,9269	24,0955	24,3756	24,3836	24,2044	24,1464	+	3	2	2	6,3	4,6	4,6	48,989	2,32E+08	15	24,1185	23,9652	24,2411	9	4	5	0,00918061	0,275853	Q88Y40	Q88Y40	asnS1	Asparagine--tRNA ligase 1
NaN	24,6726	24,5711	24,6553	24,9829	24,9491	25,2426	24,7784	25,0842	25,0266	+	2	2	2	3,4	3,4	3,4	50,156	4,77E+08	18	24,8848	24,7205	25,0162	9	4	5	0,0399938	0,295722	F9UM72	F9UM72	bcaP	Branched-chain amino acid permease
23,6866	NaN	23,9278	23,7743	23,6739	24,0482	24,2315	24,1759	24,1675	24,3637	+	6	6	6	5,7	5,7	5,7	136,24	2,71E+08	30	24,0055	23,7656	24,1974	9	4	5	0,00084229	0,431721	Q88U40	Q88U40	rexB	ATP-dependent helicase/deoxyribonuclease subunit B
24,0163	24,1219	24,3138	24,1789	NaN	24,3504	24,5747	24,5246	24,8133	24,824	+	4	4	4	20,9	20,9	20,9	33,847	4,29E+08	25	24,4131	24,1577	24,6174	9	4	5	0,00541261	0,459678	F9USA6	F9USA6	lp_2974	ABC transporter, substrate binding protein
24,5806	NaN	23,4453	23,2849	24,1345	24,9121	25,0673	24,9448	24,6285	24,495	+	3	3	3	11,5	11,5	11,5	27,077	3,33E+08	14	24,3881	23,8613	24,8096	9	4	5	0,0140613	0,948214	F9UN35	F9UN35	lp_1242	Extracellular protein, NlpC/P60 family,gamma-D-glutamate-meso-diaminopimelate muropeptidase
25,1755	23,7171	24,2145	NaN	23,937	23,71	23,5873	23,7222	23,7399	24,4425		3	3	3	15,4	15,4	15,4	25,691	1,84E+08	12	24,0273	24,261	23,8404	9	4	5	0,244738	-0,420647	F9USS8	F9USS8	larR	Lactate racemization regulatory protein
24,825	24,8534	NaN	25,1166	25,2078	24,7156	24,5392	24,4361	24,9949	24,8468		3	3	3	5,5	5,5	5,5	83,29	3,67E+08	13	24,8373	25,0007	24,7065	9	4	5	0,0767625	-0,294171	F9UMC2	F9UMC2	lp_0923	Cell surface protein, PXTG-motif cell wall anchor
24,3333	24,396	24,5097	24,2307	NaN	24,1871	24,3273	24,3994	24,5297	24,4467		6	6	6	21,7	21,7	21,7	42,438	4,42E+08	12	24,3733	24,3674	24,378	9	4	5	0,902356	0,0106064	Q890K5	Q890K5	recF	DNA replication and repair protein RecF
24,5206	25,0171	NaN	24,6594	24,5284	24,3333	24,7109	24,8617	24,7646	24,947		3	3	3	13,8	13,8	13,8	22,131	3,13E+08	19	24,7048	24,6814	24,7235	9	4	5	0,796772	0,0421214	F9UUC8	F9UUC8	lp_3458	Cell surface protein, CscD family, PXTG-motif cell wall anchor
27,405	27,1648	27,193	NaN	27,2887	27,701	27,1915	27,2185	27,1126	27,9906		3	3	3	7,2	7,2	7,2	74,258	1,46E+09	14	27,3628	27,2629	27,4428	9	4	5	0,399035	0,179944	F9UP00	F9UP00	recG	ATP-dependent DNA helicase RecG
23,9524	23,9348	NaN	23,9944	24,2196	24,1308	24,2247	24,7844	24,0955	24,0474		3	3	3	9,6	9,6	9,6	28,953	2,97E+08	12	24,1538	24,0253	24,2566	9	4	5	0,200811	0,231283	F9UT96	F9UT96	lp_3185	Branched-chain amino acid transport protein
25,6938	25,5501	NaN	25,8012	25,4736	25,7398	25,9951	25,6803	26,2348	25,8268		3	3	3	27	27	27	10,14	6,34E+08	12	25,7773	25,6297	25,8954	9	4	5	0,0810362	0,265673	Q88V67	Q88V67	rpsN	30S ribosomal protein S14
25,9615	25,5272	NaN	25,3851	25,6422	25,8804	26,0776	25,9699	25,9762	25,615		7	7	7	34,9	34,9	34,9	23,933	7,55E+08	34	25,7817	25,629	25,9038	9	4	5	0,0901672	0,274837	F9UL09	F9UL09	lp_0535	Uncharacterized protein
25,9719	NaN	25,8067	25,8781	25,6657	25,8176	26,1056	25,9792	26,4608	26,4303		3	3	3	12,1	12,1	12,1	34,377	7,23E+08	16	26,0129	25,8306	26,1587	9	4	5	0,0698696	0,328087	F9UQT4	F9UQT4	lp_2393	Lipoprotein
28,7971	26,2935	NaN	26,7295	25,9815	26,4045	27,2537	27,7769	27,7389	27,631		2	2	2	22,9	22,9	22,9	7,1677	1,68E+09	40	27,1785	26,9504	27,361	9	4	5	0,534437	0,410586	Q88UT8	Q88UT8	atpE	ATP synthase subunit c
25,5644	25,6116	NaN	25,3509	24,5476	25,0548	25,7781	26,4218	26,5058	26,2426		2	2	2	27,9	27,9	27,9	7,5118	8,08E+08	25	25,6753	25,2686	26,0006	9	4	5	0,0906602	0,731987	F9UL81	F9UL81	secE	Protein translocase subunit SecE
28,2822	28,5641	28,6917	28,7224	28,5867	27,174	27,3076	27,3615	27,1405	27,4586	+	4	4	4	22,2	22,2	22,2	22,705	7,78E+09	108	27,929	28,5694	27,2885	10	5	5	1,09E-06	-1,281	P77889	P77889	pyrE	Orotate phosphoribosyltransferase
28,2541	28,5335	28,6131	28,5825	28,2895	27,2235	27,2212	27,3575	27,1819	27,4463	+	12	12	12	50,7	50,7	50,7	45,438	8,59E+09	140	27,8703	28,4545	27,2861	10	5	5	1,26E-06	-1,16845	P77884	P77884	pyrC	Dihydroorotase
27,7106	28,0243	28,0613	28,1072	27,8595	26,8176	26,8738	26,8834	26,7709	26,871	+	12	12	12	45	45	45	34,711	1,10E+10	176	27,398	27,9526	26,8434	10	5	5	5,05E-07	-1,10923	P77883	P77883	pyrB	Aspartate carbamoyltransferase
28,2997	28,6293	28,73	28,5795	27,6937	27,3454	27,2218	27,5437	27,312	27,5583	+	50	50	50	56,8	56,8	56,8	115,76	3,88E+10	549	27,8913	28,3864	27,3962	10	5	5	0,00107607	-0,99018	P77886;Q9RL	P77886	pyrAB	Carbamoyl-phosphate synthase pyrimidine-specific large chain
29,2519	29,7349	29,7493	29,7562	29,4024	28,4503	28,5925	28,6328	28,6478	28,6947	+	10	10	10	57	57	57	31,224	1,57E+10	112	29,0913	29,5789	28,6036	10	5	5	2,60E-05	-0,975317	P77887	P77887	pyrD	Dihydroorotate dehydrogenase A (fumarate)
27,24	27,3874	27,6281	27,5296	27,4691	26,4598	26,7385	26,6705	26,6615	26,6181	+	12	12	12	42,6	42,6	42,6	39,983	5,82E+09	111	27,0403	27,4508	26,6297	10	5	5	7,39E-06	-0,821167	P77885	P77885	pyrAA	Carbamoyl-phosphate synthase pyrimidine-specific small chain
27,5966	27,8656	27,7664	27,9733	27,8323	27,9064	27,2723	26,4368	26,6381	26,6976	+	13	13	13	52,8	52,8	52,8	21,443	7,43E+09	123	27,3985	27,8068	26,9903	10	5	5	0,0178892	-0,816579	Q88VL9	Q88VL9	grpE	Protein GrpE
27,0036	27,0807	27,2543	27,1631	27,1638	26,3232	26,5115	26,5578	26,4932	26,5712	+	12	12	12	22,5	22,5	22,5	85,674	3,03E+09	69	26,8122	27,1331	26,4914	10	5	5	6,14E-06	-0,641731	F9UP85	F9UP85	mapA	Maltose phosphorylase
27,9022	28,2068	28,1402	28,1401	28,1054	27,8491	27,7507	27,1405	27,394	27,3081	+	7	7	7	33,6	33,6	33,6	23,763	9,08E+09	128	27,7937	28,099	27,4885	10	5	5	0,00284768	-0,610471	F9US84	F9US84	pgmB2	Beta-phosphoglucomutase
30,3134	30,4523	30,4898	30,3974	30,2137	29,7163	29,7818	29,733	29,7862	29,8264	+	17	17	17	79,4	79,4	79,4	19,824	5,44E+10	380	30,071	30,3733	29,7687	10	5	5	3,39E-06	-0,604596	P71479	P71479	pyrR1	Bifunctional protein PyrR 1
29,7257	29,7982	29,9771	29,9146	29,774	29,265	29,0425	29,3057	29,2631	29,5092	+	37	37	37	41,5	41,5	41,5	93,661	4,78E+10	592	29,5575	29,8379	29,2771	10	5	5	0,00020924	-0,560794	Q88VK7	Q88VK7	infB	Translation initiation factor IF-2
29,2498	29,3584	29,4465	29,4198	29,404	29,4105	29,2263	28,4893	28,5452	28,5129	+	19	19	19	62,9	62,9	62,9	26,173	2,54E+10	255	29,1063	29,3757	28,8368	10	5	5	0,0284274	-0,538828	F9UQB9	F9UQB9	divIVA	Cell division initiation protein DivIVA
29,6648	29,2066	29,2938	29,6679	29,8005	29,2579	29,102	28,6346	28,6878	29,2727	+	10	10	10	59,3	59,3	59,3	13,041	1,66E+10	111	29,2588	29,5267	28,991	10	5	5	0,0179841	-0,535723	Q88XX0	Q88XX0	rplR	50S ribosomal protein L18
30,0003	30,1216	29,9532	29,9321	30,0495	29,8412	29,4709	29,3335	29,3612	29,4939	+	7	7	7	52,7	52,7	52,7	16,066	2,43E+10	278	29,7558	30,0114	29,5002	10	5	5	0,00074888	-0,511206	F9UMC9	F9UMC9	asp2	Alkaline shock protein
27,5338	27,917	28,0009	27,909	27,7411	27,5773	27,4463	27,2452	27,2764	27,1348	+	18	18	18	54,3	54,3	54,3	43,199	1,22E+10	203	27,5782	27,8204	27,336	10	5	5	0,00283399	-0,48438	Q88ZS1	Q88ZS1	mtlD	Mannitol-1-phosphate 5-dehydrogenase
26,1991	26,4906	26,5617	26,6334	26,449	26,1058	26,0516	25,7436	25,9814	26,0676	+	6	6	6	19,7	19,7	19,7	45,971	2,23E+09	46	26,2284	26,4668	25,99	10	5	5	0,00127242	-0,476767	F9UQ91	F9UQ91	pdhC	Dihydrolipoamide acetyltransferase component of pyruvate dehydrogenase complex
28,7278	28,5948	28,4825	28,7139	28,7822	28,5887	28,267	27,9967	28,1269	27,9992	+	8	8	8	61,2	61,2	61,2	17,914	6,03E+09	77	28,428	28,6603	28,1957	10	5	5	0,00532153	-0,464534	Q88WQ9	Q88WQ9	greA2	Transcription elongation factor GreA 2
26,9791	26,8253	26,8335	26,7861	26,6542	26,8915	26,2832	25,9919	26,2317	26,3796	+	8	8	8	8,1	8,1	8,1	76,252	2,71E+09	75	26,5856	26,8156	26,3556	10	5	5	0,0191353	-0,460061	F9URQ9	F9URQ9	lp_2793	Uncharacterized protein
27,8084	28,2645	28,0539	28,2108	27,9586	27,615	27,3839	27,7247	27,7068	27,6094	+	4	4	4	42,9	42,9	42,9	11,341	5,17E+09	115	27,8336	28,0592	27,608	10	5	5	0,00233167	-0,451279	F9URP9	F9URP9	pts20B	PTS system, cellobiose-specific EIIB component
28,2893	28,3964	28,3969	28,2958	28,222	27,6883	27,6175	27,9779	27,9901	28,0769	+	33	33	33	45,7	45,7	45,7	92,36	1,32E+10	323	28,0951	28,3201	27,8701	10	5	5	0,00167836	-0,449928	F9UM19	F9UM19	rnr	Ribonuclease R
26,4493	26,77	26,7353	26,9971	26,8795	26,3297	26,2306	26,3102	26,428	26,4441	+	4	4	4	40	40	40	12,474	1,54E+09	38	26,5574	26,7662	26,3485	10	5	5	0,00303626	-0,417726	F9URP8	F9URP8	pts20A	PTS system, cellobiose-specific EIIA component
29,7082	30,0999	30,0174	30,014	29,7872	29,5263	29,2183	29,5744	29,6641	29,7152	+	2	2	2	51,9	51,9	51,9	11,701	9,20E+09	39	29,7325	29,9253	29,5397	10	5	5	0,00998067	-0,385659	F9USD6	F9USD6	pts23B	PTS system, cellobiose-specific EIIB component
25,6618	26,0393	26,0451	26,1585	25,8703	25,6061	25,6979	25,5475	25,6539	25,3538	+	8	8	8	37,7	37,7	37,7	36,401	1,83E+09	52	25,7634	25,955	25,5718	10	5	5	0,0065897	-0,383211	F9UUE7	F9UUE7	galE2	UDP-glucose 4-epimerase
27,2613	27,3988	27,4246	27,3906	27,3657	26,9873	27,0109	26,973	26,9971	27,0295	+	16	16	16	60,3	60,3	60,3	35,439	8,95E+09	157	27,1839	27,3682	26,9996	10	5	5	1,77E-06	-0,368628	F9UQ92	F9UQ92	pdhB	Pyruvate dehydrogenase complex, E1 component,beta subunit
23,8535	24,1283	24,513	23,9815	24,1646	23,4536	23,6598	23,8816	24,0204	23,7961	+	3	3	3	8,8	8,8	8,8	63,497	2,90E+08	18	23,9453	24,1282	23,7623	10	5	5	0,0379079	-0,365903	F9UM63	F9UM63	pox1	Pyruvate oxidase
32,0849	32,1864	32,2788	32,3179	32,151	32,3379	32,0044	31,4698	31,6752	31,7044	+	59	59	59	72	72	72	66,729	4,89E+11	2199	32,0211	32,2038	31,8383	10	5	5	0,048386	-0,365478	Q88VM0	Q88VM0	dnaK	Chaperone protein DnaK
26,9149	27,1155	27,0922	27,0221	27,1122	26,8011	26,7889	26,6611	26,6814	26,558	+	7	7	7	21,7	21,7	21,7	36,104	3,44E+09	69	26,8747	27,0514	26,6981	10	5	5	0,00031955	-0,353285	F9USY0	F9USY0	dak1B	Dihydroxyacetone phosphotransferase,dihydroxyacetone binding subunit
28,0625	28,0509	28,0287	28,0234	27,9941	27,5269	27,631	27,686	27,8975	27,6943	+	9	9	9	66,4	66,4	66,4	13,045	5,41E+09	132	27,8595	28,0319	27,6871	10	5	5	0,00051298	-0,3448	F9URF9	F9URF9	lp_2669	Uncharacterized protein
27,0808	27,4241	27,3843	27,3117	27,3799	26,9615	26,9568	27,1666	26,9237	26,8754	+	12	11	11	25,7	24,2	24,2	54,895	5,46E+09	148	27,1465	27,3162	26,9768	10	5	5	0,00267509	-0,339395	F9URP7;F9UU	F9URP7	pbg5	6-phospho-beta-glucosidase
25,275	25,7892	25,3014	25,4766	25,3649	25,1839	25,1266	25,02	25,1281	25,0664	+	2	2	2	22,9	22,9	22,9	14,637	5,41E+08	19	25,2732	25,4414	25,105	10	5	5	0,00881985	-0,336424	Q88RZ2	Q88RZ2	rbsD	D-ribose pyranase
25,5918	25,6175	25,4001	25,7314	25,8141	25,0153	25,1511	25,4091	25,6891	25,2162	+	2	2	2	5,8	5,8	5,8	53,492	7,62E+08	28	25,4636	25,631	25,2962	10	5	5	0,0396399	-0,334824	F9UP72	F9UP72	pepD4	Dipeptidase
30,9885	31,0487	30,8908	30,9556	31,0291	30,9052	30,9376	30,3457	30,5521	30,5419	+	11	11	11	75	75	75	15,786	4,82E+10	268	30,8195	30,9825	30,6565	10	5	5	0,0243186	-0,326048	F9UMC8	F9UMC8	asp1	Alkaline shock protein
25,3329	25,538	25,6668	25,4251	25,441	25,2911	25,2617	24,9823	25,0948	25,1632	+	5	5	5	23,6	23,6	23,6	39,226	7,27E+08	28	25,3197	25,4808	25,1586	10	5	5	0,00378789	-0,322136	Q88W73	Q88W73	pyrAA2	Carbamoyl-phosphate synthase small chain
32,4323	32,4577	32,6282	32,6383	32,4681	32,6535	32,2733	31,9781	32,0509	32,1222	+	66	66	66	88,7	88,7	88,7	57,436	8,54E+11	4407	32,3703	32,5249	32,2156	10	5	5	0,0419471	-0,309323	Q88YM5	Q88YM5	groL	60 kDa chaperonin
26,7725	26,8389	26,8855	26,7915	26,7596	26,6263	26,4887	26,4707	26,4654	26,48	+	14	14	14	46,5	46,5	46,5	41,468	4,96E+09	143	26,6579	26,8096	26,5062	10	5	5	4,59E-05	-0,303394	F9UQ93	F9UQ93	pdhA	Pyruvate dehydrogenase E1 component subunit alpha
26,46	26,6411	26,4219	26,4449	26,5975	26,1835	26,0916	26,2309	26,3188	26,2857	+	11	11	11	27,1	27,1	27,1	58,095	3,18E+09	82	26,3676	26,5131	26,2221	10	5	5	0,0012338	-0,290995	F9UM58	F9UM58	ppx3	Exopolyphosphatase
28,936	29,0325	29,1897	29,1643	29,0452	29,1284	28,8417	28,5803	28,6765	28,7008	+	18	18	18	56,4	56,4	56,4	45,013	3,06E+10	309	28,9295	29,0735	28,7856	10	5	5	0,026464	-0,287969	F9UQC3	F9UQC3	ftsZ	Cell division protein FtsZ
29,9781	30,1627	30,2331	30,1822	30,226	29,894	29,8944	29,9371	29,7684	29,8927	+	21	21	21	56,9	56,9	56,9	50,247	3,31E+10	312	30,0169	30,1564	29,8773	10	5	5	0,00090721	-0,279089	F9UL68	F9UL68	pepC1	Aminopeptidase
27,4186	27,7954	27,8157	27,7555	27,6935	27,2609	27,3676	27,5082	27,4936	27,4783	+	18	18	16	48,5	48,5	45,4	54,711	1,56E+10	247	27,5587	27,6957	27,4217	10	5	5	0,0131788	-0,274026	F9URP6	F9URP6	pbg4	6-phospho-beta-glucosidase
28,5666	28,5687	28,3541	28,5309	28,5312	28,3524	28,2173	28,1915	28,1795	28,2465	+	8	8	8	44,5	44,5	44,5	18,19	8,19E+09	132	28,3739	28,5103	28,2374	10	5	5	0,00064291	-0,272867	F9UQN0	F9UQN0	tpx	Thiol peroxidase
28,2179	28,305	28,3417	28,3561	28,2078	27,9294	28,0636	28,0415	28,1547	27,9648	+	7	7	7	8,8	8,8	8,8	52,204	8,79E+09	97	28,1583	28,2857	28,0308	10	5	5	0,00094942	-0,254926	F9USD7	F9USD7	pts23C	Permease IIC component
30,9191	30,9918	30,9542	30,9489	31,0531	30,9212	30,8107	30,6121	30,7424	30,5082	+	13	13	13	53,7	53,7	53,7	21,725	8,86E+10	687	30,8462	30,9734	30,7189	10	5	5	0,0102666	-0,254499	F9ULW6	F9ULW6	hpf	Ribosome hibernation promoting factor
27,4217	27,4605	27,3856	27,4151	27,4613	27,3777	27,3763	26,9933	27,0999	27,0272	+	10	10	10	58,1	58,1	58,1	29,266	6,95E+09	110	27,3019	27,4288	27,1749	10	5	5	0,0178801	-0,253963	F9UNK8	F9UNK8	sufC	ABC transporter, ATP-binding protein,iron-sulfur cluster assembly ATPase protein SufC
26,7027	26,616	26,6241	26,741	26,8904	26,6471	26,2282	26,4883	26,3402	26,6158	+	3	3	3	24,1	24,1	24,1	19,303	1,38E+09	37	26,5894	26,7148	26,4639	10	5	5	0,0287417	-0,25089	P59389	P59389	pyrR2	Bifunctional protein PyrR 2
27,753	27,6644	27,8063	28,1311	27,8763	27,6625	27,6071	27,4959	27,5388	27,6794	+	7	7	7	78,1	78,1	78,1	15,791	5,31E+09	57	27,7215	27,8462	27,5967	10	5	5	0,020556	-0,249482	F9UMW3	F9UMW3	lp_1163	Universal stress protein
28,5341	28,5431	28,4353	28,4438	28,6338	28,3058	28,292	28,1185	28,3076	28,3281	+	8	8	8	31,8	31,8	31,8	19,371	1,09E+10	115	28,3942	28,518	28,2704	10	5	5	0,00159647	-0,247642	F9UL17	F9UL17	lp_0543	RNA binding protein, contains ribosomal protein S1 domain
24,7163	24,7627	24,801	24,5154	24,9577	24,5066	24,6095	24,3817	24,3331	24,7043	+	4	4	4	8,9	8,9	8,9	44,713	4,93E+08	25	24,6288	24,7506	24,507	10	5	5	0,039838	-0,243572	F9URN8	F9URN8	lp_2767	Acetyltransferase, GNAT family
28,9039	29,0625	29,0624	29,0717	29,0634	28,8144	28,8441	28,8527	28,5442	28,9078	+	11	11	11	60,9	60,9	60,9	28,554	1,48E+10	188	28,9127	29,0328	28,7926	10	5	5	0,0100189	-0,240142	Q88W01	Q88W01	dapB	4-hydroxy-tetrahydrodipicolinate reductase
26,5015	26,6022	26,7098	26,5839	26,4516	26,2088	26,3203	26,4261	26,3189	26,4675	+	19	19	19	27,3	27,3	27,3	94,308	4,19E+09	159	26,4591	26,5698	26,3483	10	5	5	0,00828331	-0,221455	F9UPD2	F9UPD2	pyrAB2	Carbamoyl-phosphate synthase (glutamine-hydrolyzing)
29,2441	29,3358	29,4271	29,399	29,3353	29,1362	29,1271	29,1311	29,0574	29,2256	+	24	24	24	53,1	53,1	53,1	59,735	4,14E+10	350	29,2419	29,3482	29,1355	10	5	5	0,00088453	-0,212743	Q88Z76	Q88Z76	pyrG	CTP synthase
27,3891	27,5154	27,6391	27,5654	27,6542	27,1966	27,222	27,459	27,388	27,474	+	33	33	33	50,9	50,9	50,9	77,472	1,82E+10	361	27,4503	27,5527	27,3479	10	5	5	0,0269146	-0,204729	Q6LWE5	Q6LWE5	traE	Conjugation protein
27,9746	27,8815	27,7211	27,8796	27,7917	27,463	27,5967	27,665	27,7938	27,7262	+	8	8	8	18	18	18	49,85	4,42E+09	63	27,7493	27,8497	27,6489	10	5	5	0,0227484	-0,20076	F9UQL8	F9UQL8	thrA2	Aspartokinase
31,2681	31,4767	31,3855	31,4561	31,4554	31,4098	31,2645	31,086	31,1391	31,1496	+	16	16	16	56,6	56,6	56,6	33,62	7,73E+10	334	31,3091	31,4084	31,2098	10	5	5	0,0210933	-0,198569	Q88W32	Q88W32	ppaC	Probable manganese-dependent inorganic pyrophosphatase
28,9592	29,2521	29,2421	29,2738	29,1512	29,061	28,9438	28,8407	29,023	29,0299	+	14	14	14	68,4	68,4	68,4	25,881	3,18E+10	304	29,0777	29,1757	28,9797	10	5	5	0,0236967	-0,196015	Q88ZS6	Q88ZS6	nagB	Glucosamine-6-phosphate deaminase
30,844	30,8999	31,076	31,114	31,1276	30,9424	30,8047	30,7026	30,7252	30,9304	+	9	9	9	68,7	68,7	68,7	18,115	6,38E+10	343	30,9167	31,0123	30,8211	10	5	5	0,0380344	-0,191251	F9UPA1	F9UPA1	lp_1747	Nucleotide-binding protein, universal stress protein UspA family
28,0453	28,0295	27,9784	27,9675	27,9283	27,9077	27,8428	27,6192	27,9276	27,7288	+	9	9	9	58,3	58,3	58,3	23,461	7,43E+09	125	27,8975	27,9898	27,8052	10	5	5	0,0174319	-0,184583	Q88WL7	Q88WL7	gmk	Guanylate kinase
28,1653	28,2325	28,1461	28,1155	28,2278	28,1705	28,0945	27,9129	27,9129	27,8921	+	16	16	16	83,8	83,8	83,8	27,061	1,00E+10	153	28,087	28,1774	27,9966	10	5	5	0,0184791	-0,180872	F9US61	F9US61	rrp1	Two-component system response regulator
29,7896	29,8846	29,8407	29,7302	29,877	29,7989	29,7357	29,589	29,5273	29,5964	+	20	20	20	51,5	51,5	51,5	45,161	3,21E+10	457	29,7369	29,8244	29,6495	10	5	5	0,0169989	-0,174973	F9UPM8	F9UPM8	pepT	Peptidase T
28,7068	28,7385	28,809	28,8555	28,8534	28,6373	28,5671	28,6271	28,5535	28,7503	+	11	11	11	19,3	19,3	19,3	104,97	4,95E+09	57	28,7099	28,7927	28,6271	10	5	5	0,00707795	-0,165548	F9UP92	F9UP92	dinG	3-5 exonuclease DinG
29,4827	29,6378	29,7465	29,6866	29,5445	29,3571	29,3912	29,5831	29,4832	29,476	+	8	8	8	47,3	47,3	47,3	17,103	1,69E+10	163	29,5389	29,6196	29,4581	10	5	5	0,0310939	-0,161522	F9UPK2	F9UPK2	lp_1872	Uncharacterized protein
27,3083	27,2604	27,3157	27,3907	27,5699	27,1992	27,309	27,2249	27,1675	27,1687	+	16	16	16	55,6	55,6	55,6	34,5	7,19E+09	174	27,2914	27,369	27,2139	10	5	5	0,032992	-0,155145	F9UNW0	F9UNW0	lp_1583	Uncharacterized protein
30,0266	30,1762	30,1405	30,1567	30,2118	29,9717	29,9669	30,018	30,0346	29,96	+	12	12	12	61,2	61,2	61,2	22,972	6,38E+10	582	30,0663	30,1423	29,9902	10	5	5	0,0023474	-0,152092	Q9RE01	Q9RE01	upp	Uracil phosphoribosyltransferase
29,2011	29,4353	29,4051	29,4213	29,418	29,1821	29,1566	29,2138	29,3348	29,2347	+	12	12	12	52,7	52,7	52,7	26,898	1,49E+10	145	29,3003	29,3762	29,2244	10	5	5	0,0222054	-0,151752	F9UT59	F9UT59	treR	Trehalose operon transcriptional repressor,GntR family
28,2905	28,2918	28,2743	28,329	28,2914	28,2362	28,1718	28,0215	28,1188	28,2142	+	3	3	3	22,4	22,4	22,4	16,437	4,19E+09	56	28,2239	28,2954	28,1525	10	5	5	0,00674793	-0,142881	F9UNX2	F9UNX2	lp_1597	General stress protein, Gls24 family
28,2882	28,5675	28,5111	28,4509	28,3955	28,3293	28,3375	28,2583	28,3284	28,2454	+	11	11	11	52	52	52	24,642	1,14E+10	137	28,3712	28,4426	28,2998	10	5	5	0,0252853	-0,142868	Q88YY5	Q88YY5	rpiA	Ribose-5-phosphate isomerase A
23,7779	23,7064	23,8411	23,8699	23,6856	23,6368	23,712	23,6974	23,5034	23,6176	+	3	3	3	6,4	6,4	6,4	70,473	2,09E+08	17	23,7048	23,7762	23,6334	10	5	5	0,0246961	-0,142722	F9UPS8	F9UPS8	dnaG	DNA primase
28,8693	29,0446	29,1478	29,1097	29,1408	28,9276	28,8826	28,929	28,9477	28,9381	+	25	25	25	63,6	63,6	63,6	44,475	2,74E+10	376	28,9937	29,0624	28,925	10	5	5	0,0314725	-0,13746	F9UNY1	F9UNY1	opuA	Glycine betaine/carnitine/choline ABC transporter, ATP-binding protein
29,8427	29,8865	30,0305	29,9489	29,9753	29,8257	29,7538	29,8199	29,778	29,8263	+	47	47	47	71,4	71,4	71,4	73,818	9,05E+10	756	29,8688	29,9368	29,8007	10	5	5	0,00554085	-0,136034	Q88WU9	Q88WU9	thrS	Threonine--tRNA ligase
29,1737	29,2912	29,2463	29,2199	29,331	29,2553	29,1709	29,0797	29,0415	29,0362	+	23	23	23	68,3	68,3	68,3	41,402	3,88E+10	412	29,1846	29,2524	29,1167	10	5	5	0,0271909	-0,135716	F9UL33	F9UL33	nagA	N-acetylglucosamine-6-phosphate deacetylase
28,3957	28,4409	28,5673	28,608	28,5498	28,4345	28,3735	28,4172	28,2937	28,3823	+	17	17	17	52,9	52,9	52,9	56,845	1,49E+10	226	28,4463	28,5123	28,3802	10	5	5	0,0227709	-0,132088	F9UTV3	F9UTV3	lp_0355	Multicopper oxidase
26,965	26,9672	27,0674	26,9109	26,9784	26,9534	26,9274	26,7595	26,8658	26,7388	+	6	6	6	52,5	52,5	52,5	15,646	3,21E+09	71	26,9134	26,9778	26,849	10	5	5	0,0331747	-0,128792	F9UM75	F9UM75	lp_0864	Bis(5-nucleosyl)-tetraphosphatase [asymmetrical]
31,1416	31,2039	31,229	31,2062	31,2932	31,1996	31,1142	31,0429	31,0293	31,0441	+	36	36	36	73,3	73,3	73,3	63,11	1,43E+11	1258	31,1504	31,2148	31,086	10	5	5	0,0126467	-0,128746	F9UN60	F9UN60	ptsI	Phosphoenolpyruvate-protein phosphotransferase
29,3717	29,4872	29,438	29,4031	29,5164	29,4162	29,4295	29,2381	29,3117	29,1784	+	28	28	28	72,1	72,1	72,1	39,893	4,08E+10	413	29,379	29,4433	29,3148	10	5	5	0,0497557	-0,128527	F9UTA5	F9UTA5	ychF	Ribosome-binding ATPase YchF
26,9611	26,9493	27,0032	27,025	27,0583	26,8146	26,9311	26,881	26,9239	26,8261	+	23	23	23	41,2	41,2	41,2	64,496	9,35E+09	230	26,9374	26,9994	26,8753	10	5	5	0,00423789	-0,124034	Q88UX2	Q88UX2	ezrA	Septation ring formation regulator EzrA
31,1539	31,3409	31,2545	31,3313	31,3454	31,2063	31,1607	31,1303	31,1596	31,2602	+	8	8	8	38,7	38,7	38,7	21,63	5,23E+10	311	31,2343	31,2852	31,1834	10	5	5	0,0463474	-0,101767	F9UMV3	F9UMV3	lp_1153	Transcription regulator, TetR family
28,3878	28,5462	28,4117	28,4641	28,4801	28,3721	28,4085	28,3038	28,385	28,352	+	15	15	15	46,6	46,6	46,6	33,321	1,23E+10	220	28,4111	28,458	28,3643	10	5	5	0,0215008	-0,0936951	Q88YI4	Q88YI4	lp_0779	Nucleotide-binding protein lp_0779
27,9833	28,0334	27,982	28,0079	27,9933	27,9997	27,9554	27,8469	27,9066	27,8648	+	4	4	4	21,7	21,7	21,7	19,403	5,44E+09	42	27,9573	28	27,9147	10	5	5	0,0213525	-0,0853172	Q88W25	Q88W25	hslV	ATP-dependent protease subunit HslV
30,2369	30,2663	30,2472	30,2054	30,2627	30,2491	30,2853	30,3301	30,3011	30,2863	+	38	38	38	58,9	58,9	58,9	63,516	5,08E+10	659	30,267	30,2437	30,2904	10	5	5	0,0258076	0,0466591	Q88VK1	Q88VK1	proS	Proline--tRNA ligase
31,4301	31,4991	31,438	31,481	31,4971	31,5074	31,5518	31,5042	31,5444	31,5908	+	20	20	20	86,1	86,1	86,1	20,238	1,74E+11	1159	31,5044	31,4691	31,5397	10	5	5	0,0115138	0,0706581	Q88XX4	Q88XX4	rplE	50S ribosomal protein L5
30,1169	30,2412	30,2305	30,2608	30,2349	30,278	30,2909	30,2305	30,322	30,3232	+	19	19	19	59,7	59,7	59,7	34,265	5,98E+10	754	30,2529	30,2169	30,2889	10	5	5	0,0467654	0,0720497	Q88VY1	Q88VY1	pfkA	ATP-dependent 6-phosphofructokinase
28,9952	29,0128	28,9887	28,9782	29,0077	29,0226	29,117	29,0793	29,165	29,0166	+	24	24	24	47,7	47,7	47,7	52,614	3,04E+10	298	29,0383	28,9965	29,0801	10	5	5	0,0200842	0,0835987	Q88W26	Q88W26	hslU	ATP-dependent protease ATPase subunit HslU
31,3758	31,4057	31,2904	31,3785	31,4615	31,469	31,5054	31,4439	31,513	31,4026	+	20	20	20	90,5	90,5	90,5	16,169	1,51E+11	645	31,4246	31,3824	31,4668	10	5	5	0,0395001	0,0843872	Q88XU8	Q88XU8	rplM	50S ribosomal protein L13
26,8824	26,9647	26,9431	26,9355	26,9562	26,9685	26,9642	27,053	27,1164	27,0322	+	20	20	20	49,2	49,2	49,2	56,106	6,31E+09	156	26,9816	26,9364	27,0269	10	5	5	0,0215854	0,0905125	F9UL36	F9UL36	nadC1	Nicotinate phosphoribosyltransferase
26,4908	26,4641	26,4738	26,4704	26,5376	26,5398	26,5829	26,561	26,6381	26,5711	+	18	18	18	67,2	67,2	67,2	34,228	4,78E+09	122	26,533	26,4873	26,5786	10	5	5	0,00259334	0,0912422	Q88VG6	Q88VG6	rnz	Ribonuclease Z
31,581	31,5933	31,7224	31,6285	31,5913	31,7296	31,6855	31,7615	31,6631	31,7361	+	57	57	57	81,9	81,9	81,9	77,012	3,67E+11	2694	31,6692	31,6233	31,7152	10	5	5	0,0196241	0,091864	Q88XY8	Q88XY8	fusA	Elongation factor G
29,3647	29,3337	29,3657	29,3852	29,332	29,4908	29,5414	29,3236	29,4432	29,476	+	14	14	14	67,4	67,4	67,4	24,495	2,98E+10	279	29,4056	29,3563	29,455	10	5	5	0,0312724	0,0987511	Q88V23	Q88V23	dapH	2,3,4,5-tetrahydropyridine-2,6-dicarboxylate N-acetyltransferase
27,7902	27,8356	27,8376	27,7416	27,8453	27,8643	27,8673	27,9204	27,9774	27,9369	+	14	14	14	63,3	63,3	63,3	32,203	1,27E+10	207	27,8617	27,8101	27,9133	10	5	5	0,00757805	0,103203	F9UPE3	F9UPE3	lp_1796	DegV family protein
30,9081	30,7954	30,8204	30,8879	30,9464	30,9335	30,9543	30,9523	31,01	31,038	+	11	11	11	52,8	52,8	52,8	19,35	8,77E+10	522	30,9246	30,8717	30,9776	10	5	5	0,0147989	0,105964	Q88XX1	Q88XX1	rplF	50S ribosomal protein L6
27,8641	27,9502	27,9728	28,0188	28,0566	27,9505	28,0825	28,1617	28,1197	28,1121	+	19	19	19	41,3	41,3	41,3	49,762	1,38E+10	264	28,0289	27,9725	28,0853	10	5	5	0,0490138	0,112826	F9UQS7	F9UQS7	murT	ipid II isoglutaminyl synthase (glutamine-hydrolyzing) subunit MurT
29,7586	29,7599	29,6876	29,721	29,7148	29,8072	29,7774	29,8804	29,916	29,8281	+	8	8	8	48	48	48	19,648	1,93E+10	125	29,7851	29,7284	29,8418	10	5	5	0,0041333	0,113414	Q88WU8	Q88WU8	infC	Translation initiation factor IF-3
26,5785	26,7881	26,7837	26,6899	26,6502	26,881	26,7461	26,8567	26,8332	26,7511	+	20	20	20	40,4	40,4	40,4	52,974	6,26E+09	175	26,7558	26,6981	26,8136	10	5	5	0,0448782	0,115557	F9UTT6	F9UTT6	pepD2	Dipeptidase
31,4251	31,5091	31,3938	31,432	31,5176	31,6444	31,6361	31,4798	31,5543	31,5412	+	10	10	10	70,2	70,2	70,2	14,76	7,02E+10	863	31,5133	31,4555	31,5711	10	5	5	0,0189388	0,115641	Q88YX0	Q88YX0	rplK	50S ribosomal protein L11
26,0726	26,1015	26,0742	26,0166	25,9256	26,0951	26,2087	26,1678	26,1567	26,1478	+	11	11	11	20	20	20	74,521	3,40E+09	108	26,0967	26,0381	26,1552	10	5	5	0,0120896	0,117097	Q88XQ0	Q88XQ0	ligA	DNA ligase
32,6451	32,7455	32,8131	32,8519	32,8933	32,9362	32,9722	32,8117	32,9388	32,8885	+	22	22	22	71,3	71,3	71,3	38,728	5,12E+11	2452	32,8496	32,7898	32,9095	10	5	5	0,0493887	0,119681	F9ULE0	F9ULE0	lox	NAD-independent L-lactate dehydrogenase
29,6055	29,6863	29,6223	29,7162	29,661	29,7019	29,7685	29,8152	29,8404	29,7691	+	14	14	14	42,1	42,1	42,1	29,868	1,46E+10	159	29,7186	29,6583	29,779	10	5	5	0,00473486	0,120737	F9UM85	F9UM85	glnH1	Glutamine ABC transporter, substrate binding protein
28,1499	28,2526	28,3113	28,2714	28,2173	28,2999	28,2973	28,3935	28,3975	28,4242	+	37	37	37	37,4	37,4	37,4	94,522	3,20E+10	445	28,3015	28,2405	28,3625	10	5	5	0,012555	0,121996	F9US38	F9US38	gyrA	DNA gyrase subunit A
27,025	27,1228	27,0461	27,1073	27,0442	27,088	27,2413	27,2267	27,2239	27,1827	+	11	11	11	33,2	33,2	33,2	31,471	3,70E+09	142	27,1308	27,0691	27,1925	10	5	5	0,00657448	0,123458	Q88Z30	Q88Z30	hslO	33 kDa chaperonin
28,2089	28,0769	28,1592	28,1255	28,2273	28,3586	28,3011	28,2114	28,322	28,224	+	13	13	13	34,3	34,3	34,3	41,52	1,29E+10	197	28,2215	28,1596	28,2834	10	5	5	0,0139064	0,123822	F9US34	F9US34	dnaN	Beta sliding clamp
29,9366	30,0966	30,0109	29,9496	29,9498	30,033	30,1239	30,1251	30,2302	30,0523	+	26	26	26	79,6	79,6	79,6	34,842	6,83E+10	652	30,0508	29,9887	30,1129	10	5	5	0,0266151	0,12419	Q88XW0	Q88XW0	rpoA	DNA-directed RNA polymerase subunit alpha

26,7323	26,6972	26,7529	26,7344	26,8696	26,8881	26,8365	26,893	26,9265	26,8681	+		5	5	5	15,9	15,9	15,9	46,589	2,13E+09	73	26,8199	26,7573	26,8824	10	5	5	0,00530131	0,125133	Q88UH9	Q88UH9	lp_2507	UPF0210 protein lp_2507
30,2371	30,1972	30,3536	30,2404	30,3299	30,2713	30,3831	30,48	30,4442	30,4055	+		37	37	37	66,9	66,9	66,9	65,466	1,16E+11	977	30,3342	30,2716	30,3968	10	5	5	0,0270961	0,125193	Q88YE7	Q88YE7	glmS	Glutamine--fructose-6-phosphate aminotransferase [isomerizing]
27,2185	27,1857	27,3103	27,1244	27,1617	27,2386	27,2691	27,4221	27,3272	27,3741	+		35	35	35	49,5	49,5	49,5	85,09	1,09E+10	261	27,2632	27,2001	27,3262	10	5	5	0,0254088	0,126115	F9UMU5	F9UMU5	pcrA	ATP-dependent DNA helicase
30,2645	30,0807	30,1493	30,2961	30,2973	30,3145	30,3682	30,2469	30,3989	30,3918	+		11	11	11	32,9	32,9	32,9	32,619	4,12E+10	208	30,2808	30,2176	30,3441	10	5	5	0,0413951	0,126475	Q88X05	Q88X05	prsA1	Foldase protein PrsA 1
28,1609	28,2589	28,3163	28,2398	28,3141	28,2766	28,376	28,4676	28,4161	28,387	+		40	40	40	53,9	53,9	53,9	89,557	4,43E+10	545	28,3213	28,258	28,3847	10	5	5	0,017377	0,126656	Q88YL7	Q88YL7	secA	Protein translocase subunit SecA
27,0481	27,0904	27,1223	27,0128	27,1775	27,1379	27,1826	27,2193	27,3184	27,2353	+		8	8	8	37,3	37,3	37,3	34,446	3,89E+09	115	27,1545	27,0902	27,2187	10	5	5	0,0147704	0,128488	F9URL2	F9URL2	lp_2738	L-asparaginase
26,6192	26,5538	26,5195	26,6255	26,615	26,8229	26,7286	26,7053	26,641	26,6827	+		7	7	7	42,8	42,8	42,8	18,207	4,10E+09	76	26,6513	26,5866	26,7161	10	5	5	0,00808217	0,129508	F9UNC5	F9UNC5	lp_1360	Transcription regulator, MarR family
29,9514	29,947	30,0656	30,0076	29,9507	30,045	30,0324	30,2078	30,1391	30,1524	+		38	38	38	73,9	73,9	73,9	68,468	7,76E+10	626	30,0499	29,9845	30,1153	10	5	5	0,0122647	0,130874	F9UQ86	F9UQ86	typA	50S ribosomal subunit assembly factor BipA
25,4518	25,5438	25,425	25,3965	25,5461	25,5613	25,5953	25,746	25,6005	25,5189	+		5	5	5	50	50	50	9,4896	1,34E+09	44	25,5385	25,4727	25,6044	10	5	5	0,0278442	0,131746	F9UP62	F9UP62	lp_1699	Uncharacterized protein
31,6767	31,7553	31,6246	31,7142	31,7946	31,8584	31,8488	31,8312	31,8808	31,8054	+		21	21	21	61,6	61,6	61,6	24,77	1,28E+11	658	31,779	31,7131	31,8449	10	5	5	0,00350553	0,131826	Q88YW9	Q88YW9	rplA	50S ribosomal protein L1
31,6693	31,7877	31,8131	31,7665	31,8513	31,867	31,9026	31,9324	31,9828	31,8632	+		24	24	24	46,8	46,8	46,8	54,521	1,83E+11	1166	31,8436	31,7776	31,9096	10	5	5	0,00814413	0,132	Q88UU1	Q88UU1	atpA	ATP synthase subunit alpha
26,8531	26,8479	26,918	26,7993	26,9527	26,9114	26,99	27,0416	26,9753	27,1227	+		15	15	15	31,7	31,7	31,7	72,67	5,79E+09	121	26,9412	26,8742	27,0082	10	5	5	0,0170149	0,133977	F9UNU7	F9UNU7	pbp2x	Transpeptidase (Penicillin binding protein 2B)
29,6462	29,7971	29,8231	29,7501	29,8944	29,7975	29,9056	29,9564	29,981	29,9466	+		26	26	26	61,3	61,3	61,3	47,904	5,30E+10	535	29,8498	29,7822	29,9174	10	5	5	0,0326272	0,135229	Q88VQ7	Q88VQ7	hisS	Histidine--tRNA ligase
26,9267	27,1975	27,1761	27,1424	27,1596	27,1589	27,2135	27,278	27,3442	27,2954	+		12	12	12	45	45	45	31,342	7,58E+09	141	27,1892	27,1205	27,258	10	5	5	0,048055	0,137541	F9UM25	F9UM25	lp_0805	Hydrolase, HAD superfamily, Cof family
25,8139	25,897	25,9123	25,8441	25,9104	25,9435	25,8498	26,0761	26,0827	26,1186	+		4	4	4	33,9	33,9	33,9	17,794	9,18E+08	22	25,9448	25,8755	26,0142	10	5	5	0,0343337	0,138638	F9UN94	F9UN94	lp_1322	Nucleotide-binding protein, universal stress protein UspA family
25,6113	25,8609	25,7441	25,8372	25,7871	25,8931	25,865	25,9252	26,0335	25,8266	+		4	4	4	17,4	17,4	17,4	28,978	9,38E+08	31	25,8384	25,7681	25,9087	10	5	5	0,0374125	0,140567	F9UU19	F9UU19	lp_0433	Carbon-nitrogen hydrolase family protein
28,7836	28,8533	28,9253	28,8021	28,7755	28,9171	28,955	28,9046	29,1161	28,9791	+		14	14	14	56,5	56,5	56,5	27,479	1,51E+10	182	28,9012	28,8279	28,9744	10	5	5	0,0143116	0,146429	F9UL77	F9UL77	trmH	tRNA/rRNA methyltransferase, TrmH family
30,8884	30,9177	30,9529	30,9902	31,0335	31,0646	31,0698	31,0755	31,1362	31,1695	+		15	15	15	49,3	49,3	49,3	22,637	1,01E+11	481	31,0298	30,9565	31,1031	10	5	5	0,00224966	0,146592	Q88XY5	Q88XY5	rplD	50S ribosomal protein L4
29,7681	30,0398	30,0464	30,04	30,0508	30,089	30,2101	30,0868	30,1852	30,1071	+		23	23	23	68	68	68	35,145	6,75E+10	548	30,0623	29,989	30,1356	10	5	5	0,0429555	0,14665	F9UQM6	F9UQM6	mreB1	Cell shape-determining protein MreB
30,8337	30,7629	30,797	30,8662	30,9331	30,837	30,9303	31,0067	31,0945	31,0605	+		29	29	29	86,6	86,6	86,6	22,963	1,26E+11	867	30,9122	30,8386	30,9858	10	5	5	0,0278401	0,147221	Q88UX0	Q88UX0	rpsD	30S ribosomal protein S4
29,6384	29,7157	29,7693	29,6917	29,7871	29,7637	29,8647	29,886	29,9378	29,8981	+		41	41	41	66,4	66,4	66,4	57,457	9,56E+10	1011	29,7952	29,7204	29,87	10	5	5	0,00541293	0,14959	Q88Y74	Q88Y74	guaA	GMP synthase [glutamine-hydrolyzing]
26,9896	27,0474	26,9223	27,0014	26,9542	26,9764	27,0553	27,189	27,2691	27,1738	+		8	8	8	58,9	58,9	58,9	22,376	2,23E+09	36	27,0578	26,983	27,1327	10	5	5	0,0283845	0,149739	Q88VE3	Q88VE3	engB	Probable GTP-binding protein EngB
25,9314	26,1038	25,9159	25,9353	26,1439	26,1202	26,0368	26,1543	26,2728	26,1977	+		4	4	4	10,8	10,8	10,8	45,841	1,04E+09	41	26,0812	26,0061	26,1564	10	5	5	0,0428694	0,150291	F9UQ04	F9UQ04	lp_2049	Zinc metalloprotease
27,0131	27,0628	27,015	27,0205	27,0547	27,1367	27,1128	27,3455	27,1861	27,1412	+		6	6	6	20,3	20,3	20,3	31,764	3,45E+09	78	27,1088	27,0332	27,1845	10	5	5	0,00814235	0,151264	F9UTU3	F9UTU3	tagG	Transport permease protein
27,154	27,4261	27,4708	27,453	27,4576	27,5013	27,5952	27,5802	27,5193	27,5245	+		14	14	14	41	41	41	40,841	9,53E+09	185	27,4682	27,3923	27,5441	10	5	5	0,0419549	0,151777	Q88ZH4	Q88ZH4	tagH	Teichoic acids export ATP-binding protein TagH
27,644	27,7413	27,7928	27,7184	27,6917	27,8961	27,9685	27,7996	27,8487	27,8377	+		12	12	12	52,7	52,7	52,7	25,514	9,21E+09	177	27,7939	27,7177	27,8701	10	5	5	0,00397798	0,152472	F9ULX8	F9ULX8	phoU	Phosphate-specific transport system accessory protein PhoU
32,5166	32,5939	32,6051	32,6137	32,5997	32,6826	32,7627	32,7219	32,7644	32,76	+		28	28	28	81,5	81,5	81,5	30,921	4,56E+11	2278	32,6621	32,5858	32,7383	10	5	5	0,00020668	0,152498	F9UTT2	F9UTT2	fba	Fructose-bisphosphate aldolase
27,9178	27,9457	28,0154	27,9953	28,043	27,9986	28,1037	28,2221	28,2318	28,1252	+		18	18	18	66,3	66,3	66,3	27,461	2,13E+10	341	28,0599	27,9834	28,1363	10	5	5	0,0135977	0,152851	F9UM84	F9UM84	glnQ2	Glutamine ABC transporter, ATP-binding protein
28,1574	28,1461	28,1153	28,1396	28,1672	28,1851	28,4306	28,2296	28,3798	28,2688	+		11	11	11	59,9	59,9	59,9	18,893	1,16E+10	160	28,2219	28,1451	28,2988	10	5	5	0,0113023	0,153706	Q88VH0	Q88VH0	apt	Adenine phosphoribosyltransferase
24,7963	24,8675	24,7567	24,9301	24,7539	24,8195	24,9968	24,9911	25,1455	24,9269	+		13	13	13	27,7	27,7	27,7	62,876	1,44E+09	92	24,8984	24,8209	24,9759	10	5	5	0,0394933	0,155021	F9USG5	F9USG5	lp_3042	Multidrug ABC transporter, ATP-binding and permease protein
26,7558	26,8711	26,9434	26,8913	26,844	26,9249	26,9631	27,0231	27,112	27,0603	+		6	6	6	24,5	24,5	24,5	34,557	4,74E+09	93	26,9389	26,8611	27,0167	10	5	5	0,0091457	0,155548	F9UQJ8	F9UQJ8	lp_2279	Phosphoesterase, DHH family
27,0307	26,9838	27,0991	27,0785	27,0597	26,9945	27,1989	27,2088	27,36	27,2673	+		12	12	12	31	31	31	43,601	3,89E+09	103	27,1281	27,0503	27,2059	10	5	5	0,0396394	0,15557	F9UL63	F9UL63	lp_0593	Transcriptional attenuator, cell envelope-related, LytR family
25,8501	25,765	25,9558	25,9428	25,931	25,9406	26,0059	26,0367	26,1355	26,1277	+		9	9	9	36	36	36	35,249	2,44E+09	77	25,9691	25,8889	26,0493	10	5	5	0,0146356	0,16034	F9UNQ0	F9UNQ0	dnaI	Primosomal protein DnaI
26,9068	26,7828	26,8203	26,7662	27,0279	27,051	27,0061	27,0645	26,9785	27,0091	+		16	16	16	24,5	24,5	24,5	79,954	4,72E+09	117	26,9413	26,8608	27,0218	10	5	5	0,013226	0,161033	Q6LWF5	Q6LWF5	traI	DNA topoisomerase
25,8025	25,9184	25,9682	26,0225	26,0744	25,9839	26,1076	26,1067	26,2212	26,1727	+		8	8	8	24,8	24,8	24,8	51,78	2,21E+09	62	26,0378	25,9572	26,1184	10	5	5	0,0304177	0,161207	F9URG0	F9URG0	lp_2671	Transport protein with CBS domains, DUF21 family
24,7809	24,8778	24,8409	24,8064	25,0406	24,9848	25,0822	25,0806	25,0444	24,9642	+		2	2	2	15,8	15,8	15,8	26,804	3,66E+08	13	24,9503	24,8693	25,0312	10	5	5	0,0142321	0,161907	F9UTN9	F9UTN9	rrp2	Two-component system response regulator
31,508	31,6444	31,5137	31,6075	31,6396	31,549	31,7698	31,7211	31,8819	31,8063	+		13	13	13	76,9	76,9	76,9	13,638	1,29E+11	522	31,6641	31,5826	31,7456	10	5	5	0,0328487	0,162995	Q88XW2	Q88XW2	rpsM	30S ribosomal protein S13
31,313	31,5917	31,4566	31,4189	31,51	31,4565	31,5986	31,6375	31,7276	31,6909	+		12	12	12	66,7	66,7	66,7	11,736	1,10E+11	802	31,5401	31,458	31,6222	10	5	5	0,0377524	0,164184	Q88XY7	Q88XY7	rpsJ	30S ribosomal protein S10
28,5046	28,5674	28,6299	28,5356	28,6176	28,6628	28,6998	28,7721	28,8489	28,6968	+		10	10	10	45,2	45,2	45,2	35,961	1,40E+10	169	28,6535	28,571	28,7361	10	5	5	0,00379739	0,165062	Q88V90	Q88V90	dapF	Diaminopimelate epimerase
26,48	26,6027	26,5723	26,4325	26,6075	26,5973	26,6863	26,7891	26,7612	26,6914	+		24	24	24	47,6	47,6	47,6	74,567	8,25E+09	217	26,622	26,539	26,7051	10	5	5	0,00903668	0,166047	F9UPH6	F9UPH6	parE	DNA topoisomerase 4 subunit B
29,0809	29,3403	29,2976	29,2835	29,3095	29,3083	29,4778	29,4844	29,5213	29,3554	+		25	25	25	68,6	68,6	68,6	41,346	4,76E+10	489	29,3459	29,2624	29,4294	10	5	5	0,027276	0,167087	Q88UV8	Q88UV8	ddl	D-alanine--D-alanine ligase
30,4558	30,6288	30,5806	30,6517	30,6375	30,6222	30,7372	30,7953	30,8268	30,81	+		10	10	10	67,2	67,2	67,2	13,148	7,21E+10	280	30,6746	30,5909	30,7583	10	5	5	0,0118652	0,167405	Q88XX6	Q88XX6	rplN	50S ribosomal protein L14
27,8452	28,0592	28,0156	28,0231	28,07	28,1234	28,1709	28,2305	28,2725	28,0564	+		11	11	11	26,2	26,2	26,2	53,573	1,01E+10	178	28,0867	28,0026	28,1707	10	5	5	0,0167807	0,168128	F9UU99	F9UU99	gadB	Glutamate decarboxylase
27,7877	27,659	27,7543	27,7008	27,7782	27,7526	27,8583	27,9928	27,922	27,9971	+		23	23	23	36,1	36,1	36,1	88	1,24E+10	292	27,8203	27,736	27,9046	10	5	5	0,0117278	0,168549	F9UMB1	F9UMB1	lp_0910	DNA helicase
29,0508	29,2755	29,3175	29,3229	29,2187	29,299	29,4618	29,4478	29,398	29,4251	+		10	10	10	26,6	26,6	26,6	33,733	2,42E+10	263	29,3217	29,2371	29,4063	10	5	5	0,0192004	0,169255	F9UNV2	F9UNV2	lp_1573	Glucokinase
27,296	27,5327	27,5148	27,5664	27,4791	27,5568	27,6353	27,7136	27,6664	27,6683	+		12	12	12	31,2	31,2	31,2	43,845	9,51E+09	141	27,5629	27,4778	27,6481	10	5	5	0,0138095	0,170301	F9UTR7	F9UTR7	ndh1	NADH dehydrogenase
29,4445	29,5995	29,6597	29,5696	29,6417	29,7684	29,7982	29,7457	29,7708	29,6938	+		18	18	18	45,9	45,9	45,9	48,258	5,60E+10	367	29,6692	29,583	29,7554	10	5	5	0,0033688	0,172372	F9UTG0	F9UTG0	gshR4	Glutathione reductase
25,3712	25,5998	25,6765	25,5406	25,4902	25,7074	25,736	25,6373	25,6313	25,8311	+		4	4	4	28,8	28,8	28,8	34,391	1,03E+09	33	25,6221	25,5356	25,7086	10	5	5	0,0255726	0,172998	Q88YZ4	Q88YZ4	fabH1	3-oxoacyl-[acyl-carrier-protein] synthase 3 protein 1
24,8252	24,7983	24,8268	24,7938	24,9044	24,9013	25,0211	25,0863	24,9654	25,0399	+		2	2	2	14	14	14	12,985	4,23E+08	21	24,9163	24,8297	25,0028	10	5	5	0,00174576	0,173109	F9UUA1	F9UUA1	lp_3422	Uncharacterized protein
28,3111	28,6528	28,4987	28,4404	28,5581	28,5891	28,6828	28,7358	28,7265	28,6022	+		7	7	7	35,7	35,7	35,7	20,593	9,35E+09	93	28,5797	28,4922	28,6673	10	5	5	0,0273241	0,175071	F9UL82	F9UL82	nusG	Transcription termination/antitermination protein NusG
27,4835	27,6438	27,6518	27,6481	27,6735	27,7325	27,9412	27,8114	27,8637	27,6381	+		16	16	16	47,3	47,3	47,3	33,156	8,33E+09	148	27,7088	27,6201	27,7974	10	5	5	0,0223248	0,17724	F9UTA7	F9UTA7	parB1	Chomosome partitioning protein, DNA-binding protein
26,806	26,8737	26,8195	26,7863	26,9454	26,9455	27,0077	27,055	27,148	26,9752	+		20	20	20	53,9	53,9	53,9	50,653	6,50E+09	179	26,9362	26,8462	27,0263	10	5	5	0,00425155	0,180108	F9UQJ7	F9UQJ7	rhe3	DEAD-box ATP-dependent RNA helicase CshB
26,627	26,7946	26,8108	26,7123	26,6858	26,7928	27,0054	26,9475	26,9518	26,8501	+		10	10	10	31,6	31,6	31,6	44,218	3,81E+09	67	26,8178	26,7261	26,9095	10	5	5	0,00740123	0,183413	F9UN61	F9UN61	lp_1275	1,2-diacylglycerol 3-glucosyltransferase
26,3029	26,4712	26,6189	26,5364	26,6589	26,6281	26,6074	26,8627	26,7562	26,6614	+		6	6	6	25,4	25,4	25,4	31,855	1,64E+09	53	26,6104	26,5177	26,7032	10	5	5	0,0460337	0,185478	F9UU35	F9UU35	lp_0450	Uncharacterized protein
27,3313	27,3338	27,469	27,4366	27,3148	27,4124	27,5728	27,6377	27,6107	27,5825	+		15	15	15	64,4	64,4	64,4	30,564	9,81E+09	200	27,4702	27,3771	27,5632	10	5	5	0,00612	0,186129	F9UKV2	F9UKV2	purR	Pur operon repressor
26,2022	26,1966	26,3134	26,2589	26,202	26,2487	26,5221	26,4308	26,5723	26,3344	+		22	22	22	37,1	37,1	37,1	87,219	5,04E+09	155	26,3281	26,2346	26,4217	10	5	5	0,0186072	0,18703	Q88V16	Q88V16	mutS2	Endonuclease MutS2
25,6412	25,7702	25,7986	25,7732	25,9891	25,883	26,0058	26,0249	26,1089	25,8885	+		7	7	7	24,5	24,5	24,5	46,68	2,41E+09	82	25,8883	25,7944	25,9822	10	5	5	0,0286844	0,187796	F9UQL7	F9UQL7	lp_2306	Non-proteolytic protein, peptidase family M16
25,6546	25,5908	25,3962	25,5065	25,485	25,785	25,8008	25,6468	25,7341	25,6057	+		4	4	4	24,2	24,2	24,2	17,724	9,20E+08	29	25,6205	25,5266	25,7145	10	5	5	0,0125743	0,187892	F9UTS5	F9UTS5	lp_0321	Acetyltransferase, GNAT family
30,8412	30,9161	30,8092	30,9389	30,9261	31,0635	31,0912	31,0519	31,085	31,0794	+		18	18	18	60,8	60,8	60,8	15,344	8,25E+10	660	30,9802	30,8863	31,0742	10	5	5	0,00010864	0,187897	Q88XW7	Q88XW7	rplO	50S ribosomal protein L15
27,7169	27,8516	27,7764	27,6346	27,5527	27,8282	27,8016	27,9945	28,0247	27,8286	+		2	2	2	8,6	8,6	8,6	28,752	3,37E+09	34	27,801	27,7065	27,8955	10	5	5	0,0277295	0,189052	F9US55	F9US55	lp_0026	Hydrolase, HAD superfamily, Cof family
27,5795	27,5969	27,4629	27,5482	27,6342	27,6619	27,8963	27,7324	27,7473	27,7331	+		3	3	3	5	5	5	80,859	2,06E+09	26	27,6593	27,5643	27,7542	10	5	5	0,00428065	0,189862	F9ULI3	F9ULI3	lp_3634	Alpha-1,2-mannosidase
28,2041	28,4179	28,3661	28,4417	28,4161	28,4786	28,4856	28,5892	28,6545	28,5952	+		7	7	7	35,7	35,7	35,7	42,124	8,40E+09	107	28,4649	28,3692	28,5606	10	5	5	0,00822817	0,191465	Q88V24	Q88V24	lp_2263	N-acetyldiaminopimelate deacetylase
28,0277	28,2991	28,2079	28,1536	28,0877	28,1715	28,3928	28,3515	28,4745	28,3499	+		10	10	10	8,1	8,1	8,1	143,32	1,10E+10	156	28,2516	28,1552	28,348	10	5	5	0,0224804	0,192859	F9UTQ0	F9UTQ0	lp_0295	Transport protein, MMPL family
25,4216	25,4774	25,4707	25,4012	25,462	25,5388	25,5365	25,8439	25,6697	25,62	+		3	3	3	10,4	10,4	10,4	32,929	9,54E+08	36	25,5442	25,4466	25,6418	10	5	5	0,0102158	0,195187	F9UR45	F9UR45	lp_2520	Extracellular protein, NlpC/P60 family,gamma-D-glutamate-meso-diaminopimelate muropeptidase
27,989	28,0447	28,021	28,0202	28,0715	28,0459	28,1984	28,3264	28,2994	28,256	+		20	20	20	28,7	28,7	28,7	79,042	1,20E+10	251	28,1272	28,0293	28,2252	10	5	5	0,00528178	0,195929	F9UN67	F9UN67	lp_1283	Membrane sulfatase, alkaline phosphatase superfamily protein
27,9566	28,0242	28,0694	28,1064	28,2104	28,17	28,3567	28,3057	28,2952	28,2233	+		8	8	8	30	30	30	31,341	6,10E+09	81	28,1718	28,0734	28,2702	10	5	5	0,00632852	0,196771	F9UQL5	F9UQL5	lp_2304	Transcription regulator, Xre family
27,454	27,4649	27,5174	27,3774	27,611	27,5722	27,7231	27,719	27,7234	27,674	+		25	25	25	76,1	76,1	76,1	47,764	9,64E+09	250	27,5836	27,4849	27,6823	10	5	5	0,00351832	0,197413	F9UM99	F9UM99	hflX	GTPase HflX
28,4483	28,5702	28,6212	28,6306	28,638	28,6048	28,7123	28,91	28,8725	28,7989	+		15	15	15	56,6	56,6	56,6	37,408	1,57E+10	240	28,6807	28,5817	28,7797	10	5	5	0,0166331	0,198012	Q88WK2	Q88WK2	plsX	Phosphate acyltransferase
29,3545	29,3681	29,2223	29,2366	29,4334	29,5242	29,4875	29,5582	29,5505	29,5	+		7	7	7	22,2	22,2	22,2	37,332	1,54E+10	132	29,4235	29,323	29,5241	10	5	5	0,00154146	0,201117	F9UTJ4	F9UTJ4	lp_3312	Beta-lactamase family protein
26,3733	26,5759	26,5745	26,5543	26,4291	26,5297	26,7535	26,7979	26,7882	26,6455	+		13	13	13	62,7	62,7	62,7	25,943	2,77E+09	70	26,6022	26,5014	26,703	10	5	5	0,0158857	0,201526	F9UQE7	F9UQE7	lp_2223	GTP pyrophosphokinase
27,2724	27,599	27,488	27,4858	27,4646	27,552	27,7152	27,7433	27,7126	27,5994	+		13	13	13	43,6	43,6	43,6	45,154	8,13E+09	128	27,5632	27,462	27,6645	10	5	5	0,0141001	0,202545	P59603	P59603	argG	Argininosuccinate synthase
26,7396	27,0694	26,9166	26,9737	26,9941	27,0684	27,1434	27,1355	27,2737	27,0952	+		10	10	10	38,3	38,3	38,3	36,538	5,70E+09	122	27,0409	26,9387	27,1432	10	5	5	0,0144459	0,204549	F9UQE3	F9UQE3	lp_2219	6-phosphogluconolactonase
25,9298	25,9991	25,7509	25,9229	25,8237	25,8406	26,0257	26,1145	26,2644	26,2041	+		5	5	5	44,7	44,7	44,7	13,167	1,26E+09	28	25,9876	25,8853	26,0898	10	5	5	0,0449863	0,204576	Q88RX0	Q88RX0	rnpA	Ribonuclease P protein component
29,0521	29,2228	29,2838	29,239	29,2916	29,3517	29,4015	29,445	29,4166	29,4974	+		30	30	30	56,7	56,7	56,7	64,231	5,31E+10	559	29,3201	29,2178	29,4224	10	5	5	0,00334656	0,204615	F9USW0	F9USW0	lai	inoleic acid isomerase
28,9903	29,1323	29,1945	29,1506	29,2462	29,2199	29,2941	29,3885	29,4497	29,388	+		20	20	19	49,9	49,9	46,1	43,835	2,37E+10	308	29,2454	29,1428	29,3481	10	5	5	0,00833359	0,205289	F9UN65	F9UN65	araT1	Aminotransferase
26,7167	26,726	26,8229	26,7321	26,7919	26,9203	27,0344	27,008	26,9438	26,9121	+		24	24	24	44,8	44,8	44,8	59,561	6,96E+09	217	26,8608	26,7579	26,9637	10	5	5	0,00020892	0,205787	Q88XF3	Q88XF3	prfC	Peptide chain release factor 3
25,3886	25,4574	25,6912	25,6148	25,6256	25,7623	25,8461	25,7549	25,839	25,6067	+		7	7	7	22,1	22,1	22,1	50,181	1,14E+09	48	25,6587	25,5555	25,7618	10	5	5	0,0200504	0,206235	F9UKZ5	F9UKZ5	murF	UDP-N-acetylmuramoyl-tripeptide--D-alanyl-D-alanine ligase
29,1479	29,153	29,2931	29,2187	29,2745	29,3471	29,4859	29,4776	29,4549	29,3545	+		5	5	5	22,2	22,2	22,2	28,36	8,64E+09	115	29,3207	29,2174	29,424	10	5	5	0,00128356	0,206571	F9URD0	F9URD0	lp_2631	ipase/esterase
26,5115	26,7539	26,8011	26,6711	26,7457	26,7603	26,9983	26,8986	27,0071	26,8577	+		6	6	6	27,1	27,1	27,1	36,994	3,39E+09	67	26,8005	26,6967	26,9044	10	5	5	0,0162588	0,207721	F9UMV1	F9UMV1	lp_1150	Diacylglycerol kinase family protein
25,2075	25,2773	25,282	25,2579	25,3134	25,3703	25,3558	25,6494	25,5783	25,4432	+		8	8	8	32,3	32,3	32,3	38,586	1,32E+09	39	25,3735	25,2676	25,4794	10	5	5	0,00809932	0,21177	F9UNL3	F9UNL3	fecB	Iron chelatin ABC transporter, substrate binding protein
24,8982	24,7588	24,872	24,8076	24,9186	24,8998	24,9177	25,1661	25,191	25,1397	+		4	4	4	15,5	15,5	15,5	43,738	6,83E+08	7	24,957	24,8511	25,0628	10	5	5	0,0165241	0,211785	F9UT99	F9UT99	hpk11	Histidine kinase
28,3033	28,3075	28,4709	28,4251	28,4753	28,3968	28,6182	28,6864	28,7906	28,555	+		3	3	3	14,3	14,3	14,3	29,645	7,42E+09	75	28,5029	28,3964	28,6094	10	5	5	0,0233942	0,212976	F9UTF3	F9UTF3	lp_3256	DegV family protein
26,3744	26,4181	26,4528	26,3236	26,382	26,483	26,6792	26,6528	26,6134	26,5878	+		14	14	14	21,7	21,7	21,7	95,311	4,40E+09	113	26,4967	26,3902	26,6033	10	5	5	0,00073983	0,213083	F9UL38	F9UL38	pacL2	Cation transporting P-type ATPase
27,7453	27,9609	27,9214	27,9166	27,7304	27,9974	28,0676	28,0212	28,0612	28,1979	+		5	5	5	25,6	25,6	25,6	29,713	7,10E+09	94	27,962	27,8549	28,069	10	5	5	0,00706414	0,214126	Q88V19	Q88V19	murI	Glutamate racemase
30,8754	30,9666	31,1213	31,0036	30,9295	31,0129	31,1094	31,2563	31,2586	31,3386	+		12	12	12	44,4	44,4	44,4	26,881	9,15E+10	546	31,0872	30,9793	31,1952	10	5	5	0,0168892	0,215899	F9UP45	F9UP45	fabI	Enoyl-[acyl-carrier-protein] reductase [NADH]
29,9807	30,0784	30,1456	30,0962	30,2331	30,1493	30,345	30,4165	30,415	30,2908	+		15	15	15	39,4	39,4	39,4	63,794	5,79E+10	357	30,2151	30,1068	30,3233	10	5	5	0,00997183	0,216491	F9ULE1	F9ULE1	pox4	Pyruvate oxidase
28,7176	28,837	28,6933	28,7911	28,8228	28,9919	28,9594	28,9714	29,098	28,9288	+		12	12	12	34,1	34,1	34,1	32,529	1,40E+10	188	28,8811	28,7724	28,9899	10	5	5	0,00068347	0,217526	F9UQQ1	F9UQQ1	hicD3	L-2-hydroxyisocaproate dehydrogenase
28,7191	28,7605	28,826	28,779	28,9552	28,8893	28,9639	29,0676	29,1067	29,1002	+		9	9	9	28,4	28,4	28,4	43,784	2,00E+10	211	28,9167	28,808	29,0255	10	5	5	0,00607489	0,217579	F9UP94	F9UP94	aspAT	Aminotransferase
27,1367	27,0861	27,1544	27,1997	27,2531	27,1892	27,3859	27,4168	27,4683	27,4604	+		14	14	14	56,6	56,6	56,6	30,127	7,74E+09	144	27,2751	27,166	27,3841	10	5	5	0,0057115	0,218131	F9UQM5	F9UQM5	mreC	Cell shape-determining protein MreC
29,5816	29,6192	29,8265	29,7412	29,9176	29,7581	29,9147	29,9822	30,0364	30,0957	+		18	18	18	57,4	57,4	57,4	48,255	3,68E+10	322	29,8473	29,7372	29,9574	10	5	5	0,0328464	0,220211	F9UR64	F9UR64	npr2	NADH peroxidase
31,5419	31,4917	31,4449	31,4301	31,5245	31,7107	31,7376	31,754	31,6214	31,721	+		11	11	11	73,2	73,2	73,2	14,166	6,83E+10	289	31,5978	31,4866	31,7089	10	5	5	0,00011136	0,222344	Q88XV9	Q88XV9	rplQ	50S ribosomal protein L17
28,7731	28,8593	28,8069	28,858	28,7427	29,0106	29,15	29,1322	29,0844	28,7771	+		2	2	2	8,4	8,4	8,4	38,349	5,86E+09	10	28,9194	28,808	29,0309	10	5	5	0,0144638	0,222859	F9UUF3	F9UUF3	galM3	Aldose 1-epimerase
28,1938	28,4579	28,4612	28,4787	28,482	28,5128	28,5747	28,7949	28,7532	28,5581	+		9	9	9	24,2	24,2	24,2	47,28	1,13E+10	144	28,5267	28,4147	28,6387	10	5	5	0,0221837	0,22401	F9UTW6	F9UTW6	gshR1	Glutathione reductase
27,3519	27,4525	27,3389	27,2882	27,3291	27,5222	27,5856	27,5159	27,6857	27,5738	+		8	8	8	55	55	55	21,461	3,46E+09	70	27,4644	27,3521	27,5766	10	5	5	0,00058405	0,224508	Q88ST4	Q88ST4	folE	GTP cyclohydrolase 1
25,0309	25,5599	25,2913	25,094	25,302	25,5137	25,4742	25,4878	25,5246	25,4116	+		6	6	6	31,9	31,9	31,9	32,302	9,89E+08	42	25,369	25,2556	25,4824	10	5	5	0,0440758	0,226759	Q88XV1	Q88XV1	ecfA2	Energy-coupling factor transporter ATP-binding protein EcfA2
24,9499	24,8953	24,9345	24,8342	25,0481	25,1289	25,0975	25,3162	25,1824	25,0708	+		4	4	4	12,1	12,1	12,1	32,976	6,67E+08	23	25,0458	24,9324	25,1591	10	5	5	0,00364597	0,226761	F9UR56	F9UR56	lp_2534	DegV family protein
26,3728	26,3537	26,5223	26,3606	26,4397	26,4431	26,6472	26,7081	26,74	26,6461	+		10	10	10	23,8	23,8	23,8	70,073	2,79E+09	104	26,5234	26,4098	26,6369	10	5	5	0,00574576	0,227063	F9UQT5	F9UQT5	lp_2394	ABC transporter, ATP-binding and permease protein
24,5775	24,6611	24,5337	24,4373	24,4934	24,6451	24,6975	24,7588	24,7939	24,9486	+		10	10	10	18,2	18,2	18,2	68,699	8,72E+08	40	24,6547	24,5406	24,7688	10	5	5	0,00742496	0,22819	Q88VE9	Q88VE9	uvrC	UvrABC system protein C
27,4313	27,5383	27,5948	27,7018	27,6229	27,7449	27,8129	27,8237	27,862	27,7954	+		9	9	9	52,5	52,5	52,5	27,786	5,92E+09	103	27,6928	27,5778	27,8078	10	5	5	0,00155988	0,229955	F9UTA8	F9UTA8	parA	Chomosome partitioning protein,membrane-associated ATPase
25,2584	25,0141	25,1128	24,8913	25,2459	25,2462	25,2836	25,488	25,4135	25,2462	+		8	8	8	16,7	16,7	16,7	72,602	1,17E+09	40	25,22	25,1045	25,3355	10	5	5	0,0266847	0,230982	F9USC9	F9USC9	lp_3000	ABC transporter, ATP-binding and permease protein
25,4881	25,5754	25,5955	25,6069	25,5626	25,8956	25,91	25,8066	25,6067	25,7717	+		5	5	5	51,7	51,7	51,7	16,798	1,36E+09	40	25,6819	25,5657	25,7981	10	5	5	0,00404885	0,232441	F9USV9	F9USV9	lp_0138	Nucleoside 2-deoxyribosyltransferase
27,1875	27,125	27,0901	27,0735	27,181	27,1501	27,3712	27,3202	27,6052	27,3735	+		4	4	4	29,2	29,2	29,2	12,284	2,39E+09	27	27,2477	27,1314	27,364	10	5	5	0,015951	0,23259	F9URD1	F9URD1	trxH	Thioredoxin H-type
24,5469	24,5581	24,7882	24,8073	24,7258	24,976	24,837	24,7955	25,0783	24,9055	+		6	6	6	26,6	26,6	26,6	35,773	6,32E+08	29	24,8019	24,6853	24,9185	10	5	5	0,0146781	0,233203	F9UUB1	F9UUB1	lp_3433	Endoglucanase E-like protein, SGNH hydrolase family
31,7586	31,9453	31,8957	31,8821	31,8145	32,0035	32,067	32,1021	32,1664	32,1251	+		20	20	20	89,2	89,2	89,2	17,261	2,11E+11	961	31,976	31,8592	32,0928	10	5	5	0,00060111	0,233563	Q88XW9	Q88XW9	rpsE	30S ribosomal protein S5
25,7817	25,8065	25,7573	25,7907	25,8503	25,843	25,9964	26,0672	26,295	25,9542	+		6	6	6	20,1	20,1	20,1	37,577	1,29E+09	44	25,9142	25,7973	26,0312	10	5	5	0,0160203	0,233856	F9UN53	F9UN53	oppC	Oligopeptide ABC transporter, permease protein
26,2142	26,4206	26,4105	26,4008	26,3773	26,3624	26,6207	26,7593	26,7176	26,5397	+		7	7	7	19,1	19,1	19,1	63,619	3,33E+09	83	26,4823	26,3647	26,5999	10	5	5	0,0190484	0,235243	F9UQT6	F9UQT6	lp_2395	ABC transporter, ATP-binding and permease protein
26,6471	26,7782	26,7533	26,8602	26,7558	26,7544	26,9849	27,1227	27,1756	26,9416	+		11	11	11	16,7	16,7	16,7	67,276	5,76E+09	117	26,8774	26,7589	26,9958	10	5	5	0,0197032	0,236929	F9ULW0	F9ULW0	lp_0729	Amino acid transport protein
25,0642	25,4862	25,1328	25,0922	25,1299	25,4218	25,31	25,3289	25,5155	25,5138	+		4	4	4	19,1	19,1	19,1	33,02	8,38E+08	26	25,2995	25,181	25,418	10	5	5	0,0285112	0,236933	F9URV1	F9URV1	lp_2849	ABC transporter, ATP-binding protein
26,7459	26,8908	26,5751	26,6971	26,738	27,0398	26,9426	26,9564	26,9933	26,9008	+		7	7	7	57,1	57,1	57,1	18,211	2,99E+09	65	26,848	26,7294	26,9666	10	5	5	0,0028016	0,237214	F9URG1	F9URG1	nrdI	Ribonucleotide reductase protein NrdI
26,2354	26,2229	26,192	26,1681	26,3166	26,5909	26,3507	26,4844	26,4114	26,4912	+		6	6	6	16,7	16,7	16,7	47,571	1,00E+09	26	26,3464	26,227	26,4657	10	5	5	0,00105881	0,238705	Q88TY1	Q88TY1	dsdA	Probable D-serine dehydratase
24,8612	24,8249	24,9839	24,9612	25,0844	25,2219	25,0625	25,285	25,1245	25,2234	+		10	10	10	22,3	22,3	22,3	50,69	1,01E+09	51	25,0633	24,9431	25,1834	10	5	5	0,00424957	0,240331	F9UT42	F9UT42	lp_0243	Uncharacterized protein
25,6942	25,845	25,9944	25,7879	25,9044	25,9311	26,1154	26,0897	26,2403	26,0561	+		19	19	19	30,2	30,2	30,2	77,232	2,82E+09	91	25,9658	25,8452	26,0865	10	5	5	0,00952272	0,241312	F9UQC9	F9UQC9	pbp2B	Transpeptidase, penicillin binding protein 2B
26,1345	25,8914	26,2174	26,1419	26,2115	26,2411	26,2263	26,6497	26,3432	26,3475	+		6	6	6	38	38	38	27,177	1,50E+09	67	26,2404	26,1193	26,3616	10	5	5	0,0367146	0,242224	F9ULX1	F9ULX1	rrp3	Two-component system response regulator,phosphate transport regulon
27,9181	27,9754	28,0211	28,0725	28,1349	28,2095	28,2492	28,2135	28,4006	28,2617	+		22	22	22	62,9	62,9	62,9	42,612	1,56E+10	187	28,1457	28,0244	28,2669	10	5	5	0,00148674	0,242492	F9UMU7	F9UMU7	lp_1146	ipoprotein, pheromone
25,6488	25,6917	25,7554	25,6752	25,5213	25,7084	25,8495	25,9645	26,0517	25,938	+		7	7	7	36,4	36,4	36,4	25,846	1,97E+09	69	25,7804	25,6585	25,9024	10	5	5	0,00816213	0,243898	F9UMA3	F9UMA3	pgm3	Phosphoglycerate mutase family protein
25,8648	25,9182	26,0122	25,8221	25,9059	26,0645	26,1184	26,2369	26,1659	26,1573	+		21	21	21	33,6	33,6	33,6	83,102	5,66E+09	151	26,0266	25,9046	26,1486	10	5	5	0,00044101	0,243988	F9UPA5	F9UPA5	pbp1A	DD-transpeptidase
24,953	25,287	25,4252	25,153	25,12	25,4409	25,2628	25,4843	25,5563	25,4162	+		7	7	7	34,2	34,2	34,2	29,04	9,06E+08	28	25,3099	25,1876	25,4321	10	5	5	0,0307152	0,244468	F9USU5	F9USU5	lp_0122	Alpha-beta hydrolase superfamily
25,4343	25,704	25,6883	25,483	25,3784	25,5982	25,81	25,9806	25,9213	25,6054	+		6	6	6	21,2	21,2	21,2	26,215	1,18E+09	42	25,6604	25,5376	25,7831	10	5	5	0,0450171	0,245482	F9ULY5	F9ULY5	lp_0758	Diguanylate cyclase/phosphodiesterase, EAL domain
30,2511	30,2031	30,5175	30,3761	30,4249	30,4672	30,4522	30,7679	30,6541	30,6598	+		8	8	8	40,5	40,5	40,5	25,747	1,87E+10	94	30,4774	30,3545	30,6002	10	5	5	0,0187261	0,245699	F9UP38	F9UP38	fabG1	3-oxoacyl-[acyl-carrier protein] reductase
25,6792	26,0078	25,9839	26,0121	25,9461	26,0845	26,1318	26,3345	26,104	26,2136	+		9	9	9	39,5	39,5	39,5	32,704	2,24E+09	67	26,0497	25,9258	26,1737	10	5	5	0,0128083	0,247865	Q88Z93	Q88Z93	rsmA	Ribosomal RNA small subunit methyltransferase A
25,3353	25,377	25,5398	25,3651	25,3713	25,6338	25,6757	25,4944	25,7005	25,7418	+		15	15	15	37	37	37	62,752	2,08E+09	63	25,5235	25,3977	25,6493	10	5	5	0,00199145	0,251543	F9ULT4	F9ULT4	dnaX	DNA polymerase III subunit gamma/tau
27,2546	27,2732	27,1223	27,294	27,2667	27,4755	27,5481	27,4763	27,5307	27,4387	+		8	8	8	42,6	42,6	42,6	21,791	5,26E+09	92	27,368	27,2422	27,4939	10	5	5	0,00012696	0,25171	Q88V20	Q88V20	lp_2267	dITP/XTP pyrophosphatase
31,1365	31,3396	31,1846	31,3637	31,1965	31,1867	31,4884	31,5262	31,6661	31,625	+		12	12	12	73,6	73,6	73,6	10,291	8,79E+10	489	31,3713	31,2442	31,4985	10	5	5	0,0288595	0,254296	Q88XY2	Q88XY2	rpsS	30S ribosomal protein S19
31,3102	31,383	31,5877	31,5389	31,5331	31,6702	31,6856	31,7612	31,7534	31,7558	+		8	8	8	73,5	73,5	73,5	11,048	4,33E+10	298	31,5979	31,4706	31,7252	10	5	5	0,00193175	0,254664	Q88WN5	Q88WN5	rplU	50S ribosomal protein L21
29,1014	29,1933	29,248	29,2608	29,2742	29,3975	29,4377	29,514	29,4466	29,5565	+		12	12	12	42,6	42,6	42,6	33,455	2,10E+10	160	29,343	29,2155	29,4705	10	5	5	0,00032977	0,254949	F9ULY7	F9ULY7	trxB	Thioredoxin reductase
29,1379	29,2703	29,3119	29,2521	29,1869	29,5287	29,4354	29,4552	29,4548	29,5679	+		3	3	3	12,9	12,9	12,9	33,308	1,13E+10	141	29,3601	29,2318	29,4884	10	5	5	0,00020894	0,256553	F9UP37	F9UP37	fabD	Malonyl CoA-acyl carrier protein transacylase
28,0887	28,3775	28,2632	28,3304	28,393	28,4407	28,7114	28,4843	28,6415	28,466	+		11	11	11	52,1	52,1	52,1	19,732	1,63E+10	142	28,4197	28,2905	28,5488	10	5	5	0,0100929	0,258221	F9UU95	F9UU95	lp_3416	Transcription regulator, MarR family
25,617	25,4828	25,6356	25,618	25,914	25,9406	25,7192	25,9799	25,9099	26,0223	+		6	6	6	30,9	30,9	30,9	34,204	1,37E+09	49	25,7839	25,6535	25,9144	10	5	5	0,0179535	0,260875	F9UQP6	F9UQP6	lp_2342	Transcription regulator, Xre family
25,6298	25,9796	26,0003	25,9222	25,9536	25,9451	26,0955	26,2714	26,3636	26,1217	+		9	9	9	39,7	39,7	39,7	34,173	1,60E+09	55	26,0283	25,8971	26,1595	10	5	5	0,0299689	0,262387	F9UU31	F9UU31	ppx1	Exopolyphosphatase
26,3072	26,5287	26,5589	26,3519	26,2796	26,5032	26,5589	26,9784	26,6755	26,6255	+		6	6	6	29,7	29,7	29,7	30,593	3,58E+09	64	26,5368	26,4053	26,6683	10	5	5	0,0315172	0,263023	F9UQ68	F9UQ68	dapA	4-hydroxy-tetrahydrodipicolinate synthase
26,5319	26,8664	26,8154	26,8767	26,7634	26,9923	27,183	26,9672	27,1055	26,9214	+		15	15	15	56,3	56,3	56,3	31,782	5,07E+09	147	26,9023	26,7708	27,0339	10	5	5	0,0105537	0,263085	F9UPI6	F9UPI6	lp_1854	Ribosome biogenesis GTPase A
30,7487	30,8756	30,8116	30,8208	30,8157	30,9352	31,096	31,0996	31,2009	31,057	+		19	19	19	74,4	74,4	74,4	17,841	1,15E+11	575	30,9461	30,8145	31,0777	10	5	5	0,00053438	0,263255	Q88XY9	Q88XY9	rpsG	30S ribosomal protein S7
28,2037	28,2578	28,3777	28,4143	28,4667	28,6116	28,5952	28,558	28,671	28,6024	+		20	20	20	42,1	42,1	42,1	40,651	1,98E+10	320	28,4758	28,344	28,6076	10	5	5	0,00101502	0,263589	Q88UZ4	Q88UZ4	recA	Protein RecA
25,0887	25,1256	25,0505	24,9908	25,0877	25,0943	25,3412	25,5542	25,3342	25,3405	+		5	5	5	19,1	19,1	19,1	36,557	9,18E+08	24	25,2008	25,0687	25,3329	10	5	5	0,00853729	0,264212	Q88Y75	Q88Y75	coaA	Pantothenate kinase
27,7018	28,0269	28,0748	28,1641	28,1477	28,0034	28,2562	28,3792	28,4393	28,3637	+		2	2	2	13,3	13,3	13,3	26,618	5,17E+09	45	28,1557	28,0231	28,2884	10	5	5	0,0484798	0,265324	F9UQS8	F9UQS8	gatD	ipid II isoglutaminyl synthase (glutamine-hydrolyzing) subunit GatD

30,9872	31,0567	30,8153	31,0363	31,1579	31,064	31,333	31,2969	31,332	31,355	+	8	8	8	60,9	60,9	60,9	10,077	4,63E+10	203	31,1434	31,0107	31,2762	10	5	5	0,00921219	0,265502	Q88WN3	Q88WN3	rpmA	50S ribosomal protein L27
26,0441	26,3149	26,1384	26,0328	26,1782	26,3802	26,3688	26,4583	26,5371	26,2987	+	13	13	13	40,7	40,7	40,7	39,728	3,46E+09	119	26,2752	26,1417	26,4086	10	5	5	0,00359964	0,266932	F9UN54	F9UN54	oppD	Oligopeptide ABC transporter, ATP-binding protein
25,8228	26,1189	25,9929	26,1328	25,8912	26,1542	26,2029	26,2201	26,4711	26,2569	+	12	12	12	41,6	41,6	41,6	33,307	3,29E+09	88	26,1264	25,9917	26,261	10	5	5	0,0112924	0,269308	F9UN38	F9UN38	hicD2	L-2-hydroxyisocaproate dehydrogenase
27,5578	27,9616	28,0138	27,889	27,8398	27,8907	28,2053	28,1032	28,2958	28,1166	+	7	7	7	27,1	27,1	27,1	31,769	7,32E+09	127	27,9874	27,8524	28,1223	10	5	5	0,032143	0,269901	F9UQH2	F9UQH2	rbsK2	Ribokinase
27,5671	27,5296	27,5522	27,3464	27,5633	27,5642	27,7896	27,8389	27,932	27,7866	+	11	11	11	33,1	33,1	33,1	32,333	5,31E+09	110	27,647	27,5117	27,7823	10	5	5	0,0062479	0,270535	Q88YF4	Q88YF4	murB	UDP-N-acetylenolpyruvoylglucosamine reductase
27,5659	27,8802	27,7703	27,8025	27,7945	27,8975	28,0289	28,1346	28,1179	27,9886	+	15	15	15	48	48	48	42,808	1,09E+10	189	27,8981	27,7627	28,0335	10	5	5	0,00411562	0,270827	F9UMH0	F9UMH0	thrA1	Aspartokinase
27,1036	26,9577	26,9703	26,939	26,9899	26,9662	27,2789	27,3036	27,5965	27,1735	+	3	3	3	17,2	17,2	17,2	23,001	1,74E+09	16	27,1279	26,9921	27,2638	10	5	5	0,0339274	0,271669	F9UQ20	F9UQ20	lp_2066	Uncharacterized protein
24,1642	24,2881	23,8765	23,821	23,7904	24,1974	24,2455	24,2689	24,4096	24,196	+	6	6	6	16,9	16,9	16,9	48,663	4,70E+08	24	24,1258	23,9881	24,2635	10	5	5	0,0335218	0,275428	F9UPX5	F9UPX5	dltD	Protein DltD
25,7936	25,4288	25,9454	25,9462	25,8509	26,145	26,0473	26,178	25,9585	26,0229	+	4	4	4	80,3	80,3	80,3	7,2568	6,50E+08	17	25,9317	25,793	26,0704	10	5	5	0,0281634	0,277401	P71478	P71478	csp	Cold shock protein 1
24,6404	25,0197	24,9586	24,9356	24,9923	24,9027	25,1608	25,406	25,2878	25,1815	+	10	10	10	30,8	30,8	30,8	47,235	1,44E+09	63	25,0485	24,9093	25,1878	10	5	5	0,032947	0,27846	Q88SV6	Q88SV6	purA	Adenylosuccinate synthetase
27,5758	27,8377	27,7717	27,7629	27,8779	27,8109	28,1028	28,0752	28,1742	28,0611	+	11	11	11	35,8	35,8	35,8	35,057	8,94E+09	170	27,905	27,7652	28,0448	10	5	5	0,00844021	0,279659	Q88VA5	Q88VA5	prs2	Putative ribose-phosphate pyrophosphokinase 2
25,0529	25,0943	25,2344	25,1806	25,4446	25,452	25,3487	25,6586	25,4842	25,4653	+	6	6	6	22,7	22,7	22,7	53,727	9,42E+08	43	25,3416	25,2014	25,4818	10	5	5	0,0108408	0,280412	Q88SY9	Q88SY9	lp_3226	Uncharacterized RNA methyltransferase lp_3226
25,2642	25,6616	25,4401	25,637	25,6473	25,6801	25,8359	25,7806	25,8944	25,8641	+	5	5	5	16,7	16,7	16,7	38,544	1,20E+09	35	25,6705	25,53	25,811	10	5	5	0,0117455	0,280991	F9UQ76	F9UQ76	lon	Endopeptidase La
30,9881	30,9573	30,9532	31,0369	30,9736	31,0526	31,2625	31,3282	31,3082	31,3677	+	5	5	5	85,2	85,2	85,2	9,1351	4,29E+10	141	31,1228	30,9818	31,2638	10	5	5	0,00118489	0,282003	Q88Z52	Q88Z52	rpmE2	50S ribosomal protein L31 type B
28,0558	28,212	28,2729	28,1677	28,2964	28,3707	28,5846	28,5395	28,5796	28,3454	+	27	27	27	64,1	64,1	64,1	41,443	1,46E+10	229	28,3424	28,2009	28,4839	10	5	5	0,00301228	0,283002	F9UPS7	F9UPS7	rpoD	RNA polymerase sigma factor SigA
27,157	27,5688	27,4213	27,4917	27,5732	27,6828	27,7423	27,5926	27,8233	27,8003	+	9	9	9	55,9	55,9	55,9	24,027	5,28E+09	96	27,5853	27,4424	27,7283	10	5	5	0,011255	0,285876	Q88WT5	Q88WT5	nadD	Probable nicotinate-nucleotide adenylyltransferase
24,8575	25,0033	25,0955	25,2422	25,2993	25,4762	25,4125	25,2403	25,4691	25,3377	+	2	2	2	20,1	20,1	20,1	18,205	5,26E+08	16	25,2434	25,0996	25,3872	10	5	5	0,0137346	0,287608	F9UTV5	F9UTV5	lp_0358	Phospholipid-binding protein
26,299	26,3016	26,0693	25,8586	26,2642	26,4129	26,5448	26,5074	26,4265	26,3398	+	7	7	7	56	56	56	13,808	1,19E+09	22	26,3024	26,1585	26,4463	10	5	5	0,0153171	0,28777	F9UN36	F9UN36	lp_1243	Transcription regulator, Cro/CI family
33,6008	33,7045	33,8073	33,7503	33,8766	33,9703	34,0781	34,0018	34,069	34,0596	+	39	39	39	83,2	83,2	83,2	36,438	1,53E+12	7879	33,8918	33,7479	34,0358	10	5	5	0,00049975	0,287883	F9UM10	F9UM10	gapB	Glyceraldehyde-3-phosphate dehydrogenase
26,7647	26,8191	26,6885	26,6589	26,9122	27,0757	27,045	27,0612	27,1566	26,9509	+	13	13	13	49,2	49,2	49,2	36,599	3,93E+09	95	26,9133	26,7687	27,0579	10	5	5	0,0008845	0,289214	F9UN55	F9UN55	oppF	Oligopeptide ABC transporter, ATP-binding protein
24,342	24,0383	24,2598	24,0899	24,0605	24,1547	24,4353	24,388	24,802	24,4573	+	4	4	4	21,1	21,1	21,1	32,948	3,84E+08	22	24,3028	24,1581	24,4474	10	5	5	0,0424104	0,289331	Q88Z48	Q88Z48	htpX	Protease HtpX homolog
30,4229	30,5937	30,5648	30,5153	30,4794	30,6946	30,8147	30,8672	30,9079	30,7433	+	12	12	12	74,2	74,2	74,2	11,152	7,46E+10	301	30,6604	30,5152	30,8056	10	5	5	0,00037514	0,290321	Q88XY4	Q88XY4	rplW	50S ribosomal protein L23
27,4595	27,5327	27,5174	27,428	27,517	27,6759	27,8226	27,7974	27,8467	27,7669	+	19	19	19	68,2	68,2	68,2	42,83	1,36E+10	281	27,6364	27,4909	27,7819	10	5	5	3,90E-05	0,290974	Q88V05	Q88V05	tgt	Queuine tRNA-ribosyltransferase
25,5816	25,8044	25,8941	25,7813	25,9765	25,7303	26,0281	26,1574	26,3676	26,2099	+	6	6	6	38,6	38,6	38,6	27,354	1,47E+09	61	25,9531	25,8076	26,0986	10	5	5	0,0494366	0,29106	F9UN91	F9UN91	rsuA	Pseudouridine synthase
30,8188	30,8254	31,0102	30,8231	30,7307	30,9265	31,0301	31,27	31,3384	31,1004	+	14	14	14	48,3	48,3	48,3	15,998	7,19E+10	403	30,9873	30,8416	31,1331	10	5	5	0,0110535	0,291422	Q88WG9	Q88WG9	fabZ	3-hydroxyacyl-[acyl-carrier-protein] dehydratase FabZ
25,1536	25,2496	25,1372	25,292	25,2678	25,5418	25,4392	25,5695	25,5683	25,4388	+	9	9	9	23,8	23,8	23,8	57,993	1,25E+09	38	25,3658	25,2201	25,5115	10	5	5	0,00014979	0,291451	F9UR50	F9UR50	lp_2525	ABC transporter, ATP-binding protein
29,5329	29,3609	29,3823	29,4511	29,4023	29,6731	29,7986	29,6729	29,7526	29,6926	+	16	16	16	59,8	59,8	59,8	31,514	4,26E+10	323	29,5719	29,4259	29,718	10	5	5	7,63E-05	0,292057	F9USG8	F9USG8	lp_3045	Short-chain dehydrogenase/oxidoreductase,classical SDR family, subgroup 1
27,5134	27,5794	27,5978	27,5681	27,6088	27,6241	27,7953	28,0019	28,0805	27,8286	+	22	22	22	56,2	56,2	56,2	43,758	9,49E+09	251	27,7198	27,5735	27,8661	10	5	5	0,00736681	0,292581	F9UR33	F9UR33	lp_2504	SAM-dependent methyltransferase
30,1641	30,3686	30,5195	30,4171	30,4021	30,5345	30,6367	30,6996	30,7624	30,7055	+	15	15	15	60,1	60,1	60,1	22,893	4,89E+10	263	30,521	30,3743	30,6677	10	5	5	0,00303051	0,293442	F9URH4	F9URH4	lp_2690	Short-chain dehydrogenase, atypical SDR family,subgroup 5
29,2594	29,2727	29,4265	29,3156	29,3746	29,5121	29,6555	29,685	29,6698	29,5935	+	19	19	19	45,8	45,8	45,8	39,101	3,00E+10	289	29,4765	29,3298	29,6232	10	5	5	0,0001769	0,293444	F9ULS9	F9ULS9	nrdF	Ribonucleoside-diphosphate reductase
31,8286	31,9526	31,9124	32,0261	32,0857	32,0855	32,3384	32,2195	32,3855	32,2487	+	9	9	9	64,8	64,8	64,8	9,3825	1,11E+11	819	32,1083	31,9611	32,2555	10	5	5	0,00261535	0,294424	F9UN59	F9UN59	ptsH	Phosphocarrier protein HPr
26,1472	26,4333	26,3864	26,4007	26,5037	26,4883	26,7305	26,7166	26,7979	26,6119	+	19	19	19	42,2	42,2	42,2	51,003	6,36E+09	172	26,5216	26,3743	26,669	10	5	5	0,00659324	0,294776	Q88XP4	Q88XP4	lp_1151	Uncharacterized RNA methyltransferase lp_1151
25,9771	26,076	26,0313	26,0384	25,9127	26,0277	26,2467	26,3243	26,6391	26,2745	+	7	7	7	24,6	24,6	24,6	45,46	1,26E+09	21	26,1548	26,0071	26,3024	10	5	5	0,0202847	0,295357	F9URM4	F9URM4	cblA1	Cystathionine beta-lyase
24,4271	24,253	24,6171	24,2459	24,4394	24,7471	24,851	24,8719	24,3884	24,6021	+	7	7	7	20,1	20,1	20,1	50,887	4,01E+08	19	24,5443	24,3965	24,6921	10	5	5	0,0309106	0,295613	F9UNS6	F9UNS6	rarA	Recombination factor protein RarA ATPase, AAA family
26,8924	26,9185	27,0549	26,9631	27,0102	27,0401	27,1424	27,2799	27,4227	27,4346	+	15	15	15	41,9	41,9	41,9	42,761	5,97E+09	117	27,1159	26,9678	27,2639	10	5	5	0,00720995	0,296104	F9UT85	F9UT85	xylR	Xylose operon regulator, ROK family
25,3201	25,7633	25,8143	25,677	25,8703	25,7866	25,9449	26,0664	26,2163	25,9142	+	9	9	9	18,3	18,3	18,3	62,002	1,68E+09	76	25,8373	25,689	25,9857	10	5	5	0,0407398	0,296698	F9UT60	F9UT60	treA	Trehalose-6-phosphate hydrolase
26,7537	27,0102	27,0225	27,016	26,8713	26,9111	27,2178	27,2852	27,4532	27,2934	+	13	13	13	44,6	44,6	44,6	37,699	6,42E+09	144	27,0834	26,9347	27,2322	10	5	5	0,0209784	0,297449	Q88UV2	Q88UV2	metN2	Methionine import ATP-binding protein MetN 2
26,6583	26,6068	26,6535	26,5875	26,7032	26,7572	26,9046	26,9888	27,0505	26,9973	+	20	20	20	45,5	45,5	45,5	53,234	9,37E+09	194	26,7908	26,6419	26,9397	10	5	5	0,00064753	0,297845	F9UPJ4	F9UPJ4	ctpA	Carboxy-terminal proteinase, S41 family,peptidoglycan-bound
29,0683	29,2565	29,3761	29,3992	29,0533	29,4339	29,4915	29,6648	29,6339	29,4291	+	10	10	10	16	16	16	68,564	1,71E+10	116	29,3807	29,2307	29,5306	10	5	5	0,00969291	0,299977	F9UQ44	F9UQ44	fruA	PTS system, fructose-specific EIIABC component
28,1976	28,4209	28,4227	28,5148	28,3806	28,4639	28,6994	28,7683	28,8072	28,6985	+	10	10	10	32,6	32,6	32,6	42,832	1,12E+10	144	28,5374	28,3873	28,6875	10	5	5	0,00534726	0,300154	F9UMU4	F9UMU4	purK2	Phosphoribosylaminoimidazole carboxylase,ATPase subunit
29,4018	29,6644	29,6959	29,5692	29,6993	29,662	29,893	30,0258	30,1045	29,8513	+	35	35	35	61,9	61,9	61,9	57,628	5,77E+10	718	29,7567	29,6061	29,9073	10	5	5	0,0130409	0,301199	Q88Z28	Q88Z28	lysS	ysine--tRNA ligase
26,8236	26,9884	26,9941	26,8329	27,0769	27,0879	27,1839	27,3694	27,3951	27,1912	+	27	27	27	39,6	39,6	39,6	91,701	8,51E+09	193	27,0943	26,9432	27,2455	10	5	5	0,00435155	0,302301	F9UPH5	F9UPH5	parC	DNA topoisomerase 4 subunit A
29,2643	29,3864	29,4084	29,3337	29,4613	29,6858	29,7039	29,6392	29,6143	29,7378	+	4	4	4	27,7	27,7	27,7	21,151	1,00E+10	128	29,5235	29,3708	29,6762	10	5	5	6,38E-05	0,305399	F9ULS8	F9ULS8	lp_0691	Cytokinin riboside 5-monophosphate phosphoribohydrolase
29,5066	29,3965	29,5384	29,5063	29,7944	29,8499	29,8242	29,8803	29,8829	29,8421	+	12	12	12	53	53	53	40,864	3,42E+10	274	29,7021	29,5484	29,8559	10	5	5	0,00178112	0,30744	F9UT53	F9UT53	cblB	Cystathionine beta-lyase / cystathionine gamma-lyase
27,219	27,3562	27,3857	27,2746	27,5637	27,4827	27,6568	27,7094	27,8096	27,6796	+	14	14	14	22,9	22,9	22,9	61,086	9,67E+09	182	27,5137	27,3598	27,6676	10	5	5	0,00466003	0,307776	F9UM05	F9UM05	lp_0783	Oligopeptide ABC transporter, substrate binding protein
27,8764	27,7855	27,857	27,7936	27,8624	28,0598	28,15	28,1135	28,3166	28,0741	+	17	17	17	45,7	45,7	45,7	44,373	1,17E+10	191	27,9889	27,835	28,1428	10	5	5	0,0002689	0,307817	F9UQ00	F9UQ00	nusA	Transcription termination/antitermination protein NusA
27,2759	27,4655	27,5452	27,43	27,3039	27,579	27,614	27,7167	27,8043	27,8491	+	8	8	8	65,2	65,2	65,2	18,186	4,05E+09	62	27,5584	27,4041	27,7126	10	5	5	0,00280771	0,30851	F9UQ74	F9UQ74	comEB	ComE operon protein 1, dCMP deaminase
28,322	28,3828	28,4066	28,3754	28,472	28,5252	28,7453	28,7637	28,7868	28,6812	+	23	23	23	41,1	41,1	41,1	60,333	1,77E+10	229	28,5461	28,3918	28,7004	10	5	5	0,00040022	0,30867	F9UN51;F9UT	F9UN51	oppA	Oligopeptide ABC transporter, substrate binding protein
28,1998	28,5446	28,4145	28,2628	28,3076	28,592	28,605	28,6098	28,7545	28,713	+	3	3	3	24,3	24,3	24,3	16,044	4,49E+09	60	28,5004	28,3458	28,6549	10	5	5	0,0020909	0,309028	F9USS0	F9USS0	lp_0091	Pyridoxamine 5-phosphate oxidase family protein, FMN-binding
26,9269	27,032	27,0876	26,9502	27,0942	27,1413	27,3337	27,3609	27,5531	27,2545	+	18	18	18	43	43	43	45,431	8,58E+09	176	27,1734	27,0182	27,3287	10	5	5	0,00352714	0,310538	Q88UX4	Q88UX4	thiI	Probable tRNA sulfurtransferase
31,4232	31,5105	31,5482	31,5049	31,4283	31,7368	31,8173	31,8559	31,8692	31,6923	+	17	17	17	63,6	63,6	63,6	22,697	1,13E+11	549	31,6387	31,483	31,7943	10	5	5	7,85E-05	0,31125	Q88XY6	Q88XY6	rplC	50S ribosomal protein L31 type B
27,8267	27,9454	27,9956	27,9276	27,9692	28,156	28,2597	28,2729	28,317	28,2206	+	20	20	20	62,3	62,3	62,3	42,041	1,99E+10	307	28,0891	27,9329	28,2452	10	5	5	4,87E-05	0,312342	Q88V96	Q88V96	mnmA	tRNA-specific 2-thiouridylase MnmA
28,8091	28,9261	28,9631	28,9129	28,9907	29,2184	29,1629	29,2639	29,3711	29,1639	+	7	7	7	89,4	89,4	89,4	7,3079	1,63E+10	114	29,0782	28,9204	29,236	10	5	5	0,00021641	0,31564	F9UMI2	F9UMI2	cspC	Cold shock protein CspC
24,9086	24,9277	24,9171	24,8476	24,8896	25,1413	25,145	25,1947	25,3433	25,2446	+	4	4	4	25	25	25	22,605	5,54E+08	27	25,0559	24,8981	25,2138	10	5	5	4,84E-05	0,31567	F9ULV2	F9ULV2	rimI1	Ribosomal-protein-alanine N-acetyltransferase
26,4646	26,6544	26,6948	26,5568	26,6364	26,6442	27,1637	26,9607	27,0037	26,8152	+	6	6	6	14,1	14,1	14,1	67,707	3,28E+09	75	26,7594	26,6014	26,9175	10	5	5	0,0116117	0,316049	F9UQE9	F9UQE9	pepF1	Oligoendopeptidase F
26,9283	26,9658	27,1954	27,2049	27,1431	27,1913	27,4402	27,4184	27,5059	27,4675	+	5	5	5	24,1	24,1	24,1	42,337	3,92E+09	92	27,2461	27,0875	27,4047	10	5	5	0,00431623	0,317178	F9UQB3	F9UQB3	iscS	Cysteine desulfurase
25,0832	25,1823	25,1544	25,1067	25,0716	25,3008	25,4082	25,4675	25,5805	25,432	+	6	6	6	33,3	33,3	33,3	22,146	7,40E+08	28	25,2787	25,1196	25,4378	10	5	5	0,00021577	0,318153	Q88UT1	Q88UT1	tdk	Thymidine kinase
28,6537	28,6304	28,6542	28,6229	28,6693	28,7699	28,9121	29,1231	29,0662	28,9549	+	13	13	13	56,7	56,7	56,7	38,987	1,64E+10	176	28,8057	28,6461	28,9652	10	5	5	0,00090591	0,319111	Q88V04	Q88V04	queA	S-adenosylmethionine:tRNA ribosyltransferase-isomerase
25,4081	25,4946	25,5977	25,4848	25,4946	25,7406	25,6537	25,9749	25,96	25,7541	+	8	8	8	42,3	42,3	42,3	24,693	1,76E+09	79	25,6563	25,496	25,8167	10	5	5	0,00191418	0,320712	F9UQM3	F9UQM3	minC	Septum site-determining protein MinC
29,328	29,453	29,4784	29,4853	29,2987	29,7781	29,9058	29,6247	29,7977	29,543	+	5	5	5	29,3	29,3	29,3	9,3492	8,41E+09	69	29,5693	29,4087	29,7299	10	5	5	0,00286703	0,321163	F9UP36	F9UP36	acpA2	Acyl carrier protein
26,9336	27,1214	27,141	26,9595	27,1066	27,1905	27,2738	27,4165	27,4594	27,5473	+	4	4	4	9	9	9	74,104	2,65E+09	82	27,215	27,0524	27,3775	10	5	5	0,00308901	0,325092	Q88UZ8	Q88UZ8	mutL	DNA mismatch repair protein MutL
25,3419	25,4611	25,6554	25,4137	25,6237	25,5603	25,6083	25,992	26,1209	25,8527	+	14	14	14	39,8	39,8	39,8	55,778	2,20E+09	56	25,663	25,4992	25,8268	10	5	5	0,0294242	0,327659	F9UTG6	F9UTG6	cls	Cardiolipin synthase
27,0129	27,0606	27,1348	27,0935	27,0384	27,1624	27,3806	27,5213	27,6101	27,3187	+	12	12	12	28,7	28,7	28,7	52,27	5,68E+09	143	27,2333	27,068	27,3986	10	5	5	0,00353719	0,330569	F9UM22	F9UM22	lp_0802	Polar amino acid ABC transporter, substrate binding and permease protein
25,0631	25,4426	25,3333	25,1263	25,4747	25,707	26,032	25,3402	25,5313	25,4999	+	4	4	4	13,1	13,1	13,1	34,37	5,92E+08	22	25,455	25,288	25,6221	10	5	5	0,0490546	0,334094	F9UKV7	F9UKV7	lp_0473	ipoprotein
29,0038	29,2105	29,2267	29,1813	29,2103	29,2299	29,4722	29,5545	29,6688	29,5803	+	15	15	15	38,6	38,6	38,6	43,101	3,20E+10	252	29,3338	29,1665	29,5011	10	5	5	0,00442845	0,334568	F9US66	F9US66	htrA	Serine protease HtrA
25,4942	25,7286	25,6607	25,7749	25,6111	25,903	26,2173	26,1474	25,9211	25,7648	+	6	6	6	31,2	31,2	31,2	37,155	9,14E+08	33	25,8223	25,6539	25,9907	10	5	5	0,00828458	0,336834	F9UU28	F9UU28	dus1	tRNA-dihydrouridine synthase
27,0381	27,4305	27,1829	27,3712	27,3241	27,2903	27,6017	27,6401	27,8353	27,6698	+	3	3	3	11,2	11,2	11,2	26,172	3,46E+09	40	27,4384	27,2694	27,6074	10	5	5	0,0176601	0,338071	F9ULV1	F9ULV1	gpp	Glycoprotein endopeptidase, M22 family
28,2047	28,3562	28,4355	28,489	28,4368	28,4898	28,6253	28,8122	28,9279	28,7865	+	16	16	16	44,8	44,8	44,8	46,971	1,75E+10	180	28,5564	28,3844	28,7283	10	5	5	0,00553291	0,343887	F9UT98	F9UT98	dacA1	Serine-type D-Ala-D-Ala carboxypeptidase
25,7861	25,972	26,0479	25,8695	26,1482	26,1973	26,3875	26,1888	26,4673	26,3048	+	5	5	5	55,1	55,1	55,1	19,893	1,70E+09	59	26,1369	25,9647	26,3091	10	5	5	0,00334464	0,344375	F9USA1	F9USA1	lp_2967	Transcription regulator, MarR family
28,1632	27,9777	27,9178	27,8531	27,9453	28,2258	28,395	28,3307	28,4369	28,1908	+	3	3	3	10,5	10,5	10,5	27,573	4,47E+09	77	28,1436	27,9714	28,3158	10	5	5	0,00121103	0,344405	F9UP44	F9UP44	accA2	Acetyl-CoA carboxytransferase
27,0568	27,2301	27,0547	27,1017	27,1681	27,491	27,5026	27,5307	27,4048	27,4247	+	6	6	6	38,2	38,2	38,2	26,242	3,56E+09	72	27,2965	27,1223	27,4708	10	5	5	3,09E-05	0,348495	F9USU0	F9USU0	lp_0117	Glutamine amidotransferase, class I
25,8041	26,2451	26,1683	26,2881	26,125	26,2843	26,4454	26,4536	26,6429	26,547	+	3	3	3	13,2	13,2	13,2	35,76	2,26E+09	45	26,3004	26,1261	26,4746	10	5	5	0,0101352	0,348518	F9UNW1	F9UNW1	lp_1584	Uncharacterized protein
29,5295	29,4613	29,5631	29,5486	29,6466	29,8662	29,9516	29,9207	29,8727	29,8835	+	7	7	7	15,3	15,3	15,3	74,393	1,44E+10	91	29,7244	29,5498	29,8989	10	5	5	6,86E-06	0,349139	F9UM68	F9UM68	lp_0856	Acyltransferase
27,4842	27,7925	27,8203	27,6636	27,6557	27,7208	27,8844	28,0817	28,3596	28,1181	+	5	5	5	18,3	18,3	18,3	46,327	3,99E+09	54	27,8581	27,6833	28,0329	10	5	5	0,0224611	0,349677	F9UTB1	F9UTB1	nupC	Pyrimidine nucleoside transport protein
26,154	26,344	26,4223	26,3345	26,4359	26,5507	26,6695	26,7669	26,8316	26,6216	+	11	11	11	47,8	47,8	47,8	28,125	2,98E+09	76	26,5131	26,3382	26,688	10	5	5	0,00116032	0,349891	F9UTB7	F9UTB7	lp_3211	Cystine ABC transporter, ATP-binding protein
28,5684	28,7603	28,732	28,7654	28,6275	28,8638	29,0269	29,1209	29,1686	29,0382	+	20	20	20	62	62	62	29,89	2,07E+10	231	28,8672	28,6907	29,0437	10	5	5	0,00064354	0,352965	F9UQQ2	F9UQQ2	lp_2350	ipoprotein
28,964	29,1108	29,2039	29,1001	29,2091	29,1786	29,5294	29,5805	29,6506	29,435	+	17	17	17	48,6	48,6	48,6	45,295	2,06E+10	232	29,2962	29,1176	29,4748	10	5	5	0,00503182	0,357219	F9UT87	F9UT87	cfa2	Cyclopropane-fatty-acyl-phospholipid synthase
26,924	27,1189	27,1561	27,0416	27,0745	27,1483	27,3636	27,3696	27,657	27,5627	+	8	8	8	24,3	24,3	24,3	34,64	2,97E+09	56	27,2416	27,063	27,4202	10	5	5	0,00615753	0,357245	F9UM38	F9UM38	lp_0819	Cell surface protein, YbbR-like family
24,6333	24,9019	24,8232	24,6381	25,0077	25,097	25,0593	25,1365	25,3814	25,1228	+	10	10	10	22,9	22,9	22,9	62,826	9,35E+08	48	24,9801	24,8008	25,1594	10	5	5	0,00484841	0,358578	F9UNY0	F9UNY0	recN	DNA repair protein RecN
27,2886	27,0728	27,1194	27,2176	27,2171	27,2604	27,5005	27,7047	27,6392	27,6208	+	4	4	4	9,5	9,5	9,5	47,185	2,54E+09	54	27,3641	27,1831	27,5451	10	5	5	0,00324414	0,36204	F9UMM5	F9UMM5	secY	Protein translocase subunit SecY
26,5192	26,7656	26,6733	26,7915	26,6897	26,8417	26,9979	27,1682	27,197	27,0578	+	16	16	16	40,6	40,6	40,6	39,593	4,44E+09	156	26,8702	26,6879	27,0525	10	5	5	0,00181864	0,364643	F9UN62	F9UN62	lp_1276	Glucosyl-diacylglycerol 6-beta-glucosyltransferase
27,0773	27,3064	27,2902	27,2305	27,304	27,4225	27,6242	27,6859	27,6854	27,6169	+	12	12	12	54,1	54,1	54,1	36,149	1,07E+10	156	27,4243	27,2417	27,607	10	5	5	0,00049638	0,365281	F9ULU4	F9ULU4	galE1	UDP-glucose 4-epimerase
27,4817	27,6228	27,6576	27,6476	27,5849	27,7395	27,9553	28,0492	28,1111	27,9794	+	8	8	8	30	30	30	30,162	7,17E+09	146	27,7829	27,5989	27,9669	10	5	5	0,00081557	0,36798	F9UPJ8	F9UPJ8	lp_1868	DegV family protein
26,0578	26,2641	26,2542	26,2404	26,1381	26,6449	26,5204	26,4789	26,6502	26,5132	+	4	4	4	15,7	15,7	15,7	36,405	1,22E+09	27	26,3762	26,1909	26,5615	10	5	5	0,00012667	0,370581	F9UU33	F9UU33	lp_0448	Uncharacterized protein
25,7067	25,9474	25,8218	25,869	26,0235	26,1195	26,3384	26,3105	26,2659	26,1984	+	5	5	5	25,6	25,6	25,6	37,369	1,27E+09	34	26,0601	25,8737	26,2465	10	5	5	0,0005324	0,37284	Q88V03	Q88V03	ruvB	Holliday junction ATP-dependent DNA helicase RuvB
29,2635	29,0312	28,9924	28,994	28,8827	29,2116	29,4819	29,3904	29,6396	29,3068	+	6	6	6	13,7	13,7	13,7	35,279	1,03E+10	63	29,2194	29,0328	29,4061	10	5	5	0,00481169	0,37332	Q88WG8	Q88WG8	fabH2	3-oxoacyl-[acyl-carrier-protein] synthase 3 protein 2
25,1088	25,4807	25,4448	25,3381	25,3613	25,217	25,7436	25,8538	25,9676	25,8258	+	7	7	7	23,8	23,8	23,8	39,736	9,63E+08	37	25,5342	25,3468	25,7216	10	5	5	0,0335943	0,3748	F9UQ72	F9UQ72	holA	DNA-directed DNA polymerase III, delta chain
28,1525	28,5969	28,6577	28,5414	28,4461	28,5887	28,9221	28,9335	29,0174	28,8069	+	6	6	6	41,9	41,9	41,9	19,178	1,28E+10	118	28,6663	28,4789	28,8537	10	5	5	0,0118789	0,374839	F9UN72	F9UN72	lp_1292	Acetyltransferase, GNAT family
25,5029	25,8566	25,6045	25,5613	25,4472	25,871	25,981	25,9441	26,1412	25,9304	+	5	5	5	17,2	17,2	17,2	31,527	1,23E+09	29	25,784	25,5945	25,9735	10	5	5	0,00198024	0,379037	F9ULX3	F9ULX3	pstE	Phosphate-binding protein
25,8866	25,9727	26,0193	25,9215	26,074	26,3757	26,4373	26,4222	26,3001	26,2744	+	10	10	10	33,2	33,2	33,2	46,685	2,64E+09	75	26,1684	25,9748	26,3619	10	5	5	3,33E-05	0,387093	Q88VG4	Q88VG4	obg	Obg
29,8366	29,7554	29,3968	29,5909	29,7115	29,6457	30,3003	30,0804	30,213	30,0074	+	7	7	7	48,4	48,4	48,4	7,5598	1,88E+10	84	29,8538	29,6582	30,0494	10	5	5	0,0209421	0,391151	Q88VR5	Q88VR5	rpsU	30S ribosomal protein S21
24,6458	25,1221	24,6808	24,8151	24,5252	24,8034	25,2937	25,39	25,0766	25,1845	+	2	2	2	35,7	35,7	35,7	11,22	5,26E+08	25	24,9537	24,7578	25,1496	10	5	5	0,0260536	0,391819	F9UL70	F9UL70	lp_0603	Acetyltransferase, GNAT family
26,0744	26,0326	26,1987	26,073	26,186	26,4513	26,6002	26,5278	26,5074	26,4375	+	6	6	6	15,6	15,6	15,6	66,772	1,96E+09	63	26,3089	26,1129	26,5048	10	5	5	2,10E-05	0,391905	F9US02	F9US02	lp_2914	Hypothetical membrane protein, DUF2207 family
25,8019	25,7621	25,7915	25,9602	25,8809	25,9649	26,0435	26,2538	26,5123	26,4223	+	3	3	3	12,1	12,1	12,1	25,237	9,20E+08	35	26,0393	25,8393	26,2394	10	5	5	0,00704737	0,400027	F9UTF5	F9UTF5	lp_3259	Zinc-dependent proteinase
25,6376	25,9072	26,0327	25,9409	25,7552	26,0615	26,26	26,2415	26,3753	26,3378	+	8	8	8	26,2	26,2	26,2	49,047	1,59E+09	43	26,055	25,8547	26,2552	10	5	5	0,0019817	0,400476	F9UTG2	F9UTG2	purB	Adenylosuccinate lyase
24,3797	24,1171	23,8833	24,0388	24,1809	24,2926	24,6517	24,4422	24,872	24,351	+	5	5	5	8,6	8,6	8,6	80,068	2,49E+08	24	24,3209	24,12	24,5219	10	5	5	0,0173375	0,401894	F9UL40	F9UL40	tex	Transcription accessory protein, contains S1 RNA binding domain
24,3545	24,3522	24,4452	24,5329	24,5961	24,7923	24,8956	24,6894	24,9934	24,9303	+	9	9	9	25,8	25,8	25,8	47,147	7,41E+08	37	24,6582	24,4562	24,8602	10	5	5	0,00051477	0,404017	Q88UU5	Q88UU5	murA1	UDP-N-acetylglucosamine 1-carboxyvinyltransferase 1
27,7713	27,9023	27,7862	27,8206	27,7503	27,8871	28,1671	28,2677	28,4325	28,2996	+	7	7	7	24,9	24,9	24,9	30,943	7,83E+09	108	28,0085	27,8062	28,2108	10	5	5	0,0027933	0,404633	Q88WG0	Q88WG0	accD2	Acetyl-coenzyme A carboxylase carboxyl transferase subunit beta 2
26,1268	26,139	26,2456	26,0627	26,3541	26,515	26,7265	26,6146	26,6143	26,4812	+	19	19	19	31,8	31,8	31,8	76,124	5,04E+09	196	26,388	26,1856	26,5903	10	5	5	0,00031214	0,404691	Q88YI8	Q88YI8	uvrB	UvrABC system protein B
27,2111	27,2383	27,2303	27,2113	27,2137	27,4331	27,6576	27,7749	27,6766	27,589	+	11	11	11	39,3	39,3	39,3	42,356	6,22E+09	127	27,4236	27,221	27,6262	10	5	5	0,00010072	0,405286	F9UQN3	F9UQN3	csd2	Cysteine desulfurase
27,5643	27,6193	27,4891	27,7061	27,6696	28,0054	28,0166	28,0211	28,0206	28,0137	+	12	12	8	45,7	45,7	35,2	28,968	8,20E+09	152	27,8126	27,6097	28,0155	10	5	5	5,79E-06	0,405775	F9UTB5	F9UTB5	lp_3209	Cystine ABC transporter, substrate binding protein
24,7931	24,6313	24,7193	24,4668	24,5834	24,8483	24,9184	25,3748	25,1823	24,902	+	3	3	3	22,4	22,4	22,4	24,606	5,05E+08	19	24,842	24,6388	25,0452	10	5	5	0,00778739	0,406374	Q88WZ9	Q88WZ9	trmB	tRNA (guanine-N(7)-)-methyltransferase
26,8987	27,0387	26,9266	26,9801	27,1853	27,1861	27,2867	27,5688	27,5822	27,4566	+	9	9	9	28,5	28,5	28,5	44,769	4,57E+09	107	27,211	27,0059	27,4161	10	5	5	0,00228949	0,410196	F9UP59	F9UP59	cfa1	Cyclopropane-fatty-acyl-phospholipid synthase
24,6819	24,6342	24,7395	24,5465	24,4385	24,9335	24,8297	25,1304	25,1961	25,0028	+	7	7	7	15,6	15,6	15,6	59,727	8,41E+08	43	24,8133	24,6081	25,0185	10	5	5	0,00127018	0,410401	F9US48	F9US48	lp_0018	Oligopeptide ABC transporter,oligopeptide-binding protein
25,0421	25,2337	25,3208	25,2762	25,2269	25,339	25,722	25,7808	25,7828	25,5285	+	5	5	5	12	12	12	67,218	8,34E+08	31	25,4253	25,2199	25,6306	10	5	5	0,00315493	0,410703	F9URC3	F9URC3	pepF2	Oligoendopeptidase F
24,3111	24,2945	24,6838	24,3799	23,7497	24,7208	24,4637	24,7784	24,6133	24,9036	+	11	11	11	11,4	11,4	11,4	141,03	8,60E+08	38	24,4899	24,2838	24,696	10	5	5	0,0400268	0,412162	Q88U41	Q88U41	addA	ATP-dependent helicase/nuclease subunit A
25,4447	25,4079	25,0816	25,2931	25,3761	25,5188	25,8308	25,7683	25,7534	25,8	+	4	4	4	21,5	21,5	21,5	18,072	9,83E+08	38	25,5275	25,3207	25,7343	10	5	5	0,00127564	0,41359	Q88VC8	Q88VC8	coaD	Phosphopantetheine adenylyltransferase
27,5336	27,5614	27,6161	27,6247	27,6125	27,8297	27,8741	28,04	28,1917	28,1013	+	13	13	13	49,8	49,8	49,8	28,242	8,09E+09	142	27,7985	27,5897	28,0074	10	5	5	0,00035405	0,417707	F9UMJ2	F9UMJ2	dacB	Serine-type D-Ala-D-Ala carboxypeptidase
23,8909	24,2772	24,0824	24,2572	24,2021	24,1469	24,4482	24,8693	24,651	24,6986	+	4	4	4	15	15	15	31,708	4,87E+08	32	24,3524	24,142	24,5628	10	5	5	0,0185384	0,420818	F9UR44	F9UR44	lp_2519	Cell surface hydrolase, DUF915 family,membrane-bound
25,6618	26,1247	25,9306	25,8512	26,2683	26,2135	26,5085	26,3603	26,4829	26,3833	+	8	8	8	24,1	24,1	24,1	58,336	2,52E+09	78	26,1785	25,9673	26,3897	10	5	5	0,007177	0,422418	F9URU7	F9URU7	tagE6	Poly(Glycerol-phosphate) alpha-glucosyltransferase
27,6943	27,9502	27,9373	27,8516	27,9703	28,1734	28,2761	28,3078	28,3694	28,3908	+	11	11	11	45,9	45,9	45,9	40,71	1,02E+10	150	28,0921	27,8807	28,3035	10	5	5	0,00016425	0,422765	O08445	O08445	alr	Alanine racemase
25,2529	25,3824	25,4849	25,5049	25,4713	25,6389	25,7901	25,8811	26,0834	25,867	+	3	3	3	37,9	37,9	37,9	11,296	9,20E+08	33	25,6357	25,4193	25,8521	10	5	5	0,00099687	0,432812	F9UQI9	F9UQI9	trxA2	Thioredoxin
24,6205	24,5731	24,5126	24,5071	24,5186	24,6387	24,8612	25,2531	25,197	24,9497	+	3	3	3	42,2	42,2	42,2	9,4656	3,89E+08	10	24,7632	24,5464	24,9799	10	5	5	0,00537372	0,433559	F9UU42	F9UU42	lp_0459	Uncharacterized protein
25,1535	24,8812	24,8886	25,232	25,2032	25,0602	25,3834	25,6364	25,6484	25,8345	+	2	2	2	37,7	37,7	37,7	7,9901	5,40E+08	6	25,2921	25,0717	25,5126	10	5	5	0,0214365	0,440887	Q6LWD8	Q6LWD8	orf39	Uncharacterized protein
25,8878	26,3654	26,246	26,1452	26,1885	26,4606	26,6183	26,7577	26,6894	26,5181	+	12	12	12	35,4	35,4	35,4	42,892	3,85E+09	94	26,3877	26,1666	26,6088	10	5	5	0,00171494	0,442276	F9UT17	F9UT17	ack1	Acetate kinase
26,5591	26,8242	26,7939	26,7095	26,8085	27,0633	27,2115	27,2258	27,2514	27,168	+	21	21	21	42,5	42,5	42,5	70,735	6,53E+09	154	26,9615	26,739	27,184	10	5	5	6,86E-05	0,444973	Q88RX6	Q88RX6	mnmG	tRNA uridine 5-carboxymethylaminomethyl modification enzyme MnmG
29,4599	29,5787	29,3973	29,4149	29,6134	29,8894	29,9836	30,0111	29,9723	29,8489	+	4	4	4	46,6	46,6	46,6	11,38	1,43E+10	76	29,7169	29,4929	29,941	10	5	5	3,09E-05	0,448167	Q88XX5	Q88XX5	rplX	50S ribosomal protein L24
24,5633	24,8932	24,8991	24,8467	25,0121	25,1805	25,1499	25,3392	25,4193	25,3673	+	5	5	5	11,2	11,2	11,2	55,81	5,89E+08	32	25,067	24,8429	25,2912	10	5	5	0,00123377	0,44837	F9UTW4	F9UTW4	choS	Glycine betaine/carnitine/choline ABC transporter, substrate binding and permease protein
25,5385	25,6182	25,4799	25,5862	25,4752	26,021	26,1392	25,9081	26,0365	25,8859	+	6	6	6	26,4	26,4	26,4	33,698	1,53E+09	47	25,7689	25,5396	25,9981	10	5	5	2,89E-05	0,458558	F9UPM1	F9UPM1	lp_1893	RNA-binding protein
24,5653	24,2838	24,5041	24,1847	24,4967	24,7461	24,8157	25,0385	24,8118	24,9288	+	5	5	5	25,3	25,3	25,3	35,022	5,31E+08	16	24,6376	24,4069	24,8682	10	5	5	0,00087773	0,461269	F9UR61	F9UR61	lp_2541	ABC transporter, substrate binding protein
29,6137	29,6558	29,6625	29,5111	29,5974	29,9735	30,0657	30,1738	30,094	30,0596	+	38	38	38	52,4	52,4	52,4	82,141	6,87E+10	700	29,8407	29,6081	30,0733	10	5	5	4,04E-06	0,46521	F9ULT0	F9ULT0	nrdE	Ribonucleoside-diphosphate reductase
25,0556	25,3288	25,2008	25,1793	25,4231	25,4631	25,7188	25,7244	25,9101	25,7152	+	9	9	9	22,4	22,4	22,4	48,692	1,80E+09	87	25,4719	25,2375	25,7063	10	5	5	0,00116895	0,468805	F9UTT1	F9UTT1	acdH	Acetaldehyde dehydrogenase
24,5131	24,7931	24,9379	25,0765	24,9436	25,1263	25,3159	25,1296	25,4284	25,6674	+	7	7	7	22,5	22,5	22,5	40,616	6,87E+08	24	25,0932	24,8528	25,3335	10	5	5	0,00878335	0,480695	F9UTR9	F9UTR9	potD	Spermidine/putrescine ABC transporter,substrate binding protein
24,2778	24,4364	24,6903	24,5954	24,251	24,8922	24,8189	24,9254	25,1805	24,8454	+	5	5	5	23,8	23,8	23,8	33,469	6,62E+08	39	24,6913	24,4502	24,9325	10	5	5	0,00206132	0,482289	F9UTB3	F9UTB3	lp_3206	Transcription regulator, LysR family
25,905	26,5403	26,5362	26,476	25,9909	26,3384	26,699	27,0644	27,0375	26,7341	+	3	3	3	7,7	7,7	7,7	44,53	1,04E+09	20	26,5322	26,2897	26,7747	10	5	5	0,0363188	0,485002	F9UQQ6	F9UQQ6	rodA2	Rod-shape determining protein
29,266	29,3772	29,5565	29,4468	29,1599	29,7367	29,9251	29,7993	30,0308	29,7575	+	2	2	2	18,1	18,1	18,1	13,77	1,16E+10	38	29,6056	29,3613	29,8499	10	5	5	0,00057041	0,488605	F9UQK0	F9UQK0	yajC	Preprotein translocase, YajC subunit
25,7664	25,9711	26,2146	26,1952	26,1243	26,3369	26,5585	26,6513	26,6168	26,6148	+	2	2	2	8,8	8,8	8,8	29,919	1,26E+09	38	26,305	26,0543	26,5557	10	5	5	0,00110958	0,50134	F9ULU3	F9ULU3	lp_0708	Acyl-[acyl-carrier protein] thioesterase
28,7012	28,7844	28,858	28,901	29,0153	29,1464	29,273	29,3843	29,5954	29,4249	+	8	8	8	26,3	26,3	26,3	32,962	1,33E+10	133	29,1084	28,852	29,3648	10	5	5	0,00053068	0,512798	F9UMN8	F9UMN8	lp_1070	ipoprotein, FMN-binding protein
24,7476	24,9335	25,1327	25,0223	24,5725	25,1763	25,3362	25,4105	25,6778	25,3808	+	3	3	3	12,3	12,3	12,3	33,683	5,37E+08	20	25,139	24,8817	25,3963	10	5	5	0,00393958	0,514615	F9UN52	F9UN52	oppB	Oligopeptide ABC transporter, permease protein
25,1565	25,3341	25,1389	24,8843	25,2872	25,4732	25,8979	25,6737	25,8332	25,5443	+	4	4	4	14,6	14,6	14,6	36,013	1,07E+09	26	25,4223	25,1602	25,6845	10	5	5	0,00166118	0,524255	Q88W05	Q88W05	thyA	Thymidylate synthase
25,3772	25,6406	25,2697	25,8181	25,032	25,477	25,858	25,8855	26,3361	26,2366	+	2	2	2	7,8	7,8	7,8	28,645	7,71E+08	21	25,6931	25,4275	25,9587	10	5	5	0,0327237	0,531149	F9USW1	F9USW1	lp_0141	Extracellular protein
26,6516	27,0933	27,2004	26,9563	26,5036	27,2611	27,5427	27,3291	27,4908	27,4466	+	7	7	7	63,7	63,7	63,7	14,558	2,05E+09	77	27,1475	26,881	27,414	10	5	5	0,00554366	0,533019	F9UQ60	F9UQ60	lp_2113	Fe-S_biosyn domain-containing protein
25,6658	25,815	25,6568	25,7393	25,9505	26,0639	26,4479	26,2579	26,5207	26,2342	+	5	5	5	21,6	21,6	21,6	27,707	1,56E+09	56	26,0352	25,7655	26,3049	10	5	5	0,00056433	0,539474	F9UM47	F9UM47	lp_0829	Nitroreductase family protein
26,6751	27,1292	26,9349	26,8567	26,4921	27,0792	27,2477	27,2982	27,6278	27,5777	+	3	3	3	3,9	3,9	3,9	102,19	2,04E+09	28	27,0919	26,8176	27,3661	10	5	5	0,00653821	0,548503	F9US57	F9US57	lp_0028	Kojibiose-like phosphorylase, specific
24,8819	24,7441	25,0699	25,0603	24,8996	25,4206	25,4558	25,5309	25,4075	25,5856	+	4	4	4	16,5	16,5	16,5	38,009	5,19E+08	19	25,2056	24,9312	25,4801	10	5	5	4,94E-05	0,548912	F9UT86	F9UT86	lp_3173	Cell surface protein, membrane-anchored

30,543	30,6081	30,5677	30,3857	30,5299	31,1738	31,1587	31,1434	30,9538	31,0099	+	4	4	4	14,6	14,6	14,6	36,812	5,27E+10	407	30,8074	30,5269	31,0879	10	5	5	1,13E-05	0,561027	F9UUA0	F9UUA0	lp_3421	Extracellular protein,gamma-D-glutamate-meso-diaminopimelate muropeptidase
27,7614	28,0758	27,6378	27,8754	27,9238	27,6532	28,2684	28,7516	28,7514	28,6905	+	4	4	4	59,7	59,7	59,7	8,2356	6,14E+09	106	28,1389	27,8548	28,423	10	5	5	0,035598	0,568181	Q88XW4	Q88XW4	infA	Translation initiation factor IF-1
25,6169	25,6339	25,6159	25,669	25,7078	25,9825	26,1532	26,2508	26,4753	26,2307	+	3	3	3	12,9	12,9	12,9	28,542	1,02E+09	29	25,9336	25,6487	26,2185	10	5	5	0,00011488	0,56981	F9URS4	F9URS4	lp_2812	Extracellular protein, membrane-anchored
25,0637	25,4833	25,4903	25,4795	25,2967	25,9175	25,9507	26,1819	25,8126	25,8037	+	3	3	3	14,9	14,9	14,9	22,229	7,53E+08	9	25,648	25,3627	25,9333	10	5	5	0,00072989	0,570575	F9UP60	F9UP60	lp_1697	Adherence protein, chitin-binding domain
26,7065	26,8124	26,8021	26,8555	26,7889	27,3198	27,4097	27,3788	27,444	27,2781	+	10	10	10	43,3	43,3	43,3	24,934	3,37E+09	90	27,0796	26,7931	27,3661	10	5	5	4,23E-07	0,572989	F9UNA0	F9UNA0	dgk2	Deoxynucleoside kinase
27,3539	27,4902	27,6393	27,5253	26,954	28,6418	27,6075	27,9984	27,4852	28,161	+	3	3	3	6,5	6,5	6,5	35,025	2,79E+09	31	27,6857	27,3925	27,9788	10	5	5	0,039372	0,586228	F9URU9	F9URU9	lp_2847	Extracellular transglycosylase, with LysM peptidoglycan binding domain
25,7413	26,0775	25,8731	25,8794	25,9169	26,2434	26,5862	26,6207	26,7322	26,4349	+	14	14	14	31,1	31,1	31,1	60,218	2,49E+09	57	26,2106	25,8977	26,5235	10	5	5	0,00024911	0,625837	F9US00	F9US00	lp_2912	ABC transporter, ATP-binding protein
24,5202	24,4814	24,3909	24,4383	24,1334	24,9453	24,8255	25,3721	25,22	24,8593	+	3	3	3	17,1	17,1	17,1	25,974	6,16E+08	16	24,7186	24,3928	25,0444	10	5	5	0,00090463	0,651612	F9UKZ1	F9UKZ1	srtA	Sortase A
29,7038	29,6	29,8884	29,6855	29,5571	30,2838	30,3461	30,3987	30,2642	30,4042	+	14	14	13	19,5	19,5	18,2	82,189	3,13E+10	188	30,0132	29,687	30,3394	10	5	5	7,31E-06	0,652417	F9URD9	F9URD9	acm2	Cell wall hydrolase/muramidase
25,8787	25,9973	26,0522	25,7685	25,4278	26,1285	26,404	26,7624	27,0051	26,1093	+	4	4	4	22,9	22,9	22,9	23,745	1,24E+09	26	26,1534	25,8249	26,4819	10	5	5	0,0135017	0,656971	F9UPJ6	F9UPJ6	lp_1866	Extracellular protein, DUF2140 family
26,0421	26,1019	26,1761	26,1624	26,1919	26,5505	26,8162	26,8423	26,882	26,8942	+	2	2	2	10,6	10,6	10,6	34,176	1,34E+09	27	26,466	26,1349	26,797	10	5	5	1,16E-05	0,662164	F9UU90	F9UU90	lp_3411	Extracellular protein, DUF1002 family
27,4379	27,3222	27,3882	27,1406	27,3141	27,6362	28,148	27,9087	28,2234	28,073	+	7	7	7	37	37	37	21,684	5,26E+09	60	27,6592	27,3206	27,9978	10	5	5	0,00038516	0,677258	Q890K1	Q890K1	ssb	Single-stranded DNA-binding protein
25,3235	25,6213	25,5312	25,4748	25,47	25,5157	26,0361	26,4741	26,3633	26,4281	+	5	5	5	76	76	76	8,3253	1,47E+09	44	25,8238	25,4842	26,1635	10	5	5	0,00641275	0,679293	F9UQQ7	F9UQQ7	lp_2357	Uncharacterized protein
25,1218	25,2607	25,3813	25,2805	25,4867	25,8038	26,0819	25,9941	26,183	25,8741	+	4	4	4	10,9	10,9	10,9	36,077	9,62E+08	42	25,6468	25,3062	25,9874	10	5	5	7,50E-05	0,681187	Q88UC0	Q88UC0	lp_2585	Ferredoxin--NADP reductase
24,3462	24,7577	24,7828	24,7273	25,0464	25,2838	25,5104	25,4447	25,5267	25,3556	+	8	8	8	19,4	19,4	19,4	51,036	7,97E+08	46	25,0782	24,7321	25,4242	10	5	5	0,00044855	0,692155	Q88RX5	Q88RX5	mnmE	tRNA modification GTPase MnmE
26,7456	27,1173	26,5985	27,1874	27,1252	27,4006	27,9383	27,5611	27,9259	27,4844	+	3	3	3	36,5	36,5	36,5	14,046	1,86E+09	33	27,3084	26,9548	27,662	10	5	5	0,00255127	0,707221	Q88X97	Q88X97	mscL	arge-conductance mechanosensitive channel
26,1055	26,0951	26,2377	26,187	26,1302	26,6039	26,8447	26,8888	26,9872	27,1738	+	5	5	5	14,1	14,1	14,1	66,11	2,25E+09	41	26,5254	26,1511	26,8997	10	5	5	5,62E-05	0,748561	P37063	P37063	pox5	Pyruvate oxidase
28,5761	28,2413	28,2496	28,1744	28,0521	27,9984	29,0182	29,2775	29,4675	29,2864	+	2	2	2	59	59	59	4,5596	4,77E+09	50	28,6341	28,2587	29,0096	10	5	5	0,0265119	0,750905	Q88XW3	Q88XW3	rpmJ	50S ribosomal protein L36
29,0101	29,2454	28,8181	28,984	29,0126	29,0922	29,667	30,146	30,1317	30,0295	+	3	3	3	43,3	43,3	43,3	10,208	1,16E+10	66	29,4137	29,0141	29,8133	10	5	5	0,00536972	0,799237	Q88WJ5	Q88WJ5	rpsP	30S ribosomal protein S16
25,795	25,6649	25,6882	25,5483	25,8247	26,5388	26,4273	26,5244	26,5406	26,5672	+	9	9	9	54	54	54	24,334	1,72E+09	39	26,1119	25,7042	26,5196	10	5	5	4,22E-07	0,815437	F9UMJ3	F9UMJ3	dgk1	Deoxyadenosine kinase / deoxyguanosine kinase
30,2119	30,371	29,9151	30,2923	30,0594	30,2736	30,922	31,2866	31,3894	31,1977	+	8	8	8	73,4	73,4	73,4	7,5377	2,96E+10	167	30,5919	30,1699	31,0139	10	5	5	0,00459013	0,843905	Q88XX8	Q88XX8	rpmC	50S ribosomal protein L29
25,461	25,2422	25,6193	25,319	25,5524	26,0182	26,2473	26,5752	26,4428	26,1343	+	5	5	5	21,7	21,7	21,7	34,32	9,82E+08	22	25,8612	25,4388	26,2836	10	5	5	0,00012988	0,84478	F9URZ6	F9URZ6	endA	DNA-entry nuclease
25,6912	25,8435	25,7092	25,828	25,7356	26,0616	26,6133	26,9694	26,958	26,7747	+	2	2	2	27,9	27,9	27,9	11,715	1,34E+09	27	26,2185	25,7615	26,6754	10	5	5	0,00065863	0,913871	F9UNR4	F9UNR4	lp_1529	RNA binding protein, YhbY family
26,6328	26,8088	26,7401	26,7344	26,6686	27,5046	27,6544	27,7231	27,5824	27,7182	+	20	20	20	30,9	30,9	30,9	83,73	1,20E+10	203	27,1767	26,7169	27,6365	10	5	5	1,03E-07	0,919603	F9US18	F9US18	nrdD	Anaerobic ribonucleoside-triphosphate reductase
26,0673	26,0879	26,1254	26,1849	26,2483	26,8491	27,1311	27,176	27,0743	27,1334	+	6	6	6	18,7	18,7	18,7	49,328	2,94E+09	69	26,6078	26,1428	27,0728	10	5	5	6,98E-07	0,930032	F9UUC2	F9UUC2	nox5	NADH oxidase
29,4363	29,6919	29,206	29,4308	29,5898	30,1095	30,6513	30,7087	30,7576	30,6504	+	3	3	3	39,3	39,3	39,3	9,0845	1,56E+10	59	30,0232	29,471	30,5755	10	5	5	5,94E-05	1,10453	Q88VD4	Q88VD4	rpsT	30S ribosomal protein S20
24,1262	23,9377	23,9199	23,5914	24,1862	24,7175	25,3275	25,4441	25,2171	24,8807	+	3	3	3	6,8	6,8	6,8	45,206	3,44E+08	12	24,5348	23,9523	25,1174	10	5	5	0,00014281	1,16512	F9UN64	F9UN64	dacA2	Serine-type D-Ala-D-Ala carboxypeptidase
27,8432	27,7845	27,7517	27,5975	27,6618	28,8503	29,0248	28,9619	28,9014	28,7774	+	5	3	3	26	14,2	14,2	21,336	4,99E+09	56	28,3154	27,7277	28,9031	10	5	5	5,72E-08	1,1754	F9USE1	F9USE1	lp_3014	Extracellular transglycosylase, with LysM peptidoglycan binding domain
25,9681	25,7244	25,847	25,8156	25,8731	26,7057	27,0742	27,1256	27,2007	27,1533	+	6	6	6	18,9	18,9	18,9	47,585	1,82E+09	69	26,4488	25,8457	27,0519	10	5	5	1,68E-06	1,20625	F9UN44	F9UN44	gshR2	Glutathione reductase
24,331	24,3735	24,548	24,519	24,9072	25,7118	26,1889	25,5625	25,7643	25,722	+	2	2	2	11,5	11,5	11,5	15,006	7,58E+08	26	25,1628	24,5357	25,7899	10	5	5	2,66E-05	1,25416	F9USH2	F9USH2	lp_3050	Extracellular transglycosylase, membrane-bound
24,4414	24,2005	23,648	24,0368	23,8353	24,3726	25,0911	25,8217	26,0079	25,7572	+	4	4	4	57,1	57,1	57,1	5,6565	6,86E+08	18	24,7213	24,0324	25,4101	10	5	5	0,00322341	1,37769	Q88YX4	Q88YX4	rpmG	50S ribosomal protein L33
25,3451	25,303	25,5539	25,481	25,6753	27,0621	27,057	26,7766	26,9776	26,4725	+	2	2	2	6,4	6,4	6,4	50,646	7,12E+08	19	26,1704	25,4717	26,8692	10	5	5	5,21E-06	1,39749	F9UMR4	F9UMR4	citP	Citrate transport protein
29,3054	29,6428	29,3355	29,4294	29,5329	31,2776	31,0946	30,875	30,806	30,6462	+	5	5	5	27,4	27,4	27,4	22,111	2,22E+10	113	30,1945	29,4492	30,9399	10	5	5	2,61E-06	1,49063	F9UTQ8	F9UTQ8	lp_0304	Extracellular transglycosylase
29,6254	29,7836	29,2743	29,4789	29,5935	30,676	31,1933	31,031	31,3035	31,033	+	3	3	3	40,6	40,6	40,6	7,4188	2,40E+10	82	30,2992	29,5511	31,0473	10	5	5	4,08E-06	1,49619	Q88WU7	Q88WU7	rpmI	50S ribosomal protein L35
30,1519	30,313	30,344	30,1938	30,428	31,9714	31,9809	31,9078	32,0695	31,9096	+	6	6	4	25,8	25,8	16,9	26,309	2,90E+10	89	31,127	30,2862	31,9679	10	5	5	2,29E-09	1,6817	F9UTQ7	F9UTQ7	lp_0302	Extracellular transglycosylase
25,359	25,506	25,6534	25,7531	25,517	25,1718	24,1381	25,4022	25,3236	25,5617		2	2	2	11,5	11,5	11,5	32,702	5,66E+08	15	25,3386	25,5577	25,1195	10	5	5	0,133087	-0,438252	F9URY5	F9URY5	lp_2889	Uncharacterized protein
31,5532	31,7982	31,3209	31,7291	31,8155	31,6849	31,4076	31,0831	30,7964	31,3586		8	8	8	31,5	31,5	31,5	14,595	7,00E+10	325	31,4547	31,6434	31,2661	10	5	5	0,0664559	-0,377251	Q88XU7	Q88XU7	rpsI	30S ribosomal protein S9
25,8263	26,114	25,8695	26,0546	26,0686	26,1774	25,7072	25,2039	25,2647	25,7552		5	5	5	43,2	43,2	43,2	20,446	1,18E+09	32	25,8042	25,9866	25,6217	10	5	5	0,0874591	-0,364933	F9UTV9	F9UTV9	accB3	Biotin carboxyl carrier protein of acetyl-CoA carboxylase
26,3217	26,2922	25,8932	25,9109	25,8159	25,9566	26,0188	25,4735	25,6586	25,4519		3	3	3	20,9	20,9	20,9	16,012	9,10E+08	23	25,8793	26,0468	25,7119	10	5	5	0,0697893	-0,334888	F9URR0	F9URR0	lp_2794	Flavodoxin
27,5079	27,4508	27,3566	27,5368	27,4992	27,5756	27,6069	26,6523	27,038	26,8274		4	4	4	15,3	15,3	15,3	18,044	3,66E+09	59	27,3051	27,4703	27,14	10	5	5	0,131691	-0,33023	F9UQH9	F9UQH9	lp_2260	Extracellular protein, DUF336 family
24,3376	24,698	24,7056	24,4995	24,2957	23,8933	24,3582	24,3253	24,3717	24,1645		8	8	8	13,2	13,2	13,2	86,601	6,00E+08	33	24,3649	24,5073	24,2226	10	5	5	0,0522538	-0,284634	F9USZ3	F9USZ3	mapB	Maltose phosphorylase
30,2903	30,5486	30,2799	30,4471	30,3203	30,4922	30,2363	29,798	30,0188	29,9228		18	18	18	82	82	82	12,592	5,70E+10	619	30,2354	30,3772	30,0936	10	5	5	0,0661796	-0,283636	Q88YW7	Q88YW7	rplL	50S ribosomal protein L7/L12
26,2891	26,2849	26,7124	26,7987	26,5066	26,1914	26,0101	26,2269	26,2905	26,6483		2	2	2	16,2	16,2	16,2	18,217	1,07E+09	30	26,3959	26,5184	26,2734	10	5	5	0,138271	-0,244913	F9URS9	F9URS9	lp_2820	Hypothetical membrane protein, DUF1440 family
27,2729	27,7224	27,2838	27,9316	27,6292	27,2642	27,3482	27,2978	27,1572	27,5916		5	5	5	30,8	30,8	30,8	13,054	4,59E+09	82	27,4499	27,568	27,3318	10	5	5	0,14657	-0,236165	F9UNZ8	F9UNZ8	lp_1625	General stress protein, Gls24 family
27,6475	27,7462	27,6221	27,6498	27,7622	27,7027	27,6605	27,3588	27,3864	27,2095		5	5	5	38,1	38,1	38,1	20,463	5,28E+09	68	27,5746	27,6856	27,4636	10	5	5	0,0540911	-0,222013	F9UMG4	F9UMG4	lp_0970	Acetyltransferase, GNAT family
27,7377	28,0131	27,9631	27,8604	27,8188	27,971	27,7854	27,5342	27,5822	27,4499		6	6	6	51,2	51,2	51,2	23,04	4,17E+09	59	27,7716	27,8786	27,6645	10	5	5	0,0795532	-0,214093	F9UNK1	F9UNK1	lp_1460	tRNA binding domain protein
24,9759	25,0914	25,0831	25,0011	25,4133	25,1857	24,7627	24,9342	24,6658	24,9574		4	4	4	11,8	11,8	11,8	42,02	4,62E+08	30	25,0071	25,1129	24,9012	10	5	5	0,112747	-0,211764	F9UTH2	F9UTH2	lp_3283	Methonine synthase (Cobalamine-independent),C-terminal domain
30,7096	30,8051	30,9152	30,9316	30,7441	30,9883	30,8628	30,3984	30,4863	30,4117		44	44	44	75,7	75,7	75,7	49,435	1,72E+11	1010	30,7253	30,8211	30,6295	10	5	5	0,182334	-0,191593	Q88VE1	Q88VE1	tig	Trigger factor
29,4343	29,4699	29,7285	29,6194	29,5806	29,5571	29,5815	29,2488	29,259	29,2626		14	14	14	60,3	60,3	60,3	26,43	3,35E+10	246	29,4742	29,5665	29,3818	10	5	5	0,0827155	-0,184742	Q88V33	Q88V33	lp_2253	Probable transcriptional regulatory protein lp_2253
26,0254	26,3587	26,3417	26,3136	26,2861	25,8186	26,0327	26,2847	26,1786	26,1032		15	15	15	40	40	40	49,943	3,45E+09	101	26,1743	26,2651	26,0835	10	5	5	0,105167	-0,181541	F9UQ90	F9UQ90	pdhD	Dihydrolipoyl dehydrogenase
29,0929	28,8409	28,4909	28,599	28,9241	28,8656	28,8108	28,542	28,3808	28,4521		5	5	5	22,2	22,2	22,2	17,119	6,83E+09	77	28,6999	28,7896	28,6103	10	5	5	0,25411	-0,17931	F9ULL0	F9ULL0	lp_3663	Nucleotide-binding protein, universal stress protein UspA family
27,383	27,4683	27,4731	27,332	27,4058	27,2239	27,0428	27,1775	27,2318	27,5266		6	6	6	26	26	26	24,82	2,97E+09	51	27,3265	27,4125	27,2405	10	5	5	0,0734078	-0,171958	Q6LWE4	Q6LWE4	traD	Conjugation protein
30,7351	30,7754	30,7565	30,681	30,8057	30,7867	30,826	30,3404	30,5258	30,4383		22	22	22	88,8	88,8	88,8	31,686	1,07E+11	715	30,6671	30,7508	30,5835	10	5	5	0,126431	-0,167307	F9UU82	F9UU82	lp_3403	2,5 diketo-D-gluconic acid-like reductase, NADP dependent (Promiscuous)
30,9766	30,9868	30,7247	31,1153	31,1539	30,9298	30,9673	30,6723	30,5965	30,9564		11	11	11	78,7	78,7	78,7	10,536	5,62E+10	455	30,908	30,9915	30,8245	10	5	5	0,163674	-0,166988	Q88VD5	Q88VD5	rpsO	30S ribosomal protein S15
24,9011	24,8362	25,0161	24,8376	24,8275	24,777	24,625	25,1014	24,9185	24,1691		11	11	11	15,1	15,1	15,1	100,11	9,09E+08	25	24,801	24,8837	24,7182	10	5	5	0,337254	-0,165525	F9UNU6	F9UNU6	lp_1567	Hypothetical membrane protein
27,8454	27,9672	27,8387	27,9879	28,0006	27,7192	27,5754	27,7169	27,8868	27,9152		8	8	8	47,4	47,4	47,4	16,375	6,95E+09	106	27,8453	27,9279	27,7627	10	5	5	0,0502023	-0,165254	F9UP64	F9UP64	lp_1701	Nucleotide-binding protein, universal stress protein UspA family
30,36	30,5585	30,5091	30,3302	30,4441	30,4972	30,3594	30,1386	30,3322	30,0567		11	11	11	51,7	51,7	51,7	16,672	3,39E+10	242	30,3586	30,4404	30,2768	10	5	5	0,107909	-0,163543	F9UTM5	F9UTM5	hsp3	Small heat shock protein
25,5021	25,9386	25,6526	25,8048	26,043	25,9477	25,7595	25,4061	25,4385	25,601		3	3	3	20,7	20,7	20,7	23,474	7,68E+08	21	25,7094	25,7882	25,6305	10	5	5	0,293456	-0,157686	Q88UW9	Q88UW9	lp_2332	UPF0637 protein lp_2332
24,0223	24,3183	23,903	24,2681	24,075	23,9703	24,0121	23,9623	23,9893	23,8954		6	6	6	30	30	30	31,318	4,58E+08	14	24,0416	24,1173	23,9659	10	5	5	0,0945625	-0,151424	F9UTJ6	F9UTJ6	pflA	Pyruvate formate-lyase-activating enzyme
27,2084	27,4642	27,4717	27,5298	27,609	27,3498	27,3339	27,309	27,2767	27,2744		7	7	7	27,2	27,2	27,2	41,267	2,70E+09	41	27,3827	27,4566	27,3088	10	5	5	0,0642459	-0,147837	F9UMX1	F9UMX1	lp_1173	UDP-N-acetylglucosamine 2-epimerase
27,1111	27,4386	27,053	27,4956	27,3775	27,1949	27,1504	27,0863	27,1912	27,1201		5	5	5	53,9	53,9	53,9	12,401	3,54E+09	59	27,2219	27,2952	27,1486	10	5	5	0,149161	-0,146588	Q88VH3	Q88VH3	lp_2083	UPF0145 protein lp_2083
26,5713	26,5973	26,5109	26,574	26,9402	26,0772	26,7216	26,4978	26,5727	26,5935		4	4	4	42	42	42	13,729	1,92E+09	14	26,5656	26,6387	26,4926	10	5	5	0,307168	-0,146189	F9UNT7	F9UNT7	lp_1557	Transcription regulator, MarR family
29,1396	29,1877	28,9685	29,0807	29,1238	29,1205	28,4047	29,049	29,1546	29,0515		4	4	4	33,3	33,3	33,3	9,1025	6,54E+09	45	29,0281	29,1	28,9561	10	5	5	0,347176	-0,143974	Q890K0	Q890K0	rpsR	30S ribosomal protein S18
26,2451	26,3486	25,9683	26,2452	25,8324	25,8381	25,9183	26,1427	26,2062	25,8165		3	3	3	39,6	39,6	39,6	11,566	8,19E+08	19	26,0561	26,1279	25,9844	10	5	5	0,287311	-0,143558	Q88XP8	Q88XP8	gatC	Aspartyl/glutamyl-tRNA(Asn/Gln) amidotransferase subunit C
26,5591	26,5789	26,3808	26,4172	26,5086	26,5185	26,3981	26,2166	26,2795	26,3422		4	4	4	25,9	25,9	25,9	16,082	1,70E+09	49	26,4199	26,4889	26,351	10	5	5	0,0656549	-0,137921	F9UPT6	F9UPT6	lp_1972	Uncharacterized protein
26,3557	26,2715	26,2939	26,1328	26,1639	26,3395	25,9003	26,2664	25,9369	26,0872		5	5	5	33,3	33,3	33,3	19,046	8,90E+08	26	26,1748	26,2436	26,106	10	5	5	0,19174	-0,137511	F9ULT3	F9ULT3	tadA	tRNA-specific adenosine deaminase
28,7104	28,9582	29,0105	28,9066	28,9192	28,8566	28,8942	28,7018	28,6927	28,698		13	13	13	57,5	57,5	57,5	34,422	2,29E+10	233	28,8348	28,901	28,7687	10	5	5	0,0849702	-0,13232	F9UM27	F9UM27	pta	Phosphate acetyltransferase
24,4934	24,5283	24,5183	24,3539	24,2449	24,4963	24,357	24,3477	24,2187	24,062		3	3	3	19,5	19,5	19,5	29,797	2,93E+08	15	24,362	24,4277	24,2963	10	5	5	0,190315	-0,131436	F9UNX8	F9UNX8	lp_1603	rRNA methylase
25,699	26,0061	25,9377	26,0389	25,9738	25,8604	25,6799	25,8207	25,8373	25,821		7	7	7	30,1	30,1	30,1	37,965	1,82E+09	39	25,8675	25,9311	25,8039	10	5	5	0,0993809	-0,127254	F9USY4	F9USY4	lp_0172	Transcription regulator, LacI family,maltose-related
25,2406	25,2928	25,4444	25,4604	25,7004	25,1316	25,2352	25,3813	25,2977	25,4594		4	4	4	18,8	18,8	18,8	35,593	1,08E+09	26	25,3644	25,4277	25,3011	10	5	5	0,233886	-0,126653	F9UMR5	F9UMR5	citR	Citrate lyase regulator, SorC family
25,54	25,8658	25,7488	25,6205	25,7249	25,347	25,4826	25,7477	25,6157	25,6905		3	3	3	14,4	14,4	14,4	23,016	7,01E+08	21	25,6384	25,7	25,5767	10	5	5	0,215159	-0,123283	Q88W28	Q88W28	plsY	Glycerol-3-phosphate acyltransferase
26,2444	26,4585	26,2233	26,4312	26,0886	26,0785	25,9795	26,1937	26,1515	26,4339		3	3	3	83,3	83,3	83,3	8,1972	1,10E+09	17	26,2283	26,2892	26,1674	10	5	5	0,269351	-0,121781	F9USB8	F9USB8	lp_2987	Uncharacterized protein
27,844	27,9915	27,9649	27,884	28,1542	27,9083	27,9152	27,7945	27,8882	27,7237		8	8	8	19,6	19,6	19,6	42,505	8,21E+09	107	27,9069	27,9677	27,846	10	5	5	0,0999479	-0,121766	F9UKZ0	F9UKZ0	lp_0513	Uncharacterized protein
29,5666	29,2275	29,2138	29,1698	29,3155	29,3037	29,2563	28,9882	29,1463	29,2075		13	13	13	48,5	48,5	48,5	18,723	2,93E+10	151	29,2395	29,2986	29,1804	10	5	5	0,223674	-0,118246	Q88UT9	Q88UT9	atpF	ATP synthase subunit b
28,5379	28,7161	28,6207	28,636	28,6427	28,7456	28,6908	28,322	28,5403	28,2663		16	16	16	45,3	45,3	45,3	37,613	1,52E+10	226	28,5718	28,6307	28,513	10	5	5	0,273042	-0,117676	F9UU20	F9UU20	trpS	Tryptophan--tRNA ligase
26,8491	26,9904	26,5178	26,7708	26,9231	26,3275	26,6283	26,8027	26,8545	26,8565		2	2	2	12,4	12,4	12,4	14,226	1,92E+09	47	26,7521	26,8102	26,6939	10	5	5	0,395814	-0,116364	F9UP65	F9UP65	lp_1702	Hypothetical membrane protein, DUF805 family
31,3971	31,3144	31,1588	31,4278	31,4421	31,2981	31,1643	31,1381	31,1095	31,4523		15	15	15	73,7	73,7	73,7	13,61	7,38E+10	694	31,2903	31,3481	31,2325	10	5	5	0,198434	-0,11559	Q88WJ1	Q88WJ1	rplS	50S ribosomal protein L19
26,4584	26,9048	26,887	26,8356	27,0314	26,6344	26,8346	26,5993	26,6968	26,7816		9	9	9	30,9	30,9	30,9	47,47	4,68E+09	107	26,7664	26,8235	26,7093	10	5	5	0,314457	-0,114112	F9UNK9	F9UNK9	sufD	ABC transporter, iron-sulfur cluster assembly protein SufD
25,2703	25,6537	25,7394	25,5949	25,4555	25,4002	25,2455	25,4627	25,5611	25,4761		5	5	5	23,3	23,3	23,3	26,057	9,21E+08	24	25,4859	25,5428	25,4291	10	5	5	0,278114	-0,11365	F9UU45	F9UU45	znuC	Zinc/iron ABC transporter, ATP-binding protein
26,1399	26,4185	26,1837	26,3347	26,3606	25,9337	26,0779	26,1989	26,3451	26,3235		3	3	3	17,9	17,9	17,9	30,701	1,25E+09	24	26,2317	26,2875	26,1758	10	5	5	0,268558	-0,111683	Q88Z08	Q88Z08	thrB	Homoserine kinase
30,3383	30,0037	29,7829	30,221	30,3048	29,099	29,7919	30,4482	29,9201	30,8368		3	3	3	65	65	65	6,6809	1,91E+10	77	30,0747	30,1301	30,0192	10	5	5	0,733474	-0,110934	Q88XW8	Q88XW8	rpmD	50S ribosomal protein L30
28,3498	28,5086	28,5968	28,5953	28,5692	28,4945	28,3201	28,3964	28,369	28,4853		8	8	8	34,3	34,3	34,3	36,578	1,22E+10	142	28,4685	28,5239	28,4131	10	5	5	0,0891525	-0,110855	Q88YK1	Q88YK1	gpsA	Glycerol-3-phosphate dehydrogenase [NAD(P)+]
28,8836	28,9948	29,0666	29,138	29,0557	28,8931	28,8438	28,8448	29,0425	28,9619		11	11	11	70,8	70,8	70,8	21,453	1,30E+10	147	28,9725	29,0278	28,9172	10	5	5	0,0889226	-0,110527	Q88XQ4	Q88XQ4	xpt	Xanthine phosphoribosyltransferase
28,3419	28,5928	28,605	28,5421	28,5148	28,424	28,4307	28,4387	28,3766	28,3746		12	12	12	51,4	51,4	51,4	35,949	1,58E+10	218	28,4641	28,5193	28,4089	10	5	5	0,0554405	-0,110372	F9UQS9	F9UQS9	pmi	Mannose-6-phosphate isomerase
26,6584	26,7816	26,5266	26,5939	26,2041	26,5776	26,765	26,0161	26,5929	26,2719		3	3	3	25,5	25,5	25,5	16,949	1,93E+09	35	26,4988	26,5529	26,4447	10	5	5	0,529831	-0,10822	F9UM28	F9UM28	lp_0809	t(6)A37 threonylcarbamoyladenosine biosynthesis protein TsaE
24,3337	24,4746	24,3189	24,4326	24,4322	24,1197	24,2322	24,2155	24,4404	24,4441		4	4	4	26,2	26,2	26,2	22,152	4,31E+08	22	24,3444	24,3984	24,2904	10	5	5	0,170521	-0,108015	F9ULT2	F9ULT2	rsmC	16S rRNA methyltransferase
25,1665	25,3005	25,2752	25,1553	25,1057	25,0931	25,0104	25,1165	25,0567	25,1937		6	6	6	30,6	30,6	30,6	26,566	1,17E+09	41	25,1474	25,2006	25,0941	10	5	5	0,0583082	-0,106551	F9USM3	F9USM3	lp_3110	Short-chain dehydrogenase/oxidoreductase
26,388	26,3605	26,5081	26,3367	26,5034	26,242	26,3433	26,4287	26,2168	26,3358		14	14	14	41,9	41,9	41,9	52,587	3,52E+09	95	26,3663	26,4194	26,3133	10	5	5	0,0785177	-0,106041	F9UNL2	F9UNL2	sufB	ABC transporter component, iron-sulfur cluster assembly protein SufB, iron regulated
26,7893	26,8847	27,0207	26,7829	26,8564	26,5628	26,5884	26,9833	26,7859	26,8838		7	7	7	36,7	36,7	36,7	29,944	2,72E+09	40	26,8138	26,8668	26,7608	10	5	5	0,285309	-0,105976	F9UKZ4	F9UKZ4	lp_0517	Uncharacterized protein
26,1889	26,4283	26,1531	26,1748	26,3083	26,2042	26,1476	26,1798	26,0631	26,1415		9	9	9	45,5	45,5	45,5	18,071	1,61E+09	45	26,199	26,2507	26,1472	10	5	5	0,108003	-0,103454	Q88YG7	Q88YG7	smpB	SsrA-binding protein
27,0613	27,152	26,9935	27,0209	27,1267	27,1086	27,1701	26,7703	26,891	26,8983		8	8	8	43,9	43,9	43,9	26,844	3,00E+09	42	27,0193	27,0709	26,9676	10	5	5	0,234214	-0,103235	F9UM23	F9UM23	glnQ1	Glutamine ABC transporter, ATP-binding protein
31,6551	31,8275	31,8122	31,8198	31,8646	31,8293	31,8328	31,527	31,7087	31,566		25	25	25	74,2	74,2	74,2	26,971	2,29E+11	1027	31,7443	31,7959	31,6928	10	5	5	0,199044	-0,103101	Q88YH4	Q88YH4	tpiA	Triosephosphate isomerase
26,3022	26,4785	26,5428	26,4279	26,2166	26,1962	26,0873	26,5044	26,2719	26,4035		12	12	12	20	20	20	86,621	2,23E+09	62	26,3431	26,3936	26,2927	10	5	5	0,317524	-0,100957	Q88V72	Q88V72	ftsK	DNA translocase FtsK
25,7685	25,795	25,8303	26,0514	25,8834	25,7945	25,8826	25,4306	25,7904	25,9362		4	4	4	14,6	14,6	14,6	35,857	8,15E+08	23	25,8163	25,8657	25,7669	10	5	5	0,359538	-0,0988663	F9UTW5	F9UTW5	choQ	Glycine betaine/carnitine/choline ABC transporter, ATP-binding protein
24,8539	24,6393	24,5261	24,9223	24,6752	24,2878	24,5337	24,824	24,5914	24,9081		4	4	4	61,2	61,2	61,2	11,251	5,49E+08	17	24,6762	24,7234	24,629	10	5	5	0,49451	-0,0943775	F9UQ61	F9UQ61	lp_2114	NTP pyrophosphohydrolase
26,4158	26,1661	26,3805	26,1801	26,3615	26,3575	26,0971	26,1102	26,1689	26,3013		2	2	2	11	11	11	25,722	1,09E+09	25	26,2539	26,3008	26,207	10	5	5	0,242395	-0,0937889	F9UQI8	F9UQI8	lp_2269	Uncharacterized protein
29,7531	30,0284	29,8399	29,9147	29,8606	29,7381	29,7203	29,7811	29,8525	29,8361		11	11	11	38	38	38	29,345	2,60E+10	266	29,8325	29,8793	29,7857	10	5	5	0,11156	-0,0936848	F9UT13	F9UT13	lp_0206	Oxidoreductase, aldo/keto reductase family
29,2838	29,4626	29,3785	29,3505	29,3801	29,1323	29,2983	29,3448	29,3368	29,2955		26	26	26	53,5	53,5	53,5	53,361	3,20E+10	383	29,3263	29,3711	29,2815	10	5	5	0,0999028	-0,089571	F9USD8	F9USD8	pbg6	6-phospho-beta-glucosidase
32,134	32,2371	32,1248	32,1931	32,2613	32,1674	32,1684	32,0718	32,0418	32,066		27	27	27	89,1	89,1	89,1	26,087	3,13E+11	1445	32,1466	32,1901	32,1031	10	5	5	0,0523079	-0,0870239	Q88T35	Q88T35	gpmA2	2,3-bisphosphoglycerate-dependent phosphoglycerate mutase 2
31,7651	31,6924	31,799	31,7122	31,8539	31,784	31,7866	31,5676	31,7	31,5533		45	45	45	85,3	85,3	85,3	47,159	3,37E+11	1395	31,7214	31,7645	31,6783	10	5	5	0,178579	-0,0862137	F9UPL0	F9UPL0	rpsA	30S ribosomal protein S1
29,3854	29,558	29,5188	29,467	29,4589	29,8811	29,8698	28,8858	29,2135	29,1177		15	15	15	68,4	68,4	68,4	20,593	2,90E+10	273	29,4356	29,4776	29,3936	10	5	5	0,693956	-0,0840195	Q88VJ7	Q88VJ7	frr	Ribosome-recycling factor
28,2878	28,3714	28,3751	28,3376	28,3301	28,1602	28,3612	28,2108	28,386	28,1702		20	20	20	50,6	50,6	50,6	48,136	2,64E+10	347	28,2991	28,3404	28,2577	10	5	5	0,141976	-0,0827385	Q88XZ7	Q88XZ7	serS2	Serine--tRNA ligase 2
27,2084	27,4433	27,1871	27,2544	26,9146	27,0598	27,0826	26,947	27,1898	27,3179		3	3	3	36,7	36,7	36,7	12,876	1,81E+09	28	27,1605	27,2016	27,1194	10	5	5	0,459047	-0,0821079	F9UQT3	F9UQT3	lp_2391	Uncharacterized protein
25,7107	25,3869	25,8558	25,4047	25,3748	25,61	25,5153	25,7502	24,9402	25,5088		2	2	2	15,8	15,8	15,8	20,089	7,99E+08	49	25,5057	25,5466	25,4649	10	5	5	0,644277	-0,081683	F9UT36	F9UT36	lp_0235	Uncharacterized protein
27,6094	27,7574	27,594	27,6132	27,6767	27,4901	27,572	27,6446	27,5871	27,5491		4	4	4	23,5	23,5	23,5	23,739	3,68E+09	71	27,6094	27,6501	27,5686	10	5	5	0,0722699	-0,0815849	F9UM86	F9UM86	glnM	Glutamine ABC transporter, permease protein
26,8482	26,7031	26,6061	26,5442	26,4651	26,3747	26,4948	26,5532	26,881	26,4629		3	3	3	2,6	2,6	2,6	69,489	1,16E+09	18	26,5933	26,6333	26,5533	10	5	5	0,484962	-0,0800179	F9UT61;F9UT	F9UT61;F9UTE0	pts4ABC	PTS system, trehalose-specific IIBC component
32,8672	32,9547	32,9474	32,9605	33,0224	32,925	32,9622	32,8255	32,8426	32,8151		50	50	50	97	97	97	37,181	6,79E+11	4629	32,9123	32,9505	32,8741	10	5	5	0,0816863	-0,0763855	Q88VJ2	Q88VJ2	ldhD	D-lactate dehydrogenase
24,9325	24,9083	24,8795	24,9114	25,2128	24,9836	25,1221	24,6152	24,8766	24,8684		2	2	2	7,1	7,1	7,1	30,856	4,01E+08	23	24,931	24,9689	24,8932	10	5	5	0,485789	-0,0756882	Q88XV2	Q88XV2	ecfA1	Energy-coupling factor transporter ATP-binding protein EcfA1
27,7639	28,1577	28,1281	28,0478	28,1129	27,8774	27,9145	27,9826	28,0281	28,033		18	18	18	58	58	58	43,848	1,59E+10	279	28,0046	28,0421	27,9671	10	5	5	0,36575	-0,0749863	F9UTR4	F9UTR4	ack2	Acetate kinase
28,126	28,4486	28,3121	28,3534	28,3523	28,2562	28,3109	28,2695	28,4078	27,982		3	3	3	16,5	16,5	16,5	24,505	2,88E+09	39	28,2819	28,3185	28,2453	10	5	5	0,432908	-0,073188	F9UQB4	F9UQB4	mtn	5-methylthioadenosine/S-adenosylhomocysteine nucleosidase
26,7556	26,7132	26,7291	26,6757	26,8147	26,9212	26,9968	26,3635	26,6235	26,4416		9	9	9	28,6	28,6	28,6	36,275	4,00E+09	93	26,7035	26,7377	26,6693	10	5	5	0,608541	-0,0683491	F9UQS3	F9UQS3	lp_2376	Threonylcarbamoyl-AMP synthase
26,8566	26,908	26,8312	26,9493	26,7912	26,6302	26,7839	26,7634	26,8689	26,9482		7	7	7	32,1	32,1	32,1	40,101	2,16E+09	51	26,8331	26,8673	26,7989	10	5	5	0,289892	-0,0683342	F9UMR6	F9UMR6	mae	Malic enzyme, NAD-dependent
28,2775	28,4503	28,4837	28,5201	28,5595	28,2866	28,317	28,3945	28,4432	28,5102		29	29	29	56,8	56,8	56,8	59,038	2,70E+10	438	28,4243	28,4582	28,3903	10	5	5	0,316412	-0,0679142	F9UKZ6	F9UKZ6	rhe1	DEAD-box ATP-dependent RNA helicase CshA
27,8886	27,9261	27,824	27,7824	28,0517	27,7198	27,87	27,9096	27,8061	27,8297		7	7	7	18,4	18,4	18,4	50,707	6,24E+09	82	27,8608	27,8946	27,827	10	5	5	0,266457	-0,0675232	F9UP42	F9UP42	accC2	Acetyl-CoA carboxylase, biotin carboxylase subunit
25,3106	25,5473	25,4201	25,2159	25,1785	25,1842	25,1678	25,5594	25,1996	25,2241		3	3	3	12	12	12	32,639	6,02E+08	28	25,3008	25,3345	25,267	10	5	5	0,519111	-0,0674591	F9UQC5	F9UQC5	divIB	Cell division protein DivIB
29,653	29,7373	29,8899	29,7749	29,7355	29,7224	29,7535	29,686	29,6659	29,6314		55	55	55	68,1	68,1	68,1	81,724	1,32E+11	1137	29,725	29,7581	29,6918	10	5	5	0,170459	-0,0662907	F9UN58	F9UN58	clpE	ATP-dependent Clp protease, ATP-binding subunit ClpE
28,1399	28,3055	28,4311	28,3503	28,4264	28,1471	28,2082	28,2791	28,3182	28,383		27	27	27	59,1	59,1	59,1	44,756	2,44E+10	387	28,2989	28,3306	28,2671	10	5	5	0,373073	-0,0635414	F9UNU0	F9UNU0	mltG	Endolytic murein transglycosylase
26,355	26,5866	26,7576	26,7357	26,7176	26,6064	26,5675	26,4254	26,6248	26,616		5	5	5	17,7	17,7	17,7	50,404	2,25E+09	59	26,5993	26,6305	26,568	10	5	5	0,47631	-0,0625103	F9USK6	F9USK6	gabD	Succinate-semialdehyde dehydrogenase (NAD(P)+)
27,9256	28,0676	28,1273	28,0709	28,0112	28,1213	28,1472	27,8879	27,9993	27,7535		26	26	26	53,2	53,2	53,2	46,518	2,06E+10	326	28,0112	28,0405	27,9819	10	5	5	0,489898	-0,058638	Q88VE2	Q88VE2	clpX	ATP-dependent Clp protease ATP-binding subunit ClpX
27,8755	28,0138	27,938	27,8361	27,9228	27,8726	27,904	28,0394	27,7694	27,7091		4	4	4	8	8	8	44,453	6,48E+09	112	27,8881	27,9172	27,8589	10	5	5	0,39245	-0,0583652	F9ULC7	F9ULC7	lp_3572	FAD-dependent pyridine nucleotide-disulphide oxidoreductase
26,4561	26,7009	26,6106	26,5723	26,5769	26,2659	26,5038	26,705	26,5929	26,5596		15	15	15	39,9	39,9	39,9	40,736	3,74E+09	112	26,5544	26,5834	26,5254	10	5	5	0,503601	-0,0579079	F9URN7	F9URN7	lp_2766	Uncharacterized protein
26,2637	26,6242	26,5929	26,4309	26,7243	26,6108	26,8695	26,4209	26,3497	26,0982		11	11	11	40,6	40,6	40,6	41,068	3,65E+09	83	26,4985	26,5272	26,4698	10	5	5	0,716731	-0,0573738	Q88UT2	Q88UT2	prfA	Peptide chain release factor 1
24,8112	25,255	25,2176	25,1565	25,3507	24,9366	25,1249	25,0383	25,2606	25,1474		3	3	3	14,1	14,1	14,1	32,929	6,35E+08	21	25,1299	25,1582	25,1016	10	5	5	0,611549	-0,0566097	F9UP90	F9UP90	mvaK1	Mevalonate kinase
30,2868	30,3513	30,3686	30,3539	30,3129	30,3068	30,4297	30,1571	30,2233	30,2873		17	17	17	49,2	49,2	49,2	40,55	4,58E+10	352	30,3078	30,3347	30,2808	10	5	5	0,294235	-0,0538635	Q88VM1	Q88VM1	dnaJ	Chaperone protein DnaJ
24,1026	24,3998	24,7353	24,6599	24,3257	23,9568	24,2228	24,4124	24,7657	24,6001		2	2	2	9,9	9,9	9,9	27,13	3,72E+08	12	24,4181	24,4446	24,3915	10	5	5	0,778353	-0,0531006	F9UKY5	F9UKY5	lp_0507	Uncharacterized protein
29,2785	29,5922	29,5674	29,5143	29,5517	29,4579	29,4571	29,4376	29,4606	29,4293		16	16	16	45,7	45,7	45,7	34,493	2,80E+10	222	29,4747	29,5008	29,4485	10	5	5	0,388107	-0,0523331	F9UM66	F9UM66	pepR1	Proline iminopeptidase
25,1029	25,2093	25,1698	25,2153	25,1621	25,2703	25,0843	25,0452	25,1901	25,0082		6	6	6	43,6	43,6	43,6	18,603	7,63E+08	37	25,1457	25,1719	25,1196	10	5	5	0,348217	-0,0522629	F9USX1	F9USX1	lp_0158	Uncharacterized protein
29,1554	29,2709	29,369	29,277	29,0392	29,2354	29,1294	29,2113	29,0441	29,2407		52	50	50	58,5	56,7	56,7	96,569	7,42E+10	717	29,1973	29,2223	29,1722	10	5	5	0,484343	-0,0501022	Q88VX7	Q88VX7	clpB	Chaperone protein ClpB
25,2693	25,1531	25,1084	25,2224	25,5464	25,1535	25,301	25,3084	24,9413	25,3486		3	3	3	22,6	22,6	22,6	24,255	5,35E+08	30	25,2352	25,2599	25,2106	10	5	5	0,658055	-0,0493397	F9UM87	F9UM87	glnP1	Glutamine ABC transporter, permease protein
26,187	26,4157	26,3037	26,2771	26,2715	26,2312	26,4006	26,1656	26,3273	26,095		4	4	4	20,6	20,6	20,6	18,13	1,11E+09	39	26,2675	26,291	26,2439	10	5	5	0,495964	-0,0470657	F9UTE6	F9UTE6	fur	Ferric uptake regulator
27,8485	28,0191	27,8759	27,668	27,6893	27,9193	27,7995	27,926	27,7239	27,5091		3	3	3	15,1	15,1	15,1	20,854	2,73E+09	42	27,7979	27,8201	27,7756	10	5	5	0,668551	-0,0445789	Q88VB2	Q88VB2	def	Peptide deformylase
26,7776	27,2024	26,9974	26,9067	27,2164	26,8164	27,1095	26,9242	27,0785	26,956		9	9	9	40,6	40,6	40,6	33,009	3,65E+09	84	26,9985	27,0201	26,9769	10	5	5	0,677999	-0,0431675	Q88VR2	Q88VR2	nfo	Probable endonuclease 4
23,899	23,8409	23,8125	23,9066	23,8835	23,8045	23,4741	23,9793	23,6886	24,1822		2	2	2	9,5	9,5	9,5	23,686	1,98E+08	15	23,8471	23,8685	23,8257	10	5	5	0,735892	-0,0427822	F9ULY8	F9ULY8	lp_0762	Hypothetical membrane protein, DUF421 family
28,3509	28,4465	28,6451	28,5515	28,5743	28,5843	28,5144	28,3848	28,4496	28,4257		18	18	18	43,7	43,7	43,7	60,784	2,46E+10	344	28,4927	28,5137	28,4718	10	5	5	0,521238	-0,0418964	F9UNZ9	F9UNZ9	lp_1626	Phosphatase, dihydroxyacetone kinase family
29,0854	29,1026	29,2495	29,1979	29,2316	29,137	29,1826	29,0863	29,1002	29,157		39	39	39	49,3	49,3	49,3	80,802	6,57E+10	595	29,153	29,1734	29,1326	10	5	5	0,314165	-0,040778	Q88Z31	Q88Z31	ftsH	ATP-dependent zinc metalloprotease FtsH

29,4337	29,6594	29,4061	29,5351	29,531	29,3263	29,4196	29,4905	29,5781	29,5492		5	5	5	47,8	47,8	47,8	15,019	1,11E+10	68	29,4929	29,5131	29,4727	10	5	5	0,545256	-0,0403145	F9UQI0	F9UQI0	lp_2261	Uncharacterized protein
25,2408	25,2162	25,4095	25,5199	25,2405	25,2351	25,0655	25,3293	25,3642	25,4321		4	4	4	15,6	15,6	15,6	25,625	8,41E+08	30	25,3053	25,3254	25,2852	10	5	5	0,657197	-0,0401447	Q88YY8	Q88YY8	gpmA1	2,3-bisphosphoglycerate-dependent phosphoglycerate mutase 1
27,0537	27,4725	27,1388	27,2385	27,3036	27,1082	27,3487	27,3765	27,1363	27,0378		2	2	2	18,6	18,6	18,6	17,615	1,64E+09	29	27,2215	27,2414	27,2015	10	5	5	0,696746	-0,0399101	Q6LWF0	Q6LWF0	traJ	Conjugation protein
27,0751	27,1387	27,2945	27,0877	27,3409	27,0354	27,1106	27,3116	27,1282	27,1605		18	18	18	70,1	70,1	70,1	34,805	5,30E+09	162	27,1683	27,1874	27,1492	10	5	5	0,60673	-0,0381287	F9UNY3	F9UNY3	opuC	Glycine betaine/carnitine/choline ABC transporter, substrate binding protein
28,0116	28,2509	28,1865	28,1517	28,2255	28,0739	28,0757	28,1921	28,1669	28,1371		12	12	12	54,8	54,8	54,8	36,709	1,21E+10	183	28,1472	28,1652	28,1291	10	5	5	0,47603	-0,0360779	F9UP30	F9UP30	adh1	Alcohol dehydrogenase, zinc-binding
29,7675	29,8001	29,8988	29,8341	29,8242	29,7499	29,774	29,8171	29,768	29,8377		61	61	59	67,4	67,4	65,6	92,655	1,01E+11	997	29,8071	29,8249	29,7893	10	5	5	0,227085	-0,0356133	F9UMJ6	F9UMJ6	clpC	ATP-dependent Clp protease, ATP-binding subunit ClpC
24,6951	25,3171	24,6517	25,1847	24,8665	24,6544	24,3578	25,0161	25,3411	25,1698		3	3	3	52,9	52,9	52,9	7,7428	6,75E+08	35	24,9254	24,943	24,9079	10	5	5	0,878056	-0,0351616	Q88WL6	Q88WL6	rpoZ	DNA-directed RNA polymerase subunit omega
28,6127	28,8082	28,9121	28,8322	28,8337	28,6567	28,7382	28,8533	28,7805	28,7952		23	23	23	55,7	55,7	55,7	48,75	3,39E+10	392	28,7823	28,7998	28,7648	10	5	5	0,573944	-0,0349899	Q88WZ5	Q88WZ5	murC	UDP-N-acetylmuramate--L-alanine ligase
26,1849	26,3227	26,3616	26,2786	26,2461	25,987	26,1023	26,3907	26,5148	26,2318		6	6	6	31	31	31	20,99	2,31E+09	44	26,2621	26,2788	26,2453	10	5	5	0,746396	-0,0334583	F9UQR0	F9UQR0	lp_2360	Ribosomal protein serine-acetylating enzyme
27,2649	27,3534	27,46	27,3102	26,9047	27,2982	27,0217	27,2233	27,2155	27,3709		18	18	18	40,4	40,4	40,4	53,586	9,39E+09	174	27,2423	27,2586	27,2259	10	5	5	0,775352	-0,0327091	F9UP05	F9UP05	ftsY	Signal recognition particle receptor FtsY
28,7981	28,9732	28,9317	28,8872	28,9018	28,9121	28,9037	28,8549	28,8483	28,8097		22	22	22	50,2	50,2	50,2	56,786	3,58E+10	353	28,8821	28,8984	28,8657	10	5	5	0,373608	-0,0326736	F9URG7	F9URG7	gpd	Glucose-6-phosphate 1-dehydrogenase
27,9567	28,1483	28,1606	28,1342	28,1895	28,1516	27,9979	28,0445	28,0174	28,2146		7	7	7	68,6	68,6	68,6	16,918	8,30E+09	128	28,1015	28,1179	28,0852	10	5	5	0,59374	-0,0326588	F9URE5	F9URE5	lp_2652	Universal stress protein
31,7686	31,6882	31,6503	31,8813	31,8106	31,647	31,6604	31,7711	31,7527	31,8058		11	11	11	100	100	100	10,293	9,79E+10	441	31,7436	31,7598	31,7274	10	5	5	0,550727	-0,0324211	Q88YM6	Q88YM6	groS	10 kDa chaperonin
32,3087	32,4275	32,4807	32,4315	32,47	32,3733	32,4131	32,4042	32,353	32,4127		65	65	65	89,4	89,4	89,4	62,866	9,09E+11	3783	32,4075	32,4237	32,3913	10	5	5	0,352984	-0,0323868	F9UPM3	F9UPM3	pyk	Pyruvate kinase
29,3099	29,3912	29,3897	29,4473	29,4383	29,4131	29,3747	29,3234	29,3342	29,3789		9	9	9	54	54	54	17,053	1,00E+10	146	29,3801	29,3953	29,3648	10	5	5	0,329635	-0,0304176	F9UNJ5	F9UNJ5	lp_1454	Nucleotide-binding protein, histidine triad family
26,1787	26,3964	26,2062	26,3047	26,3167	26,0629	26,1613	26,3454	26,398	26,2885		4	4	4	19,2	19,2	19,2	29,169	2,21E+09	47	26,2659	26,2805	26,2512	10	5	5	0,698617	-0,0293007	F9UNC1	F9UNC1	pltR	Two-component system response regulator
28,959	29,0059	29,0829	29,0157	29,1091	28,9006	29,0547	29,0287	29,0581	28,9888		22	22	22	54,5	54,5	54,5	48,324	3,58E+10	449	29,0203	29,0345	29,0062	10	5	5	0,497409	-0,0283405	F9UQC4	F9UQC4	ftsA	Cell division protein FtsA
26,9827	26,9527	26,7157	26,7794	26,5649	26,764	26,7915	26,773	26,6664	26,8589		3	3	3	3,8	3,8	3,8	118	9,99E+08	13	26,7849	26,7991	26,7708	10	5	5	0,742352	-0,0283363	F9UNT0	F9UNT0	sbcC	Exonuclease SbcC
30,3442	30,4861	30,6889	30,5622	30,6385	30,4828	30,5603	30,5187	30,4545	30,5707		14	14	14	70,3	70,3	70,3	20,366	5,41E+10	354	30,5307	30,544	30,5174	10	5	5	0,691477	-0,0265667	Q88WN1	Q88WN1	efp	Elongation factor P
25,7545	25,6474	25,8069	25,838	25,7892	25,8126	25,5892	25,673	25,7933	25,8368		4	4	4	22,9	22,9	22,9	18,18	7,93E+08	47	25,7541	25,7672	25,741	10	5	5	0,66091	-0,0262218	F9UTL6	F9UTL6	pgpA	Phosphatidylglycerophosphatase
25,9523	25,5147	25,6174	25,8893	25,3956	26,0031	25,8279	25,4258	25,6668	25,3187		3	3	3	16,3	16,3	16,3	23,242	6,83E+08	18	25,6612	25,6739	25,6485	10	5	5	0,881562	-0,0254276	F9UM81	F9UM81	gph1	Phosphohydrolase
28,1047	28,2568	28,0802	28,2025	28,1293	28,0687	28,217	28,1357	28,1015	28,1237		5	5	5	65,4	65,4	65,4	12,065	3,32E+09	43	28,142	28,1547	28,1293	10	5	5	0,552664	-0,0254063	F9UTM1	F9UTM1	lp_3348	Uncharacterized protein
28,4608	28,7633	28,6961	28,5813	28,5322	28,5217	28,5494	28,6374	28,7049	28,4981		7	7	7	50,6	50,6	50,6	17,128	7,88E+09	116	28,5945	28,6067	28,5823	10	5	5	0,725399	-0,0243988	F9URL9	F9URL9	lp_2745	Nucleotide-binding protein, universal stress protein UspA family
26,5205	26,6521	26,6788	26,5497	26,7032	26,5645	26,6085	26,5593	26,6039	26,6476		9	9	9	29	29	29	34,743	2,07E+09	80	26,6088	26,6209	26,5967	10	5	5	0,559979	-0,0241219	F9UM56	F9UM56	ppx2	Exopolyphosphatase
28,2892	28,3025	28,3071	28,2756	28,3466	28,2023	28,2803	28,3497	28,2868	28,2833		29	29	29	46,6	46,6	46,6	72,125	2,21E+10	348	28,2923	28,3042	28,2805	10	5	5	0,393209	-0,0237141	F9US37	F9US37	gyrB	DNA gyrase subunit B
25,3862	25,577	25,1438	25,0802	25,223	24,9932	25,0801	25,4318	25,4491	25,3391		6	6	6	19,3	19,3	19,3	45,645	1,09E+09	19	25,2703	25,2821	25,2586	10	5	5	0,861139	-0,0234222	F9UTV6	F9UTV6	lp_0359	AAA domain-containing protein
26,0486	26,2195	26,1565	26,2266	26,346	26,0924	26,1538	26,1819	26,202	26,2554		9	9	9	31,4	31,4	31,4	38,908	2,17E+09	63	26,1883	26,1995	26,1771	10	5	5	0,697994	-0,0223434	Q88VL8	Q88VL8	hrcA	Heat-inducible transcription repressor HrcA
32,7133	32,8059	32,826	32,8313	32,9547	32,8906	32,8386	32,7521	32,7636	32,7749		37	37	37	73,3	73,3	73,3	48,029	7,46E+11	4066	32,8151	32,8262	32,8039	10	5	5	0,645567	-0,0223007	Q88YH3	Q88YH3	eno1	Enolase 1
27,0465	27,1089	27,184	27,0756	26,933	27,3138	27,0947	27,0026	27,036	26,8009		4	4	4	24	24	24	26,947	1,77E+09	39	27,0596	27,0696	27,0496	10	5	5	0,833783	-0,0199829	F9UQ57	F9UQ57	glnQ3	Glutamine ABC transporter, ATP-binding protein
29,3009	29,2633	29,1876	29,3105	29,2081	29,256	29,3066	29,2286	29,2336	29,1466		8	8	8	60,5	60,5	60,5	16,286	1,36E+10	158	29,2442	29,2541	29,2343	10	5	5	0,593499	-0,0198132	F9URX6	F9URX6	lp_2877	Universal stress protein
29,6592	29,8403	29,7552	29,8364	29,798	29,7555	29,7544	29,9333	29,7769	29,5704		5	5	5	29,8	29,8	29,8	20,392	2,12E+10	100	29,768	29,7778	29,7581	10	5	5	0,774344	-0,0197403	F9UMC6	F9UMC6	lp_0927	Hypothetical membrane protein
25,4219	25,6726	25,8433	25,6598	25,7847	25,6652	25,611	25,6328	25,73	25,645		4	4	4	30,2	30,2	30,2	26,767	1,00E+09	36	25,6666	25,6765	25,6568	10	5	5	0,800485	-0,019632	F9UNJ6	F9UNJ6	lp_1455	ABC transporter, ATP-binding protein
24,1641	24,0818	24,0671	24,4003	24,381	24,058	24,2282	24,0189	24,2726	24,4187		3	3	3	13,9	13,9	13,9	33,991	2,61E+08	13	24,2091	24,2189	24,1993	10	5	5	0,853654	-0,0195602	F9UPM0	F9UPM0	xerD	Tyrosine recombinase XerD
25,2933	25,3582	25,701	25,3041	25,498	25,3013	25,3699	25,3924	25,5106	25,4871		7	7	7	24,5	24,5	24,5	48,26	1,49E+09	59	25,4216	25,4309	25,4123	10	5	5	0,833241	-0,0186802	Q88W23	Q88W23	trmFO	Methylenetetrahydrofolate--tRNA-(uracil-5-)-methyltransferase TrmF
27,7106	27,5362	27,7239	27,5712	27,7275	27,7382	27,7279	27,6299	27,5397	27,5793		9	9	9	57,8	57,8	57,8	20,318	5,45E+09	127	27,6485	27,6539	27,643	10	5	5	0,853348	-0,0109161	F9UL19	F9UL19	hprT	Hypoxanthine phosphoribosyltransferase
32,3742	32,5811	32,5635	32,5374	32,6287	32,5409	32,5764	32,5129	32,5301	32,4733		54	54	54	92,2	92,2	92,2	42,796	8,01E+11	3531	32,5319	32,537	32,5267	10	5	5	0,831113	-0,0102516	Q88YH5	Q88YH5	pgk	Phosphoglycerate kinase
24,4095	24,571	24,971	25,1724	25,122	24,917	24,4371	24,9971	24,7252	25,1183		4	4	4	11,1	11,1	11,1	50,748	6,72E+08	21	24,8441	24,8492	24,839	10	5	5	0,959055	-0,0102436	F9US92	F9US92	tanL	Tannase (Tannin acylhydrolase)
28,0739	27,9216	27,6638	27,7732	27,8102	28,291	27,8709	27,5977	27,7332	27,7011		4	4	4	34,5	34,5	34,5	15,697	4,50E+09	82	27,8437	27,8485	27,8388	10	5	5	0,946111	-0,00975304	Q88UU4	Q88UU4	atpC	ATP synthase epsilon chain
28,4722	28,6484	28,6192	28,5368	28,6505	28,5279	28,4949	28,6713	28,5999	28,5889		29	29	29	47,6	47,6	47,6	92,627	2,46E+10	323	28,581	28,5854	28,5766	10	5	5	0,854052	-0,00883331	Q88XA9	Q88XA9	leuS	Leucine--tRNA ligase
27,7308	27,9723	27,9209	27,8906	28,025	27,7826	27,9217	27,8678	27,9897	27,9354		15	15	15	58,4	58,4	58,4	34,871	8,98E+09	173	27,9037	27,9079	27,8994	10	5	5	0,892778	-0,00847931	Q88YK3	Q88YK3	hprK	HPr kinase/phosphorylase
28,225	28,3567	28,4186	28,3335	28,1483	28,2356	28,233	28,3569	28,2837	28,3348		35	35	35	46	46	46	88,118	2,92E+10	531	28,2926	28,2964	28,2888	10	5	5	0,892819	-0,00759888	Q88WR2	Q88WR2	pheT	Phenylalanine--tRNA ligase beta subunit
26,5061	26,0926	26,211	26,5576	26,3118	26,5904	26,2622	26,179	26,4231	26,1872		3	3	3	52,8	52,8	52,8	8,7184	1,24E+09	27	26,3321	26,3358	26,3284	10	5	5	0,951205	-0,00744019	Q88VB0	Q88VB0	rpoY	DNA-directed RNA polymerase subunit epsilon
26,2105	26,4724	26,3255	26,3189	26,3257	26,2597	26,3256	26,357	26,3709	26,3031		12	12	12	43,2	43,2	43,2	35,283	4,30E+09	100	26,3269	26,3306	26,3233	10	5	5	0,877587	-0,00733871	F9UL45	F9UL45	pts9AB	EIIAB-Man
27,3684	27,4767	27,5822	27,5457	27,6176	27,4003	27,5199	27,6499	27,4529	27,5361		16	16	15	47,3	47,3	45,8	54,545	9,30E+09	219	27,515	27,5181	27,5118	10	5	5	0,920573	-0,0062809	F9UUI9	F9UUI9	pbg9	6-phospho-beta-glucosidase
26,6162	26,4853	26,8992	26,7457	26,9203	26,6538	26,6414	26,764	26,6686	26,9192		4	4	4	17	17	17	33,861	1,11E+09	27	26,7314	26,7334	26,7294	10	5	5	0,968723	-0,00396576	F9UTJ8	F9UTJ8	lp_3318	Aldo/keto reductase family protein
25,9279	26,0515	26,1054	26,0582	26,1605	26,1415	25,9896	26,0554	25,9898	26,112		8	8	8	34,4	34,4	34,4	31,676	2,01E+09	52	26,0592	26,0607	26,0577	10	5	5	0,952179	-0,00306053	F9UP53	F9UP53	lp_1690	Hypothetical membrane protein, SPFH domain/Band 7 family
24,9028	25,6848	25,0212	25,0571	25,3044	25,5189	25,2953	24,918	25,331	24,8968		3	3	3	18,7	18,7	18,7	35,449	6,46E+08	36	25,193	25,1941	25,192	10	5	5	0,991422	-0,00205345	F9URT3	F9URT3	lp_2825	Purine nucleosidase, inosine-uridine preferring
25,464	25,9507	25,7786	25,8586	25,8001	25,4971	25,7005	25,8641	25,7644	26,0221		3	3	3	16	16	16	27,52	8,22E+08	23	25,77	25,7704	25,7697	10	5	5	0,995206	-0,00074234	Q890C2	Q890C2	thiM	Hydroxyethylthiazole kinase
26,8018	27,1275	26,8794	26,9895	26,7927	26,8614	26,8358	26,8976	27,0828	26,9164		5	5	5	33,6	33,6	33,6	17,452	2,31E+09	46	26,9185	26,9182	26,9188	10	5	5	0,993531	0,000640488	Q8VL04	Q8VL04	argR2	Arginine repressor
28,4069	28,5093	28,4885	28,4737	28,4863	28,4191	28,4117	28,4436	28,5863	28,5084		15	15	15	40,2	40,2	40,2	41,705	1,24E+10	166	28,4734	28,4729	28,4738	10	5	5	0,981768	0,000877762	F9UNR3	F9UNR3	lp_1528	Ribosome biogenesis GTPase, YqeH family
29,3672	29,4301	29,5654	29,4353	29,3312	29,3225	29,3119	29,5008	29,4975	29,5029		33	33	33	49,3	49,3	49,3	93,948	4,70E+10	451	29,4265	29,4259	29,4271	10	5	5	0,983886	0,00125313	F9UMD4	F9UMD4	pepN	Aminopeptidase
27,1207	27,2882	27,2404	27,2415	27,2758	27,1199	27,164	27,3771	27,2053	27,3075		10	10	10	26,5	26,5	26,5	57,67	4,56E+09	124	27,234	27,2333	27,2348	10	5	5	0,979928	0,00144768	F9URU6	F9URU6	tagE5	Poly(Glycerol-phosphate) alpha-glucosyltransferase
30,5395	30,7261	30,681	30,6903	30,6294	30,7257	30,7424	30,5794	30,6621	30,5643		30	30	30	71,2	71,2	71,2	31,615	9,53E+10	633	30,654	30,6533	30,6548	10	5	5	0,976426	0,00148697	Q88VJ5	Q88VJ5	tsf	Elongation factor Ts
27,8589	27,9032	27,9495	27,8949	27,9608	27,8718	27,8791	28,0218	27,9489	27,8539		34	34	34	47,8	47,8	47,8	86,281	1,89E+10	328	27,9143	27,9135	27,9151	10	5	5	0,964605	0,00166359	F9UPV0	F9UPV0	relA	(p)ppGpp synthase
27,3994	27,4546	27,3347	27,4072	27,4894	27,2397	27,3616	27,481	27,5506	27,462		7	7	7	39,4	39,4	39,4	15,819	5,97E+09	84	27,418	27,4171	27,419	10	5	5	0,975046	0,00194016	F9UNV4	F9UNV4	lp_1576	Rhodanese family protein
29,0033	29,1224	29,0811	29,0393	29,099	29,2721	29,2277	28,9425	28,9663	28,9495		13	13	13	72,6	72,6	72,6	24,462	2,55E+10	247	29,0703	29,069	29,0716	10	5	5	0,97373	0,00259132	Q88XW5	Q88XW5	adk	Adenylate kinase
24,8242	24,8966	25,1314	25,1354	25,1319	24,7109	25,1341	25,1737	25,1009	25,0147		4	4	4	17,7	17,7	17,7	24,96	8,62E+08	27	25,0254	25,0239	25,0269	10	5	5	0,978515	0,00298119	F9UQG1	F9UQG1	lp_2236	Uncharacterized protein
29,3469	29,5471	29,5273	29,4928	29,6079	29,6049	29,6469	29,4389	29,4205	29,4266		19	19	19	70,6	70,6	70,6	44,402	4,54E+10	385	29,506	29,5044	29,5076	10	5	5	0,962423	0,00318298	Q88UT5	Q88UT5	glyA	Serine hydroxymethyltransferase
27,6122	27,5435	27,6536	27,6356	27,464	27,4513	27,4911	27,6092	27,5963	27,78		29	29	29	41,8	41,8	41,8	80,715	1,61E+10	274	27,5837	27,5818	27,5856	10	5	5	0,956043	0,00381012	F9UPI3	F9UPI3	topA	DNA topoisomerase 1
25,194	25,291	25,3867	25,0783	25,4638	25,3837	25,4399	24,9628	25,448	25,199		2	2	2	13,3	13,3	13,3	29,63	4,59E+08	17	25,2847	25,2828	25,2867	10	5	5	0,973756	0,00390549	F9UM42	F9UM42	lp_0824	Hydrolase, HAD superfamily, Cof family
28,4476	28,6788	28,6896	28,6756	28,6883	28,4863	28,5675	28,6558	28,7709	28,7222		11	11	11	27,8	27,8	27,8	53,31	1,46E+10	161	28,6382	28,636	28,6405	10	5	5	0,949291	0,00458221	Q6LWE6	Q6LWE6	traF	Conjugation protein
27,1588	27,1971	27,1807	27,0639	27,0456	26,9636	27,0483	27,1854	27,2744	27,2042		9	9	9	27,6	27,6	27,6	34,525	4,26E+09	64	27,1322	27,1293	27,1352	10	5	5	0,929324	0,00589752	F9UR98	F9UR98	lp_2589	Polyphosphate:nucleotide phosphotransferase,PPK2 family
29,3955	29,3376	29,5319	29,4629	28,7805	29,4266	28,847	29,4593	29,3237	29,4824		15	15	15	24,2	24,2	24,2	103,76	5,85E+09	102	29,3047	29,3017	29,3078	10	5	5	0,973431	0,00615082	F9UNK2	F9UNK2	ftsK1	DNA translocase FtsK
27,8751	28,1711	28,0936	28,2126	28,2513	28,1982	28,2387	28,1096	28,0828	28,0112		17	17	17	62,8	62,8	62,8	33,263	1,66E+10	203	28,1244	28,1207	28,1281	10	5	5	0,927397	0,00735321	F9UMR9	F9UMR9	citE	Citrate (pro-3S)-lyase subunit beta
26,6442	26,9274	26,7015	26,7447	26,8145	26,9752	26,7095	26,8282	26,6638	26,7004		3	3	3	30,3	30,3	30,3	20,747	1,33E+09	27	26,7709	26,7664	26,7754	10	5	5	0,907697	0,00899391	F9URN3	F9URN3	lp_2762	Phosphohydrolase, MutT/nudix family
26,0179	26,3262	26,2391	26,4134	26,2668	26,0264	26,2613	26,2679	26,4209	26,3321		2	2	2	29,2	29,2	29,2	10,394	1,33E+09	37	26,2572	26,2527	26,2617	10	5	5	0,924723	0,00905457	Q88XS8	Q88XS8	citD	Citrate lyase acyl carrier protein
27,0197	26,9113	27,2837	26,9203	27,119	27,3318	27,0677	27,2048	26,7127	26,986		5	5	5	19,2	19,2	19,2	32,674	2,42E+09	52	27,0557	27,0508	27,0606	10	5	5	0,93977	0,00982132	F9UPH8	F9UPH8	lp_1843	Aldose 1-epimerase family protein
26,3396	25,8871	25,7158	25,2928	25,8005	25,9416	25,7085	25,7174	25,7877	25,9308		4	4	4	22,6	22,6	22,6	24,834	6,57E+08	20	25,8122	25,8072	25,8172	10	5	5	0,955811	0,0100163	F9URT1	F9URT1	lp_2823	ABC transporter, ATP-binding protein
27,4202	27,666	27,5878	27,6178	27,4777	27,3038	27,6384	27,6395	27,6818	27,5564		6	6	6	21	21	21	43,855	2,64E+09	31	27,559	27,5539	27,564	10	5	5	0,904818	0,0101162	Q88X61	Q88X61	sat	Sulfate adenylyltransferase
28,151	28,2721	28,1381	28,1058	27,9773	28,2007	28,1529	28,0929	28,2004	28,0483		7	7	7	41,6	41,6	41,6	18,23	4,04E+09	63	28,134	28,1289	28,139	10	5	5	0,860723	0,0101456	F9UT25	F9UT25	gpo	Glutathione peroxidase
28,5824	28,6785	28,7155	28,7346	28,6926	28,6983	28,79	28,6724	28,7446	28,5531		22	22	22	46	46	46	50,798	2,76E+10	349	28,6862	28,6807	28,6917	10	5	5	0,824676	0,0109795	F9UN93	F9UN93	pepV	Xaa-His dipeptidase
27,9068	28,0636	27,8534	27,9612	27,8914	27,844	28,0979	27,8382	28,0327	27,9188		3	3	3	19,4	19,4	19,4	15,758	3,40E+09	46	27,9408	27,9353	27,9463	10	5	5	0,866244	0,0109959	Q88WM9	Q88WM9	nusB	Transcription antitermination protein NusB
26,1016	26,2669	26,1092	26,0876	26,1613	25,9833	26,1067	26,265	26,2266	26,2068		5	5	5	53,1	53,1	53,1	11,677	2,03E+09	47	26,1515	26,1453	26,1577	10	5	5	0,843151	0,0123695	F9UPV5	F9UPV5	lp_1992	Uncharacterized protein
28,6589	28,7734	28,7152	28,6915	28,7003	28,7921	28,6236	28,6563	28,7531	28,7797		6	6	6	55,5	55,5	55,5	16,494	7,93E+09	60	28,7144	28,7079	28,721	10	5	5	0,745299	0,0130871	F9UKW1	F9UKW1	lp_0479	Uncharacterized protein
27,426	27,5356	27,4616	27,4727	27,4585	27,2812	27,5086	27,5326	27,5815	27,5166		18	18	18	45,1	45,1	45,1	63,639	1,20E+10	225	27,4775	27,4709	27,4841	10	5	5	0,817112	0,0132122	F9UM65	F9UM65	pox2	Pyruvate oxidase
24,3055	24,6105	24,6225	24,61	24,6101	24,3484	24,5931	24,7051	24,5713	24,6112		5	5	5	12,8	12,8	12,8	44,975	4,66E+08	24	24,5588	24,5517	24,5658	10	5	5	0,872707	0,014106	F9URV2	F9URV2	lp_2850	ABC transporter, permease protein
29,9553	29,9351	30,0371	29,9927	30,0022	30,0903	29,9657	30,036	29,8844	30,0188		51	51	49	66,9	66,9	62,7	88,73	1,40E+11	1225	29,9918	29,9845	29,999	10	5	5	0,720065	0,0145645	Q88U67	Q88U67	lp_2659	Probable phosphoketolase 1
26,6387	26,4645	26,4743	26,3135	26,1295	26,1977	26,352	26,4286	26,6512	26,4651		5	5	5	26,8	26,8	26,8	16,745	2,99E+09	58	26,4115	26,4041	26,4189	10	5	5	0,899095	0,0148312	F9UTK1	F9UTK1	lp_3322	Uncharacterized protein
24,8803	25,2764	25,3053	25,1048	25,0728	25,1922	25,2195	25,0668	25,0674	25,1701		8	8	8	28,4	28,4	28,4	37,901	1,20E+09	42	25,1355	25,1279	25,1432	10	5	5	0,859017	0,0152889	F9UQT2	F9UQT2	bcaT	Branched-chain-amino-acid transaminase
30,9491	31,0765	31,1079	31,1764	31,1951	31,139	31,0897	31,0715	31,1541	31,1277		21	21	21	61,9	61,9	61,9	39,906	7,87E+10	543	31,1087	31,101	31,1164	10	5	5	0,748383	0,0154049	F9UTA3	F9UTA3	guaB	Inosine 5-monophosphate (IMP) dehydrogenase
32,0723	32,1471	32,2112	32,1229	32,1907	32,1571	32,1494	32,1786	32,1823	32,1552		55	55	55	56,5	56,5	56,5	77,775	4,27E+11	1917	32,1567	32,1488	32,1645	10	5	5	0,556209	0,0156921	F9ULD9	F9ULD9	clpL	ATP-dependent Clp protease, ATP-binding subunit ClpL
32,4744	32,5327	32,5254	32,5657	32,6321	32,773	32,5732	32,4833	32,427	32,5523		13	13	13	88,4	88,4	88,4	18,021	1,60E+11	804	32,5539	32,5461	32,5618	10	5	5	0,813064	0,0157143	F9USN8	F9USN8	dps1	DNA-binding ferritin-like protein, DPS family
25,5831	25,763	25,7206	25,6114	25,7694	25,7297	25,6813	25,7816	25,7588	25,579		3	3	3	4,7	4,7	4,7	78,2	1,02E+09	25	25,6978	25,6895	25,7061	10	5	5	0,761536	0,0166107	F9UM34	F9UM34	nhaP1	Na(+)/H(+) antiporter
25,9564	26,0943	25,8514	25,9307	26,1185	25,8204	26,0491	26,0418	26,133	25,9918		3	3	3	16,2	16,2	16,2	20,293	9,31E+08	23	25,9987	25,9903	26,0072	10	5	5	0,820837	0,0169701	Q88Z39	Q88Z39	pth	Peptidyl-tRNA hydrolase
27,7722	27,8464	27,8709	27,8367	27,9609	27,7602	27,9024	27,898	27,9395	27,875		24	24	24	55	55	55	61,231	1,51E+10	252	27,8662	27,8574	27,875	10	5	5	0,694335	0,0175995	F9UMJ0	F9UMJ0	als	Acetolactate synthase
29,8837	29,8602	29,9217	29,8759	29,8121	29,8884	29,8514	29,8352	29,9047	29,9653		15	15	15	59,2	59,2	59,2	25,881	5,15E+10	358	29,8798	29,8707	29,889	10	5	5	0,545223	0,0182625	Q88VJ6	Q88VJ6	pyrH	Uridylate kinase
26,3873	26,5675	26,5914	26,5256	26,6462	26,4198	26,4796	26,599	26,6102	26,7023		15	15	15	37,2	37,2	37,2	43,378	4,83E+09	134	26,5529	26,5436	26,5622	10	5	5	0,786865	0,018602	F9UTI2	F9UTI2	folP	Dihydropteroate pyrophosphorylase
25,3669	25,8197	25,6724	25,561	25,5169	25,6172	25,5303	25,7541	25,6169	25,5118		4	4	4	25,3	25,3	25,3	21,649	9,05E+08	31	25,5967	25,5874	25,6061	10	5	5	0,835758	0,0186886	Q88XG4	Q88XG4	lp_1241	UPF0374 protein lp_1241
26,3633	26,4857	26,4563	26,5539	26,6422	26,4978	26,4961	26,4437	26,5941	26,5639		10	10	10	37,5	37,5	37,5	37,105	3,94E+09	116	26,5097	26,5003	26,5191	10	5	5	0,736372	0,0188099	F9UUF4	F9UUF4	rafR	Transcription regulator. LacI family, raffinose related
26,8027	26,9695	26,8001	26,8307	26,9605	26,9188	26,8367	26,9158	26,8567	26,9303		3	3	3	23,8	23,8	23,8	16,776	1,54E+09	36	26,8822	26,8727	26,8917	10	5	5	0,666964	0,0189686	F9UTD0	F9UTD0	lp_3227	Uncharacterized protein
28,9019	29,1527	28,9055	28,995	29,0601	28,8896	28,9422	29,0259	29,2304	29,0292		3	3	3	7,6	7,6	7,6	38,206	5,12E+09	36	29,0133	29,003	29,0235	10	5	5	0,792662	0,0204163	F9UPG0	F9UPG0	tarJ	Ribulose-5-phosphate reductase
29,5363	29,6103	29,1132	29,6234	29,5161	29,4269	29,4324	29,2921	29,703	29,6493		4	4	4	49,2	49,2	49,2	7,1604	7,05E+09	70	29,4903	29,4799	29,5007	10	5	5	0,867347	0,0208813	Q88XX3	Q88XX3	rpsZ	30S ribosomal protein S14 type Z
25,0577	25,1612	25,3088	25,3324	25,2256	25,3122	25,1537	25,1953	25,3136	25,2189		9	9	9	19	19	19	48,805	1,28E+09	51	25,2279	25,2171	25,2387	10	5	5	0,726021	0,0216042	F9UPK6	F9UPK6	lp_1877	TPR_REGION domain-containing protein
26,4342	26,554	26,5863	26,441	26,3973	26,487	26,4066	26,5843	26,4559	26,5886		37	37	37	52,4	52,4	52,4	98,801	1,08E+10	286	26,4935	26,4826	26,5045	10	5	5	0,680857	0,0219498	F9UNP5	F9UNP5	polA	DNA polymerase I
28,9545	29,0863	29,1277	29,116	29,1325	29,1047	29,1247	29,0422	29,1035	29,1532		15	15	15	60,4	60,4	60,4	35,829	2,29E+10	220	29,0945	29,0834	29,1056	10	5	5	0,573305	0,0222466	F9UQQ9	F9UQQ9	mreB2	Cell shape-determining protein MreB
28,0727	28,3175	28,1699	28,1781	28,1357	28,2247	28,1722	28,1645	28,2563	28,1682		11	11	11	60,4	60,4	60,4	21,044	1,25E+10	169	28,186	28,1748	28,1972	10	5	5	0,626005	0,0224136	F9URY3	F9URY3	lp_2887	NADP oxidoreductase, coenzyme F420-dependent
30,3777	30,5711	30,3956	30,48	30,6327	30,5201	30,5792	30,5099	30,4553	30,5054		14	14	14	63,3	63,3	63,3	16,469	3,94E+10	348	30,5027	30,4914	30,514	10	5	5	0,681936	0,0225704	Q890J8	Q890J8	rplI	50S ribosomal protein L9
27,5879	27,6232	27,6869	27,6034	27,6873	27,5185	27,5594	27,7846	27,7411	27,7011		19	19	19	27,4	27,4	27,4	73,283	7,52E+09	174	27,6493	27,6377	27,661	10	5	5	0,689361	0,0232121	F9UPG2	F9UPG2	tarL	Ribitolphosphotransferase
30,8524	30,9158	30,957	30,9829	31,0412	31,0223	31,017	30,902	30,966	30,9583		24	24	24	79,7	79,7	79,7	34,248	1,00E+11	749	30,9615	30,9499	30,9731	10	5	5	0,563331	0,0232716	F9ULY4	F9ULY4	galU	UTP--glucose-1-phosphate uridylyltransferase
30,6751	30,9681	30,9128	30,9177	30,9801	30,8285	30,9312	30,9426	30,9507	30,9173		22	22	22	45,1	45,1	45,1	35,879	8,31E+10	405	30,9024	30,8908	30,9141	10	5	5	0,706834	0,0232998	Q88Z84	Q88Z84	prs1	Ribose-phosphate pyrophosphokinase 1
26,5562	26,8734	26,6811	26,7225	26,9318	26,6369	26,9031	26,7943	26,8069	26,7601		3	3	3	14,9	14,9	14,9	19,323	2,11E+09	49	26,7666	26,753	26,7802	10	5	5	0,74225	0,027269	Q88WJ3	Q88WJ3	rimM	Ribosome maturation factor RimM
30,1469	30,2243	30,189	30,2007	30,2681	30,2724	30,339	30,1402	30,2495	30,1645		19	19	19	93	93	93	17,428	7,38E+10	416	30,2195	30,2058	30,2331	10	5	5	0,528183	0,0273434	Q88YI6	Q88YI6	luxS	S-ribosylhomocysteine lyase
24,6876	24,9256	24,801	24,6587	24,6534	24,6376	24,7275	24,8468	24,9096	24,7426		9	9	9	13,7	13,7	13,7	105,2	8,73E+08	37	24,7591	24,7453	24,7728	10	5	5	0,707598	0,0275345	F9UL55	F9UL55	manR	Sigma54 activator, mannose PTS operon regulator
28,3353	28,4439	28,456	28,4465	28,4919	28,4499	28,4997	28,501	28,4438	28,4181		40	40	40	63,7	63,7	63,7	76,796	4,73E+10	622	28,4486	28,4347	28,4625	10	5	5	0,395689	0,027774	Q88Z97	Q88Z97	metG	Methionine--tRNA ligase
25,2369	25,4481	25,3979	25,6528	25,4921	25,4202	25,4923	25,522	25,4829	25,4586		5	5	5	28,2	28,2	28,2	27,892	8,63E+08	26	25,4604	25,4456	25,4752	10	5	5	0,68121	0,0296406	F9UL35	F9UL35	tagA	N-acetylglucosaminyldiphosphoundecaprenol N-acetyl-beta-D-mannosaminyltransferase
28,2298	28,4552	28,4155	28,3817	28,5012	28,3362	28,4501	28,521	28,5235	28,3023		18	18	18	28,7	28,7	28,7	63,903	1,70E+10	280	28,4117	28,3967	28,4266	10	5	5	0,659108	0,0299026	F9UQ67	F9UQ67	rnj	Ribonuclease J
30,558	30,6872	30,7274	30,6577	30,7234	30,6652	30,7151	30,7355	30,7178	30,6743		21	21	21	64,9	64,9	64,9	38,256	9,89E+10	536	30,6862	30,6707	30,7016	10	5	5	0,38785	0,0308331	F9UR85	F9UR85	asd2	Aspartate-semialdehyde dehydrogenase
25,1721	25,3735	25,3135	25,3257	25,3117	25,1375	25,2251	25,3705	25,4885	25,4338		7	7	7	16,5	16,5	16,5	80,922	1,35E+09	42	25,3152	25,2993	25,3311	10	5	5	0,677092	0,0318001	F9UR92	F9UR92	lp_2580	Sulfatase, alkaline phosphatase superfamily,membrane-bound
27,4792	27,577	27,5131	27,6442	27,5021	27,5223	27,4681	27,6566	27,6674	27,5631		4	4	4	6,9	6,9	6,9	78,02	1,66E+09	22	27,5593	27,5431	27,5755	10	5	5	0,525232	0,0323959	F9UT33	F9UT33	mtlR	Transcription regulator, BglG family, mannitol operon
29,1956	29,2416	29,3551	29,2783	28,8281	29,228	29,0266	29,3679	29,1407	29,3014		35	35	35	48,5	48,5	48,5	106,32	3,12E+10	417	29,1963	29,1797	29,2129	10	5	5	0,769733	0,0331852	Q88V89	Q88V89	ileS	Isoleucine--tRNA ligase
26,9465	26,8225	26,9769	26,8202	26,8809	26,9861	26,8997	26,9015	26,8945	26,9325		9	9	9	37,4	37,4	37,4	29,235	3,38E+09	127	26,9061	26,8894	26,9228	10	5	5	0,382143	0,0334396	F9URN0	F9URN0	lp_2759	Hydrolase, HAD superfamily, Cof family
26,1715	26,362	26,3832	26,3421	26,116	26,3104	26,3122	26,3798	26,3803	26,1656		6	6	6	26,8	26,8	26,8	33,054	2,46E+09	65	26,2923	26,275	26,3097	10	5	5	0,619759	0,0347008	F9UTW0	F9UTW0	lp_0363	Purine nucleosidase
23,7941	23,8985	24,255	23,9701	24,2964	24,1716	24,0818	23,983	24,0165	24,1351		2	2	2	12,3	12,3	12,3	29,375	2,70E+08	12	24,0602	24,0428	24,0776	10	5	5	0,749797	0,0347881	F9UKV8	F9UKV8	lp_0475	Hydrolase, HAD superfamily, Cof family
27,9891	28,1745	28,1612	28,1785	28,1962	28,033	28,117	28,1512	28,233	28,3399		12	12	12	68,3	68,3	68,3	20,832	1,38E+10	217	28,1574	28,1399	28,1748	10	5	5	0,604147	0,0349117	F9UKZ2	F9UKZ2	lp_0515	Uncharacterized protein
29,9341	30,0665	30,0725	30,1234	30,1025	29,9496	29,9906	30,0878	30,207	30,239		10	10	10	77,5	77,5	77,5	17,551	5,53E+10	339	30,0773	30,0598	30,0948	10	5	5	0,610427	0,0350082	F9UQP4	F9UQP4	lp_2340	Universal stress protein
25,2208	25,4295	25,5421	25,1408	25,4282	25,3227	25,3481	25,485	25,3478	25,4404		14	14	14	28,9	28,9	28,9	74,176	1,98E+09	73	25,3705	25,3523	25,3888	10	5	5	0,661608	0,0365463	F9ULV5	F9ULV5	lp_0723	ABC transporter, ATP-binding protein
29,1368	29,0571	29,0209	29,0117	29,0221	29,1284	29,1384	29,0254	29,1226	29,017		13	13	13	53,6	53,6	53,6	31,233	1,97E+10	208	29,068	29,0497	29,0864	10	5	5	0,330397	0,0366409	F9UT26	F9UT26	lp_0221	2,5 diketo-D-gluconic acid-like reductase,NADP dependent (Promiscuous)
25,3736	25,5645	25,3281	25,5862	25,4505	25,4799	25,6731	25,6409	25,4455	25,248		3	3	3	11,9	11,9	11,9	27,806	7,03E+08	19	25,479	25,4606	25,4975	10	5	5	0,698336	0,0368755	Q88V56	Q88V56	mecA	Adapter protein MecA
29,6312	29,7504	29,6969	29,7564	29,7464	29,6842	29,6465	29,8205	29,8682	29,7534		16	16	16	53,5	53,5	53,5	26,372	2,77E+10	270	29,7354	29,7162	29,7545	10	5	5	0,443642	0,0383003	F9UNS7	F9UNS7	rrp5	Two-component system response regulator
28,7736	28,8217	28,8771	28,7132	28,8508	28,7878	28,7916	28,7862	29,0583	28,8084		8	8	8	22,2	22,2	22,2	30,615	1,17E+10	138	28,8269	28,8073	28,8465	10	5	5	0,535993	0,0391666	F9USZ6	F9USZ6	sacK1	Fructokinase
24,9518	25,2015	25,3079	25,084	25,2574	25,0261	25,0602	25,364	25,266	25,2842		7	7	7	23,3	23,3	23,3	32,024	1,38E+09	58	25,1803	25,1605	25,2001	10	5	5	0,679653	0,0395378	Q88Z91	Q88Z91	ispE	4-diphosphocytidyl-2-C-methyl-D-erythritol kinase
25,9304	26,1073	26,1991	26,175	26,0635	25,9658	26,1332	26,3087	26,2095	26,0635		8	8	8	32,1	32,1	32,1	33,625	2,41E+09	65	26,1156	26,0951	26,1361	10	5	5	0,602768	0,0410622	F9UL47	F9UL47	pts9D	PTS system, mannose-specific EIID component
29,6297	29,7163	29,8398	29,7388	29,0995	29,6347	29,3147	29,7331	29,6573	29,8917		97	97	97	70,5	70,5	70,5	135,32	1,40E+11	1627	29,6256	29,6048	29,6463	10	5	5	0,803509	0,0414566	Q88XZ2	Q88XZ2	rpoC	DNA-directed RNA polymerase subunit beta
27,3551	27,4094	27,2692	27,3519	27,3179	27,2713	27,4199	27,3167	27,4159	27,4888		9	9	9	68,1	68,1	68,1	12,611	4,92E+09	95	27,3616	27,3407	27,3825	10	5	5	0,383891	0,0418053	F9UQB2	F9UQB2	lp_2179	Cysteine desulfurase associated protein,DUF1831 family
24,126	24,6174	24,4341	24,0176	24,2031	24,1945	24,399	24,7083	24,4492	23,8677		2	2	2	16,6	16,6	16,6	17,962	3,92E+08	18	24,3017	24,2797	24,3237	10	5	5	0,810118	0,0440784	F9UUC1	F9UUC1	lp_3448	Acetyltransferase, GNAT family
27,4876	27,6759	27,7579	27,7181	27,5181	27,6785	27,662	27,5549	27,7475	27,7358		5	5	5	24,9	24,9	24,9	23,393	5,28E+09	69	27,6536	27,6315	27,6757	10	5	5	0,511058	0,0441929	F9UNZ5	F9UNZ5	rpe	Ribulose-phosphate 3-epimerase
25,458	25,7676	25,5031	25,4638	25,3944	25,6758	25,7033	25,4123	25,4927	25,5248		6	6	6	58,8	58,8	58,8	13,217	1,33E+09	36	25,5396	25,5174	25,5618	10	5	5	0,617173	0,0444019	Q88X33	Q88X33	lp_1415	UPF0342 protein lp_1415
28,9163	29,0276	29,0511	29,0515	29,0954	29,0441	29,0919	29,0595	29,0659	29,1048		17	17	17	61,8	61,8	61,8	25,213	2,05E+10	309	29,0508	29,0284	29,0732	10	5	5	0,198901	0,0448822	Q88YM8	Q88YM8	rex	Redox-sensing transcriptional repressor Rex
26,4472	26,6041	26,6411	26,6595	26,7255	26,5234	26,7448	26,6536	26,6811	26,699		9	9	9	32,3	32,3	32,3	35,838	4,61E+09	111	26,6379	26,6155	26,6604	10	5	5	0,472569	0,0449142	F9UPT5	F9UPT5	phoH	Phosphate starvation-inducible protein
26,0929	26,2218	26,2996	26,0978	26,3167	26,1053	26,235	26,2689	26,4965	26,1495		12	12	12	41,3	41,3	41,3	47,384	3,94E+09	135	26,2284	26,2058	26,251	10	5	5	0,60071	0,0452782	F9URK6	F9URK6	lp_2732	NADPH-dependent FMN reductase family protein
29,717	29,7057	29,7918	29,6371	29,7402	29,6152	29,8179	29,8391	29,9081	29,6414		22	22	22	41,4	41,4	41,4	63,536	2,98E+10	319	29,7414	29,7184	29,7644	10	5	5	0,485516	0,0459984	F9ULZ0	F9ULZ0	pgm	Phosphoglucomutase
25,2594	25,2898	25,3363	25,0249	25,0666	25,175	25,1962	25,2606	25,2787	25,299		8	8	8	14,7	14,7	14,7	70,498	1,12E+09	45	25,2186	25,1954	25,2419	10	5	5	0,50779	0,0465153	F9US62	F9US62	hpk1	Histidine kinase
25,4516	25,7991	25,697	25,7554	25,8874	25,6129	25,7063	25,8713	25,838	25,7953		7	7	7	20,8	20,8	20,8	47,977	1,87E+09	63	25,7414	25,7181	25,7648	10	5	5	0,607327	0,0466537	F9UTE4	F9UTE4	lp_3245	Uncharacterized protein
26,2125	25,9989	25,9811	26,0309	25,894	26,0568	26,2629	26,1147	26,0425	25,8753		7	7	7	23,9	23,9	23,9	50,08	2,23E+09	43	26,047	26,0235	26,0704	10	5	5	0,580472	0,0469624	Q88Z86	Q88Z86	glmU	Bifunctional protein GlmU
24,4809	24,7312	24,5284	24,6716	24,6345	24,5613	24,4635	24,6852	24,76	24,8148		7	7	7	23,7	23,7	23,7	49,877	7,03E+08	26	24,6331	24,6093	24,657	10	5	5	0,563915	0,047649	F9US09	F9US09	npp	Nucleotide pyrophosphatase

26,5008	27,0352	26,848	26,7099	26,3783	26,564	26,8104	26,6963	26,8853	26,7558		5	5	5	38,5	38,5	38,5	13,817	2,21E+09	56	26,7184	26,6944	26,7424	10	5	5	0,721418	0,0479214	F9UTI7	F9UTI7	folB	7,8-dihydroneopterin aldolase
28,4813	28,6933	28,6255	28,6126	28,6152	28,5835	28,7144	28,809	28,687	28,4758		24	24	24	58,1	58,1	58,1	53,901	1,55E+10	272	28,6298	28,6056	28,6539	10	5	5	0,48959	0,0483509	F9UP07	F9UP07	ffh	Signal recognition particle protein
25,3763	25,6915	25,4264	25,5606	25,5356	25,4397	25,6349	25,6576	25,6081	25,4926		2	2	2	7,4	7,4	7,4	31,66	6,46E+08	24	25,5423	25,5181	25,5666	10	5	5	0,505896	0,0484924	F9UQD9	F9UQD9	rrmA	rRNA large subunit methyltransferase A
28,0714	28,2394	28,1069	28,0651	28,2289	28,2408	28,1688	28,2648	28,162	28,1246		6	6	6	53,5	53,5	53,5	17,604	7,43E+09	101	28,1673	28,1423	28,1922	10	5	5	0,31255	0,0498772	F9UU78	F9UU78	lp_3397	Nucleoside 2-deoxyribosyltransferase
27,7124	27,833	27,7909	27,6959	27,77	27,8344	27,8347	27,8451	27,8174	27,7223		12	12	12	44,8	44,8	44,8	29,1	1,31E+10	226	27,7856	27,7605	27,8108	10	5	5	0,175727	0,0503174	F9UQM2	F9UQM2	minD	Cell division inhibitor MinD
25,9874	26,1254	26,1322	26,0267	26,1294	26,0755	26,1082	26,2437	26,0703	26,1618		9	9	9	42,1	42,1	42,1	23,928	2,66E+09	63	26,1061	26,0802	26,1319	10	5	5	0,278678	0,0516994	F9URW1	F9URW1	nth1	Endonuclease III
25,9484	26,0633	25,957	25,9605	26,1175	25,9654	26,0005	26,1376	26,178	26,0254		7	7	7	31,1	31,1	31,1	42,992	2,24E+09	47	26,0354	26,0093	26,0614	10	5	5	0,358238	0,0520714	F9USG7	F9USG7	amd	Aminohydrolase/peptidase, M20D family
24,7543	25,0138	24,6285	25,0518	25,0333	24,6881	25,2964	24,8387	24,9014	25,0198		2	2	2	15,1	15,1	15,1	10,412	4,51E+08	9	24,9226	24,8963	24,9489	10	5	5	0,704412	0,0525261	Q6LWH6	Q6LWH6	orf1	DNA-damage-inducible protein
28,6932	28,8463	28,8397	28,8469	28,8643	28,7457	28,8431	28,973	28,9753	28,8175		21	21	21	52,4	52,4	52,4	47,076	2,72E+10	357	28,8445	28,8181	28,8709	10	5	5	0,36471	0,0528263	Q88TV5	Q88TV5	tyrS	Tyrosine--tRNA ligase
30,4075	30,5688	30,6061	30,5677	30,6324	30,4435	30,5755	30,6356	30,7583	30,6375		32	32	32	63,6	63,6	63,6	52,982	1,28E+11	993	30,5833	30,5565	30,6101	10	5	5	0,430027	0,0535408	F9UNS5	F9UNS5	gnd2	6-phosphogluconate dehydrogenase, decarboxylating
31,7359	31,584	31,5094	31,5038	31,6456	31,6142	31,666	31,6073	31,6866	31,675		8	8	8	51,8	51,8	51,8	15,189	8,11E+10	402	31,6228	31,5957	31,6498	10	5	5	0,27978	0,0540733	Q88XZ0	Q88XZ0	rpsL	30S ribosomal protein S12
27,2934	27,6172	27,6267	27,5123	27,5024	27,4414	27,2995	27,5858	27,6633	27,8389		2	2	2	11,9	11,9	11,9	25,932	3,53E+09	36	27,5381	27,5104	27,5658	10	5	5	0,629014	0,05536	F9UPY7	F9UPY7	aldB	Alpha-acetolactate decarboxylase
27,7726	27,9484	27,9172	27,9038	27,7627	27,753	27,8699	28,1183	27,9709	27,8701		5	5	5	37,3	37,3	37,3	24,076	7,23E+09	80	27,8887	27,861	27,9164	10	5	5	0,465695	0,0554672	F9URM6	F9URM6	lp_2754	Hypothetical membrane protein
25,1941	25,4514	25,6341	25,3295	25,3349	25,3187	25,5315	25,474	25,4379	25,461		7	7	7	10,9	10,9	10,9	91,547	1,24E+09	55	25,4167	25,3888	25,4446	10	5	5	0,513073	0,0558144	Q88YC0	Q88YC0	pepX	Xaa-Pro dipeptidyl-peptidase
27,0777	27,3944	27,211	27,3265	27,237	27,3189	27,2823	27,3084	27,394	27,2255		15	15	15	49,1	49,1	49,1	43,112	6,50E+09	169	27,2776	27,2493	27,3058	10	5	5	0,377151	0,0565128	F9UPA9	F9UPA9	lp_1756	RNA methyltransferase
30,5944	30,6264	30,6837	30,5941	30,693	30,7192	30,7222	30,6593	30,7045	30,6716		26	26	26	60,1	60,1	60,1	48,375	6,37E+10	574	30,6668	30,6383	30,6954	10	5	5	0,0505899	0,0570217	Q88YE8	Q88YE8	glmM	Phosphoglucosamine mutase
26,5685	26,4148	26,6629	26,5104	26,9518	26,5331	26,6088	26,7027	26,7201	26,8339		9	9	9	34,3	34,3	34,3	32,203	3,58E+09	87	26,6507	26,6217	26,6797	10	5	5	0,596056	0,0580418	F9UT54	F9UT54	cbs	Cystathionine beta-synthase
24,2149	24,1313	24,5532	24,5822	24,1975	24,5023	24,4506	24,4955	24,3301	24,1921		4	4	4	6,6	6,6	6,6	92,986	3,20E+08	16	24,365	24,3358	24,3941	10	5	5	0,618528	0,0583206	F9UQA3	F9UQA3	recD	ATP-dependent RecD-like DNA helicase
28,8496	28,8713	28,9936	28,9562	29,1354	28,9676	29,0141	29,0472	29,0785	28,9914		19	19	19	53	53	53	36,329	2,67E+10	361	28,9905	28,9612	29,0197	10	5	5	0,315173	0,0585449	F9UQH6	F9UQH6	ccpA	Catabolite control protein A
26,799	27,0989	26,9608	27,1917	27,1149	26,9198	26,8906	27,3428	27,1476	27,1581		6	6	6	29,5	29,5	29,5	27,15	1,60E+09	29	27,0624	27,033	27,0918	10	5	5	0,603903	0,0587551	Q88SI6	Q88SI6	menG	Demethylmenaquinone methyltransferase
27,7612	27,8397	27,9437	27,7152	27,6743	27,8671	27,9591	27,7353	27,8659	27,8009		9	9	9	40,2	40,2	40,2	28,712	6,96E+09	109	27,8162	27,7868	27,8457	10	5	5	0,360971	0,0588432	F9USX2	F9USX2	lp_0159	Short-chain dehydrogenase/oxidoreductase
25,6275	25,8223	25,8125	25,9781	25,8334	25,7501	25,727	25,8938	25,9335	26,0671		7	7	7	33,6	33,6	33,6	31,726	1,49E+09	44	25,8445	25,8148	25,8743	10	5	5	0,497268	0,0595634	F9UMV7	F9UMV7	lp_1157	Transcription regulator, RpiR family
31,3239	31,5505	31,5448	31,5815	31,5891	31,5985	31,6015	31,5671	31,5627	31,558		27	27	27	70,9	70,9	70,9	34,205	1,66E+11	865	31,5477	31,5179	31,5776	10	5	5	0,268212	0,059642	P56512	P56512	ldh1	L-lactate dehydrogenase 1
28,4834	28,6485	28,6229	28,5649	28,5823	28,5552	28,6023	28,6903	28,6782	28,6765		19	19	19	40,1	40,1	40,1	71,921	2,17E+10	271	28,6105	28,5804	28,6405	10	5	5	0,159765	0,0600574	F9UUC0	F9UUC0	pepO	Endopeptidase Pep
29,5444	29,7989	29,8001	29,7688	29,8544	29,6585	29,8487	29,8535	29,9824	29,726		24	24	24	57	57	57	54,961	4,62E+10	422	29,7836	29,7533	29,8138	10	5	5	0,459739	0,0605045	F9UMS0	F9UMS0	citF	Citrate lyase alpha chain
28,997	29,0524	29,1785	29,0284	28,859	29,0259	28,9669	29,1111	29,1203	29,1964		46	46	46	58,8	58,8	58,8	97,264	5,72E+10	704	29,0536	29,023	29,0841	10	5	5	0,374735	0,0610695	Q88V10	Q88V10	alaS	Alanine--tRNA ligase
31,4786	31,5248	31,5775	31,5276	31,6425	31,6085	31,6673	31,5816	31,6476	31,5532		30	30	30	69,4	69,4	69,4	50,831	2,23E+11	1419	31,5809	31,5502	31,6116	10	5	5	0,115735	0,061441	Q88UU3	Q88UU3	atpD	ATP synthase subunit beta
24,4681	24,8633	24,9466	24,65	24,9531	24,5632	24,7577	25,0203	24,9941	24,8531		2	2	2	5,3	5,3	5,3	55,951	3,23E+08	21	24,8069	24,7762	24,8377	10	5	5	0,639406	0,0614529	Q88YD9;Q88Z	Q88YD9;Q88ZF1	glpK2	Glycerol kinase 2
25,4552	25,4926	25,109	25,2248	24,9693	24,923	25,3978	25,3552	25,5672	25,3157		5	5	5	62,4	62,4	62,4	12,746	8,62E+08	40	25,281	25,2502	25,3118	10	5	5	0,683884	0,0616348	F9URZ5	F9URZ5	lp_2903	Transcription regulator, ArsR family
25,7512	25,7035	25,679	25,6414	25,8261	25,7608	25,8308	25,755	25,8015	25,7617		3	3	3	12,4	12,4	12,4	29,824	1,02E+09	31	25,7511	25,7202	25,782	10	5	5	0,117223	0,061718	Q88V61	Q88V61	nadK	NAD kinase
30,9807	30,9415	30,9639	30,9343	30,9942	30,9739	31,0768	31,0113	31,1018	30,9618		32	32	32	79,8	79,8	79,8	30,225	1,91E+11	1341	30,994	30,9629	31,0251	10	5	5	0,0714965	0,0622063	Q88VJ4	Q88VJ4	rpsB	30S ribosomal protein S2
33,0778	33,2667	33,3534	33,3324	33,4442	33,2763	33,4034	33,3632	33,3839	33,3629		45	45	45	83,5	83,5	83,5	43,377	1,14E+12	6704	33,3264	33,2949	33,3579	10	5	5	0,36049	0,0630463	Q88VE0	Q88VE0	tuf	Elongation factor Tu
30,9457	30,9537	30,8621	30,9809	31,019	31,0092	30,9783	31,0093	30,992	31,0931		17	17	17	84,7	84,7	84,7	14,643	7,60E+10	678	30,9843	30,9523	31,0164	10	5	5	0,0862921	0,0640839	Q88XX2	Q88XX2	rpsH	30S ribosomal protein S8
27,8704	28,0804	28,0395	28,035	28,09	27,8805	28,0273	28,1819	28,2251	28,123		26	26	26	59,9	59,9	59,9	61,806	2,05E+10	381	28,0553	28,023	28,0876	10	5	5	0,403512	0,0645275	F9UQ97	F9UQ97	rnj	Ribonuclease J
29,0889	29,1744	29,2705	29,2202	29,3296	29,181	29,2267	29,3077	29,3102	29,3846		22	22	22	60,2	60,2	60,2	36,844	4,20E+10	504	29,2494	29,2167	29,282	10	5	5	0,263541	0,0653229	F9UPH4	F9UPH4	lp_1838	Transcription regulator, LysR family
30,0491	30,1002	30,2462	30,1954	30,2016	30,1241	30,2239	30,2264	30,3065	30,2466		22	22	22	64	64	64	34,522	4,25E+10	382	30,192	30,1585	30,2255	10	5	5	0,189048	0,0669987	Q88UU2	Q88UU2	atpG	ATP synthase gamma chain
27,8397	27,8435	27,9212	27,7508	27,9545	27,8866	27,9605	28,0548	27,9699	27,7758		11	11	11	41,8	41,8	41,8	34,538	7,92E+09	142	27,8957	27,8619	27,9295	10	5	5	0,283231	0,0675762	Q88VS2	Q88VS2	glyQ	Glycine--tRNA ligase alpha subunit
32,4961	32,816	32,4989	32,8274	32,7611	32,4912	32,7509	32,7527	32,8751	32,8702		17	17	17	91,2	91,2	91,2	9,6209	3,55E+11	1203	32,714	32,6799	32,748	10	5	5	0,52531	0,0681274	F9UPK7	F9UPK7	hbsU	DNA-binding protein HU
25,3412	25,5288	25,6349	25,5505	25,4852	25,6465	25,6317	25,6772	25,4481	25,4799		4	4	4	12,6	12,6	12,6	33,164	1,33E+09	45	25,5424	25,5081	25,5767	10	5	5	0,338124	0,0685577	F9UQ89	F9UQ89	lp_2150	Malate/L-lactate/L-2-hydroxyisocapronate dehydrogenase
26,5218	26,6734	26,751	26,6994	26,555	26,4349	26,6867	26,8227	26,8578	26,7428		12	12	12	26,4	26,4	26,4	64,417	4,29E+09	89	26,6746	26,6401	26,709	10	5	5	0,449377	0,0688747	F9UPE1	F9UPE1	rqcH	Rqc2 homolog RqcH
26,7575	26,9284	26,7827	26,8991	26,7797	26,8453	26,5688	27,0649	27,1321	26,882		4	4	4	30,3	30,3	30,3	12,004	1,76E+09	37	26,864	26,8295	26,8986	10	5	5	0,526828	0,0691563	F9ULT9	F9ULT9	lp_0704	Uncharacterized protein
27,4369	27,6078	27,6817	27,6509	27,7221	27,6492	27,6611	27,81	27,7136	27,6153		7	7	7	15,3	15,3	15,3	49,997	4,22E+09	82	27,6549	27,6199	27,6898	10	5	5	0,276954	0,0699505	F9ULL7	F9ULL7	pepC2	Aminopeptidase
25,3696	25,4725	25,5968	25,557	25,4816	25,5215	25,5555	25,6595	25,557	25,5379		11	11	11	22,1	22,1	22,1	75,994	2,00E+09	100	25,5309	25,4955	25,5663	10	5	5	0,162499	0,070776	F9US42	F9US42	lp_0012	Cyclic-di-AMP phosphodiesterase
25,8856	25,3946	25,3889	25,2294	25,2581	25,4785	25,4607	25,5192	25,6438	25,4092		8	8	8	26,1	26,1	26,1	26,609	1,70E+09	55	25,4668	25,4313	25,5023	10	5	5	0,585438	0,0709305	F9UTA4	F9UTA4	lp_3195	Hypothetical membrane protein, DUF1129 family
26,378	26,3275	26,3908	26,3031	26,4098	26,5027	26,4945	26,4763	26,4613	26,2318		7	7	7	41,6	41,6	41,6	24,314	2,49E+09	56	26,3976	26,3618	26,4333	10	5	5	0,22747	0,0714729	Q88YP6	Q88YP6	tmk	Thymidylate kinase
25,5582	25,7791	25,7444	25,5703	25,6738	25,7378	25,5497	25,6547	25,8878	25,855		3	3	3	10,4	10,4	10,4	45,482	6,37E+08	26	25,7011	25,6651	25,737	10	5	5	0,377532	0,0718449	F9UU32	F9UU32	mvaA	3-hydroxy-3-methylglutaryl coenzyme A reductase
31,3886	31,4816	31,4928	31,4778	31,5636	31,4527	31,5849	31,5894	31,6371	31,5042		34	34	34	66,2	66,2	66,2	49,846	2,19E+11	1130	31,5173	31,4809	31,5537	10	5	5	0,130703	0,0727623	Q88UI4	Q88UI4	pgi	Glucose-6-phosphate isomerase
26,556	26,7651	26,6863	26,5926	26,7437	26,7017	26,7787	26,7145	26,7791	26,7341		10	10	10	41,3	41,3	41,3	31,69	2,92E+09	62	26,7052	26,6688	26,7416	10	5	5	0,136884	0,0728779	F9UTA9	F9UTA9	parB2	Chromosome partitioning protein, DNA-binding protein
26,7206	26,8255	26,7924	26,7178	26,7778	26,7735	26,8831	26,796	26,9203	26,8295		4	4	4	19,5	19,5	19,5	22,914	2,60E+09	44	26,8037	26,7668	26,8405	10	5	5	0,0640044	0,0736488	F9UNR6	F9UNR6	lp_1531	Hydrolase, HAD superfamily
29,7197	30,0688	29,9078	30,1943	30,0208	29,8412	30,0907	30,2275	30,006	30,1143		8	8	8	82,8	82,8	82,8	11,428	3,50E+10	170	30,0191	29,9823	30,0559	10	5	5	0,493707	0,073661	Q890K2	Q890K2	rpsF	30S ribosomal protein S6
27,765	27,9262	27,9315	27,87	27,9432	27,9159	27,961	28,0076	27,9493	27,9727		17	17	17	61,4	61,4	61,4	38,175	1,31E+10	213	27,9242	27,8872	27,9613	10	5	5	0,0752671	0,0741253	F9URD8	F9URD8	lplA1	Lipoate--protein ligase
24,5082	24,9223	24,9465	24,7565	24,9771	24,9473	24,8326	25,0866	24,8338	24,7848		4	4	4	19,5	19,5	19,5	30,151	6,95E+08	36	24,8596	24,8221	24,897	10	5	5	0,487405	0,0748753	F9UMP2	F9UMP2	ecfT	Energy-coupling factor transporter transmembrane protein EcfT
31,1661	31,3321	31,1151	31,172	31,2458	31,2832	31,3127	31,2599	31,3326	31,2209		9	9	9	58,3	58,3	58,3	16,075	9,56E+10	457	31,244	31,2062	31,2818	10	5	5	0,113479	0,075605	Q88XX9	Q88XX9	rplP	50S ribosomal protein L16
25,5483	25,5673	25,5557	25,4777	25,5224	25,8781	25,7112	25,465	25,5165	25,4796		7	7	7	44,6	44,6	44,6	29,415	1,60E+09	36	25,5722	25,5343	25,6101	10	5	5	0,381516	0,0757877	F9UU39	F9UU39	lp_0456	DNase, TatD family
26,5989	26,911	26,966	26,943	26,9554	26,804	26,9247	27,0016	27,0485	26,9748		8	8	8	31,6	31,6	31,6	36,991	3,93E+09	81	26,9128	26,8749	26,9507	10	5	5	0,377406	0,0758686	F9UNG8	F9UNG8	lp_1418	Metal-dependent phosphohydrolase, HD family
28,351	28,5226	28,4452	28,5193	28,5808	28,5539	28,5656	28,5601	28,5456	28,574		20	20	20	58,9	58,9	58,9	48,641	2,49E+10	354	28,5218	28,4838	28,5598	10	5	5	0,0928619	0,0760406	F9UMW9	F9UMW9	gdh	Glutamate dehydrogenase
31,6923	31,8361	31,6567	31,801	31,7481	31,8406	31,8263	31,8365	31,8118	31,7994		24	24	24	76	76	76	24,208	1,52E+11	586	31,7849	31,7468	31,8229	10	5	5	0,0558858	0,0761002	Q88XY0	Q88XY0	rpsC	30S ribosomal protein S3
26,2563	26,6873	26,3246	26,4556	26,4669	26,525	26,6029	26,4221	26,5498	26,4743		7	7	7	38,3	38,3	38,3	18,591	3,06E+09	72	26,4765	26,4381	26,5148	10	5	5	0,366826	0,0766602	F9URF2	F9URF2	lp_2662	Cys-tRNA(Pro)/Cys-tRNA(Cys) deacylase
24,0313	24,0096	24,3076	24,2353	24,1977	24,0918	24,2062	24,2917	24,4735	24,1036		6	6	6	23,2	23,2	23,2	35,112	4,82E+08	39	24,1948	24,1563	24,2333	10	5	5	0,423243	0,0770481	Q88YI2	Q88YI2	whiA	Probable cell division protein WhiA
25,4767	25,6109	25,5249	25,5523	25,504	25,4709	25,6032	25,7335	25,7118	25,5382		10	10	10	48,8	48,8	48,8	37,552	1,27E+09	50	25,5726	25,5338	25,6115	10	5	5	0,19584	0,0777367	Q88Y39	Q88Y39	asnA	Aspartate--ammonia ligase
30,9286	30,9905	31,1028	31,0735	31,0438	31,063	31,1254	31,1079	31,064	31,1679		12	12	12	67,8	67,8	67,8	12,481	6,15E+10	524	31,0667	31,0278	31,1057	10	5	5	0,0671415	0,077808	Q88XY1	Q88XY1	rplV	50S ribosomal protein L22
26,9074	27,1503	27,019	27,1914	27,1436	27,2069	26,9628	27,3068	27,1815	27,1449		4	4	4	11,4	11,4	11,4	34,247	1,84E+09	40	27,1215	27,0823	27,1606	10	5	5	0,338476	0,0782631	Q88WR8	Q88WR8	yidC1	Membrane protein insertase YidC 1
26,2664	26,5243	26,3997	26,5219	26,4131	26,4858	26,3629	26,6424	26,5141	26,5141		5	5	5	15	15	15	56,08	1,62E+09	31	26,4645	26,4251	26,5039	10	5	5	0,260718	0,0787834	Q88VM6	Q88VM6	dltA	D-alanine--D-alanyl carrier protein ligase
26,6122	26,9032	26,8483	26,7191	26,6181	26,7401	26,7695	26,872	26,8624	26,8548		5	5	5	26,9	26,9	26,9	19,886	2,69E+09	41	26,78	26,7402	26,8198	10	5	5	0,255982	0,0795986	Q88WV2	Q88WV2	nrdR	Transcriptional repressor NrdR
26,747	26,6409	26,6755	26,5982	26,6182	26,6421	26,7537	26,8187	26,8048	26,6596		9	9	9	42,1	42,1	42,1	27,557	2,15E+09	72	26,6959	26,6559	26,7358	10	5	5	0,112833	0,0798306	F9UR35	F9UR35	rrp7	Two-component system response regulator
28,095	28,3138	28,3131	28,3755	28,3673	28,2641	28,3442	28,4624	28,3946	28,4082		24	24	24	78,6	78,6	78,6	45,83	1,83E+10	268	28,3338	28,2929	28,3747	10	5	5	0,217944	0,081744	Q88Z54	Q88Z54	murA2	UDP-N-acetylglucosamine 1-carboxyvinyltransferase 2
24,1023	24,2725	24,1003	24,3981	24,3128	24,3352	24,2535	24,34	24,3075	24,3587		4	4	4	10,8	10,8	10,8	65,86	4,06E+08	23	24,2781	24,2372	24,319	10	5	5	0,222489	0,0817955	F9USG4	F9USG4	lp_3040	Multidrug ABC transporter, ATP-binding and permease protein
28,4533	28,6475	28,5808	28,5751	28,6374	28,6334	28,5901	28,6908	28,7003	28,6895		9	9	9	31,2	31,2	31,2	50,22	1,20E+10	146	28,6198	28,5788	28,6609	10	5	5	0,0779369	0,0820358	F9UTP6	F9UTP6	glcD	Glycolate dehydrogenase, subunit GlcD,FAD-binding
28,3351	28,3007	28,3285	28,2891	28,5152	28,4228	28,4659	28,4583	28,4231	28,4135		9	9	9	57,8	57,8	57,8	29,082	1,25E+10	121	28,3952	28,3537	28,4367	10	5	5	0,0870665	0,0830219	F9UT55	F9UT55	pepM	Methionine aminopeptidase
29,8445	30,0115	29,9157	29,8672	29,872	29,9892	29,9796	29,9064	30,0757	29,9775		12	12	12	79,3	79,3	79,3	15,999	5,05E+10	457	29,9439	29,9022	29,9857	10	5	5	0,0705684	0,0835239	F9USV1	F9USV1	hsp1	Small heat shock protein
26,6234	27,0871	27,1375	26,9663	26,9085	26,844	26,8443	27,251	27,1613	27,0473		4	4	4	25,9	25,9	25,9	19,801	2,25E+09	64	26,9871	26,9446	27,0296	10	5	5	0,505844	0,0850048	F9UM35	F9UM35	lp_0816	Transcription regulator, MarR family
28,0807	28,3997	28,0262	28,3438	28,1386	28,2852	28,3748	28,0471	28,2498	28,4574		7	7	7	69	69	69	12,926	5,16E+09	51	28,2403	28,1978	28,2829	10	5	5	0,424467	0,0850674	Q88W95	Q88W95	gpsB	Cell cycle protein GpsB
25,2848	25,2778	25,362	25,2627	25,4904	25,1603	25,4928	25,4554	25,5148	25,48		7	7	7	21,3	21,3	21,3	57,479	1,35E+09	50	25,3781	25,3355	25,4207	10	5	5	0,308354	0,0851234	F9UR34	F9UR34	hpk7	Histidine kinase
26,5425	26,7352	26,7049	26,724	26,759	26,7231	26,6978	26,8644	26,9558	26,6503		5	5	5	17,1	17,1	17,1	36,604	2,16E+09	75	26,7357	26,6931	26,7783	10	5	5	0,250981	0,0851639	F9UM03	F9UM03	lp_0780	Putative gluconeogenesis factor
27,0495	27,1722	27,1866	27,2121	27,1994	27,0488	27,2133	27,2962	27,2813	27,4067		12	12	12	42,3	42,3	42,3	37,693	7,57E+09	184	27,2066	27,1639	27,2493	10	5	5	0,231211	0,0853325	F9UMI5	F9UMI5	lp_1000	Transcriptional attenuator, cell envelope-related, LytR family
26,6815	27,0582	27,1062	27,0571	26,9796	26,9146	26,9075	27,2435	27,0941	27,1513		5	5	5	32,2	32,2	32,2	24,087	2,15E+09	61	27,0193	26,9765	27,0622	10	5	5	0,421625	0,0856594	F9UQ15	F9UQ15	plsC	1-acylglycerol-3-phosphate O-acyltransferase
25,091	25,0783	25,1573	25,1259	25,3638	24,9481	25,2117	25,2009	25,4093	25,4754		9	9	9	32,9	32,9	32,9	50,493	9,57E+08	42	25,2062	25,1633	25,2491	10	5	5	0,44229	0,0857883	Q88WM7	Q88WM7	xseA	Exodeoxyribonuclease 7 large subunit
32,0662	32,0917	31,9764	32,0336	32,0439	31,9385	32,0745	32,1793	32,2933	32,1564		24	24	24	65,6	65,6	65,6	30,191	1,78E+11	1126	32,0854	32,0424	32,1284	10	5	5	0,202885	0,0860306	Q88XY3	Q88XY3	rplB	50S ribosomal protein L2
25,9991	26,0697	26,1602	26,2038	26,1263	26,1837	26,1224	26,2058	26,3195	26,1584		9	9	9	31,4	31,4	31,4	40,055	2,24E+09	68	26,1549	26,1118	26,198	10	5	5	0,116028	0,086166	F9UMA6	F9UMA6	lp_0903	Phosphohydrolase, MutT/nudix family
28,1394	28,4309	28,3034	28,3484	28,3398	28,3551	28,4613	28,4962	28,426	28,2562		12	12	12	46,9	46,9	46,9	30,589	1,42E+10	230	28,3557	28,3124	28,399	10	5	5	0,214426	0,0865898	Q88Z14	Q88Z14	nadE	NH(3)-dependent NAD(+) synthetase
27,663	27,9061	28,0171	27,9335	28,0238	27,9489	27,8966	28,0176	27,9647	28,1502		7	7	7	29,6	29,6	29,6	35,905	8,09E+09	140	27,9522	27,9087	27,9956	10	5	5	0,300515	0,0869061	F9UMU0	F9UMU0	lp_1136	Oxidoreductase, NAD(P)-dependent
27,354	27,4855	27,132	27,3313	27,1461	27,1158	27,3743	27,3196	27,5767	27,4982		7	7	7	31,6	31,6	31,6	13,097	4,18E+09	85	27,3334	27,2898	27,3769	10	5	5	0,425769	0,0871567	Q88VK8	Q88VK8	rbfA	Ribosome-binding factor A
25,8157	25,9333	25,9722	25,9544	25,8335	25,849	25,985	26,1139	26,019	25,982		13	13	13	61,8	61,8	61,8	26,334	3,45E+09	104	25,9458	25,9018	25,9898	10	5	5	0,137825	0,0879765	F9UL34	F9UL34	lp_0563	Transcription regulator, GntR family
27,7234	27,9194	27,9107	27,857	27,951	27,8774	27,9457	28,0555	27,9572	27,9716		35	35	35	53,5	53,5	53,5	78,531	2,11E+10	378	27,9169	27,8723	27,9615	10	5	5	0,108106	0,0891575	Q88VS3	Q88VS3	glyS	Glycine--tRNA ligase beta subunit
28,7316	28,9237	28,8816	28,8661	28,9331	28,9436	28,9535	29,0375	29,0026	28,8456		40	40	40	81,9	81,9	81,9	53,192	5,69E+10	657	28,9119	28,8672	28,9565	10	5	5	0,103536	0,089315	Q88XP6	Q88XP6	gatB	Aspartyl/glutamyl-tRNA(Asn/Gln) amidotransferase subunit B
29,1565	29,1607	29,2763	29,1438	29,267	29,2067	29,3174	29,3056	29,3851	29,2368		35	35	35	57	57	57	67,602	4,25E+10	481	29,2456	29,2008	29,2903	10	5	5	0,0702073	0,0894665	Q88VQ8	Q88VQ8	aspS	Aspartate--tRNA ligase
25,7019	25,7364	25,692	25,9933	25,8764	25,7923	26,0318	25,8056	25,9222	25,8977		10	10	10	23,2	23,2	23,2	51,76	1,61E+09	61	25,845	25,8	25,8899	10	5	5	0,252651	0,0899197	F9US44	F9US44	dnaC	Replicative DNA helicase
27,3567	27,3302	27,309	27,1584	27,5347	27,5023	27,3801	27,4068	27,4493	27,4017		8	8	8	21,4	21,4	21,4	40,686	3,32E+09	80	27,3829	27,3378	27,428	10	5	5	0,195434	0,0902519	Q88ZJ6	Q88ZJ6	potA	Spermidine/putrescine import ATP-binding protein PotA
29,103	29,1787	29,1977	29,2359	29,32	29,2507	29,3723	29,3015	29,2739	29,2881		27	27	27	52,8	52,8	52,8	52,088	2,70E+10	361	29,2522	29,2071	29,2973	10	5	5	0,0592388	0,0902569	Q88XP7	Q88XP7	gatA	Glutamyl-tRNA(Gln) amidotransferase subunit A
25,5672	25,8534	25,8774	25,7488	25,7821	25,7902	25,9369	25,9677	25,8851	25,7012		3	3	3	15,3	15,3	15,3	29,674	1,27E+09	31	25,811	25,7658	25,8562	10	5	5	0,254015	0,0904224	F9UQC0	F9UQC0	lp_2190	Cell division protein, RNA-binding S4 domain-containing protein
26,2568	26,3802	26,3801	26,13	26,1723	26,573	26,2585	26,3063	26,2958	26,3447		6	6	6	41,6	41,6	41,6	16,917	2,25E+09	55	26,3098	26,2639	26,3557	10	5	5	0,263094	0,0917671	F9UR52	F9UR52	lp_2528	Lactoylglutathione lyase family protein
27,2695	27,468	27,4669	27,3909	27,3519	27,4597	27,6913	27,4801	27,4801	27,2954		12	12	12	43,2	43,2	43,2	42,652	4,65E+09	88	27,4354	27,3895	27,4813	10	5	5	0,244874	0,0918488	Q88YL5	Q88YL5	prfB	Peptide chain release factor 2
25,5356	25,2747	25,3163	25,4293	25,1544	25,4798	25,5291	25,3154	25,4083	25,4384		5	5	5	19,7	19,7	19,7	33,115	7,22E+08	23	25,3881	25,3421	25,4342	10	5	5	0,252007	0,0921371	P59390	P59390	ldh2	L-lactate dehydrogenase 2
25,9925	26,1751	26,2268	26,081	26,0684	25,9809	26,0857	26,2681	26,4485	26,2218		11	11	11	48,8	48,8	48,8	32,356	2,98E+09	76	26,1549	26,1087	26,201	10	5	5	0,335606	0,0922531	F9UTW1	F9UTW1	lp_0364	Short-chain dehydrogenase/oxidoreductase
27,2395	27,3235	27,459	27,2947	26,9056	27,1819	27,18	27,4571	27,3807	27,4877		33	33	33	37,1	37,1	37,1	127,2	1,68E+10	377	27,291	27,2445	27,3375	10	5	5	0,436178	0,0929821	F9UQ80	F9UQ80	pycA	Pyruvate carboxylase
26,3142	26,5695	26,5356	26,5746	26,3082	26,5741	26,8023	26,5488	26,6362	26,2065		2	2	2	8,3	8,3	8,3	23,648	1,20E+09	22	26,507	26,4604	26,5536	10	5	5	0,441471	0,0931656	Q6LWE1	Q6LWE1	orf36	Uncharacterized protein
25,9629	26,1734	26,0589	26,0796	26,1139	26,0095	26,4231	26,2394	26,4457	25,7428		5	5	5	28,6	28,6	28,6	20,852	8,70E+08	20	26,1249	26,0777	26,1721	10	5	5	0,511384	0,0943668	F9URX7	F9URX7	maa	Maltose L-acetyltransferase
26,3003	26,6262	26,5234	26,3905	26,3315	26,3454	26,4947	26,6759	26,5111	26,6197		4	4	4	25,9	25,9	25,9	23,543	2,28E+09	49	26,4819	26,4344	26,5294	10	5	5	0,289383	0,0949982	F9UTM7	F9UTM7	lp_3355	Short-chain dehydrogenase/oxidoreductase,atypical SDR family, subgroup 6
27,5733	27,7646	27,6691	27,6585	27,6903	27,7025	27,8783	27,7468	27,7862	27,7272		21	21	21	60	60	60	52,764	1,60E+10	227	27,7197	27,6712	27,7682	10	5	5	0,0559803	0,0970463	Q88YX9	Q88YX9	cysS	Cysteine--tRNA ligase
28,8533	28,9688	29,0387	28,9036	29,0174	28,8997	29,0848	29,154	29,1215	29,0106		39	39	39	66,2	66,2	66,2	62,909	3,79E+10	536	29,0053	28,9564	29,0541	10	5	5	0,125418	0,0977474	Q88X53	Q88X53	argS	Arginine--tRNA ligase
26,6632	26,5014	26,4887	26,6532	26,5218	26,4245	26,6651	26,8942	26,7782	26,5575		9	9	9	16,8	16,8	16,8	54,145	2,45E+09	50	26,6148	26,5657	26,6639	10	5	5	0,30955	0,098222	F9UNF9	F9UNF9	lp_1407	Aminotransferase (PLP-dependent) with N-terminal regulator domain, GntR family
29,5137	29,5376	29,5117	29,5934	29,5461	29,5204	29,5536	29,6652	29,7711	29,6842		10	10	10	61,4	61,4	61,4	19,25	1,67E+10	141	29,5897	29,5405	29,6389	10	5	5	0,0740501	0,0984077	F9UQF4	F9UQF4	lp_2230	ABM domain-containing protein
26,5467	26,747	26,6396	26,7058	26,7264	26,6277	26,727	26,7953	26,8918	26,8178		19	19	19	48,1	48,1	48,1	48,946	7,64E+09	174	26,7225	26,6731	26,7719	10	5	5	0,124502	0,0988056	F9UKY8	F9UKY8	rho	Transcription termination factor Rho
28,6162	28,72	28,7755	28,6974	28,7974	28,6871	28,8385	28,8939	28,9044	28,7785		40	40	40	73,3	73,3	73,3	73,139	4,93E+10	645	28,7709	28,7213	28,8205	10	5	5	0,0893409	0,099189	F9UMH1;F9U	F9UMH1	asnB1	Asparagine synthase (Glutamine-hydrolysing)
26,3241	26,3524	26,6055	26,4583	26,6834	26,2934	26,5797	26,6765	26,7314	26,6415		8	8	8	20,6	20,6	20,6	68,489	1,74E+09	42	26,5346	26,4848	26,5845	10	5	5	0,365352	0,0997635	F9UMA5	F9UMA5	napA2	Na(+)/H(+) antiporter
25,9095	26,0752	26,1349	25,8695	26,0317	26,1284	26,17	26,1069	26,1312	25,9842		3	3	3	37,3	37,3	37,3	12,999	1,21E+09	30	26,0541	26,0042	26,1041	10	5	5	0,129632	0,0999714	F9UNR7	F9UNR7	rsfS	Ribosomal silencing factor RsfS
26,7724	26,9373	26,9038	26,8214	26,9209	26,891	26,9683	27,0249	27,1325	26,8529		15	15	15	42,2	42,2	42,2	51,462	5,89E+09	137	26,9225	26,8712	26,9739	10	5	5	0,119366	0,102746	Q890K8	Q890K8	dnaA	Chromosomal replication initiator protein DnaA
28,7979	28,5585	28,3662	28,4536	28,4872	28,5183	28,8065	28,5665	28,7737	28,5169		3	3	3	17,6	17,6	17,6	21,584	4,58E+09	41	28,5845	28,5327	28,6364	10	5	5	0,315862	0,103716	F9UPL2	F9UPL2	lp_1884	Extracellular protein, with LysM peptidoglycan binding domain
27,4198	27,501	27,3991	27,4021	27,3575	27,5459	27,682	27,458	27,6093	27,3067		17	17	17	49,1	49,1	49,1	39,363	7,63E+09	131	27,4681	27,4159	27,5204	10	5	5	0,168682	0,104497	Q88WR3	Q88WR3	pheS	Phenylalanine--tRNA ligase alpha subunit
27,7376	27,7861	27,6346	27,7434	27,7371	27,7285	27,99	27,7526	27,9065	27,8001		11	11	11	44,2	44,2	44,2	23,935	5,42E+09	96	27,7816	27,7278	27,8355	10	5	5	0,0867426	0,107794	F9UMI1	F9UMI1	lp_0996	Metal-dependent regulator
24,8882	25,1768	25,2097	25,1054	24,6233	25,0356	24,7949	25,345	25,1713	25,201		14	14	14	15,2	15,2	15,2	132,21	1,43E+09	65	25,0551	25,0007	25,1096	10	5	5	0,470159	0,10891	F9UL13	F9UL13	mfd	Transcription-repair-coupling factor
25,8388	25,5222	25,7511	25,6012	25,6627	25,7199	25,6724	25,808	25,99	25,7306		4	4	4	13,3	13,3	13,3	39,126	1,26E+09	24	25,7297	25,6752	25,7842	10	5	5	0,203808	0,108955	F9URB3	F9URB3	lp_2606	NAD(P)-dependent oxidoreductase
25,1733	25,4952	25,5007	25,3824	25,1489	25,4222	25,4787	25,5243	25,4182	25,4025		10	10	10	19,1	19,1	19,1	84,479	1,59E+09	57	25,3947	25,3401	25,4492	10	5	5	0,207282	0,109071	F9UTJ5	F9UTJ5	pflB	Formate acetyltransferase
27,7732	27,9381	27,8959	27,9395	28,0273	27,8986	27,9688	28,069	28,1591	28,0243		18	18	18	57,9	57,9	57,9	46,672	1,17E+10	203	27,9694	27,9148	28,024	10	5	5	0,109012	0,109147	F9UL42	F9UL42	hom2	Homoserine dehydrogenase
25,7191	26,049	25,9893	25,8824	25,9103	25,7438	25,9076	26,0873	26,1158	26,2444		7	7	7	55,6	55,6	55,6	18,701	2,16E+09	50	25,9649	25,91	26,0198	10	5	5	0,321382	0,10979	F9URP0	F9URP0	lp_2770	Transcription regulator, biotin repressor family
27,8241	27,9415	27,8883	28,0791	27,9334	28,116	28,0697	27,9896	27,983	28,0576		3	3	3	11,6	11,6	11,6	25,9	3,43E+09	69	27,9882	27,9333	28,0432	10	5	5	0,0550772	0,109918	Q88W46	Q88W46	tarI	Ribitol-5-phosphate cytidylyltransferase
25,4524	25,4162	25,5653	25,703	25,5016	25,6312	25,6611	25,7216	25,6611	25,5225		7	7	7	25,9	25,9	25,9	50,301	9,30E+08	37	25,5836	25,5277	25,6395	10	5	5	0,100114	0,111813	F9UP82	F9UP82	lp_1724	23S rRNA methylase
25,8856	25,8883	26,0791	26,0802	25,9955	25,9763	25,9187	26,2325	26,0835	26,2807		7	7	7	34,4	34,4	34,4	26,757	1,57E+09	44	26,042	25,9858	26,0983	10	5	5	0,209211	0,11257	Q88T09	Q88T09	rsmG	Ribosomal RNA small subunit methyltransferase G
27,851	28,0953	28,0578	28,0134	27,9578	27,9008	28,0329	28,2749	28,2666	28,0633		16	16	16	31,2	31,2	31,2	57,936	9,14E+09	154	28,0514	27,995	28,1077	10	5	5	0,215075	0,112658	Q88W76	Q88W76	fhs	Formate--tetrahydrofolate ligase
26,7371	26,859	26,8502	26,7747	26,8116	26,7613	26,862	26,9785	27,1267	26,87		25	25	25	48,9	48,9	48,9	68,344	8,55E+09	205	26,8631	26,8065	26,9197	10	5	5	0,125913	0,113158	Q88VN0	Q88VN0	lepA1	Elongation factor 4 1
24,0714	24,1605	23,8937	24,3214	24,1612	24,0155	24,1206	24,1886	24,4079	24,4434		6	6	6	16,5	16,5	16,5	59,464	5,22E+08	32	24,1784	24,1216	24,2352	10	5	5	0,324683	0,113557	F9UMS6	F9UMS6	mleS	Malolactic enzyme
30,3171	30,475	30,415	30,3839	30,3356	30,3464	30,528	30,5245	30,6011	30,495		39	39	39	73,9	73,9	73,9	50,972	9,03E+10	711	30,4422	30,3853	30,499	10	5	5	0,055012	0,113657	F9UNV9	F9UNV9	glnA	Glutamine synthetase
25,4115	25,6376	25,7116	25,1918	25,3771	25,4023	25,5669	25,6304	25,6508	25,6489		4	4	4	18,7	18,7	18,7	27,699	8,62E+08	32	25,5229	25,4659	25,5799	10	5	5	0,308849	0,11393	F9UQ42	F9UQ42	fruR	Transcription regulator, DeoR family, fructose operon
27,9008	28,0623	28,1274	28,0406	28,1098	28,0657	28,0712	28,243	28,2762	28,1547		10	10	10	51,1	51,1	51,1	25,78	1,53E+10	232	28,1052	28,0482	28,1622	10	5	5	0,0887304	0,113995	F9UTA0	F9UTA0	rrp11	Two component system response regulator
26,6733	26,6536	26,5882	26,5224	26,4182	26,5546	26,6761	26,7544	26,8334	26,6096		9	9	9	26,2	26,2	26,2	43,404	3,79E+09	69	26,6284	26,5711	26,6856	10	5	5	0,131476	0,114497	F9UMN9	F9UMN9	apbE1	FAD:protein FMN transferase
25,8714	25,9584	25,9647	26,0119	25,9862	26,2176	26,2082	26,0773	26,0306	25,8359		6	6	6	44,4	44,4	44,4	27,757	1,62E+09	51	26,0162	25,9585	26,0739	10	5	5	0,155712	0,115395	F9URL3	F9URL3	lp_2739	ABC transporter, ATP-binding protein
28,9514	29,0534	29,155	29,0868	28,9548	29,0535	29,137	29,2776	29,1779	29,1338		49	49	49	58,5	58,5	58,5	101,53	4,43E+10	600	29,0981	29,0403	29,1559	10	5	5	0,0628032	0,11564	Q88UX7	Q88UX7	valS	Valine--tRNA ligase
28,4766	28,7334	28,7839	28,7415	28,8456	28,7588	28,9129	28,8455	28,9133	28,731		19	19	19	60	60	60	42,679	2,83E+10	291	28,7743	28,7162	28,8323	10	5	5	0,153735	0,116093	Q88XB8	Q88XB8	metK	S-adenosylmethionine synthase
30,3587	30,0144	29,8838	29,9916	29,9705	29,9818	30,1284	30,1703	30,282	30,2409		6	6	6	42,7	42,7	42,7	10,347	3,78E+10	252	30,1022	30,0438	30,1607	10	5	5	0,26232	0,116909	Q88XX7	Q88XX7	rpsQ	30S ribosomal protein S17
27,684	27,6237	27,8461	27,7858	27,8624	27,8067	27,7372	27,8858	27,954	28,0071		9	9	9	45,6	45,6	45,6	24,64	7,70E+09	186	27,8193	27,7604	27,8782	10	5	5	0,117792	0,117749	Q88VZ4	Q88VZ4	cmk	Cytidylate kinase
31,4948	31,5618	31,8458	31,8666	31,9136	31,8843	31,8145	31,7504	31,7812	32,0424		4	4	4	22,9	22,9	22,9	42,447	5,88E+10	485	31,7955	31,7365	31,8546	10	5	5	0,275271	0,118041	F9UP39	F9UP39	fabF	3-oxoacyl-[acyl-carrier-protein] synthase 2
28,0874	28,2857	28,3951	28,4054	28,3968	28,2228	28,4345	28,5177	28,5654	28,4207		10	10	10	25	25	25	62,061	4,26E+09	77	28,3732	28,3141	28,4322	10	5	5	0,199878	0,118134	F9UNS8	F9UNS8	hpk5	Signal transduction histidine-protein kinase ArlS
26,1861	26,4053	26,2769	26,3428	26,4663	26,3571	26,6249	26,3181	26,4975	26,4708		3	3	3	15,5	15,5	15,5	38,014	1,07E+09	18	26,3946	26,3355	26,4537	10	5	5	0,144815	0,118195	Q88WB6	Q88WB6	fni	Isopentenyl-diphosphate delta-isomerase
28,0911	28,2131	28,169	28,1324	28,2197	28,0129	28,2615	28,4538	28,4973	28,192		22	22	22	34,6	34,6	34,6	71,908	1,13E+10	160	28,2243	28,1651	28,2835	10	5	5	0,233132	0,118433	F9UMN7	F9UMN7	ndh2	NADH dehydrogenase, membrane-anchored
29,0154	28,9255	28,9572	28,9898	29,0989	29,2183	29,2229	28,9897	29,1502	28,9981		10	10	10	45,2	45,2	45,2	32,448	9,52E+09	159	29,0566	28,9974	29,1158	10	5	5	0,0808575	0,118488	F9UQ43	F9UQ43	fruK	Tagatose-6-phosphate kinase

24,9328	24,7872	24,6206	24,4431	24,2154	24,7064	25,0532	24,7557	24,8518	24,2248		4	4	4	34,9	34,9	34,9	24,054	6,30E+08	22	24,6591	24,5998	24,7184	10	5	5	0,542326	0,118521	Q88WR0	Q88WR0	udk	Uridine kinase
25,5992	25,6647	25,5964	25,6744	25,7655	25,6147	25,7861	25,8648	25,8725	25,7582		18	18	18	29,1	29,1	29,1	82,226	3,28E+09	122	25,7197	25,66	25,7793	10	5	5	0,0657719	0,119255	Q88YD2	Q88YD2	ppk	Polyphosphate kinase
27,7073	27,9454	27,9693	27,9261	28,0377	27,8302	27,9915	28,1116	28,2223	28,0286		30	30	30	50,3	50,3	50,3	58,161	2,03E+10	345	27,977	27,9172	28,0368	10	5	5	0,200229	0,11968	Q88UZ5	Q88UZ5	rny	Ribonuclease Y
29,767	30,0217	30,0657	30,0033	30,0723	29,9382	30,1007	30,1323	30,23	30,1303		39	39	39	67,3	67,3	67,3	56,918	8,84E+10	872	30,0462	29,986	30,1063	10	5	5	0,140531	0,120297	Q88YY0	Q88YY0	gltX	Glutamate--tRNA ligase
24,5724	24,6683	24,641	24,789	24,9083	24,8211	25,0953	24,5762	24,8158	24,8745		2	2	2	8,7	8,7	8,7	32,054	3,82E+08	20	24,7762	24,7158	24,8366	10	5	5	0,270018	0,120756	F9UMB3	F9UMB3	lp_0912	Enoyl-[acyl-carrier protein] reductase (NADH)
25,5188	25,6806	25,5657	25,6931	25,671	25,4725	25,7954	25,6085	26,0454	25,8123		4	4	4	18,7	18,7	18,7	20,647	1,09E+09	30	25,6863	25,6258	25,7468	10	5	5	0,276758	0,120975	F9UQ78	F9UQ78	lp_2134	N6-adenine-specific methylase
26,3799	26,3947	26,3731	26,4064	26,4299	26,3928	26,5838	26,6303	26,6352	26,3526		5	5	5	45,9	45,9	45,9	13,96	2,51E+09	61	26,4579	26,3968	26,5189	10	5	5	0,0824177	0,122153	F9US72	F9US72	lp_0052	6-carboxy-5,6,7,8-tetrahydropterin synthase
32,5581	32,6098	32,7189	32,5817	31,7748	32,6238	32,1064	32,7024	32,6048	32,8189		66	66	66	64,5	64,5	64,5	134,5	1,55E+11	1052	32,51	32,4486	32,5713	10	5	5	0,575221	0,122614	Q88XZ3	Q88XZ3	rpoB	DNA-directed RNA polymerase subunit beta
27,1715	27,3421	27,3075	27,3513	27,4192	27,2993	27,4521	27,4501	27,5622	27,4446		11	11	11	40,2	40,2	40,2	40,389	4,93E+09	88	27,38	27,3183	27,4417	10	5	5	0,0680799	0,123361	F9UPC4	F9UPC4	lp_1776	Oxidoreductase, FAD-dependent
25,2141	25,4322	25,3951	25,3271	25,2981	25,2317	25,4223	25,5091	25,693	25,4456		8	8	8	21,3	21,3	21,3	52,273	1,39E+09	57	25,3968	25,3333	25,4603	10	5	5	0,166933	0,127003	F9UKV9	F9UKV9	lp_0476	Metal-dependent phosphohydrolase, HD family
25,8803	25,9794	25,9269	25,8193	26,104	25,9702	26,1011	26,1314	26,1494	25,9935		14	14	14	31,3	31,3	31,3	71,368	3,02E+09	94	26,0055	25,942	26,0691	10	5	5	0,0693785	0,127135	F9UPK1	F9UPK1	lp_1871	ABC transporter, ATP-binding protein, ChvD family
28,8801	29,162	29,1091	29,0916	29,1479	29,2982	29,347	29,1353	29,1401	29,1067		21	21	21	84,8	84,8	84,8	31,891	3,69E+10	374	29,1418	29,0781	29,2055	10	5	5	0,109148	0,127355	F9USV8	F9USV8	lp_0137	Aldo/keto reductase family protein
27,3822	27,7287	27,7938	27,7419	27,7284	27,7267	27,7253	27,8122	27,8777	27,8709		7	7	7	24,8	24,8	24,8	37,108	5,36E+09	81	27,7388	27,675	27,8025	10	5	5	0,155281	0,127555	F9UMW1	F9UMW1	lp_1161	Oxidoreductase, NAD-dependent
25,1693	25,3719	25,4668	25,394	25,6889	25,3716	25,5717	25,5614	25,6388	25,5881		9	9	9	25,3	25,3	25,3	50,019	2,05E+09	83	25,4822	25,4182	25,5463	10	5	5	0,216106	0,128136	F9UL72	F9UL72	radA	DNA repair protein RadA
26,0984	26,2364	25,8988	26,0156	26,0379	26,146	26,2345	26,1039	26,2383	26,2069		8	8	8	53	53	53	15,474	1,49E+09	51	26,1217	26,0574	26,1859	10	5	5	0,0689227	0,128497	F9UN40	F9UN40	tagD2	Glycerol-3-phosphate cytidylyltransferase
29,3624	29,4153	29,4022	29,3751	29,3212	29,3245	29,4784	29,5444	29,6555	29,5178		15	15	15	80,7	80,7	80,7	20,021	4,20E+10	366	29,4397	29,3752	29,5041	10	5	5	0,0508584	0,128859	Q88UU0	Q88UU0	atpH	ATP synthase subunit delta
28,1768	28,2975	28,1608	28,0114	28,1131	28,1789	28,357	28,2233	28,4331	28,2148		3	3	3	33,1	33,1	33,1	15,047	7,19E+09	67	28,2167	28,1519	28,2814	10	5	5	0,0899125	0,129518	F9US20	F9US20	def2	Formylmethionine deformylase
28,9837	29,24	29,1535	29,0851	29,071	29,0926	29,225	29,2775	29,3631	29,225		7	7	7	18,7	18,7	18,7	41,174	2,18E+10	161	29,1716	29,1067	29,2366	10	5	5	0,0672987	0,129969	F9UQH7	F9UQH7	pepQ	Xaa-Pro dipeptidase
25,5917	25,8017	25,5183	25,6877	25,8859	25,8956	25,799	25,9272	25,5122	26,0012		4	4	4	26,6	26,6	26,6	21,665	9,38E+08	18	25,7621	25,6971	25,827	10	5	5	0,264552	0,129983	Q88V02	Q88V02	ruvA	Holliday junction ATP-dependent DNA helicase RuvA
30,5386	30,7611	30,766	30,7688	30,7528	30,7623	30,9756	30,7629	30,9151	30,8238		13	13	13	58,7	58,7	58,7	21,529	5,85E+10	328	30,7827	30,7175	30,8479	10	5	5	0,0672862	0,130478	Q88YH9	Q88YH9	clpP	ATP-dependent Clp protease proteolytic subunit
25,4217	25,4243	25,6164	25,2944	25,3372	25,5138	25,4713	25,4645	25,7439	25,5608		10	10	10	23	23	23	51,867	1,80E+09	53	25,4848	25,4188	25,5508	10	5	5	0,118113	0,132043	F9UNP9	F9UNP9	dnaB	Replication initiation and membrane attachment protein DnaB
26,1642	26,4501	26,3434	26,3375	26,4769	26,4231	26,3991	26,4801	26,5492	26,581		2	2	2	10,9	10,9	10,9	24,818	1,43E+09	40	26,4205	26,3544	26,4865	10	5	5	0,0778058	0,13209	F9UQQ3	F9UQQ3	lp_2351	D-Methionine-like ABC transporter,permease protein
26,9526	27,177	27,1676	26,9364	27,043	27,2184	27,2703	27,0681	27,2691	27,1175		7	7	7	47,9	47,9	47,9	16,023	4,18E+09	72	27,122	27,0553	27,1887	10	5	5	0,0762181	0,133353	Q88V13	Q88V13	lp_2274	Putative pre-16S rRNA nuclease
24,8673	25,0278	24,9402	24,8849	24,9753	25,087	24,8383	25,0934	25,2643	25,09		6	6	6	27,1	27,1	27,1	27,111	6,12E+08	40	25,0068	24,9391	25,0746	10	5	5	0,104871	0,135516	F9ULZ1	F9ULZ1	lp_0765	Diguanylate cyclase/phosphodiesterase, EAL domain
28,429	28,6836	28,6844	28,7262	28,7323	28,5121	28,798	28,8446	28,9592	28,821		19	19	19	58,6	58,6	58,6	53,81	2,44E+10	265	28,719	28,6511	28,787	10	5	5	0,182809	0,135872	Q88Y23	Q88Y23	murE	UDP-N-acetylmuramoyl-L-alanyl-D-glutamate--2,6-diaminopimelate ligase
24,4994	24,6379	24,808	24,4295	24,5739	24,7994	24,7916	24,5775	24,7081	24,7522		5	5	5	24,1	24,1	24,1	40,512	4,69E+08	28	24,6578	24,5898	24,7258	10	5	5	0,113027	0,136	F9URD2	F9URD2	cgs	Cysteine gamma synthase/O-succinylhomoserine (Thiol)-lyase
25,7505	25,6613	25,6896	25,7022	25,7353	25,7385	26,023	25,8907	25,953	25,6211		8	8	8	18,5	18,5	18,5	74,575	1,49E+09	53	25,7765	25,7078	25,8452	10	5	5	0,103573	0,137448	F9UNZ3	F9UNZ3	pkn1	Non-specific serine/threonine protein kinase
25,556	25,6879	25,7782	25,7149	25,6792	25,947	25,7218	25,9104	25,8496	25,6772		6	6	6	32	32	32	27,375	9,40E+08	13	25,7522	25,6832	25,8212	10	5	5	0,0626097	0,137966	F9UP96	F9UP96	dnaD	DNA replication protein DnaD
30,9251	31,0321	30,9835	31,0246	30,8322	31,2123	31,2121	30,9975	31,115	30,9512		12	12	12	65,9	65,9	65,9	17,926	8,36E+10	284	31,0286	30,9595	31,0976	10	5	5	0,0676234	0,138107	Q88YW8	Q88YW8	rplJ	50S ribosomal protein L10
31,2064	31,4551	31,2356	31,3722	31,4272	31,2266	31,4364	31,5373	31,6053	31,5819		5	5	5	31,4	31,4	31,4	13,59	5,93E+10	393	31,4084	31,3393	31,4775	10	5	5	0,144517	0,138191	Q88WU6	Q88WU6	rplT	50S ribosomal protein L20
28,6645	28,8617	28,91	28,8328	28,805	28,807	29,0677	28,964	29,0602	28,8664		15	15	15	43,6	43,6	43,6	52,36	1,47E+10	171	28,8839	28,8148	28,953	10	5	5	0,0705354	0,138218	F9UMF3	F9UMF3	pepD3	Dipeptidase
25,649	25,6628	25,8029	25,9056	25,7852	25,8847	25,8434	25,9659	25,672	26,1344		5	5	5	24,6	24,6	24,6	26,375	8,71E+08	17	25,8306	25,7611	25,9001	10	5	5	0,15901	0,138976	F9URB8	F9URB8	lp_2614	ABC transporter, ATP-binding protein
28,1494	28,3542	28,2964	28,3427	28,4629	28,2679	28,5564	28,4723	28,5712	28,4406		12	12	12	33,3	33,3	33,3	48,777	9,53E+09	133	28,3914	28,3211	28,4617	10	5	5	0,095691	0,14055	Q88VZ6	Q88VZ6	der	GTPase Der
25,1704	25,4587	25,4875	25,4701	25,6529	25,4785	25,6639	25,607	25,6924	25,5031		4	4	4	19,8	19,8	19,8	28,725	7,29E+08	25	25,5184	25,4479	25,589	10	5	5	0,150465	0,141073	F9UQG0	F9UQG0	lp_2235	Acid sugar phosphatase
25,5119	25,5416	25,6501	25,8952	25,9203	25,6492	25,7932	25,9036	25,8456	26,0329		2	2	2	9,3	9,3	9,3	32,813	6,33E+08	20	25,7744	25,7038	25,8449	10	5	5	0,22409	0,141085	F9UM32	F9UM32	lp_0813	Oxidoreductase, NAD-dependent
24,6478	24,5428	24,5733	24,7044	24,086	24,5877	24,3718	24,7728	24,722	24,8083		16	16	16	15,9	15,9	15,9	131,88	1,18E+09	63	24,5817	24,5109	24,6525	10	5	5	0,326946	0,141669	Q88WJ9	Q88WJ9	smc	Chromosome partition protein Smc
25,2663	25,6522	25,4632	25,4149	25,4268	25,4122	25,6066	25,7029	25,7164	25,4992		6	6	6	50	50	50	16,31	1,39E+09	35	25,5161	25,4447	25,5874	10	5	5	0,132348	0,142794	F9UM52	F9UM52	spx1	RNA polymerase (RNAP)-binding regulatory protein, arsenate reductase (ArsC) family, Spx subfamily
25,6686	25,4718	25,7549	25,6196	25,7048	25,8331	25,8875	25,8073	25,7597	25,6486		5	5	5	11,9	11,9	11,9	48,77	1,08E+09	37	25,7156	25,6439	25,7873	10	5	5	0,0525331	0,143325	F9UMS2	F9UMS2	lp_1113	Fumarate reductase, flavoprotein subunit
26,4411	26,7432	26,6373	26,6468	26,6889	26,5889	26,7922	26,8595	26,9397	26,6944		14	14	14	32	32	32	54,569	6,13E+09	161	26,7032	26,6314	26,7749	10	5	5	0,110534	0,143505	F9URM9	F9URM9	thrC	Threonine synthase
28,6554	28,676	28,6647	28,6445	28,5235	28,6088	28,9044	28,8578	28,9907	28,5211		6	6	6	21,1	21,1	21,1	35,541	9,59E+09	112	28,7047	28,6328	28,7766	10	5	5	0,165793	0,143719	F9UQ24	F9UQ24	lp_2071	Glycosyltransferase, family 2
26,0154	26,0519	25,853	26,2964	26,1085	26,0745	26,287	26,2623	26,2314	26,1962		5	5	5	27,4	27,4	27,4	26,075	1,69E+09	28	26,1377	26,065	26,2103	10	5	5	0,110077	0,145247	Q88YG1	Q88YG1	ung	Uracil-DNA glycosylase
28,0353	28,3167	28,2155	28,3713	28,4334	28,3134	28,4146	28,3342	28,6186	28,4198		11	11	11	33,9	33,9	33,9	38,704	9,34E+09	153	28,3473	28,2744	28,4201	10	5	5	0,136834	0,14569	Q88V81	Q88V81	murG	UDP-N-acetylglucosamine--N-acetylmuramyl-(pentapeptide) pyrophosphoryl-undecaprenol N-acetylglucosamine transferase
25,7604	25,9465	25,8168	25,6529	25,3947	25,7128	25,769	25,9181	26,0182	25,8858		2	2	2	8	8	8	25,367	8,05E+08	14	25,7875	25,7142	25,8608	10	5	5	0,210244	0,14654	F9UMX3	F9UMX3	glpF4	Glycerol uptake facilitator protein 4
29,6142	29,7888	29,8043	29,7752	29,9227	29,7807	29,9482	29,9843	30,0517	29,8818		27	27	26	56	56	56	49,867	6,12E+10	602	29,8552	29,781	29,9293	10	5	5	0,0593242	0,148336	Q88WA8	Q88WA8	asnS2	Asparagine--tRNA ligase 2
25,3924	25,1327	25,4111	25,166	25,3626	25,1394	25,4332	25,5685	25,4775	25,5902		6	6	6	9,6	9,6	9,6	83,017	1,22E+09	39	25,3674	25,2929	25,4418	10	5	5	0,176308	0,148823	Q88UW5	Q88UW5	gshAB	Glutathione biosynthesis bifunctional protein GshAB
24,7745	24,4643	24,8798	24,7064	24,5524	25,0065	24,5673	24,7611	24,9608	24,8303		4	4	4	17,2	17,2	17,2	29,658	6,24E+08	30	24,7503	24,6755	24,8252	10	5	5	0,203899	0,149687	F9UNB7	F9UNB7	lp_1353	Hydrolase, HAD superfamily, Cof family
24,9701	24,8545	24,7862	24,5699	24,4241	24,8519	24,7279	24,8054	25,0898	24,8797		2	2	2	12	12	12	21,459	5,15E+08	15	24,7959	24,721	24,8709	10	5	5	0,231399	0,149991	Q6LWH0	Q6LWH0	tnpR1	Putative resolvase
25,3206	25,6348	25,5381	25,6708	25,8584	25,6951	25,7124	25,7232	25,8436	25,8052		3	3	3	22	22	22	18,677	7,30E+08	21	25,6802	25,6045	25,7559	10	5	5	0,140797	0,151376	F9UPN4	F9UPN4	lp_1911	Transcription regulator, MarR family
25,8823	25,8543	25,8666	25,9086	25,9868	25,5682	26,2203	26,0194	26,2861	26,1652		9	9	9	23,7	23,7	23,7	51,126	1,40E+09	55	25,9758	25,8997	26,0519	10	5	5	0,278273	0,152146	F9UL10	F9UL10	lp_0536	Cell surface protein, ErfK/YbiS/YcfS/YnhG family
26,5928	26,7987	26,7593	26,7316	26,5543	26,6799	26,8059	26,864	27,017	26,8449		8	8	8	54,2	54,2	54,2	17,614	3,90E+09	87	26,7648	26,6873	26,8423	10	5	5	0,0647166	0,155007	Q88XZ6	Q88XZ6	ctsR	Transcriptional regulator CtsR
30,6612	31,086	31,042	31,0121	30,9759	30,9192	30,9794	31,1495	31,2862	31,234		4	4	4	32,6	32,6	32,6	13,778	4,64E+10	186	31,0345	30,9554	31,1137	10	5	5	0,166484	0,158228	Q88XW1	Q88XW1	rpsK	30S ribosomal protein S11
27,1457	27,2008	27,2292	27,1863	27,3093	27,1426	27,3549	27,5091	27,5721	27,2904		16	16	16	30,2	30,2	30,2	56,759	8,01E+09	161	27,294	27,2143	27,3738	10	5	5	0,0864544	0,159553	Q6LWF1	Q6LWF1	traK	Conjugation protein
25,3104	25,2353	25,2651	25,2989	25,3363	25,1397	25,2865	25,4757	25,8145	25,5295		11	11	11	53,8	53,8	53,8	26,621	1,22E+09	40	25,3692	25,2892	25,4492	10	5	5	0,205191	0,159974	F9UPL6	F9UPL6	rluB	Pseudouridine synthase
26,9431	27,2353	27,1285	27,1815	26,9162	27,1574	27,201	27,1815	27,3621	27,3027		7	7	7	23,5	23,5	23,5	44,923	2,28E+09	52	27,1609	27,0809	27,2409	10	5	5	0,0658868	0,160009	F9UNL0	F9UNL0	sufS	Cysteine desulfurase
26,1009	26,2353	26,1199	26,2154	26,2225	26,5506	26,4627	26,1778	26,415	26,0985		4	4	4	27,8	27,8	27,8	27,258	2,33E+09	50	26,2599	26,1788	26,3409	10	5	5	0,112653	0,162151	F9UNZ2	F9UNZ2	pppL	Serine/threonine specific protein phosphatase
26,01	25,9953	26,1358	25,986	26,2724	26,1188	26,1087	26,3484	26,3721	26,2779		11	11	11	25,5	25,5	25,5	50,117	2,77E+09	99	26,1625	26,0799	26,2452	10	5	5	0,0685941	0,165267	F9UNZ1	F9UNZ1	sunL	16S rRNA m5C967 methyltransferase
26,4231	26,2558	26,661	26,5693	26,5022	26,3425	26,6851	26,7398	26,7169	26,7574		5	5	5	15,6	15,6	15,6	36,644	2,27E+09	32	26,5653	26,4823	26,6483	10	5	5	0,147589	0,166036	F9US21	F9US21	apbE2	FAD:protein FMN transferase
26,3221	26,3472	26,2218	26,0089	26,1224	26,5681	26,6676	26,3171	26,2474	26,0578		9	9	9	71,2	71,2	71,2	19,524	2,52E+09	57	26,2881	26,2045	26,3716	10	5	5	0,224645	0,167122	F9UQD6	F9UQD6	cspR	Putative tRNA (cytidine(34)-2-O)-methyltransferase
25,3689	25,6325	25,4409	25,7521	25,8998	25,5109	25,7865	25,9876	25,7029	25,9418		6	6	6	48,3	48,3	48,3	16,741	1,04E+09	26	25,7024	25,6188	25,786	10	5	5	0,234849	0,167139	F9ULD4	F9ULD4	spx5	RNA polymerase (RNAP)-binding regulatory protein, arsenate reductase (ArsC) family, Spx subfamily
26,8561	27,1626	26,8862	26,882	26,9182	26,571	27,2508	27,2268	27,4156	27,0785		2	2	2	27,8	27,8	27,8	10,113	1,78E+09	31	27,0248	26,941	27,1085	10	5	5	0,311876	0,167507	Q88WR7	Q88WR7	acyP	Acylphosphatase
27,7302	28,0687	27,9822	27,8838	28,0909	27,8899	28,1595	28,1442	28,3575	28,0597		11	11	11	32,2	32,2	32,2	34,401	6,95E+09	146	28,0367	27,9512	28,1222	10	5	5	0,127946	0,170977	Q88WL3	Q88WL3	fmt	Methionyl-tRNA formyltransferase
27,56	27,8394	27,8627	27,7457	27,7206	27,6668	27,8542	27,9808	28,2091	27,8806		11	11	11	30,7	30,7	30,7	50,137	1,34E+10	201	27,832	27,7457	27,9183	10	5	5	0,134427	0,172606	Q88V80	Q88V80	murD	UDP-N-acetylmuramoylalanine--D-glutamate ligase
26,5374	26,7075	26,7964	26,811	26,8547	26,7062	26,9532	27,0051	26,991	26,9148		7	7	7	24,6	24,6	24,6	44,332	2,90E+09	74	26,8277	26,7414	26,914	10	5	5	0,0583812	0,172635	F9UL73	F9UL73	lp_0607	ATPase, PilT family
27,0693	27,0949	27,0742	27,111	26,9439	27,0201	27,1265	27,3122	27,4543	27,2493		13	13	12	58	58	54,6	23,316	5,36E+09	101	27,1456	27,0586	27,2325	10	5	5	0,0629766	0,173829	F9URW3	F9URW3	sip1	Signal peptidase I
24,97	25,2732	25,0281	25,1138	24,9415	25,2557	25,4336	25,1383	25,2011	25,1752		3	3	3	22,3	22,3	22,3	21,692	7,39E+08	29	25,1531	25,0653	25,2408	10	5	5	0,0573581	0,17546	F9UM77	F9UM77	gmk2	Guanylate kinase
26,5253	26,7251	26,6276	26,7574	26,7595	26,6786	26,8431	26,8291	27,0688	26,8612		3	3	3	15	15	15	29,583	1,58E+09	21	26,7676	26,679	26,8561	10	5	5	0,0504642	0,177193	Q88WM8	Q88WM8	folD	Bifunctional protein FolD
25,0276	25,1838	25,1617	24,8764	25,0614	24,9418	25,1974	25,3279	25,4623	25,3071		4	4	4	16,8	16,8	16,8	32,824	6,60E+08	33	25,1547	25,0622	25,2473	10	5	5	0,110305	0,185086	F9UQI1	F9UQI1	lp_2262	Mechanosensitive ion channel family protein
25,979	26,1474	26,2585	26,1336	25,6117	26,2141	26,0216	26,3394	26,2376	26,2608		14	14	14	16,7	16,7	16,7	99,979	2,60E+09	94	26,1204	26,026	26,2147	10	5	5	0,16798	0,188658	Q88UZ7	Q88UZ7	mutS	DNA mismatch repair protein MutS
25,7383	25,8087	25,8558	25,4711	25,8788	25,7353	25,9119	26,0414	26,0082	26,0078		4	4	4	26,3	26,3	26,3	21,313	1,19E+09	27	25,8457	25,7505	25,9409	10	5	5	0,0737096	0,19041	F9UU88	F9UU88	lp_3409	Transcription regulator, TetR family
25,1413	25,2819	25,3312	25,3932	25,3209	25,211	25,4047	25,7208	25,7254	25,364		4	4	4	10,5	10,5	10,5	43,426	1,10E+09	35	25,3894	25,2937	25,4852	10	5	5	0,121907	0,191459	F9UNY8	F9UNY8	coaBC	Coenzyme A biosynthesis bifunctional protein CoaBC
24,7191	24,7471	25,0173	24,8184	25,0393	24,886	24,9235	25,1228	25,2603	25,1067		3	3	3	23,3	23,3	23,3	23,398	3,85E+08	9	24,964	24,8682	25,0599	10	5	5	0,0821797	0,191619	F9UQG3	F9UQG3	lp_2238	Metallophosphatase
27,292	27,5198	27,7023	27,5527	27,3757	27,4303	27,6404	27,7521	27,865	27,7336		7	7	7	28,9	28,9	28,9	32,88	3,40E+09	56	27,5864	27,4885	27,6843	10	5	5	0,0912496	0,195817	F9ULU2	F9ULU2	rsmI	Ribosomal RNA small subunit methyltransferase I
25,5756	25,7491	25,9815	25,8898	25,5226	25,8714	26,0218	25,8346	26,0732	25,9013		2	2	2	8	8	8	29,909	6,00E+08	14	25,8421	25,7437	25,9405	10	5	5	0,0826018	0,196764	F9UMD0	F9UMD0	lp_0931	Fumarylacetoacetate hydrolase family protein
25,3413	25,148	25,1304	24,9892	25,0864	25,0326	25,2265	25,3455	25,6015	25,4883		4	4	4	15,6	15,6	15,6	33,867	6,96E+08	28	25,239	25,1391	25,3389	10	5	5	0,120215	0,199838	F9UPC9	F9UPC9	rluD1	Pseudouridine synthase
27,4792	27,6965	27,609	27,6	27,6154	27,5381	27,7886	27,8444	28,0615	27,7764		10	10	10	28,4	28,4	28,4	40,15	5,29E+09	108	27,7009	27,6	27,8018	10	5	5	0,0563624	0,201776	F9UND1	F9UND1	lp_1371	Extracellular protein, membrane-anchored
24,9077	25,2387	24,9759	25,2536	25,2137	25,1201	25,1699	25,4425	25,5814	25,2868		14	14	14	32,7	32,7	32,7	49,656	1,60E+09	86	25,219	25,1179	25,3201	10	5	5	0,110163	0,202215	F9UMW2	F9UMW2	rhe2	ATP-dependent RNA helicase
24,6989	24,5649	24,8363	24,7239	24,8966	24,8379	25,0502	24,905	25,2612	24,7265		2	2	2	9,3	9,3	9,3	32,95	5,79E+08	17	24,8502	24,7441	24,9562	10	5	5	0,0875746	0,212047	F9URJ0	F9URJ0	lp_2713	NmrA domain-containing protein
26,3937	26,8191	26,7314	26,7136	26,7814	26,5675	26,8303	26,9654	27,1452	26,9947		4	4	4	18,4	18,4	18,4	34,259	2,83E+09	52	26,7942	26,6878	26,9006	10	5	5	0,122613	0,212776	F9URQ7	F9URQ7	lp_2790	2-hydroxyacid dehydrogenase
26,9326	26,6734	26,4985	26,5695	26,5992	26,797	26,9978	26,6703	26,9328	26,9441		7	7	7	21,1	21,1	21,1	44,62	2,28E+09	49	26,7615	26,6546	26,8684	10	5	5	0,0560189	0,213765	Q890J4	Q890J4	proA	Gamma-glutamyl phosphate reductase
27,9796	28,2505	28,3563	28,2751	28,216	28,0315	28,3388	28,5621	28,5994	28,6194		20	20	20	61,3	61,3	61,3	34,284	8,46E+09	148	28,3229	28,2155	28,4302	10	5	5	0,132844	0,214716	Q88VS0	Q88VS0	era	GTPase Era
24,971	25,0875	25,242	25,1936	24,3689	25,292	24,493	25,5067	25,2985	25,3477		18	18	18	16,8	16,8	16,8	162,04	2,03E+09	69	25,0801	24,9726	25,1876	10	5	5	0,392633	0,214985	Q88VK2	Q88VK2	polC	DNA polymerase III PolC-type
24,8495	25,0942	24,6724	24,3581	24,6278	24,9415	25,0626	24,905	24,9294	24,9012		3	3	3	11,3	11,3	11,3	32,472	4,06E+08	23	24,8342	24,7204	24,9479	10	5	5	0,107898	0,227534	F9UN48	F9UN48	lp_1258	Transcription regulator, ysR family
24,3743	24,876	24,6757	24,5559	24,7056	24,7533	24,6367	24,9477	25,0587	24,93		4	4	4	17,2	17,2	17,2	34,136	6,85E+08	29	24,7514	24,6375	24,8653	10	5	5	0,0769927	0,227768	F9USL7	F9USL7	fhuD	Iron chelatin ABC transporter, substrate binding protein
26,4525	26,6067	26,6709	26,5942	26,462	26,4864	26,6449	27,0045	27,0795	26,7111		12	12	12	28,3	28,3	28,3	53,847	2,83E+09	68	26,6713	26,5572	26,7853	10	5	5	0,0929515	0,228035	F9UQ58	F9UQ58	glnPH2	Glutamine ABC transporter, substrate binding and permease protein
25,1479	25,4177	25,6087	25,6728	25,3269	25,5912	25,6012	25,7161	25,7429	25,6698		9	9	9	35,8	35,8	35,8	30,892	1,87E+09	54	25,5495	25,4348	25,6643	10	5	5	0,0506363	0,229432	Q88WV4	Q88WV4	mutM	Formamidopyrimidine-DNA glycosylase
24,3475	24,5466	24,321	24,361	24,3366	24,3962	24,502	24,9196	24,8999	24,3582		3	3	3	4,9	4,9	4,9	95,883	3,24E+08	17	24,4989	24,3825	24,6152	10	5	5	0,109963	0,232663	F9UNH9	F9UNH9	lp_1435	Hypothetical membrane protein
25,2708	25,3418	25,6601	25,5537	25,6891	25,6819	25,4734	25,9524	25,991	25,6196		3	3	3	20,4	20,4	20,4	27,628	1,17E+09	20	25,6234	25,5031	25,7437	10	5	5	0,1017	0,240588	F9UNT5	F9UNT5	lp_1555	rRNA methylase, SpoU family
26,0992	26,0247	26,3383	26,206	26,1985	25,9145	26,2221	26,7414	26,6951	26,5092		4	4	4	9,4	9,4	9,4	53,901	1,15E+09	22	26,2949	26,1733	26,4165	10	5	5	0,17654	0,243121	F9UL24	F9UL24	dtpT	Di-/tripeptide transport protein
24,4723	24,5706	24,4855	24,3072	24,0352	24,4712	24,546	24,5133	24,8927	24,6848		5	5	5	18	18	18	41,074	4,44E+08	19	24,4979	24,3742	24,6216	10	5	5	0,07699	0,247449	F9UUH6	F9UUH6	lp_3510	PTS-associated protein
24,5914	24,3876	24,9006	24,8847	24,8974	24,7794	24,9367	25,0352	25,0394	25,127		5	5	5	9	9	9	75,703	5,92E+08	24	24,8579	24,7323	24,9835	10	5	5	0,0695624	0,25119	Q88SV0	Q88SV0	kup2	Probable potassium transport system protein kup 2
28,6091	28,6357	28,7261	28,5807	28,6215	28,4935	28,6597	29,0835	29,3325	28,8829		8	8	8	32,2	32,2	32,2	23,213	1,35E+10	108	28,7625	28,6347	28,8904	10	5	5	0,128629	0,255753	F9UNB8	F9UNB8	lp_1354	Uncharacterized protein
24,9336	25,2422	25,3271	24,8819	25,0784	25,2725	25,2396	25,5075	25,5441	25,1817		6	6	6	18,9	18,9	18,9	37,185	8,82E+08	33	25,2209	25,0927	25,3491	10	5	5	0,0532593	0,256422	F9ULU0	F9ULU0	holB	DNA-directed DNA polymerase III, delta subunit
25,8096	25,8495	25,6235	25,8229	26,1367	25,8925	26,1956	25,9635	26,3309	26,1533		6	6	6	18,3	18,3	18,3	38,129	1,25E+09	41	25,9778	25,8484	26,1072	10	5	5	0,0539097	0,258712	F9URT0	F9URT0	lp_2822	ABC transporter, permease protein
25,9311	26,0574	25,8825	26,1593	25,5949	26,2972	26,3287	25,9352	26,305	26,0813		3	3	3	26,6	26,6	26,6	23,876	1,29E+09	32	26,0573	25,925	26,1895	10	5	5	0,0642417	0,264446	F9UTV2	F9UTV2	rpiA2	Ribose-5-phosphate isomerase
26,5918	26,5374	26,6943	26,5078	26,0642	26,7347	26,5151	26,8825	26,704	26,9032		43	43	43	51,1	51,1	51,1	104,98	1,09E+10	329	26,6135	26,4791	26,7479	10	5	5	0,0711131	0,268781	Q88YI7	Q88YI7	uvrA	UvrABC system protein A
26,0408	26,2997	26,4267	26,018	25,9861	26,0657	26,3205	26,7179	26,8495	26,3347		8	8	8	29,1	29,1	29,1	32,04	1,79E+09	59	26,306	26,1543	26,4577	10	5	5	0,108417	0,303391	F9ULK7	F9ULK7	rbsK1	Ribokinase
24,3854	24,5994	24,5725	24,5534	24,6968	24,3736	24,598	25,3872	25,2132	24,9113		7	7	7	16,1	16,1	16,1	64,675	5,60E+08	28	24,7291	24,5615	24,8966	10	5	5	0,122764	0,335128	F9UNE0	F9UNE0	astA	Arylsulfate sulfotransferase
26,347	26,4374	26,2624	26,6346	26,7907	26,3451	26,7651	27,0777	27,2998	26,7634		3	3	3	8,7	8,7	8,7	44,021	1,16E+09	16	26,6723	26,4944	26,8502	10	5	5	0,0956566	0,355801	F9ULX0	F9ULX0	lp_0742	Hypothetical membrane protein
25,0892	25,4387	25,607	25,3352	25,0076	24,9245	25,5991	25,993	26,1327	25,637		3	3	3	17,6	17,6	17,6	30,075	1,11E+09	30	25,4764	25,2955	25,6573	10	5	5	0,165533	0,361759	F9UM80	F9UM80	lp_0871	Hypothetical membrane protein
27,9704	28,1923	27,5239	27,9909	27,9724	27,3709	28,1153	28,6302	28,6911	28,6735		5	5	5	53,8	53,8	53,8	8,8144	8,72E+09	98	28,1131	27,93	28,2962	10	5	5	0,223332	0,366252	Q88VI8	Q88VI8	lp_2061	UPF0154 protein lp_2061
28,4896	28,8132	28,4501	28,2732	28,3402	27,9837	28,7991	29,1132	29,3009	29,0921		5	5	5	65,3	65,3	65,3	6,0839	6,62E+09	50	28,6655	28,4733	28,8578	10	5	5	0,163763	0,38453	F9UNU8	F9UNU8	rpmG	50S ribosomal protein L33
26,0571	26,8261	26,0094	26,5909	26,063	25,6139	26,3329	27,0551	26,922	27,5483		5	5	5	30,1	30,1	30,1	7,9869	2,49E+09	50	26,5019	26,3093	26,6944	10	5	5	0,331033	0,385098	Q88WD3	Q88WD3	lp_1708	UPF0337 protein lp_1708
27,2772	27,9452	27,4573	27,8067	27,7242	27,2172	27,8868	28,4084	28,2871	28,3858		3	3	3	32,8	32,8	32,8	6,4433	2,63E+09	31	27,8396	27,6421	28,0371	10	5	5	0,161331	0,394933	F9UNV5	F9UNV5	lp_1577	Hypothetical extracellular protein
24,7047	25,2599	24,6796	25,1251	25,2615	24,9077	25,336	25,3631	25,6815	25,7525		3	3	3	31,5	31,5	31,5	10,295	5,24E+08	30	25,2072	25,0062	25,4082	10	5	5	0,0780779	0,402005	Q88V12	Q88V12	lp_2275	UPF0297 protein lp_2275
26,1658	26,3851	25,4644	25,9713	25,7024	25,7	26,1753	26,8169	26,6276	26,413		5	5	5	20,1	20,1	20,1	33,023	1,27E+09	36	26,1422	25,9378	26,3465	10	5	5	0,145458	0,408749	F9URA1	F9URA1	rnh	Ribonuclease H
25,9501	26,3291	25,779	26,4658	25,8532	25,4816	26,4497	27,3015	27,3025	27,3881		2	2	2	34,1	34,1	34,1	4,5785	2,13E+09	31	26,4301	26,0754	26,7847	10	5	5	0,108305	0,709234	F9ULD1	F9ULD1	lp_3576	Uncharacterized protein
NaN	NaN	NaN	NaN	NaN	NaN	24,4171	24,1004	24,2119	24,353		2	2	2	11,5	11,5	11,5	17,447	1,82E+08	6	24,2706	NaN	24,2706	4	0	4	1	NaN	F9UTV7	F9UTV7	lp_0360	Transcription regulator, Rrf2 family
NaN	NaN	23,8257	NaN	NaN	NaN	24,0784	24,1399	24,1777	24,0679		3	3	3	11,2	11,2	11,2	28,837	1,43E+08	14	24,0579	23,8257	24,116	5	1	4	1	0,290272	Q88VJ8	Q88VJ8	uppS	Ditrans,polycis-undecaprenyl-diphosphate synthase ((2E,6E)-farnesyl-diphosphate specific)
24,2686	NaN	NaN	NaN	NaN	NaN	24,8415	24,5983	24,7358	24,4253		2	2	2	9,1	9,1	9,1	29,568	2,39E+08	10	24,5739	24,2686	24,6502	5	1	4	1	0,381678	F9USH9	F9USH9	lp_3060	Transcription regulator, AraC family
22,7022	22,3469	NaN	NaN	NaN	22,6238	22,8995	24,3909	NaN	22,8086		2	2	2	4,5	4,5	4,5	52,607	7,66E+07	7	22,962	22,5245	23,1807	6	2	4	0,350126	0,656177	F9ULX2	F9ULX2	hpk3	Histidine kinase
24,7435	24,9443	24,8206	NaN	NaN	NaN	24,9378	25,1473	25,2589	25,1136	+	2	2	2	4,3	4,3	4,3	48,253	2,92E+08	10	24,9951	24,8361	25,1144	7	3	4	0,030046	0,278294	F9UL60	F9UL60	accC1	Acetyl-CoA carboxylase, biotin carboxylase subunit
24,2701	24,3028	24,3619	NaN	NaN	24,5309	NaN	24,5863	24,8062	24,6707	+	2	2	2	8,1	8,1	8,1	32,952	2,80E+08	17	24,5041	24,3116	24,6485	7	3	4	0,00621686	0,336902	Q88WK9	Q88WK9	rsgA1	Small ribosomal subunit biogenesis GTPase RsgA 1
23,6605	23,7304	23,7737	NaN	NaN	23,3275	NaN	23,7296	23,7062	23,828		4	4	4	27,2	27,2	27,2	16,896	2,08E+08	12	23,6794	23,7216	23,6478	7	3	4	0,603372	-0,073727	F9UM78	F9UM78	lp_0868	Metal uptake regulator
24,4776	NaN	24,3079	NaN	24,5673	24,6221	24,6499	NaN	24,2857	24,2859		3	3	3	16,2	16,2	16,2	45,369	2,14E+08	14	24,4566	24,4509	24,4609	7	3	4	0,944187	0,00998418	F9URV7	F9URV7	dapE2	Succinyl-diaminopimelate desuccinylase
27,5369	NaN	27,2628	26,4287	NaN	27,2239	27,0262	27,2469	27,2396	NaN		5	5	5	14,2	14,2	14,2	41,604	1,48E+09	6	27,1379	27,0762	27,1842	7	3	4	0,720953	0,108008	F9UNG1	F9UNG1	arcT	Aminotransferase
NaN	25,9094	25,7857	NaN	25,553	25,8186	NaN	25,7949	25,9061	26,085		3	3	3	9,6	9,6	9,6	43,474	5,44E+08	18	25,8361	25,7494	25,9011	7	3	4	0,251362	0,151775	Q88V41	Q88V41	ackA	Acetate kinase
NaN	NaN	24,1515	23,8731	22,9847	23,8324	NaN	23,8259	23,8621	23,8103		5	5	5	26	26	26	30,219	2,28E+08	17	23,7629	23,6698	23,8327	7	3	4	0,604198	0,162884	F9UR65	F9UR65	lp_2548	Beta-hydroxyacid dehydrogenase,3-hydroxyisobutyrate dehydrogenase family
24,0474	24,933	NaN	NaN	24,4696	NaN	24,6821	24,9752	25,1242	24,9903		3	3	3	53,8	53,8	53,8	7,4083	4,49E+08	16	24,746	24,4834	24,9429	7	3	4	0,114468	0,459581	F9UU98	F9UU98	lp_3419	Uncharacterized protein
26,194	26,7179	NaN	26,6258	26,6377	26,2439	26,1359	26,2954	26,2602	NaN	+	2	2	2	8,5	8,5	8,5	31,65	7,76E+08	13	26,3889	26,5438	26,2339	8	4	4	0,0456177	-0,309978	F9US99	F9US99	lp_2965	Bifunctional protein: transcription regulator sugar kinase, ROK family
NaN	25,7064	25,7944	25,6564	25,7167	NaN	25,8999	25,9034	25,9933	25,8205	+	2	2	2	12,3	12,3	12,3	26,136	4,55E+08	18	25,8114	25,7185	25,9043	8	4	4	0,00639751	0,185789	F9UTB6	F9UTB6	lp_3210	Cystine ABC transporter, permease protein
25,6262	25,8326	25,841	NaN	25,9257	25,9062	26,0789	26,0673	25,9632	NaN	+	3	3	3	24,8	24,8	24,8	13,989	6,38E+08	16	25,9052	25,8064	26,0039	8	4	4	0,0408461	0,197529	F9UTM3	F9UTM3	lp_3350	Uncharacterized protein
24,2125	NaN	24,4597	24,4745	24,4423	24,9384	25,0444	25,1007	25,4236	NaN	+	3	3	3	13	13	13	32,281	3,79E+08	12	24,762	24,3973	25,1268	8	4	4	0,0009609	0,729533	F9UMF8	F9UMF8	rluE	Pseudouridine synthase
NaN	24,0688	24,3964	24,4488	24,2357	23,8203	24,0408	23,9146	24,2245	NaN		2	2	2	10,4	10,4	10,4	25,796	2,57E+08	10	24,1437	24,2874	24	8	4	4	0,0573406	-0,28739	F9UU96	F9UU96	lp_3417	Transcription regulator, TetR family
25,2309	25,7175	25,7612	24,7994	NaN	25,008	25,2334	NaN	25,2447	25,0744		5	5	5	44,8	44,8	44,8	16,982	5,52E+08	19	25,2587	25,3772	25,1401	8	4	4	0,35092	-0,237147	F9UT41	F9UT41	ndk	Nucleoside-diphosphate kinase
25,4733	NaN	25,4905	25,6093	25,5249	25,2638	25,2906	25,5505	25,8853	NaN		3	3	3	13,2	13,2	13,2	30,55	4,47E+08	14	25,511	25,5245	25,4975	8	4	4	0,861078	-0,0269699	Q88VR4	Q88VR4	lp_1974	Putative pyruvate, phosphate dikinase regulatory protein
23,7588	NaN	23,7069	24,0847	24,0609	24,11	23,8321	NaN	24,1602	23,4196		2	2	2	22,5	22,5	22,5	14,638	2,14E+08	13	23,8917	23,9028	23,8805	8	4	4	0,913009	-0,022377	F9URB9	F9URB9	lp_2615	Transcription regulator, GntR family
24,1728	24,9253	24,9184	NaN	23,9874	NaN	24,5974	24,405	24,5938	24,6056		3	3	3	24,2	24,2	24,2	17,126	3,12E+08	9	24,5257	24,501	24,5504	8	4	4	0,850061	0,0494747	Q88X36	Q88X36	argR1	Arginine regulator
24,4828	24,3968	24,5954	NaN	24,2212	24,3495	24,3596	NaN	24,7052	24,4879		5	5	5	16	16	16	50,388	3,65E+08	17	24,4498	24,4241	24,4756	8	4	4	0,668285	0,0515003	F9UNR0	F9UNR0	ica1	Glycosyltransferase, family 2
24,7125	24,9996	25,0677	24,8686	NaN	24,9973	24,6608	25,3357	25,0239	NaN		2	2	2	8,2	8,2	8,2	32,317	2,86E+08	19	24,9583	24,9121	25,0044	8	4	4	0,58166	0,0923266	Q88YK2	Q88YK2	lgt	Phosphatidylglycerol--prolipoprotein diacylglyceryl transferase
25,2744	25,4328	NaN	25,1278	25,1642	25,275	25,3103	25,4761	25,4165	NaN		2	2	2	10	10	10	36,421	4,26E+08	12	25,3096	25,2498	25,3695	8	4	4	0,198612	0,119672	F9UQ98	F9UQ98	lp_2159	Diacylglycerol/lipid kinase
NaN	23,6417	23,5082	23,5469	24,0092	23,7596	23,7754	23,9189	23,8962	NaN		3	3	3	15,2	15,2	15,2	27,913	2,06E+08	15	23,757	23,6765	23,8375	8	4	4	0,233247	0,161004	F9URQ1	F9URQ1	lp_2783	Glycosyltransferase, family 2
23,9006	24,0498	NaN	24,2736	24,1333	23,9716	NaN	24,4521	24,6026	24,2627		3	3	3	9,8	9,8	9,8	50,16	2,76E+08	13	24,2058	24,0893	24,3222	8	4	4	0,187939	0,23293	F9US63	F9US63	lp_0038	Regulator of two-component system, YycH family
24,9943	25,1666	25,1785	25,1901	24,5387	23,9995	NaN	24,1141	23,99	23,8199	+	5	5	5	20,4	20,4	20,4	42,645	6,98E+08	21	24,5546	25,0136	23,9809	9	5	4	0,00023807	-1,03279	Q88SE8	Q88SE8	galK	Galactokinase
24,4171	24,886	24,9014	24,5827	24,6967	NaN	23,9788	23,9814	24,1128	23,9396	+	5	5	5	12	12	12	45,668	3,93E+08	16	24,3885	24,6968	24,0032	9	5	4	0,00038807	-0,693601	F9USY7	F9USY7	mdxE	Maltodextrin-binding protein
25,8596	25,6915	25,7379	25,6943	25,6225	25,763	25,8544	26,0223	NaN	26,026	+	5	5	5	17,5	17,5	17,5	31,514	1,07E+09	19	25,808	25,7212	25,9164	9	5	4	0,030658	0,195269	F9UM37	F9UM37	dacA	Diadenylate cyclase

23,8134	23,7653	23,5132	23,531	23,7543	NaN	23,9371	23,843	23,7968	23,9334	+	4	4	4	15,5	15,5	15,5	32,919	2,34E+08	18	23,7653	23,6754	23,8776	9	5	4	0,036058	0,202139	F9UPG9	F9UPG9	lp_1833	Integrase, N-terminal SAM-like
24,7027	24,7921	24,5725	24,6462	24,7891	NaN	24,9649	25,1065	24,9992	24,6857	+	5	5	5	15,2	15,2	15,2	38,076	4,02E+08	21	24,8065	24,7005	24,9391	9	5	4	0,0360071	0,23856	Q88YH7	Q88YH7	cggR	Central glycolytic genes regulator
26,5741	26,6328	26,4788	26,5748	26,7095	26,8989	26,7174	26,9778	NaN	26,8165	+	2	2	2	8,4	8,4	8,4	36,966	1,06E+09	13	26,709	26,594	26,8526	9	5	4	0,00544908	0,258629	F9UPY8	F9UPY8	ribC2	Riboflavin biosynthesis protein
24,0318	24,1137	24,2513	23,9786	24,1163	24,3682	NaN	24,3323	24,3791	24,4191	+	5	5	5	25,1	25,1	25,1	26,048	4,36E+08	21	24,2212	24,0983	24,3747	9	5	4	0,00147621	0,276361	F9URV3;F9US	F9URV3	lp_2851	Short-chain dehydrogenase/oxidoreductase
24,4742	24,3479	24,4162	24,1777	24,2483	NaN	24,5796	24,7954	24,8825	24,9057	+	3	3	3	12,8	12,8	12,8	30,967	3,28E+08	16	24,5364	24,3329	24,7908	9	5	4	0,00137277	0,45793	F9UPU6	F9UPU6	lytH	Probable cell wall amidase lytH
24,1582	24,2831	24,6021	24,1507	24,005	23,9867	NaN	23,9027	24,2252	24,0116		3	3	3	5,7	5,7	5,7	49,168	2,28E+08	12	24,1473	24,2399	24,0316	9	5	4	0,150764	-0,208294	F9UU22	F9UU22	pts7C	Permease IIC component
24,6327	25,1915	24,7267	24,8204	24,8145	NaN	24,8011	24,814	24,4006	24,5787		2	2	2	2,6	2,6	2,6	41,923	3,31E+08	27	24,7533	24,8372	24,6486	9	5	4	0,214875	-0,188558	F9UQ81	F9UQ81	ftsW	Cell division protein FtsW
24,543	24,4859	24,2295	24,4236	24,7947	24,2607	24,4219	24,4134	NaN	24,3865		3	3	3	20	20	20	13,813	2,99E+08	15	24,4399	24,4953	24,3707	9	5	4	0,290058	-0,124675	F9UL94;F9UQ	F9UL94	lp_0631	Prophage P1 protein 8, phage transcription regulator, Cro/CI family
24,4188	24,6977	24,7018	24,5107	24,6203	24,7228	NaN	24,4176	24,3352	24,4237		2	2	2	22,7	22,7	22,7	15,139	3,78E+08	16	24,5387	24,5899	24,4748	9	5	4	0,275952	-0,115043	F9US78	F9US78	lp_0058	Flavoprotein
23,8821	24,1128	24,3026	24,1881	24,6468	24,1826	NaN	24,112	23,9907	24,1802		3	3	3	19	19	19	21,153	3,55E+08	22	24,1775	24,2265	24,1164	9	5	4	0,480426	-0,110125	F9UNW2	F9UNW2	lp_1585	2-deoxyuridine 5-triphosphate nucleotidohydrolase (dUTPase)
24,0154	24,1629	24,2621	24,4558	24,366	24,1428	24,1938	24,1258	24,1172	NaN		2	2	2	6,9	6,9	6,9	35,501	2,19E+08	15	24,2046	24,2524	24,1449	9	5	4	0,264127	-0,107536	F9USL4	F9USL4	lp_3100	Aldo/keto reductase family protein
25,0463	25,1507	24,7794	25,0577	25,0121	NaN	24,7992	25,219	25,1358	24,8399		4	4	4	20,9	20,9	20,9	45,139	7,32E+08	12	25,0045	25,0092	24,9985	9	5	4	0,928733	-0,0107439	Q88W02	Q88W02	cca	CCA-adding enzyme
24,905	24,9942	25,0652	24,7906	24,7856	NaN	25,067	24,8605	25,0067	24,728		4	4	4	13,7	13,7	13,7	20,644	4,31E+08	18	24,9114	24,9081	24,9155	9	5	4	0,937832	0,00740061	Q88XQ3	Q88XQ3	lp_1140	UPF0316 protein lp_1140
26,484	26,5631	26,5666	26,3463	26,4965	26,4655	NaN	26,5906	26,5905	26,3929		3	3	3	12,6	12,6	12,6	26,652	1,32E+09	27	26,4996	26,4913	26,5099	9	5	4	0,774361	0,0185957	Q88WK0	Q88WK0	rnc	Ribonuclease 3
23,6235	23,5278	23,8485	23,7389	23,4067	23,8007	23,7218	23,6393	NaN	23,5456		2	2	2	10,4	10,4	10,4	22,011	1,52E+08	10	23,6503	23,6291	23,6769	9	5	4	0,647839	0,0477762	F9US17	F9US17	nrdG	Anaerobic ribonucleoside-triphosphate reductase-activating protein
24,8416	25,0627	25,2398	25,2519	24,8615	25,2054	25,0342	25,1552	25,0929	NaN		4	4	4	14,3	14,3	14,3	28,382	4,57E+08	18	25,0828	25,0515	25,1219	9	5	4	0,525346	0,0703803	F9UPF2	F9UPF2	lp_1807	Hypothetical membrane protein, DUF1003 family
24,5681	24,5626	24,8163	24,6408	24,9582	24,6941	NaN	24,8682	24,5697	25,0343		2	2	2	8,2	8,2	8,2	24,292	2,89E+08	26	24,7458	24,7092	24,7916	9	5	4	0,531005	0,0823654	F9UQA9	F9UQA9	ktrA	Potassium uptake protein
25,6671	25,3917	25,3135	25,2734	25,552	NaN	25,472	25,7331	25,4171	25,4976		6	6	6	11,6	11,6	11,6	65,562	5,65E+08	18	25,4797	25,4395	25,5299	9	5	4	0,414026	0,0903922	F9UUD3	F9UUD3	ubiB	Ubiquinone biosynthesis protein, ABC1 family
25,952	26,263	26,0741	25,9954	26,1293	26,2124	26,2956	26,1014	NaN	26,2386		2	2	2	37	37	37	9,0889	1,08E+09	19	26,1402	26,0828	26,212	9	5	4	0,113417	0,129233	F9UP02	F9UP02	acpA1	Acyl carrier protein
25,6739	25,7351	25,6347	25,632	25,8669	NaN	25,7857	25,4022	26,2484	25,9338		6	6	6	20,5	20,5	20,5	38,216	1,12E+09	18	25,7681	25,7085	25,8425	9	5	4	0,435432	0,134034	F9UN47	F9UN47	lp_1257	Uncharacterized protein
23,9653	24,0188	24,296	23,9501	24,008	NaN	24,1374	24,1249	24,2321	24,3153		2	2	2	4	4	4	72,21	3,72E+08	10	24,1164	24,0476	24,2024	9	5	4	0,1005	0,154806	F9UU49	F9UU49	copB	Copper transporting ATPase
24,6263	24,6057	24,4638	24,9423	25,0161	25,2252	NaN	24,7457	24,6736	24,9128		3	3	3	25,7	25,7	25,7	24,1	3,55E+08	14	24,8013	24,7309	24,8893	9	5	4	0,358521	0,158458	F9US70	F9US70	lp_0048	Uracil-DNA glycosylase family protein
25,333	24,9493	25,1213	24,918	25,0071	24,9077	25,2095	25,4681	25,4458	NaN		3	3	3	13,4	13,4	13,4	29,602	4,96E+08	22	25,1511	25,0657	25,2578	9	5	4	0,221037	0,192032	F9UM31	F9UM31	exoA	Exodeoxyribonuclease III
25,1669	25,0126	25,1958	24,7853	25,0799	NaN	25,252	25,5768	25,0736	25,2045		2	2	2	21,1	21,1	21,1	10,874	6,02E+08	15	25,1497	25,0481	25,2767	9	5	4	0,110958	0,228602	F9UL15	F9UL15	lp_0541	Ribosome-associated heat shock protein
25,1838	24,896	24,7339	24,5888	24,1569	NaN	24,9441	24,892	25,1506	24,8312		2	2	2	24,5	24,5	24,5	16,082	4,37E+08	20	24,8197	24,7119	24,9545	9	5	4	0,269848	0,242631	F9URF8	F9URF8	hsp2	Small heat shock protein
24,3343	24,6645	24,8315	24,8178	24,6454	NaN	24,9661	25,3007	25,0038	24,4931		3	3	3	22,1	22,1	22,1	15,318	3,85E+08	21	24,7841	24,6587	24,9409	9	5	4	0,157708	0,282211	F9UL76	F9UL76	mrnC	Mini-ribonuclease 3
27,1431	27,0419	26,9528	25,0625	26,873	27,1653	26,9383	26,8446	NaN	26,7623		3	3	3	13,2	13,2	13,2	37,563	1,25E+09	23	26,7537	26,6146	26,9276	9	5	4	0,508714	0,312971	Q88YN0	Q88YN0	tsaD	tRNA N6-adenosine threonylcarbamoyltransferase
24,3683	25,3764	24,3329	24,3199	24,0032	NaN	24,4185	25,0798	24,9931	25,0062		4	4	4	6,8	6,8	6,8	90,083	3,97E+08	8	24,6554	24,4801	24,8744	9	5	4	0,225978	0,394275	F9UNY9	F9UNY9	priA	Primosomal protein N
23,8875	24,0115	23,1267	23,8548	24,3039	24,3164	24,2225	24,4565	24,4099	NaN		3	3	3	18	18	18	22,261	2,26E+08	14	24,0655	23,8369	24,3513	9	5	4	0,0561909	0,51443	F9USU9	F9USU9	lp_0127	NADPH-dependent FMN reductase family protein
NaN	NaN	NaN	NaN	NaN	NaN	NaN	24,5636	24,4436	24,5077		2	2	2	9,5	9,5	9,5	31,558	1,09E+08	5	24,505	NaN	24,505	3	0	3	1	NaN	F9UKV4	F9UKV4	bla1	Beta-lactamase
NaN	NaN	NaN	NaN	NaN	NaN	26,3935	26,4775	NaN	26,4076		2	2	2	5,3	5,3	5,3	44,006	5,72E+08	9	26,4262	NaN	26,4262	3	0	3	1	NaN	F9UN39	F9UN39	lp_1247	Transport protein, DUF20 family
23,7418	NaN	NaN	NaN	23,9519	24,0328	24,0195	24,0307	NaN	NaN		5	5	5	12	12	12	66,554	2,28E+08	7	23,9553	23,8468	24,0277	5	2	3	0,104576	0,180829	F9UMF7	F9UMF7	recQ1	DNA helicase
24,5402	NaN	NaN	23,9479	NaN	NaN	24,8493	24,7373	24,6438	NaN		2	2	2	13,4	13,4	13,4	21,073	2,05E+08	12	24,5437	24,244	24,7435	5	2	3	0,122166	0,499438	F9UQF7	F9UQF7	ppiB	Peptidyl-prolyl cis-trans isomerase
24,2436	NaN	24,2581	24,0893	NaN	NaN	NaN	24,833	24,4603	24,5252	+	2	2	2	27,7	27,7	27,7	10,786	2,26E+08	11	24,4016	24,197	24,6061	6	3	3	0,0322479	0,409138	F9UPZ8	F9UPZ8	lp_2041	Ribosomal protein HS6-type (S12/L30/L7a)
NaN	25,504	25,9483	26,0128	NaN	26,3038	NaN	26,3402	NaN	26,246	+	2	2	2	11,9	11,9	11,9	28,96	6,53E+08	14	26,0592	25,8217	26,2967	6	3	3	0,0429416	0,474968	F9UR63	F9UR63	lp_2543	ABC transporter, ATP-binding protein
24,434	NaN	24,2056	NaN	24,8158	24,3733	24,1362	NaN	NaN	24,9502		2	2	2	16	16	16	20,564	2,94E+08	17	24,4858	24,4851	24,4866	6	3	3	0,996462	0,00141589	F9UT67	F9UT67	lpdB	Flavin prenyltransferase pdB
NaN	27,1542	27,0726	NaN	26,9344	26,7301	27,2105	NaN	NaN	27,4443		11	9	9	17,8	13,7	13,7	89,689	1,52E+09	9	27,091	27,0537	27,1283	6	3	3	0,751429	0,074578	Q88S87	Q88S87	lp_3551	Probable phosphoketolase 2
NaN	NaN	25,2317	25,311	25,2115	NaN	NaN	25,5286	25,4144	25,1081		3	3	3	12,5	12,5	12,5	54,495	4,27E+08	8	25,3009	25,2514	25,3504	6	3	3	0,486095	0,0990054	F9UN42	F9UN42	gntK	Gluconokinase
25,2805	25,2736	NaN	25,4821	NaN	25,5101	25,4608	25,3951	NaN	NaN		4	4	4	28,8	28,8	28,8	20,737	5,08E+08	16	25,4004	25,3454	25,4553	6	3	3	0,221945	0,109938	F9UNS0	F9UNS0	lp_1535	Uncharacterized protein
NaN	23,9333	23,7809	23,9167	23,8938	NaN	NaN	23,6646	23,713	23,6748	+	2	2	2	19,9	19,9	19,9	16,147	1,62E+08	9	23,7967	23,8812	23,6841	7	4	3	0,00566847	-0,197043	Q88V75	Q88V75	mraZ	Transcriptional regulator MraZ
25,5871	25,761	NaN	25,3983	25,6159	25,7714	25,7798	NaN	NaN	25,6841		2	2	2	6	6	6	26,442	5,08E+08	16	25,6568	25,5906	25,7451	7	4	3	0,153248	0,154531	Q88ZA0	Q88ZA0	cobB	NAD-dependent protein deacetylase
24,0993	24,6728	24,7223	24,667	24,582	NaN	23,9734	23,8554	24,2075	NaN	+	4	4	4	11,6	11,6	11,6	63,845	3,65E+08	15	24,3475	24,5487	24,0121	8	5	3	0,0198645	-0,536564	F9UT32	F9UT32	pts2CB	EIICB-Mtl
25,0882	25,512	25,3717	25,6868	25,5065	NaN	NaN	25,9554	25,7935	25,8198	+	2	2	2	10,8	10,8	10,8	26,8	4,72E+08	9	25,5917	25,433	25,8562	8	5	3	0,021943	0,423207	F9UMH7	F9UMH7	lp_0990	Uncharacterized protein
25,6017	25,7578	25,6312	25,7275	25,4991	NaN	NaN	25,3509	25,8819	24,0172		2	2	2	13	13	13	26,838	6,61E+08	17	25,4334	25,6435	25,0834	8	5	3	0,220629	-0,560112	F9UQF3	F9UQF3	lp_2229	Metal-dependent hydrolase, beta-lactamase superfamily III
23,2954	23,2307	23,5905	23,5772	23,4136	23,3187	23,3324	NaN	NaN	23,3422		2	2	2	14	14	14	24,528	1,35E+08	12	23,3876	23,4215	23,3311	8	5	3	0,386418	-0,0904022	F9UUB9	F9UUB9	lp_3444	Transcription regulator, Crp family
23,3913	23,5335	23,6437	23,4527	22,9662	23,3735	23,2232	23,3838	NaN	NaN		2	2	2	22,8	22,8	22,8	14,791	9,29E+07	10	23,371	23,3975	23,3268	8	5	3	0,672275	-0,0706758	F9UPL9	F9UPL9	lp_1891	Uncharacterized protein
24,4217	24,7688	24,5293	24,4044	24,1916	24,4231	24,2037	NaN	24,6524	NaN		6	6	6	10,3	10,3	10,3	89,58	3,36E+08	15	24,4494	24,4632	24,4264	8	5	3	0,822619	-0,0367755	F9US03	F9US03	uvrA3	Excinuclease ABC, subunit A
25,3935	25,1807	25,1803	25,2456	25,2297	25,1073	25,2261	25,3278	NaN	NaN		3	3	3	16,2	16,2	16,2	25,62	3,52E+08	12	25,2364	25,246	25,2204	8	5	3	0,726977	-0,0255774	F9UPN0	F9UPN0	lp_1906	S-adenosyl-L-methionine-dependent methyltransferase
25,7672	25,7641	25,7768	25,8515	25,7552	25,9599	25,925	NaN	25,6319	NaN		3	3	3	22,8	22,8	22,8	16,946	6,43E+08	11	25,804	25,783	25,839	8	5	3	0,50741	0,0559887	F9UTD8	F9UTD8	lp_3238	Transcription regulator, MerR family
24,9234	24,9718	25,0921	24,906	25,1844	25,0294	NaN	NaN	25,0885	25,2907		2	2	2	9,6	9,6	9,6	24,479	4,57E+08	21	25,0608	25,0156	25,1362	8	5	3	0,235772	0,120649	F9UQF8	F9UQF8	lp_2232	Hypothetical membrane protein, DedA family
24,527	24,6657	24,4066	24,4225	24,3938	24,6809	24,6491	NaN	24,5686	NaN		2	2	2	10,2	10,2	10,2	28,324	2,44E+08	15	24,5393	24,4831	24,6329	8	5	3	0,0851222	0,149754	F9UST7	F9UST7	thiD	Phosphomethylpyrimidine kinase & hydroxymethylpyrimidine kinase
25,826	25,3785	25,7351	25,6869	25,8928	NaN	26,1329	25,8943	25,7462	NaN		3	3	3	11,1	11,1	11,1	32,31	6,15E+08	18	25,7866	25,7039	25,9245	8	5	3	0,176835	0,220636	F9URR8	F9URR8	lp_2803	2,5 diketo-D-gluconic acid-like reductase NADP dependent (Promiscuous)
24,4889	23,869	23,6008	24,4092	24,4134	NaN	NaN	24,6113	24,5406	24,3546		3	3	3	8,8	8,8	8,8	40,374	2,09E+08	9	24,286	24,1563	24,5022	8	5	3	0,205258	0,345891	F9ULW1	F9ULW1	tagO	Undecaprenyl-phosphate N-acetyl-glucosaminyl transferase
24,3448	NaN	24,3847	24,3927	NaN	NaN	24,9627	NaN	24,8252	NaN	+	2	2	2	10	10	10	24,957	2,33E+08	13	24,582	24,3741	24,8939	5	3	2	0,00247129	0,519886	F9UQA5	F9UQA5	pgm6	Phosphoglycerate mutase family protein
23,3415	23,2843	22,9274	22,5861	NaN	22,6103	23,0098	NaN	NaN	NaN		3	3	3	5,2	5,2	5,2	87,319	7,25E+07	8	22,9599	23,0348	22,8101	6	4	2	0,481863	-0,224738	F9UUJ6	F9UUJ6	agl	Alpha-glucosidase (Malto-oligosaccharides)
25,0481	25,077	NaN	24,9082	25,3299	24,9788	NaN	NaN	25,3071	NaN		5	5	5	58,3	58,3	58,3	13,193	4,80E+08	10	25,1082	25,0908	25,1429	6	4	2	0,768703	0,0521421	Q88Z44	Q88Z44	acpS	Holo-[acyl-carrier-protein] synthase
25,352	25,4897	25,832	25,3403	25,068	24,2527	NaN	24,8062	NaN	NaN	+	2	2	2	17,4	17,4	17,4	16,017	4,40E+08	17	25,163	25,4164	24,5295	7	5	2	0,0175615	-0,886931	F9URD6	F9URD6	lp_2641	Uncharacterized protein
26,2206	26,2705	26,6503	26,413	26,8304	26,5884	NaN	NaN	NaN	26,0978		4	4	4	54,7	54,7	54,7	6,175	8,54E+08	17	26,4387	26,477	26,3431	7	5	2	0,590348	-0,133912	Q6LWF7	Q6LWF7	orf20	Uncharacterized protein (Fragment)
23,7958	24,0171	23,8758	23,7431	23,787	NaN	NaN	24,1716	23,7877	NaN		2	2	2	7,8	7,8	7,8	43,946	1,27E+08	10	23,8826	23,8438	23,9796	7	5	2	0,343283	0,135867	F9UUD1	F9UUD1	lp_3461	ATPase, MoxR family
25,1691	NaN	NaN	23,6847	23,8371	23,5457	NaN	NaN	NaN	NaN		4	4	4	18,7	18,7	18,7	26,914	2,61E+08	9	24,0592	24,2303	23,5457	4	3	1	1	-0,684642	F9USU4	F9USU4	lp_0121	Uncharacterized protein
23,9313	23,6733	24,1204	NaN	NaN	NaN	NaN	NaN	23,5444	NaN		4	4	4	13,9	13,9	13,9	47,784	2,29E+08	15	23,8173	23,9083	23,5444	4	3	1	1	-0,363937	Q88Z60	Q88Z60	serS1	Serine--tRNA ligase 1
24,7153	24,5416	NaN	24,8391	NaN	NaN	NaN	NaN	NaN	24,3478		6	6	6	9,5	9,5	9,5	97,814	3,74E+08	12	24,611	24,6987	24,3478	4	3	1	1	-0,350876	F9ULS7	F9ULS7	mprF	Phosphatidylglycerol lysyltransferase
25,0587	25,3836	25,414	NaN	NaN	NaN	NaN	NaN	NaN	25,0259		3	3	3	9,8	9,8	9,8	32,861	3,97E+08	8	25,2206	25,2854	25,0259	4	3	1	1	-0,25952	F9UPQ9	F9UPQ9	lp_1938	Transcription regulator, ysR family
24,1558	NaN	23,7088	23,5992	NaN	23,6051	NaN	NaN	NaN	NaN		2	2	2	7,4	7,4	7,4	31,735	1,12E+08	8	23,7673	23,8213	23,6051	4	3	1	1	-0,216176	F9UM59	F9UM59	licD	Lipooligosaccharide cholinephosphotransferase
NaN	24,5888	24,4041	24,31	NaN	NaN	NaN	24,367	NaN	NaN		4	4	4	7,8	7,8	7,8	99,717	2,79E+08	5	24,4175	24,4343	24,367	4	3	1	1	-0,0672976	F9USU6	F9USU6	pacL1	Cation transporting P-type ATPase
27,1415	NaN	27,4342	27,7769	27,6763	NaN	NaN	NaN	NaN	26,5723		2	2	2	6,6	6,6	6,6	57,877	1,03E+09	6	27,3203	27,5072	26,5723	5	4	1	1	-0,934881	F9UN84	F9UN84	tagE2	Poly(Glycerol-phosphate) alpha-glucosyltransferase
27,025	26,4788	26,5642	NaN	26,8557	26,225	NaN	NaN	NaN	NaN		2	2	2	13,6	13,6	13,6	15,611	8,95E+08	18	26,6297	26,7309	26,225	5	4	1	1	-0,505865	F9UPE2	F9UPE2	lp_1795	Transcription regulator, MarR family
25,2289	NaN	25,2329	25,3711	25,3936	NaN	NaN	NaN	25,0618	NaN		4	4	4	21,7	21,7	21,7	30,14	4,11E+08	5	25,2577	25,3066	25,0618	5	4	1	1	-0,244881	F9UUH1	F9UUH1	estA	Acetyl esterase (Promiscuous)
24,1108	NaN	24,3262	23,9301	23,7324	NaN	NaN	NaN	24,0752	NaN		3	3	3	22,3	22,3	22,3	20,509	1,52E+08	12	24,0349	24,0249	24,0752	5	4	1	1	0,0503435	F9UQB6	F9UQB6	lp_2183	ADP-ribose pyrophosphatase
25,934	26,0774	25,9978	26,1911	26,3113	25,7914	NaN	NaN	NaN	NaN		3	3	3	10,7	10,7	10,7	37,175	6,68E+08	18	26,0505	26,1023	25,7914	6	5	1	1	-0,310933	F9UQG6	F9UQG6	lp_2243	Type I restriction-modification system,adenine-specific methyltransferase subunit
23,5104	23,7473	24,0853	23,7869	23,681	NaN	NaN	NaN	NaN	23,5723		2	2	2	20,8	20,8	20,8	16,138	1,46E+08	6	23,7305	23,7622	23,5723	6	5	1	1	-0,189872	F9UT34	F9UT34	pts2A	PTS system, mannitol-specific EIIA component
23,481	23,548	23,6978	23,4715	23,7445	NaN	NaN	23,6917	NaN	NaN		3	3	3	14,6	14,6	14,6	23,972	1,20E+08	16	23,6058	23,5886	23,6917	6	5	1	1	0,103117	F9UQM1	F9UQM1	glnP2	Glutamine/histidine ABC transporter, permease protein
27,3907	27,2701	27,2378	27,0346	27,1697	27,3518	NaN	NaN	NaN	NaN		2	2	2	9,3	9,3	9,3	21,369	1,09E+09	22	27,2424	27,2206	27,3518	6	5	1	1	0,131202	F9UQL4	F9UQL4	pgsA	CDP-diacylglycerol--glycerol-3-phosphate 3-phosphatidyltransferase
24,8015	25,2331	25,2608	25,423	24,9673	NaN	NaN	NaN	25,8663	NaN		2	2	2	21,7	21,7	21,7	11,903	3,45E+08	15	25,2587	25,1372	25,8663	6	5	1	1	0,729111	F9UUB4	F9UUB4	trxA3	Thioredoxin
26,6085	26,6684	NaN	26,7484	26,5318	NaN	NaN	NaN	NaN	NaN		3	3	3	11,3	11,3	11,3	34,33	7,04E+08	4	26,6393	26,6393	NaN	4	4	0	1	NaN	F9UMS5	F9UMS5	mleR1	Malolactic regulator, LysR family
24,4326	24,8352	24,6829	24,7828	24,7578	NaN	NaN	NaN	NaN	NaN		2	2	2	8,2	8,2	8,2	28,491	2,60E+08	16	24,6983	24,6983	NaN	5	5	0	1	NaN	F9ULA6	F9ULA6	lp_3549	Transcription regulator, DeoR family
24,133	24,0736	24,217	24,1447	24,226	NaN	NaN	NaN	NaN	NaN		2	2	2	8,7	8,7	8,7	27,376	2,18E+08	11	24,1589	24,1589	NaN	5	5	0	1	NaN	F9URF1	F9URF1	lp_2661	Transcription regulator, GntR family
23,8722	24,1297	23,7318	23,7737	23,37	NaN	NaN	NaN	NaN	NaN		2	2	2	5,2	5,2	5,2	39,084	1,72E+08	12	23,7755	23,7755	NaN	5	5	0	1	NaN	F9UTH1	F9UTH1	lp_3281	Hypothetical membrane protein, DUF20 family
26,0732	25,905	25,696	25,8385	25,6309	NaN	NaN	NaN	NaN	NaN		2	2	2	12,6	12,6	12,6	19,738	5,48E+08	21	25,8287	25,8287	NaN	5	5	0	1	NaN	F9UTS6	F9UTS6	lp_0322	Acetyltransferase, GNAT family
23,3508	23,4744	23,5516	23,8031	23,7088	NaN	NaN	NaN	NaN	NaN		4	4	4	25,9	25,9	25,9	28,054	2,22E+08	7	23,5778	23,5778	NaN	5	5	0	1	NaN	Q88YK7	Q88YK7	pstB2	Phosphate import ATP-binding protein PstB 2
24,1381	25,0706	24,321	24,7322	24,4987	NaN	NaN	NaN	NaN	NaN		4	4	4	28,3	28,3	28,3	18,109	3,08E+08	6	24,5521	24,5521	NaN	5	5	0	1	NaN	Q890H4	Q890H4	rlmH	Ribosomal RNA large subunit methyltransferase H

Table S2. Total data of proteins identified in this study for *Lacticaseibacillus paracasei* 126 along with Log2 fold change (in blue) and significance values (p, in red) of the 30 μg/mL punicalagin (P) treatment in comparison to the non-treated control (B). Control samples in yellow, samples treated with P in green

LFQ intensity 74C1	LFQ intensity 74C2	LFQ intensity 74C3	LFQ intensity 74C4	LFQ intensity 74C5	LFQ intensity 74P1	LFQ intensity 74P2	LFQ intensity 74P3	LFQ intensity 74P4	LFQ intensity 74P5	T-test Significant	Peptides	Razor + unique peptides	Unique peptides	Sequence coverage [%]	Unique + razor sequence coverage [%]	Unique sequence coverage [%]	Mol. weight [kDa]	Intensity	MS/MS count	Mean	Mean Control	Mean Punicalagin	Valid values	Valid values Control	Valid values Punicalagin	T-test p-value	T-test Difference	Protein IDs	Majority protein IDs	Gene name	Protein name
NaN	26,1292	26,1861	26,161	NaN	26,0787	26,1714	26,1923	26,4578	26,1411		2	2	2	32,2	32,2	32,2	9,8794	3,19E+08	17	26,1897	26,1588	26,2083	8	3	5	0,593666	0,049484	Q034V4	Q034V4	LSEI_2545	Ribosome-associated heat shock protein implicated in the recycling of the 50S subunit (S4 paralog)
23,8202	23,7829	NaN	23,6583	23,8152	23,838	24,1728	24,1657	23,9864	24,0603	+	4	4	4	13,6	13,6	13,6	39,446	1,41E+08	13	23,9222	23,7691	24,0446	9	4	5	0,00956626	0,275503	Q03C61	Q03C61	LSEI_0352	CRISPR-associated protein, Cse4 family
NaN	25,5801	25,6955	26,3336	25,9559	24,3589	25,2685	24,384	25,4024	23,9382	+	2	2	2	18,2	18,2	18,2	7,7781	4,76E+08	27	25,213	25,8913	24,6704	9	4	5	0,0106374	-1,22086	Q038A1	Q038A1	rpmI	50S ribosomal protein L35
NaN	24,7857	24,6763	24,7394	24,542	24,5809	24,7231	24,8282	24,8364	24,7457		2	2	2	9,3	9,3	9,3	32,358	1,90E+08	8	24,7175	24,6858	24,7428	9	4	5	0,44251	0,056999	Q03AA9	Q03AA9	LSEI_1069	Uncharacterized protein
NaN	24,5129	24,2931	24,4114	24,6874	25,1661	24,8102	24,5923	25,8232	25,4522	+	2	2	2	29,3	29,3	29,3	9,2045	1,70E+08	14	24,861	24,4762	25,1688	9	4	5	0,0323719	0,692644	Q038H0	Q038H0	rpoZ	DNA-directed RNA polymerase subunit omega
23,9898	23,9199	24,1201	NaN	23,6739	24,9924	25,4137	25,2387	25,2266	25,3214	+	2	2	2	14,9	14,9	14,9	15,727	1,35E+08	12	24,6552	23,9259	25,2386	9	4	5	8,59E-06	1,31264	Q036C9	Q036C9	LSEI_2196	NusG_II domain-containing protein
23,7265	23,8187	24,3731	NaN	24,35	24,9905	25,4811	25,0829	24,9576	25,767	+	5	5	5	25,1	25,1	25,1	25,987	2,43E+08	14	24,7275	24,0671	25,2558	9	4	5	0,00143537	1,18874	Q03CP9	Q03CP9	LSEI_0159	Cell surface protein
23,7606	23,9174	24,1562	24,5942	NaN	26,1915	26,0624	25,5762	25,8478	25,9292	+	3	3	3	15,7	15,7	15,7	31,149	3,81E+08	9	25,1151	24,1071	25,9214	9	4	5	3,86E-05	1,81428	Q034W4	Q034W4	LSEI_2535	WxL domain-containing protein
23,7731	23,4515	23,3632	23,1156	23,4671	22,507	22,7807	22,759	23,2559	22,5549	+	7	7	7	24,8	24,8	24,8	31,972	1,49E+08	14	23,1028	23,4341	22,7715	10	5	5	0,00448571	-0,662614	Q039E7	Q039E7	LSEI_1397	Ribosome biogenesis GTPase A
23,7936	23,4333	23,5953	23,5935	23,2449	23,0436	22,9957	22,9701	23,3931	22,8944	+	6	6	6	21,1	21,1	21,1	37,414	1,92E+08	20	23,2957	23,5321	23,0594	10	5	5	0,00569397	-0,472729	Q03AL3	Q03AL3	LSEI_0960	Putative gluconeogenesis factor
22,5275	23,3892	23,1001	23,0293	22,886	23,1349	23,1062	23,1372	23,5656	23,1376		3	3	3	18,9	18,9	18,9	26,424	1,04E+08	14	23,1014	22,9864	23,2163	10	5	5	0,203435	0,229857	Q03AX5	Q03AX5	LSEI_0811	Predicted hydrolase (HAD superfamily)
23,6529	23,7851	23,7945	23,6423	23,6781	23,1314	23,4116	23,5442	23,5197	22,8027	+	4	4	4	15,7	15,7	15,7	49,06	1,43E+08	15	23,4962	23,7106	23,2819	10	5	5	0,0178083	-0,428681	Q035H4	Q035H4	fumC	Fumarate hydratase class II
23,159	23,6385	23,575	23,3416	23,2814	23,1888	23,3221	23,3534	23,2703	23,5949		5	5	5	7,9	7,9	7,9	80,576	1,34E+08	21	23,3725	23,3991	23,3459	10	5	5	0,650758	-0,0532051	Q039U7	Q039U7	LSEI_1240	Transcriptional accessory protein
22,7018	23,2232	22,8116	22,6451	22,9368	23,3877	23,1329	23,304	23,4477	23,6246	+	6	6	6	10,8	10,8	10,8	72,517	1,27E+08	6	23,1215	22,8637	23,3794	10	5	5	0,0042964	0,515648	Q038H6	Q038H6	LSEI_1622	Non-specific serine/threonine protein kinase
23,7181	23,7044	23,5538	23,7241	23,662	23,334	23,4862	23,3411	23,2597	23,528	+	13	13	13	24,5	24,5	24,5	63,962	3,97E+08	32	23,5311	23,6725	23,3898	10	5	5	0,00144104	-0,282676	Q037R7	Q037R7	LSEI_1784	Pyruvate oxidase
23,0632	23,4953	23,5174	23,816	23,2954	23,2989	23,3791	23,4147	23,8753	23,037		3	3	3	11,4	11,4	11,4	25,681	1,40E+08	15	23,4192	23,4375	23,401	10	5	5	0,848396	-0,0364571	Q037W6	Q037W6	mecA	Adapter protein MecA
24,3866	24,746	24,5242	24,3674	24,3035	23,2461	23,5651	23,1755	23,9153	23,1807	+	5	5	5	18,6	18,6	18,6	40,586	3,01E+08	20	23,941	24,4655	23,4166	10	5	5	0,000209907	-1,04898	Q037U5	Q037U5	purK	N5-carboxyaminoimidazole ribonucleotide synthase
23,5165	23,7044	23,631	23,768	23,3651	22,9086	23,4411	23,3577	23,8219	23,815		9	9	9	21,1	21,1	21,1	49,38	2,96E+08	28	23,5329	23,597	23,4689	10	5	5	0,504869	-0,128133	Q03A30	Q03A30	murT	Lipid II isoglutaminyl synthase (glutamine-hydrolyzing) subunit MurT
23,3116	23,3554	23,2135	23,5721	23,1502	23,4002	23,5442	23,3768	23,7601	23,274		13	13	13	12,4	12,4	12,4	131,48	2,31E+08	28	23,3958	23,3206	23,471	10	5	5	0,212477	0,15047	Q034V2	Q034V2	mfd	Transcription-repair-coupling factor
23,6204	23,4364	23,4111	23,4945	23,3806	23,4807	23,5078	23,4807	23,4068	23,5949		10	10	10	48,8	48,8	48,8	28,248	2,63E+08	17	23,4814	23,4686	23,4942	10	5	5	0,635835	0,0256062	Q03CF9	Q03CF9	LSEI_0250	Lactose transport regulator
23,6992	23,9606	23,9791	23,8237	23,7995	23,3418	23,6582	23,5901	23,3719	23,5627	+	10	10	9	16,1	16,1	15,2	90,41	3,60E+08	21	23,6787	23,8524	23,5049	10	5	5	0,00277238	-0,347486	Q037Y7	Q037Y7	LSEI_1712	DNA translocase FtsK
23,4148	23,3069	23,4303	23,5986	23,5533	23,2781	23,4801	23,4493	23,8316	23,5107		3	3	3	14,9	14,9	14,9	24,598	1,18E+08	12	23,4854	23,4608	23,51	10	5	5	0,648548	0,0492077	Q038D4	Q038D4	LSEI_1665	DNA-binding response regulator, CitB family (Rec-wHTH domains)
24,5278	23,8074	24,3378	24,5413	23,9491	24,0631	23,6579	23,8878	23,097	23,0025	+	7	7	7	32,7	32,7	32,7	33,497	2,87E+08	19	23,8872	24,2327	23,5417	10	5	5	0,0287329	-0,691046	Q039L0	Q039L0	LSEI_1331	BS_ysoA related protein with TPR repeats
23,665	23,5782	23,4512	23,6032	23,4464	23,7636	23,5153	23,5043	23,4047	23,885		6	6	6	21,3	21,3	21,3	31,923	2,02E+08	17	23,5817	23,5488	23,6146	10	5	5	0,527825	0,0657864	Q039Q7	Q039Q7	LSEI_1282	Phosphate-binding protein
24,5307	24,0862	23,7105	24,0432	23,7297	23,909	23,748	23,4675	23,4727	23,7339		11	11	11	34,8	34,8	34,8	39,137	3,21E+08	29	23,8431	24,0201	23,6662	10	5	5	0,0739227	-0,353849	Q03AV3	Q03AV3	LSEI_0862	Glycosyltransferase
24,1377	24,0046	23,9909	23,7818	23,9093	23,6965	23,5462	23,5665	23,7625	23,7642	+	10	10	10	19,8	19,8	19,8	62,626	2,58E+08	31	23,816	23,9649	23,6672	10	5	5	0,00416221	-0,297707	Q035W5	Q035W5	dnaX	DNA polymerase III subunit gamma/tau
23,7691	24,1725	23,9827	23,7201	23,739	24,0259	23,6901	23,7939	23,7043	23,1275		4	4	4	28,1	28,1	28,1	25,419	1,95E+08	22	23,7725	23,8767	23,6683	10	5	5	0,260312	-0,208353	Q03CC2	Q03CC2	LSEI_0290	Uncharacterized protein
23,1119	23,8661	24,1482	24,0757	23,8768	24,0224	23,683	23,6604	23,8943	23,3086		4	4	4	13,5	13,5	13,5	36,843	1,23E+08	9	23,7647	23,8157	23,7137	10	5	5	0,656491	-0,102003	Q03C01	Q03C01	LSEI_0415	Uncharacterized protein
23,969	24,0241	23,7428	23,5962	23,8764	23,4224	23,9519	23,9693	23,5645	23,7869		9	9	9	24	24	24	62,744	3,41E+08	24	23,7903	23,8417	23,739	10	5	5	0,461155	-0,1027	Q038G5	Q038G5	LSEI_1633	DNA repair protein RecN
23,8023	23,8357	23,8945	23,7443	23,7335	23,5515	23,6411	23,7225	24,018	23,8012		6	6	6	21,8	21,8	21,8	36,059	2,63E+08	29	23,7745	23,802	23,7469	10	5	5	0,533993	-0,0551743	Q036Y4	Q036Y4	coaA	Pantothenate kinase
22,7626	23,5392	23,2907	23,6082	23,8443	23,903	23,7741	23,6772	23,9117	23,7887		3	3	3	15,6	15,6	15,6	17,722	1,55E+08	12	23,61	23,409	23,8109	10	5	5	0,0664159	0,401942	Q034Q5	Q034Q5	LSEI_2596	PTS system IIA component, Gat family
23,2721	24,0037	23,8371	23,9713	24,0585	24,0503	23,7595	24,0383	23,9844	23,2621		3	3	3	24,3	24,3	24,3	18,249	1,85E+08	10	23,8237	23,8285	23,8189	10	5	5	0,964145	-0,00959625	Q034P0	Q034P0	LSEI_2612	Phospholipid-binding protein
23,5617	24,0181	24,0885	23,9181	23,8314	23,7884	23,8295	23,8822	23,8776	23,7185		3	3	3	16,9	16,9	16,9	26,617	1,54E+08	12	23,8514	23,8836	23,8192	10	5	5	0,523535	-0,0643532	Q037Y0	Q037Y0	LSEI_1719	ABC-type multidrug transport system, ATPase component
24,3625	24,3157	24,1073	24,435	24,2821	23,5069	23,8379	23,9333	24,0976	23,7444	+	5	5	5	24,4	24,4	24,4	31,86	2,38E+08	16	24,0623	24,3005	23,824	10	5	5	0,0028761	-0,476476	Q039D2	Q039D2	LSEI_1412	Pyruvate formate-lyase-activating enzyme
23,6547	23,881	23,7882	23,5648	23,6565	23,635	23,7619	23,7085	24,0001	24,0149		8	8	8	36,1	36,1	36,1	33,122	3,35E+08	25	23,7666	23,709	23,8241	10	5	5	0,263127	0,115041	Q033X8	Q033X8	LSEI_2780	Exopolyphosphatase-related protein
24,5519	24,3456	24,2125	24,14	24,2676	23,7645	23,9787	23,6619	24,1091	23,7599	+	7	7	7	22,8	22,8	22,8	33,719	2,70E+08	25	24,0792	24,3035	23,8548	10	5	5	0,00322308	-0,448718	Q03BE6	Q03BE6	ldh	L-lactate dehydrogenase
23,5917	23,7392	23,7509	24,0048	23,6001	23,7658	23,893	23,8814	23,9596	23,8784		4	4	4	16,8	16,8	16,8	27,36	1,71E+08	4	23,8065	23,7374	23,8756	10	5	5	0,126187	0,13827	Q037N1	Q037N1	LSEI_1824	Unchracterized domain and predicted acyltransferase
24,3137	23,9833	23,9382	24,0341	24,1011	23,9287	23,6533	23,8511	24,0022	24,0615		11	11	11	32,9	32,9	32,9	38,443	3,65E+08	42	23,9867	24,0741	23,8994	10	5	5	0,108461	-0,174715	Q038X1	Q038X1	LSEI_1473	Lipoate--protein ligase
23,5845	24,2271	24,0918	24,1079	24,2687	23,7735	23,7918	23,8325	24,2415	23,8581		5	5	5	15	15	15	33,673	2,39E+08	15	23,9778	24,056	23,8995	10	5	5	0,328042	-0,156496	Q034S7	Q034S7	LSEI_2572	Predicted integral membrane protein
24,8665	24,7795	24,5112	24,3917	24,7567	24,0889	24,133	23,7547	23,6005	24,0793	+	9	9	9	32,9	32,9	32,9	36,349	4,06E+08	22	24,2962	24,6611	23,9313	10	5	5	0,00078621	-0,729837	Q037X6	Q037X6	LSEI_1723	Predicted HD-superfamily hydrolase
23,2791	24,3745	24,0511	24,244	24,1145	23,7849	23,9961	23,8978	24,1793	23,8783		5	5	5	32	32	32	20,019	3,11E+08	17	23,98	24,0126	23,9473	10	5	5	0,755692	-0,0653713	Q038A8	Q038A8	LSEI_1691	Predicted hydrolase of the HAD superfamily
24,0989	24,2682	24,3502	24,3036	23,9255	24,0554	23,7789	23,8406	24,1725	24,1488		2	2	2	13	13	13	24,959	2,35E+08	14	24,0943	24,1893	23,9992	10	5	5	0,129007	-0,190059	Q03CK1	Q03CK1	gatD	Lipid II isoglutaminyl synthase (glutamine-hydrolyzing) subunit GatD
24,5708	24,2979	24,1338	24,6642	24,2331	24,111	23,7804	24,045	23,9931	24,0734	+	7	7	7	31,4	31,4	31,4	25,057	4,81E+08	28	24,1903	24,3799	24,0006	10	5	5	0,0118743	-0,379372	Q037H0	Q037H0	LSEI_1893	Putative intracellular protease/amidase
24,4231	24,4418	24,2955	24,3089	24,1275	24,1228	24,0263	24,1025	24,1476	23,8614	+	10	10	10	30,7	30,7	30,7	52,021	4,96E+08	43	24,1857	24,3194	24,0521	10	5	5	0,00813164	-0,267279	Q034S3	Q034S3	LSEI_2576	HD superfamily phosphohydrolase
24,1548	24,0625	23,8533	23,2992	23,9895	23,8199	24,14	24,211	23,9914	24,1254		5	5	5	10,9	10,9	10,9	60,354	1,89E+08	15	23,9647	23,8719	24,0576	10	5	5	0,297107	0,18568	Q037M2	Q037M2	LSEI_1841	Acetolactate synthase, large subunit
24,2537	24,5589	24,1956	24,0861	24,1061	23,7964	24,097	24,0623	24,3117	24,0268		8	8	8	29,2	29,2	29,2	43,178	3,20E+08	17	24,1494	24,2401	24,0588	10	5	5	0,16463	-0,181239	Q036J7	Q036J7	LSEI_2125	Alcohol-acetaldehyde dehydrogenase
24,1128	24,4192	24,1661	24,378	24,3882	24,2156	24,1605	24,1524	24,5672	23,2034		6	6	6	34,8	34,8	34,8	22,792	3,49E+08	25	24,1763	24,2928	24,0599	10	5	5	0,35259	-0,232987	Q033T7	Q033T7	LSEI_2822	Amidase
23,9694	24,1203	23,9637	24,1411	23,8822	24,1556	23,8593	24,2258	24,0316	24,0578		7	7	7	15,1	15,1	15,1	72,11	2,64E+08	20	24,0407	24,0153	24,066	10	5	5	0,542278	0,050692	Q035Z3	Q035Z3	mutL	DNA mismatch repair protein MutL
23,8087	24,4142	24,0876	24,1579	23,9202	24,0079	24,1706	24,1779	24,0962	24,0292		4	4	4	18,7	18,7	18,7	30,481	1,98E+08	12	24,087	24,0777	24,0963	10	5	5	0,869597	0,0186241	Q035B2	Q035B2	ecfA1	Energy-coupling factor transporter ATP-binding protein EcfA1
23,8818	24,3504	24,2743	24,2266	24,338	23,966	24,1247	24,2373	24,1147	24,1257		7	7	7	24	24	24	48,664	3,51E+08	22	24,164	24,2142	24,1137	10	5	5	0,327145	-0,100517	Q038H4	Q038H4	LSEI_1624	16S rRNA m5C967 methyltransferase
23,8268	24,1011	24,2668	23,9942	24,2538	23,9008	24,0518	24,3031	24,1479	24,1963		4	4	4	13,4	13,4	13,4	44,58	2,22E+08	16	24,1043	24,0885	24,12	10	5	5	0,776494	0,0314571	Q035M3	Q035M3	proA	Gamma-glutamyl phosphate reductase
24,5026	24,7146	24,2899	24,2571	24,1771	24,3285	24,2675	24,2274	24,5129	23,2839		10	10	10	24,7	24,7	24,7	54,617	4,16E+08	24	24,2562	24,3883	24,124	10	5	5	0,296795	-0,264243	Q037K6	Q037K6	LSEI_1857	Citrate lyase alpha chain
24,1504	24,3276	24,1749	24,3926	24,3703	24,2762	24,379	23,9232	24,5309	23,5627		9	9	9	64,4	64,4	64,4	22,839	3,64E+08	36	24,2088	24,2832	24,1344	10	5	5	0,43642	-0,148759	Q038R0	Q038R0	LSEI_1537	p-Ser-HPr phosphatase
25,0905	25,521	25,5801	25,5142	25,1725	23,7519	24,3452	23,7843	24,4387	24,4136	+	6	6	6	30,7	30,7	30,7	27,036	2,82E+08	16	24,7612	25,3757	24,1467	10	5	5	0,00016474	-1,22895	Q037U6	Q037U6	purC	Phosphoribosylaminoimidazole-succinocarboxamide synthase
22,7628	23,7348	23,9456	23,9235	23,4726	24,0108	24,0972	24,0383	24,448	24,2598	+	2	2	2	28,9	28,9	28,9	9,3206	1,04E+08	12	23,8693	23,5679	24,1708	10	5	5	0,032325	0,602969	Q034Q9	Q034Q9	LSEI_2592	Uncharacterized protein
24,4893	24,2773	24,1348	24,4648	24,1407	23,9588	24,1082	24,1507	24,515	24,1362		5	5	5	18,8	18,8	18,8	42,771	2,70E+08	34	24,2376	24,3014	24,1738	10	5	5	0,316278	-0,127615	Q037R6	Q037R6	LSEI_1785	Hydroxymethylglutaryl-CoA synthase
23,1787	23,7099	23,6838	24,1774	24,0163	24,1768	24,179	23,9754	24,3679	24,2034	+	3	3	3	19,4	19,4	19,4	13,781	1,50E+08	21	23,9668	23,7532	24,1805	10	5	5	0,0469938	0,427289	Q038C6	Q038C6	LSEI_1673	Predicted transcriptional regulator
24,5541	24,8334	24,5561	24,6616	24,6723	24,0239	24,2753	24,2738	24,2145	24,1162	+	12	12	12	29,8	29,8	29,8	62,753	5,23E+08	33	24,4181	24,6555	24,1807	10	5	5	0,000148652	-0,474746	Q03BY3	Q03BY3	LSEI_0433	Pyruvate oxidase
24,5853	23,9867	24,1706	24,1283	24,1544	24,3377	24,2947	23,976	24,2259	24,1234		5	5	5	27,6	27,6	27,6	29,429	2,47E+08	9	24,1983	24,2051	24,1915	10	5	5	0,91278	-0,0135235	Q03B81	Q03B81	LSEI_0701	ABC-type uncharacterized transport system, periplasmic component
24,3834	24,5532	24,2512	23,8748	24,1301	24,3366	24,1508	24,4413	24,0383	24,039		12	12	12	37	37	37	50,901	3,19E+08	33	24,2199	24,2386	24,2012	10	5	5	0,797363	-0,0373325	Q03AB7	Q03AB7	LSEI_1061	tRNA (Uracil-5-)-methyltransferase related enzyme
23,9269	24,1771	24,4677	24,375	24,1624	24,2972	23,9813	24,1859	24,3026	24,3041		3	3	3	14,7	14,7	14,7	25,746	1,59E+08	12	24,218	24,2218	24,2142	10	5	5	0,947781	-0,00762024	Q037Z9	Q037Z9	LSEI_1700	Uncharacterized protein
24,9758	24,3572	24,1411	24,4574	24,1863	23,8733	24,3653	24,2604	24,5063	24,1017		4	4	4	25,7	25,7	25,7	20,544	2,41E+08	23	24,3225	24,4236	24,2214	10	5	5	0,306588	-0,202187	Q036Q3	Q036Q3	efp	Elongation factor P
23,8743	24,4627	24,1183	24,4344	24,3336	24,2511	24,5304	24,2548	24,0636	24,0259		11	11	11	11,4	11,4	11,4	130,73	3,61E+08	23	24,2349	24,2447	24,2252	10	5	5	0,894394	-0,0195034	Q038J2	Q038J2	smc	Chromosome partition protein Smc
24,8276	24,8521	24,4378	24,6157	24,4915	24,1979	24,1517	24,3473	23,965	24,4788	+	5	5	5	24,1	24,1	24,1	36,184	3,21E+08	24	24,4365	24,6449	24,2281	10	5	5	0,0090563	-0,416782	Q03B60	Q03B60	LSEI_0725	NADPH:quinone reductase related Zn-dependent oxidoreductase
23,7297	24,222	24,477	24,2552	23,9914	24,2445	24,2713	24,2258	24,5152	23,9902		6	6	6	30,7	30,7	30,7	22,149	2,38E+08	20	24,1922	24,1351	24,2494	10	5	5	0,473824	0,114326	Q037V8	Q037V8	LSEI_1742	Anaerobic ribonucleoside-triphosphate reductase-activating protein
24,2031	24,3133	24,113	24,1653	23,9322	24,1761	24,1223	24,3342	24,3093	24,3176		9	9	9	29,1	29,1	29,1	45,374	3,60E+08	17	24,1987	24,1454	24,2519	10	5	5	0,198354	0,10652	Q03AV4	Q03AV4	LSEI_0861	1,2-diacylglycerol 3-glucosyltransferase
24,1222	23,7339	24,2017	24,1087	24,2879	24,031	24,3126	24,4657	24,2975	24,1655		5	5	5	24,3	24,3	24,3	33,518	2,60E+08	22	24,1727	24,0909	24,2545	10	5	5	0,209533	0,163612	Q037J0	Q037J0	LSEI_1873	ABC-type multidrug transport system, ATPase component
23,8126	24,4263	24,3145	24,5768	24,1299	24,1423	24,2113	24,0949	24,5446	24,2818		5	5	5	42,7	42,7	42,7	18,302	1,99E+08	20	24,2535	24,252	24,255	10	5	5	0,985099	0,00296173	Q03AJ7	Q03AJ7	smpB	SsrA-binding protein
24,2013	24,5121	24,434	24,2829	24,2133	24,0614	24,2488	24,2209	24,6421	24,1988		8	8	8	29,1	29,1	29,1	35,026	3,69E+08	32	24,3016	24,3288	24,2744	10	5	5	0,650082	-0,0543552	Q039J0	Q039J0	rnz	Ribonuclease Z
24,3146	24,5114	24,5725	24,6439	24,5259	24,2924	24,0062	24,1422	24,2243	24,8306		7	7	7	29,3	29,3	29,3	36,321	3,40E+08	31	24,4064	24,5137	24,2991	10	5	5	0,194346	-0,214552	Q035Y0	Q035Y0	tsaD	tRNA N6-adenosine threonylcarbamoyltransferase
24,4147	24,7516	24,5417	24,686	24,5724	24,4159	24,1542	24,1843	24,3956	24,4234	+	5	5	5	16,1	16,1	16,1	45,915	3,12E+08	23	24,454	24,5933	24,3147	10	5	5	0,0103947	-0,278572	Q036B9	Q036B9	LSEI_2206	Pyrimidine-nucleoside phosphorylase
24,3108	24,4375	24,1129	24,03	23,9977	24,0802	24,2883	24,2693	24,5419	24,4361		7	7	7	33,8	33,8	33,8	30,907	3,85E+08	28	24,2505	24,1778	24,3231	10	5	5	0,244121	0,145367	Q03AN4	Q03AN4	pstB	Phosphate import ATP-binding protein PstB
24,623	24,621	24,2962	24,4859	24,4707	23,9277	24,1185	24,2841	24,8254	24,4997		7	7	7	40,2	40,2	40,2	23,734	3,26E+08	24	24,4152	24,4994	24,3311	10	5	5	0,342093	-0,168275	Q035W9	Q035W9	tmk	Thymidylate kinase
24,2164	24,7842	24,3584	24,6761	24,4314	24,3212	24,0799	24,3778	24,5801	24,3061		5	5	5	32,1	32,1	32,1	18,388	3,10E+08	23	24,4132	24,4933	24,333	10	5	5	0,256911	-0,160295	Q03B43	Q03B43	LSEI_0742	Thiol peroxidase (Atypical 2-Cys peroxiredoxin)
24,5214	24,4242	24,3945	24,2524	24,4444	24,311	24,3786	24,379	24,291	24,3197		7	7	7	18,7	18,7	18,7	46,267	4,20E+08	36	24,3716	24,4074	24,3358	10	5	5	0,171633	-0,0715408	Q039J3	Q039J3	obg	GTPase Obg
24,2093	24,9018	24,8042	24,1507	24,5177	24,3772	24,3155	24,3757	24,6631	24,0161		5	5	5	42	42	42	19,585	2,01E+08	13	24,4331	24,5167	24,3495	10	5	5	0,388187	-0,167189	Q03AQ9	Q03AQ9	LSEI_0914	Putative tRNA (cytidine(34)-2-O)-methyltransferase
24,2895	24,6641	24,9795	24,9289	24,7731	24,3329	24,1073	24,3635	24,8685	24,1346		5	5	5	35	35	35	18,03	2,77E+08	22	24,5442	24,727	24,3614	10	5	5	0,0819242	-0,365657	Q03C15	Q03C15	LSEI_0401	Phosphotransferase system, mannose/fructose/N-acetylgalactosamine-specific component IIB
24,3821	24,4219	24,2376	24,4235	24,3231	24,3614	24,4345	24,2964	24,5071	24,2531		9	9	9	29,5	29,5	29,5	38,265	4,65E+08	31	24,3641	24,3576	24,3705	10	5	5	0,829266	0,0128746	Q03AR8	Q03AR8	LSEI_0904	FAD:protein FMN transferase
24,8666	24,7784	24,6361	24,577	24,6378	24,2935	24,2792	24,3772	24,5808	24,4238	+	7	7	7	11,8	11,8	11,8	63,943	3,43E+08	29	24,545	24,6992	24,3909	10	5	5	0,00372063	-0,308248	Q03A35	Q03A35	LSEI_1148	ABC-type multidrug transport system, ATPase and permease component
24,8628	24,4474	24,5631	24,5366	24,7138	24,448	24,362	24,2279	24,4493	24,4762	+	5	5	5	26	26	26	26,376	3,21E+08	13	24,5087	24,6248	24,3927	10	5	5	0,0275427	-0,23206	Q03AS1	Q03AS1	LSEI_0901	Uncharacterized protein, RelA_SpoT domain
24,4227	24,6315	24,5268	24,7575	24,5188	24,3322	24,5972	24,1303	24,3318	24,5775		17	17	17	25,6	25,6	25,6	84,916	7,82E+08	49	24,4826	24,5715	24,3938	10	5	5	0,126744	-0,17763	Q03AC4	Q03AC4	LSEI_1054	ATP-dependent DNA helicase
24,474	24,5598	24,3005	24,5196	24,3423	24,1862	24,4345	24,3458	24,2703	24,7963		7	7	7	15,6	15,6	15,6	50,431	3,54E+08	29	24,4229	24,4392	24,4066	10	5	5	0,787922	-0,0325825	Q03B04	Q03B04	cshB	DEAD-box ATP-dependent RNA helicase CshB
24,6464	23,8971	24,2843	24,4524	24,3968	24,6755	24,3989	24,5674	24,0221	24,4722		7	7	7	30,9	30,9	30,9	24,681	2,74E+08	15	24,3813	24,3354	24,4272	10	5	5	0,597243	0,0918213	Q039Q4	Q039Q4	LSEI_1285	5-bromo-4-chloroindolyl phosphate hydrolysis protein
24,6055	24,3255	24,3464	24,4471	24,4859	24,238	24,5031	24,4625	24,5226	24,4329		5	5	5	15,9	15,9	15,9	33,125	3,48E+08	33	24,4369	24,4421	24,4318	10	5	5	0,889878	-0,0102688	Q036T3	Q036T3	LSEI_2034	FAD:protein FMN transferase
24,7764	24,9963	24,9325	24,9006	24,8942	24,3735	24,4493	24,5348	24,3066	24,5289	+	6	6	6	22,4	22,4	22,4	36,469	3,83E+08	32	24,6693	24,9	24,4386	10	5	5	3,98E-05	-0,461394	Q03A04	Q03A04	LSEI_1181	Fe-S cluster assembly ABC-type transport system, permease component
24,6197	24,97	24,7519	24,7104	24,6571	24,4794	24,3938	24,6146	24,7132	24,0805		11	11	11	42,5	42,5	42,5	41,446	6,40E+08	45	24,5991	24,7418	24,4563	10	5	5	0,0515577	-0,285537	Q038A9	Q038A9	LSEI_1690	Predicted GTPase
24,8583	24,5499	24,3153	24,4322	24,2528	24,2615	24,3769	24,3622	24,7612	24,523		7	7	7	44	44	44	26,689	3,79E+08	22	24,4693	24,4817	24,4569	10	5	5	0,86193	-0,0247402	Q03AX4	Q03AX4	LSEI_0812	Biotin operon repressor
24,7162	24,8401	24,7886	24,8957	24,7861	24,2098	24,5015	24,4908	24,7073	24,3822	+	10	10	10	41,2	41,2	41,2	34,866	6,23E+08	47	24,6318	24,8053	24,4583	10	5	5	0,00394572	-0,347019	Q038Y9	Q038Y9	pyrB	Aspartate carbamoyltransferase
24,9698	24,6863	24,8095	24,835	24,4366	24,1304	24,4284	24,5747	24,5204	24,6704		8	8	8	27,6	27,6	27,6	44,298	4,57E+08	21	24,6061	24,7474	24,4649	10	5	5	0,0595902	-0,282588	Q03AQ1	Q03AQ1	cinA	Putative competence-damage inducible protein
25,4538	25,4134	25,3373	25,3307	25,359	24,1992	24,4083	24,6261	24,4805	24,6767	+	9	9	9	19,5	19,5	19,5	54,699	8,35E+08	55	24,9285	25,3788	24,4782	10	5	5	7,20E-06	-0,900693	Q037V3	Q037V3	purH	Bifunctional purine biosynthesis protein PurH
24,9098	24,6798	24,1608	24,1185	24,0438	24,5112	24,3358	24,5953	24,3592	24,5965		5	5	5	30,3	30,3	30,3	33,153	3,17E+08	19	24,4311	24,3826	24,4796	10	5	5	0,608518	0,0970596	Q03CD7	Q03CD7	LSEI_0275	Alpha/beta hydrolase superfamily protein
24,7299	24,9429	24,9085	24,7888	24,7201	24,4707	24,5898	24,3767	24,353	24,6089	+	19	19	19	22,9	22,9	22,9	124,92	9,35E+08	64	24,6489	24,818	24,4798	10	5	5	0,00128371	-0,338216	Q039M6	Q039M6	LSEI_1315	Pyruvate carboxylase
24,5274	24,5804	24,6406	24,6764	24,6821	24,1654	24,7619	24,5418	24,6873	24,2572		9	9	9	25,6	25,6	25,6	37,303	4,62E+08	42	24,5521	24,6214	24,4827	10	5	5	0,2846	-0,138669	Q03AB8	Q03AB8	LSEI_1060	Diacylglycerol kinase
23,8681	24,6639	24,5011	24,6066	24,2823	24,4042	24,2698	24,6359	24,8319	24,298		5	5	5	38,1	38,1	38,1	11,999	2,58E+08	17	24,4362	24,3844	24,4879	10	5	5	0,58117	0,103556	Q037Y5	Q037Y5	LSEI_1714	Thiol-disulfide isomerase and thioredoxin
24,3563	24,7436	24,5736	24,5305	24,6608	24,3792	24,5755	24,6162	24,581	24,3628		10	10	10	38,8	38,8	38,8	40,406	6,04E+08	38	24,5379	24,5729	24,5029	10	5	5	0,434267	-0,0700161	Q03CR7	Q03CR7	LSEI_0141	Microcin C7 resistance MccF related protein
24,0255	25,0127	24,8842	24,8316	24,6941	24,5193	24,5463	24,2833	24,6202	24,5715		4	4	4	43,1	43,1	43,1	13,431	3,27E+08	17	24,5989	24,6896	24,5081	10	5	5	0,351157	-0,181503	Q03B48	Q03B48	LSEI_0737	Uncharacterized protein
25,3051	24,39	24,4053	24,7815	24,3363	24,4169	24,4504	24,6784	24,4147	24,61		5	5	5	24,3	24,3	24,3	26,711	2,82E+08	14	24,5788	24,6436	24,5141	10	5	5	0,517212	-0,129577	Q03AN9	Q03AN9	LSEI_0934	DNA-binding response regulator, OmpR family (Rec-wHTH domains)
25,3032	25,0765	24,6587	24,7012	24,5047	24,367	24,3985	24,5497	24,9057	24,3994		10	10	10	32,5	32,5	32,5	28,544	4,90E+08	26	24,6864	24,8488	24,5241	10	5	5	0,106251	-0,324786	Q03BY0	Q03BY0	LSEI_0437	SAM-dependent methyltransferase
24,4246	24,1659	24,448	24,7147	24,6804	24,4253	24,5224	24,4846	24,5975	24,5963		4	4	4	19,9	19,9	19,9	33,948	2,02E+08	17	24,506	24,4867	24,5252	10	5	5	0,722725	0,0385216	Q03BA5	Q03BA5	LSEI_0677	Tagatose-6-phosphate kinase
24,6722	24,762	24,53	24,6628	24,5006	24,3919	24,6982	24,549	24,5676	24,4383		4	4	4	22,5	22,5	22,5	26,222	3,15E+08	14	24,5773	24,6255	24,529	10	5	5	0,218411	-0,0965321	Q035X8	Q035X8	LSEI_2250	Metal-dependent protease-like protein, putative molecular chaperone
24,6711	24,8459	24,8222	24,7529	24,6252	24,3037	24,4669	24,4725	24,6411	24,7948		10	10	10	19	19	19	69,902	6,19E+08	37	24,6396	24,7435	24,5358	10	5	5	0,0581859	-0,207681	Q033L1	Q033L1	mnmG	tRNA uridine 5-carboxymethylaminomethyl modification enzyme MnmG
24,4957	24,6675	24,6607	24,5461	24,3393	24,3719	24,4765	24,5276	24,899	24,4718		6	6	6	27	27	27	29,318	4,05E+08	25	24,5456	24,5419	24,5494	10	5	5	0,947047	0,00748482	Q03BH7	Q03BH7	LSEI_0601	Amino acid ABC transporter substrate-binding protein, PAAT family
24,8149	24,6976	24,5584	24,427	24,5391	24,4022	24,3932	24,5477	24,5991	24,8469		10	10	10	41,1	41,1	41,1	35,348	5,73E+08	40	24,5826	24,6074	24,5578	10	5	5	0,654412	-0,0495827	Q03AM8	Q03AM8	hprK	HPr kinase/phosphorylase
24,8892	24,5725	24,7428	24,5486	24,3489	24,4459	24,5649	24,5596	24,7096	24,577		10	10	10	35,6	35,6	35,6	40,982	5,10E+08	46	24,5959	24,6204	24,5714	10	5	5	0,640133	-0,048996	Q03AH0	Q03AH0	potA	Spermidine/putrescine import ATP-binding protein PotA
24,3564	24,7123	24,8225	24,9337	24,7364	24,5632	24,5814	24,5509	24,8442	24,3277		11	11	11	23,5	23,5	23,5	59,643	4,22E+08	33	24,6429	24,7123	24,5735	10	5	5	0,306411	-0,138787	Q03CN4	Q03CN4	LSEI_0175	ABC-type oligopeptide transport system, periplasmic component
23,586	23,6561	23,9101	23,8868	23,8339	24,6018	24,6241	24,6303	24,6337	24,3834	+	5	5	5	20,6	20,6	20,6	30,317	2,58E+08	15	24,1746	23,7746	24,5747	10	5	5	9,10E-06	0,800064	Q034N0	Q034N0	LSEI_2622	Beta-propeller domains of methanol dehydrogenase type
24,575	24,6374	24,8306	24,7725	24,5675	24,5614	24,4196	24,4001	24,7743	24,7266		3	3	3	15,3	15,3	15,3	24,695	2,54E+08	22	24,6265	24,6766	24,5764	10	5	5	0,314423	-0,100203	Q039Q1	Q039Q1	LSEI_1288	5-methylthioadenosine/S-adenosylhomocysteine nucleosidase
24,7388	24,7715	24,7652	24,6311	24,4089	24,5506	24,5138	24,7016	24,4437	24,7079		6	6	6	23	23	23	47,616	5,14E+08	16	24,6233	24,6631	24,5835	10	5	5	0,382697	-0,0795761	Q039P3	Q039P3	LSEI_1296	Acetyltransferase
24,2741	24,2718	24,3747	24,6985	24,5822	24,5084	24,6563	24,6915	24,663	24,4512		8	8	8	16,7	16,7	16,7	71,165	3,35E+08	20	24,5172	24,4402	24,5941	10	5	5	0,155964	0,153842	Q03C98	Q03C98	LSEI_0314	Cell division protein FtsI/penicillin-binding protein 2
24,9532	24,6038	24,665	24,6312	24,6784	24,5748	24,4467	24,8071	24,5413	24,6143		9	9	9	34,8	34,8	34,8	35,817	5,10E+08	39	24,6516	24,7063	24,5968	10	5	5	0,242001	-0,109502	Q03AW0	Q03AW0	LSEI_0826	Ferredoxin--NADP reductase
24,5614	24,6288	24,5795	24,4639	24,2332	24,4112	24,6847	24,5926	24,7101	24,6444		16	16	16	28,5	28,5	28,5	84,127	7,07E+08	51	24,551	24,4934	24,6086	10	5	5	0,227599	0,115236	Q037N9	Q037N9	LSEI_1814	ABC-type antimicrobial peptide transport system, ATPase component
24,5621	24,7523	24,8628	24,7289	24,2828	24,3244	24,6445	24,6834	24,9326	24,4641		7	7	7	23,3	23,3	23,3	40,38	4,67E+08	47	24,6238	24,6378	24,6098	10	5	5	0,851304	-0,0279675	Q03A62	Q03A62	LSEI_1119	5-(Carboxyamino)imidazole ribonucleotide synthase
24,6161	24,9448	24,7415	24,7477	24,6264	24,5216	24,6767	24,5647	24,6205	24,6832		12	12	12	17	17	17	94,97	6,16E+08	35	24,6743	24,7353	24,6134	10	5	5	0,106347	-0,121961	Q037Q1	Q037Q1	LSEI_1801	Cation transport ATPase
24,9687	25,1461	24,932	24,9548	24,9218	24,5151	24,7195	24,6124	24,8398	24,4089	+	9	9	9	34,9	34,9	34,9	28,476	4,64E+08	30	24,8019	24,9847	24,6192	10	5	5	0,0027882	-0,36553	Q034I3	Q034I3	LSEI_2670	Transcriptional regulator, IclR family
24,5966	24,7599	24,8672	24,7799	24,7591	24,6458	24,5332	24,5901	24,8692	24,5544		6	6	6	30,6	30,6	30,6	32,24	4,69E+08	26	24,6955	24,7525	24,6385	10	5	5	0,166272	-0,114003	Q03CI2	Q03CI2	LSEI_0227	6-phosphogluconate dehydrogenase (Decarboxylating)
23,279	23,9048	23,8407	23,8579	23,7095	24,5844	24,7897	24,6194	24,7059	24,4999	+	5	5	5	9,2	9,2	9,2	73,066	2,99E+08	23	24,1791	23,7184	24,6399	10	5	5	7,81E-05	0,921501	Q034H7	Q034H7	LSEI_2676	Uncharacterized protein
24,6286	24,8608	24,7568	24,7207	24,6965	24,7111	24,6088	24,5724	24,7772	24,5348		9	9	9	36,6	36,6	36,6	33,163	6,16E+08	49	24,6868	24,7327	24,6409	10	5	5	0,158617	-0,0918262	Q03AH7	Q03AH7	LSEI_0999	ABC-type uncharacterized transport system, ATPase component
24,7467	24,7966	24,8921	24,9986	24,9335	24,6459	24,6483	24,6731	24,6334	24,7278	+	8	8	8	18,8	18,8	18,8	59,113	5,65E+08	38	24,7696	24,8735	24,6657	10	5	5	0,00270154	-0,207785	Q03BC4	Q03BC4	LSEI_0657	ATPase component of ABC transporter with duplicated ATPase domains
24,675	25,0493	24,9347	24,9399	24,8045	24,6094	24,577	24,5926	24,9562	24,598		12	12	12	42,1	42,1	42,1	36,522	8,08E+08	49	24,7736	24,8807	24,6666	10	5	5	0,0584173	-0,214067	Q039F7	Q039F7	thyA	Thymidylate synthase
24,8391	24,5312	24,4089	24,3491	24,5007	24,497	24,5403	24,6924	24,9207	24,687		9	9	9	52,3	52,3	52,3	21,906	5,30E+08	27	24,5966	24,5258	24,6675	10	5	5	0,244211	0,141674	Q035W7	Q035W7	recR	Recombination protein RecR
24,8723	25,1798	25,0814	24,8177	24,9076	24,4303	24,6822	24,686	24,8656	24,6954	+	13	13	13	22,9	22,9	22,9	67,251	8,93E+08	70	24,8218	24,9718	24,6719	10	5	5	0,0151594	-0,299855	Q03CG3	Q03CG3	LSEI_0246	Oligopeptidase F, Metallo peptidase, MEROPS family M03B
25,0379	25,04	24,8544	24,779	24,8035	24,5075	24,7265	24,9173	24,5506	24,6854	+	24	24	24	34,1	34,1	34,1	84,544	9,98E+08	64	24,7902	24,9029	24,6774	10	5	5	0,0399825	-0,22552	Q038Q8	Q038Q8	LSEI_1539	(p)ppGpp synthase
25,2598	25,1103	24,7633	24,7909	24,5306	24,1798	24,5966	24,9107	24,8744	24,888		7	7	7	32	32	32	34,714	4,16E+08	26	24,7904	24,891	24,6899	10	5	5	0,32358	-0,20113	Q03CZ4	Q03CZ4	LSEI_0064	Periplasmic glycine betaine/choline-binding (Lipo)protein of an ABC-type transport system (Osmoprotectant binding protein)
24,6163	24,667	24,691	24,6357	24,7856	24,5315	24,7296	24,6985	24,7838	24,718		18	18	18	51,5	51,5	51,5	53,502	1,13E+09	69	24,6857	24,6791	24,6923	10	5	5	0,805998	0,0131641	Q035S0	Q035S0	cysS	Cysteine--tRNA ligase
24,6311	24,9942	24,6306	24,6863	24,5468	24,834	24,6733	24,9087	24,8636	24,3065		14	14	14	29,7	29,7	29,7	61,879	8,55E+08	72	24,7075	24,6978	24,7172	10	5	5	0,888682	0,0194416	Q03BF0	Q03BF0	LSEI_0630	Alpha, alpha-phosphotrehalase
25,006	24,9412	24,9646	25,0272	25,1343	24,7963	24,8035	24,8639	24,769	24,4229	+	13	13	13	29,5	29,5	29,5	64,455	9,60E+08	61	24,8729	25,0147	24,7311	10	5	5	0,0105612	-0,283551	Q039U1	Q039U1	ezrA	Septation ring formation regulator EzrA
24,3019	25,1689	25,1863	24,83	24,7122	24,7377	24,8746	24,9093	24,3429	24,8931		4	4	4	34,2	34,2	34,2	17,368	2,74E+08	19	24,7957	24,8399	24,7515	10	5	5	0,662828	-0,0883121	Q036K2	Q036K2	LSEI_2120	Transcriptional regulator, MarR family
23,9859	25,2147	25,1392	25,0084	24,9866	24,9476	25,0086	24,9292	24,9944	23,9782		7	7	7	52,6	52,6	52,6	17,311	4,68E+08	24	24,8193	24,867	24,7716	10	5	5	0,758489	-0,095369	Q03B31	Q03B31	LSEI_0755	Transcriptional regulator
24,9509	24,6346	24,9005	24,8354	24,752	24,5074	24,8054	24,8609	24,7175	24,9788		9	9	9	34,8	34,8	34,8	40,962	6,86E+08	40	24,7943	24,8147	24,774	10	5	5	0,685458	-0,0406712	Q03A29	Q03A29	prfA	Peptide chain release factor 1
25,2697	25,0536	25,0868	24,5737	25,2783	24,9263	24,571	24,7155	24,7005	24,9676		5	5	5	28,9	28,9	28,9	31,099	2,55E+08	17	24,9143	25,0524	24,7762	10	5	5	0,0993419	-0,276213	Q039Y0	Q039Y0	LSEI_1206	Histidinol-phosphatase

24,6525	24,9252	24,8793	24,8529	24,7982	24,7009	24,6883	24,9268	25,0973	24,5009		12	12	12	21,9	21,9	21,9	63,336	7,87E+08	51	24,8022	24,8216	24,7828	10	5	5	0,742016	-0,0387825	Q03BF4	Q03BF4	LSEI_0626	ABC-type multidrug transport system, ATPase and permease component
24,5385	24,6542	24,7316	24,6147	24,5631	24,6083	24,8531	24,8083	24,6454	25,0449		19	19	19	23,6	23,6	23,6	106,32	9,72E+08	71	24,7062	24,6204	24,792	10	5	5	0,0801506	0,171584	Q03AL6	Q03AL6	uvrA	UvrABC system protein A
24,7061	24,9297	24,7259	25,1134	25,0898	24,5188	24,6759	24,8039	25,1736	24,7971		8	8	8	27,3	27,3	27,3	30,984	5,22E+08	38	24,8534	24,913	24,7939	10	5	5	0,414592	-0,119127	Q03AH3	Q03AH3	murB	UDP-N-acetylenolpyruvoylglucosamine reductase
24,5727	24,7464	24,7382	24,7892	24,6404	24,6971	24,7975	24,9159	24,5606	25,0128		16	16	16	26,8	26,8	26,8	78,079	1,01E+09	69	24,7471	24,6974	24,7968	10	5	5	0,295727	0,0993935	Q039E3	Q039E3	topA	DNA topoisomerase 1
24,5792	25,0155	24,9757	24,8717	24,8603	24,6261	24,7718	24,7294	25,0226	24,855		6	6	6	15,8	15,8	15,8	37,039	3,03E+08	32	24,8307	24,8605	24,801	10	5	5	0,573064	-0,0595081	Q039Z7	Q039Z7	LSEI_1188	Uncharacterized protein
26,025	25,639	25,6121	25,2352	25,591	25,0317	25,1377	24,9421	25,1183	23,8138	+	6	6	6	24	24	24	34,894	5,50E+08	16	25,2146	25,6205	24,8087	10	5	5	0,0201117	-0,811728	Q034P1	Q034P1	LSEI_2611	Uncharacterized protein
25,1451	25,5374	25,201	25,2816	24,8131	25,0563	25,0988	25,2208	24,2758	24,3952		6	6	6	7,5	7,5	7,5	94,544	2,86E+08	17	25,0025	25,1956	24,8094	10	5	5	0,129278	-0,386256	Q03B11	Q03B11	LSEI_0775	Aldehyde-alcohol dehydrogenase
24,4961	24,79	24,9136	24,7639	24,726	24,5908	24,8962	24,7802	24,9562	24,8313		11	11	11	33,8	33,8	33,8	45,455	6,44E+08	53	24,7744	24,7379	24,8109	10	5	5	0,452549	0,0730179	Q03A97	Q03A97	LSEI_1082	PepS aminopeptidase, Metallo peptidase, MEROPS family M29
25,0023	25,3132	25,0256	24,7634	25,0352	24,887	24,9269	24,9884	24,5765	24,6801		21	21	21	31,2	31,2	31,2	87,706	1,04E+09	44	24,9199	25,0279	24,8118	10	5	5	0,102391	-0,216171	Q036X9	Q036X9	LSEI_1985	DNA helicase
24,8132	24,9581	24,9979	24,8099	24,7414	24,4873	24,9023	24,7129	25,0527	24,9464		4	4	4	11,6	11,6	11,6	46,541	4,76E+08	33	24,8422	24,8641	24,8203	10	5	5	0,703657	-0,0437622	Q03AI9	Q03AI9	LSEI_0987	Predicted hydrolase of the metallo-beta-lactamase superfamily
25,8558	25,2887	25,0453	25,1991	25,2553	25,0421	24,765	24,7925	25,1894	24,3164	+	10	10	10	59,1	59,1	59,1	25	1,06E+09	47	25,0749	25,3288	24,8211	10	5	5	0,0369498	-0,507764	Q03AL8	Q03AL8	LSEI_0955	Predicted hydrolase of HD superfamily
24,7705	25,152	25,1872	25,2075	24,9428	24,79	24,7229	24,7668	25,1713	24,7018		16	16	16	45,7	45,7	45,7	38,994	1,11E+09	93	24,9413	25,052	24,8306	10	5	5	0,105037	-0,221431	Q039R7	Q039R7	murG	UDP-N-acetylglucosamine--N-acetylmuramyl-(pentapeptide) pyrophosphoryl-undecaprenol N-acetylglucosamine transferase
24,5534	24,6037	24,687	24,6868	24,6846	24,6845	24,8169	24,8974	24,9476	24,8466	+	4	4	4	18,3	18,3	18,3	22,786	3,00E+08	24	24,7408	24,6431	24,8386	10	5	5	0,00574634	0,195482	Q03B55	Q03B55	lexA	LexA repressor
24,5575	24,8821	24,7945	24,3732	24,7274	24,9623	24,6986	24,8396	24,8188	24,8769		6	6	6	29,1	29,1	29,1	28,511	3,49E+08	24	24,7531	24,667	24,8392	10	5	5	0,124229	0,17227	Q03B92	Q03B92	LSEI_0690	Esterase/lipase
24,1996	24,4133	24,478	24,999	24,5975	24,727	24,6652	24,9012	24,8035	25,1015		4	4	4	24	24	24	16,193	1,96E+08	13	24,6886	24,5375	24,8397	10	5	5	0,0832259	0,30221	Q039Y9	Q039Y9	LSEI_1197	Uncharacterized protein
24,3641	24,2905	24,4613	24,4522	24,3663	24,4897	24,7601	25,0547	24,9236	25,0038	+	7	7	7	32,5	32,5	32,5	29,712	4,42E+08	29	24,6166	24,3869	24,8464	10	5	5	0,00263122	0,459503	Q033V3	Q033V3	LSEI_2805	YycI domain-containing protein
24,2222	24,9119	24,9758	24,9367	24,8804	24,6682	24,665	24,5979	24,9326	25,4048		7	7	7	44,3	44,3	44,3	18,129	4,70E+08	23	24,8195	24,7854	24,8537	10	5	5	0,74851	0,0682892	Q039M3	Q039M3	coaD	Phosphopantetheine adenylyltransferase
24,7374	24,784	24,9013	25,0582	24,9809	24,8286	24,7406	24,7597	24,9917	24,9634		8	8	8	30,1	30,1	30,1	33,251	3,97E+08	33	24,8746	24,8923	24,8568	10	5	5	0,664284	-0,035537	Q03CA2	Q03CA2	LSEI_0310	Ribose-binding protein
25,2888	25,3292	25,1556	24,8206	25,0841	24,7255	24,9334	25,0127	24,7778	24,893	+	17	17	17	30,8	30,8	30,8	82,695	7,49E+08	64	25,002	25,1356	24,8685	10	5	5	0,0335561	-0,267175	Q034N6	Q034N6	ppk	Polyphosphate kinase
23,5187	24,9902	24,9495	25,2362	25,1725	25,0588	24,9148	24,2849	25,0764	25,0762		5	5	5	48,9	48,9	48,9	15,274	4,05E+08	24	24,8278	24,7734	24,8822	10	5	5	0,76572	0,10879	Q036J5	Q036J5	LSEI_2127	Transcriptional regulator
25,6048	25,0823	25,3023	25,3049	25,353	24,8756	24,8139	24,9418	24,9779	24,807	+	8	8	8	42	42	42	28,151	6,59E+08	28	25,1063	25,3295	24,8832	10	5	5	0,0011061	-0,446245	Q03A05	Q03A05	LSEI_1180	Iron-regulated ABC transporter ATPase subunit SufC
24,7193	24,5699	24,7878	24,7229	24,7028	24,6955	24,9021	24,9049	24,9759	24,9883	+	8	8	8	29,2	29,2	29,2	29,638	4,69E+08	30	24,7969	24,7005	24,8933	10	5	5	0,0161807	0,192805	Q036U6	Q036U6	LSEI_2021	Amino acid ABC transporter substrate-binding protein, PAAT family
22,9289	25,0788	25,0884	25,2191	25,1895	24,717	25,0413	24,3584	25,3304	25,0351		4	4	4	36,7	36,7	36,7	11,764	4,64E+08	32	24,7987	24,7009	24,8965	10	5	5	0,690705	0,195523	Q035W3	Q035W3	LSEI_2265	Uncharacterized protein
24,8876	25,3511	25,4442	25,5059	25,3857	24,9387	24,832	24,5095	25,3799	24,8281	+	3	3	3	17	17	17	27,225	3,64E+08	22	25,1063	25,3149	24,8977	10	5	5	0,0474564	-0,417247	Q037P3	Q037P3	proC	Pyrroline-5-carboxylate reductase
24,0172	24,3715	24,3436	24,2826	24,0657	24,7963	25,1512	24,9865	25,1454	24,4114	+	2	2	2	7,2	7,2	7,2	32,296	2,04E+08	20	24,5572	24,2161	24,8982	10	5	5	0,00238142	0,68204	Q03BM0	Q03BM0	LSEI_0553	Uncharacterized protein
24,8333	25,0777	25,1686	24,972	24,9752	24,7484	24,6391	24,9284	25,1546	25,0441		6	6	6	26,3	26,3	26,3	28,816	4,54E+08	34	24,9541	25,0054	24,9029	10	5	5	0,377852	-0,102446	Q035X4	Q035X4	LSEI_2254	Acyl-ACP thioesterase
24,8947	25,3123	25,0968	25,1061	25,0709	24,8562	24,7686	25,0091	25,1054	24,8015		3	3	3	7,6	7,6	7,6	51,134	2,95E+08	13	25,0022	25,0962	24,9082	10	5	5	0,0762376	-0,188003	Q03AW7	Q03AW7	LSEI_0819	5-nucleotidase/2,3-cyclic phosphodiesterase related esterase
25,0247	25,3056	25,0592	24,9969	25,0592	24,7701	24,8309	24,8925	25,2292	24,8341		6	6	6	27,3	27,3	27,3	25,891	6,53E+08	50	25,0002	25,0891	24,9114	10	5	5	0,109601	-0,177752	Q03CD2	Q03CD2	deoD	Purine nucleoside phosphorylase DeoD-type
24,3922	24,6113	24,5506	24,6552	24,5735	24,8106	24,8934	24,9132	24,9136	25,0357	+	18	18	18	33,8	33,8	33,8	77,224	1,21E+09	83	24,7349	24,5566	24,9133	10	5	5	0,000256133	0,356726	Q038D7	Q038D7	LSEI_1662	Cell elongation-specific peptidoglycan D,D-transpeptidase
24,5009	24,5323	24,5931	24,5082	24,495	24,8865	24,8176	25,0203	24,7443	25,1128	+	5	5	5	24,8	24,8	24,8	32,494	4,53E+08	21	24,7211	24,5259	24,9163	10	5	5	0,000491623	0,390358	Q03B50	Q03B50	LSEI_0735	Uncharacterized protein
24,7164	24,7623	24,7363	24,744	24,5435	24,9959	24,7666	25,0459	24,9802	24,8657	+	7	7	7	38,4	38,4	38,4	27,425	3,68E+08	23	24,8157	24,7005	24,9308	10	5	5	0,00724331	0,23032	Q036U5	Q036U5	LSEI_2022	Amino acid ABC transporter ATP-binding protein, PAAT family
25,1148	25,1561	25,0721	25,3616	25,2388	24,3095	24,9724	24,9722	25,2753	25,142		6	6	6	47,8	47,8	47,8	17,44	3,27E+08	25	25,0615	25,1887	24,9343	10	5	5	0,181754	-0,254414	Q03AS9	Q03AS9	LSEI_0893	Glutathione peroxidase
24,9493	25,2163	25,1922	25,3884	25,2097	24,918	24,9781	24,9147	25,4516	24,4097		9	9	9	40,7	40,7	40,7	28,133	5,71E+08	43	25,0628	25,1912	24,9344	10	5	5	0,190211	-0,256796	Q039M9	Q039M9	LSEI_1312	Archaeal fructose-1,6-bisphosphatase related enzyme of inositol monophosphatase family
24,7999	24,911	24,9418	25,0755	24,9199	24,7818	24,8119	25,047	25,0016	25,0418		9	9	9	25,1	25,1	25,1	50,156	5,46E+08	41	24,9332	24,9296	24,9368	10	5	5	0,923489	0,00720406	Q033L0	Q033L0	mnmE	tRNA modification GTPase MnmE
24,7549	24,871	24,8188	24,855	25,0054	24,7135	24,6241	25,1228	25,3032	24,9348		7	7	7	24,9	24,9	24,9	41,685	5,73E+08	39	24,9003	24,861	24,9397	10	5	5	0,568738	0,0786766	Q039P7	Q039P7	mnmA	tRNA-specific 2-thiouridylase MnmA
26,365	25,7976	25,8922	25,9157	25,8261	24,7836	25,1948	24,9629	25,0803	24,734	+	9	9	9	40,4	40,4	40,4	21,149	9,56E+08	57	25,4552	25,9593	24,9511	10	5	5	7,27E-05	-1,00819	Q03D46	Q03D46	LSEI_0010	Single-stranded DNA-binding protein
25,7439	25,4842	25,5598	25,5977	25,4165	24,8506	25,0715	24,9665	25,0524	24,82	+	12	12	12	42,2	42,2	42,2	38,476	1,18E+09	75	25,2563	25,5604	24,9522	10	5	5	4,13E-05	-0,608231	Q038N1	Q038N1	hrcA	Heat-inducible transcription repressor HrcA
24,7985	24,7509	24,8754	25,095	24,9299	24,6588	24,9456	24,8953	25,285	25,0293		5	5	5	35,6	35,6	35,6	25,894	4,55E+08	27	24,9264	24,8899	24,9628	10	5	5	0,553168	0,0728558	Q03AT5	Q03AT5	LSEI_0886	Pseudouridine synthase
24,8701	25,0192	25,0029	25,1633	25,1815	24,7118	24,9089	24,9151	25,2691	25,0355		9	9	9	39,6	39,6	39,6	27,572	4,62E+08	43	25,0077	25,0474	24,9681	10	5	5	0,483185	-0,0793266	Q03CJ7	Q03CJ7	LSEI_0212	Chromosome segregation ATPase
25,3133	25,7412	25,4948	25,3184	25,3295	24,6975	24,88	25,195	24,9029	25,2008	+	10	10	10	22,9	22,9	22,9	64,351	9,63E+08	31	25,2073	25,4395	24,9752	10	5	5	0,0067003	-0,464217	Q03C00	Q03C00	LSEI_0416	Predicted oxidoreductase Myosin-crossreactive antigen ortholog
24,7839	24,9615	25,0214	24,842	24,9573	24,7915	25,0837	24,8969	25,2812	24,857		6	6	6	20	20	20	34,002	5,50E+08	30	24,9476	24,9132	24,9821	10	5	5	0,507275	0,0688507	Q038T2	Q038T2	era	GTPase Era
24,6624	24,8236	24,7499	24,9945	24,6997	24,9088	24,8268	24,9597	25,0773	25,1487	+	8	8	8	26	26	26	35,124	5,24E+08	42	24,8851	24,786	24,9842	10	5	5	0,0427325	0,198246	Q03C80	Q03C80	LSEI_0332	Ornithine cyclodeaminase
24,4734	24,9593	24,8205	24,6973	24,5739	25,0592	24,9349	24,9305	25,1422	24,8866	+	4	4	4	18,7	18,7	18,7	23,476	3,08E+08	22	24,8478	24,7049	24,9907	10	5	5	0,0198635	0,28581	Q039F2	Q039F2	LSEI_1392	Uncharacterized protein
25,1745	25,1859	24,907	25,0305	24,8817	24,8528	25,0325	24,9892	24,9389	25,2009		25	25	25	37,8	37,8	37,8	90,289	1,36E+09	84	25,0194	25,0359	25,0029	10	5	5	0,711557	-0,0330788	Q039D5	Q039D5	parC	DNA topoisomerase 4 subunit A
24,8566	25,1373	25,0135	25,1289	25,0627	24,6885	24,7704	25,0956	25,4752	25,0485		6	6	6	31,6	31,6	31,6	23,81	5,50E+08	33	25,0277	25,0398	25,0156	10	5	5	0,874342	-0,0241665	Q033Q1	Q033Q1	LSEI_2861	Acetyltransferase
25,0954	25,0088	25,0267	25,0332	25,3474	24,9729	24,93	24,8736	25,2754	25,0843		6	6	6	26,9	26,9	26,9	40,4	6,85E+08	49	25,0648	25,1023	25,0272	10	5	5	0,451828	-0,0750694	Q037Q9	Q037Q9	LSEI_1792	Probable succinyl-diaminopimelate desuccinylase
24,8738	25,0436	25,0553	24,9685	24,8738	24,784	25,1265	25,0345	25,282	25,0254		7	7	7	13,8	13,8	13,8	66,731	3,43E+08	32	25,0067	24,963	25,0505	10	5	5	0,359817	0,0874893	Q036X1	Q036X1	LSEI_1993	ABC-type antimicrobial peptide transport system, permease component
25,1155	25,114	25,0157	24,9449	25,327	25,0054	25,1063	24,9273	25,1314	25,0941		4	4	4	22	22	22	28,26	4,57E+08	23	25,0781	25,1034	25,0529	10	5	5	0,518134	-0,0505367	Q03CI5	Q03CI5	LSEI_0224	Aspartate racemase
25,6046	25,7751	25,6317	25,5012	25,432	25,1704	25,2705	25,3253	25,4412	24,0842		13	13	13	37,4	37,4	37,4	43,217	1,29E+09	60	25,3236	25,5889	25,0583	10	5	5	0,0703809	-0,53063	Q03B22	Q03B22	LSEI_0764	Methionine synthase II (Cobalamin-independent)
24,9917	25,1138	25,1264	25,1549	24,8873	25,1508	25,0111	25,0819	24,9687	25,1024		8	8	8	23,4	23,4	23,4	54,743	5,09E+08	29	25,0589	25,0548	25,063	10	5	5	0,895265	0,00814552	Q036H0	Q036H0	LSEI_2153	Membrane protease subunit, stomatin/prohibitin family
26,1814	25,6648	25,6101	25,7535	25,7417	24,879	25,0217	25,1128	25,1941	25,1194	+	4	4	4	29,2	29,2	29,2	18,926	4,77E+08	26	25,4278	25,7903	25,0654	10	5	5	0,000228213	-0,724912	Q037W3	Q037W3	LSEI_1737	Monooxygenase
25,7099	25,4875	25,3318	25,2573	25,2854	25,031	25,0415	25,139	25,255	24,8643	+	9	9	9	33,9	33,9	33,9	36,923	9,70E+08	58	25,2403	25,4144	25,0662	10	5	5	0,0110665	-0,348205	Q034W6	Q034W6	LSEI_2533	tRNA-dihydrouridine synthase
25,5126	25,539	25,3547	25,2708	25,2921	24,75	25,0181	25,0814	25,3657	25,1323	+	5	5	5	15,6	15,6	15,6	45,27	3,60E+08	26	25,2317	25,3939	25,0695	10	5	5	0,0214202	-0,324351	Q039U0	Q039U0	thiI	Probable tRNA sulfurtransferase
25,7601	25,7497	25,6873	25,6619	25,6963	24,965	25,083	25,1679	25,3288	24,8252	+	12	12	12	38,9	38,9	38,9	49,179	8,23E+08	57	25,3925	25,7111	25,074	10	5	5	8,86E-05	-0,637085	Q035V0	Q035V0	LSEI_2278	NAD-dependent aldehyde dehydrogenase
26,0412	25,0829	24,8776	24,5642	24,8219	25,6099	25,0208	25,0371	24,759	24,9548		4	4	4	30,5	30,5	30,5	26,799	2,87E+08	14	25,0769	25,0776	25,0763	10	5	5	0,996753	-0,0012249	Q033R4	Q033R4	LSEI_2848	Amino acid ABC transporter ATP-binding protein, PAAT family
24,3028	24,7789	24,6721	24,7864	24,4751	24,9247	25,0112	25,0473	25,0657	25,3392	+	14	14	14	28,1	28,1	28,1	74,851	7,67E+08	53	24,8403	24,603	25,0776	10	5	5	0,00362729	0,474559	Q037X4	Q037X4	LSEI_1725	DD-transpeptidase
25,5763	25,418	25,2132	25,2672	25,2898	24,9309	24,9621	24,8965	25,4383	25,1763		12	12	12	47,6	47,6	47,6	41,298	9,55E+08	60	25,2169	25,3529	25,0808	10	5	5	0,054546	-0,272092	Q034T9	Q034T9	alr	Alanine racemase
24,7018	25,116	25,0941	25,1289	24,9695	24,9132	25,1058	25,0033	25,3118	25,0984		19	19	19	42,1	42,1	42,1	53,788	1,37E+09	90	25,0443	25,0021	25,0865	10	5	5	0,441	0,0844414	Q03C90	Q03C90	LSEI_0322	Fumarate reductase, flavoprotein subunit
25,2397	25,0091	25,1086	25,1246	25,1539	25,1037	24,97	25,0653	25,2486	25,0787		6	6	6	40,7	40,7	40,7	23,014	4,89E+08	25	25,1102	25,1272	25,0933	10	5	5	0,577282	-0,0339001	Q03CD4	Q03CD4	deoC	Deoxyribose-phosphate aldolase
24,6276	25,1271	25,057	24,9778	25,0537	25,0605	25,0402	24,9313	25,3875	25,0592		7	7	7	36,6	36,6	36,6	25,897	5,70E+08	48	25,0322	24,9686	25,0957	10	5	5	0,309428	0,12711	Q037W0	Q037W0	LSEI_1740	DNA-binding response regulator, OmpR family (Rec-wHTH domains)
25,1535	25,3758	25,305	25,2944	25,1617	24,9904	25,0358	25,0759	25,3037	25,09		12	12	12	29,3	29,3	29,3	57,301	7,48E+08	48	25,1786	25,2581	25,0992	10	5	5	0,0508738	-0,158925	Q034N5	Q034N5	LSEI_2617	Exopolyphosphatase
24,63	25,17	25,1482	25,1653	25,0861	25,0438	25,0863	24,9551	25,314	25,1111		9	9	9	60,5	60,5	60,5	19,029	7,99E+08	52	25,071	25,0399	25,1021	10	5	5	0,616724	0,06213	Q038J9	Q038J9	rimM	Ribosome maturation factor RimM
25,3563	25,3889	25,3477	25,3838	25,5025	24,9064	25,0807	25,0529	25,3054	25,1789	+	8	8	8	28,6	28,6	28,6	37,799	1,05E+09	75	25,2504	25,3958	25,1049	10	5	5	0,00374278	-0,290984	Q036S6	Q036S6	LSEI_2041	Branched-chain-amino-acid aminotransferase
24,4113	25,2045	25,2512	25,5417	25,4414	25,299	24,6113	25,2862	25,5479	24,8286		4	4	4	32,4	32,4	32,4	15,231	5,14E+08	26	25,1423	25,17	25,1146	10	5	5	0,838358	-0,0553898	Q03AQ8	Q03AQ8	LSEI_0915	Uncharacterized protein
24,8627	25,1782	25,1041	25,2126	24,9252	24,8403	25,1324	25,1797	25,2126	25,2291		9	9	9	49,2	49,2	49,2	29,409	8,63E+08	45	25,0877	25,0566	25,1188	10	5	5	0,549547	0,0622574	Q03AH4	Q03AH4	LSEI_1002	Exodeoxyribonuclease III
24,8786	25,0637	25,0035	24,7439	25,1484	24,8232	25,0169	25,1785	25,3634	25,215		4	4	4	41	41	41	16,209	3,27E+08	21	25,0435	24,9676	25,1194	10	5	5	0,229017	0,151778	Q037T3	Q037T3	LSEI_1767	Acetyltransferase, GNAT family
23,6994	23,9785	24,1388	24,2257	23,9336	25,1559	25,2681	24,9606	25,1398	25,1872	+	5	5	5	13	13	13	61,82	2,97E+08	22	24,5688	23,9952	25,1423	10	5	5	4,06E-06	1,14712	Q03CC1	Q03CC1	LSEI_0291	Beta-N-acetylhexosaminidase
25,7687	24,9055	24,6853	24,5188	25,045	25,4421	24,7329	25,1244	25,1606	25,3101		5	5	5	24,4	24,4	24,4	29,092	5,02E+08	24	25,0693	24,9846	25,154	10	5	5	0,511693	0,169374	Q036L2	Q036L2	accD	Acetyl-coenzyme A carboxylase carboxyl transferase subunit beta
25,1078	25,1908	25,435	24,8993	25,0245	25,2377	24,9933	25,0931	25,178	25,3136		4	4	4	12,6	12,6	12,6	42,561	3,55E+08	18	25,1473	25,1315	25,1631	10	5	5	0,772355	0,0316532	Q03BB8	Q03BB8	galK	Galactokinase
25,3834	25,5564	25,4134	25,3846	25,3104	25,039	25,165	25,2157	25,3638	25,118	+	17	17	17	47	47	47	54,657	1,62E+09	88	25,295	25,4096	25,1803	10	5	5	0,00954932	-0,229342	Q037P6	Q037P6	LSEI_1806	Nicotinate phosphoribosyltransferase
25,4548	25,4405	25,3393	25,2924	25,6183	25,1748	25,2159	25,2338	25,3401	24,9448	+	7	7	7	38,6	38,6	38,6	13,363	7,24E+08	51	25,3055	25,4291	25,1819	10	5	5	0,0208768	-0,247178	Q035Z0	Q035Z0	LSEI_2237	Uncharacterized protein
23,3741	23,6822	24,3048	23,6845	23,5097	24,8479	25,213	25,1415	25,6178	25,1117	+	2	2	2	20,4	20,4	20,4	5,6366	2,23E+08	16	24,4487	23,7111	25,1864	10	5	5	8,40E-05	1,47532	Q035T6	Q035T6	rpmG2	50S ribosomal protein L33
25,1338	25,0807	25,3058	25,1985	25,2226	25,0428	25,1992	25,1449	25,273	25,2741		21	21	21	31,2	31,2	31,2	98,989	9,69E+08	53	25,1876	25,1883	25,1868	10	5	5	0,980001	-0,0015007	Q037Z0	Q037Z0	polA	DNA polymerase I
25,8972	25,6671	25,3673	25,5414	25,2953	25,1961	25,0368	24,9509	25,5385	25,2241	+	4	4	4	17,3	17,3	17,3	33,007	6,88E+08	23	25,3715	25,5537	25,1892	10	5	5	0,0387908	-0,364415	Q03AP1	Q03AP1	LSEI_0932	Cell division protein FtsX
26,1037	25,4746	25,5399	25,4212	25,4724	25,1787	25,4252	25,3663	25,0029	25,0183	+	13	13	13	31,9	31,9	31,9	59,458	8,33E+08	32	25,4003	25,6024	25,1983	10	5	5	0,0301452	-0,404092	Q037T6	Q037T6	prfC	Peptide chain release factor 3
25,4794	25,4144	25,2589	25,2927	25,2885	25,1071	25,1	24,9825	25,5522	25,2731		12	12	12	37,2	37,2	37,2	37,006	1,15E+09	44	25,2749	25,3468	25,203	10	5	5	0,218049	-0,143803	Q03AE8	Q03AE8	LSEI_1029	Adenine-specific DNA methylase
25,014	25,2053	25,2003	25,1502	25,1723	25,0638	25,1506	25,1198	25,454	25,2323		11	11	11	61	61	61	20,829	9,53E+08	28	25,1763	25,1484	25,2041	10	5	5	0,488152	0,0556953	Q037M0	Q037M0	LSEI_1843	Uncharacterized protein
25,8195	25,4693	25,6655	25,6959	25,8375	25,2904	25,1961	25,265	25,0082	25,3219	+	8	8	8	20,4	20,4	20,4	35,947	8,87E+08	35	25,4569	25,6975	25,2163	10	5	5	0,000540079	-0,481221	Q03BZ5	Q03BZ5	LSEI_0421	Dihydroxyacetone kinase DhaK subunit
25,3519	25,4977	25,2225	25,2325	25,3215	25,148	25,2172	25,2364	25,088	25,485		25	25	25	42	42	42	86,009	1,22E+09	86	25,2801	25,3252	25,2349	10	5	5	0,314424	-0,0903145	Q03AZ5	Q03AZ5	mutS2	Endonuclease MutS2
26,4204	25,7424	25,646	25,6068	25,1862	25,186	25,1834	25,2146	25,5799	25,0245		7	7	7	29,9	29,9	29,9	28,678	5,36E+08	24	25,479	25,7204	25,2377	10	5	5	0,0590277	-0,48269	Q036R8	Q036R8	LSEI_2049	Tyrosine-protein phosphatase
25,3013	25,5063	25,3412	25,5578	25,478	25,0625	24,93	25,1752	25,7573	25,2636		7	7	7	35,1	35,1	35,1	30,9	4,40E+08	23	25,3373	25,4369	25,2377	10	5	5	0,220108	-0,199212	Q038S5	Q038S5	LSEI_1522	Putative pyruvate, phosphate dikinase regulatory protein
25,3812	22,3464	22,4913	21,8655	22,0658	28,3466	24,6893	26,721	21,6931	24,7992		2	2	2	28,4	28,4	28,4	8,8648	3,57E+08	10	24,0399	22,83	25,2499	10	5	5	0,0975737	2,41985	Q03AZ0	Q03AZ0	dltC	D-alanyl carrier protein
25,1146	25,0384	25,1018	25,1608	24,9755	24,9933	25,2899	25,2528	25,4824	25,2528		8	8	8	36,2	36,2	36,2	31,496	6,89E+08	53	25,1662	25,0782	25,2543	10	5	5	0,0702968	0,176046	Q038S4	Q038S4	nfo	Probable endonuclease 4
25,5935	25,7779	25,5727	25,7231	25,8128	25,0592	25,218	25,2046	25,5177	25,2793	+	8	8	8	40	40	40	33,598	9,66E+08	44	25,4759	25,696	25,2558	10	5	5	0,00113045	-0,440221	Q03CL2	Q03CL2	LSEI_0197	Lactate dehydrogenase related enzyme
24,9067	25,3419	25,2465	25,3294	25,1061	25,0466	25,2236	25,2854	25,4565	25,3481		13	13	13	33,7	33,7	33,7	59,508	9,77E+08	55	25,2291	25,1861	25,272	10	5	5	0,442371	0,0859249	Q036Q4	Q036Q4	LSEI_2065	ABC-type oligopeptide transport system, periplasmic component
25,3195	25,379	25,4097	25,4343	25,3943	25,0657	25,3312	25,267	25,3665	25,4115		7	7	7	56,3	56,3	56,3	18,604	8,07E+08	33	25,3379	25,3874	25,2884	10	5	5	0,157361	-0,0990189	Q03AW1	Q03AW1	LSEI_0825	Phosphatidylglycerophosphatase A related protein
25,4909	25,5306	25,6642	25,6857	25,5818	25,1748	25,2152	25,3294	25,4953	25,2698	+	10	10	9	31,1	31,1	29	46,819	1,36E+09	57	25,4438	25,5906	25,2969	10	5	5	0,00241512	-0,293729	Q034M5	Q034M5	LSEI_2627	Glutathione reductase
24,3826	25,2813	25,2499	25,2413	24,986	25,039	25,2973	25,109	25,7221	25,3295		3	3	3	42,5	42,5	42,5	8,7186	3,13E+08	23	25,1638	25,0282	25,2994	10	5	5	0,227492	0,271174	Q03CL4	Q03CL4	LSEI_0195	Uncharacterized protein
26,6921	26,6595	26,6834	26,6239	26,2896	24,9757	25,3272	25,2455	25,7674	25,2115	+	9	9	9	32,8	32,8	32,8	44,046	8,15E+08	39	25,9476	26,5897	25,3055	10	5	5	2,68E-05	-1,28423	Q034K5	Q034K5	LSEI_2648	HTH containing DNA-binding domain and MocR-like aminotransferase
25,396	25,8411	25,8572	25,7674	25,5568	25,1109	25,5127	25,4145	25,799	24,7174		8	8	8	37,4	37,4	37,4	35,651	6,50E+08	32	25,4973	25,6837	25,3109	10	5	5	0,106888	-0,372802	Q033N0	Q033N0	LSEI_2884	Esterase/lipase
25,532	25,7932	25,4354	25,5345	25,386	25,2129	25,2609	25,3606	25,4148	25,3363	+	12	12	12	22,3	22,3	22,3	61,543	1,16E+09	68	25,4267	25,5362	25,3171	10	5	5	0,024078	-0,219108	Q039P1	Q039P1	rnj	Ribonuclease J
25,3649	25,3967	25,5664	25,4383	25,4484	25,2669	25,4389	25,3547	25,2096	25,3254	+	14	14	14	35,8	35,8	35,8	58,611	1,29E+09	55	25,381	25,4429	25,3191	10	5	5	0,0441245	-0,123838	Q03AP9	Q03AP9	rny	Ribonuclease Y
23,7743	24,8128	24,6681	24,9491	24,7957	25,1012	25,387	25,2012	25,4727	25,4766	+	4	4	4	32,9	32,9	32,9	9,2927	3,93E+08	55	24,9639	24,6	25,3277	10	5	5	0,0117895	0,727712	Q035M8	Q035M8	LSEI_2352	Uncharacterized protein
25,4721	25,4441	25,5651	25,6839	25,5124	25,0876	25,3149	25,3647	25,4834	25,4103	+	9	9	9	40,8	40,8	40,8	33,597	8,80E+08	39	25,4338	25,5355	25,3322	10	5	5	0,0335364	-0,203357	Q038W9	Q038W9	LSEI_1475	D-3-phosphoglycerate dehydrogenase
25,14	25,5016	25,6134	25,745	25,6299	25,1599	25,3145	25,2933	25,6849	25,2245		2	2	2	17,4	17,4	17,4	18,411	4,24E+08	14	25,4307	25,526	25,3354	10	5	5	0,206098	-0,190535	Q038P2	Q038P2	LSEI_1556	Cys-tRNA(Pro)/Cys-tRNA(Cys) deacylase
25,5429	25,8135	25,7245	25,598	25,579	25,1247	25,4792	25,4688	25,312	25,3151	+	6	6	6	16,8	16,8	16,8	56,736	6,31E+08	35	25,4958	25,6516	25,34	10	5	5	0,00531486	-0,311609	Q039I4	Q039I4	LSEI_1360	23-cyclic-nucleotide 2-phosphodiesterase/3-nucleotidase
24,7353	25,2016	25,0106	24,9937	25,0111	25,1046	25,4064	25,1826	25,669	25,3389	+	9	9	9	39,2	39,2	39,2	22,585	7,12E+08	43	25,1654	24,9905	25,3403	10	5	5	0,021794	0,349841	Q03CF5	Q03CF5	LSEI_0254	Signal peptidase I
25,4022	25,347	25,2837	25,4336	25,3745	25,2748	25,2444	25,2097	25,6848	25,3268		9	9	9	40,9	40,9	40,9	27,726	7,90E+08	52	25,3581	25,3682	25,3481	10	5	5	0,828647	-0,0201382	Q035S2	Q035S2	LSEI_2306	rRNA methylase
26,0609	25,8918	25,7281	25,6315	25,6323	25,3197	25,2021	25,4855	25,484	25,2535	+	19	19	19	56,1	56,1	56,1	46,54	1,62E+09	84	25,5689	25,7889	25,349	10	5	5	0,00249588	-0,439935	Q03CZ2	Q03CZ2	LSEI_0066	ABC-type proline/glycine betaine transport system, ATPase component
25,1007	25,7126	25,579	25,4443	25,4902	25,2438	25,5794	25,3801	25,7032	24,8658		7	7	7	42,5	42,5	42,5	21,202	6,38E+08	43	25,4099	25,4654	25,3545	10	5	5	0,550174	-0,110871	Q037H8	Q037H8	LSEI_1885	Acetyltransferase, including N-acetylase of ribosomal protein
25,3125	25,5333	25,4532	25,4719	25,3997	25,2176	25,2889	25,3182	25,532	25,4497		14	14	14	46,7	46,7	46,7	42,173	1,09E+09	56	25,3977	25,4341	25,3613	10	5	5	0,314846	-0,0728424	Q038C9	Q038C9	mltG	Endolytic murein transglycosylase
24,4305	25,2091	25,1589	25,1007	24,8738	25,1012	25,3941	25,3267	25,5377	25,4857	+	3	3	3	26,5	26,5	26,5	18,049	3,20E+08	16	25,1618	24,9546	25,3691	10	5	5	0,033777	0,41448	Q037Z3	Q037Z3	nrdR	Transcriptional repressor NrdR
25,5756	25,407	25,3576	25,5231	25,5422	25,1279	25,5073	25,3818	25,6999	25,1304		8	8	8	27,8	27,8	27,8	34,74	8,52E+08	47	25,4253	25,4811	25,3694	10	5	5	0,372116	-0,111664	Q03AA3	Q03AA3	LSEI_1075	Predicted dehydrogenase related protein
25,5696	25,5756	25,2423	25,4699	25,5189	25,1041	25,2981	25,3239	25,7012	25,4691		12	12	12	53,4	53,4	53,4	38,132	1,10E+09	65	25,4273	25,4753	25,3793	10	5	5	0,43432	-0,096035	Q03CV7	Q03CV7	asd	Aspartate-semialdehyde dehydrogenase
25,2281	25,6212	25,7516	25,709	25,6119	25,1025	25,2479	25,2822	25,8466	25,4657		7	7	7	34,5	34,5	34,5	30,683	8,44E+08	32	25,4867	25,5843	25,389	10	5	5	0,252219	-0,195361	Q03CG4	Q03CG4	LSEI_0245	3-hydroxyisobutyrate dehydrogenase related beta-hydroxyacid dehydrogenase
25,2469	25,4228	25,4158	25,422	25,3299	25,317	25,3453	25,352	25,433	25,5165		23	23	23	37	37	37	74,465	2,10E+09	148	25,3801	25,3675	25,3928	10	5	5	0,629743	0,0252857	Q039D6	Q039D6	parE	DNA topoisomerase 4 subunit B
25,3094	25,7103	25,5022	25,7155	25,6371	25,461	25,39	25,5328	25,2326	25,4175		16	16	16	35,4	35,4	35,4	73,594	1,45E+09	80	25,4908	25,5749	25,4068	10	5	5	0,1033	-0,168127	Q037P2	Q037P2	LSEI_1810	Dipeptidyl aminopeptidase/acylaminoacyl-peptidase
25,1238	25,7417	25,5851	25,7766	25,5668	25,2221	25,4669	25,3313	25,6478	25,3696		6	6	6	36,6	36,6	36,6	17,348	8,66E+08	54	25,4832	25,5588	25,4075	10	5	5	0,300605	-0,151281	Q033Y2	Q033Y2	LSEI_2776	Phosphotransferase system, mannose/fructose/N-acetylgalactosamine-specific component IIB
25,9716	25,8108	25,6879	25,8013	25,7306	25,3109	25,365	25,4746	25,4637	25,4284	+	18	18	18	20,9	20,9	20,9	116,93	1,62E+09	67	25,6045	25,8004	25,4085	10	5	5	0,000135845	-0,391904	Q03CZ8	Q03CZ8	LSEI_0060	Predicted membrane protein
25,0164	25,7697	25,5943	25,8224	25,7123	25,3686	25,2922	25,2521	25,6178	25,525		6	6	6	34,4	34,4	34,4	20,506	5,57E+08	34	25,4971	25,583	25,4111	10	5	5	0,320589	-0,17188	Q035Q3	Q035Q3	LSEI_2325	dUTP diphosphatase
24,4393	25,2478	25,2084	25,4992	25,3797	25,2739	25,2201	25,1597	25,9694	25,4383		7	7	7	35,9	35,9	35,9	22,011	6,01E+08	32	25,2836	25,1549	25,4123	10	5	5	0,309159	0,257383	Q036J1	Q036J1	LSEI_2131	Transcriptional regulator
23,8446	24,1945	24,3005	24,3054	24,3903	25,1224	25,5117	25,1556	25,6236	25,6627	+	4	4	4	27,2	27,2	27,2	22,376	2,38E+08	17	24,8111	24,2071	25,4152	10	5	5	4,18E-05	1,20815	Q034G0	Q034G0	LSEI_2694	Uncharacterized protein
24,3024	24,0459	24,4004	24,3567	24,2418	25,3267	25,6183	25,651	24,8998	25,6186	+	17	17	17	10,8	10,8	10,8	200,11	7,14E+08	45	24,8461	24,2694	25,4229	10	5	5	7,73E-05	1,15346	Q035V8	Q035V8	LSEI_2270	Lactocepin I, Serine peptidase, MEROPS family S08A
26,5544	25,8414	25,8436	26,0242	25,529	25,8298	25,5085	25,3331	25,4363	25,0168	+	8	8	8	63,6	63,6	63,6	20,809	1,09E+09	34	25,6917	25,9585	25,4249	10	5	5	0,0373845	-0,53363	Q035T8	Q035T8	nusG	Transcription termination/antitermination protein NusG
24,9255	25,0659	25,0797	25,0577	25,3145	25,7479	25,3453	25,3475	25,385	25,3485	+	3	3	3	12,8	12,8	12,8	46,575	3,70E+08	20	25,2617	25,0887	25,4348	10	5	5	0,00884828	0,346178	Q03AS5	Q03AS5	LSEI_0897	UPF0210 protein LSEI_0897
25,4899	25,5812	25,592	25,509	25,5869	25,382	25,4172	25,2836	25,5553	25,5419		12	12	12	39,8	39,8	39,8	50,603	1,42E+09	73	25,4939	25,5518	25,436	10	5	5	0,069915	-0,1158	Q034T6	Q034T6	murF	UDP-N-acetylmuramoyl-tripeptide--D-alanyl-D-alanine ligase
24,7764	25,386	25,3899	25,4431	25,3166	25,3388	25,3737	25,4665	25,6395	25,3643		12	12	12	49,3	49,3	49,3	30,535	1,03E+09	78	25,3495	25,2624	25,4365	10	5	5	0,232882	0,174156	Q039T2	Q039T2	LSEI_1256	Cell shape-determining protein MreC
25,4954	25,5972	25,8433	25,8756	25,8051	25,1478	25,3458	25,4497	25,7995	25,486		5	5	5	28,2	28,2	28,2	27,204	8,24E+08	31	25,5845	25,7233	25,4458	10	5	5	0,0651687	-0,277564	Q033S3	Q033S3	LSEI_2838	Amino acid ABC transporter ATP-binding protein, PAAT family
25,91	26,1346	26,017	26,0555	25,9198	25,3355	25,5214	25,47	25,4532	25,474	+	23	23	23	34,5	34,5	34,5	82,299	2,27E+09	136	25,7291	26,0074	25,4508	10	5	5	5,41E-06	-0,556616	Q035U1	Q035U1	rtpR	Adenosylcobalamin-dependent ribonucleoside-triphosphate reductase
25,4526	25,5942	25,7242	25,8181	25,6951	25,2887	25,4629	25,4908	26,0666	24,956		6	6	6	30,6	30,6	30,6	27,389	5,31E+08	26	25,5549	25,6568	25,453	10	5	5	0,317018	-0,203839	Q039Y1	Q039Y1	LSEI_1205	Deoxynucleoside kinase
25,6202	25,7228	25,6196	25,8024	25,7341	25,4331	25,2534	25,4049	25,7823	25,4499	+	8	8	8	34,3	34,3	34,3	26,803	8,96E+08	40	25,5823	25,6998	25,4647	10	5	5	0,0363131	-0,235106	Q037W4	Q037W4	LSEI_1736	Metal-dependent hydrolase of the beta-lactamase superfamily III
25,5266	25,5992	25,5741	25,7121	25,6179	25,3469	25,4373	25,5003	25,5449	25,5198	+	8	8	8	25,4	25,4	25,4	51,986	8,73E+08	43	25,5379	25,606	25,4699	10	5	5	0,0198187	-0,136132	Q03AY7	Q03AY7	murI	Multifunctional fusion protein
25,409	25,5949	25,6178	25,6389	25,3311	25,2657	25,8176	25,1989	25,513	25,5898		10	10	10	33	33	33	38,541	6,65E+08	37	25,4977	25,5183	25,477	10	5	5	0,755603	-0,0413342	Q035T3	Q035T3	LSEI_2295	FAD:protein FMN transferase
23,9275	24,3672	24,4672	24,7099	24,5617	25,5382	25,4084	25,3205	25,5767	25,5575	+	6	6	6	21	21	21	42,035	3,52E+08	15	24,9435	24,4067	25,4802	10	5	5	6,36E-05	1,07353	Q037Q0	Q037Q0	LSEI_1802	Beta-lactamase class C related penicillin binding protein
25,5412	25,745	25,5881	25,6909	25,5463	25,366	25,4347	25,3853	25,6755	25,61		11	11	11	48,6	48,6	48,6	31,201	1,53E+09	92	25,5583	25,6223	25,4943	10	5	5	0,124741	-0,127962	Q034R4	Q034R4	LSEI_2587	Pur operon repressor
24,1853	25,0487	24,8415	25,4497	24,9242	25,1313	25,2842	25,3497	25,9943	25,7266	+	4	4	4	77,9	77,9	77,9	7,8408	4,19E+08	18	25,1935	24,8899	25,4972	10	5	5	0,0469074	0,607319	Q03CL8	Q03CL8	LSEI_0191	Uncharacterized protein
25,6507	25,6279	25,4379	25,5132	25,5026	25,3627	25,5052	25,3694	25,7287	25,5275		12	12	12	51,8	51,8	51,8	34,66	1,04E+09	84	25,5226	25,5465	25,4987	10	5	5	0,556749	-0,0477608	Q036U9	Q036U9	LSEI_2018	Nucleoside-diphosphate-sugar epimerase
26,5181	26,1034	25,6397	25,7951	25,5763	25,4322	25,3253	25,6603	25,6493	25,4396		10	10	10	44,8	44,8	44,8	31,95	9,57E+08	48	25,7139	25,9265	25,5013	10	5	5	0,0513625	-0,425197	Q039A6	Q039A6	LSEI_1438	Uncharacterized protein
25,842	26,1293	25,7666	25,7442	25,6441	25,4301	25,3864	25,5565	25,5589	25,5985	+	12	12	12	27,4	27,4	27,4	67,913	1,29E+09	63	25,6656	25,8252	25,5061	10	5	5	0,00848255	-0,319173	Q03B41	Q03B41	LSEI_0745	Oligopeptidase F, Metallo peptidase, MEROPS family M03B
25,5641	25,7304	25,6513	25,6512	25,5964	25,5751	25,4169	25,5971	25,3187	25,6301		18	18	18	30,3	30,3	30,3	82,333	1,42E+09	77	25,5731	25,6387	25,5076	10	5	5	0,0828811	-0,131116	Q03CS7	Q03CS7	LSEI_0131	Ribonucleoside-triphosphate reductase class III catalytic subunit / ribonucleoside-triphosphate reductase
24,8477	24,1398	23,9729	23,7714	23,6767	25,9056	25,1565	24,6486	24,512	27,333	+	6	6	6	28,5	28,5	28,5	31,234	2,02E+08	13	24,7964	24,0817	25,5112	10	5	5	0,0333187	1,42946	Q035B3	Q035B3	ecfA2	Energy-coupling factor transporter ATP-binding protein EcfA2
25,2817	25,4984	25,3408	25,3689	25,4402	25,4707	25,408	25,3358	25,7155	25,6275		6	6	6	33,9	33,9	33,9	26,04	6,17E+08	44	25,4487	25,386	25,5115	10	5	5	0,153965	0,12552	Q038U6	Q038U6	LSEI_1500	Predicted SAM-dependent methyltransferase
25,4908	25,8507	25,8043	25,709	25,6729	25,3734	25,3683	25,5522	25,8314	25,4548		12	12	12	32,9	32,9	32,9	48,659	1,31E+09	66	25,6108	25,7055	25,516	10	5	5	0,111567	-0,189522	Q036H5	Q036H5	LSEI_2148	Predicted ATP-grasp enzyme
25,4565	25,3522	25,4326	25,3497	25,6191	24,9961	25,5736	25,6991	25,4538	25,8771		7	7	7	20	20	20	57,298	5,49E+08	29	25,481	25,442	25,5199	10	5	5	0,631868	0,0779202	Q035G7	Q035G7	LSEI_2417	Putative multicopper oxidase
25,4645	25,7683	25,8249	25,6601	25,5715	25,2176	25,5812	25,5391	25,9111	25,4224		8	8	8	46,6	46,6	46,6	23,734	5,85E+08	34	25,5961	25,6579	25,5343	10	5	5	0,372296	-0,123571	Q038G9	Q038G9	gmk	Guanylate kinase

25,1747	25,5179	25,5223	25,6934	25,5798	25,3416	25,3518	25,5859	25,9418	25,4543		5	5	5	31,3	31,3	31,3	23,734	4,99E+08	21	25,5163	25,4976	25,5351	10	5	5	0,796849	0,0374493	Q03B09	Q03B09	LSEI_0777	Mn-dependent transcriptional regulator
25,6404	25,5551	25,5464	25,6407	25,608	25,3672	25,4032	25,5542	25,7121	25,664		16	16	16	56,1	56,1	56,1	43,099	1,32E+09	66	25,5691	25,5981	25,5401	10	5	5	0,440298	-0,0579861	Q03CD3	Q03CD3	deoB	Phosphopentomutase
25,3926	25,8163	25,6034	25,713	25,7897	25,5043	25,6823	25,3573	25,7809	25,3876		7	7	7	50	50	50	21,614	8,47E+08	64	25,6027	25,663	25,5425	10	5	5	0,316743	-0,120539	Q03CK2	Q03CK2	LSEI_0207	Predicted dinucleotide-binding enzyme
25,3181	25,0802	25,0753	25,2182	25,4112	25,3619	25,6201	25,6393	25,5109	25,5965	+	14	14	14	57,4	57,4	57,4	27,098	1,01E+09	58	25,3832	25,2206	25,5457	10	5	5	0,00450377	0,325137	Q033V0	Q033V0	LSEI_2808	DNA-binding response regulator, OmpR family (Rec-wHTH domains)
26,8212	26,5636	26,3972	26,4775	26,3753	25,6982	25,731	25,5204	25,8505	24,9835	+	8	8	8	15,8	15,8	15,8	64,785	1,38E+09	42	26,0418	26,5269	25,5567	10	5	5	0,000500396	-0,970199	Q033V9	Q033V9	LSEI_2799	Cation transport ATPase
25,2441	25,7706	25,7564	25,7762	25,6587	25,5059	25,5791	25,3802	25,8635	25,4789		6	6	6	30,8	30,8	30,8	29,539	7,58E+08	25	25,6013	25,6412	25,5615	10	5	5	0,558368	-0,0796875	Q03B87	Q03B87	LSEI_0695	Hydroxymethylpyrimidine/phosphomethylpyrimidine kinase
25,538	25,8294	25,4934	25,4262	25,4252	25,5871	25,5837	25,6633	25,4043	25,5865		24	24	24	41	41	41	71,514	1,97E+09	114	25,5537	25,5424	25,565	10	5	5	0,800431	0,0225414	Q038X2	Q038X2	LSEI_1472	Oligopeptidase O2, Metallo peptidase, MEROPS family M13
25,4185	25,8668	25,8579	25,7446	25,8506	25,429	25,4659	25,5668	25,8627	25,58		4	4	4	11,4	11,4	11,4	35,005	4,84E+08	27	25,6643	25,7477	25,5809	10	5	5	0,182541	-0,166807	Q035X5	Q035X5	LSEI_2253	Asparaginase
25,7384	25,6134	25,693	25,6143	25,7971	25,4826	25,4876	25,5826	25,7515	25,6114		12	12	12	49	49	49	44,585	8,80E+08	51	25,6372	25,6912	25,5831	10	5	5	0,113004	-0,108108	Q03A26	Q03A26	glyA	Serine hydroxymethyltransferase
25,1803	25,3222	25,5632	25,4838	25,5867	25,7587	25,6571	25,7547	25,0673	25,6977		17	7	7	48,6	29,1	29,1	36,424	6,92E+08	30	25,5072	25,4272	25,5871	10	5	5	0,324635	0,159842	Q03BA4	Q03BA4	lacD	Tagatose 1,6-diphosphate aldolase
25,5615	25,781	25,5753	25,4522	25,5365	25,4751	25,4567	25,589	25,5612	25,8583		18	18	18	30,9	30,9	30,9	84,224	1,12E+09	60	25,5847	25,5813	25,5881	10	5	5	0,94204	0,00676727	Q039P8	Q039P8	LSEI_1291	Excinuclease ATPase subunit
25,6402	25,431	25,4912	25,6238	25,6565	25,627	25,3982	25,4951	25,669	25,7817		9	9	9	27,5	27,5	27,5	35,038	8,78E+08	50	25,5814	25,5685	25,5942	10	5	5	0,759475	0,0256313	Q03BC7	Q03BC7	LSEI_0654	Predicted iron-dependent peroxidase
25,5765	25,8173	25,6301	25,8072	25,6242	25,417	25,4977	25,5712	25,8245	25,7749		6	6	6	22,7	22,7	22,7	44,565	7,05E+08	38	25,6541	25,6911	25,617	10	5	5	0,451794	-0,0740036	Q038E4	Q038E4	LSEI_1655	Cystathionine beta-lyase family protein
25,6807	26,3252	26,7725	26,6068	26,6346	25,5287	25,6996	25,4928	25,9784	25,3926	+	4	4	4	33	33	33	13,382	7,21E+08	23	26,0112	26,404	25,6184	10	5	5	0,00733432	-0,785572	Q03B35	Q03B35	LSEI_0751	Uncharacterized protein
24,8561	24,7884	24,6591	24,7745	24,772	25,6906	25,5935	25,7529	25,4363	25,6759	+	11	11	11	33,2	33,2	33,2	38,21	6,96E+08	36	25,1999	24,77	25,6298	10	5	5	8,16E-07	0,859823	Q03AF9	Q03AF9	LSEI_1017	Uncharacterized protein
25,0445	25,4382	25,5497	25,6372	25,1335	25,2441	25,5714	25,7377	25,9902	25,6465		5	5	5	15,2	15,2	15,2	41,856	6,23E+08	33	25,4993	25,3606	25,638	10	5	5	0,137065	0,277335	Q039Q0	Q039Q0	LSEI_1289	Cysteine desulfurase
25,5235	25,8362	25,717	25,6899	25,602	25,4357	25,7295	25,6161	25,7678	25,6591		19	19	19	33,7	33,7	33,7	76,714	9,13E+08	66	25,6577	25,6737	25,6417	10	5	5	0,693743	-0,0320538	Q03AL7	Q03AL7	uvrB	UvrABC system protein B
25,3478	26,0523	25,6399	25,709	25,4247	25,3988	25,5057	25,5447	26,1008	25,6752		6	6	6	14,1	14,1	14,1	48,221	8,06E+08	53	25,6399	25,6347	25,645	10	5	5	0,95425	0,0102959	Q035A3	Q035A3	secY	Protein translocase subunit SecY
25,4984	25,4932	25,5171	25,4797	25,3496	25,5171	25,638	25,5633	25,7903	25,7351	+	16	16	16	30,4	30,4	30,4	60,153	1,54E+09	96	25,5582	25,4676	25,6488	10	5	5	0,015712	0,181183	Q039X9	Q039X9	LSEI_1207	ABC-type oligopeptide transport system, periplasmic component
23,8998	25,5946	25,4116	25,7262	25,5053	25,201	25,7457	25,521	26,1279	25,6566		3	3	3	32	32	32	11,001	5,08E+08	37	25,439	25,2275	25,6505	10	5	5	0,283972	0,422969	Q037T7	Q037T7	LSEI_1763	Uncharacterized protein
25,4059	25,5771	25,7926	25,7295	25,5999	25,6656	25,6059	25,6245	25,7353	25,6218		6	6	6	34,1	34,1	34,1	27,125	5,25E+08	23	25,6358	25,621	25,6506	10	5	5	0,687469	0,0296135	Q036X0	Q036X0	LSEI_1994	ABC-type antimicrobial peptide transport system, ATPase component
25,4892	25,8417	25,9682	25,7604	25,7731	25,5309	25,6864	25,6301	25,7603	25,7175		16	16	16	36,8	36,8	36,8	56,024	1,36E+09	83	25,7158	25,7665	25,665	10	5	5	0,281913	-0,101509	Q03B08	Q03B08	zwf	Glucose-6-phosphate 1-dehydrogenase
25,6146	25,6425	25,4966	25,4924	25,4302	25,4778	25,5739	25,6	25,8845	25,7928		8	8	8	22,7	22,7	22,7	47,781	8,23E+08	38	25,6005	25,5353	25,6658	10	5	5	0,162763	0,130536	Q03AY9	Q03AY9	LSEI_0797	Protein DltD
25,8631	25,813	25,7921	25,7136	25,5443	25,7351	25,7785	25,71	25,7608	25,3499		14	14	12	29,3	29,3	27,7	52,409	1,52E+09	70	25,706	25,7452	25,6669	10	5	5	0,444997	-0,0783604	Q033R3	Q033R3	LSEI_2849	Amino acid ABC transporter substrate-binding protein, PAAT family / amino acid ABC transporter membrane protein, PAAT family
25,2946	25,5633	25,5416	25,5756	25,5475	25,4428	25,525	25,6604	25,9508	25,7591		10	10	10	55,1	55,1	55,1	25,958	1,13E+09	56	25,5861	25,5045	25,6676	10	5	5	0,154605	0,163094	Q037M3	Q037M3	LSEI_1840	Alpha-acetolactate decarboxylase
25,5905	25,7169	25,7558	25,5847	25,6004	25,5273	25,7024	25,6113	25,6161	25,8894		7	7	7	19,7	19,7	19,7	58,177	1,04E+09	35	25,6595	25,6496	25,6693	10	5	5	0,789897	0,0196606	Q033S4	Q033S4	LSEI_2837	Amino acid ABC transporter substrate-binding protein, PAAT family / amino acid ABC transporter membrane protein, PAAT family
25,8224	25,8541	25,7469	25,9137	25,8188	25,5337	25,4983	25,6625	25,8793	25,7861		6	6	6	29,1	29,1	29,1	34,861	8,51E+08	24	25,7516	25,8312	25,6719	10	5	5	0,0739035	-0,159236	Q03A27	Q03A27	LSEI_1157	Threonylcarbamoyl-AMP synthase
24,4475	25,835	25,6896	25,74	25,8139	25,8396	25,7191	25,3234	26,0327	25,4729		4	4	4	52,8	52,8	52,8	11,61	3,85E+08	21	25,5914	25,5052	25,6775	10	5	5	0,574365	0,172363	Q03BD0	Q03BD0	LSEI_0651	UPF0145 protein LSEI_0651
26,0118	25,9097	25,9826	26,139	26,0045	25,3329	25,586	25,4436	26,1629	25,9241		6	6	6	20,8	20,8	20,8	34,714	6,67E+08	34	25,8497	26,0095	25,6899	10	5	5	0,0791121	-0,319596	Q034N4	Q034N4	LSEI_2618	Predicted oxidoreductase
25,6099	25,9802	25,8921	25,9446	25,8574	25,6359	25,4231	25,5589	26,0555	25,8081		10	10	10	52,4	52,4	52,4	25,874	1,27E+09	61	25,7766	25,8568	25,6963	10	5	5	0,24266	-0,160522	Q03AN2	Q03AN2	LSEI_0941	Phosphate-specific transport system accessory protein PhoU
25,9746	25,6804	25,3983	25,3083	25,4298	25,8	25,5201	25,6703	25,6689	25,8593		10	10	10	50	50	50	28,408	6,11E+08	46	25,631	25,5583	25,7037	10	5	5	0,31137	0,145445	Q03AW5	Q03AW5	LSEI_0821	Acid sugar phosphatase
25,8992	25,8327	25,8087	25,9679	26,0433	25,4933	25,6866	25,8263	25,7148	25,8449	+	15	15	15	30,3	30,3	30,3	52,251	1,95E+09	109	25,8118	25,9104	25,7132	10	5	5	0,0325643	-0,197164	Q036Y1	Q036Y1	LSEI_1983	Dipeptidase
25,2306	25,3968	25,4185	25,5744	25,3836	25,6001	25,6319	25,6407	26,0236	25,7319	+	10	10	10	45,8	45,8	45,8	26,993	6,62E+08	37	25,5632	25,4008	25,7256	10	5	5	0,00907726	0,324834	Q039J4	Q039J4	LSEI_1349	Transcriptional regulator, DeoR family
25,994	25,9993	25,7964	25,8619	26,0785	25,6247	25,7537	25,8318	25,8248	25,6241	+	11	11	11	72,5	72,5	72,5	16,649	1,43E+09	86	25,8389	25,946	25,7318	10	5	5	0,014259	-0,214207	Q036H6	Q036H6	LSEI_2147	Universal stress protein UspA related nucleotide-binding protein
25,7823	25,9421	25,9676	26,0803	26,1555	25,6107	25,5162	25,7158	25,8365	26,0114		7	7	7	25,3	25,3	25,3	29,992	9,20E+08	48	25,8618	25,9856	25,7381	10	5	5	0,0505458	-0,247462	Q037L3	Q037L3	LSEI_1850	Esterase/lipase
26,3917	25,8797	25,9742	25,827	25,8553	25,7617	26,0286	25,5712	25,835	25,5035		14	14	14	75,4	75,4	75,4	25,548	1,98E+09	72	25,8628	25,9856	25,74	10	5	5	0,118902	-0,245597	Q03AP2	Q03AP2	ftsE	Cell division ATP-binding protein FtsE
26,0862	25,9734	26,0045	26,0224	26,0884	25,6613	25,644	25,6554	26,0373	25,7411	+	11	11	11	44,1	44,1	44,1	34,918	1,29E+09	62	25,8914	26,035	25,7478	10	5	5	0,00611521	-0,287176	Q039S2	Q039S2	rsmH	Ribosomal RNA small subunit methyltransferase H
25,8801	26,0738	26,0779	26,0348	25,9709	25,6506	25,7483	25,8516	25,8439	25,6603	+	9	9	9	29,3	29,3	29,3	37,705	9,82E+08	53	25,8792	26,0075	25,7509	10	5	5	0,00197917	-0,256563	Q03AM6	Q03AM6	gpsA	Glycerol-3-phosphate dehydrogenase [NAD(P)+]
25,9267	26,0807	26,215	26,1841	26,2197	25,811	25,6168	25,6311	25,8751	25,8278	+	8	8	8	24,4	24,4	24,4	39,4	9,49E+08	49	25,9388	26,1252	25,7524	10	5	5	0,00130271	-0,372829	Q038Z1	Q038Z1	carA	Carbamoyl-phosphate synthase small chain
25,7402	25,8149	25,7265	25,9176	25,879	25,6608	25,6356	25,634	26,0614	25,7756		10	10	10	50,2	50,2	50,2	25,916	9,69E+08	47	25,7846	25,8157	25,7535	10	5	5	0,50668	-0,0621914	Q038J1	Q038J1	rnc	Ribonuclease 3
26,3272	26,195	26,0295	26,042	26,0323	25,7324	25,681	25,7999	25,8553	25,7286	+	24	24	24	71,2	71,2	71,2	48,51	2,83E+09	128	25,9423	26,1252	25,7594	10	5	5	0,000586887	-0,365768	Q03A61	Q03A61	LSEI_1120	Adenylosuccinate lyase
25,4148	26,0452	25,9937	25,986	25,7944	25,6922	25,861	25,677	26,147	25,5306		7	7	7	40,7	40,7	40,7	18,026	1,20E+09	47	25,8142	25,8468	25,7816	10	5	5	0,688093	-0,0652653	Q036B6	Q036B6	LSEI_2209	Uncharacterized protein
25,8633	25,9769	25,966	25,8237	25,8304	25,5909	25,7881	25,7607	25,871	25,9053		16	16	16	45,4	45,4	45,4	54,314	1,67E+09	72	25,8376	25,8921	25,7832	10	5	5	0,127691	-0,108878	Q03A40	Q03A40	LSEI_1142	Dipeptidase
25,8864	25,8186	25,8563	25,898	26,0023	25,6543	25,7768	25,8866	25,8155	25,8117		12	12	12	41,1	41,1	41,1	36,173	1,43E+09	83	25,8407	25,8923	25,789	10	5	5	0,0678085	-0,103333	Q038J3	Q038J3	ftsY	Signal recognition particle receptor FtsY
25,4821	25,9528	25,9281	26,0672	25,9868	25,6561	25,6525	25,7127	26,2393	25,7462		9	9	9	59,2	59,2	59,2	22,247	1,14E+09	52	25,8424	25,8834	25,8014	10	5	5	0,602686	-0,0820217	Q03AR7	Q03AR7	cutC	Copper homeostasis protein CutC
25,9165	25,9027	25,7591	25,9094	25,7215	25,6334	25,7276	25,8258	26,0534	25,7795		13	13	13	38,1	38,1	38,1	39,188	1,43E+09	58	25,8229	25,8418	25,8039	10	5	5	0,654987	-0,0378975	Q03A87	Q03A87	LSEI_1092	Phosphomevalonate kinase
24,3987	23,9862	23,6844	23,5323	23,9513	25,5688	26,1134	26,0964	25,1555	26,1043	+	3	3	3	9,9	9,9	9,9	37,677	2,30E+08	23	24,8591	23,9106	25,8077	10	5	5	5,30E-05	1,8971	Q03AQ0	Q03AQ0	recA	Protein RecA
25,8872	25,9767	26,0228	26,0895	25,9246	25,7541	25,8006	25,836	26,0772	25,5902		13	13	13	50,2	50,2	50,2	32,151	1,22E+09	51	25,8959	25,9802	25,8116	10	5	5	0,0866574	-0,1686	Q03AI2	Q03AI2	LSEI_0994	Predicted hydrolase of the HAD superfamily
26,0105	26,0541	25,9732	25,837	25,9007	25,8515	25,7707	25,8668	25,6144	26,0031		21	21	21	35,8	35,8	35,8	82,007	2,09E+09	115	25,8882	25,9551	25,8213	10	5	5	0,110928	-0,133792	Q03BD8	Q03BD8	LSEI_0643	Uncharacterized protein
25,8773	26,2344	26,2832	26,0105	26,1478	25,9773	25,4558	26,0559	25,9884	25,661		10	10	10	31	31	31	47,514	1,29E+09	31	25,9691	26,1106	25,8277	10	5	5	0,0733477	-0,282968	Q038U5	Q038U5	sigA	RNA polymerase sigma factor SigA
26,5929	26,242	26,0709	25,9423	26,0453	25,9815	25,7857	25,8604	25,713	25,8594	+	20	20	20	47,9	47,9	47,9	52,951	1,70E+09	66	26,0093	26,1787	25,84	10	5	5	0,0246092	-0,338693	Q038J6	Q038J6	ffh	Signal recognition particle protein
26,1674	26,3306	26,1344	26,2058	25,954	26,0055	26,0678	25,9957	25,8026	25,3371		12	12	12	16,2	16,2	16,2	94,791	6,71E+08	33	26,0001	26,1585	25,8417	10	5	5	0,0633298	-0,316722	Q035Z2	Q035Z2	mutS	DNA mismatch repair protein MutS
25,8192	25,9272	25,6562	25,444	25,5825	25,8511	25,9542	26,0822	26,1349	25,2468		8	8	8	57,1	57,1	57,1	18,897	6,51E+08	31	25,7698	25,6858	25,8538	10	5	5	0,380648	0,167998	Q038P1	Q038P1	apt	Adenine phosphoribosyltransferase
26,5182	25,879	25,988	26,6518	26,0008	26,1969	25,7623	26,1853	25,8065	25,3198		7	7	7	32,5	32,5	32,5	34,724	1,00E+09	44	25,9309	26,0075	25,8542	10	5	5	0,496644	-0,153362	Q036K3	Q036K3	fabH	3-oxoacyl-[acyl-carrier-protein] synthase
26,0182	25,8829	25,8103	25,972	25,7317	25,7592	25,7425	25,8555	25,9767	25,9887		12	12	12	28,7	28,7	28,7	48,611	1,41E+09	70	25,8738	25,883	25,8645	10	5	5	0,807863	-0,0185074	Q039G4	Q039G4	der	GTPase Der
25,4361	25,5943	25,619	25,7653	25,6035	25,5737	25,7543	25,7883	26,2024	26,0438		6	6	6	26,5	26,5	26,5	31,378	7,86E+08	58	25,7381	25,6036	25,8725	10	5	5	0,0604636	0,268854	Q034W5	Q034W5	hslO	3 kDa chaperonin
25,6484	25,8546	25,8319	25,9681	25,9306	25,6213	25,9417	25,8581	26,1042	25,8751		7	7	7	29,9	29,9	29,9	18,743	1,42E+09	53	25,8634	25,8467	25,8801	10	5	5	0,735956	0,033392	Q039E0	Q039E0	hslV	ATP-dependent protease subunit HslV
26,1663	25,8554	25,8374	25,7956	25,9315	25,6703	25,9112	25,868	26,0522	25,9012		14	14	14	41,2	41,2	41,2	47,565	1,80E+09	89	25,8989	25,9172	25,8806	10	5	5	0,694861	-0,0366543	Q038S0	Q038S0	hisS	Histidine--tRNA ligase
25,6672	26,4213	26,3126	26,4984	26,024	25,9579	25,8259	25,6863	26,1642	25,8425		3	3	3	31,1	31,1	31,1	11,208	8,78E+08	25	26,04	26,1847	25,8953	10	5	5	0,131063	-0,28936	Q038B0	Q038B0	LSEI_1689	RNA-binding protein, KH domain
26,1368	26,1128	26,0544	25,92	26,0046	25,8682	25,7837	25,9489	25,7783	26,1058		29	29	29	36,3	36,3	36,3	96,771	2,85E+09	159	25,9713	26,0457	25,897	10	5	5	0,0734611	-0,148759	Q03D50	Q03D50	gyrA	DNA gyrase subunit A
26,5123	26,3135	26,3147	26,2774	25,9018	25,9168	25,6751	25,9086	26,0631	25,9842	+	5	5	5	9,1	9,1	9,1	70,429	6,78E+08	34	26,0868	26,264	25,9096	10	5	5	0,0175041	-0,354404	Q03BE9	Q03BE9	LSEI_0631	PTS system IIB component, Glc family / PTS system IIC component, Glc family / PTS system IIA component, Glc family
25,1028	26,0091	26,0817	26,3307	25,9947	25,9863	25,7238	25,7743	26,1753	25,9111		2	2	2	25,7	25,7	25,7	8,2653	5,21E+08	14	25,909	25,9038	25,9142	10	5	5	0,964207	0,0103733	Q039P0	Q039P0	rpoY	DNA-directed RNA polymerase subunit epsilon
25,7786	26,3484	26,0056	25,8536	26,1502	25,6479	25,8786	25,8081	26,2121	26,0393		7	7	7	53,6	53,6	53,6	21,384	7,49E+08	34	25,9722	26,0273	25,9172	10	5	5	0,457806	-0,110109	Q03A64	Q03A64	xpt	Xanthine phosphoribosyltransferase
25,318	25,8069	25,8676	25,909	25,9569	25,9943	25,657	25,8279	26,2113	25,9488		8	8	8	45,4	45,4	45,4	21,349	8,78E+08	38	25,8498	25,7717	25,9279	10	5	5	0,32207	0,156174	Q03AV9	Q03AV9	LSEI_0827	Peptidyl-prolyl cis-trans isomerase
25,7959	25,917	25,7684	25,7463	25,8865	25,8992	25,9605	25,9467	25,9032	25,9818	+	11	11	11	32,5	32,5	32,5	50,327	1,51E+09	81	25,8805	25,8228	25,9383	10	5	5	0,0145848	0,115483	Q03AX3	Q03AX3	LSEI_0813	Peptidase V, Metallo peptidase, MEROPS family M20A
26,1995	26,4032	26,151	26,0151	26,0219	25,6705	25,9535	25,9512	26,0129	26,1036		17	17	17	44,5	44,5	44,5	56,57	2,32E+09	99	26,0482	26,1581	25,9383	10	5	5	0,0621315	-0,219806	Q03CI6	Q03CI6	murE	UDP-N-acetylmuramyl-tripeptide synthetase
25,395	25,7251	26,0337	25,994	26,1732	25,9857	25,8281	25,7953	25,9643	26,123		7	7	7	45,6	45,6	45,6	33,347	8,16E+08	39	25,9017	25,8642	25,9393	10	5	5	0,63031	0,0750702	Q03A46	Q03A46	LSEI_1135	Predicted dehydrogenase related protein
24,9995	25,0504	25,1275	25,1172	25,1051	25,8896	25,9635	25,9164	25,7105	26,2198	+	17	17	17	22,7	22,7	22,7	98,861	1,12E+09	65	25,51	25,0799	25,94	10	5	5	8,13E-06	0,860022	Q03C06	Q03C06	LSEI_0410	Predicted membrane protein
26,0563	26,2503	26,2689	26,2192	25,9687	25,8696	25,8665	25,9992	26,1283	25,8426	+	16	16	16	37,7	37,7	37,7	47,807	2,57E+09	119	26,047	26,1527	25,9413	10	5	5	0,0302458	-0,21142	Q036T6	Q036T6	tyrS	Tyrosine--tRNA ligase
25,4363	25,6973	25,5293	25,5433	25,5513	25,8032	26,0664	25,9427	26,117	25,7965	+	5	5	5	30	30	30	26,179	5,48E+08	30	25,7483	25,5515	25,9452	10	5	5	0,000989929	0,393685	Q036I3	Q036I3	LSEI_2139	Sortase (Surface protein transpeptidase)
25,8434	25,9494	26,1105	26,2758	26,0375	25,8465	25,9408	25,9223	26,1506	25,9525		18	18	18	56,9	56,9	56,9	32,616	2,70E+09	107	26,003	26,0434	25,9625	10	5	5	0,390411	-0,0808056	Q03CJ6	Q03CJ6	LSEI_0213	Chromosome segregation DNA-binding protein
26,6249	26,3596	26,0846	26,2023	26,0801	25,8877	25,812	26,023	26,2274	25,8728	+	17	17	16	47,9	47,9	44,8	36,347	2,75E+09	123	26,1174	26,2703	25,9645	10	5	5	0,0418187	-0,305743	Q038I7	Q038I7	LSEI_1611	ABC-type oligopeptide transport system, ATPase component
26,1666	25,9197	25,7393	25,7274	26,0651	26,0761	25,8748	25,8898	26,0892	25,9095		10	10	10	56,6	56,6	56,6	23,297	1,25E+09	71	25,9457	25,9236	25,9679	10	5	5	0,666918	0,0442379	Q037N0	Q037N0	LSEI_1827	Putative NADH-flavin reductase
25,9857	26,0723	26,0452	26,234	26,1606	25,8142	25,9109	25,9934	25,9883	26,1373		13	13	13	40,2	40,2	40,2	39,146	1,95E+09	71	26,0342	26,0996	25,9688	10	5	5	0,0945399	-0,130744	Q038C7	Q038C7	pheS	Phenylalanine--tRNA ligase alpha subunit
26,0793	26,2441	26,1047	25,985	25,8825	25,9656	25,8435	25,9249	26,0866	26,0306		11	11	11	64,4	64,4	64,4	26,554	1,32E+09	56	26,0147	26,0591	25,9702	10	5	5	0,262409	-0,0888966	Q037P5	Q037P5	LSEI_1807	Transcriptional regulator, GntR family
26,3396	26,2135	26,2221	26,2716	26,2822	25,8113	26,0399	25,8041	26,4286	25,7803	+	9	9	9	39,8	39,8	39,8	23,075	1,74E+09	68	26,1193	26,2658	25,9729	10	5	5	0,0476184	-0,292946	Q039B9	Q039B9	LSEI_1425	Nitroreductase
26,0895	25,5368	25,7989	26,0115	26,0034	25,9152	26,0033	26,0741	25,6358	26,2692		19	19	19	48,2	48,2	48,2	44,628	1,88E+09	85	25,9338	25,888	25,9795	10	5	5	0,543646	0,0914818	Q039Q5	Q039Q5	LSEI_1284	Tellurite resistance protein
25,7595	26,2049	26,1054	26,0905	25,9936	25,911	26,0791	26,0572	26,4169	25,4529		7	7	7	28,9	28,9	28,9	25,485	1,36E+09	88	26,0071	26,0308	25,9834	10	5	5	0,792006	-0,0473682	Q03CJ0	Q03CJ0	LSEI_0219	DNA-binding response regulator, OmpR family (Rec-wHTH domains)
23,9603	24,9531	25,0452	25,3846	24,9251	25,6249	25,9123	25,444	26,6374	26,3088	+	3	3	3	38,4	38,4	38,4	8,04	3,47E+08	18	25,4196	24,8537	25,9855	10	5	5	0,00806174	1,13183	Q03A12	Q03A12	LSEI_1172	Uncharacterized protein
25,9769	26,0158	26,236	26,3376	26,1987	25,9902	25,8186	26,0059	26,4011	25,7528		12	12	12	49,5	49,5	49,5	34,167	2,07E+09	62	26,0734	26,153	25,9937	10	5	5	0,261528	-0,159274	Q036X6	Q036X6	LSEI_1988	Proline iminopeptidase
25,9443	26,3033	26,2761	26,2507	26,1104	25,8243	25,9263	25,923	26,4688	25,8377		11	11	11	57,5	57,5	57,5	24,99	1,20E+09	65	26,0865	26,177	25,996	10	5	5	0,224608	-0,180935	Q036X2	Q036X2	LSEI_1992	Phosphoglycerate mutase family protein
25,3543	26,4985	26,3757	26,6917	26,1993	25,9732	25,8734	25,9789	26,3947	25,7965		4	4	4	25,4	25,4	25,4	15,087	9,45E+08	27	26,1136	26,2239	26,0034	10	5	5	0,410218	-0,220538	Q03CK0	Q03CK0	LSEI_0209	Uncharacterized protein
26,4737	26,5179	26,337	26,3547	26,3088	25,8806	25,9792	26,0147	26,2237	26,0029	+	9	9	9	32,8	32,8	32,8	36,503	1,96E+09	58	26,2093	26,3984	26,0202	10	5	5	0,00061406	-0,378208	Q03AR1	Q03AR1	guaC	GMP reductase
26,0011	26,1649	26,1599	26,3398	26,2334	25,8397	25,9896	25,9107	26,3481	26,0212		11	11	11	66,2	66,2	66,2	23,29	1,41E+09	64	26,1008	26,1798	26,0218	10	5	5	0,165315	-0,157971	Q035F8	Q035F8	LSEI_2428	Saccharopine dehydrogenase related protein
25,6671	26,0644	26,0712	26,0245	26,0308	25,9046	25,8969	26,0093	26,3323	26,032		7	7	7	31,1	31,1	31,1	27,916	1,41E+09	66	26,0033	25,9716	26,035	10	5	5	0,580442	0,0634491	Q036L3	Q036L3	LSEI_2109	Acetyl-CoA carboxytransferase
26,1375	26,257	26,2547	26,1273	26,3118	25,948	26,1082	26,1337	26,2694	25,7877		11	11	11	31,3	31,3	31,3	44,868	1,94E+09	77	26,1335	26,2177	26,0494	10	5	5	0,100305	-0,168254	Q034S9	Q034S9	murA	UDP-N-acetylglucosamine 1-carboxyvinyltransferase
25,2752	25,8211	25,7935	25,8666	25,9099	26,058	26,0105	25,9326	26,1122	26,1841	+	5	5	5	37,2	37,2	37,2	12,588	6,07E+08	26	25,8964	25,7333	26,0595	10	5	5	0,0300502	0,326187	Q039P9	Q039P9	LSEI_1290	Uncharacterized protein
26,4737	26,4164	26,132	26,0963	26,2219	25,9732	25,9879	26,048	26,2048	26,0936	+	20	20	20	33,6	33,6	33,6	62,323	2,57E+09	113	26,1648	26,2681	26,0615	10	5	5	0,0438762	-0,206554	Q039L1	Q039L1	rnj	Ribonuclease J
26,1642	26,0508	26,0996	26,0156	26,1317	25,9677	26,012	26,1306	26,2237	25,993		11	11	11	43,1	43,1	43,1	31,858	1,54E+09	69	26,0789	26,0924	26,0654	10	5	5	0,638899	-0,0269981	Q03B85	Q03B85	LSEI_0697	Uncharacterized protein
25,879	26,1447	26,0494	26,2051	25,9525	25,8976	26,1434	25,999	26,3664	25,9233		7	7	7	30,5	30,5	30,5	34,354	1,38E+09	44	26,056	26,0461	26,0659	10	5	5	0,855167	0,0198212	Q038H3	Q038H3	fmt	Methionyl-tRNA formyltransferase
25,3214	25,6887	25,6504	25,7432	25,6173	25,9047	26,0055	26,134	26,2188	26,0977	+	12	12	12	44,4	44,4	44,4	41,607	1,40E+09	62	25,8382	25,6042	26,0722	10	5	5	0,000909816	0,467952	Q03AC2	Q03AC2	LSEI_1056	Uncharacterized protein
26,9504	26,8956	26,7524	26,7234	26,5942	26,0532	26,0051	26,0466	26,05	26,3462	+	26	26	26	48,9	48,9	48,9	85,142	5,20E+09	166	26,4417	26,7832	26,1002	10	5	5	5,83E-05	-0,682959	Q039D4	Q039D4	LSEI_1410	Formate acetyltransferase
27,7659	27,5831	27,1855	26,5422	26,5164	26,0037	26,1462	26,0331	26,454	25,974	+	4	4	4	12,5	12,5	12,5	30,812	9,92E+08	30	26,6204	27,1186	26,1222	10	5	5	0,00647556	-0,996391	Q03BL2	Q03BL2	LSEI_0562	PTS system IID component, Man family
26,1528	26,2785	26,1023	25,8686	26,1344	26,1789	26,0921	26,0314	26,2721	26,0604		12	12	12	41,9	41,9	41,9	34,507	1,08E+09	51	26,1171	26,1073	26,127	10	5	5	0,8117	0,0196712	Q03B30	Q03B30	LSEI_0756	Hydrolase of the alpha/beta superfamily
26,2286	26,4442	26,3634	26,4056	26,4036	26,1687	26,147	26,1074	26,2938	25,9629	+	14	14	14	63,8	63,8	63,8	22,639	1,73E+09	68	26,2525	26,3691	26,1359	10	5	5	0,00718679	-0,23314	Q038B2	Q038B2	LSEI_1687	NAD metabolism hydrolase of HD superfamily
26,0662	26,1504	26,0287	26,1376	26,023	25,911	26,0154	26,181	26,4295	26,2537		10	10	10	40	40	40	30,085	1,75E+09	88	26,1197	26,0812	26,1581	10	5	5	0,439904	0,0769066	Q039F4	Q039F4	LSEI_1390	Uncharacterized protein
26,7455	26,7917	26,6303	26,5914	26,7025	25,9821	26,0527	26,1731	26,3551	26,2495	+	16	16	16	42,9	42,9	42,9	48,378	2,15E+09	78	26,4274	26,6923	26,1625	10	5	5	0,000118354	-0,529798	Q039R5	Q039R5	ftsA	Cell division protein FtsA
25,9536	26,2078	26,0585	26,159	26,0492	25,9402	26,3027	26,3201	26,4413	25,8256		17	17	17	30,1	30,1	30,1	73,625	1,18E+09	65	26,1258	26,0856	26,166	10	5	5	0,545934	0,0803627	Q03AC3	Q03AC3	ligA	DNA ligase
25,4022	26,3666	26,4041	26,2926	26,4009	26,1131	26,1106	26,1684	26,3288	26,1523		7	7	7	56,4	56,4	56,4	16,515	1,62E+09	58	26,174	26,1733	26,1747	10	5	5	0,994532	0,00139923	Q03B38	Q03B38	LSEI_0748	Nucleoside diphosphate kinase
27,1175	27,0163	26,706	26,6395	26,5417	26,0775	26,1668	26,1506	26,1696	26,3345	+	20	20	20	30,1	30,1	30,1	70,793	2,84E+09	98	26,492	26,8042	26,1798	10	5	5	0,000786927	-0,624387	Q035T1	Q035T1	LSEI_2297	NADH dehydrogenase, FAD-containing subunit
26,3157	26,4424	26,3966	26,5302	26,3206	26,2912	26,1143	26,1312	26,1525	26,2198	+	6	6	6	28,6	28,6	28,6	30,512	1,39E+09	39	26,2915	26,4011	26,1818	10	5	5	0,00284752	-0,219297	Q03CJ3	Q03CJ3	LSEI_0216	Uncharacterized membrane-bound protein conserved in bacteria
26,2263	26,0659	26,1819	26,2686	26,296	26,1684	26,0407	26,2271	26,318	26,2861		8	8	8	26	26	26	37,7	1,13E+09	45	26,2079	26,2077	26,2081	10	5	5	0,996137	0,00031738	Q034M8	Q034M8	LSEI_2624	Tryptophan--tRNA ligase
25,6326	25,7939	25,7556	25,6714	25,5223	25,9073	26,3453	26,2058	26,258	26,3719	+	20	20	20	36,9	36,9	36,9	78,034	1,86E+09	89	25,9464	25,6752	26,2177	10	5	5	0,000477486	0,542502	Q03AU7	Q03AU7	LSEI_0868	Phosphoglycerol transferase related protein, alkaline phosphatase superfamily
25,9848	26,4289	26,3994	26,4089	26,5204	26,0844	26,0476	26,2756	26,4623	26,2237		11	11	11	52,5	52,5	52,5	37,898	2,12E+09	63	26,2836	26,3485	26,2187	10	5	5	0,308244	-0,129774	Q03CC0	Q03CC0	LSEI_0292	Mannose-6-phosphate isomerase
25,4708	25,5246	25,5374	25,5826	25,5271	25,9672	26,21	26,3514	26,2599	26,4162	+	8	6	6	15,6	14	14	52,876	6,19E+08	33	25,8847	25,5285	26,2409	10	5	5	1,86E-05	0,712428	Q033R2	Q033R2	LSEI_2850	Amino acid ABC transporter membrane protein, PAAT family / amino acid ABC transporter substrate-binding protein, PAAT family
26,8427	26,7636	26,7521	26,6734	26,7135	26,1954	26,2582	26,4255	26,0158	26,4057	+	26	26	26	32,7	32,7	32,7	103,12	5,35E+09	181	26,5046	26,7491	26,2601	10	5	5	0,000288727	-0,488937	Q038M5	Q038M5	infB	Translation initiation factor IF-2
26,2151	26,4649	26,3887	26,5308	26,407	26,2547	26,2478	26,1104	26,2122	26,4824		12	12	12	29,1	29,1	29,1	64,272	8,04E+08	42	26,3314	26,4013	26,2615	10	5	5	0,121155	-0,139767	Q039A5	Q039A5	rqcH	Rqc2 homolog RqcH
25,5264	25,5282	25,4292	25,75	25,403	25,9516	26,4976	26,2287	26,0896	26,5735	+	10	10	10	36	36	36	36,311	8,63E+08	46	25,8978	25,5274	26,2682	10	5	5	0,000530342	0,740851	Q035Y6	Q035Y6	LSEI_2241	Transcriptional regulator
24,8704	26,1719	26,1584	26,3242	26,041	26,0532	26,2597	26,1113	26,5923	26,3609		7	7	7	39,3	39,3	39,3	13,309	7,56E+08	43	26,0943	25,9132	26,2755	10	5	5	0,233938	0,362317	Q038M6	Q038M6	rbfA	Ribosome-binding factor A
26,3149	26,506	26,5449	26,5611	26,381	26,0675	26,2477	26,2538	26,6127	26,2827		11	11	10	46,3	46,3	43,4	41,962	2,10E+09	66	26,3772	26,4616	26,2929	10	5	5	0,133036	-0,168715	Q036G6	Q036G6	LSEI_2157	Aminotransferase
26,1564	26,6024	26,5407	26,5868	26,4368	26,0549	26,2094	26,254	26,5772	26,4054		11	11	11	37,6	37,6	37,6	42,533	2,14E+09	62	26,3824	26,4646	26,3002	10	5	5	0,211877	-0,164451	Q038W0	Q038W0	LSEI_1486	Aminotransferase
26,2957	26,3071	26,3241	26,3996	26,3756	26,404	26,2364	26,3541	26,4029	26,1214		7	7	7	30,6	30,6	30,6	32,138	9,97E+08	40	26,3221	26,3404	26,3038	10	5	5	0,548116	-0,0366497	Q039J5	Q039J5	LSEI_1348	Tagatose-6-phosphate kinase
26,7339	26,8151	26,9393	26,9286	26,8927	26,0931	26,3186	26,2325	26,4761	26,432	+	14	14	14	55,5	55,5	55,5	35,943	2,58E+09	88	26,5862	26,8619	26,3105	10	5	5	0,000117396	-0,551461	Q03C04	Q03C04	LSEI_0412	Penicillin amidase, Cysteine peptidase, MEROPS family C59
24,1879	25,8177	26,1051	26,4014	26,1964	26,3153	26,4347	25,8711	26,7268	26,2055		5	5	5	34,6	34,6	34,6	9,73	9,44E+08	41	26,0262	25,7417	26,3107	10	5	5	0,21598	0,568952	Q038K3	Q038K3	LSEI_1595	UPF0291 protein LSEI_1595
26,2618	26,2585	26,4245	26,4446	26,3415	26,182	26,2071	26,3662	26,4763	26,3459		12	12	12	55,5	55,5	55,5	37,581	2,36E+09	97	26,3308	26,3462	26,3155	10	5	5	0,658831	-0,0306843	Q03AR4	Q03AR4	LSEI_0908	6-phosphogluconolactonase
26,6074	26,6455	26,5448	26,5265	26,3259	26,0837	26,2563	26,2599	26,6882	26,3101		17	17	17	61	61	61	32,653	3,44E+09	150	26,4248	26,53	26,3196	10	5	5	0,102441	-0,210392	Q039D8	Q039D8	LSEI_1406	Galactose mutarotase related enzyme
26,4221	26,7819	26,6584	26,661	26,6007	26,1909	26,3545	26,3648	26,3525	26,3434	+	32	32	32	52,2	52,2	52,2	73,065	4,17E+09	159	26,473	26,6248	26,3212	10	5	5	0,00195121	-0,303605	Q036G1	Q036G1	LSEI_2162	Asparagine synthase (Glutamine-hydrolyzing)
26,3547	26,5116	26,4306	26,4069	26,4283	26,3111	26,2303	26,3504	26,1652	26,5605		24	24	24	39,4	39,4	39,4	88,98	3,46E+09	155	26,3749	26,4264	26,3235	10	5	5	0,190664	-0,102896	Q03AJ8	Q03AJ8	rnr	Ribonuclease R
26,1765	26,4093	26,2625	26,2052	26,3634	26,0512	26,5909	26,3553	26,3912	26,2903		11	11	11	35,4	35,4	35,4	44,206	6,50E+08	35	26,3096	26,2834	26,3358	10	5	5	0,607186	0,0524025	Q039G0	Q039G0	cca	CCA-adding enzyme
26,904	26,7859	26,6851	26,8579	26,8391	26,2972	26,2786	26,2828	26,5112	26,3879	+	13	13	13	37,5	37,5	37,5	41,448	2,68E+09	85	26,583	26,8144	26,3515	10	5	5	4,59E-05	-0,462866	Q038N5	Q038N5	dnaJ	Chaperone protein DnaJ
26,8819	26,7566	26,658	26,5619	26,5422	26,3328	26,2984	26,3653	26,3581	26,4991	+	33	33	33	49,8	49,8	49,8	89,366	4,88E+09	174	26,5254	26,6801	26,3707	10	5	5	0,00260797	-0,309365	Q03AP4	Q03AP4	secA	Protein translocase subunit SecA
26,6845	26,3462	26,4108	26,4637	26,6431	26,3027	26,292	26,3088	26,401	26,579		13	13	13	49,2	49,2	49,2	34,038	2,28E+09	63	26,4432	26,5096	26,3767	10	5	5	0,158016	-0,132944	Q033T4	Q033T4	LSEI_2825	Aryl-alcohol dehydrogenase related enzyme
26,5484	26,6398	26,621	26,4666	26,5534	26,2495	26,4924	26,4245	26,1719	26,5754		19	19	19	31,3	31,3	31,3	87,744	2,76E+09	89	26,4743	26,5659	26,3828	10	5	5	0,0541727	-0,183094	Q038C8	Q038C8	pheT	Phenylalanine--tRNA ligase beta subunit
26,2063	26,2577	26,5973	26,7166	26,6502	26,2925	26,1918	26,1445	26,7394	26,597		6	6	6	20,2	20,2	20,2	34,138	1,12E+09	33	26,4393	26,4856	26,3931	10	5	5	0,573274	-0,0925514	Q037W8	Q037W8	pyrD	Dihydroorotate dehydrogenase
26,1216	26,3133	26,2325	26,2773	26,2386	26,5192	26,2736	26,3428	26,3074	26,524	+	5	5	5	44,8	44,8	44,8	23,595	7,08E+08	29	26,315	26,2367	26,3934	10	5	5	0,036397	0,15673	Q03CM2	Q03CM2	LSEI_0187	Uncharacterized protein
26,5906	26,5324	26,6324	26,7279	26,7897	26,4127	26,239	26,2994	26,495	26,5244	+	24	24	24	69,3	69,3	69,3	42,584	5,27E+09	249	26,5244	26,6546	26,3941	10	5	5	0,00679594	-0,260518	Q033M9	Q033M9	mtlD	Mannitol-1-phosphate 5-dehydrogenase
27,0298	26,507	26,2646	26,1957	26,5651	26,3834	26,4466	26,3708	26,4261	26,4072		18	18	18	54,4	54,4	54,4	49,055	2,14E+09	72	26,4596	26,5124	26,4068	10	5	5	0,494786	-0,105616	Q036L1	Q036L1	LSEI_2111	Acetyl-CoA carboxylase carboxyltransferase subunit alpha
26,8099	26,9072	26,6902	26,5601	26,5723	26,4125	26,4078	26,519	26,3287	26,3737	+	26	26	26	48,3	48,3	48,3	64,43	4,31E+09	155	26,5582	26,708	26,4084	10	5	5	0,00378474	-0,299596	Q038L9	Q038L9	proS	Proline--tRNA ligase
26,5975	26,6312	26,6255	26,6576	26,5315	26,5367	26,2925	26,3885	26,3398	26,502	+	10	10	10	38,2	38,2	38,2	38,981	1,44E+09	38	26,5103	26,6086	26,4119	10	5	5	0,00506263	-0,19674	Q03B86	Q03B86	LSEI_0696	Uncharacterized protein
27,0358	27,2698	27,1496	27,1375	27,083	26,3652	26,3294	26,3637	26,6055	26,4131	+	15	15	15	35,8	35,8	35,8	49,077	2,75E+09	94	26,7753	27,1351	26,4154	10	5	5	3,16E-06	-0,719748	Q039N3	Q039N3	LSEI_1308	Dihydrolipoyl dehydrogenase
26,6371	26,7154	26,6395	26,5301	26,5965	26,3579	26,3438	26,4615	26,3725	26,626	+	18	18	18	48,4	48,4	48,4	50,489	2,64E+09	104	26,528	26,6237	26,4323	10	5	5	0,0135314	-0,191389	Q039R8	Q039R8	murD	UDP-N-acetylmuramoylalanine--D-glutamate ligase
26,8769	26,5385	26,4877	26,5089	26,6308	26,4237	26,4257	26,4713	26,3765	26,4872	+	17	17	17	47,8	47,8	47,8	56,143	2,43E+09	93	26,5227	26,6085	26,4369	10	5	5	0,0489911	-0,171682	Q03AZ2	Q03AZ2	dltA	D-alanine--D-alanyl carrier protein ligase
26,3125	26,7064	26,6546	26,599	26,5065	26,3188	26,3511	26,4743	26,4362	26,6436		32	32	32	38,1	38,1	38,1	105,01	5,26E+09	223	26,5003	26,5558	26,4448	10	5	5	0,251207	-0,111	Q039Q9	Q039Q9	ileS	Isoleucine--tRNA ligase
26,8746	26,4145	26,4352	26,5585	26,5654	26,2488	26,4358	26,4346	26,6382	26,4796		14	14	14	46,3	46,3	46,3	34,211	2,42E+09	110	26,5085	26,5696	26,4474	10	5	5	0,269615	-0,122243	Q03AM5	Q03AM5	LSEI_0948	Thioredoxin reductase
26,5036	26,4294	26,4529	26,4305	26,501	26,391	26,4355	26,4599	26,3682	26,5886		23	23	23	58,8	58,8	58,8	53,365	4,79E+09	200	26,4561	26,4635	26,4486	10	5	5	0,731766	-0,0148666	Q03AB9	Q03AB9	gatB	Aspartyl/glutamyl-tRNA(Asn/Gln) amidotransferase subunit B

26,8591	26,9537	26,824	26,7519	26,7797	26,3668	26,3662	26,3806	26,6176	26,5191	+	27	27	27	56	56	56	58,958	4,90E+09	182	26,6419	26,8337	26,4501	10	5	5	0,000257584	-0,383591	Q038Y4	Q038Y4	fhs	Formate--tetrahydrofolate ligase
26,7771	26,7466	26,8855	26,9536	26,9399	26,3276	26,383	26,1898	26,8014	26,5571	+	15	15	15	61	61	61	25,824	2,73E+09	77	26,6562	26,8605	26,4518	10	5	5	0,00696863	-0,408748	Q033M5	Q033M5	nagB	Glucosamine-6-phosphate deaminase
25,7332	25,2744	25,3839	25,3573	24,9728	26,1078	26,548	26,4007	26,5195	26,715	+	11	11	11	19	19	19	74,651	3,97E+08	32	25,9013	25,3443	26,4582	10	5	5	0,000107531	1,11385	Q03CU3	Q03CU3	LSEI_0115	Cyclic-di-AMP phosphodiesterase
26,7768	26,8157	26,7183	26,7045	26,6885	26,3553	26,4554	26,4897	26,4598	26,6033	+	32	32	32	54,6	54,6	54,6	76,108	6,64E+09	239	26,6067	26,7407	26,4727	10	5	5	0,000415836	-0,268064	Q038U3	Q038U3	glyS	Glycine--tRNA ligase beta subunit
26,6192	26,8309	27,0379	27,1627	26,9963	26,5095	26,2945	26,4816	26,7138	26,4071	+	7	7	7	58,4	58,4	58,4	19,982	2,18E+09	42	26,7053	26,9294	26,4813	10	5	5	0,00492528	-0,448075	Q03BK0;Q03	Q03BK0;Q035L3	LSEI_0577	Uncharacterized protein
26,1938	26,4919	26,5697	26,5767	26,5952	26,2776	26,3929	26,4539	26,8186	26,4795		13	13	13	53,9	53,9	53,9	33,678	3,50E+09	127	26,485	26,4855	26,4845	10	5	5	0,993545	-0,00098114	Q033N2	Q033N2	LSEI_2882	Phosphoribosyl pyrophosphate synthase
26,1135	26,6857	26,5578	26,6648	26,554	26,3929	26,4107	26,3954	26,7112	26,5424		5	5	5	30,3	30,3	30,3	25,091	1,06E+09	40	26,5028	26,5151	26,4905	10	5	5	0,843785	-0,0246155	Q03AI1	Q03AI1	ung	Uracil-DNA glycosylase
26,1941	26,5928	26,4633	26,6251	26,4878	26,285	26,4931	26,4054	26,8033	26,5088		10	10	10	40,6	40,6	40,6	24,171	2,32E+09	78	26,4859	26,4726	26,4991	10	5	5	0,822906	0,0265125	Q035Y3	Q035Y3	rex	Redox-sensing transcriptional repressor Rex
26,3009	26,6002	26,476	26,3928	26,3931	26,295	26,338	26,4191	27,1064	26,3841		7	7	7	54,3	54,3	54,3	20,585	2,13E+09	62	26,4706	26,4326	26,5085	10	5	5	0,645897	0,0759262	Q039N7	Q039N7	def	Peptide deformylase
25,786	26,8129	26,8107	26,866	26,9557	26,8232	26,5607	26,6013	26,3365	26,2223		3	3	3	20,9	20,9	20,9	16,585	6,59E+08	22	26,5775	26,6462	26,5088	10	5	5	0,584034	-0,137429	Q038F7	Q038F7	LSEI_1641	Uncharacterized protein
26,4672	26,4127	26,3917	26,4791	26,4805	26,3327	26,4792	26,4853	26,7119	26,5678		10	10	10	47,9	47,9	47,9	22,451	1,26E+09	57	26,4808	26,4463	26,5154	10	5	5	0,316585	0,069117	Q037Y6	Q037Y6	LSEI_1713	EMAP domain
26,901	26,7922	26,6849	26,6148	26,682	26,4844	26,3957	26,68	26,5672	26,4655	+	27	27	27	67,7	67,7	67,7	56,329	4,73E+09	151	26,6268	26,735	26,5186	10	5	5	0,0148895	-0,216427	Q034T7	Q034T7	cshA	DEAD-box ATP-dependent RNA helicase CshA
26,6494	26,7949	26,7075	26,6983	26,7331	26,5002	26,4291	26,6657	26,3145	26,6887	+	28	28	28	41,7	41,7	41,7	91,526	4,44E+09	153	26,6182	26,7166	26,5197	10	5	5	0,0300249	-0,196978	Q03AT8	Q03AT8	leuS	Leucine--tRNA ligase
26,6517	26,8485	26,8199	26,7251	26,6235	26,3545	26,3792	26,6794	26,7572	26,476		6	6	6	24,2	24,2	24,2	34,246	1,06E+09	32	26,6315	26,7338	26,5293	10	5	5	0,0573221	-0,204501	Q038U2	Q038U2	glyQ	Glycine--tRNA ligase alpha subunit
26,8364	26,8856	26,7015	26,7563	26,7056	26,6217	26,4867	26,5698	26,6387	26,3802	+	9	9	9	36,2	36,2	36,2	37,962	1,94E+09	68	26,6582	26,7771	26,5394	10	5	5	0,00420834	-0,237688	Q038I6	Q038I6	LSEI_1612	ABC-type dipeptide/oligopeptide/nickel transport system, ATPase component
26,8697	26,7502	26,6691	26,7349	26,7496	26,4194	26,5265	26,5639	26,5972	26,5931	+	13	13	13	54,1	54,1	54,1	38,974	2,18E+09	95	26,6474	26,7547	26,54	10	5	5	0,00161445	-0,214712	Q038F5	Q038F5	LSEI_1643	Aminopeptidase P
27,1421	27,0444	26,9014	27,0793	26,9969	26,3882	26,4479	26,4059	27,0307	26,4326	+	13	13	13	83,4	83,4	83,4	17,531	1,75E+09	61	26,7869	27,0328	26,5411	10	5	5	0,00522141	-0,491755	Q039F9	Q039F9	LSEI_1385	Uncharacterized protein
26,3659	26,7008	26,6036	26,5309	26,5327	26,3857	26,5928	26,5783	26,4538	26,7186		38	38	38	52,3	52,3	52,3	100,67	5,81E+09	258	26,5463	26,5468	26,5458	10	5	5	0,990871	-0,00094185	Q039T7	Q039T7	valS	Valine--tRNA ligase
26,8924	26,7391	26,6378	26,5873	26,5416	26,4855	26,5983	26,5514	26,5946	26,6313		13	13	13	45,2	45,2	45,2	36,173	2,17E+09	87	26,6259	26,6796	26,5722	10	5	5	0,149431	-0,107409	Q038I4	Q038I4	plsX	Phosphate acyltransferase
26,821	26,6641	26,5633	26,5833	26,6252	26,5435	26,4781	26,5549	26,7725	26,513		21	21	21	73,4	73,4	73,4	36,296	3,74E+09	123	26,6119	26,6514	26,5724	10	5	5	0,286335	-0,0789978	Q03BB7	Q03BB7	LSEI_0665	UDP-glucose 4-epimerase
26,8636	26,9415	26,9785	26,7539	26,7881	26,5354	26,5503	26,4425	26,7352	26,6284	+	13	13	13	35,4	35,4	35,4	52,095	2,24E+09	78	26,7217	26,8651	26,5784	10	5	5	0,00230449	-0,286739	Q033Z2	Q033Z2	LSEI_2766	Nicotinate phosphoribosyltransferase
26,5858	26,7326	26,5926	26,5792	26,4857	26,5389	26,5307	26,6298	26,5871	26,6543		29	29	29	57,5	57,5	57,5	62,304	4,52E+09	169	26,5917	26,5952	26,5882	10	5	5	0,883351	-0,00702782	Q037X1	Q037X1	argS	Arginine--tRNA ligase
26,0996	26,0019	25,9876	25,9852	25,9758	26,5489	26,6999	26,6225	26,4113	26,7313	+	20	20	20	35,9	35,9	35,9	83,507	2,46E+09	83	26,3064	26,01	26,6028	10	5	5	1,15E-05	0,592764	Q038W4	Q038W4	LSEI_1481	DD-transpeptidase
26,6776	26,6716	26,7632	26,8311	26,9566	26,5448	26,552	26,6082	26,6899	26,6613	+	13	13	13	60,9	60,9	60,9	29,974	3,09E+09	103	26,6956	26,78	26,6112	10	5	5	0,0233975	-0,168773	Q03A91	Q03A91	map	Methionine aminopeptidase
27,1059	26,8024	26,7948	26,7727	26,8401	26,4835	26,6407	26,6217	26,5347	26,7833	+	15	15	15	38,4	38,4	38,4	59,662	3,98E+09	119	26,738	26,8632	26,6128	10	5	5	0,0142162	-0,250376	Q038I2	Q038I2	LSEI_1616	Dihydroxyacetone kinase related enzyme
25,8256	26,547	26,4881	26,4614	26,3989	26,278	26,552	26,6697	27,029	26,5437		7	7	7	66,9	66,9	66,9	14,728	9,40E+08	39	26,4794	26,3442	26,6145	10	5	5	0,170577	0,270269	Q03CL1	Q03CL1	LSEI_0198	Pyridoxine 5-phosphate oxidase V related favin-nucleotide-binding protein
26,8198	26,5238	26,773	26,7764	26,8196	26,4733	26,5219	26,5654	26,8046	26,7562		11	11	11	42,8	42,8	42,8	34,402	2,49E+09	92	26,6834	26,7425	26,6243	10	5	5	0,207267	-0,118235	Q03B05	Q03B05	LSEI_0781	Phosphoesterase, DHH family protein
26,4769	26,2493	26,4734	26,5264	26,4312	26,5195	26,5675	26,619	26,7655	26,681	+	10	10	10	50,7	50,7	50,7	24,519	1,05E+09	42	26,531	26,4315	26,6305	10	5	5	0,0149647	0,199057	Q039G6	Q039G6	cmk	Cytidylate kinase
26,5949	26,4621	26,4165	26,3903	26,3652	26,4682	26,6317	26,5297	26,8312	26,694	+	13	13	13	35,1	35,1	35,1	52,715	1,91E+09	79	26,5384	26,4458	26,631	10	5	5	0,0396156	0,185172	Q039J9	Q039J9	LSEI_1344	Amino acid ABC transporter membrane protein, PAAT family / amino acid ABC transporter substrate-binding protein, PAAT family
26,958	26,4215	26,515	26,4583	26,6486	26,5485	26,5905	26,6812	26,4718	26,8679		14	14	14	41,5	41,5	41,5	45,831	3,22E+09	97	26,6161	26,6003	26,632	10	5	5	0,796384	0,0316872	Q038M2	Q038M2	nusA	Transcription termination/antitermination protein NusA
26,9172	26,9321	26,9814	26,9943	27,1067	26,6051	26,6144	26,4973	26,8009	26,6716	+	11	11	11	44,3	44,3	44,3	40,812	2,30E+09	85	26,8121	26,9863	26,6379	10	5	5	0,000390704	-0,348485	Q039N6	Q039N6	pdhA	Pyruvate dehydrogenase E1 component subunit alpha
26,0688	26,1064	25,9577	26,0958	25,8582	26,4045	26,7585	26,5008	26,764	26,7664	+	6	6	6	22,2	22,2	22,2	32,6	9,56E+08	45	26,3281	26,0174	26,6388	10	5	5	0,000134413	0,621431	Q036R7	Q036R7	LSEI_2050	Transcriptional regulator
26,4031	26,5774	26,6546	26,6048	26,6182	26,5535	26,4666	26,5593	26,9725	26,6422		12	12	12	37,8	37,8	37,8	33,543	2,09E+09	94	26,6052	26,5716	26,6388	10	5	5	0,513526	0,0671818	Q038E0	Q038E0	LSEI_1659	Glucokinase
26,3134	26,5512	26,5109	26,7416	26,7019	26,5826	26,5369	26,5384	26,83	26,7176		13	13	13	60	60	60	23,472	2,68E+09	76	26,6025	26,5638	26,6411	10	5	5	0,441916	0,0773167	Q03CK3	Q03CK3	pcp	Pyrrolidone-carboxylate peptidase
27,2914	27,0056	26,9892	26,9454	27,118	26,7193	26,6457	26,6175	26,3502	26,8891	+	14	14	14	39,7	39,7	39,7	58,078	3,03E+09	87	26,8572	27,0699	26,6444	10	5	5	0,00414437	-0,425553	Q039N4	Q039N4	LSEI_1307	Dihydrolipoamide acetyltransferase component of pyruvate dehydrogenase complex
27,3798	27,2502	27,1342	27,0606	27,1556	27,1441	26,8212	26,844	27,0318	25,4885		14	14	14	54,2	54,2	54,2	30,436	3,16E+09	94	26,931	27,1961	26,6659	10	5	5	0,12077	-0,530151	Q03A08	Q03A08	LSEI_1177	Lipoprotein
26,6116	26,9928	26,7862	26,7012	26,6101	26,4996	26,758	26,685	27,2226	26,2773		5	5	5	35,4	35,4	35,4	17,356	1,43E+09	45	26,7144	26,7404	26,6885	10	5	5	0,771383	-0,0518822	Q034V6	Q034V6	LSEI_2543	RNA binding protein (S1 domain)
27,0733	26,9587	26,7891	26,7174	26,8853	26,6125	26,6894	26,7828	26,4786	26,8979		33	33	33	55,2	55,2	55,2	67,595	5,14E+09	249	26,7885	26,8847	26,6922	10	5	5	0,0774378	-0,192509	Q038S1	Q038S1	aspS	Aspartate--tRNA ligase
26,1601	26,9041	26,9748	27,1224	26,9053	26,6031	26,5733	26,5402	27,0122	26,7354		10	10	10	60,4	60,4	60,4	24,611	2,30E+09	93	26,7531	26,8133	26,6929	10	5	5	0,541692	-0,120466	Q03B32	Q03B32	LSEI_0754	Predicted phosphatase
26,3006	27,0833	27,0745	27,2587	26,9912	26,6763	26,7451	26,6818	26,7956	26,566		4	4	4	29,3	29,3	29,3	19,361	1,65E+09	44	26,8173	26,9417	26,693	10	5	5	0,182744	-0,248716	Q03A21	Q03A21	atpH	ATP synthase subunit delta
26,0151	26,0828	26,3634	26,4008	26,379	26,7935	26,8044	26,7296	26,0618	27,0905	+	19	19	19	22	22	22	133,94	3,23E+09	118	26,4721	26,2482	26,696	10	5	5	0,0454371	0,447783	Q037G3	Q037G3	LSEI_1901	LysM domain-containing protein
26,0663	27,0309	26,8927	26,9813	26,8324	26,5978	26,7596	26,5019	26,9713	26,6668		6	6	6	37	37	37	16,066	1,64E+09	48	26,7301	26,7607	26,6995	10	5	5	0,76056	-0,0612484	Q038E1	Q038E1	LSEI_1658	Rhodanese-related sulfurtransferase
26,5577	26,142	26,4528	26,8461	27,0363	26,895	26,535	26,5769	26,538	26,9555		14	14	14	53,3	53,3	53,3	28,528	2,95E+09	103	26,6535	26,607	26,7001	10	5	5	0,621134	0,0930832	Q039R0	Q039R0	LSEI_1279	Cell division initiation protein
27,4815	27,1136	27,1247	27,3228	27,2614	26,7503	26,509	26,8269	26,8542	26,6237	+	14	14	14	87,4	87,4	87,4	20,472	3,28E+09	122	26,9868	27,2608	26,7128	10	5	5	0,000391248	-0,547976	Q038Y7	Q038Y7	pyrR	Bifunctional protein PyrR
26,5023	26,9792	26,7265	26,7516	26,5723	26,8317	26,8097	26,8051	26,9275	26,195		4	4	4	44,1	44,1	44,1	15,673	1,78E+09	53	26,7101	26,7064	26,7138	10	5	5	0,963082	0,00741997	Q036K1	Q036K1	LSEI_2121	3-hydroxymyristoyl/3-hydroxydecanoyl-(Acyl carrier protein) dehydratase
25,4179	25,8188	25,9722	25,9117	25,7713	26,7728	26,6039	26,7854	26,5902	26,8216	+	5	5	5	26,6	26,6	26,6	34,848	8,69E+08	38	26,2466	25,7784	26,7148	10	5	5	2,49E-05	0,936431	Q034N8	Q034N8	LSEI_2614	Predicted secreted protein
24,8023	25,477	26,2802	26,5914	26,8865	26,7793	26,5549	26,636	26,8046	26,8326		3	3	3	47,5	47,5	47,5	9,0378	8,79E+08	29	26,3645	26,0075	26,7215	10	5	5	0,101472	0,71404	Q038I5	Q038I5	acpP	Acyl carrier protein
26,9224	27,1847	26,8463	26,8697	26,6461	26,6433	26,6384	26,6479	27,005	26,6996		11	11	11	51,6	51,6	51,6	24,889	1,90E+09	83	26,8103	26,8938	26,7268	10	5	5	0,172658	-0,167005	Q034U8	Q034U8	LSEI_2551	CBS domain containing protein
26,6378	26,5741	26,7111	26,4759	26,7371	26,5199	26,8618	26,7902	26,5918	26,9144		18	18	18	20	20	20	161,52	1,33E+09	60	26,6814	26,6272	26,7356	10	5	5	0,264248	0,108406	Q038M0	Q038M0	polC	DNA polymerase III PolC-type
26,3268	26,0887	25,6417	25,9893	25,7204	26,5577	26,7756	26,8407	26,7189	26,9439	+	6	6	6	19,2	19,2	19,2	46,69	4,11E+08	24	26,3604	25,9534	26,7674	10	5	5	0,000403136	0,813995	Q039G2	Q039G2	LSEI_1382	TPR repeats containing protein
27,3341	26,9508	26,9761	26,7038	26,8563	26,6891	26,7347	26,7336	26,8666	26,8622		11	11	11	27	27	27	37,306	2,11E+09	81	26,8707	26,9642	26,7772	10	5	5	0,128417	-0,186982	Q039D1	Q039D1	LSEI_1413	Transcriptional regulator
25,6888	26,3981	26,3024	26,4795	26,1917	26,8159	26,6964	26,7004	27,0804	26,6151	+	7	7	7	40,6	40,6	40,6	23,938	1,12E+09	54	26,4969	26,2121	26,7817	10	5	5	0,00772656	0,569548	Q036R4	Q036R4	LSEI_2055	Predicted acyltransferase
26,5802	26,8867	26,9383	26,9778	27,0086	26,7471	26,7114	26,6952	27,0612	26,6995		12	12	12	65,5	65,5	65,5	25,017	3,67E+09	98	26,8306	26,8783	26,7829	10	5	5	0,387241	-0,095443	Q034H8	Q034H8	rpiA	Ribose-5-phosphate isomerase A
26,9345	27,0262	27,019	27,0051	27,1369	26,7738	26,7347	26,8367	26,8135	26,8007	+	23	23	23	49,1	49,1	49,1	48,815	5,17E+09	200	26,9081	27,0243	26,7919	10	5	5	0,000234606	-0,232491	Q037Y8	Q037Y8	murC	UDP-N-acetylmuramate--L-alanine ligase
26,7979	26,9933	26,9743	27,0121	26,9358	26,6148	26,8108	26,8176	26,9878	26,7553		21	21	21	58,1	58,1	58,1	44,698	4,28E+09	140	26,87	26,9427	26,7973	10	5	5	0,0753587	-0,145418	Q03AS8	Q03AS8	LSEI_0894	Bifunctional PLP-dependent enzyme with beta-cystathionase and maltose regulon repressor activities
26,8947	26,8371	26,4553	26,6003	26,6645	26,7308	26,7794	26,6407	27,098	26,7405		12	12	12	61,4	61,4	61,4	29,122	1,89E+09	75	26,7441	26,6904	26,7979	10	5	5	0,364721	0,107481	Q03B77	Q03B77	LSEI_0705	ABC-type uncharacterized transport system, ATPase component
26,5667	27,0243	26,8902	26,9492	26,7638	26,5255	26,7722	26,7653	26,9282	27,0122		2	2	2	10,8	10,8	10,8	20,614	6,16E+08	27	26,8198	26,8388	26,8007	10	5	5	0,750107	-0,0381424	Q033Z1	Q033Z1	LSEI_2767	Amidase
27,024	26,9066	26,8187	26,6776	26,7781	26,7132	26,7853	26,8236	26,7732	26,9266		29	29	29	50,8	50,8	50,8	72,485	4,72E+09	227	26,8227	26,841	26,8044	10	5	5	0,607567	-0,0366203	Q03D51	Q03D51	gyrB	DNA gyrase subunit B
25,5139	26,5591	26,4987	26,5904	26,3941	26,7839	26,7782	26,717	26,9859	26,7691	+	4	4	4	34,4	34,4	34,4	14,371	6,66E+08	30	26,559	26,3113	26,8068	10	5	5	0,0438685	0,495567	Q038Q1	Q038Q1	LSEI_1546	Uncharacterized protein
25,7758	26,6068	26,8113	27,0639	26,8341	26,5988	26,8075	26,6674	27,0835	26,8888		10	10	10	61,9	61,9	61,9	16,187	2,29E+09	81	26,7138	26,6184	26,8092	10	5	5	0,446966	0,190797	Q038S7	Q038S7	LSEI_1520	GatB/YqeY domain protein
27,0183	27,2113	27,1023	27,1147	26,9983	26,6162	26,7261	26,9061	26,7819	27,0212	+	33	33	33	46,2	46,2	46,2	94,396	5,98E+09	240	26,9496	27,089	26,8103	10	5	5	0,00831364	-0,278663	Q03BV1	Q03BV1	LSEI_0470	Aminopeptidase
25,5781	27,3473	27,1428	27,7013	26,882	26,9919	26,9115	26,5236	26,8126	26,9431		4	4	4	39,8	39,8	39,8	13,531	9,98E+08	27	26,8834	26,9303	26,8365	10	5	5	0,807914	-0,0937565	Q034S0	Q034S0	mscL	Large-conductance mechanosensitive channel
26,2602	26,2708	26,4353	26,4644	26,3899	26,7594	26,9136	26,915	27,0069	26,7796	+	15	15	15	45,9	45,9	45,9	40,681	2,75E+09	119	26,6195	26,3641	26,8749	10	5	5	3,76E-05	0,510783	Q03AG7	Q03AG7	LSEI_1009	Spermidine/putrescine-binding periplasmic protein
27,0629	26,9082	26,7534	26,886	26,9347	26,9801	26,8569	26,8364	26,8263	26,9408		15	15	15	41,3	41,3	41,3	51,332	3,31E+09	113	26,8986	26,909	26,8881	10	5	5	0,728244	-0,0209545	Q03AC0	Q03AC0	gatA	Glutamyl-tRNA(Gln) amidotransferase subunit A
25,8426	26,9058	27,2376	27,4644	27,5275	27,0051	26,8197	26,6889	27,0164	26,9919		11	11	11	61,1	61,1	61,1	20,556	1,56E+09	55	26,95	26,9956	26,9044	10	5	5	0,77953	-0,091164	Q03BZ6	Q03BZ6	LSEI_0420	Dihydroxyacetone kinase DhaL subunit
26,8946	27,0644	27,0663	26,8919	26,9898	26,8516	26,796	26,903	26,9427	27,0454		23	23	23	43,6	43,6	43,6	59,781	4,58E+09	147	26,9446	26,9814	26,9077	10	5	5	0,234259	-0,0736416	Q034S8	Q034S8	pyrG	CTP synthase
27,1584	27,1835	27,2904	27,5042	27,4494	26,9421	26,8352	26,9413	27,0623	26,8924	+	18	18	18	59,3	59,3	59,3	35,318	6,75E+09	171	27,1259	27,3172	26,9347	10	5	5	0,00127019	-0,382512	Q033T0	Q033T0	LSEI_2829	EIIAB-Man
27,2397	27,1374	27,0619	27,0249	27,1027	26,9119	26,9139	26,9276	27,0203	26,9765	+	25	25	25	56,7	56,7	56,7	48,62	5,52E+09	210	27,0317	27,1133	26,95	10	5	5	0,0049062	-0,163265	Q037M7	Q037M7	serS	Serine--tRNA ligase
27,3719	26,9606	26,8127	26,887	26,8691	26,8669	26,904	27,0093	26,8534	27,1391		11	11	11	34	34	34	43,027	1,96E+09	60	26,9674	26,9803	26,9546	10	5	5	0,82729	-0,025721	Q034K4	Q034K4	recA	Protein RecA
27,2029	27,3599	26,907	26,9116	26,9694	27,1895	26,459	26,8248	27,1357	27,2035		9	9	9	45,6	45,6	45,6	25,86	2,73E+09	51	27,0163	27,0702	26,9625	10	5	5	0,543347	-0,107668	Q038L4	Q038L4	pyrH	Uridylate kinase
27,0991	26,9208	27,0054	27,1202	27,0458	26,854	26,7897	26,9718	27,1915	27,0059		13	13	13	35,4	35,4	35,4	33,723	2,72E+09	103	27,0004	27,0383	26,9626	10	5	5	0,359775	-0,0756855	Q03A19	Q03A19	atpG	ATP synthase gamma chain
26,9123	26,9791	27,0462	27,1102	27,1173	26,8555	26,9006	27,0457	27,1465	26,8931		9	9	9	29,7	29,7	29,7	40,347	2,38E+09	44	27,0007	27,033	26,9683	10	5	5	0,36622	-0,0647541	Q03AX8	Q03AX8	LSEI_0808	Xaa-Pro aminopeptidase, Metallo peptidase, MEROPS family M24B
27,4513	27,2966	27,2313	27,266	27,1848	26,9269	26,8847	26,974	27,0518	27,0082	+	10	10	9	35,9	35,9	33,6	43,261	2,35E+09	92	27,1276	27,286	26,9691	10	5	5	0,000375475	-0,316877	Q03CP2	Q03CP2	ackA	Acetate kinase
27,2707	27,3622	27,2888	27,3839	27,3125	26,805	26,905	26,8541	27,268	27,0201	+	16	16	16	62,2	62,2	62,2	35,378	4,11E+09	165	27,147	27,3236	26,9704	10	5	5	0,0032486	-0,353162	Q039N5	Q039N5	LSEI_1306	Acetoin dehydrogenase complex, E1 component, beta subunit
25,0936	25,3581	25,2897	25,7544	25,6456	26,8585	26,9877	27,2145	26,5347	27,3007	+	5	5	5	31,6	31,6	31,6	23,027	8,70E+08	37	26,2038	25,4283	26,9792	10	5	5	2,74E-05	1,55093	Q03CG0	Q03CG0	LSEI_0249	Cell wall-associated hydrolase
30,0504	27,0187	27,1845	27,2695	27,4758	27,1835	26,9753	26,7397	27,0524	27,1046		6	6	6	23,9	23,9	23,9	27,106	3,16E+09	85	27,4054	27,7998	27,0111	10	5	5	0,205613	-0,788708	Q03AF5	Q03AF5	LSEI_1022	Probable transcriptional regulatory protein LSEI_1022
27,2636	27,3767	27,3307	27,1673	27,2234	26,9234	27,0426	26,9883	27,2572	26,9426	+	17	17	17	45	45	45	45,242	5,46E+09	139	27,1516	27,2724	27,0308	10	5	5	0,00921446	-0,241562	Q036L4	Q036L4	LSEI_2108	Cyclopropane-fatty-acyl-phospholipid synthase
27,1478	26,8252	26,9513	27,018	27,1357	26,8893	26,9563	27,042	27,004	27,2713		23	23	23	42,9	42,9	42,9	52,024	4,75E+09	181	27,0241	27,0156	27,0326	10	5	5	0,852742	0,0169643	Q039D9	Q039D9	LSEI_1405	ATP-dependent protease HslVU (ClpYQ), ATPase subunit
27,4447	27,2087	27,2712	27,3958	27,4059	26,942	26,9803	27,0311	27,2129	27,031	+	14	14	14	47,9	47,9	47,9	37,025	3,48E+09	104	27,1924	27,3453	27,0395	10	5	5	0,00147488	-0,305819	Q034L5	Q034L5	LSEI_2638	Aryl-alcohol dehydrogenase related enzyme
26,6497	27,0289	26,8424	26,9659	26,7081	26,8777	27,2513	27,0479	27,2867	26,8535		4	4	4	80,3	80,3	80,3	7,2028	1,77E+09	26	26,9512	26,839	27,0634	10	5	5	0,0890185	0,224453	Q03BE5	Q03BE5	LSEI_0636	Cold-shock DNA-binding protein family
26,6931	26,9441	27,0479	27,0819	26,9432	27,08	26,8974	27,0703	27,3319	26,9692		8	8	8	45,9	45,9	45,9	20,304	1,38E+09	53	27,0059	26,9421	27,0697	10	5	5	0,23901	0,127666	Q034V8	Q034V8	LSEI_2541	Hypoxanthine phosphoribosyltransferase
26,0297	25,9215	26,2119	26,472	26,1241	26,8855	27,1744	27,0779	27,0901	27,1368	+	9	9	9	38	38	38	36,625	1,15E+09	33	26,6124	26,1519	27,0729	10	5	5	2,39E-05	0,921085	Q036W4	Q036W4	LSEI_2000	Predicted xylanase/chitin deacetylase
27,1231	26,9812	27,0688	27,1683	27,1546	26,9873	27,0452	26,9447	27,3625	27,0954		8	8	8	39,9	39,9	39,9	27,325	1,81E+09	55	27,0931	27,0992	27,087	10	5	5	0,884343	-0,0121624	Q039J8	Q039J8	LSEI_1345	Amino acid ABC transporter ATP-binding protein, PAAT family
26,8225	26,6488	26,6755	26,6573	26,7488	26,8687	27,2718	27,203	27,013	27,0989	+	22	22	22	37,6	37,6	37,6	76,937	3,60E+09	137	26,9008	26,7106	27,0911	10	5	5	0,00125884	0,380485	Q039S0	Q039S0	LSEI_1269	Cell division protein FtsI/penicillin-binding protein 2
26,9418	26,9961	27,176	27,5017	27,3011	27,0887	26,8483	27,0363	27,323	27,1814		10	10	10	53,8	53,8	53,8	17,172	2,69E+09	72	27,1394	27,1833	27,0955	10	5	5	0,515139	-0,0877903	Q038D1	Q038D1	greA	Transcription elongation factor GreA
26,5267	26,5481	26,3214	26,2445	26,4978	26,9861	27,1194	27,0252	26,998	27,3654	+	7	7	7	28,4	28,4	28,4	34,981	8,86E+08	39	26,7633	26,4277	27,0988	10	5	5	9,26E-05	0,671106	Q037Q3	Q037Q3	LSEI_1799	ABC-type uncharacterized transport system, periplasmic component
27,4451	27,2661	27,1587	26,8572	27,0124	27,1438	26,9748	27,0716	27,2172	27,1002		10	10	10	36,6	36,6	36,6	33,85	2,52E+09	87	27,1247	27,1479	27,1015	10	5	5	0,68172	-0,0463509	Q039Z5	Q039Z5	LSEI_1190	Transcriptional regulator, xre family
26,9098	27,3282	27,1949	27,3223	27,3758	27,1278	27,0502	27,0042	27,4118	26,9325		8	8	8	66,2	66,2	66,2	16,22	2,70E+09	98	27,1657	27,2262	27,1053	10	5	5	0,337434	-0,120881	Q036B2	Q036B2	LSEI_2213	Universal stress protein
27,1491	27,2535	27,1218	27,2461	27,0119	27,2596	27,0121	27,1382	27,2182	26,9568		14	14	14	56,8	56,8	56,8	34,22	2,99E+09	111	27,1367	27,1565	27,117	10	5	5	0,604446	-0,039489	Q035E1	Q035E1	LSEI_2445	ABC-type phosphate/phosphonate transport system, periplasmic component
27,231	27,4192	27,4415	27,5194	27,4668	27,0208	27,1086	27,1167	27,238	27,147	+	11	11	11	54,7	54,7	54,7	30,774	4,99E+09	129	27,2709	27,4156	27,1262	10	5	5	0,00135098	-0,289365	Q03B28	Q03B28	LSEI_0758	Aldo/keto reductase related enzyme
26,3736	26,8436	26,8743	26,8899	26,7694	26,9708	27,144	26,9765	27,3979	27,1713	+	8	8	8	33,5	33,5	33,5	22,827	2,23E+09	66	26,9411	26,7502	27,1321	10	5	5	0,0152118	0,381925	Q038Y1	Q038Y1	LSEI_1463	Predicted oxidoreductase
27,2954	27,5628	27,4111	27,3309	27,3177	27,034	27,1409	27,1692	27,0941	27,3631	+	37	37	37	52	52	52	97,252	8,05E+09	244	27,2719	27,3836	27,1603	10	5	5	0,0166698	-0,223329	Q03B02	Q03B02	alaS	Alanine--tRNA ligase
25,2745	25,4816	25,6011	25,5997	25,5367	26,9798	27,3167	27,2853	26,8278	27,4554	+	20	20	20	12,4	12,4	12,4	240,37	1,91E+09	89	26,3359	25,4987	27,173	10	5	5	1,31E-06	1,67428	Q034X4	Q034X4	LSEI_2512	Predicted outer membrane protein
27,3957	27,4908	27,3908	27,3872	27,3354	27,0227	27,0695	27,2043	27,3179	27,3886	+	17	17	17	44	44	44	50,09	5,55E+09	162	27,3003	27,4	27,2006	10	5	5	0,0278977	-0,199358	Q038W1	Q038W1	asnS	Asparagine--tRNA ligase
27,3983	27,3241	27,2984	27,3188	27,4173	27,1801	27,1756	27,2471	27,0657	27,4912		50	49	49	67,3	67,3	67,3	77,249	1,33E+10	433	27,2917	27,3514	27,2319	10	5	5	0,1493	-0,119447	Q037T8	Q037T8	LSEI_1762	ATP-binding subunit of Clp protease and DnaK/DnaJ chaperones
27,904	27,6784	27,533	27,5982	27,4586	27,3056	27,2955	27,3901	27,4411	26,7483	+	6	6	6	49,4	49,4	49,4	17,321	2,01E+09	54	27,4353	27,6345	27,2361	10	5	5	0,0262829	-0,398331	Q038A0	Q038A0	infC	Translation initiation factor IF-3
26,5502	26,9944	27,0022	26,9789	26,8779	27,1208	27,3321	27,1632	27,5438	27,1662	+	22	22	22	54,6	54,6	54,6	46,541	4,06E+09	138	27,073	26,8807	27,2652	10	5	5	0,0106835	0,38449	Q033S2	Q033S2	LSEI_2840	Carbohydrate ABC transporter substrate-binding protein, CUT1 family
26,4187	27,6435	27,6699	28,0334	27,5587	27,0938	27,3089	27,253	27,4639	27,2274		8	8	8	60,8	60,8	60,8	17,816	2,75E+09	64	27,3671	27,4648	27,2694	10	5	5	0,505538	-0,195459	Q03B95	Q03B95	LSEI_0687	Heat shock protein Hsp20
27,4605	27,3886	27,3743	27,3084	27,4535	27,1114	27,2206	27,4106	27,2217	27,3888		31	31	31	63,8	63,8	63,8	57,881	7,92E+09	270	27,3338	27,3971	27,2706	10	5	5	0,079745	-0,12646	Q036Y5	Q036Y5	guaA	GMP synthase [glutamine-hydrolyzing]
27,6924	27,4605	27,3556	27,4924	27,515	27,0957	27,2811	27,2927	27,4267	27,3025	+	14	14	14	45,5	45,5	45,5	32,571	4,36E+09	105	27,3915	27,5032	27,2797	10	5	5	0,0188254	-0,223419	Q034P5	Q034P5	LSEI_2607	Malate dehydrogenase (NAD)
26,4416	26,2044	26,5214	26,258	26,4021	27,1117	27,5643	27,5168	27,1488	27,1057	+	9	9	9	22	22	22	40,172	1,41E+09	83	26,8275	26,3655	27,2894	10	5	5	5,28E-05	0,923924	Q03CG2	Q03CG2	LSEI_0247	Transcriptional regulator
27,588	27,7558	27,6223	27,5461	27,5707	27,2398	27,2157	27,334	27,212	27,4654	+	42	42	42	63,2	63,2	63,2	89,631	1,22E+10	406	27,455	27,6166	27,2934	10	5	5	0,000719402	-0,323198	Q03CN5	Q03CN5	LSEI_0174	Probable phosphoketolase
27,2185	27,4136	27,4449	27,4983	27,3316	27,2601	27,1481	27,3092	27,5168	27,2345		7	7	7	33,4	33,4	33,4	38,362	2,77E+09	67	27,3376	27,3814	27,2937	10	5	5	0,297293	-0,0876366	Q03AI5	Q03AI5	LSEI_0991	Carbohydrate ABC transporter ATP-binding protein, CUT1 family
27,5238	27,2604	27,4127	27,422	27,6726	27,414	27,201	27,2796	27,3289	27,266		17	17	17	67,1	67,1	67,1	34,961	6,20E+09	208	27,3781	27,4583	27,2979	10	5	5	0,07014	-0,160429	Q03AI0	Q03AI0	LSEI_0996	Phosphotransacetylase
25,7405	28,0359	27,9063	28,4389	27,8405	27,256	27,333	27,0724	27,6828	27,148		11	11	11	74,8	74,8	74,8	14,283	2,01E+09	52	27,4454	27,5924	27,2985	10	5	5	0,562195	-0,293943	Q038N9	Q038N9	LSEI_1559	Uncharacterized protein
27,7186	27,5413	27,5245	27,5289	27,5074	27,3177	27,2156	27,2683	27,4507	27,2902	+	19	19	19	61,7	61,7	61,7	34,971	6,22E+09	156	27,4363	27,5641	27,3085	10	5	5	0,00171428	-0,255663	Q03A15	Q03A15	mreB	Cell shape-determining protein MreB
27,6919	27,6285	27,5621	27,734	27,6355	27,1412	27,309	27,2954	27,6265	27,214	+	11	11	11	44,7	44,7	44,7	32,412	2,82E+09	99	27,4838	27,6504	27,3172	10	5	5	0,00535743	-0,333168	Q034Q3	Q034Q3	LSEI_2598	Galactose mutarotase related enzyme
26,6505	27,1323	27,2796	27,6494	27,3128	27,3963	27,3582	27,0173	27,5551	27,313		9	9	9	48,1	48,1	48,1	17,739	3,50E+09	122	27,2664	27,2049	27,328	10	5	5	0,523632	0,123079	Q03A22	Q03A22	atpF	ATP synthase subunit b
27,001	27,8186	27,8503	28,1481	27,8185	27,1415	27,3141	27,2635	27,5928	27,3558		12	12	12	78,6	78,6	78,6	16,505	3,97E+09	117	27,5304	27,7273	27,3335	10	5	5	0,0919345	-0,393751	Q033V8	Q033V8	LSEI_2800	Heat shock protein Hsp20
27,5054	27,42	27,3806	27,4118	27,3501	27,1798	27,2837	27,3426	27,5163	27,358		22	22	22	76,1	76,1	76,1	40,375	5,54E+09	150	27,3748	27,4136	27,3361	10	5	5	0,237775	-0,0774998	Q03B16	Q03B16	queA	S-adenosylmethionine:tRNA ribosyltransferase-isomerase
27,2753	27,4367	27,3348	27,3661	27,4473	27,16	27,3389	27,4134	27,4283	27,4863		19	19	19	46,3	46,3	46,3	49,834	5,61E+09	173	27,3687	27,3721	27,3654	10	5	5	0,92086	-0,00666084	Q03C19	Q03C19	LSEI_0397	Uncharacterized NAD(FAD)-dependent dehydrogenase
26,124	27,0649	26,9757	26,9747	26,8791	27,3371	27,2987	27,1959	27,6262	27,3902	+	7	7	7	75,8	75,8	75,8	14,779	2,37E+09	67	27,0866	26,8037	27,3696	10	5	5	0,0162835	0,565948	Q03C50	Q03C50	LSEI_0363	Pyridoxine 5-phosphate oxidase V related favin-nucleotide-binding protein
25,8785	26,9852	27,0982	27,3871	27,1393	27,4808	27,2822	26,913	27,7737	27,4044		13	13	13	80,6	80,6	80,6	11,572	2,99E+09	74	27,1342	26,8977	27,3708	10	5	5	0,151155	0,473146	Q03D47	Q03D47	rpsF	30S ribosomal protein S6
27,6204	27,7015	27,6278	27,6055	27,5464	27,2628	27,3267	27,4344	27,4928	27,5493	+	37	37	37	61,8	61,8	61,8	74,898	1,03E+10	324	27,5168	27,6203	27,4132	10	5	5	0,00742036	-0,207081	Q034P8	Q034P8	metG	Methionine--tRNA ligase
27,7561	27,9442	27,7239	27,6813	27,6615	27,4499	27,4455	27,64	27,3156	27,2454	+	24	24	24	47	47	47	56,528	4,07E+09	151	27,5864	27,7534	27,4193	10	5	5	0,00416684	-0,33411	Q034W7	Q034W7	lysS	Lysine--tRNA ligase
27,3259	27,3481	27,3717	27,2642	27,3006	27,1562	27,4909	27,5868	27,5163	27,3466		12	12	12	42,9	42,9	42,9	37,826	4,49E+09	126	27,3707	27,3221	27,4194	10	5	5	0,251699	0,0972931	Q038X5	Q038X5	LSEI_1469	Ribonucleoside-diphosphate reductase
27,469	26,9412	27,4661	27,9014	27,8985	27,6505	27,3813	27,1295	27,3031	27,6982		5	5	5	28,6	28,6	28,6	18,727	2,10E+09	55	27,4839	27,5353	27,4325	10	5	5	0,633117	-0,102732	Q03AY0	Q03AY0	LSEI_0806	Uncharacterized protein
27,3369	27,0234	27,0213	27,0902	27,0706	27,3419	27,4717	27,3593	27,5433	27,5791	+	22	22	22	55,3	55,3	55,3	46,901	4,72E+09	178	27,2838	27,1085	27,4591	10	5	5	0,00166331	0,35061	Q03CI8	Q03CI8	LSEI_0221	Serine-type D-Ala-D-Ala carboxypeptidase
27,9458	27,7272	27,7387	27,8161	27,8057	27,3935	27,4888	27,4429	27,6893	27,4407	+	18	18	18	48	48	48	42,356	6,71E+09	175	27,6489	27,8067	27,491	10	5	5	0,00124291	-0,315661	Q037P4	Q037P4	LSEI_1808	N-acetylglucosamine 6-phosphate deacetylase
27,7071	27,804	27,7127	27,7015	27,6314	27,3239	27,4859	27,5605	27,4865	27,6719	+	28	28	28	43,8	43,8	43,8	81,864	8,80E+09	223	27,6085	27,7113	27,5057	10	5	5	0,0115244	-0,205595	Q038X6	Q038X6	LSEI_1468	Ribonucleoside-diphosphate reductase
28,0878	27,7154	27,5972	27,5757	27,7767	27,5263	27,4482	27,5327	27,4542	27,6276	+	27	27	27	67,1	67,1	67,1	52,64	9,88E+09	311	27,6342	27,7505	27,5178	10	5	5	0,0444216	-0,232754	Q03CJ2	Q03CJ2	guaB	Inosine-5-monophosphate dehydrogenase
27,862	27,454	27,4858	27,5474	27,5236	27,3785	27,4514	27,5444	27,5667	27,6863		21	21	21	63,3	63,3	63,3	40,274	6,33E+09	169	27,55	27,5745	27,5255	10	5	5	0,602056	-0,0490574	Q03CJ4	Q03CJ4	ychF	Ribosome-binding ATPase YchF
28,0713	28,3669	28,2141	28,1464	27,9704	27,4919	27,8485	27,5806	27,6695	27,0781	+	4	4	4	28	28	28	13,422	3,40E+09	81	27,8438	28,1538	27,5337	10	5	5	0,00266507	-0,620116	Q038A2	Q038A2	rplT	50S ribosomal protein L20
28,2337	28,4736	28,4736	28,4063	28,3001	27,4744	27,4976	27,4118	27,9891	27,4125	+	14	14	14	72	72	72	18,601	7,89E+09	171	27,9673	28,3775	27,5571	10	5	5	0,000128082	-0,820358	Q03BL4	Q03BL4	LSEI_0560	Phosphotransferase system, mannose/fructose/N-acetylgalactosamine-specific component IIB
27,2595	27,9031	27,6249	27,7172	27,7185	27,4266	27,5566	27,5516	27,6987	27,5664		10	10	10	60	60	60	17,985	2,92E+09	106	27,6023	27,6446	27,56	10	5	5	0,481775	-0,0846542	Q039Z4	Q039Z4	LSEI_1191	Universal stress protein
25,0729	27,1846	27,1527	27,2878	26,8867	27,4109	27,5093	27,2791	28,1197	27,5606		5	5	5	77,8	77,8	77,8	8,1475	1,99E+09	57	27,1464	26,7169	27,5759	10	5	5	0,0870273	0,858974	Q035A5	Q035A5	infA	Translation initiation factor IF-1
27,8906	27,4232	27,5661	27,6255	27,8489	27,745	27,5263	27,6362	27,3801	27,7271		18	18	18	53,9	53,9	53,9	44,277	4,22E+09	138	27,6369	27,6709	27,603	10	5	5	0,557754	-0,0679169	Q039R4	Q039R4	ftsZ	Cell division protein FtsZ
25,4421	25,2888	25,6477	25,7294	25,9453	27,6617	27,5621	27,5966	27,3491	27,8505	+	5	5	5	15,2	15,2	15,2	32,635	1,60E+09	67	26,6073	25,6107	27,604	10	5	5	5,68E-07	1,99332	Q035C7	Q035C7	LSEI_2459	Uncharacterized protein
27,8718	27,6531	27,7029	27,848	27,7557	27,4864	27,566	27,6757	27,6507	27,7103	+	19	19	19	45,2	45,2	45,2	48,795	4,25E+09	138	27,6921	27,7663	27,6178	10	5	5	0,0340458	-0,148498	Q03AF8	Q03AF8	glmM	Phosphoglucosamine mutase
27,7011	27,5966	27,6128	27,5987	27,6824	27,6479	27,5407	27,6188	27,5142	27,7729		27	27	27	52,5	52,5	52,5	53,594	9,44E+09	276	27,6286	27,6383	27,6189	10	5	5	0,711981	-0,0194107	Q03AN1	Q03AN1	LSEI_0942	DUF4097 domain-containing protein
27,277	28,2189	28,3775	28,1733	27,9661	27,4372	27,7151	27,5001	28,0022	27,4707		5	5	5	24,4	24,4	24,4	22,543	1,53E+09	40	27,8138	28,0025	27,6251	10	5	5	0,124755	-0,37747	Q038Z5	Q038Z5	pyrE	Orotate phosphoribosyltransferase
26,0508	26,6122	27,1733	27,9523	27,5899	27,6657	27,3304	27,3054	28,0117	27,8303		4	4	4	30,5	30,5	30,5	15,051	1,48E+09	25	27,3522	27,0757	27,6287	10	5	5	0,169983	0,553026	Q038W7	Q038W7	gpsB	Cell cycle protein GpsB
27,4551	27,3263	27,2122	27,3368	27,3937	27,5929	27,4219	27,7906	27,8016	27,5576	+	7	7	7	27,1	27,1	27,1	33,608	1,18E+09	54	27,4889	27,3448	27,6329	10	5	5	0,00840907	0,288112	Q03AL4	Q03AL4	LSEI_0959	Nucleotide-binding protein LSEI_0959
27,517	27,6238	27,5511	27,6257	27,5036	27,6137	27,5903	27,6734	27,758	27,7001	+	19	19	19	68,9	68,9	68,9	41,456	7,90E+09	222	27,6157	27,5642	27,6671	10	5	5	0,0320937	0,102875	Q03D54	Q03D54	LSEI_0002	Beta sliding clamp
26,5446	26,4271	25,5839	26,838	26,4898	28,0115	27,3693	27,6012	27,6712	27,7524	+	2	2	2	2,5	2,5	2,5	102,35	8,79E+08	12	27,0289	26,3767	27,6811	10	5	5	0,000537709	1,30445	Q03C76	Q03C76	LSEI_0336	Predicted membrane protein

27,4659	27,7073	27,6098	27,803	27,8444	27,6648	27,6063	27,6999	27,7056	27,7427		13	13	13	70,2	70,2	70,2	16,961	4,56E+09	127	27,685	27,6861	27,6839	10	5	5	0,976411	-0,00220032	Q03CU2	Q03CU2	rplI	50S ribosomal protein L9
27,0519	27,1639	27,318	27,4956	27,2823	27,6122	27,6549	27,5361	27,8286	27,828	+	7	7	7	35,6	35,6	35,6	22,012	2,85E+09	79	27,4771	27,2623	27,692	10	5	5	0,001964	0,429606	Q034M9	Q034M9	LSEI_2623	Uncharacterized protein
28,6527	27,1271	26,729	27,3031	27,3302	27,6348	27,6555	27,5222	27,9144	27,7487		3	3	3	37,7	37,7	37,7	6,9771	1,06E+09	39	27,5618	27,4284	27,6951	10	5	5	0,443617	0,266687	Q038I0	Q038I0	rpmB	50S ribosomal protein L28
26,8021	27,0527	27,0293	27,0064	27,1127	27,5121	27,7701	27,7549	27,7106	27,764	+	7	7	7	22,6	22,6	22,6	44,95	1,61E+09	46	27,3515	27,0006	27,7023	10	5	5	1,00E-05	0,701727	Q033V6	Q033V6	LSEI_2802	Trypsin-like serine protease with PDZ domain
26,6333	26,6943	26,6148	26,6447	26,5665	27,6055	27,8297	27,7791	27,316	28,0508	+	28	28	28	37,5	37,5	37,5	100,41	5,06E+09	159	27,1735	26,6307	27,7162	10	5	5	2,33E-05	1,08551	Q03AX6	Q03AX6	LSEI_0810	DD-transpeptidase
27,1241	28,2138	28,3518	28,5454	28,3007	27,8318	27,6824	27,464	28,229	27,4633		6	6	6	57,3	57,3	57,3	10,215	4,47E+09	65	27,9206	28,1072	27,7341	10	5	5	0,23284	-0,373059	Q03AR2	Q03AR2	rpsN	30S ribosomal protein S14
27,5885	27,0415	27,4017	27,772	27,5974	27,8266	27,6569	27,5777	27,7424	27,8986		9	9	9	41,6	41,6	41,6	20,593	2,48E+09	75	27,6103	27,4802	27,7404	10	5	5	0,0939616	0,260239	Q038L5	Q038L5	frr	Ribosome-recycling factor
28,1804	28,2472	28,1202	28,0104	28,0442	27,5163	27,7171	27,7556	27,6988	28,0503	+	45	45	45	47,5	47,5	47,5	115,97	5,85E+09	235	27,934	28,1205	27,7476	10	5	5	0,00476808	-0,372882	Q038Z2	Q038Z2	carB	Carbamoyl-phosphate synthase large chain
27,7684	28,0972	28,0066	28,1002	28,0655	27,7997	27,7211	27,6905	28,0642	27,4827		12	12	12	67,9	67,9	67,9	22,808	5,81E+09	140	27,8796	28,0076	27,7516	10	5	5	0,052877	-0,255932	Q03A25	Q03A25	upp	Uracil phosphoribosyltransferase
27,3191	27,0481	27,4917	27,6024	27,3529	27,8091	27,7747	27,6857	27,6584	27,9346	+	7	7	7	82,2	82,2	82,2	8,5436	3,24E+09	122	27,5677	27,3628	27,7725	10	5	5	0,00468941	0,409672	Q035B9	Q035B9	LSEI_2467	Uncharacterized protein
27,9889	27,9622	27,8371	27,8351	27,881	27,6907	27,7413	27,8287	27,6395	27,9781		49	49	49	67,5	67,5	67,5	91,733	9,28E+09	306	27,8383	27,9009	27,7757	10	5	5	0,1006	-0,125187	Q034W9	Q034W9	LSEI_2517	ATP-binding subunit of Clp protease and DnaK/DnaJ chaperones
27,3276	27,5894	27,7022	27,6391	27,7003	27,8102	27,6244	27,7132	27,9549	27,843		10	10	10	57,2	57,2	57,2	19,607	4,90E+09	107	27,6904	27,5917	27,7891	10	5	5	0,0581815	0,197416	Q035A4	Q035A4	adk	Adenylate kinase
28,0949	28,1373	28,0327	28,1055	28,0728	27,6353	27,8688	27,7298	27,9053	27,8188	+	17	17	17	46,2	46,2	46,2	43	7,39E+09	186	27,9401	28,0886	27,7916	10	5	5	0,000444344	-0,29702	Q03AU4	Q03AU4	metK	S-adenosylmethionine synthase
27,9697	27,9284	27,8582	27,8085	27,8046	27,7328	27,8193	27,9827	27,3357	28,1076		86	86	86	68,2	68,2	68,2	136,46	1,96E+10	570	27,8348	27,8739	27,7956	10	5	5	0,580768	-0,0782642	Q034X1	Q034X1	rpoC	DNA-directed RNA polymerase subunit beta
29,9356	28,2566	28,0444	28,4313	28,0738	27,7197	27,9437	27,6079	28,0869	27,7128		4	4	4	19,4	19,4	19,4	15,177	2,88E+09	40	28,1813	28,5484	27,8142	10	5	5	0,0786946	-0,73414	Q03B14	Q03B14	LSEI_0772	Protein translocase subunit yajC
27,8347	27,8555	27,8055	27,8286	27,7854	27,803	27,7891	27,8081	27,7486	27,9573		31	31	31	44,3	44,3	44,3	77,741	7,69E+09	244	27,8216	27,8219	27,8212	10	5	5	0,985129	-0,00072289	Q034V9	Q034V9	ftsH	ATP-dependent zinc metalloprotease FtsH
27,0335	26,9936	26,9754	26,9515	26,9201	27,2415	28,1091	28,1617	27,6391	28,0255	+	6	6	6	31,4	31,4	31,4	25,448	2,19E+09	72	27,4051	26,9748	27,8354	10	5	5	0,00118859	0,860578	Q033J7	Q033J7	LSEI_A08	Acetoin/pyruvate dehydrogenase complex, E3 component, dihydrolipoamide dehydrogenase
28,8782	28,2034	28,0411	28,1007	27,9486	27,6488	27,9669	27,9984	27,9751	27,5986		13	13	13	53,8	53,8	53,8	36,905	5,14E+09	80	28,036	28,2344	27,8375	10	5	5	0,0677156	-0,39686	Q03CR3	Q03CR3	LSEI_0145	(R)-2-hydroxyisocaproate dehydrogenase
26,0744	26,7699	26,969	27,2745	26,8132	27,7279	27,8616	27,5207	28,1865	27,9095	+	4	4	4	39,3	39,3	39,3	9,3168	1,64E+09	41	27,3107	26,7802	27,8412	10	5	5	0,00154138	1,06102	Q039L4	Q039L4	rpsT	30S ribosomal protein S20
27,6523	27,8526	27,8637	27,803	27,7747	27,6944	27,8533	27,9092	27,9876	27,8202		12	12	12	53,5	53,5	53,5	26,318	5,73E+09	137	27,8211	27,7893	27,8529	10	5	5	0,332669	0,0636646	Q038C0	Q038C0	LSEI_1679	DNA-binding response regulator, OmpR family (Rec-wHTH domains)
27,6169	28,2305	28,1658	28,2181	28,0009	27,7441	27,7343	27,7804	28,1307	27,8808		9	9	9	38,3	38,3	38,3	33,473	4,71E+09	108	27,9503	28,0465	27,8541	10	5	5	0,196687	-0,192394	Q033T2	Q033T2	LSEI_2827	PTS system D-glucose-specific IID component, Man family
26,7262	26,9072	26,9464	26,9823	26,9041	27,7607	27,8598	28,0533	27,7693	27,9123	+	17	17	17	43,9	43,9	43,9	50,413	4,53E+09	119	27,3822	26,8933	27,8711	10	5	5	6,31E-07	0,977796	Q034U9	Q034U9	LSEI_2550	Peptidoglycan transpeptidase, ErfK-YbiS-YhnG family
28,0526	28,0897	28,0331	28,1123	28,2204	27,904	27,7195	27,9523	27,9176	27,8672	+	26	26	26	60,3	60,3	60,3	47,391	1,15E+10	239	27,9869	28,1016	27,8721	10	5	5	0,0022692	-0,229525	Q03CT6	Q03CT6	purA	Adenylosuccinate synthetase
27,3704	28,2109	27,9589	27,987	27,8624	28,0292	27,9582	27,8541	27,7689	27,7832		9	9	9	66,8	66,8	66,8	23,083	2,44E+09	71	27,8783	27,8779	27,8787	10	5	5	0,995793	0,0008049	Q03A95	Q03A95	LSEI_1084	Transcriptional regulator, TetR family
28,3675	27,8745	28,008	28,0163	27,8052	27,9644	27,8641	28,0049	27,7494	28,1325		5	5	5	28,9	28,9	28,9	20,914	2,82E+09	39	27,9787	28,0143	27,943	10	5	5	0,558104	-0,0712578	Q038F6	Q038F6	efp	Elongation factor P
28,2471	28,228	28,2663	28,2032	28,1451	27,8126	28,0166	28,0222	28,0415	28,0433	+	30	30	30	60,7	60,7	60,7	50,601	1,19E+10	293	28,1026	28,2179	27,9872	10	5	5	0,0014727	-0,230737	Q035P9	Q035P9	LSEI_2329	Aminopeptidase
27,8898	27,8856	27,9161	27,9049	27,87	27,9007	28,0458	28,145	27,5742	28,3568		56	56	56	50,3	50,3	50,3	133,7	2,11E+10	490	27,9489	27,8933	28,0045	10	5	5	0,420297	0,111207	Q034X0	Q034X0	rpoB	DNA-directed RNA polymerase subunit beta
26,6432	26,6109	26,8601	26,7203	26,982	28,2108	27,9988	27,9504	27,4744	28,3965	+	5	5	5	29,4	29,4	29,4	31,677	2,49E+09	47	27,3847	26,7633	28,0062	10	5	5	8,24E-05	1,24291	Q03BH5	Q03BH5	LSEI_0603	Uncharacterized protein
28,2692	28,3416	28,3309	28,3151	28,2677	27,8548	27,9767	28,0606	28,0463	28,1243	+	34	34	34	60,3	60,3	60,3	63,565	1,28E+10	308	28,1587	28,3049	28,0126	10	5	5	0,000309905	-0,292331	Q03AM4	Q03AM4	LSEI_0949	Alpha-phosphoglucomutase
28,0507	28,2598	28,2269	28,2174	28,1181	27,9451	27,966	28,0197	28,2617	28,1179		31	31	31	63	63	63	50,426	1,59E+10	364	28,1183	28,1746	28,0621	10	5	5	0,146763	-0,112519	Q038E7	Q038E7	LSEI_1652	Glutamine synthetase
28,2542	28,0925	28,1003	28,0278	28,0507	27,9248	28,0129	28,1454	28,1991	28,1409		24	24	24	45,1	45,1	45,1	56,87	9,91E+09	262	28,0948	28,1051	28,0846	10	5	5	0,757327	-0,0204765	Q035R5	Q035R5	gltX	Glutamate--tRNA ligase
28,3839	27,9784	27,9672	27,9584	28,1886	28,2445	27,8832	28,188	28,0647	28,1327		20	20	20	63,1	63,1	63,1	34,281	6,51E+09	191	28,099	28,0953	28,1026	10	5	5	0,945855	0,00732765	Q036K5	Q036K5	LSEI_2117	Dioxygenase
28,1203	28,0121	28,1144	28,2884	28,31	27,9978	28,0424	28,1475	28,2441	28,0872		19	19	19	67,4	67,4	67,4	33,818	1,12E+10	210	28,1364	28,169	28,1038	10	5	5	0,385614	-0,0652039	Q03A86	Q03A86	LSEI_1093	UTP--glucose-1-phosphate uridylyltransferase
28,3125	28,2302	28,1801	28,0916	27,9443	27,9604	28,0506	28,1255	28,2588	28,1476		18	18	18	70,3	70,3	70,3	39,02	9,01E+09	237	28,1302	28,1517	28,1086	10	5	5	0,605573	-0,0431862	Q03CR5	Q03CR5	ddl	D-alanine--D-alanine ligase
28,0621	27,9889	28,1994	28,2925	28,3323	28,1485	27,9793	28,1195	28,2127	28,1674		13	13	13	60,7	60,7	60,7	30,087	5,74E+09	136	28,1503	28,1751	28,1255	10	5	5	0,536517	-0,0495583	Q037P8	Q037P8	nadE	NH(3)-dependent NAD(+) synthetase
28,3148	28,3362	28,1997	28,2654	28,1524	28,1413	28,1225	28,1464	28,1626	28,167	+	16	16	16	23,2	23,2	23,2	70,003	8,82E+09	134	28,2008	28,2537	28,148	10	5	5	0,0175087	-0,105743	Q039J6	Q039J6	LSEI_1347	PTS system D-fructose-specific IIC component (F1P-forming), Frc family / PTS system D-fructose-specific IIB component (F1P-forming), Frc family
27,5601	28,5416	28,5018	28,6652	28,8351	28,1612	28,0179	27,9961	28,5076	28,1143		9	9	9	66,2	66,2	66,2	17,266	5,20E+09	93	28,2901	28,4207	28,1594	10	5	5	0,310135	-0,261328	Q03B21	Q03B21	luxS	S-ribosylhomocysteine lyase
28,206	28,3268	28,2083	28,1037	28,2699	27,9409	28,137	28,1633	28,3793	28,2878		16	16	16	27,5	27,5	27,5	86,541	1,90E+09	59	28,2023	28,2229	28,1816	10	5	5	0,632852	-0,0412941	Q03BF5	Q03BF5	metE	5-methyltetrahydropteroyltriglutamate--homocysteine methyltransferase
28,4674	28,4825	28,3683	28,2879	28,3178	28,1318	28,1456	28,2452	28,2077	28,2432	+	30	30	30	56,6	56,6	56,6	65,811	2,21E+10	401	28,2897	28,3848	28,1947	10	5	5	0,00320876	-0,190097	Q03AF7	Q03AF7	glmS	Glutamine--fructose-6-phosphate aminotransferase [isomerizing]
28,0526	28,21	28,0878	28,3189	28,0214	28,3247	28,1998	28,0054	28,5212	28,2708		2	2	2	32,8	32,8	32,8	7,1225	2,43E+09	83	28,2013	28,1382	28,2644	10	5	5	0,244801	0,126206	Q034Z6	Q034Z6	rpsZ	30S ribosomal protein S14 type Z
28,0691	28,1288	28,2641	28,389	28,1672	28,0281	28,6813	28,3257	28,274	28,0632		8	8	8	15,8	15,8	15,8	50,318	1,46E+09	23	28,239	28,2036	28,2744	10	5	5	0,600085	0,0708054	Q038G0	Q038G0	xseA	Exodeoxyribonuclease 7 large subunit
28,1032	28,2468	28,2241	28,3541	28,3937	28,1064	28,2618	28,3218	28,4166	28,2884		15	15	15	54,9	54,9	54,9	31,649	7,74E+09	156	28,2717	28,2644	28,279	10	5	5	0,844164	0,0146137	Q03CI4	Q03CI4	LSEI_0225	Aldo/keto reductase of diketogulonate reductase family
28,5051	28,5865	28,4878	28,5288	28,4993	28,1331	28,2623	28,3039	28,3362	28,3783	+	32	32	32	72	72	72	52,17	1,66E+10	383	28,4021	28,5215	28,2828	10	5	5	0,000776668	-0,238756	Q038B8	Q038B8	LSEI_1681	6-phosphogluconate dehydrogenase, decarboxylating
28,4858	28,4716	28,5964	28,5402	28,48	28,2229	28,3087	28,3308	28,3928	28,2422	+	13	13	13	47,8	47,8	47,8	35,442	5,78E+09	133	28,4072	28,5148	28,2995	10	5	5	0,000542915	-0,215329	Q034R6	Q034R6	prs	Ribose-phosphate pyrophosphokinase
29,8658	28,4093	28,4954	28,5275	28,4729	28,4078	28,252	28,2394	28,4019	28,2867		12	12	12	61,7	61,7	61,7	21,524	7,23E+09	152	28,5359	28,7542	28,3175	10	5	5	0,158786	-0,436625	Q03AL0	Q03AL0	clpP	ATP-dependent Clp protease proteolytic subunit
26,5865	27,1642	27,2835	27,5956	27,5	28,3032	28,536	28,2644	27,8357	28,8119	+	9	9	9	12,2	12,2	12,2	107,93	1,75E+09	72	27,7881	27,2259	28,3502	10	5	5	0,00156313	1,12428	Q03BL0	Q03BL0	LSEI_0564	Beta-fructosidase (Levanase/invertase)
27,6919	27,5205	27,6247	27,741	27,9466	28,3106	28,3554	28,1776	28,1756	28,8656	+	11	11	11	6,3	6,3	6,3	193,89	1,25E+09	37	28,041	27,7049	28,377	10	5	5	0,00172282	0,672046	Q033Y9	Q033Y9	LSEI_2769	Alpha-glucosidase, family 31 of glycosyl hydrolase
28,7375	28,6054	28,5109	28,464	28,5195	28,2514	28,3288	28,3973	28,4065	28,5076	+	40	40	40	60,9	60,9	60,9	74,615	1,69E+10	427	28,4729	28,5675	28,3783	10	5	5	0,0188142	-0,189181	Q037Z6	Q037Z6	thrS	Threonine--tRNA ligase
25,1702	26,4237	26,5132	26,7631	26,3952	28,1891	28,3819	28,2137	28,7511	28,5643	+	4	4	4	27,1	27,1	27,1	15,075	1,43E+09	33	27,3365	26,2531	28,42	10	5	5	8,65E-05	2,16694	Q034V5	Q034V5	LSEI_2544	Septum formation initiator
27,9747	28,45	28,34	28,5022	27,8473	28,1366	28,2105	28,1171	28,6784	28,9622		6	6	6	62,5	62,5	62,5	7,4786	4,45E+09	80	28,3219	28,2228	28,421	10	5	5	0,383247	0,198145	Q034Z1	Q034Z1	rpmC	50S ribosomal protein L29
28,4453	28,5562	28,489	28,4267	28,3789	28,3053	28,341	28,3708	28,5937	28,5215		11	11	11	52,4	52,4	52,4	20,998	5,93E+09	139	28,4428	28,4592	28,4265	10	5	5	0,619021	-0,0327194	Q03AP5	Q03AP5	hpf	Ribosome hibernation promoting factor
28,3913	28,4005	28,303	28,2296	28,3413	28,6817	28,3086	28,3957	28,524	28,4141		19	19	19	78	78	78	32,718	1,25E+10	247	28,399	28,3331	28,4648	10	5	5	0,102239	0,131714	Q03BU1	Q03BU1	LSEI_0480	Cysteine synthase
28,9254	28,5566	28,581	28,6585	28,6356	28,4568	28,4413	28,5259	28,6284	28,3294	+	19	19	19	69,4	69,4	69,4	36,402	7,45E+09	186	28,5739	28,6714	28,4763	10	5	5	0,045586	-0,195077	Q03AX7	Q03AX7	LSEI_0809	Catabolite control protein A
27,4366	27,9596	28,5196	29,1381	29,005	28,5383	28,3204	28,4048	28,4606	28,7043		9	9	9	46,9	46,9	46,9	21,776	7,81E+09	125	28,4487	28,4118	28,4857	10	5	5	0,826561	0,0739159	Q038N2	Q038N2	grpE	Protein GrpE
27,4455	28,4196	28,4746	28,5872	28,4046	28,6434	28,5347	28,369	28,5781	28,3753		14	14	14	95,3	95,3	95,3	13,986	8,73E+09	166	28,3832	28,2663	28,5001	10	5	5	0,30827	0,233771	Q036B1	Q036B1	LSEI_2214	Transcriptional regulator, xre family
28,9446	28,6593	28,5656	28,5926	28,5867	28,4105	28,586	28,5092	28,4528	28,7418		25	25	25	50,3	50,3	50,3	67,942	1,58E+10	296	28,6049	28,6698	28,5401	10	5	5	0,194175	-0,129687	Q039M8	Q039M8	bipA	50S ribosomal subunit assembly factor BipA
26,6581	27,626	27,7834	27,8032	27,6459	28,4125	28,6921	28,5229	28,6608	28,5652	+	3	3	3	27,2	27,2	27,2	13,698	1,66E+09	52	28,037	27,5033	28,5707	10	5	5	0,00127045	1,06737	Q03CV5	Q03CV5	LSEI_0103	Uncharacterized protein
27,7785	28,0296	27,9677	28,0003	27,8446	28,3917	28,5777	28,4922	28,7353	28,7398	+	23	23	23	51,1	51,1	51,1	61,392	9,88E+09	218	28,2557	27,9242	28,5873	10	5	5	4,54E-05	0,663179	Q037H1	Q037H1	LSEI_1892	ABC-type oligopeptide transport system, periplasmic component
29,0146	28,7393	28,6891	28,8205	28,6792	28,5515	28,5572	28,5567	28,7862	28,4888	+	19	19	19	49	49	49	33,498	8,23E+09	157	28,6883	28,7885	28,5881	10	5	5	0,0370585	-0,200466	Q037X7	Q037X7	prsA	Foldase protein PrsA
29,631	28,4301	28,5802	28,8622	28,7076	28,6534	28,4556	28,729	28,4567	28,6949		27	27	27	71	71	71	31,7	1,16E+10	245	28,7201	28,8422	28,5979	10	5	5	0,294581	-0,244309	Q038L3	Q038L3	tsf	Elongation factor Ts
28,3141	28,8323	28,7127	28,6505	28,6565	28,5134	28,6258	28,5578	28,7812	28,5123		11	11	11	67,4	67,4	67,4	25,295	6,65E+09	122	28,6157	28,6332	28,5981	10	5	5	0,733967	-0,0351139	Q036K7	Q036K7	LSEI_2115	3-oxoacyl-[acyl-carrier-protein] reductase
28,6939	28,5701	28,7953	28,7386	28,7263	28,7297	28,5727	28,7573	28,4549	28,7423		26	26	26	78,9	78,9	78,9	52,925	1,91E+10	394	28,6781	28,7048	28,6514	10	5	5	0,467933	-0,0534618	Q03A18	Q03A18	atpD	ATP synthase subunit beta
29,0387	28,4611	28,6989	28,6666	28,9655	29,0434	28,6755	28,5356	28,4036	28,6341		7	7	7	49,3	49,3	49,3	16,037	4,89E+09	83	28,7123	28,7662	28,6585	10	5	5	0,493465	-0,107708	Q034Z0	Q034Z0	rplP	50S ribosomal protein L16
27,7192	28,4222	28,3014	27,9209	28,7208	29,1464	28,6226	28,477	28,5602	28,5977		10	10	10	55,1	55,1	55,1	9,1275	4,92E+09	109	28,4488	28,2169	28,6808	10	5	5	0,0627004	0,463885	Q03D45	Q03D45	rpsR	30S ribosomal protein S18
29,0453	28,679	28,8294	28,7432	28,8024	28,6418	28,7511	28,7055	28,7549	28,5779		19	19	19	56,4	56,4	56,4	34,758	1,38E+10	285	28,753	28,8198	28,6862	10	5	5	0,0954204	-0,13362	Q035A9	Q035A9	rpoA	DNA-directed RNA polymerase subunit alpha
28,9708	28,9382	28,882	28,8599	28,9022	28,7551	28,704	28,6656	28,9984	28,6188	+	13	13	13	54,5	54,5	54,5	34,229	1,26E+10	189	28,8295	28,9106	28,7484	10	5	5	0,0473403	-0,162207	Q039I0	Q039I0	pfkA	ATP-dependent 6-phosphofructokinase
26,7325	28,2299	28,2003	28,3065	28,0961	28,6606	28,8247	28,609	29,1231	28,6802	+	11	11	11	40,6	40,6	40,6	14,019	5,47E+09	154	28,3463	27,913	28,7795	10	5	5	0,023797	0,866491	Q035U2	Q035U2	LSEI_2286	DUF4430 domain-containing protein
28,2364	28,9479	28,8356	28,7942	28,7362	28,7399	28,8945	28,8013	28,9289	28,8507		14	14	14	73,9	73,9	73,9	13,376	1,20E+10	238	28,7766	28,7101	28,8431	10	5	5	0,328698	0,132971	Q038K1	Q038K1	rplS	50S ribosomal protein L19
28,4834	28,383	28,3926	28,4466	28,8017	29,2195	29,0233	28,9487	28,0304	29,08		6	6	6	44,7	44,7	44,7	11,35	2,72E+09	61	28,6809	28,5014	28,8604	10	5	5	0,150572	0,35895	Q038F2	Q038F2	rplU	50S ribosomal protein L21
28,4817	28,6333	28,6388	28,6612	28,6773	28,4751	28,8406	28,8166	29,034	29,1645		11	10	10	37,6	34,8	34,8	42,596	2,61E+09	50	28,7423	28,6185	28,8662	10	5	5	0,0768811	0,247674	Q03AV0	Q03AV0	LSEI_0865	Aminotransferase
28,1178	29,0077	28,8206	28,7595	28,84	28,7694	28,888	28,7696	29,221	28,8614		7	7	7	45,9	45,9	45,9	13,035	8,07E+09	92	28,8055	28,7091	28,9019	10	5	5	0,301672	0,192756	Q034Z3	Q034Z3	rplN	50S ribosomal protein L14
29,1463	29,0819	29,2376	29,4041	29,2732	29,006	28,7978	28,939	29,0223	29,0546	+	15	15	15	65,2	65,2	65,2	33,782	1,03E+10	135	29,0963	29,2286	28,9639	10	5	5	0,00610966	-0,264716	Q039D0	Q039D0	LSEI_1414	Pyrophosphate phospho-hydrolase
28,7426	28,1622	28,3835	28,4611	28,4172	29,1086	29,029	28,9497	29,1232	28,7187	+	8	8	8	53,4	53,4	53,4	11,23	1,00E+10	181	28,7096	28,4333	28,9858	10	5	5	0,00162343	0,552518	Q034Z4	Q034Z4	rplX	50S ribosomal protein L24
28,9618	28,9659	28,939	29,0405	28,9662	28,8972	28,9975	29,1022	29,1179	29,0224		18	18	18	60,6	60,6	60,6	34,076	1,58E+10	236	29,0011	28,9747	29,0274	10	5	5	0,258318	0,0527489	Q03B79	Q03B79	LSEI_0703	ABC-type uncharacterized transport system, periplasmic component
29,3455	29,2181	29,1939	29,2473	29,177	28,8546	29,0132	29,0837	29,1206	29,1417	+	28	28	28	49,1	49,1	49,1	55,3	1,92E+10	435	29,1396	29,2364	29,0428	10	5	5	0,0119234	-0,193597	Q03A20	Q03A20	atpA	ATP synthase subunit alpha
29,3691	28,9441	28,9083	28,8435	29,1481	29,1024	29,0126	29,1283	28,9001	29,1112		11	11	11	58,8	58,8	58,8	41,152	1,10E+10	117	29,0468	29,0426	29,0509	10	5	5	0,939194	0,00828552	Q036K8	Q036K8	LSEI_2114	3-oxoacyl-[acyl-carrier-protein] synthase 2
29,1405	29,0392	29,0006	28,9753	29,1328	29,0084	29,0589	29,0932	28,9104	29,2644		54	54	53	73,2	73,2	73,2	78,535	4,42E+10	727	29,0624	29,0577	29,0671	10	5	5	0,892672	0,00937271	Q036R9	Q036R9	LSEI_2048	ATP-binding subunit of Clp protease and DnaK/DnaJ chaperones
29,4377	29,4226	29,4091	29,4439	29,4504	29,0596	29,013	29,106	28,9248	29,2489	+	43	43	43	76,9	76,9	76,9	63,409	3,84E+10	592	29,2516	29,4327	29,0705	10	5	5	0,000156269	-0,362267	Q037U1	Q037U1	LSEI_1759	Phosphoenolpyruvate-protein phosphotransferase
29,0791	29,3635	29,3611	29,3834	29,2533	29,0587	29,028	29,0159	29,2006	29,075	+	32	32	32	65	65	65	48,056	3,20E+10	564	29,1818	29,2881	29,0756	10	5	5	0,0121383	-0,212417	Q03AI8	Q03AI8	LSEI_0988	Carbohydrate ABC transporter substrate-binding protein, CUT1 family
29,134	29,4364	29,2833	29,413	29,2979	29,2019	29,1264	28,9868	29,4124	28,9637		14	14	14	63	63	63	15,466	1,88E+10	246	29,2256	29,3129	29,1382	10	5	5	0,111791	-0,174691	Q035A2	Q035A2	rplO	50S ribosomal protein L15
29,2232	29,174	29,2686	29,4136	29,2982	29,0791	29,2347	29,1801	29,2952	29,2617		16	16	16	52,9	52,9	52,9	35,001	9,47E+09	148	29,2428	29,2755	29,2102	10	5	5	0,271598	-0,0653416	Q039T3	Q039T3	mreB	Cell shape-determining protein MreB
27,4244	29,3084	29,0954	28,2632	28,9613	29,717	29,1524	28,9124	29,2627	29,2266		2	2	2	33	33	33	10,521	5,63E+09	64	28,9324	28,6105	29,2542	10	5	5	0,118769	0,643674	Q038J7	Q038J7	rpsP	30S ribosomal protein S16
28,5984	28,959	28,8959	28,8131	28,9086	29,3145	29,2033	29,5291	29,3958	29,1067	+	4	4	4	89,4	89,4	89,4	7,3549	7,61E+09	127	29,0724	28,835	29,3099	10	5	5	0,00121904	0,474861	Q03A67	Q03A67	LSEI_1114	Cold-shock DNA-binding protein family
28,0485	28,2124	28,3279	28,284	28,294	29,2069	29,4317	29,4376	29,1696	29,3357	+	22	22	22	59,1	59,1	59,1	46,996	1,71E+10	334	28,7748	28,2334	29,3163	10	5	5	5,04E-07	1,08294	Q038R1	Q038R1	LSEI_1536	N-acetylmuramoyl-L-alanine amidase
29,4442	29,4112	29,0789	29,1448	29,3452	29,5511	29,3424	29,2407	29,2946	29,2834		12	12	12	80,8	80,8	80,8	17,535	1,83E+10	351	29,3137	29,2849	29,3424	10	5	5	0,546076	0,0575447	Q035A0	Q035A0	rpsE	30S ribosomal protein S5
29,166	29,7596	29,6857	29,6372	29,5477	29,3709	29,3822	29,3473	29,5128	29,509		18	18	18	28,3	28,3	28,3	66,416	2,14E+10	267	29,4918	29,5592	29,4244	10	5	5	0,25566	-0,134813	Q038J0	Q038J0	LSEI_1608	ABC-type oligopeptide transport system, periplasmic component
29,384	29,6216	29,4384	29,4546	29,3523	29,39	29,4251	29,4559	29,6877	29,198		7	7	7	58,9	58,9	58,9	13,772	1,07E+10	129	29,4408	29,4502	29,4313	10	5	5	0,841167	-0,0188622	Q035A8	Q035A8	rpsK	30S ribosomal protein S11
25,2884	27,6665	27,7156	28,3141	27,4592	29,7458	29,2136	29,2963	29,1937	29,836	+	4	4	4	38,3	38,3	38,3	5,431	2,66E+09	43	28,3729	27,2888	29,4571	10	5	5	0,00378537	2,1683	Q03B97	Q03B97	LSEI_0685	Uncharacterized protein
29,1207	29,2252	29,2413	29,3177	29,3738	29,6829	29,395	29,2803	29,5272	29,4597	+	15	15	15	78,6	78,6	78,6	18,177	1,49E+10	330	29,3624	29,2557	29,469	10	5	5	0,0282855	0,2133	Q035V5	Q035V5	rplJ	50S ribosomal protein L10
29,738	29,6881	29,4915	29,589	29,2862	29,4395	29,3649	29,4637	29,7852	29,4478		11	11	11	53,3	53,3	53,3	22,627	1,53E+10	159	29,5294	29,5586	29,5002	10	5	5	0,605557	-0,0583664	Q034Y3	Q034Y3	rplC	50S ribosomal protein L3
27,3356	28,7357	28,6963	28,8096	28,5735	29,2615	29,5148	29,0482	30,1045	29,667	+	4	4	4	61,2	61,2	61,2	5,8618	4,91E+09	136	28,9747	28,4301	29,5192	10	5	5	0,0108744	1,08905	Q037L6	Q037L6	rpmG1	50S ribosomal protein L3
28,1459	29,5237	29,645	30,058	29,822	29,6575	29,5632	29,1688	29,6845	29,6038		13	13	13	86,1	86,1	86,1	12,518	1,62E+10	249	29,4873	29,4389	29,5356	10	5	5	0,788587	0,0966255	Q035V6	Q035V6	rplL	50S ribosomal protein L7/L12
29,765	29,729	29,6924	29,7601	29,6544	29,5899	29,4588	29,5747	29,8011	29,4915		10	10	10	65,3	65,3	65,3	19,334	2,12E+10	284	29,6517	29,7202	29,5832	10	5	5	0,062513	-0,137001	Q034Z8	Q034Z8	rplF	50S ribosomal protein L6
30,096	29,8677	29,7048	29,6173	29,6599	29,586	29,5376	29,6505	29,5833	29,571		30	30	30	63,5	63,5	63,5	49,322	2,54E+10	414	29,6874	29,7891	29,5857	10	5	5	0,052742	-0,203441	Q03A55	Q03A55	pgi	Glucose-6-phosphate isomerase
29,7757	29,4498	29,6106	29,8073	29,9242	29,7037	29,3842	29,5834	29,2159	30,1389		57	57	57	80,6	80,6	80,6	67,563	3,98E+10	767	29,6594	29,7135	29,6052	10	5	5	0,559457	-0,108303	Q038N3	Q038N3	dnaK	Chaperone protein DnaK
29,8379	29,6339	29,4215	29,5722	29,517	29,603	29,5397	29,5801	29,8178	29,593		5	5	5	43,7	43,7	43,7	10,202	6,97E+09	63	29,6116	29,5965	29,6267	10	5	5	0,732278	0,0301804	Q034Z2	Q034Z2	rpsQ	30S ribosomal protein S17
30,6519	29,6277	29,5699	29,5967	29,6131	29,6	29,6577	29,7024	29,7223	29,7098		20	20	20	68,7	68,7	68,7	29,546	3,78E+10	537	29,7451	29,8119	29,6784	10	5	5	0,545539	-0,13344	Q038L2	Q038L2	rpsB	30S ribosomal protein S2
28,4731	27,8708	27,9827	28,06	28,2976	29,7664	29,8989	29,7872	29,403	29,9613	+	15	15	15	41,5	41,5	41,5	41,403	9,32E+09	168	28,9501	28,1369	29,7634	10	5	5	3,80E-06	1,6265	Q033W0	Q033W0	LSEI_2798	Uncharacterized protein
28,3975	29,7859	29,5731	29,7532	29,6385	29,7466	29,8806	29,6566	30,0989	29,5831		7	7	7	50	50	50	6,9481	8,98E+09	167	29,6114	29,4296	29,7931	10	5	5	0,224785	0,363511	Q038S6	Q038S6	rpsU	30S ribosomal protein S21
30,0933	29,8337	29,6457	29,4832	29,5552	29,8761	29,8421	29,8532	29,4883	29,9313		40	40	40	72,4	72,4	72,4	76,819	4,31E+10	690	29,7602	29,7222	29,7982	10	5	5	0,589615	0,0759838	Q034X8	Q034X8	fusA	Elongation factor G
29,8766	29,1564	29,4569	29,5959	29,7836	29,9762	29,8436	29,9566	29,2736	30,2855		32	32	32	64,7	64,7	64,7	49,378	1,58E+10	264	29,7205	29,5739	29,8671	10	5	5	0,197788	0,293233	Q039K4	Q039K4	tig	Trigger factor
28,5354	29,6733	29,5375	29,8247	29,7617	29,7955	30,0707	29,4721	30,3209	29,7751		7	7	7	54,2	54,2	54,2	10,382	6,17E+09	386	29,6767	29,4665	29,8869	10	5	5	0,16893	0,420339	Q038F4	Q038F4	rpmA	50S ribosomal protein L27
29,7874	29,8628	30,0152	30,1031	29,9987	29,8812	29,738	29,8545	30,0943	29,9066		19	19	19	69	69	69	24,48	3,59E+10	573	29,9242	29,9534	29,8949	10	5	5	0,489274	-0,0585232	Q035U9	Q035U9	rplA	50S ribosomal protein L1
28,7712	30,2881	30,0651	30,142	30,0161	29,8477	30,0373	29,6715	30,2138	29,8973		7	7	7	69,7	69,7	69,7	10,3	1,22E+10	140	29,895	29,8565	29,9335	10	5	5	0,797191	0,0770386	Q039L3	Q039L3	rpsO	30S ribosomal protein S15
26,9372	29,7161	30,3906	30,4941	30,1061	30,0475	29,8161	29,8382	30,1972	29,8702		4	4	4	19,2	19,2	19,2	14,273	7,96E+09	96	29,7413	29,5288	29,9538	10	5	5	0,541088	0,425029	Q035C4	Q035C4	rpsI	30S ribosomal protein S9
29,2691	30,0188	29,8995	29,9669	29,6982	29,9184	29,8194	29,964	30,2575	29,9051		4	4	4	31,5	31,5	31,5	14,229	1,07E+10	164	29,8717	29,7705	29,9729	10	5	5	0,230182	0,202398	Q035B0	Q035B0	rplQ	50S ribosomal protein L17
30,0457	30,0132	29,996	30,2186	30,0889	30,0453	29,9699	29,9727	30,0198	29,9417		20	20	20	67,7	67,7	67,7	24,903	3,00E+10	392	30,0312	30,0725	29,9899	10	5	5	0,0971677	-0,0825996	Q034Y9	Q034Y9	rpsC	30S ribosomal protein S3
27,9525	29,3767	29,0228	29,3041	29,1986	30,0085	30,2372	29,8096	29,6126	30,3483	+	3	3	3	50,8	50,8	50,8	6,7489	6,89E+09	62	29,4871	28,971	30,0032	10	5	5	0,00797668	1,03229	Q035A1	Q035A1	rpmD	50S ribosomal protein L30
30,5238	30,0131	30,0794	30,2007	30,2969	30,0897	30,0058	30,1148	29,9313	30,2034		30	30	30	69,9	69,9	69,9	47,677	5,03E+10	541	30,1459	30,2228	30,069	10	5	5	0,166997	-0,153777	Q039G5	Q039G5	LSEI_1379	SSU ribosomal protein S1P
29,6853	30,285	30,1071	30,1548	30,1516	29,9952	30,2002	30,036	30,4182	30,0321		8	8	8	49,3	49,3	49,3	15,973	1,28E+10	195	30,1066	30,0768	30,1364	10	5	5	0,656878	0,0595734	Q036L0	Q036L0	LSEI_2112	(3R)-hydroxymyristoyl-[acyl-carrier-protein] dehydratase
29,5271	30,4298	30,3616	30,5444	30,3137	30,1047	30,2022	29,9503	30,4736	30,0137		20	20	20	82,1	82,1	82,1	17,923	4,26E+10	469	30,1921	30,2353	30,1489	10	5	5	0,681818	-0,086385	Q034X7	Q034X7	rpsG	30S ribosomal protein S7
28,8771	30,3466	30,3161	30,2617	30,1389	30,0603	30,1898	30,059	30,434	30,1907		5	5	5	61,4	61,4	61,4	9,5477	1,36E+10	82	30,0874	29,9881	30,1868	10	5	5	0,510104	0,198687	Q034T0	Q034T0	rpmE2	50S ribosomal protein L31 type B
29,7604	30,3638	30,3991	30,3669	30,3571	30,2135	30,0536	30,1403	30,5645	30,0986		17	17	17	90,5	90,5	90,5	16,581	3,90E+10	401	30,2318	30,2495	30,2141	10	5	5	0,822898	-0,0353554	Q035C3	Q035C3	rplM	50S ribosomal protein L13
30,9286	30,5973	30,4839	30,6368	30,4959	30,1082	30,1911	30,1968	30,4298	30,2868	+	24	24	24	70,5	70,5	70,5	30,336	3,90E+10	465	30,4355	30,6285	30,2426	10	5	5	0,00414034	-0,385961	Q034Y6	Q034Y6	rplB	50S ribosomal protein L2
30,1527	30,2178	29,9534	29,852	30,2596	30,4753	30,214	30,07	30,3106	30,1447		10	10	10	69,2	69,2	69,2	12,729	2,10E+10	217	30,165	30,0871	30,2429	10	5	5	0,178493	0,155821	Q034Y8	Q034Y8	rplV	50S ribosomal protein L22
30,1721	30,4559	30,1801	30,2621	30,3367	30,1499	30,2903	30,0552	30,4241	30,3447		6	6	6	56,5	56,5	56,5	15,173	1,22E+10	214	30,2671	30,2814	30,2528	10	5	5	0,745877	-0,0285606	Q034X6	Q034X6	rpsL	30S ribosomal protein S12
29,208	30,3271	30,3162	30,5354	30,3216	30,4162	30,269	30,0218	30,3946	30,2318		16	16	16	65,3	65,3	65,3	13,491	2,41E+10	353	30,2042	30,1417	30,2667	10	5	5	0,62698	0,124992	Q035A7	Q035A7	rpsM	30S ribosomal protein S13
29,5978	29,6457	29,7101	29,968	29,8801	30,3746	30,4074	30,1561	30,2413	30,1737	+	7	7	7	57,1	57,1	57,1	13,001	7,84E+09	142	30,0155	29,7604	30,2706	10	5	5	0,000385418	0,510246	Q034Z9	Q034Z9	rplR	50S ribosomal protein L18
30,3349	30,3086	30,3433	30,411	30,3911	30,2305	30,3592	30,4009	30,3788	30,1969		20	20	20	54,9	54,9	54,9	32,805	1,78E+10	278	30,3355	30,3578	30,3133	10	5	5	0,357432	-0,0445095	Q035T2	Q035T2	LSEI_2296	Uncharacterized protein
28,4792	28,8742	29,5528	30,019	29,8758	30,4162	30,3143	30,2474	30,1836	30,5844	+	5	5	5	65,8	65,8	65,8	9,0138	1,39E+10	106	29,8547	29,3602	30,3492	10	5	5	0,0116408	0,988972	Q036K4	Q036K4	acpP	Acyl carrier protein
29,3555	30,5845	30,4317	30,6954	30,5318	30,3838	30,558	30,2379	30,7238	29,9675		12	12	12	68,8	68,8	68,8	10,577	2,05E+10	237	30,347	30,3198	30,3742	10	5	5	0,849392	0,0544018	Q034Y7	Q034Y7	rpsS	30S ribosomal protein S19
30,6407	30,6413	30,4164	30,5227	30,4453	30,4259	30,3187	30,3521	30,5831	30,3188		24	24	24	83,3	83,3	83,3	23,306	5,08E+10	605	30,4665	30,5333	30,3997	10	5	5	0,0877999	-0,133559	Q039U3	Q039U3	rpsD	30S ribosomal protein S4
30,046	30,5116	30,4468	30,54	30,5336	30,407	30,3962	30,2771	30,801	30,5192		10	10	10	76	76	76	11,56	2,40E+10	287	30,4478	30,4156	30,4801	10	5	5	0,631337	0,0644932	Q034Y5	Q034Y5	rplW	50S ribosomal protein L2
30,2218	30,5626	30,3688	30,4621	30,3671	30,3598	30,4557	30,4269	30,7656	30,5143		12	12	12	44,9	44,9	44,9	22,486	3,32E+10	372	30,4505	30,3965	30,5045	10	5	5	0,263989	0,107984	Q034Y4	Q034Y4	rplD	50S ribosomal protein L4
30,169	31,0464	31,009	31,1372	30,9607	30,569	30,5307	30,3853	30,8647	30,3633		11	11	11	92,5	92,5	92,5	10,039	2,00E+10	364	30,7035	30,8645	30,5426	10	5	5	0,142397	-0,321862	Q035Y8	Q035Y8	groS	10 kDa chaperonin
30,1177	30,5982	30,6298	30,8173	30,7119	30,4794	30,5112	30,5182	30,8131	30,5533		16	16	16	56,3	56,3	56,3	25,98	5,91E+10	745	30,575	30,575	30,575	10	5	5	0,999807	3,36E-05	Q036I8	Q036I8	gpmA	23-bisphosphoglycerate-dependent phosphoglycerate mutase
29,8705	30,6829	30,4976	30,7287	30,6103	30,8282	30,6549	30,5423	30,7101	30,7436		7	7	7	62,4	62,4	62,4	14,852	1,30E+10	221	30,5869	30,478	30,6958	10	5	5	0,220465	0,217786	Q035U8	Q035U8	rplK	50S ribosomal protein L11
30,743	30,7776	30,8461	30,8839	30,9463	30,656	30,6277	30,7835	30,5792	30,9307		53	53	53	78,6	78,6	78,6	62,806	1,27E+11	1555	30,7774	30,8394	30,7154	10	5	5	0,129032	-0,12398	Q039H9	Q039H9	LSEI_1365	Pyruvate kinase
29,7371	30,8185	30,5914	30,4932	30,5439	30,6807	30,6926	30,6934	30,8506	30,8131		14	14	14	68,9	68,9	68,9	14,774	2,33E+10	312	30,5914	30,4368	30,7461	10	5	5	0,136726	0,309247	Q034Z7	Q034Z7	rpsH	30S ribosomal protein S8
31,2857	30,7165	30,782	30,7742	30,7334	30,8423	30,7289	30,7663	30,6362	30,8022		13	13	13	60	60	60	20,168	3,55E+10	399	30,8068	30,8584	30,7552	10	5	5	0,388432	-0,103176	Q034Z5	Q034Z5	rplE	50S ribosomal protein L5
30,3447	30,6961	30,7382	30,7934	30,8583	30,662	30,6558	30,7762	30,9478	30,7507		16	16	16	84	84	84	31,489	4,94E+10	441	30,7223	30,6861	30,7585	10	5	5	0,506541	0,0723614	Q03BY4	Q03BY4	LSEI_0432	Fructose-bisphosphate aldolase
30,1322	30,1652	30,051	30,1979	29,8004	30,5015	31,015	30,6296	30,8424	31,1812	+	6	6	6	16,2	16,2	16,2	45,176	8,95E+09	53	30,4516	30,0693	30,8339	10	5	5	0,000683265	0,764591	Q03AH6	Q03AH6	LSEI_1000	ABC-type Na+ efflux pump, permease component
31,0392	30,9392	31,0996	31,1985	31,2155	30,9389	30,9641	31,0585	31,1122	31,0863		29	29	19	76,2	76,2	56,6	36,366	7,69E+10	618	31,0652	31,0984	31,032	10	5	5	0,312662	-0,0664028	Q034Q4	Q034Q4	lacD	Tagatose 1,6-diphosphate aldolase
29,6893	31,0682	31,0329	31,1904	30,8876	30,9161	31,1117	30,8747	31,38	31,0479		9	9	9	59,8	59,8	59,8	11,736	3,12E+10	281	30,9199	30,7737	31,0661	10	5	5	0,342081	0,29241	Q034Y2	Q034Y2	rpsJ	30S ribosomal protein S10
31,2828	31,2586	31,4266	31,5081	31,5506	31,3048	31,0226	31,2105	31,1607	31,2777	+	31	31	31	62,6	62,6	62,6	35,53	8,05E+10	1237	31,3003	31,4053	31,1953	10	5	5	0,0259685	-0,210075	Q034V0	Q034V0	ldh	L-lactate dehydrogenase

29,2217	30,2313	30,5266	30,5269	30,6839	31,1826	31,0283	31,212	31,0043	31,5867	+	5	5	5	8,9	8,9	8,9	70,711	1,30E+10	30	30,7204	30,2381	31,2028	10	5	5	0,00945128	0,964653	Q039F8	Q039F8	LSEI_1386	ATPase component of ABC transporter with duplicated ATPase domains
31,3298	31,2525	31,375	31,4227	31,4438	31,1582	31,1553	31,2932	31,3134	31,2831	+	49	49	49	92,9	92,9	92,9	42,237	1,54E+11	1257	31,3027	31,3648	31,2406	10	5	5	0,0342824	-0,124123	Q03AK6	Q03AK6	pgk	Phosphoglycerate kinase
31,4696	31,7041	31,3895	31,281	31,3346	30,9841	31,2816	31,1132	31,25	31,6344		17	17	17	28,6	28,6	28,6	88,463	1,76E+10	81	31,3442	31,4357	31,2526	10	5	5	0,202335	-0,183094	Q038E8	Q038E8	pepX	Xaa-Pro dipeptidyl-peptidase
30,9994	30,9268	31,0692	31,2379	31,4525	31,3048	31,1054	31,2693	31,3394	31,4338		20	20	20	80,9	80,9	80,9	26,979	5,47E+10	465	31,2138	31,1372	31,2905	10	5	5	0,195029	0,153378	Q03AK5	Q03AK5	tpiA	Triosephosphate isomerase
29,9588	29,5761	29,7512	29,7024	29,9007	32,2051	31,8042	31,8902	31,006	32,1933	+	9	9	9	37	37	37	35,039	1,69E+10	228	30,7988	29,7778	31,8198	10	5	5	1,99E-05	2,04195	Q036A1	Q036A1	LSEI_2226	Cell wall-associated hydrolase
30,6318	31,4665	31,5274	31,75	31,5438	31,9485	31,9195	31,6688	32,1227	31,8557	+	5	5	5	42	42	42	9,2534	3,99E+10	289	31,6435	31,3839	31,9031	10	5	5	0,0367682	0,519143	Q037U0	Q037U0	LSEI_1760	Phosphocarrier protein HPr
32,0241	31,6267	31,7594	31,8959	32,0064	32,0198	31,8438	32,0093	31,5613	32,1805		58	58	58	92,8	92,8	92,8	57,427	1,56E+11	2253	31,8927	31,8625	31,923	10	5	5	0,652671	0,0604412	Q035Y9	Q035Y9	groL	60 kDa chaperonin
32,286	31,9819	32,0011	32,0045	32,1416	32,1713	31,8789	32,0461	31,9126	32,0911		35	35	35	75,3	75,3	75,3	47,086	1,21E+11	1015	32,0515	32,083	32,02	10	5	5	0,453326	-0,0630043	Q03AK4	Q03AK4	eno	Enolase
30,0565	31,7148	31,8489	31,8697	31,9403	32,3108	32,0939	31,811	32,44	32,2892		17	17	17	85,7	85,7	85,7	9,5259	1,07E+11	951	31,8375	31,486	32,189	10	5	5	0,0981626	0,702929	Q039G3	Q039G3	LSEI_1381	DNA-binding protein HU
32,5991	32,7118	32,6895	32,7714	32,8121	32,6994	32,7458	32,7682	32,8811	32,8702		26	26	26	67,6	67,6	67,6	36,706	2,62E+11	3770	32,7549	32,7168	32,7929	10	5	5	0,173663	0,076152	Q03AK7	Q03AK7	LSEI_0967	Glyceraldehyde-3-phosphate dehydrogenase
32,6151	32,4364	32,5139	32,4797	32,7013	32,8073	32,7479	32,9084	32,7527	32,9966	+	35	35	35	77,8	77,8	77,8	43,573	2,57E+11	1700	32,6959	32,5493	32,8426	10	5	5	0,00258057	0,293305	Q039K9	Q039K9	tuf	Elongation factor Tu
31,8355	32,1269	32,4011	32,7103	32,6099	34,0304	34,2658	33,9465	33,8275	34,3891	+	31	31	31	50	50	50	40,802	2,07E+11	2315	33,2143	32,3367	34,0919	10	5	5	1,57E-05	1,75512	Q03D36	Q03D36	LSEI_0020	Surface antigen
32,2456	32,524	32,7165	32,8598	32,7542	33,9336	34,2303	34,0484	33,9975	34,4001	+	28	28	28	50	50	50	41,107	2,72E+11	1849	33,371	32,62	34,122	10	5	5	4,44E-06	1,50196	Q036T8	Q036T8	LSEI_2029	Surface antigen
31,4823	32,1764	32,5028	32,4908	32,7443	34,3284	34,2986	34,245	33,4546	34,6297	+	18	18	18	22,2	22,2	22,2	49,408	2,05E+11	2027	33,2353	32,2793	34,1913	10	5	5	0,000186	1,91195	Q03CD1	Q03CD1	LSEI_0281	Cell wall-associated hydrolase
25,0276	NaN	NaN	NaN	NaN	26,041	27,3975	26,5405	NaN	25,4519		2	2	2	18,8	18,8	18,8	13,87	2,45E+08	3	26,0917	25,0276	26,3577	5	1	4	1	1,33011	Q039M0;Q03	Q039M0;Q036F	LSEI_1321	Transposase
22,9318	NaN	NaN	NaN	23,3786	23,8457	23,8986	23,9399	24,0885	NaN	+	2	2	2	4	4	4	49,774	6,39E+07	3	23,6805	23,1552	23,9431	6	2	4	0,00748966	0,787937	Q03AX2	Q03AX2	LSEI_0814	Glycerophosphoryl diester phosphodiesterase
NaN	NaN	NaN	23,0419	23,0526	24,4768	23,8097	24,0022	NaN	25,4653		3	3	3	16,3	16,3	16,3	29,146	9,24E+07	9	23,9748	23,0473	24,4385	6	2	4	0,0662193	1,39124	Q033W3	Q033W3	LSEI_2795	Uncharacterized protein
23,3299	NaN	NaN	23,3314	23,023	23,8268	NaN	23,6875	23,6102	23,8437	+	5	5	5	24,1	24,1	24,1	33,87	1,15E+08	7	23,5218	23,2281	23,7421	7	3	4	0,00517269	0,513942	Q038Q5	Q038Q5	prmA	Ribosomal protein L11 methyltransferase
23,4092	NaN	23,3038	22,852	23,4541	23,768	NaN	23,7307	23,8586	23,7236	+	3	3	3	24,7	24,7	24,7	22,216	8,97E+07	8	23,5125	23,2548	23,7702	8	4	4	0,0107543	0,515407	Q039K2	Q039K2	engB	Probable GTP-binding protein EngB
23,6629	24,0256	24,0403	NaN	23,8776	23,779	23,9795	23,7808	NaN	23,8703		3	3	3	8,2	8,2	8,2	47,849	1,11E+08	9	23,877	23,9016	23,8524	8	4	4	0,638986	-0,0492206	Q039E2	Q039E2	trmFO	Methylenetetrahydrofolate--tRNA-(uracil-5-)-methyltransferase TrmFO
NaN	24,3554	24,0942	24,3225	24,0772	24,2811	24,3126	NaN	24,0513	24,0496		3	3	3	21,8	21,8	21,8	10,282	1,01E+08	8	24,193	24,2123	24,1737	8	4	4	0,719062	-0,0386567	Q035W8	Q035W8	LSEI_2260	Uncharacterized protein
NaN	25,2377	24,5532	24,6324	24,4183	24,1579	24,1701	24,1087	24,2707	NaN	+	2	2	2	18	18	18	12,857	1,44E+08	5	24,4436	24,7104	24,1769	8	4	4	0,0275616	-0,533548	Q03BZ7	Q03BZ7	LSEI_0419	Phosphoenolpyruvate--glycerone phosphotransferase
NaN	23,6117	23,4391	23,8996	23,8475	23,7836	23,9492	NaN	24,8523	24,4153		3	3	3	37,2	37,2	37,2	10,121	1,09E+08	10	23,9748	23,6995	24,2501	8	4	4	0,0819654	0,550658	Q03B00	Q03B00	LSEI_0786	UPF0297 protein LSEI_0786
24,9235	NaN	24,4443	24,5413	24,2555	24,094	24,396	24,315	24,5739	NaN		2	2	2	5,3	5,3	5,3	34,049	1,93E+08	16	24,4429	24,5412	24,3447	8	4	4	0,297633	-0,196421	Q03B78	Q03B78	LSEI_0704	ABC-type uncharacterized transport system, permease component
23,7512	24,2382	24,3825	NaN	24,3997	24,5436	24,5397	NaN	25,088	24,8721	+	4	4	4	24	24	24	22,709	1,64E+08	17	24,4769	24,1929	24,7609	8	4	4	0,0308907	0,567953	Q036U0	Q036U0	LSEI_2027	Guanylate kinase
22,5154	22,7803	22,6685	22,274	22,4256	22,4655	22,3284	22,4604	22,6658	NaN		3	3	3	11,2	11,2	11,2	31,261	5,86E+07	7	22,5093	22,5328	22,48	9	5	4	0,668422	-0,052735	Q037I4	Q037I4	LSEI_1879	Alpha/beta hydrolase superfamily protein
22,6661	23,0069	22,8652	22,6174	22,6475	22,6553	22,9632	22,6214	22,681	NaN		4	4	4	8,2	8,2	8,2	65,215	1,01E+08	7	22,7471	22,7606	22,7302	9	5	4	0,790226	-0,0303873	Q038K5	Q038K5	LSEI_1593	ABC-type multidrug transport system, ATPase and permease component
23,3624	23,5009	23,4659	23,171	23,2764	22,9329	22,9037	23,0483	22,8532	NaN	+	5	5	5	7,2	7,2	7,2	101,39	1,40E+08	15	23,1683	23,3553	22,9345	9	5	4	0,000999452	-0,420841	Q03AG4	Q03AG4	LSEI_1012	Cation transport ATPase
23,4444	23,3395	23,1595	23,3336	23,0984	22,9318	22,8834	22,9464	22,9935	NaN	+	8	8	8	19,9	19,9	19,9	47,667	1,48E+08	14	23,1256	23,2751	22,9388	9	5	4	0,00280918	-0,336294	Q03AA2	Q03AA2	LSEI_1076	Superfamily II DNA and RNA helicase
22,9415	22,9195	22,9452	22,9828	22,981	22,8931	23,0318	22,9948	22,9149	NaN		3	3	3	4,8	4,8	4,8	82,438	8,30E+07	7	22,9561	22,954	22,9586	9	5	4	0,889195	0,0046134	Q038P0	Q038P0	LSEI_1558	Single-stranded-DNA-specific exonuclease RecJ
22,7692	23,2905	23,1133	23,0725	23,0074	NaN	22,9574	23,0622	23,2045	22,8966		2	2	2	10,1	10,1	10,1	31,653	6,79E+07	10	23,0415	23,0506	23,0302	9	5	4	0,861332	-0,0204227	Q037R2	Q037R2	LSEI_1789	Sugar phosphate isomerase/epimerase
23,6876	24,7528	24,4984	24,4719	24,293	23,6991	22,6783	23,0125	23,1548	NaN	+	3	3	3	16,2	16,2	16,2	25,127	1,63E+08	16	23,8054	24,3407	23,1362	9	5	4	0,0032815	-1,20456	Q033I5	Q033I5	LSEI_A20	Site-specific recombinase, DNA invertase Pin related protein
22,8705	23,5243	23,2236	23,2539	22,9289	22,8897	23,0218	23,3979	23,3709	NaN		4	4	4	16,8	16,8	16,8	31,946	1,19E+08	13	23,1646	23,1602	23,1701	9	5	4	0,95664	0,00985374	Q03AY2	Q03AY2	LSEI_0804	Small-conductance mechanosensitive channel
23,069	23,457	23,3629	23,2472	23,2067	23,3225	23,3915	23,3635	23,5289	NaN		3	3	3	12,3	12,3	12,3	35,717	1,23E+08	11	23,3277	23,2686	23,4016	9	5	4	0,161781	0,133039	Q039M2	Q039M2	LSEI_1319	Endopeptidase La
23,0178	23,368	23,4819	23,3988	23,5565	23,3476	23,3321	23,3009	23,6631	NaN		4	4	4	8	8	8	50,132	9,81E+07	12	23,3852	23,3646	23,4109	9	5	4	0,72969	0,0463135	Q037Z4	Q037Z4	LSEI_1705	Replicative DNA helicase loader DnaB
23,1268	23,2627	23,6172	23,3861	23,528	23,3974	23,3231	23,4238	23,5256	NaN		7	7	7	14,3	14,3	14,3	72,612	1,58E+08	12	23,399	23,3842	23,4175	9	5	4	0,763963	0,0333079	Q035Y2	Q035Y2	LSEI_2246	ATPase component of ABC transporter with duplicated ATPase domains
23,7696	23,6883	23,6957	23,8247	24,1774	23,8267	23,796	23,4051	NaN	22,768		9	9	9	44,8	44,8	44,8	29,132	2,47E+08	15	23,6613	23,8312	23,4489	9	5	4	0,154341	-0,382225	Q039R1	Q039R1	LSEI_1278	Cell division protein, S4-like domain
23,2268	23,3978	23,4285	23,5348	23,5433	23,3314	23,3576	23,5226	23,6516	NaN		4	4	4	10,6	10,6	10,6	51,322	1,42E+08	15	23,4438	23,4263	23,4658	9	5	4	0,682291	0,0395664	Q03CU1	Q03CU1	LSEI_0117	Replicative DNA helicase
23,7292	24,1087	24,0215	23,8125	24,0104	23,4437	23,4409	23,2487	23,7329	NaN	+	5	4	4	14,9	12,9	12,9	48,294	1,91E+08	13	23,7276	23,9365	23,4665	9	5	4	0,0055392	-0,46993	Q036D8	Q036D8	LSEI_2187	Glutathione reductase
23,6343	23,6522	23,6961	23,7445	23,368	23,6339	23,626	23,3321	23,5028	NaN		7	7	7	43,8	43,8	43,8	20,82	2,04E+08	10	23,5767	23,619	23,5237	9	5	4	0,357542	-0,0953362	Q034V1	Q034V1	pth	Peptidyl-tRNA hydrolase
24,0387	24,2733	24,1264	23,9927	24,2034	NaN	23,6551	24,1304	23,8958	22,8198		4	4	4	11,1	11,1	11,1	54,303	1,43E+08	15	23,9039	24,1269	23,6253	9	5	4	0,0923982	-0,501585	Q03BB6	Q03BB6	galT	Galactose-1-phosphate uridylyltransferase
23,3423	24,0623	24,1559	23,989	23,8703	23,4442	23,5395	23,5414	24,0043	NaN		5	5	5	11,4	11,4	11,4	52,005	1,57E+08	18	23,7722	23,884	23,6324	9	5	4	0,241282	-0,251623	Q035Z8	Q035Z8	LSEI_2229	Hemolysins related protein with CBS domains
22,8401	23,5037	23,2421	23,7007	23,0371	23,3764	23,6569	23,6536	23,9574	NaN		3	3	3	14,1	14,1	14,1	21,032	8,63E+07	9	23,4409	23,2647	23,6611	9	5	4	0,0934579	0,396311	Q03B18	Q03B18	ruvA	Holliday junction ATP-dependent DNA helicase RuvA
24,0055	24,3055	24,0082	24,0935	23,6523	23,4785	23,9824	23,4239	23,9144	NaN		8	8	8	17,8	17,8	17,8	51,883	2,37E+08	24	23,8738	24,013	23,6998	9	5	4	0,115651	-0,313159	Q037K8	Q037K8	LSEI_1855	Pyruvate/oxaloacetate carboxyltransferase
24,2206	24,3583	23,7151	23,3628	23,2949	23,5796	23,8189	23,6549	23,8034	NaN		3	3	3	33,6	33,6	33,6	14,593	1,06E+08	7	23,7565	23,7903	23,7142	9	5	4	0,770688	-0,0761403	Q039H5	Q039H5	LSEI_1369	RibT protein, riboflavin biosynthesis acetyltransferase (GNAT) family
24,1346	24,5139	24,2627	23,8109	23,9952	23,8006	23,9054	NaN	23,813	23,6659	+	3	3	3	18,1	18,1	18,1	23,979	1,58E+08	5	23,9891	24,1434	23,7962	9	5	4	0,0443219	-0,347234	Q039Y7	Q039Y7	LSEI_1199	L-serine deaminase
23,7645	23,7271	23,7396	23,7728	23,7472	23,6049	23,7352	23,9572	23,9039	NaN		4	4	4	13,8	13,8	13,8	42,867	2,17E+08	19	23,7725	23,7503	23,8003	9	5	4	0,505589	0,0500424	Q033L5	Q033L5	LSEI_2899	Metal-dependent amidase/aminoacylase/carboxypeptidase
23,4664	23,6683	23,7783	23,7883	23,6509	23,8465	23,5393	NaN	23,9907	23,8836		4	4	4	22,6	22,6	22,6	29,838	1,58E+08	8	23,7347	23,6704	23,815	9	5	4	0,220858	0,144621	Q034K8	Q034K8	LSEI_2645	Predicted hydrolase of the HAD superfamily
22,913	23,5792	23,978	24,0244	23,871	23,6032	23,8182	23,7988	24,1639	NaN		3	3	3	11,2	11,2	11,2	36,521	1,26E+08	13	23,75	23,6731	23,846	9	5	4	0,518199	0,17291	Q038A6	Q038A6	LSEI_1693	Lactoylglutathione lyase related lyase
24,517	23,9551	23,8329	23,8919	23,9924	23,7259	23,7298	23,9026	24,0281	NaN		5	5	5	17,4	17,4	17,4	27,286	1,71E+08	18	23,9529	24,0379	23,8466	9	5	4	0,252624	-0,191253	Q039H2	Q039H2	LSEI_1372	Pseudouridine synthase
23,8347	23,8072	23,9258	24,1836	23,6837	23,6511	23,7687	23,8341	24,1548	NaN		6	6	6	29,5	29,5	29,5	33,468	2,92E+08	24	23,8715	23,887	23,8522	9	5	4	0,802577	-0,0347944	Q03B76	Q03B76	LSEI_0706	NADPH:quinone reductase related Zn-dependent oxidoreductase
23,0307	24,1737	24,1508	24,2199	23,8076	23,9126	24	23,6659	24,0755	NaN		3	3	3	22,2	22,2	22,2	14,862	1,52E+08	14	23,893	23,8766	23,9135	9	5	4	0,893259	0,0369445	Q038F8	Q038F8	nusB	Transcription antitermination protein NusB
24,015	24,2497	24,1884	23,9575	24,0223	23,9765	23,9424	23,8191	24,148	NaN		6	6	6	27,4	27,4	27,4	24,808	2,31E+08	18	24,0354	24,0866	23,9715	9	5	4	0,228295	-0,115063	Q035P2	Q035P2	gpmA	23-bisphosphoglycerate-dependent phosphoglycerate mutase
23,5869	23,6171	23,8668	23,4435	23,7087	23,8856	NaN	23,9835	24,1178	23,9046	+	4	4	4	23,8	23,8	23,8	25,842	1,53E+08	7	23,7905	23,6446	23,9729	9	5	4	0,00908024	0,328258	Q03B39	Q03B39	LSEI_0747	Carbonyl reductase
24,1831	24,2477	24,4405	24,3065	24,7401	24,1092	23,6388	24,0444	NaN	24,1987	+	5	5	5	23,5	23,5	23,5	29,291	1,96E+08	9	24,2121	24,3836	23,9978	9	5	4	0,0426996	-0,385791	Q033Y4	Q033Y4	murQ	N-acetylmuramic acid 6-phosphate etherase
24,1917	24,1847	24,095	23,9856	23,8955	23,9887	23,9089	23,8809	24,2526	NaN		8	8	8	26	26	26	38,262	2,01E+08	18	24,0426	24,0705	24,0078	9	5	4	0,546317	-0,0627304	Q03A07	Q03A07	metN	Methionine import ATP-binding protein MetN
23,8672	24,0039	24,002	23,943	23,8569	24,2641	23,6857	24,0561	24,0998	NaN		7	7	7	23,1	23,1	23,1	42,669	2,09E+08	20	23,9754	23,9346	24,0264	9	5	4	0,443363	0,091834	Q038H1	Q038H1	coaBC	Coenzyme A biosynthesis bifunctional protein CoaBC
23,5529	24,3281	24,1958	24,2252	24,2891	24,2204	24,4048	24,1987	NaN	23,3012		3	3	3	15,1	15,1	15,1	17,478	1,68E+08	12	24,0796	24,1182	24,0313	9	5	4	0,758213	-0,0869208	Q038S9	Q038S9	ybeY	Endoribonuclease YbeY
23,7435	24,1993	24,0269	23,9912	23,6982	23,9911	23,7572	23,9176	24,4839	NaN		5	5	5	36,8	36,8	36,8	24,236	2,17E+08	17	23,9787	23,9318	24,0374	9	5	4	0,561934	0,105646	Q038D0	Q038D0	udk	Uridine kinase
25,5649	24,9704	24,9154	24,7567	24,7779	24,0933	23,958	24,0415	NaN	24,3871	+	5	5	5	17,3	17,3	17,3	44,101	3,02E+08	19	24,6072	24,997	24,12	9	5	4	0,00218985	-0,877063	Q03A47	Q03A47	LSEI_1134	Glycosyltransferase
23,9523	24,1645	24,3115	24,2777	24,547	24,1107	24,0215	24,0016	24,4035	NaN		5	5	5	23,2	23,2	23,2	32,518	2,84E+08	26	24,1989	24,2506	24,1343	9	5	4	0,424138	-0,116282	Q038X3	Q038X3	LSEI_1471	Fhu operon transcription regulator
23,0264	23,3467	23,4269	23,4967	23,5871	23,6989	24,193	24,4694	NaN	24,3075	+	2	2	2	4,8	4,8	4,8	45,446	1,01E+08	15	23,7281	23,3768	24,1672	9	5	4	0,00339189	0,790455	Q034G7	Q034G7	LSEI_2687	Lyase_8_N domain-containing protein
23,4515	23,9316	24,0043	23,8471	23,9432	24,2829	23,9914	24,1643	24,3494	NaN	+	6	6	6	44,9	44,9	44,9	22,777	2,57E+08	16	23,9962	23,8355	24,197	9	5	4	0,0289557	0,36145	Q03BE1	Q03BE1	LSEI_0640	Predicted nucleoside-diphosphate-sugar epimerase
24,9185	24,7556	24,6907	24,6485	24,4147	23,9939	24,2867	24,346	NaN	24,186	+	11	11	11	32,4	32,4	32,4	53,536	5,07E+08	27	24,4712	24,6856	24,2031	9	5	4	0,00404407	-0,482447	Q03A01	Q03A01	LSEI_1184	Iron-regulated ABC transporter membrane component SufB
26,0285	24,4314	24,6857	24,6355	24,2573	24,323	24,3022	24,26	23,9481	NaN		6	6	6	26,8	26,8	26,8	36,537	3,08E+08	13	24,5413	24,8077	24,2083	9	5	4	0,144528	-0,599362	Q036U4	Q036U4	LSEI_2023	Uncharacterized protein
23,9208	24,1911	24,0701	24,1415	24,0235	23,8839	24,1933	24,1845	24,6575	NaN		8	8	8	36,6	36,6	36,6	26,23	3,36E+08	26	24,1407	24,0694	24,2298	9	5	4	0,32044	0,160413	Q03CJ9	Q03CJ9	rsmG	Ribosomal RNA small subunit methyltransferase G
24,2176	24,2341	24,0384	24,5075	24,1344	24,0425	24,3165	24,2801	24,3329	NaN		6	6	6	16,6	16,6	16,6	51,724	2,38E+08	17	24,2338	24,2264	24,243	9	5	4	0,881011	0,0166087	Q03A94	Q03A94	LSEI_1085	Uncharacterized protein
24,5344	24,7618	24,3659	24,3139	24,5902	24,3582	24,0576	24,1557	24,5092	NaN		7	7	7	32,7	32,7	32,7	28,223	3,18E+08	16	24,4052	24,5132	24,2702	9	5	4	0,0982286	-0,243062	Q039I9	Q039I9	LSEI_1354	Short-chain dehydrogenase of various substrate specificities
24,6695	24,8848	24,4858	24,3258	24,5054	23,8761	24,4227	24,0012	24,8302	NaN		7	7	7	37,5	37,5	37,5	24,297	2,24E+08	16	24,4446	24,5743	24,2825	9	5	4	0,223885	-0,291751	Q038K9	Q038K9	LSEI_1589	1-acyl-sn-glycerol-3-phosphate acyltransferase
24,0535	24,4923	24,1455	24,107	24,1973	24,0304	24,5147	24,2873	24,6681	NaN		4	4	4	25,7	25,7	25,7	21,972	1,99E+08	18	24,2773	24,1991	24,3751	9	5	4	0,279289	0,175999	Q03CQ3	Q03CQ3	LSEI_0155	N-acetyltransferase domain-containing protein
24,5115	24,6115	24,514	24,4893	24,1705	24,4036	24,6622	24,2573	24,2728	NaN		4	4	4	20,1	20,1	20,1	28,307	2,32E+08	14	24,4325	24,4594	24,399	9	5	4	0,626105	-0,0603743	Q03AN3	Q03AN3	pstB	Phosphate import ATP-binding protein PstB
24,9317	25,0462	24,6859	24,407	24,3268	24,431	24,2078	24,3502	24,6423	NaN		8	8	8	18,9	18,9	18,9	54,28	4,31E+08	26	24,5588	24,6795	24,4078	9	5	4	0,17184	-0,271674	Q036V0	Q036V0	LSEI_2017	Lipopolysaccharide synthesis sugar transferase
24,2872	24,539	24,6168	24,5787	24,2289	24,2376	24,6	24,2805	24,5314	NaN		4	4	4	25,3	25,3	25,3	27,729	2,06E+08	11	24,4333	24,4501	24,4124	9	5	4	0,762604	-0,0377741	Q038C4	Q038C4	LSEI_1675	rRNA methylase
25,0326	24,7764	24,4702	23,6635	24,5328	25,18	24,3167	24,9051	23,2591	NaN		5	5	5	19,2	19,2	19,2	42,462	2,71E+08	21	24,4596	24,4951	24,4152	9	5	4	0,865877	-0,0798733	Q038W8	Q038W8	LSEI_1476	Predicted N6-adenine-specific DNA methylase
23,9032	24,3224	24,403	24,2377	24,4602	24,5	24,0693	24,3994	24,7176	NaN		2	2	2	8,7	8,7	8,7	28,942	1,78E+08	12	24,3348	24,2653	24,4216	9	5	4	0,368027	0,156285	Q03BG0	Q03BG0	LSEI_0620	ABC-type multidrug transport system, ATPase component
24,6321	25,0618	25,1142	25,1186	25,0155	24,1586	24,4294	24,4322	24,6707	NaN	+	6	6	6	35,9	35,9	35,9	25,375	3,43E+08	31	24,737	24,9884	24,4227	9	5	4	0,00461637	-0,565711	Q03D49	Q03D49	deoC	Deoxyribose-phosphate aldolase
24,3994	24,2703	24,67	24,368	24,6304	24,6759	24,3327	24,5049	24,2413	NaN		5	5	5	22,4	22,4	22,4	28,105	1,91E+08	10	24,4548	24,4676	24,4387	9	5	4	0,819482	-0,0289245	Q033K8	Q033K8	LSEI_2906	Predicted RNA-binding protein
24,926	24,2478	24,6184	24,3102	24,4889	24,5038	24,4151	24,484	24,7607	NaN		6	6	6	26,4	26,4	26,4	35,331	2,89E+08	11	24,5283	24,5182	24,5409	9	5	4	0,886129	0,0226665	Q036G7	Q036G7	LSEI_2156	Lactate dehydrogenase related 2-hydroxyacid dehydrogenase
24,1929	24,4056	24,5933	24,5819	24,4948	24,6916	24,7841	24,5391	NaN	24,5879		5	5	5	50,4	50,4	50,4	13,449	3,17E+08	25	24,5413	24,4537	24,6507	9	5	4	0,0796636	0,196974	Q03D24	Q03D24	LSEI_0032	Uncharacterized protein
24,5898	24,7467	24,7498	24,8399	24,6305	24,5788	24,5654	NaN	24,8076	24,718		8	8	8	24,9	24,9	24,9	50,613	4,16E+08	28	24,6918	24,7113	24,6674	9	5	4	0,562303	-0,0438901	Q03D55	Q03D55	dnaA	Chromosomal replication initiator protein DnaA
24,3028	24,4835	24,4702	24,458	24,5596	24,6191	24,7175	24,8059	24,9111	NaN	+	5	5	5	18,3	18,3	18,3	35,949	2,54E+08	21	24,592	24,4549	24,7634	9	5	4	0,00376076	0,308556	Q038S8	Q038S8	LSEI_1519	Phosphate starvation-inducible protein PhoH, predicted ATPase
23,6232	24,9518	24,9993	25,0523	24,9366	24,6037	24,7651	24,8754	24,9005	NaN		7	7	7	49,1	49,1	49,1	18,411	2,63E+08	18	24,7453	24,7126	24,7862	9	5	4	0,822175	0,0735723	Q039F6	Q039F6	LSEI_1388	Dihydrofolate reductase
22,6961	24,681	24,5955	24,6955	24,5816	24,7441	24,8966	24,7753	25,2581	NaN		2	2	2	5,9	5,9	5,9	25,581	2,08E+08	17	24,5471	24,25	24,9185	9	5	4	0,183652	0,668564	Q037Y9	Q037Y9	LSEI_1710	Integral membrane protein, interacts with FtsH
25,4949	25,0993	25,2409	24,9904	25,3043	24,9453	24,9925	25,1365	24,8754	NaN		10	10	10	27,6	27,6	27,6	49,46	5,56E+08	36	25,12	25,226	24,9874	9	5	4	0,0659446	-0,23854	Q035Q0	Q035Q0	LSEI_2328	Aminopeptidase
24,2513	24,7011	24,6816	24,5466	24,5369	24,6595	24,9524	25,0206	25,3366	NaN	+	3	3	3	11,9	11,9	11,9	30,526	2,59E+08	20	24,743	24,5435	24,9923	9	5	4	0,0215267	0,448785	Q03AN7	Q03AN7	LSEI_0936	Phosphate-binding protein
25,0891	25,3303	25,1363	25,3237	25,067	24,8911	25,097	25,1543	25,3376	NaN		5	5	5	19,2	19,2	19,2	31,453	3,16E+08	13	25,1585	25,1893	25,12	9	5	4	0,525364	-0,0692816	Q03CJ8	Q03CJ8	LSEI_0211	Effector of nucleoid occlusion Noc
24,9938	25,4752	25,1173	25,2466	25,1589	24,9884	25,09	25,1933	25,4452	NaN		7	7	7	52,9	52,9	52,9	16,819	5,21E+08	30	25,1899	25,1984	25,1792	9	5	4	0,882695	-0,019175	Q03A45	Q03A45	LSEI_1136	Cytosine/adenosine deaminase
25,5661	25,3694	25,2749	25,3836	25,3971	25,3706	25,2603	25,1639	25,637	NaN		7	7	7	28,8	28,8	28,8	29,017	3,85E+08	27	25,3803	25,3982	25,358	9	5	4	0,711133	-0,0402855	Q038U7	Q038U7	LSEI_1499	GTP cyclohydrolase 1 type 2 homolog
25,5557	25,3537	25,3338	25,4608	25,585	25,608	25,1543	25,4149	25,4821	NaN		8	8	8	25,1	25,1	25,1	48,187	4,13E+08	21	25,4387	25,4578	25,4148	9	5	4	0,685842	-0,0430066	Q036W5	Q036W5	hflX	GTPase HflX
25,2429	25,223	25,299	25,2354	25,3643	25,1171	25,2542	25,6534	25,6898	NaN		3	3	3	16	16	16	27,808	3,84E+08	14	25,3421	25,2729	25,4286	9	5	4	0,267636	0,155703	Q03BX9	Q03BX9	LSEI_0438	Predicted glutamine amidotransferase
25,0626	25,0145	24,5905	25,5231	25,2823	25,3604	25,6814	25,2941	25,4691	NaN		5	5	4	23	23	19	33,312	4,39E+08	13	25,2531	25,0946	25,4512	9	5	4	0,103875	0,35666	Q035F9	Q035F9	LSEI_2427	ABC-type metal ion transport system, periplasmic component/surface adhesin
25,8567	26,3069	26,3525	26,3064	26,1012	25,5471	25,3365	25,6956	25,7426	NaN	+	4	4	4	20,4	20,4	20,4	23,188	5,09E+08	22	25,9162	26,1847	25,5805	9	5	4	0,00258976	-0,604283	Q038H8	Q038H8	rpe	Ribulose-phosphate 3-epimerase
24,6645	26,0206	25,3235	26,0782	25,8116	26,5655	25,9564	25,8934	24,6154	NaN		4	4	4	48,6	48,6	48,6	15,98	4,49E+08	11	25,6588	25,5797	25,7577	9	5	4	0,715318	0,177984	Q037X9	Q037X9	LSEI_1720	Diadenosine tetraphosphate (Ap4A) hydrolase related HIT family hydrolase
24,7419	26,1414	25,9362	26,2522	25,9851	26,131	26,1869	25,8276	26,4682	NaN		4	4	4	36,1	36,1	36,1	10,818	5,77E+08	24	25,9634	25,8114	26,1534	9	5	4	0,335185	0,342054	Q038M3	Q038M3	LSEI_1575	Predicted nucleic-acid-binding protein implicated in transcription termination
26,6742	26,7762	26,8611	26,8928	26,8782	27,0204	26,6742	26,5819	24,4497	NaN		5	5	5	20,8	20,8	20,8	31,984	1,20E+09	41	26,5343	26,8165	26,1816	9	5	4	0,258125	-0,634944	Q036K6	Q036K6	LSEI_2116	Malonyl CoA-acyl carrier protein transacylase
25,7987	26,5853	26,6225	26,7717	26,4154	26,3732	26,4477	26,6473	26,7235	NaN		4	4	4	19,7	19,7	19,7	26,909	7,47E+08	22	26,4873	26,4387	26,5479	9	5	4	0,61179	0,10919	Q03CG7	Q03CG7	LSEI_0242	ABC-type Mn/Zn transport system, ATPase component
23,5122	23,7173	NaN	NaN	NaN	23,4768	NaN	23,5588	23,2539	NaN		3	3	3	11,2	11,2	11,2	29,257	7,20E+07	10	23,5038	23,6148	23,4298	5	2	3	0,279013	-0,184913	Q034Q6	Q034Q6	LSEI_2595	Mg-dependent DNase
22,2797	NaN	NaN	NaN	22,9704	NaN	NaN	23,5133	23,6412	23,712	+	2	2	2	11,4	11,4	11,4	22,759	4,78E+07	10	23,2233	22,6251	23,6222	5	2	3	0,0338296	0,997092	Q03BG6	Q03BG6	LSEI_0614	WxL domain-containing protein
NaN	24,7915	NaN	NaN	24,7157	NaN	24,4811	24,3608	24,8935	NaN		2	2	2	16	16	16	16,852	1,31E+08	6	24,6485	24,7536	24,5785	5	2	3	0,465773	-0,175115	Q038G4	Q038G4	argR	Arginine repressor
24,1981	23,8685	NaN	23,4868	NaN	23,4599	23,5564	23,5976	NaN	NaN		3	3	3	12,3	12,3	12,3	36,141	9,66E+07	3	23,6945	23,8511	23,538	6	3	3	0,209342	-0,313152	Q035R4	Q035R4	LSEI_2314	Predicted dehydrogenase related protein
NaN	NaN	25,9579	25,9021	25,7054	NaN	25,8583	NaN	25,9144	26,0032		3	3	3	12,2	12,2	12,2	35,709	3,51E+08	7	25,8902	25,8551	25,9253	6	3	3	0,467372	0,0701459	Q038I8	Q038I8	LSEI_1610	ABC-type dipeptide/oligopeptide/nickel transport system, permease component
22,422	23,0896	23,2233	NaN	23,1237	23,1767	23,034	23,5742	NaN	NaN		2	2	2	6,8	6,8	6,8	39,271	6,52E+07	7	23,0919	22,9646	23,2616	7	4	3	0,297382	0,296961	Q034S1	Q034S1	LSEI_2578	DUF218 domain-containing protein
NaN	23,593	23,9271	24,1409	23,7831	23,5473	23,507	23,308	NaN	NaN	+	4	4	4	34,3	34,3	34,3	14,477	1,10E+08	8	23,6866	23,861	23,4541	7	4	3	0,0423541	-0,406878	Q033V7	Q033V7	LSEI_2801	Fe-S_biosyn domain-containing protein
24,0098	NaN	23,8931	23,5355	23,9481	24,0249	23,9696	NaN	23,8251	NaN		6	6	6	15,4	15,4	15,4	66,161	1,15E+08	10	23,8866	23,8466	23,9399	7	4	3	0,521647	0,0932465	Q03A98	Q03A98	LSEI_1081	DNA helicase
NaN	23,9137	24,245	24,6248	24,4285	24,1379	23,9158	NaN	NaN	24,2792		2	2	2	24,6	24,6	24,6	13,162	1,12E+08	6	24,2207	24,303	24,111	7	4	3	0,380097	-0,192036	Q037X5	Q037X5	LSEI_1724	42 protein LSEI_1724
NaN	24,2749	24,2351	24,308	24,2163	24,3346	24,3502	24,3695	NaN	NaN	+	3	3	3	8,6	8,6	8,6	43,167	1,48E+08	5	24,2984	24,2586	24,3514	7	4	3	0,0153158	0,0928736	Q03AC7	Q03AC7	LSEI_1051	Predicted membrane protein
NaN	23,5784	23,753	23,6081	24,1396	24,5127	24,7402	24,8202	NaN	NaN	+	3	3	3	14,9	14,9	14,9	22,067	1,82E+08	19	24,1646	23,7698	24,691	7	4	3	0,00297424	0,921253	Q03CV1	Q03CV1	LSEI_0107	Phospholipase A2 family enzyme
24,626	25,3885	NaN	25,3019	25,3033	24,9929	25,093	25,4233	NaN	NaN		3	3	3	15,5	15,5	15,5	23,565	2,65E+08	17	25,1613	25,1549	25,1697	7	4	3	0,952564	0,0147878	Q038W2	Q038W2	LSEI_1484	DNA replication protein DnaD
22,123	22,6049	22,3535	22,4562	22,2009	22,2009	22,3634	NaN	22,0857	NaN		3	3	3	8	8	8	34,323	4,70E+07	7	22,2986	22,3477	22,2167	8	5	3	0,351023	-0,13101	Q03B03	Q03B03	LSEI_0783	Uncharacterized protein
22,4161	23,3217	23,2182	23,1621	22,8832	22,9996	22,793	22,7782	NaN	NaN		4	3	3	6	4,9	4,9	83,214	6,24E+07	6	22,9465	23,0003	22,8569	8	5	3	0,545235	-0,143327	Q03AQ7	Q03AQ7	LSEI_0916	DNA translocase FtsK
22,5914	23,4403	23,2679	23,3303	23,1906	23,1329	23,0899	23,0375	NaN	NaN		2	2	2	13,5	13,5	13,5	19,661	7,69E+07	5	23,1351	23,1641	23,0868	8	5	3	0,711687	-0,0773404	Q035U4	Q035U4	LSEI_2284	Corrinoid adenosyltransferase
23,374	24,0545	23,6713	23,6031	23,813	NaN	NaN	23,2197	23,219	22,9046	+	4	4	4	14,8	14,8	14,8	38,403	1,31E+08	10	23,4824	23,7032	23,1144	8	5	3	0,0130488	-0,588759	Q036C8	Q036C8	LSEI_2197	Uncharacterized protein
23,9501	24,9136	24,1324	23,955	23,4294	23,8228	NaN	NaN	22,8272	22,9835		3	3	3	39,9	39,9	39,9	17,078	1,34E+08	9	23,7518	24,0761	23,2112	8	5	3	0,0693494	-0,864929	Q036X5	Q036X5	LSEI_1989	Uncharacterized protein
23,1322	23,6573	22,9806	22,8784	22,6759	23,1283	23,2091	23,3281	NaN	NaN		4	4	4	36,9	36,9	36,9	18,199	1,03E+08	5	23,1237	23,0649	23,2218	8	5	3	0,511301	0,156968	Q035W4	Q035W4	tadA	tRNA-specific adenosine deaminase
23,4685	23,8312	23,6766	23,645	23,5855	23,4011	NaN	23,3741	23,044	NaN	+	3	3	3	9,1	9,1	9,1	33,286	1,12E+08	14	23,5033	23,6414	23,2731	8	5	3	0,0186808	-0,36831	Q033N8	Q033N8	LSEI_2874	NtA domain-containing protein
24,0756	24,0669	23,9442	23,8063	23,9135	23,4403	22,9677	23,4598	NaN	NaN	+	10	10	10	17,7	17,7	17,7	71,223	2,32E+08	30	23,7093	23,9613	23,2892	8	5	3	0,00253849	-0,672067	Q039Z0	Q039Z0	LSEI_1196	Glutathione biosynthesis bifunctional protein
22,767	23,1047	23,1481	23,3056	23,2286	23,3023	23,3056	23,3119	NaN	NaN		4	3	3	19,7	15,7	15,7	33,165	9,14E+07	6	23,1842	23,1108	23,3066	8	5	3	0,163882	0,195794	Q035F3	Q035F3	LSEI_2433	ABC-type metal ion transport system, periplasmic component/surface adhesin
24,6124	23,3052	23,1718	22,9928	23,449	23,8056	23,685	NaN	NaN	22,5606		5	5	5	27,1	27,1	27,1	31,848	1,90E+08	11	23,4478	23,5062	23,3504	8	5	3	0,756126	-0,155871	Q039H7	Q039H7	LSEI_1367	RNA-binding protein, S1-like domain
24,0417	24,9278	23,5581	24,4936	24,3892	23,7811	23,9828	NaN	NaN	22,3826		3	3	3	11,6	11,6	11,6	32,167	1,34E+08	10	23,9446	24,2821	23,3821	8	5	3	0,108945	-0,899945	Q03AG0	Q03AG0	dacA	Diadenylate cyclase
24,6681	24,2063	23,8993	23,5628	24,0231	23,4007	23,5512	23,6442	NaN	NaN		4	4	4	18,8	18,8	18,8	34,79	1,68E+08	16	23,8694	24,0719	23,532	8	5	3	0,0728531	-0,539887	Q033N1	Q033N1	LSEI_2883	Hydrolase of the alpha/beta superfamily
23,2676	23,9037	23,8232	23,8657	23,7908	23,5831	23,6648	23,7837	NaN	NaN		3	3	3	17,2	17,2	17,2	19,051	1,19E+08	7	23,7103	23,7302	23,6772	8	5	3	0,754643	-0,0529957	Q03AY6	Q03AY6	LSEI_0800	Phosphoesterase
23,217	24,4444	24,3791	24,2715	24,332	23,7576	23,8165	23,5291	NaN	NaN		4	4	4	31,5	31,5	31,5	16,276	1,49E+08	15	23,9684	24,1288	23,701	8	5	3	0,220587	-0,427756	Q03BE3	Q03BE3	LSEI_0638	Predicted redox protein, regulator of disulfide bond formation
23,2853	23,9582	23,6931	23,5237	23,4317	23,6681	23,7837	23,712	NaN	NaN		2	2	2	5,3	5,3	5,3	48,263	8,96E+07	9	23,632	23,5784	23,7212	8	5	3	0,395853	0,142832	Q03BX6	Q03BX6	LSEI_0441	Amino acid transporter
23,7426	23,9617	24,0027	24,0315	23,9917	23,849	23,7692	NaN	23,5769	NaN		5	5	5	13,8	13,8	13,8	46,935	1,95E+08	16	23,8657	23,9461	23,7317	8	5	3	0,056816	-0,214353	Q03CC3	Q03CC3	LSEI_0289	Carbohydrate ABC transporter substrate-binding protein, CUT1 family
23,8417	23,9396	23,8547	23,6557	23,8463	NaN	NaN	23,5261	23,9044	23,8167		3	3	3	16,7	16,7	16,7	24,628	1,35E+08	11	23,7981	23,8276	23,749	8	5	3	0,478759	-0,0785615	Q038B1	Q038B1	nadD	Probable nicotinate-nucleotide adenylyltransferase
23,4059	23,8796	23,7395	23,7452	23,5466	23,8877	23,7124	NaN	23,795	NaN		4	4	4	11,5	11,5	11,5	65,715	1,03E+08	7	23,714	23,6634	23,7984	8	5	3	0,293231	0,135008	Q03BF3	Q03BF3	LSEI_0627	ABC-type multidrug transport system, ATPase and permease component
24,2775	23,8441	23,8751	23,947	23,397	23,8006	23,8983	NaN	23,9415	NaN		5	5	5	23,6	23,6	23,6	31,336	1,30E+08	19	23,8726	23,8682	23,8801	8	5	3	0,951986	0,0119371	Q037K5	Q037K5	LSEI_1858	Citrate lyase beta subunit
23,687	24,0498	24,0336	23,7869	23,6723	23,8129	23,8963	24,1101	NaN	NaN		4	4	4	17,1	17,1	17,1	32,248	1,80E+08	18	23,8811	23,8459	23,9398	8	5	3	0,489135	0,0938774	Q034Q8	Q034Q8	rsmA	Ribosomal RNA small subunit methyltransferase A
23,6934	23,6688	23,8048	23,7782	23,8817	24,1144	23,7606	24,0292	NaN	NaN		5	5	5	29,3	29,3	29,3	32,738	1,66E+08	7	23,8414	23,7654	23,9681	8	5	3	0,0728102	0,202703	Q038V5	Q038V5	LSEI_1491	Mevalonate kinase
23,5692	23,914	23,9513	24,1267	23,9327	23,9283	24,0203	23,9796	NaN	NaN		2	2	2	8,2	8,2	8,2	46,176	1,11E+08	9	23,9278	23,8988	23,9761	8	5	3	0,551378	0,0773155	Q039T6	Q039T6	LSEI_1252	Folylpolyglutamate synthase
24,4206	24,4809	24,4241	24,3435	23,949	24,0267	24,0109	24,0346	NaN	NaN		5	5	5	18	18	18	39,783	2,01E+08	15	24,2113	24,3236	24,0241	8	5	3	0,0583495	-0,299482	Q037H6	Q037H6	LSEI_1887	ABC-type dipeptide/oligopeptide/nickel transport system, ATPase component
24,541	24,7126	24,0991	24,4629	24,3727	24,1931	24,0532	23,9529	NaN	NaN	+	7	7	7	31,2	31,2	31,2	30,279	2,25E+08	12	24,2984	24,4377	24,0664	8	5	3	0,0423608	-0,371277	Q033P2	Q033P2	LSEI_2870	3-hydroxyisobutyrate dehydrogenase related beta-hydroxyacid dehydrogenase
24,8488	23,9421	23,5465	23,9596	23,7133	NaN	24,1637	24,0524	23,9927	NaN		5	5	5	22,8	22,8	22,8	25,275	1,95E+08	15	24,0274	24,0021	24,0696	8	5	3	0,830701	0,0675156	Q034K9	Q034K9	LSEI_2644	Phosphoglycerate mutase family protein
24,6178	24,133	24,2857	24,2125	24,4675	24,1414	24,1608	NaN	24,1865	NaN		4	4	4	21,7	21,7	21,7	29,321	2,06E+08	10	24,2756	24,3433	24,1629	8	5	3	0,176991	-0,18038	Q03C18	Q03C18	LSEI_0398	Predicted hydrolase of the HAD superfamily
24,8569	25,1214	24,3977	24,36	24,2719	23,6526	NaN	24,3442	24,877	NaN		3	3	3	7,3	7,3	7,3	33,07	1,99E+08	9	24,4852	24,6016	24,2913	8	5	3	0,396168	-0,310289	Q038I9	Q038I9	LSEI_1609	ABC-type dipeptide/oligopeptide/nickel transport system, permease component

23,9986	24,0601	24,1687	24,2246	24,0523	24,2567	NaN	24,2283	24,4208	NaN	+	3	3	3	13,4	13,4	13,4	35,534	1,60E+08	14	24,1763	24,1009	24,3019	8	5	3	0,0291641	0,201075	Q036F4	Q036F4	LSEI_2171	Geranylgeranyl pyrophosphate synthase
24,9319	25,1511	24,3645	24,5782	24,2153	24,1774	NaN	24,308	24,4576	NaN		3	3	3	9,2	9,2	9,2	57,843	1,95E+08	8	24,523	24,6482	24,3143	8	5	3	0,212847	-0,333856	Q035H1	Q035H1	LSEI_2413	Mn2+ and Fe2+ transporter of the NRAMP family
24,2898	24,5292	25,3836	24,9157	25,28	25,0232	24,7223	NaN	23,2919	NaN		2	2	2	22	22	22	18,371	2,43E+08	14	24,6795	24,8797	24,3458	8	5	3	0,309069	-0,533873	Q03AY8	Q03AY8	LSEI_0798	Predicted hydrocarbon binding protein, V4R domain
24,2812	24,4346	24,2415	24,0054	23,8581	24,3485	24,2075	NaN	24,7482	NaN		4	4	4	23,4	23,4	23,4	26,748	1,64E+08	14	24,2656	24,1642	24,4347	8	5	3	0,185842	0,270542	Q038L0	Q038L0	LSEI_1588	Predicted O-methyltransferase
24,4531	24,7472	24,799	23,4339	24,4543	24,5032	24,4209	24,6259	NaN	NaN		2	2	2	10,2	10,2	10,2	29,142	1,50E+08	10	24,4297	24,3775	24,5167	8	5	3	0,689391	0,139172	Q038K0	Q038K0	trmD	tRNA (guanine-N(1)-)-methyltransferase
25,4034	24,5707	24,7236	24,51	24,3851	NaN	24,5384	24,549	24,5618	NaN		6	6	6	22,1	22,1	22,1	31,781	2,65E+08	12	24,6552	24,7186	24,5497	8	5	3	0,507489	-0,168815	Q039I3	Q039I3	LSEI_1361	Uncharacterized protein
25,1194	25,6135	25,4021	25,7832	25,581	25,1945	25,0975	23,9908	NaN	NaN		3	3	3	25,8	25,8	25,8	17,35	4,23E+08	15	25,2228	25,4998	24,7609	8	5	3	0,0600465	-0,738905	Q03AL1	Q03AL1	LSEI_0962	Transcriptional regulator, MarR family
25,1632	25,8588	25,958	25,2474	24,5692	24,8797	24,8835	NaN	24,8692	NaN		2	2	2	28,2	28,2	28,2	14,812	2,21E+08	14	25,1786	25,3593	24,8775	8	5	3	0,203575	-0,481856	Q03AY1	Q03AY1	LSEI_0805	Methyl-accepting chemotaxis-like protein
24,4271	24,6797	24,7926	24,7657	24,523	25,3964	NaN	24,536	24,927	NaN		5	5	5	19,5	19,5	19,5	33,572	2,54E+08	13	24,7559	24,6376	24,9531	8	5	3	0,173962	0,315516	Q03AA8	Q03AA8	LSEI_1070	SPFH domain, Band 7 family protein
24,6817	25,0304	24,8801	24,9956	25,1601	24,8176	NaN	24,9816	25,7274	NaN		5	5	5	24,9	24,9	24,9	25,623	2,91E+08	18	25,0343	24,9496	25,1755	8	5	3	0,365642	0,225933	Q03D34	Q03D34	LSEI_0022	ABC-type antimicrobial peptide transport system, ATPase component
25,6922	25,0652	25,2847	25,2361	25,4282	25,2221	25,2282	25,199	NaN	NaN		5	5	5	18,2	18,2	18,2	41,443	3,33E+08	19	25,2945	25,3413	25,2164	8	5	3	0,407936	-0,124874	Q03AP3	Q03AP3	prfB	Peptide chain release factor 2
24,3924	24,7979	24,7593	24,6009	24,9933	25,3945	25,1504	25,1536	NaN	NaN	+	2	2	2	26,6	26,6	26,6	11,89	2,11E+08	10	24,9053	24,7088	25,2328	8	5	3	0,0117753	0,524082	Q035X0	Q035X0	LSEI_2258	Uncharacterized protein
24,665	26,7401	26,465	26,7104	26,7727	26,6468	25,5494	24,8202	NaN	NaN		2	2	2	17,2	17,2	17,2	15,237	4,60E+08	11	26,0462	26,2706	25,6721	8	5	3	0,40267	-0,598511	Q03BL5	Q03BL5	LSEI_0559	Phosphotransferase system, mannose/fructose-specific component IIA
25,7845	25,6159	25,551	25,0863	25,6422	25,7286	25,602	25,7202	NaN	NaN		2	2	2	8,9	8,9	8,9	26,692	2,94E+08	7	25,5913	25,536	25,6836	8	5	3	0,39478	0,14762	Q038Q6	Q038Q6	LSEI_1541	Ribosomal RNA small subunit methyltransferase E
26,4535	26,5054	26,4852	26,1351	26,5039	26,7599	NaN	26,9397	27,0232	NaN	+	2	2	2	9,4	9,4	9,4	33,124	4,59E+08	10	26,6007	26,4166	26,9076	8	5	3	0,00433399	0,490955	Q038A7	Q038A7	LSEI_1692	Lactoylglutathione lyase related lyase
22,6694	23,0085	NaN	NaN	22,5355	NaN	22,8489	22,9074	NaN	NaN		4	4	4	9,5	9,5	9,5	52,231	5,25E+07	7	22,7939	22,7378	22,8782	5	3	2	0,498939	0,140356	Q033U8	Q033U8	LSEI_2810	Amino acid ABC transporter membrane protein, PAAT family / amino acid ABC transporter substrate-binding protein, PAAT family
24,3936	NaN	NaN	25,1891	24,4546	24,7035	23,4807	NaN	NaN	NaN		4	4	4	29,6	29,6	29,6	17,993	1,66E+08	11	24,4443	24,6791	24,0921	5	3	2	0,373469	-0,587009	Q038M1	Q038M1	rimP	Ribosome maturation factor RimP
NaN	21,9915	22,7368	22,9023	NaN	24,8367	NaN	24,9261	NaN	NaN	+	2	2	2	2,9	2,9	2,9	97,895	8,81E+07	12	23,4787	22,5436	24,8814	5	3	2	0,00759984	2,33782	Q03BW3	Q03BW3	LSEI_0455	Uncharacterized protein encoded in toxicity protection region of plasmid R478, contains von Willebrand factor (VWF) domain
22,7987	23,409	23,1745	NaN	23,3717	23,4368	23,2857	NaN	NaN	NaN		3	3	3	8	8	8	59,788	6,05E+07	3	23,2461	23,1885	23,3613	6	4	2	0,465904	0,172815	Q037M6	Q037M6	LSEI_1837	Mga domain-containing protein
24,097	24,2244	24,1473	NaN	23,8475	NaN	23,7764	23,7832	NaN	NaN		3	3	3	7,6	7,6	7,6	53,504	1,22E+08	11	23,9793	24,0791	23,7798	6	4	2	0,070654	-0,299294	Q036C0	Q036C0	LSEI_2205	Cytochrome bd quinol oxidase subunit 1 apoprotein
NaN	25,6382	25,4299	25,348	24,9704	NaN	NaN	25,8736	25,7286	NaN		3	3	3	55,4	55,4	55,4	8,2803	2,21E+08	9	25,4981	25,3467	25,8011	6	4	2	0,100838	0,454432	Q036R1	Q036R1	LSEI_2058	Uncharacterized protein
28,0008	27,6156	NaN	28,0919	27,994	28,9345	28,8	NaN	NaN	NaN	+	2	2	2	5	5	5	49,716	1,64E+09	29	28,2395	27,9256	28,8672	6	4	2	0,00454106	0,94163	Q038K8	Q038K8	LSEI_1590	Amino acid/polyamine/organocation transporter, APC superfamily
25,728	21,2608	21,7855	22,1666	22,9138	NaN	21,4582	22,952	NaN	NaN		6	6	6	18	18	18	44,699	1,36E+08	14	22,6093	22,7709	22,2051	7	5	2	0,697655	-0,565847	Q033U7	Q033U7	argG	Argininosuccinate synthase
22,7408	22,7909	22,5124	22,2699	22,4049	22,4416	22,5593	NaN	NaN	NaN		2	2	2	10,2	10,2	10,2	28,65	4,41E+07	4	22,5314	22,5438	22,5005	7	5	2	0,807148	-0,043292	Q037S3	Q037S3	LSEI_1777	Transcriptional regulator, GntR family
22,3863	22,6782	22,5161	22,8892	22,8613	22,3702	NaN	22,7195	NaN	NaN		3	3	3	8,8	8,8	8,8	51,526	6,91E+07	12	22,6315	22,6662	22,5449	7	5	2	0,544974	-0,121371	Q035U6	Q035U6	LSEI_2282	Amidase family enzyme
22,131	22,3362	22,4626	22,5172	22,5667	22,651	22,5287	NaN	NaN	NaN		3	3	3	6,3	6,3	6,3	55,398	5,19E+07	6	22,4562	22,4027	22,5899	7	5	2	0,223014	0,187137	Q034M3	Q034M3	LSEI_2629	ABC-type proline/glycine betaine transport system, permease and substrate binding protein
22,0635	22,7216	22,6432	22,8188	22,6828	22,4997	22,6978	NaN	NaN	NaN		3	3	3	4,8	4,8	4,8	90,892	4,74E+07	9	22,5896	22,586	22,5988	7	5	2	0,957731	0,0128134	Q034L6	Q034L6	LSEI_2637	Excinuclease ATPase subunit
22,9767	22,8232	22,8492	22,832	22,8305	22,6143	NaN	NaN	22,6413	NaN	+	2	2	2	5,8	5,8	5,8	44,115	6,12E+07	5	22,7953	22,8623	22,6278	7	5	2	0,00490491	-0,234561	Q037V4	Q037V4	purD	Phosphoribosylamine--glycine ligase
21,821	22,7641	22,5632	22,6665	22,7862	22,7289	22,5321	NaN	NaN	NaN		2	2	2	10,6	10,6	10,6	22,664	4,63E+07	7	22,5517	22,5202	22,6305	7	5	2	0,731968	0,110259	Q035P3	Q035P3	LSEI_2335	CBS domain containing protein
22,5923	22,6223	22,96	23,088	22,8132	22,5816	22,6893	NaN	NaN	NaN		2	2	2	11,5	11,5	11,5	27,103	5,05E+07	4	22,7638	22,8152	22,6354	7	5	2	0,31847	-0,17971	Q036H7	Q036H7	sfsA	Sugar fermentation stimulation protein homolog
23,0108	22,7859	22,8312	22,794	23,1413	23,1198	NaN	NaN	NaN	22,6267		4	4	4	17,7	17,7	17,7	32,09	8,33E+07	5	22,9014	22,9126	22,8733	7	5	2	0,831449	-0,0393774	Q03BX4	Q03BX4	LSEI_0443	Aldo/keto reductase of diketogulonate reductase family
23,2833	23,7403	23,9621	23,8467	23,7289	NaN	NaN	22,7409	23,841	NaN		3	3	3	8,4	8,4	8,4	36,061	9,12E+07	12	23,5919	23,7123	23,2909	7	5	2	0,281474	-0,421317	Q038V4	Q038V4	LSEI_1492	Diphosphomevalonate decarboxylase
23,1228	23,3167	23,2935	23,2677	23,3141	23,2463	23,3441	NaN	NaN	NaN		4	4	4	6,4	6,4	6,4	69,661	1,04E+08	6	23,2722	23,263	23,2952	7	5	2	0,645091	0,032197	Q03A34	Q03A34	LSEI_1149	ABC-type multidrug transport system, ATPase and permease component
23,4496	23,441	23,4442	23,6277	23,4043	23,2577	23,3846	NaN	NaN	NaN		6	6	6	8,1	8,1	8,1	94,744	1,15E+08	13	23,4299	23,4734	23,3211	7	5	2	0,0946933	-0,152238	Q03BL6	Q03BL6	LSEI_0558	Transcriptional regulator containing an AAA-type ATPase domain and a DNA-binding domain
23,9966	23,6822	23,6352	23,4081	23,4491	23,2381	23,417	NaN	NaN	NaN		8	8	8	21,7	21,7	21,7	48,689	1,52E+08	13	23,5466	23,6342	23,3275	7	5	2	0,151676	-0,30671	Q039E9	Q039E9	LSEI_1395	Periplasmic protease
24,0616	23,5394	23,3625	23,229	23,6956	23,3756	23,4391	NaN	NaN	NaN		4	4	4	18	18	18	25,495	1,19E+08	8	23,529	23,5776	23,4073	7	5	2	0,513556	-0,170297	Q038Z4	Q038Z4	pyrF	Orotidine 5-phosphate decarboxylase
23,632	24,057	23,7464	23,7409	23,7025	23,4232	23,5975	NaN	NaN	NaN		4	4	4	18,1	18,1	18,1	30,35	1,71E+08	11	23,6999	23,7758	23,5103	7	5	2	0,098413	-0,265411	Q038G3	Q038G3	LSEI_1635	Predicted rRNA methylase
24,1073	24,1544	24,3296	24,6024	24,3972	23,9576	NaN	NaN	23,1674	NaN	+	3	3	3	18,1	18,1	18,1	20,429	1,14E+08	13	24,1023	24,3182	23,5625	7	5	2	0,0321025	-0,755725	Q038B6	Q038B6	LSEI_1683	Predicted metal-binding, possibly nucleic acid-binding protein
22,6021	23,7127	23,8386	24,1406	23,8193	23,5763	23,5587	NaN	NaN	NaN		5	5	5	32,2	32,2	32,2	16,234	1,36E+08	17	23,6069	23,6226	23,5675	7	5	2	0,905897	-0,0551098	Q039S3	Q039S3	mraZ	Transcriptional regulator MraZ
22,9391	23,3052	23,0435	22,9793	23,346	23,8114	NaN	23,5316	NaN	NaN	+	3	3	3	40,9	40,9	40,9	12,167	8,35E+07	10	23,2794	23,1226	23,6715	7	5	2	0,0186245	0,548871	Q037G9	Q037G9	LSEI_1894	Transcriptional regulator, ArsR family
23,9604	24,0893	24,0319	24,168	23,9193	23,6987	NaN	23,6721	NaN	NaN	+	5	5	5	34,7	34,7	34,7	18,965	1,80E+08	16	23,9342	24,0338	23,6854	7	5	2	0,00553264	-0,348394	Q037M9	Q037M9	LSEI_1828	Uncharacterized protein
23,6105	24,0331	23,545	23,5612	23,4942	NaN	23,721	23,7353	NaN	NaN		6	5	5	21,2	19	19	43,689	1,21E+08	7	23,6715	23,6488	23,7281	7	5	2	0,648591	0,0793371	Q036F3	Q036F3	ackA	Acetate kinase
24,2586	24,6379	24,545	24,4434	24,4111	NaN	NaN	23,2235	24,4487	NaN		4	4	4	28,3	28,3	28,3	19,203	2,17E+08	16	24,2812	24,4592	23,8361	7	5	2	0,127573	-0,623105	Q03B34	Q03B34	LSEI_0752	Acetyltransferase, GNAT family
23,5504	24,1494	23,6435	23,7987	23,4908	23,9704	23,8378	NaN	NaN	NaN		4	4	4	21,6	21,6	21,6	32,215	1,35E+08	13	23,7773	23,7266	23,9041	7	5	2	0,415881	0,177509	Q037H7	Q037H7	LSEI_1886	ABC-type oligopeptide transport system, ATPase component
23,3932	23,7003	23,4154	23,5593	23,6079	24,0731	NaN	NaN	23,9076	NaN	+	3	3	3	16	16	16	27,45	1,03E+08	5	23,6653	23,5352	23,9903	7	5	2	0,0079758	0,455085	Q03BF1	Q03BF1	LSEI_0629	Transcriptional regulator, GntR family
24,3505	25,5019	24,756	25,0002	24,89	24,2454	24,1186	NaN	NaN	NaN		4	4	4	29,7	29,7	29,7	16,161	2,37E+08	15	24,6947	24,8997	24,182	7	5	2	0,0708694	-0,717708	Q037Q2	Q037Q2	LSEI_1800	Uncharacterized protein
24,1088	23,8852	23,9142	24,0491	24,3528	24,5483	24,0019	NaN	NaN	NaN		2	2	2	21,7	21,7	21,7	17,142	1,11E+08	6	24,1229	24,062	24,2751	7	5	2	0,338172	0,213072	Q03B29	Q03B29	LSEI_0757	Ribonucleotide reduction protein
24,0881	24,26	24,3138	24,5334	24,1974	NaN	24,3155	24,346	NaN	NaN		5	5	5	13,5	13,5	13,5	47,242	1,84E+08	27	24,2935	24,2785	24,3308	7	5	2	0,690977	0,0522612	Q03B06	Q03B06	LSEI_0780	Transcriptional regulator with CBS domains
24,3824	24,5005	24,4487	24,4656	24,4451	24,313	NaN	24,3943	NaN	NaN		7	7	7	14,7	14,7	14,7	68,392	2,88E+08	13	24,4214	24,4485	24,3537	7	5	2	0,0578906	-0,094808	Q038N6	Q038N6	lepA	Elongation factor 4
23,8611	24,5279	24,3837	24,4638	24,3888	24,3921	24,3205	NaN	NaN	NaN		3	3	3	22,5	22,5	22,5	19,344	1,83E+08	17	24,334	24,3251	24,3563	7	5	2	0,881969	0,0312466	Q03AX9	Q03AX9	LSEI_0807	Small molecule binding protein, 3H domain
24,4314	24,8519	25,0103	24,5136	24,4171	24,0809	NaN	25,0718	NaN	NaN		5	5	5	15,7	15,7	15,7	37,358	1,41E+08	11	24,6253	24,6449	24,5764	7	5	2	0,844169	-0,0684952	Q03AK8	Q03AK8	LSEI_0966	Central glycolytic genes regulator
24,8612	24,3816	24,4182	24,1319	24,8666	25,2339	NaN	24,5434	NaN	NaN		3	3	3	49,7	49,7	49,7	15,935	1,75E+08	5	24,6338	24,5319	24,8886	7	5	2	0,291513	0,356769	Q036K9	Q036K9	LSEI_2113	Biotin carboxyl carrier protein of acetyl-CoA carboxylase
24,4586	25,2979	25,3933	25,5462	24,3955	25,4555	26,2801	NaN	NaN	NaN		2	2	2	28,8	28,8	28,8	12,259	2,40E+08	12	25,261	25,0183	25,8678	7	5	2	0,126712	0,849505	Q036T4	Q036T4	LSEI_2033	Transcriptional regulator, ArsR family
27,7971	28,3235	28,4697	28,3158	28,3739	28,2491	NaN	29,1349	NaN	NaN		5	5	5	14,8	14,8	14,8	63,624	1,90E+09	10	28,3806	28,256	28,692	7	5	2	0,214085	0,435953	Q036H9	Q036H9	LSEI_2143	Pyruvate oxidase
22,7773	22,8627	NaN	NaN	22,5605	NaN	22,7679	NaN	NaN	NaN		3	3	3	5,6	5,6	5,6	57,491	5,10E+07	3	22,7421	22,7335	22,7679	4	3	1	1	0,0344009	Q03CF3	Q03CF3	LSEI_0256	ATPase component of ABC transporter with duplicated ATPase domains
22,9539	NaN	23,2942	23,4705	NaN	NaN	NaN	23,5265	NaN	NaN		2	2	2	4,8	4,8	4,8	49,045	5,83E+07	10	23,3112	23,2395	23,5265	4	3	1	1	0,286968	Q03CC7	Q03CC7	LSEI_0285	Uncharacterized NAD(FAD)-dependent dehydrogenase
NaN	23,5093	23,3955	NaN	23,9456	NaN	NaN	NaN	23,837	NaN		2	2	2	3,6	3,6	3,6	69,229	9,57E+07	11	23,6718	23,6168	23,837	4	3	1	1	0,220242	Q033L7	Q033L7	LSEI_2897	Adhesion exoprotein
NaN	25,3525	25,0032	25,2562	NaN	NaN	NaN	NaN	24,428	NaN		2	2	2	13,7	13,7	13,7	20,909	1,64E+08	12	25,01	25,204	24,428	4	3	1	1	-0,776005	Q033K6	Q033K6	rnpA	Ribonuclease P protein component
22,0223	25,4068	25,2559	23,5348	NaN	NaN	NaN	NaN	NaN	22,0997		2	2	2	28,9	28,9	28,9	12,672	1,25E+08	10	23,6639	24,0549	22,0997	5	4	1	1	-1,95528	Q034L3	Q034L3	LSEI_2640	Uncharacterized protein
23,6128	22,6179	22,1621	NaN	22,5385	NaN	22,5219	NaN	NaN	NaN		3	3	3	23,2	23,2	23,2	18,584	6,81E+07	6	22,6906	22,7328	22,5219	5	4	1	1	-0,210982	Q038Q4	Q038Q4	LSEI_1543	Uncharacterized protein
23,134	23,2339	22,9734	22,3426	NaN	NaN	NaN	NaN	22,8571	NaN		3	3	3	10	10	10	35,001	6,22E+07	9	22,9082	22,921	22,8571	5	4	1	1	-0,0638571	Q03A71	Q03A71	LSEI_1110	Glycosyltransferase related enzyme
22,4916	23,1092	NaN	23,2032	23,1749	22,8878	NaN	NaN	NaN	NaN		2	2	2	8,1	8,1	8,1	37,507	5,19E+07	13	22,9733	22,9947	22,8878	5	4	1	1	-0,106856	Q03A93	Q03A93	LSEI_1086	Ribonuclease BN-like family enzyme
NaN	24,0914	24,7966	24,1244	24,0966	NaN	23,3652	NaN	NaN	NaN		2	2	2	17,5	17,5	17,5	20,321	1,24E+08	7	24,0948	24,2772	23,3652	5	4	1	1	-0,91204	Q039I5	Q039I5	LSEI_1359	BS_ykrK family protein
NaN	24,0855	23,8362	23,6253	23,4109	23,5909	NaN	NaN	NaN	NaN		7	7	7	9,7	9,7	9,7	81,383	1,16E+08	8	23,7098	23,7395	23,5909	5	4	1	1	-0,148569	Q036N1	Q036N1	LSEI_2089	Uncharacterized protein
23,8796	NaN	23,5247	23,637	23,5256	23,5956	NaN	NaN	NaN	NaN		2	2	2	10,5	10,5	10,5	26,56	6,58E+07	9	23,6325	23,6417	23,5956	5	4	1	1	-0,0461345	Q037I2	Q037I2	LSEI_1881	Uncharacterized ABC transporter, ATPase component
NaN	23,0702	24,0504	25,1236	23,9364	24,8884	NaN	NaN	NaN	NaN		2	2	2	30,5	30,5	30,5	6,7297	1,30E+08	5	24,2138	24,0452	24,8884	5	4	1	1	0,843202	Q038E2	Q038E2	LSEI_1657	Uncharacterized protein
25,0538	25,8227	NaN	24,8738	25,2865	26,5484	NaN	NaN	NaN	NaN		3	3	3	13	13	13	30,345	2,88E+08	11	25,517	25,2592	26,5484	5	4	1	1	1,28921	Q039W5	Q039W5	LSEI_1221	Uncharacterized protein
21,9731	22,3875	22,2981	21,7815	22,0911	22,1521	NaN	NaN	NaN	NaN		3	3	3	6,9	6,9	6,9	38,675	4,16E+07	6	22,1139	22,1063	22,1521	6	5	1	1	0,0457767	Q039L5	Q039L5	LSEI_1326	DNA polymerase III, delta subunit
22,0899	22,2276	22,2491	22,1694	22,1659	22,1822	NaN	NaN	NaN	NaN		4	4	4	7,9	7,9	7,9	79,575	4,68E+07	4	22,1807	22,1804	22,1822	6	5	1	1	0,00178986	Q038I3	Q038I3	recG	ATP-dependent DNA helicase RecG
22,4782	23,2127	23,1847	22,8242	22,7833	22,3655	NaN	NaN	NaN	NaN		3	3	3	5,1	5,1	5,1	68,901	6,28E+07	11	22,8081	22,8966	22,3655	6	5	1	1	-0,531125	Q039J7	Q039J7	uvrC	UvrABC system protein C
22,5989	22,6435	22,8111	22,91	22,5703	22,3945	NaN	NaN	NaN	NaN		3	3	3	7,3	7,3	7,3	53,995	5,98E+07	12	22,6547	22,7068	22,3945	6	5	1	1	-0,312268	Q036T0	Q036T0	glgA	Glycogen synthase
22,9514	23,0103	23,1426	22,9949	22,8031	22,7227	NaN	NaN	NaN	NaN		5	5	5	18,9	18,9	18,9	29,772	8,67E+07	7	22,9375	22,9805	22,7227	6	5	1	1	-0,257804	Q034T5	Q034T5	LSEI_2564	Uncharacterized protein
23,1394	23,4008	23,4463	23,2754	23,5318	22,8006	NaN	NaN	NaN	NaN		3	3	3	17,9	17,9	17,9	35,686	8,82E+07	4	23,2657	23,3587	22,8006	6	5	1	1	-0,558156	Q03AE3	Q03AE3	LSEI_1034	Lipopolysaccharide biosynthesis glycosyltransferase
24,0989	22,7695	22,9185	22,9215	22,811	22,8794	NaN	NaN	NaN	NaN		2	2	2	4,7	4,7	4,7	52,485	9,28E+07	7	23,0665	23,1039	22,8794	6	5	1	1	-0,224476	Q03BE7	Q03BE7	LSEI_0633	Amino acid/polyamine/organocation transporter, APC superfamily
22,8683	23,0257	23,1492	23,1494	23,3115	22,9225	NaN	NaN	NaN	NaN		2	2	2	5,8	5,8	5,8	53,767	6,51E+07	7	23,0711	23,1008	22,9225	6	5	1	1	-0,178331	Q03D23	Q03D23	LSEI_0033	Dipeptidase
22,4932	23,2617	23,1726	23,3231	22,8255	NaN	22,9571	NaN	NaN	NaN		3	3	3	6	6	6	70,377	6,73E+07	9	23,0055	23,0152	22,9571	6	5	1	1	-0,0581017	Q03AB3	Q03AB3	LSEI_1065	Neutral endopeptidase
23,2469	23,478	23,3327	23,4511	23,2517	23,0629	NaN	NaN	NaN	NaN		4	4	4	16,2	16,2	16,2	52,388	1,16E+08	7	23,3039	23,3521	23,0629	6	5	1	1	-0,289166	Q03BY9	Q03BY9	LSEI_0427	DNA polymerase III, alpha subunit (Gram-positive type)
22,7688	23,2659	23,1069	23,2754	23,2549	23,2842	NaN	NaN	NaN	NaN		2	2	2	6	6	6	32,252	7,28E+07	12	23,1594	23,1344	23,2842	6	5	1	1	0,149797	Q039R6	Q039R6	divIB	Cell division protein DivIB
23,9802	23,444	23,3016	23,3086	23,5229	NaN	23,2914	NaN	NaN	NaN		3	3	3	12	12	12	33,686	1,03E+08	7	23,4748	23,5114	23,2914	6	5	1	1	-0,220077	Q03AB4	Q03AB4	LSEI_1064	Polyphosphate:AMP phosphotransferase
23,5334	24,0312	23,9208	23,8777	23,9138	NaN	NaN	23,7129	NaN	NaN		4	4	4	18,3	18,3	18,3	28,267	1,24E+08	7	23,8316	23,8554	23,7129	6	5	1	1	-0,142453	Q035E0	Q035E0	phnC	Phosphonates import ATP-binding protein PhnC
23,1827	24,9571	24,3119	24,4413	24,3142	23,885	NaN	NaN	NaN	NaN		2	2	2	4,2	4,2	4,2	28,712	1,00E+08	7	24,182	24,2414	23,885	6	5	1	1	-0,356427	Q038L7	Q038L7	LSEI_1581	Phosphatidate cytidylyltransferase
24,0449	24,1338	23,8623	23,5947	23,7547	NaN	23,8951	NaN	NaN	NaN		4	4	4	15,2	15,2	15,2	42,874	1,19E+08	10	23,8809	23,8781	23,8951	6	5	1	1	0,0170589	Q036S9	Q036S9	LSEI_2038	ADP-glucose pyrophosphorylase
23,2246	24,1205	23,8169	23,611	23,6012	NaN	NaN	23,9714	NaN	NaN		3	3	3	9,2	9,2	9,2	35,888	1,17E+08	7	23,7243	23,6748	23,9714	6	5	1	1	0,296561	Q037H4	Q037H4	LSEI_1889	ABC-type dipeptide/oligopeptide/nickel transport system, permease component
25,183	24,2296	24,0351	24,13	22,7207	24,0228	NaN	NaN	NaN	NaN		4	4	4	9,3	9,3	9,3	50,86	1,13E+08	13	24,0535	24,0597	24,0228	6	5	1	1	-0,0368988	Q03CQ7	Q03CQ7	LSEI_0151	Xanthine/uracil/vitamin C permease
23,9997	24,4125	24,5328	24,6343	24,408	24,294	NaN	NaN	NaN	NaN		4	4	4	12,2	12,2	12,2	35,493	1,38E+08	10	24,3802	24,3975	24,294	6	5	1	1	-0,1035	Q035S8	Q035S8	LSEI_2300	Geranylgeranyl pyrophosphate synthase
24,2268	24,4585	23,9784	23,9927	24,2559	24,4445	NaN	NaN	NaN	NaN		3	3	3	7,7	7,7	7,7	45,107	1,06E+08	9	24,2261	24,1825	24,4445	6	5	1	1	0,262025	Q038L8	Q038L8	LSEI_1580	Zinc metalloprotease
24,7914	24,6208	24,4281	24,4809	24,5401	24,473	NaN	NaN	NaN	NaN		4	4	4	17,6	17,6	17,6	42,588	1,75E+08	12	24,5557	24,5723	24,473	6	5	1	1	-0,0992401	Q03BX7	Q03BX7	LSEI_0440	Predicted metal-dependent hydrolase of the TIM-barrel fold
26,4201	25,3827	25,4206	25,2962	24,7867	24,9186	NaN	NaN	NaN	NaN		3	3	3	15	15	15	45,112	2,96E+08	12	25,3708	25,4613	24,9186	6	5	1	1	-0,542698	Q038Z0	Q038Z0	pyrC	Dihydroorotase
23,755	24,8287	24,3569	24,807	24,3359	25,079	NaN	NaN	NaN	NaN		4	4	4	39,4	39,4	39,4	11,024	1,76E+08	17	24,5271	24,4167	25,079	6	5	1	1	0,662297	Q035P0	Q035P0	LSEI_2338	Uncharacterized protein
23,1819	24,2135	24,3498	NaN	NaN	NaN	NaN	NaN	NaN	NaN		2	2	2	15,5	15,5	15,5	20,412	9,37E+07	6	23,9151	23,9151	NaN	3	3	0	1	NaN	Q034Y0	Q034Y0	LSEI_2506	Alkyl hydroperoxide reductase C
NaN	NaN	21,984	22,3042	21,8878	NaN	NaN	NaN	NaN	NaN		2	2	2	53,1	53,1	53,1	5,7966	1,82E+07	6	22,0587	22,0587	NaN	3	3	0	1	NaN	Q035F2	Q035F2	LSEI_2434	Uncharacterized protein
21,9859	NaN	22,4635	NaN	22,6738	NaN	NaN	NaN	NaN	NaN		2	2	2	7	7	7	44,966	4,03E+07	3	22,3744	22,3744	NaN	3	3	0	1	NaN	Q035L2	Q035L2	LSEI_2368	DUF2075 domain-containing protein
NaN	25,7078	22,978	24,0631	NaN	NaN	NaN	NaN	NaN	NaN		2	2	2	15,1	15,1	15,1	19,037	1,03E+08	6	24,2496	24,2496	NaN	3	3	0	1	NaN	Q035X6	Q035X6	folT	Folate transporter FolT
22,7125	23,01	NaN	23,0635	NaN	NaN	NaN	NaN	NaN	NaN		5	5	5	9,9	9,9	9,9	82,436	8,21E+07	5	22,9287	22,9287	NaN	3	3	0	1	NaN	Q036P1	Q036P1	LSEI_2079	Alpha-galactosidase
23,3259	NaN	23,423	23,514	NaN	NaN	NaN	NaN	NaN	NaN		2	2	2	5,8	5,8	5,8	32,421	6,76E+07	2	23,421	23,421	NaN	3	3	0	1	NaN	Q037L4	Q037L4	LSEI_1849	Ribosomal large subunit pseudouridine synthase D
NaN	25,1495	24,7289	NaN	24,4546	NaN	NaN	NaN	NaN	NaN		2	2	2	20,5	20,5	20,5	16,657	1,58E+08	3	24,7777	24,7777	NaN	3	3	0	1	NaN	Q037X2	Q037X2	argR	Arginine repressor
NaN	24,4817	24,6787	24,7022	NaN	NaN	NaN	NaN	NaN	NaN		2	2	2	31,5	31,5	31,5	8,2099	8,62E+07	6	24,6209	24,6209	NaN	3	3	0	1	NaN	Q038K4	Q038K4	LSEI_1594	Uncharacterized protein
24,9454	NaN	22,8868	NaN	23,6608	NaN	NaN	NaN	NaN	NaN		3	3	3	6,5	6,5	6,5	39,595	1,15E+08	7	23,831	23,831	NaN	3	3	0	1	NaN	Q039A3	Q039A3	LSEI_1441	Predicted membrane protein, putative toxin regulator
NaN	23,2008	22,8363	22,8403	NaN	NaN	NaN	NaN	NaN	NaN		2	2	2	12,9	12,9	12,9	19,713	5,02E+07	2	22,9591	22,9591	NaN	3	3	0	1	NaN	Q039Q3	Q039Q3	LSEI_1286	NUDIX family hydrolase
23,2505	23,3483	23,2957	NaN	NaN	NaN	NaN	NaN	NaN	NaN		3	3	3	20,7	20,7	20,7	27,52	6,80E+07	7	23,2982	23,2982	NaN	3	3	0	1	NaN	Q03AD7	Q03AD7	LSEI_1041	DNA-binding response regulator, OmpR family (Rec-wHTH domains)
NaN	23,7148	NaN	24,2666	24,234	NaN	NaN	NaN	NaN	NaN		3	3	3	17,3	17,3	17,3	24,942	5,64E+07	6	24,0718	24,0718	NaN	3	3	0	1	NaN	Q03AI4	Q03AI4	LSEI_0992	Acetyltransferase, GNAT family
25,1077	NaN	24,4149	24,5226	NaN	NaN	NaN	NaN	NaN	NaN		3	3	3	15,5	15,5	15,5	29,892	1,33E+08	8	24,6817	24,6817	NaN	3	3	0	1	NaN	Q03AS0	Q03AS0	nadK	NAD kinase
22,3788	22,2676	NaN	NaN	22,2833	NaN	NaN	NaN	NaN	NaN		2	2	2	13,2	13,2	13,2	22,541	3,19E+07	2	22,3099	22,3099	NaN	3	3	0	1	NaN	Q03AS2	Q03AS2	LSEI_0900	CYTH domain-containing protein
23,9933	NaN	23,3918	22,7769	NaN	NaN	NaN	NaN	NaN	NaN		2	2	2	8,7	8,7	8,7	25,973	6,55E+07	5	23,3873	23,3873	NaN	3	3	0	1	NaN	Q03AU0	Q03AU0	LSEI_0881	Uncharacterized protein
NaN	21,8889	21,7752	NaN	21,6566	NaN	NaN	NaN	NaN	NaN		2	2	2	4	4	4	54,511	1,76E+07	3	21,7736	21,7736	NaN	3	3	0	1	NaN	Q03AU3	Q03AU3	LSEI_0878	Permease of the major facilitator superfamily
21,9828	23,2761	22,9679	NaN	NaN	NaN	NaN	NaN	NaN	NaN		2	2	2	15,6	15,6	15,6	18,156	4,41E+07	10	22,7423	22,7423	NaN	3	3	0	1	NaN	Q03BU9	Q03BU9	LSEI_0472	Predicted periplasmic/secreted protein
NaN	22,4696	22,5813	22,2559	NaN	NaN	NaN	NaN	NaN	NaN		6	6	6	7,8	7,8	7,8	84,856	5,17E+07	9	22,4356	22,4356	NaN	3	3	0	1	NaN	Q03C12	Q03C12	LSEI_0404	Alpha-glucosidase, family 31 of glycosyl hydrolase
23,1461	NaN	22,9384	NaN	23,0853	NaN	NaN	NaN	NaN	NaN		3	3	3	16,1	16,1	16,1	32,247	5,96E+07	5	23,0566	23,0566	NaN	3	3	0	1	NaN	Q03CR0	Q03CR0	LSEI_0148	NADPH:quinone reductase related Zn-dependent oxidoreductase
23,4716	23,8426	23,7464	NaN	NaN	NaN	NaN	NaN	NaN	NaN		5	5	5	16	16	16	43,316	1,04E+08	8	23,6869	23,6869	NaN	3	3	0	1	NaN	Q03CU0	Q03CU0	LSEI_0118	Uncharacterized protein
22,6083	22,3375	22,3509	NaN	NaN	NaN	NaN	NaN	NaN	NaN		2	2	2	5,1	5,1	5,1	47,944	4,08E+07	3	22,4322	22,4322	NaN	3	3	0	1	NaN	Q03CW0	Q03CW0	lysA	Diaminopimelate decarboxylase
NaN	22,8468	22,6804	22,5156	22,7703	NaN	NaN	NaN	NaN	NaN		2	2	2	11,7	11,7	11,7	29,666	3,57E+07	3	22,7033	22,7033	NaN	4	4	0	1	NaN	Q034N3	Q034N3	LSEI_2619	SAM-dependent methyltransferase
NaN	23,5161	23,4044	23,506	23,412	NaN	NaN	NaN	NaN	NaN		5	5	5	31,7	31,7	31,7	25,105	1,23E+08	8	23,4596	23,4596	NaN	4	4	0	1	NaN	Q034P3	Q034P3	LSEI_2609	NAD-dependent protein deacetylase, SIR2 family
22,7118	22,7524	23,026	23,3261	NaN	NaN	NaN	NaN	NaN	NaN		4	4	4	25	25	25	21,125	3,58E+07	9	22,9541	22,9541	NaN	4	4	0	1	NaN	Q037Z2	Q037Z2	coaE	Dephospho-CoA kinase
22,5741	22,872	22,915	22,8546	NaN	NaN	NaN	NaN	NaN	NaN		2	2	2	11,8	11,8	11,8	22,849	4,85E+07	7	22,8039	22,8039	NaN	4	4	0	1	NaN	Q039S8	Q039S8	LSEI_1261	Amino acid ABC transporter ATP-binding protein, PAAT family
25,784	26,933	26,2897	26,427	NaN	NaN	NaN	NaN	NaN	NaN		2	2	2	37,4	37,4	37,4	10,796	2,97E+08	10	26,3584	26,3584	NaN	4	4	0	1	NaN	Q03A10	Q03A10	LSEI_1174	Glycine cleavage system H protein (Lipoate-binding)
23,1366	NaN	23,3113	23,4102	23,3314	NaN	NaN	NaN	NaN	NaN		3	3	3	20	20	20	26,329	8,75E+07	5	23,2974	23,2974	NaN	4	4	0	1	NaN	Q03A37	Q03A37	LSEI_1146	Phosphoglycerate mutase family protein
22,8511	23,3329	22,6719	22,5742	NaN	NaN	NaN	NaN	NaN	NaN		3	3	3	14,8	14,8	14,8	31,494	4,01E+07	4	22,8575	22,8575	NaN	4	4	0	1	NaN	Q03BU2	Q03BU2	LSEI_0479	Serine O-acetyltransferase
22,2597	23,0309	22,8029	22,6896	NaN	NaN	NaN	NaN	NaN	NaN		3	3	3	5,2	5,2	5,2	57,189	4,02E+07	4	22,6958	22,6958	NaN	4	4	0	1	NaN	Q03CI1	Q03CI1	LSEI_0228	Gluconate kinase, FGGY family
23,2989	23,1398	23,079	23,1548	23,1827	NaN	NaN	NaN	NaN	NaN		2	2	2	5	5	5	80,514	5,84E+07	3	23,1711	23,1711	NaN	5	5	0	1	NaN	Q033K4	Q033K4	LSEI_A01	Cation transport ATPase
23,2771	23,4669	23,4981	23,4004	23,4147	NaN	NaN	NaN	NaN	NaN		2	2	2	11,8	11,8	11,8	32,147	6,76E+07	4	23,4114	23,4114	NaN	5	5	0	1	NaN	Q033R5	Q033R5	LSEI_2847	Fructosamine-3-kinase
23,8782	24,0064	23,4477	23,6007	23,6586	NaN	NaN	NaN	NaN	NaN		4	4	4	9,5	9,5	9,5	53,501	1,44E+08	8	23,7183	23,7183	NaN	5	5	0	1	NaN	Q033V2	Q033V2	LSEI_2806	YycH domain-containing protein
23,4118	23,126	23,2309	23,1445	23,3177	NaN	NaN	NaN	NaN	NaN		4	4	4	13,5	13,5	13,5	28,382	9,06E+07	4	23,2462	23,2462	NaN	5	5	0	1	NaN	Q033X0	Q033X0	LSEI_2788	Protein tyrosine/serine phosphatase
23,5803	24,5344	24,1831	23,7343	23,614	NaN	NaN	NaN	NaN	NaN		7	7	7	24,3	24,3	24,3	35,357	2,22E+08	9	23,9292	23,9292	NaN	5	5	0	1	NaN	Q034N7	Q034N7	LSEI_2615	Exopolyphosphatase
23,3231	22,7031	22,5969	22,4477	22,0107	NaN	NaN	NaN	NaN	NaN		3	3	3	4,2	4,2	4,2	76,69	4,60E+07	6	22,6163	22,6163	NaN	5	5	0	1	NaN	Q035P5	Q035P5	kup	Probable potassium transport system protein kup
23,1553	23,0439	23,0155	23,1703	23,2413	NaN	NaN	NaN	NaN	NaN		2	2	2	11,3	11,3	11,3	33,216	5,90E+07	3	23,1253	23,1253	NaN	5	5	0	1	NaN	Q035P6	Q035P6	LSEI_2332	ABC-type multidrug transport system, ATPase component
22,0285	22,1947	22,2491	22,2009	22,0974	NaN	NaN	NaN	NaN	NaN		3	3	3	6,2	6,2	6,2	50,308	2,78E+07	5	22,1541	22,1541	NaN	5	5	0	1	NaN	Q037S6	Q037S6	LSEI_1774	23S rRNA m(5)U-1939 methyltransferase
23,5554	23,837	23,4152	23,818	24,1383	NaN	NaN	NaN	NaN	NaN		5	5	5	27,3	27,3	27,3	19,359	1,24E+08	24	23,7528	23,7528	NaN	5	5	0	1	NaN	Q037T2	Q037T2	LSEI_1768	Uncharacterized protein
22,4385	22,6255	22,522	22,7009	22,6892	NaN	NaN	NaN	NaN	NaN		2	2	2	9,4	9,4	9,4	28,198	4,64E+07	6	22,5952	22,5952	NaN	5	5	0	1	NaN	Q037W1	Q037W1	LSEI_1739	ABC-type antimicrobial peptide transport system, ATPase component
25,111	25,0208	25,0692	25,181	24,9342	NaN	NaN	NaN	NaN	NaN		2	2	2	4,5	4,5	4,5	55,468	1,72E+08	6	25,0632	25,0632	NaN	5	5	0	1	NaN	Q038Y0	Q038Y0	LSEI_1464	Cardiolipin synthase
23,6672	23,5444	23,5152	23,6155	23,5598	NaN	NaN	NaN	NaN	NaN		3	3	3	9	9	9	42,374	1,06E+08	5	23,5804	23,5804	NaN	5	5	0	1	NaN	Q03A03	Q03A03	LSEI_1182	Cysteine desulfurase
23,5984	23,7804	23,8465	23,8681	24,2452	NaN	NaN	NaN	NaN	NaN		3	3	3	13,4	13,4	13,4	42,612	9,09E+07	4	23,8677	23,8677	NaN	5	5	0	1	NaN	Q03A09	Q03A09	LSEI_1175	Probable succinyl-diaminopimelate desuccinylase
23,8675	23,628	23,037	24,3893	23,8041	NaN	NaN	NaN	NaN	NaN		3	3	3	34,8	34,8	34,8	10,386	9,63E+07	5	23,7452	23,7452	NaN	5	5	0	1	NaN	Q03AU9	Q03AU9	LSEI_0866	Uncharacterized protein
22,5328	22,8637	22,7985	22,5433	22,7391	NaN	NaN	NaN	NaN	NaN		3	3	3	10,1	10,1	10,1	31,565	5,03E+07	7	22,6955	22,6955	NaN	5	5	0	1	NaN	Q03C05	Q03C05	LSEI_0411	ABC-type metal ion transport system, periplasmic component/surface antigen
23,4074	23,526	23,4689	23,6423	23,5735	NaN	NaN	NaN	NaN	NaN		3	3	3	15,2	15,2	15,2	32,036	7,33E+07	8	23,5236	23,5236	NaN	5	5	0	1	NaN	Q03CG5	Q03CG5	LSEI_0244	Diadenosine tetraphosphatase related serine/threonine protein phosphatase

Table S3. Total data of proteins identified in this study for *Enterococcus faecium* 74 along with Log2 fold change (in blue) and significance values (p, in red) of the 30 µg/mL punicalagin (P) treatment in comparison to the non-treated control (B). Control samples in yellow, samples treated with P in green.

LFQ intensity 126C_1	LFQ intensity 126C_2	LFQ intensity 126C_3	LFQ intensity 126C_4	LFQ intensity 126C_5	LFQ intensity 126P_1	LFQ intensity 126P_2	LFQ intensity 126P_3	LFQ intensity 126P_4	LFQ intensity 126P_5	T-test Significant	Peptides	Razor + unique peptides	Unique peptides	Sequence coverage [%]	Unique + razor sequence coverage [%]	Unique sequence coverage [%]	Mol. weight [kDa]	Intensity	MS/MS count	Mean	Mean Control	Mean Punicalagin	Valid values	Valid values Control	Valid values Punicalagin	T-test p-value	T-test Difference	Protein IDs	Majority protein IDs	Gene name	Protein name
27,8867	27,8485	27,8778	27,8051	27,6917	27,1643	26,9115	27,0866	27,1778	26,3245	+	4	4	4	50	50	50	11,101	4,01E+09	43	27,3775	27,822	26,9329	10	5	5	0,000611643	-0,889014	A0A132P6P5	A0A132P6P5	rplU	50S ribosomal protein L21
23,0945	23,1871	23,3022	23,5773	23,3062	22,3707	22,5355	22,4618	23,0028	22,3789	+	2	2	2	13,2	13,2	13,2	16,634	1,56E+08	10	22,9217	23,2935	22,55	10	5	5	0,000804586	-0,743498	A0A132Z5E1	A0A132Z5E1	hit	HIT domain-containing protein
27,5333	27,5079	27,4917	27,4492	27,4615	26,9001	26,6267	26,6934	27,0574	26,4737	+	8	8	8	49,8	49,8	49,8	22,544	6,58E+09	91	27,1195	27,4887	26,7503	10	5	5	0,000102219	-0,738424	D8V1G6	D8V1G6	rplD	50S ribosomal protein L4
27,7712	27,77	27,7294	27,7264	27,5291	26,9618	26,9276	26,9933	27,3807	26,7655	+	9	9	9	61,6	61,6	61,6	15,481	3,37E+09	87	27,3555	27,7052	27,0058	10	5	5	0,000234761	-0,699412	A0A132P5G2	A0A132P5G2	rplO	50S ribosomal protein L15
27,3663	27,7675	27,947	28,0203	27,9043	27,2454	27,1013	27,0581	27,2927	26,8723	+	6	6	6	33,5	33,5	33,5	22,778	2,72E+09	55	27,4575	27,8011	27,114	10	5	5	0,00108232	-0,6871	A0A132P5N2	A0A132P5N2	rplC_2	50S ribosomal protein L3
24,6854	24,3185	24,4572	24,4831	24,4187	23,9489	23,9015	23,9574	24,0754	23,0705	+	3	3	3	11,7	11,7	11,7	33,877	3,49E+08	15	24,1317	24,4726	23,7907	10	5	5	0,00748886	-0,68185	A0A133CQG2	A0A133CQG2	arcC	Carbamate kinase
25,0294	25,0069	25,0135	24,8874	24,9969	24,365	24,1352	24,5001	24,4932	24,2207	+	4	4	4	8,4	8,4	8,4	67,621	7,53E+08	28	24,6648	24,9868	24,3428	10	5	5	3,19E-05	-0,643977	A0A132PAP8	A0A132PAP8	fruA_3	PTS fructose transporter subunit II
25,2842	25,0737	25,1693	25,2186	25,2096	24,7127	24,5528	24,3842	24,8398	24,3556	+	9	9	9	45	45	45	26,931	1,05E+09	42	24,88	25,1911	24,569	10	5	5	0,000244945	-0,622055	A0A133CKH2	A0A133CKH2	fabI	Enoyl-[acyl-carrier-protein] reductase [NADH]
25,2393	25,0612	25,2268	25,1225	25,1479	24,7103	24,3915	24,3005	24,8712	24,44	+	3	3	3	31,6	31,6	31,6	19,606	6,42E+08	18	24,8511	25,1595	24,5427	10	5	5	0,000563308	-0,616857	A0A133CVC2	A0A133CVC2	infC	Translation initiation factor IF-3
25,2256	25,2723	25,2846	25,2965	25,2427	24,6439	24,4774	24,5905	24,7885	24,7565	+	16	16	16	40,6	40,6	40,6	56,48	2,24E+09	99	24,9579	25,2643	24,6514	10	5	5	5,64E-06	-0,612956	A0A2A8RHK3	A0A2A8RHK3	cshA	DEAD-box ATP-dependent RNA helicase
23,6086	23,6052	23,8809	23,816	23,7157	23,2327	23,1497	23,1099	23,3868	22,778	+	9	9	9	28,5	28,5	28,5	41,769	4,16E+08	34	23,4284	23,7253	23,1314	10	5	5	0,000831175	-0,593847	A0A132Z2H8	A0A132Z2H8	A5810_0020	Uncharacterized protein
26,5654	26,6065	26,8001	26,7358	26,8322	26,2665	26,0272	26,1146	26,3787	25,7985	+	7	7	7	28,6	28,6	28,6	33,206	4,70E+09	82	26,4125	26,708	26,1171	10	5	5	0,00079617	-0,590931	A0A133CST4	A0A133CST4	manZ_1	PTS mannose family transporter subunit IID
26,8393	26,8059	26,8435	26,8669	26,8313	26,3425	26,1793	26,1823	26,5839	25,9789	+	11	11	11	53,3	53,3	53,3	24,536	5,58E+09	176	26,5454	26,8374	26,2534	10	5	5	0,000420075	-0,583988	A0A132Z3T8	A0A132Z3T8	rplA	50S ribosomal protein L1
26,1623	26,5956	25,7762	25,6623	26,2939	25,302	25,2456	25,5402	25,7957	25,6886	+	22	22	22	48,2	48,2	48,2	70,329	5,39E+09	169	25,8062	26,0981	25,5144	10	5	5	0,0199454	-0,583621	Q1JTV5	Q1JTV5	pheDC	Phenylalanine and tyrosine decarboxylase
24,9641	25,1246	25,1147	24,9788	24,9743	24,4689	24,3772	24,5541	24,3273	24,5627	+	13	13	13	19,1	19,1	19,1	84,576	1,26E+09	54	24,7447	25,0313	24,458	10	5	5	1,08E-05	-0,573235	A0A132P9P2	A0A132P9P2	infB	Translation initiation factor IF-2
25,1839	25,2151	25,2853	25,2979	25,3197	24,9609	24,7089	24,6626	24,7307	24,4468	+	8	8	8	47,4	47,4	47,4	23,827	1,23E+09	70	24,9812	25,2604	24,702	10	5	5	0,000189944	-0,558377	A0A133CTJ1	A0A133CTJ1	CUN04_1018	Epimerase
24,4794	24,4887	24,5117	24,4968	24,9142	24,0011	23,9578	24,1978	24,064	23,9078	+	10	10	10	26,9	26,9	26,9	52,907	9,89E+08	51	24,3019	24,5781	24,0257	10	5	5	0,00048678	-0,552443	A0A132Z2I4	A0A132Z2I4	gatA	Glutamyl-tRNA(Gln) amidotransferase subunit A
24,5702	24,3545	24,3049	24,2352	24,3844	23,8906	23,8573	23,9093	23,9271	23,6135	+	9	9	9	22,3	22,3	22,3	49,58	6,86E+08	41	24,1047	24,3698	23,8396	10	5	5	0,000171202	-0,530255	A0A133CK82	A0A133CK82	carB2	Carbamoyl phosphate synthase-like protein
27,0103	26,8467	26,9779	26,846	26,9453	26,4458	26,321	26,4216	26,5774	26,219	+	18	18	18	56,7	56,7	56,7	47,508	6,48E+09	169	26,6611	26,9252	26,397	10	5	5	6,13E-05	-0,528289	A0A133CHB5	A0A133CHB5	tyrS1	Tyrosine--tRNA ligase
24,6582	24,679	24,6365	24,7258	24,8264	24,3994	24,1212	24,1216	24,3372	23,9077	+	6	6	6	24,9	24,9	24,9	26,13	6,69E+08	37	24,4413	24,7052	24,1774	10	5	5	0,000500018	-0,527729	A0A133CHJ6	A0A133CHJ6	fabG	3-oxoacyl-[acyl-carrier-protein] reductase
25,1082	25,1646	25,027	24,9286	24,9961	24,3289	24,5519	24,6204	24,7099	24,4069	+	5	5	5	39,5	39,5	39,5	21,422	6,36E+08	21	24,7843	25,0449	24,5236	10	5	5	0,0001999	-0,521297	A0A132P3C8	A0A132P3C8	rfbC	dTDP-4-dehydrorhamnose 3,5-epimerase
23,8926	24,0692	23,954	23,9912	24,1394	23,4466	23,4499	23,4223	23,7661	23,3731	+	6	6	6	21,5	21,5	21,5	41,87	4,36E+08	29	23,7504	24,0093	23,4916	10	5	5	0,000235185	-0,517728	A0A132Z8I9	A0A132Z8I9	dnaB_1	Beta sliding clamp
26,3586	26,2994	26,2677	26,4058	26,3389	26,0503	25,7126	25,7151	26,1379	25,5403	+	10	10	10	59,4	59,4	59,4	11,041	3,17E+09	84	26,0827	26,3341	25,8312	10	5	5	0,00240175	-0,502831	A0A0M1PHS8	A0A0M1PHS8	rplW_2	50S ribosomal protein L23
23,6299	23,5953	23,7212	23,6582	23,8306	23,2772	23,2863	23,2064	23,3576	22,7951	+	4	4	4	23,1	23,1	23,1	21,231	3,14E+08	23	23,4358	23,687	23,1845	10	5	5	0,0016825	-0,502482	A0A132Z571	A0A132Z571	AWT83_1300	DUF1269 domain-containing protein
24,2401	24,1982	24,0418	24,0993	24,2071	23,5343	23,489	23,8924	23,7145	23,6447	+	14	14	14	19,5	19,5	19,5	89,723	1,06E+09	61	23,9061	24,1573	23,655	10	5	5	0,000249634	-0,502337	A0A133CK83	A0A133CK83	pheT	Phenylalanine--tRNA ligase beta subunit
27,0061	26,9069	26,9682	26,9287	26,8703	26,5437	26,3907	26,5012	26,6157	26,1199	+	16	16	16	59,1	59,1	59,1	23,167	8,09E+09	180	26,6851	26,9361	26,4342	10	5	5	0,000517119	-0,501826	A0A132P597	A0A132P597	rpsD	30S ribosomal protein S4
27,5873	27,4635	27,5152	27,5388	27,4734	27,1398	26,9192	27,0135	27,2773	26,7363	+	4	4	4	25,2	25,2	25,2	12,45	4,54E+09	72	27,2664	27,5156	27,0172	10	5	5	0,00078856	-0,498405	D8V1H1	D8V1H1	rplV	50S ribosomal protein L22
24,9215	24,8331	24,839	24,6923	24,8066	24,2062	24,2871	24,4049	24,507	24,2701	+	7	7	7	29	29	29	43,413	8,30E+08	41	24,5768	24,8185	24,3351	10	5	5	7,47E-05	-0,483414	A0A133CHH8	A0A133CHH8	fabF	3-oxoacyl-[acyl-carrier-protein] synthase 2
24,3764	24,2912	24,2628	24,1762	24,3058	23,6885	23,5888	23,9843	24,0126	23,7279	+	8	8	8	37,3	37,3	37,3	39,25	9,07E+08	42	24,0414	24,2824	23,8004	10	5	5	0,000688411	-0,482047	A0A132Z810	A0A132Z810	pepQ_1	Aminopeptidase P family protein
27,5455	27,2274	27,3595	27,1744	27,2156	26,9224	26,6771	26,81	27,0405	26,71	+	11	11	11	60,3	60,3	60,3	13,534	3,81E+09	99	27,0682	27,3045	26,832	10	5	5	0,00113436	-0,472445	A0A0M1PG91	A0A0M1PG91	rpsM	30S ribosomal protein S13
23,0791	23,0639	23,1571	23,1409	23,0683	22,7042	22,6039	22,5904	22,8474	22,4086	+	5	5	5	23,8	23,8	23,8	25,595	1,98E+08	18	22,8664	23,1019	22,6309	10	5	5	0,000231926	-0,470995	A0A132Z8Z5	A0A132Z8Z5	B1P95_09875	DUF1129 domain-containing protein
24,3382	24,2861	24,3384	24,2368	24,4876	23,6573	23,7325	24,1112	24,1308	23,7335	+	15	15	15	42,7	42,7	42,7	58,673	1,22E+09	50	24,1052	24,3374	23,8731	10	5	5	0,00298823	-0,464371	A0A132P3G6	A0A132P3G6	zwf	Glucose-6-phosphate 1-dehydrogenase
24,7636	24,5938	24,4714	24,4573	24,5375	24,1777	23,9847	23,9705	24,1323	24,2583	+	9	9	9	24,7	24,7	24,7	47,946	9,85E+08	41	24,3347	24,5647	24,1047	10	5	5	0,000380921	-0,460044	A0A133CJ11	A0A133CJ11	ftsA	Cell division protein FtsA
26,2953	26,2363	26,2394	26,193	26,2063	25,78	25,6997	25,8725	25,767	25,7526	+	17	17	17	35,8	35,8	35,8	72,406	4,82E+09	116	26,0042	26,2341	25,7744	10	5	5	7,11E-07	-0,459687	A0A133CKH8	A0A133CKH8	tkt	Transketolase
25,5258	25,9843	26,09	25,963	26,3516	25,5867	25,4953	25,4356	25,5865	25,5139	+	10	10	10	18,5	18,5	18,5	72,927	1,07E+09	22	25,7533	25,9829	25,5236	10	5	5	0,00989813	-0,459338	A0A132Z0T1	A0A132Z0T1	asnB	Asparagine synthase (Glutamine-hydrolyzing)
23,8431	23,8291	23,8233	23,7292	23,9001	23,3974	23,2856	23,4705	23,6098	23,084	+	6	6	6	14,8	14,8	14,8	56,131	4,47E+08	20	23,5972	23,825	23,3694	10	5	5	0,00118487	-0,455507	A0A132Z360	A0A132Z360	murE	UDP-N-acetylmuramoyl-L-alanyl-D-glutamate--L-lysine ligase
24,3645	24,2991	24,3625	24,3733	24,3817	23,95	24,1216	23,9899	23,9642	23,4897	+	3	3	3	13,4	13,4	13,4	27,371	3,45E+08	20	24,1297	24,3562	23,9031	10	5	5	0,00313136	-0,453144	A0A132P986	A0A132P986	atpB	ATP synthase subunit a
27,2677	27,2931	27,2971	27,1658	27,2619	26,797	26,655	26,9134	26,893	26,7763	+	18	18	18	49,8	49,8	49,8	49,36	6,40E+09	177	27,032	27,2571	26,8069	10	5	5	2,49E-05	-0,45016	A0A132P3T0	A0A132P3T0	pepV2	Dipeptidase
27,4833	27,4108	27,4484	27,4851	27,483	27,0212	27,0338	27,0662	27,136	26,8051	+	11	11	11	51,3	51,3	51,3	30,296	9,90E+09	127	27,2373	27,4621	27,0125	10	5	5	5,08E-05	-0,449645	A0A132P5H1	A0A132P5H1	rplB	50S ribosomal protein L2
25,1853	25,0024	25,0644	25,0045	25,0951	24,7265	24,6575	24,6366	24,5144	24,5709	+	13	13	13	39,4	39,4	39,4	44,852	2,16E+09	63	24,8458	25,0704	24,6212	10	5	5	1,79E-05	-0,4492	A0A132ZG94	A0A132ZG94	cfa	Class I SAM-dependent methyltransferase
24,6206	24,5797	24,693	24,6338	24,7217	24,3938	24,0889	24,3129	24,2654	23,9436	+	6	6	6	24,3	24,3	24,3	33,134	7,58E+08	30	24,4253	24,6497	24,2009	10	5	5	0,00076567	-0,44884	A0A132P8F1	A0A132P8F1	atpG	ATP synthase gamma chain
27,0751	27,1339	27,0781	27,0303	27,1058	26,6861	26,5267	26,7461	26,6839	26,5516	+	12	12	12	41,4	41,4	41,4	34,221	5,33E+09	130	26,8618	27,0846	26,6389	10	5	5	1,03E-05	-0,445754	A0A133CEQ8	A0A133CEQ8	ldh_1	L-lactate dehydrogenase
24,3803	24,2572	24,3235	24,2322	24,3077	23,8407	23,7943	23,9829	23,8609	23,8064	+	22	22	22	37,9	37,9	37,9	77,029	1,96E+09	110	24,0786	24,3002	23,857	10	5	5	6,21E-06	-0,443145	A0A132PAI0	A0A132PAI0	pnp	Polyribonucleotide nucleotidyltransferase
27,1515	27,1013	27,2511	27,186	27,1935	26,8459	26,5975	26,8769	26,9033	26,451	+	8	8	8	62,7	62,7	62,7	10,938	3,82E+09	72	26,9558	27,1767	26,7349	10	5	5	0,00143412	-0,441747	A0A133N7B0	A0A133N7B0	rplX	50S ribosomal protein L24
27,588	27,6328	27,6334	27,6112	27,4849	27,1545	27,1534	27,2659	27,2673	26,9309	+	12	12	12	37	37	37	35,142	9,15E+09	200	27,3722	27,5901	27,1544	10	5	5	0,000191887	-0,435644	A0A133CQG4	A0A133CQG4	ldh1	L-lactate dehydrogenase
23,7892	23,7379	23,8124	23,8869	24,1044	23,4507	23,4833	23,9059	22,9	23,4209	+	8	8	8	59,1	59,1	59,1	20,373	5,13E+08	20	23,6492	23,8661	23,4322	10	5	5	0,0358143	-0,433955	A0A133CS68	A0A133CS68	nusG	Transcription termination/antitermination protein NusG
25,0825	25,0072	25,0055	24,936	24,9588	24,5754	24,5795	24,4774	24,5531	24,6433	+	13	13	13	24,5	24,5	24,5	73,877	1,70E+09	64	24,7819	24,998	24,5657	10	5	5	2,47E-06	-0,43227	A0A132ZHA7	A0A132ZHA7	thrS	Threonine--tRNA ligase
32,0143	32,0727	32,1373	32,0872	32,1064	31,6043	31,6282	31,8064	31,7399	31,5085	+	18	18	16	66,4	66,4	63,1	35,763	1,17E+11	987	31,8705	32,0836	31,6574	10	5	5	6,42E-05	-0,42614	A0A132Z6I6	A0A132Z6I6	gap	Glyceraldehyde-3-phosphate dehydrogenase
24,7074	24,6595	24,66	24,798	24,7795	24,1957	24,1387	24,2007	24,5552	24,3941	+	5	5	5	16,2	16,2	16,2	41,38	5,24E+08	20	24,5089	24,7209	24,2969	10	5	5	0,000918913	-0,424012	A0A132ZDD8	A0A132ZDD8	nagA	N-acetylglucosamine-6-phosphate deacetylase
24,4879	24,3936	24,5377	24,4948	24,6819	24,1137	24,0786	24,3697	24,211	23,7102	+	7	7	7	20,5	20,5	20,5	49,258	6,70E+08	23	24,3079	24,5192	24,0966	10	5	5	0,00740275	-0,422539	A0A133CQN0	A0A133CQN0	hisS	Histidine--tRNA ligase
27,1904	27,0912	27,2048	27,014	27,1764	26,7956	26,6514	26,7733	26,7634	26,5888	+	8	8	8	52,2	52,2	52,2	21,945	4,85E+09	86	26,9249	27,1354	26,7145	10	5	5	5,30E-05	-0,420846	A0A132Z3K4	A0A132Z3K4	rplY	50S ribosomal protein L25
24,5389	24,3907	24,3783	24,2722	24,2839	24,0053	23,9551	23,9767	24,0294	23,7949	+	5	5	5	23,3	23,3	23,3	33,39	5,59E+08	36	24,1626	24,3728	23,9523	10	5	5	0,000161687	-0,420509	A0A132ZHC7	A0A132ZHC7	fabD	Malonyl CoA-acyl carrier protein transacylase
26,4737	26,3678	26,4015	26,3062	26,4537	26,012	25,9713	26,0055	26,071	25,8439	+	11	11	11	22	22	22	51,863	3,34E+09	71	26,1907	26,4006	25,9807	10	5	5	2,40E-05	-0,419862	A0A132Z266	A0A132Z266	pepV	Dipeptidase PepV
22,6037	22,8516	22,7406	22,7573	22,7611	22,4351	22,3822	22,3546	22,2485	22,1962	+	5	5	5	21,1	21,1	21,1	32,472	2,15E+08	19	22,5331	22,7428	22,3233	10	5	5	0,000105351	-0,419506	A0A133CI75	A0A133CI75	accD	Acetyl-coenzyme A carboxylase carboxyl transferase subunit beta
24,2038	24,1942	24,1283	24,1032	24,2273	23,8226	23,8635	23,8294	23,7343	23,5186	+	14	14	14	25,2	25,2	25,2	78,601	1,18E+09	64	23,9625	24,1714	23,7537	10	5	5	0,000246446	-0,417693	A0A132Z1H7	A0A132Z1H7	glyS	Glycine--tRNA ligase beta subunit
24,2113	24,0186	24,2215	24,1017	23,9587	23,677	23,6185	23,8041	23,7166	23,6122	+	7	7	7	26,7	26,7	26,7	35,766	6,31E+08	26	23,894	24,1024	23,6857	10	5	5	0,000161904	-0,416666	A0A132Z7J1	A0A132Z7J1	plsX_2	Phosphate acyltransferase
23,2651	23,2737	23,3687	23,2156	22,8659	22,7341	22,5873	22,7261	23,1846	22,6801	+	4	4	4	23,5	23,5	23,5	28,364	2,12E+08	18	22,9901	23,1978	22,7824	10	5	5	0,0153196	-0,415354	A0A132YYV5	A0A132YYV5	proC	Pyrroline-5-carboxylate reductase
27,3888	27,251	27,4485	27,4053	27,3275	27,0806	26,7322	26,951	27,176	26,8207	+	10	10	10	56	56	56	16,862	2,85E+09	57	27,1581	27,3642	26,9521	10	5	5	0,00160521	-0,412089	A0A132P5Q2	A0A132P5Q2	rplI	50S ribosomal protein L9
24,5437	24,5281	24,3498	24,3967	24,5536	23,9794	24,1271	23,9876	24,1718	24,0485	+	5	5	5	13,3	13,3	13,3	50,047	5,73E+08	24	24,2686	24,4744	24,0629	10	5	5	8,77E-05	-0,41149	A0A132ZG70	A0A132ZG70	cfiB_2	Biotin carboxylase
27,773	27,5876	27,6368	27,5964	27,6517	27,2538	27,1871	27,2559	27,362	27,1605	+	20	20	20	54,3	54,3	54,3	52,684	8,56E+09	217	27,4465	27,6491	27,2439	10	5	5	3,05E-05	-0,405235	A0A132ZCB2	A0A132ZCB2	gnd	6-phosphogluconate dehydrogenase, decarboxylating
28,54	28,4118	28,4827	28,3986	28,5187	28,0769	27,961	28,0492	28,2017	28,0508	+	14	12	12	30,1	26,8	26,8	36,156	9,00E+09	161	28,2691	28,4703	28,0679	10	5	5	3,09E-05	-0,402425	A0A133CK87	A0A133CK87	gapA_1	Glyceraldehyde-3-phosphate dehydrogenase
24,9931	24,9923	25,0847	24,9186	25,0119	24,5502	24,6218	24,579	24,7536	24,4892	+	9	9	9	37,3	37,3	37,3	39,472	1,09E+09	39	24,7994	25,0001	24,5987	10	5	5	5,36E-05	-0,401361	A0A133CV71	A0A133CV71	pepA	Glutamyl aminopeptidase
24,4597	24,4159	24,417	24,3755	24,5862	24,0234	23,9937	24,0856	24,1582	23,9907	+	12	12	12	36,8	36,8	36,8	54,264	7,98E+08	29	24,2506	24,4509	24,0503	10	5	5	3,41E-05	-0,400555	A0A132P631	A0A132P631	cysS	Cysteine--tRNA ligase
24,4225	24,5129	24,3945	24,2771	24,5505	23,9631	23,8631	24,1091	24,3058	23,9354	+	12	12	12	17	17	17	98,343	1,20E+09	57	24,2334	24,4315	24,0353	10	5	5	0,00260575	-0,396199	A0A132Z061	A0A132Z061	alaS_1	Alanine--tRNA ligase
24,3066	24,3776	24,3473	24,3024	24,5601	24,3061	24,2284	23,794	23,7712	23,8153	+	3	3	3	28,8	28,8	28,8	17,023	4,13E+08	22	24,1809	24,3788	23,983	10	5	5	0,0138486	-0,395797	A0A132P674	A0A132P674	yugI_1	RNA-binding protein S1
25,295	25,0138	25,2178	25,0944	25,0651	24,7886	24,4948	24,6853	25,1706	24,5738	+	4	4	4	35,3	35,3	35,3	13,622	9,11E+08	26	24,9399	25,1372	24,7426	10	5	5	0,0155292	-0,394618	A0A132P515	A0A132P515	rplT1	50S ribosomal protein L20
24,7089	24,5278	24,6536	24,5942	24,725	24,2572	24,2193	24,2631	24,4651	24,0388	+	9	9	9	35,6	35,6	35,6	33,783	1,01E+09	61	24,4453	24,6419	24,2487	10	5	5	0,000929233	-0,393199	A0A133CQB1	A0A133CQB1	glcK	Glucokinase
25,5611	25,4016	25,6422	25,4889	25,6556	25,1939	25,0545	25,1644	25,3352	25,0395	+	11	11	11	37,8	37,8	37,8	35,273	2,37E+09	85	25,3537	25,5499	25,1575	10	5	5	0,00059618	-0,392365	A0A133CQ76	A0A133CQ76	prs1	Ribose-phosphate pyrophosphokinase
22,8972	22,9158	22,7671	22,7255	22,8524	22,483	22,3656	22,76	22,3254	22,2682	+	8	8	8	22,6	22,6	22,6	47,714	3,40E+08	23	22,636	22,8316	22,4404	10	5	5	0,00331292	-0,391164	A0A132P5N4	A0A132P5N4	purA	Adenylosuccinate synthetase
27,3687	26,9842	27,187	26,949	27,0472	26,7582	26,5584	26,6377	27,1336	26,5231	+	7	7	7	54	54	54	14,314	4,59E+09	129	26,9147	27,1072	26,7222	10	5	5	0,0211724	-0,385019	A0A132ZIW6	A0A132ZIW6	rplQ	50S ribosomal protein L17
23,9968	23,9542	23,7448	23,8019	23,7853	23,6202	23,298	23,4335	23,6571	23,3504	+	4	4	4	27,2	27,2	27,2	27,635	3,56E+08	17	23,6642	23,8566	23,4718	10	5	5	0,00226919	-0,384756	A0A133CZL4	A0A133CZL4	macB_2	ABC transporter ATP-binding protein
27,9898	27,8171	27,8923	27,6529	27,8425	27,4295	27,3537	27,5035	27,7159	27,2691	+	6	6	6	47,8	47,8	47,8	19,221	7,07E+09	100	27,6466	27,8389	27,4544	10	5	5	0,00347547	-0,384545	A0A132ZIW7	A0A132ZIW7	rplF	50S ribosomal protein L6
25,118	25,2855	25,3635	25,2122	25,3113	25,0999	24,8844	24,9534	24,9441	24,5127	+	5	5	5	21,6	21,6	21,6	38,497	1,12E+09	33	25,0685	25,2581	24,8789	10	5	5	0,00759357	-0,379228	A0A132Z6I9	A0A132Z6I9	rmlB	dTDP-glucose 4,6-dehydratase
26,2155	25,8956	25,9899	25,675	25,8512	25,5824	25,5111	25,4841	25,7956	25,3609	+	9	9	9	26,6	26,6	26,6	50,535	2,31E+09	65	25,7361	25,9254	25,5468	10	5	5	0,0105632	-0,378604	A0A132Z6F6	A0A132Z6F6	npr_1	FAD-dependent oxidoreductase
26,3155	26,2441	26,2183	26,2075	26,2489	25,7993	25,756	25,9127	26,0529	25,8247	+	16	16	16	40,2	40,2	40,2	44,333	4,11E+09	115	26,058	26,2469	25,8691	10	5	5	0,000143209	-0,377735	A0A132Z7D2	A0A132Z7D2	ftsZ	Cell division protein FtsZ
29,6619	29,6536	29,6312	29,4705	29,5949	29,1766	29,1673	29,386	29,1864	29,2189	+	33	33	33	56,9	56,9	56,9	63,647	4,33E+10	564	29,4147	29,6024	29,227	10	5	5	0,000112723	-0,375409	A0A132YXT5	A0A132YXT5	pyk	Pyruvate kinase
26,5899	26,5096	26,5152	26,4393	26,5909	26,1284	26,1521	26,2069	26,0971	26,2035	+	13	13	13	19,2	19,2	19,2	77,593	2,39E+09	84	26,3433	26,529	26,1576	10	5	5	6,09E-06	-0,371372	A0A132P6D8	A0A132P6D8	hflB	ATP-dependent zinc metalloprotease FtsH
29,532	29,6104	29,5324	29,4891	29,4613	29,125	29,0427	29,2336	29,3018	29,0708	+	30	30	30	71,5	71,5	71,5	46,511	7,78E+10	1034	29,3399	29,525	29,1548	10	5	5	0,000152705	-0,370248	A0A133CMW8	A0A133CMW8	eno	Enolase
25,5075	25,2859	25,3666	25,3773	25,5117	24,9053	25,0492	25,1915	25,2404	24,8169	+	7	7	7	28,9	28,9	28,9	33,481	1,46E+09	45	25,2252	25,4098	25,0407	10	5	5	0,00388159	-0,369167	A0A133CPF0	A0A133CPF0	ppaC_1	Probable manganese-dependent inorganic pyrophosphatase
25,3333	25,1637	25,2043	25,2078	25,2956	24,8201	24,8581	25,0726	24,6896	24,9228	+	14	14	14	28,2	28,2	28,2	68,056	2,02E+09	78	25,0568	25,241	24,8726	10	5	5	0,00078588	-0,368302	A0A132Z103	A0A132Z103	tufA3	50S ribosomal subunit assembly factor BipA
23,2636	23,2698	23,3343	22,8742	23,3586	22,9559	22,7456	23,0159	23,0645	22,5285	+	6	6	6	14,8	14,8	14,8	72,109	3,17E+08	20	23,0411	23,2201	22,8621	10	5	5	0,0275381	-0,358038	A0A133CM53	A0A133CM53	pepO	M13 family peptidase
29,4918	29,3994	29,3745	29,2871	29,4286	28,7946	28,8548	29,1225	29,1912	29,2404	+	35	35	35	59,9	59,9	59,9	65,683	2,33E+10	433	29,2185	29,3963	29,0407	10	5	5	0,00623704	-0,355555	A0A132P9J8	A0A132P9J8	dnaK	Chaperone protein DnaK
24,4465	24,4173	24,5178	24,5369	24,6897	24,0868	24,1197	24,2611	24,4843	23,931	+	10	10	10	55,8	55,8	55,8	23,771	1,30E+09	84	24,3491	24,5216	24,1766	10	5	5	0,0108329	-0,345061	A0A132P5H0	A0A132P5H0	adk_2	Adenylate kinase
26,0209	25,5134	25,6532	25,6103	25,6831	25,529	25,3673	25,3244	25,2749	25,2796	+	8	8	8	19,5	19,5	19,5	48,737	1,08E+09	37	25,5256	25,6962	25,355	10	5	5	0,00827132	-0,341127	A0A132Z8U4	A0A132Z8U4	glmM_1	Phosphoglucosamine mutase
28,9452	29,1824	29,2178	29,1334	29,1494	28,825	28,765	28,8613	28,8297	28,6611	+	18	18	18	58,7	58,7	58,7	49,689	1,58E+10	179	28,957	29,1256	28,7884	10	5	5	0,00045434	-0,337238	A0A133CQG6	A0A133CQG6	pgi_1	Glucose-6-phosphate isomerase
24,1524	23,8117	23,682	23,741	24,1214	23,1748	23,7393	23,5205	23,661	23,7278	+	5	5	5	12,5	12,5	12,5	61,142	3,26E+08	17	23,7332	23,9017	23,5647	10	5	5	0,0471737	-0,337006	A0A133CGU9	A0A133CGU9	rnjA	Ribonuclease J
25,066	25,2061	25,2693	25,2371	25,253	24,8813	24,8439	24,9078	25,0349	24,6884	+	10	10	10	42,5	42,5	42,5	32,572	1,66E+09	58	25,0388	25,2063	24,8713	10	5	5	0,00103101	-0,335014	A0A132P296	A0A132P296	cysK	Cysteine synthase
23,458	23,44	23,5861	23,4728	23,5912	23,3237	23,0323	23,2671	23,2107	23,043	+	10	10	10	20,4	20,4	20,4	69,702	5,64E+08	35	23,3425	23,5096	23,1754	10	5	5	0,00111449	-0,33424	A0A132ZEU6	A0A132ZEU6	pepF	M03 family oligopeptidase F
24,3449	24,6386	24,6981	24,7972	24,8287	24,3106	24,3328	24,4285	24,5044	24,0687	+	10	10	10	47,8	47,8	47,8	22,931	1,60E+09	75	24,4953	24,6615	24,329	10	5	5	0,018952	-0,332457	A0A132P446	A0A132P446	upp	Uracil phosphoribosyltransferase
28,1695	28,054	28,0749	28,0271	27,9944	27,8201	27,6449	27,73	27,9668	27,5096	+	13	13	13	69,9	69,9	69,9	17,477	9,26E+09	203	27,8991	28,064	27,7343	10	5	5	0,00408133	-0,329693	A0A0M1PHU0	A0A0M1PHU0	rpsE_2	30S ribosomal protein S5
23,952	24,0322	24,0374	23,9813	24,1219	23,6743	23,7682	23,7593	23,6797	23,6084	+	11	11	11	25,3	25,3	25,3	66,672	8,02E+08	70	23,8615	24,025	23,698	10	5	5	4,88E-05	-0,326983	A0A132Z0T9	A0A132Z0T9	aspS	Aspartate--tRNA ligase
24,4342	24,6	24,6834	24,6443	24,7302	24,2171	24,3707	24,3042	24,3819	24,1896	+	12	12	12	41	41	41	45,154	8,50E+08	69	24,4556	24,6184	24,2927	10	5	5	0,000952847	-0,325732	A0A133CIZ6	A0A133CIZ6	pepS	Aminopeptidase
23,2434	23,1138	23,2852	23,1498	23,5069	22,8875	22,8913	22,8659	23,1751	22,8683	+	10	10	10	23,5	23,5	23,5	55,633	4,23E+08	23	23,0987	23,2598	22,9376	10	5	5	0,00770128	-0,322221	A0A133CPE5	A0A133CPE5	pncB	Nicotinate phosphoribosyltransferase
24,0895	24,0508	24,1661	24,0683	24,1188	23,9702	23,6857	23,8891	23,8859	23,458	+	6	6	6	37	37	37	24,683	6,52E+08	42	23,9382	24,0987	23,7778	10	5	5	0,00961134	-0,320915	A0A132Z300	A0A132Z300	mtnN	5-methylthioadenosine/S-adenosylhomocysteine nucleosidase
28,3435	28,1791	28,1832	28,1612	28,2525	27,7602	27,8617	28,0467	28,0491	27,8284	+	15	15	15	41,8	41,8	41,8	44,823	5,32E+09	108	28,0666	28,2239	27,9092	10	5	5	0,00167856	-0,314666	A0A133CIR2	A0A133CIR2	rpsA	30S ribosomal protein S1
27,2136	27,0111	27,1714	27,042	27,0973	26,7847	26,7248	26,995	26,868	26,5966	+	13	13	13	37,7	37,7	37,7	35,661	5,93E+09	106	26,9504	27,1071	26,7938	10	5	5	0,00360554	-0,313258	A0A133CSS3	A0A133CSS3	manX	EIIAB-Man
23,5135	23,4924	23,6484	23,432	23,6949	23,3491	23,4305	23,2277	23,2932	22,916	+	5	5	5	11,5	11,5	11,5	49,6	3,07E+08	16	23,3998	23,5562	23,2433	10	5	5	0,0148843	-0,312941	A0A132P9Q4	A0A132P9Q4	murC	UDP-N-acetylmuramate--L-alanine ligase
29,1806	28,9598	29,0655	29,035	29,1197	28,754	28,699	28,8331	28,9186	28,622	+	20	20	20	59,1	59,1	59,1	42,171	3,03E+10	402	28,9187	29,0721	28,7653	10	5	5	0,00133401	-0,30676	A0A132Z539	A0A132Z539	pgk	Phosphoglycerate kinase
27,3649	27,2858	27,2244	27,0519	27,227	26,8431	26,8212	27,0226	26,8991	27,0383	+	32	32	30	48,3	48,3	46,2	83,394	5,99E+09	192	27,0778	27,2308	26,9249	10	5	5	0,00207785	-0,305924	A0A132ZG15	A0A132ZG15	AWT83_1481	AAA domain-containing protein
22,0037	22,18	22,0587	22,0862	22,0318	21,9273	21,7187	21,6727	21,9268	21,619	+	4	4	4	14,4	14,4	14,4	40,897	9,22E+07	8	21,9225	22,0721	21,7729	10	5	5	0,00308613	-0,299188	A0A132P2H9	A0A132P2H9	pdhA	Pyruvate dehydrogenase E1 component subunit alpha
24,7638	24,6139	24,8053	24,4892	24,4373	24,4131	24,2005	24,3357	24,5813	24,0995	+	6	6	6	31,8	31,8	31,8	32,384	7,80E+08	21	24,474	24,6219	24,326	10	5	5	0,028314	-0,29588	A0A132Z6Y8	A0A132Z6Y8	rfbD	dTDP-4-dehydrorhamnose reductase
22,9743	22,8382	22,8097	22,9267	23,0248	22,6343	22,5064	22,7358	22,714	22,5192	+	6	6	6	19	19	19	35,761	2,46E+08	23	22,7683	22,9147	22,6219	10	5	5	0,00157549	-0,292807	A0A132Z0H4	A0A132Z0H4	ppsC	Alcohol dehydrogenase
29,3836	29,415	29,5526	29,518	29,3779	29,2135	29,1207	29,1296	29,4673	28,8692	+	7	7	7	57,7	57,7	57,7	14,344	1,08E+10	149	29,3047	29,4494	29,1601	10	5	5	0,0224344	-0,289347	A0A132P6U0	A0A132P6U0	rpsI	30S ribosomal protein S9
25,27	25,2864	25,2809	25,3378	25,3105	24,9981	24,9149	24,9913	25,2821	24,9594	+	8	8	8	40,6	40,6	40,6	26,863	1,51E+09	38	25,1631	25,2971	25,0291	10	5	5	0,00364523	-0,267978	A0A133CT31	A0A133CT31	AWT83_1408	Uncharacterized protein
29,712	29,7619	29,7763	29,7689	29,7525	29,4647	29,4265	29,6008	29,6119	29,3521	+	8	8	8	40,5	40,5	40,5	30,752	2,04E+10	145	29,6228	29,7543	29,4912	10	5	5	0,000937409	-0,263104	A0A132Z6Z1	A0A132Z6Z1	fba_1	Fructose-1,6-bisphosphate aldolase, class II
23,7218	23,7153	23,7642	23,7216	23,6432	23,1702	23,499	23,4764	23,7989	23,3063	+	11	11	11	46,3	46,3	46,3	35,451	6,72E+08	36	23,5817	23,7132	23,4502	10	5	5	0,0402096	-0,263055	A0A132Z0C1	A0A132Z0C1	iolS_1	Aldo/keto reductase
24,4553	24,3842	24,3721	24,1834	24,1072	23,9581	23,945	24,2589	23,8743	24,1522	+	15	15	15	18	18	18	106,76	1,07E+09	67	24,1691	24,3004	24,0377	10	5	5	0,0275225	-0,262727	A0A132Z792	A0A132Z792	ileS	Isoleucine--tRNA ligase
23,8189	23,581	23,4702	23,6499	23,7945	23,4446	23,5391	23,3685	23,4629	23,1884	+	6	6	6	19,8	19,8	19,8	40,246	3,08E+08	24	23,5318	23,6629	23,4007	10	5	5	0,0180863	-0,262206	Q47755	Q47755	ddl	D-alanine--D-alanine ligase
29,2173	29,1666	29,2373	29,0532	29,0365	28,749	28,8195	29,0849	28,9407	28,8378	+	28	28	25	68,6	68,6	62,5	43,224	4,24E+10	525	29,0143	29,1422	28,8864	10	5	5	0,00724667	-0,255803	A0A133CTY1	A0A133CTY1	tufA	Elongation factor Tu
23,5085	23,341	23,4571	23,2226	23,4246	23,0779	23,1061	23,3798	23,0793	23,034	+	4	4	4	15	15	15	39,695	2,71E+08	14	23,2631	23,3907	23,1354	10	5	5	0,0126504	-0,255321	A0A132P6N6	A0A132P6N6	pepA2	M42 family metallopeptidase
29,0894	28,9371	29,0507	28,983	28,975	28,8401	28,7023	28,8723	28,8506	28,513	+	13	13	13	53,2	53,2	53,2	24,348	1,69E+10	170	28,8814	29,0071	28,7557	10	5	5	0,00878908	-0,25139	A0A132ZIX8	A0A132ZIX8	rpsC	30S ribosomal protein S3
26,3305	26,1368	26,3025	26,2554	26,3463	26,1232	25,8912	26,1713	25,9337	26,0263	+	19	19	19	36,7	36,7	36,7	63,929	4,56E+09	109	26,1517	26,2743	26,0291	10	5	5	0,00564934	-0,24516	A0A133CFJ8	A0A133CFJ8	DTPHA_6018	Phospho-sugar mutase
28,4512	28,4424	28,4753	28,3649	28,3391	28,1158	28,1582	28,2616	28,0828	28,2557	+	35	35	35	57,6	57,6	57,6	76,71	2,35E+10	395	28,2947	28,4146	28,1748	10	5	5	0,000691848	-0,239729	A0A133CU87	A0A133CU87	fusA_2	Elongation factor G
26,3069	26,4279	26,4815	26,3345	26,5018	26,1667	26,1167	26,284	26,299	25,9928	+	21	21	21	42,8	42,8	42,8	57,129	3,36E+09	130	26,2912	26,4105	26,1719	10	5	5	0,00832561	-0,238663	A0A132ZJ97	A0A132ZJ97	lysS	Lysine--tRNA ligase
25,5564	25,7987	25,7037	25,8281	25,6415	25,56	25,5906	25,4395	25,5787	25,1931	+	5	5	5	34,2	34,2	34,2	20,984	1,18E+09	23	25,589	25,7057	25,4724	10	5	5	0,0320263	-0,233303	A0A132P7B1	A0A132P7B1	efp	Elongation factor P
22,98	22,8154	22,9272	23,2206	22,9485	22,6953	22,6924	22,8045	22,8475	22,6983	+	2	2	2	7,7	7,7	7,7	46,704	1,34E+08	9	22,863	22,9784	22,7476	10	5	5	0,014466	-0,230737	A0A132PAC8	A0A132PAC8	pyrC_1	Dihydroorotase
28,5476	28,8519	28,7706	28,8026	28,9366	28,5686	28,5021	28,6425	28,5711	28,4835	+	17	17	17	35,7	35,7	35,7	56,428	1,59E+10	252	28,6677	28,7818	28,5536	10	5	5	0,0121761	-0,228275	A0A133CTP7	A0A133CTP7	atpA	ATP synthase subunit alpha
26,8339	26,6539	26,8489	26,6999	26,7178	26,5308	26,4141	26,6663	26,7567	26,2625	+	12	12	12	77,4	77,4	77,4	17,371	4,78E+09	116	26,6385	26,7509	26,5261	10	5	5	0,0473869	-0,224784	A0A132P4S0	A0A132P4S0	uspA4	Universal stress protein
27,2963	27,2216	27,5047	27,2672	27,4276	27,1308	26,9609	27,2348	27,2421	27,0319	+	10	10	10	51,8	51,8	51,8	27,033	6,59E+09	81	27,2318	27,3435	27,1201	10	5	5	0,0193559	-0,223386	A0A133CMP5	A0A133CMP5	tpiA	Triosephosphate isomerase
24,9756	24,9269	25,0246	25,0493	24,9088	24,7829	24,6797	24,7799	25,0401	24,4977	+	3	3	3	19	19	19	20,45	8,57E+08	38	24,8665	24,977	24,7561	10	5	5	0,0429865	-0,220992	A0A132P8Q5	A0A132P8Q5	azr	Flavin reductase
26,0927	26,1193	26,0829	25,8288	26,0972	25,7029	25,8049	25,8682	25,9204	25,8266	+	27	25	25	42,3	40,6	40,6	97,933	2,92E+09	113	25,9344	26,0442	25,8246	10	5	5	0,0098078	-0,219567	A0A132YYS0	A0A132YYS0	clpB	Chaperone protein ClpB
22,219	22,1465	22,5451	22,2661	22,3354	22,2159	22,031	22,0739	22,1086	22,0271	+	2	2	2	11	11	11	19,541	9,05E+07	10	22,1969	22,3024	22,0913	10	5	5	0,0244286	-0,211126	A0A132PB85	A0A132PB85	ywlG	UPF0340 protein AS238_13075

26,0357	25,9139	25,8203	25,8433	25,957	25,7082	25,5883	25,7279	25,7366	25,7771	+	19	19	19	39,6	39,6	39,6	63,958	2,62E+09	122	25,8108	25,914	25,7076	10	5	5	0,00344642	-0,206416	A0A132Z4F9	A0A132Z4F9	argS	Arginine--tRNA ligase
28,0365	28,0792	28,1594	28,105	28,023	27,8499	27,728	28,0154	28,0582	27,7312	+	15	15	15	43,6	43,6	43,6	51,057	9,87E+09	216	27,9786	28,0806	27,8765	10	5	5	0,0241541	-0,204096	A0A5P3FTW7	A0A5P3FTW7	atpD	ATP synthase subunit beta
27,3751	27,3953	27,4311	27,3978	27,4314	27,0772	27,0919	27,3136	27,2142	27,3154	+	16	16	16	39,3	39,3	39,3	63,291	7,35E+09	179	27,3043	27,4061	27,2025	10	5	5	0,00478658	-0,203637	A0A132ZFW2	A0A132ZFW2	ptsI	Phosphoenolpyruvate-protein phosphotransferase
24,512	24,532	24,5866	24,5636	24,6137	24,1746	24,302	24,4964	24,3862	24,4324	+	8	8	8	30	30	30	49,339	8,47E+08	40	24,4599	24,5616	24,3583	10	5	5	0,00853956	-0,203289	A0A132YYW9	A0A132YYW9	glmU_1	Bifunctional protein GlmU
27,212	27,1665	27,3009	27,2427	27,1128	27,0771	27,0377	27,0905	27,0797	26,831	+	13	13	13	45	45	45	34,211	7,79E+09	182	27,1151	27,207	27,0232	10	5	5	0,0137666	-0,183764	A0A132YXU1	A0A132YXU1	pfkA	ATP-dependent 6-phosphofructokinase
24,86	24,9908	25,0723	24,9458	24,928	24,8444	24,808	24,8834	24,6835	24,7009	+	16	16	16	32,5	32,5	32,5	64,542	1,04E+09	39	24,8717	24,9594	24,7841	10	5	5	0,0106022	-0,175305	A0A132Z3U0	A0A132Z3U0	proS	Proline--tRNA ligase
25,0126	25,0189	25,068	24,9257	25,1309	24,977	24,8363	24,9127	24,8882	24,8035	+	8	8	8	21,6	21,6	21,6	49,677	8,12E+08	55	24,9574	25,0312	24,8835	10	5	5	0,0116056	-0,147708	A0A133CIV0	A0A133CIV0	murD	UDP-N-acetylmuramoylalanine--D-glutamate ligase
26,4265	26,4885	26,4982	26,4787	26,4298	26,3619	26,2386	26,3454	26,3945	26,2434	+	14	14	14	36,8	36,8	36,8	53,012	1,66E+09	66	26,3905	26,4643	26,3168	10	5	5	0,0030924	-0,147554	A0A132Z8H7	A0A132Z8H7	guaB	Inosine-5-monophosphate dehydrogenase
23,0316	23,0858	22,9607	23,0411	23,0366	22,9103	22,8124	22,9589	23,0056	22,7967	+	2	2	2	10,8	10,8	10,8	34,901	1,54E+08	10	22,964	23,0312	22,8968	10	5	5	0,0180101	-0,134414	A0A133CRL5	A0A133CRL5	pyrDA	Dihydroorotate dehydrogenase
26,5115	26,6063	26,6266	26,5489	26,4048	26,8247	26,7621	26,7052	26,7794	26,6067	+	19	19	19	43,2	43,2	43,2	57,877	5,90E+09	212	26,6376	26,5396	26,7356	10	5	5	0,00694239	0,195996	A0A133CL60	A0A133CL60	guaA	GMP synthase [glutamine-hydrolyzing]
27,3376	27,2569	27,3151	27,2302	27,3186	27,7753	27,4396	27,3407	27,4781	27,4192	+	15	15	15	43,9	43,9	43,9	35,17	4,69E+09	115	27,3911	27,2917	27,4906	10	5	5	0,033098	0,198898	A0A132ZIV5	A0A132ZIV5	rpoA	DNA-directed RNA polymerase subunit alpha
24,0252	23,6605	23,7126	23,774	23,7777	24,0374	23,9546	24,1427	24,1139	23,9071	+	2	2	2	8,8	8,8	8,8	36,648	2,33E+08	17	23,9106	23,79	24,0312	10	5	5	0,0141211	0,241151	A0A132ZCT4	A0A132ZCT4	galE3	UDP-glucose 4-epimerase
29,6619	29,3093	29,7141	29,4381	29,5176	29,9023	29,6361	29,7302	29,9936	29,8836	+	10	10	10	66,7	66,7	66,7	11,695	1,85E+10	187	29,6787	29,5282	29,8291	10	5	5	0,0151256	0,300937	A0A133CTY8	A0A133CTY8	rpsJ	30S ribosomal protein S10
24,1576	24,3938	24,2861	24,0074	24,1491	24,6189	24,846	24,4526	24,3065	24,4947	+	4	4	4	13,5	13,5	13,5	47,949	2,78E+08	23	24,3713	24,1988	24,5438	10	5	5	0,0150567	0,344951	A0A132Z6L6	A0A132Z6L6	obg_2	GTPase Obg
25,8822	25,8639	25,5206	25,8566	25,9007	26,2023	26,1901	26,0716	26,1254	26,1626	+	2	2	2	5,8	5,8	5,8	31,376	6,39E+08	9	25,9776	25,8048	26,1504	10	5	5	0,0017775	0,345593	A0A132YYV0	A0A132YYV0	mutM	Formamidopyrimidine-DNA glycosylase
27,3179	27,3707	27,1734	27,341	27,3399	27,4425	27,5838	27,7229	27,8266	27,7605	+	15	15	15	34,2	34,2	34,2	55,245	7,62E+09	152	27,4879	27,3086	27,6673	10	5	5	0,00164289	0,358643	A0A132P622	A0A132P622	gltX	Glutamate--tRNA ligase
25,1316	24,9091	25,0886	25,0742	25,0916	25,2245	25,2878	25,5867	25,6342	25,3815	+	3	3	3	37,8	37,8	37,8	8,5634	7,35E+08	52	25,241	25,059	25,4229	10	5	5	0,00363456	0,36393	A0A133CHI6	A0A133CHI6	acpP2	Acyl carrier protein
24,3349	23,6899	24,1749	24,0104	24,3028	24,6768	24,6221	24,4149	24,6784	24,296	+	3	3	3	47,2	47,2	47,2	8,2495	4,05E+08	26	24,3201	24,1026	24,5376	10	5	5	0,015017	0,435045	A0A0M1PHU6	A0A0M1PHU6	infA	Translation initiation factor IF-1
26,4682	26,4362	26,3531	26,2604	26,3815	26,7283	27,0383	26,9147	26,536	26,9513	+	44	44	44	37,7	37,7	37,7	136,91	7,90E+09	303	26,6068	26,3799	26,8337	10	5	5	0,00157785	0,453839	A0A133CUF4	A0A133CUF4	rpoC	DNA-directed RNA polymerase subunit beta
27,4695	27,2124	27,2547	27,3789	26,99	27,7086	27,9211	27,4305	27,9509	27,6328	+	5	5	5	40,1	40,1	40,1	15,215	4,06E+09	72	27,4949	27,2611	27,7288	10	5	5	0,0059704	0,467681	A0A0M1PG56	A0A0M1PG56	rpsL	30S ribosomal protein S12
24,3465	24,2737	24,2424	24,2434	23,8731	24,7326	24,8121	24,7415	24,4527	24,7129	+	17	17	17	30,7	30,7	30,7	72,545	9,37E+08	67	24,4431	24,1958	24,6904	10	5	5	0,00138115	0,494548	A0A132P5R2	A0A132P5R2	gyrB	DNA gyrase subunit B
26,9047	26,431	26,8171	26,6315	26,9641	27,3254	27,2111	27,2566	27,3873	27,0493	+	4	4	4	32,1	32,1	32,1	9,1904	2,13E+09	29	26,9978	26,7497	27,246	10	5	5	0,00234002	0,496278	A0A132P2F6	A0A132P2F6	rpsT_1	30S ribosomal protein S20
25,3428	25,1627	25,2512	25,0562	25,3665	25,4159	25,6258	25,8288	25,8763	26,0184	+	6	6	6	21,2	21,2	21,2	33,684	1,02E+09	37	25,4945	25,2359	25,753	10	5	5	0,00257157	0,517151	A0A133CUL3	A0A133CUL3	cad	FMN-binding domain-containing protein
25,3714	25,0577	25,0812	25,0471	25,0638	25,7265	25,9804	25,5839	25,4012	25,5351	+	17	17	17	22,8	22,8	22,8	101,76	2,21E+09	101	25,3848	25,1242	25,6454	10	5	5	0,00207149	0,521177	A0A132Z880	A0A132Z880	valS_2	Valine--tRNA ligase
26,7345	26,938	26,7981	26,9757	26,597	27,1032	27,4687	27,5298	27,3881	27,4416	+	10	10	10	30,1	30,1	30,1	40,438	3,14E+09	72	27,0975	26,8087	27,3863	10	5	5	0,000456158	0,577595	A0A132Z8I6	A0A132Z8I6	ychF	Ribosome-binding ATPase YchF
28,0877	27,9091	28,1525	27,9663	27,8461	28,7665	28,6693	28,6105	28,3172	28,4978	+	10	10	10	52,4	52,4	52,4	16,321	8,92E+09	186	28,2823	27,9924	28,5722	10	5	5	0,000301387	0,579896	A0A132P5U7	A0A132P5U7	rplM	50S ribosomal protein L13
28,685	28,0528	28,1235	28,3147	27,9459	28,8601	28,875	28,8704	29,0174	28,5908	+	6	6	6	52,8	52,8	52,8	10,622	7,05E+09	127	28,5336	28,2244	28,8427	10	5	5	0,00299767	0,618367	A0A133CU41	A0A133CU41	rpsO	30S ribosomal protein S15
23,1451	22,7855	22,98	22,5664	22,9325	23,6495	23,6778	23,6726	23,2798	23,4118	+	4	4	4	8	8	8	60,482	2,50E+08	21	23,2101	22,8819	23,5383	10	5	5	0,000857708	0,656397	A0A132Z4K5	A0A132Z4K5	prfC	Peptide chain release factor 3
25,6428	25,2	25,2679	25,2851	25,4345	26,1599	26,0483	25,8549	26,1217	26,0066	+	4	4	4	18,3	18,3	18,3	24,377	6,51E+08	16	25,7022	25,3661	26,0383	10	5	5	0,000106502	0,672213	A0A132Z1Y7	A0A132Z1Y7	rex1	Redox-sensing transcriptional repressor Rex
30,1005	29,119	29,9524	29,5528	30,0889	30,5922	30,3714	30,5328	30,69	30,2434	+	6	6	6	64,8	64,8	64,8	9,682	1,64E+10	141	30,1243	29,7627	30,486	10	5	5	0,0077904	0,723243	A0A0M1PD93	A0A0M1PD93	hupA_1	DNA-binding protein HU
22,5065	22,2897	22,1944	22,1257	22,2636	23,1609	23,2953	22,837	22,5719	23,176	+	4	4	4	6,3	6,3	6,3	76,129	1,78E+08	15	22,6421	22,276	23,0082	10	5	5	0,00111076	0,732206	A0A132Z1G7	A0A132Z1G7	uvrB	UvrABC system protein B
27,2332	26,9478	27,1472	27,0568	26,672	28,2063	27,2816	27,2665	28,1222	28,0558	+	5	5	5	16,2	16,2	16,2	31,941	1,97E+09	35	27,399	27,0114	27,7865	10	5	5	0,0101997	0,775101	Q49SF3	Q49SF3	AWT83_1198	KR domain-containing protein
23,3757	23,0781	23,0243	23,0382	22,8779	24,2299	23,8281	23,8111	23,6072	23,8222	+	7	7	7	14,3	14,3	14,3	65,835	2,83E+08	19	23,4693	23,0788	23,8597	10	5	5	0,000323375	0,780874	A0A132Z8C6	A0A132Z8C6	glmS	Glutamine--fructose-6-phosphate aminotransferase [isomerizing]
27,5525	27,4088	27,4503	27,5943	27,3225	28,2841	28,334	28,3217	28,5256	27,8941	+	9	9	9	48,7	48,7	48,7	13,204	9,01E+09	125	27,8688	27,4657	28,2719	10	5	5	0,000107289	0,806199	A0A0M1PBF3	A0A0M1PBF3	rplS	50S ribosomal protein L19
23,057	22,8753	22,6976	22,837	23,0396	23,8538	23,7396	23,648	23,6058	23,7254	+	2	2	2	3,7	3,7	3,7	49,151	1,75E+08	15	23,3079	22,9013	23,7145	10	5	5	7,09E-06	0,813227	A0A133N5X2	A0A133N5X2	rho	Transcription termination factor Rho
22,5861	22,4738	22,7124	22,5746	22,7465	23,673	23,7687	23,2534	23,1913	23,5003	+	5	5	5	7,3	7,3	7,3	76,055	1,61E+08	22	23,048	22,6187	23,4773	10	5	5	0,000117841	0,858662	A0A133CPC3	A0A133CPC3	parE	DNA topoisomerase 4 subunit B
25,0154	24,9924	24,8259	24,9854	24,8968	25,8541	25,9688	25,8392	25,9198	25,6444	+	7	7	7	19,2	19,2	19,2	46,225	1,19E+09	32	25,3942	24,9432	25,8453	10	5	5	7,74E-07	0,902084	A0A132P9J9	A0A132P9J9	clpX_1	ATP-dependent Clp protease ATP-binding subunit ClpX
24,1494	23,826	23,7944	23,8166	23,8422	24,8538	24,9007	24,7175	24,4769	25,0512	+	5	5	5	6,3	6,3	6,3	103,77	5,32E+08	38	24,3429	23,8857	24,8	10	5	5	5,28E-05	0,914313	A0A133CK43	A0A133CK43	uvrA	UvrABC system protein B system protein A
27,5094	27,3601	27,6382	27,5519	27,3172	28,5559	28,455	28,3122	28,5489	28,1474	+	7	7	7	39,3	39,3	39,3	13,074	1,16E+10	158	27,9396	27,4753	28,4039	10	5	5	1,28E-05	0,928522	A0A132P5M0	A0A132P5M0	rplN	50S ribosomal protein L14
29,3387	28,8132	29,2901	28,9499	28,8256	30,0534	30,1606	29,6223	29,9633	30,0615	+	5	5	5	35	35	35	14,847	9,93E+09	79	29,5079	29,0435	29,9722	10	5	5	0,000224136	0,928715	A0A132P4N2	A0A132P4N2	rplK	50S ribosomal protein L11
24,0146	23,7762	23,6341	23,7403	23,6594	24,8683	24,6565	24,6628	24,8466	24,4927	+	2	2	2	8,4	8,4	8,4	24,871	2,77E+08	16	24,2351	23,7649	24,7054	10	5	5	1,05E-05	0,94047	A0A132ZJ25	A0A132ZJ25	rpiA_2	Ribose-5-phosphate isomerase A
28,6887	28,1584	28,4186	28,3075	28,7026	29,7168	29,2318	29,1199	29,7783	29,1493	+	4	4	4	56,5	56,5	56,5	7,3435	6,02E+09	71	28,9272	28,4551	29,3992	10	5	5	0,000748899	0,944085	A0A0W7UEK9	A0A0W7UEK9	rpmC	50S ribosomal protein L29
23,7042	23,4079	23,4713	23,133	23,1882	24,4089	24,4676	24,3048	24,0855	24,3713	+	6	6	6	27,6	27,6	27,6	28,539	5,20E+08	33	23,8543	23,3809	24,3276	10	5	5	5,54E-05	0,946657	A0A132Z470	A0A132Z470	sufC	ABC superfamily ATP binding cassette transporter, ABC protein
23,5831	23,6855	23,4853	23,3693	23,8846	24,5846	24,9404	24,4169	24,4413	24,4361	+	10	10	10	11,7	11,7	11,7	92,05	7,79E+08	53	24,0827	23,6016	24,5639	10	5	5	8,61E-05	0,962281	A0A132Z2W3	A0A132Z2W3	leuS	Leucine--tRNA ligase
23,8672	23,7239	23,9582	24,0078	23,992	24,9538	24,9855	24,9291	25,1139	24,9106	+	2	2	2	7,4	7,4	7,4	33,975	3,84E+08	19	24,4442	23,9098	24,9786	10	5	5	1,61E-07	1,06877	A0A132Z8J9	A0A132Z8J9	parB	Chromosome partitioning protein ParB
23,5116	24,3683	24,604	24,6844	24,3388	25,5483	25,89	25,3459	24,9251	25,4663	+	5	5	5	28,4	28,4	28,4	25,873	9,03E+08	46	24,8683	24,3014	25,4351	10	5	5	0,00243592	1,1337	A0A132P5X4	A0A132P5X4	srrA_1	DNA-binding response regulator
24,2855	23,8926	24,1018	24,0287	24,172	25,386	25,3271	25,2816	24,8225	25,4071	+	10	10	10	17,3	17,3	17,3	91,797	1,00E+09	49	24,6705	24,0961	25,2449	10	5	5	1,74E-05	1,14879	A0A132Z8F9	A0A132Z8F9	gyrA	DNA gyrase subunit A
24,1363	24,0636	24,0829	24,3701	24,2625	25,4589	25,4095	25,5462	24,9066	25,3673	+	5	5	5	14,5	14,5	14,5	46,793	7,35E+08	36	24,7604	24,1831	25,3377	10	5	5	1,63E-05	1,15463	A0A132Z1N3	A0A132Z1N3	B1P95_03705	Mannosyl-glycoprotein endo-beta-N-acetylglucosamidase
23,3229	23,1293	23,1533	23,1953	23,2598	24,3765	24,5893	24,5229	23,9787	24,3796	+	6	6	6	12,2	12,2	12,2	71,208	4,74E+08	28	23,7908	23,2121	24,3694	10	5	5	6,55E-06	1,15727	A0A133D0H0	A0A133D0H0	B1P95_06245	Peptidoglycan hydrolase
26,4075	26,6889	26,602	26,1058	26,2564	27,7171	27,5777	27,7418	27,153	27,7535	+	9	9	9	17,4	17,4	17,4	81,821	1,82E+09	51	27,0004	26,4121	27,5886	10	5	5	6,71E-05	1,17651	A0A132Z0Q8	A0A132Z0Q8	nrdE	Ribonucleoside-diphosphate reductase
27,0585	26,6572	27,0009	26,8614	27,1368	28,382	28,2692	27,8328	28,3824	28,0487	+	4	4	4	34,5	34,5	34,5	6,958	4,01E+09	20	27,563	26,9429	28,183	10	5	5	1,69E-05	1,24009	A0A133N3M7	A0A133N3M7	rpsU	30S ribosomal protein S21
26,9749	26,4469	26,3712	26,2862	26,5041	27,6591	27,8535	27,7023	27,9727	27,7659	+	3	3	3	16,2	16,2	16,2	20,786	1,63E+09	26	27,1537	26,5166	27,7907	10	5	5	1,15E-05	1,27407	A0A133MZI7	A0A133MZI7	frr	Ribosome-recycling factor
26,1126	25,8719	25,8962	25,8735	25,7114	27,2058	27,3193	27,1206	27,1099	27,0869	+	5	5	5	19,2	19,2	19,2	37,513	1,57E+09	83	26,5308	25,8931	27,1685	10	5	5	1,78E-07	1,27537	A0A132YZI1	A0A132YZI1	recA_1	Protein RecA
27,6652	26,6022	26,6337	27,1014	26,6495	27,5524	27,45	27,7054	30,2301	28,264	+	4	4	4	21,8	21,8	21,8	27,617	3,44E+09	19	27,5854	26,9304	28,2404	10	5	5	0,0463006	1,31001	A0A132Z3T2	A0A132Z3T2	relA	GTP diphosphokinase
23,8886	23,7206	23,7407	23,5737	23,7623	25,0836	25,172	25,1732	24,7505	25,0698	+	13	13	13	25,1	25,1	25,1	76,873	1,43E+09	97	24,3935	23,7372	25,0498	10	5	5	6,01E-07	1,31267	A0A132ZA27	A0A132ZA27	B1P95_00715	N-acetylmuramoyl-L-alanine amidase
23,5115	23,8011	23,8074	23,8771	23,0984	25,0931	25,0213	25,3977	25,1708	24,7131	+	5	5	5	27,4	27,4	27,4	26,786	6,57E+08	26	24,3492	23,6191	25,0792	10	5	5	4,35E-05	1,46008	A0A132Z3I3	A0A132Z3I3	vicR	DNA-binding response regulator
25,4392	25,1415	25,1308	25,2138	25,2196	26,7133	26,8727	26,8492	26,5805	26,765	+	5	5	5	4,6	4,6	4,6	117,34	1,42E+09	34	25,9926	25,229	26,7561	10	5	5	4,10E-08	1,52716	A0A5P3FXN5	A0A5P3FXN5	carB	Carbamoyl-phosphate synthase large chain
24,951	24,9937	24,786	24,6916	25,0095	26,3507	26,4542	26,6698	25,8761	26,804	+	14	14	14	24,2	24,2	24,2	55,494	3,63E+09	93	25,6586	24,8864	26,4309	10	5	5	1,86E-05	1,54459	Q9K2J9	Q9K2J9	sagA	NlpC/P60 family lipoprotein
22,9209	22,9848	23,1265	23,0935	23,2149	24,8805	24,6975	24,9451	24,8033	24,4459	+	4	4	4	19,3	19,3	19,3	32,12	3,64E+08	17	23,9113	23,0681	24,7545	10	5	5	1,78E-07	1,68638	A0A132ZBS9	A0A132ZBS9	amiC	Peptidoglycan hydrolase
24,9991	24,9048	24,9336	24,7647	25,133	27	26,961	26,6561	26,5797	26,6503	+	5	5	5	14,6	14,6	14,6	37,39	1,37E+09	55	25,8582	24,947	26,7694	10	5	5	1,35E-07	1,82242	A0A133CSI4	A0A133CSI4	nrdF	Ribonucleoside-diphosphate reductase subunit beta
28,0724	27,7797	27,9861	28,0355	27,9538	30,1472	30,1947	30,049	29,9751	29,7971	+	5	5	5	42	42	42	9,3125	1,89E+10	169	28,9991	27,9655	30,0326	10	5	5	1,01E-08	2,06711	A0A0M1PFJ8	A0A0M1PFJ8	ptsH	Phosphocarrier protein HPr
25,2968	23,631	24,4647	24,2828	23,8479	23,3243	23,2995	23,8118	25,115	23,3292		5	5	5	53,8	53,8	53,8	9,1116	3,77E+08	14	24,0403	24,3047	23,776	10	5	5	0,276417	-0,528699	A0A132P6M3	A0A132P6M3	rpsR	30S ribosomal protein S18
27,5069	27,8129	27,7358	27,5319	27,3727	26,9142	27,2514	27,3265	27,8076	26,9494		16	16	16	56,9	56,9	56,9	38,382	7,34E+09	186	27,4209	27,592	27,2498	10	5	5	0,0938687	-0,342207	A0A133CQ32	A0A133CQ32	arcB	Ornithine carbamoyltransferase
27,7065	27,165	27,2537	27,0192	26,5891	26,9093	26,835	26,1219	27,4682	26,7703		6	6	6	36,5	36,5	36,5	17,773	2,32E+09	69	26,9838	27,1467	26,8209	10	5	5	0,278516	-0,325767	D8V1G7	D8V1G7	rplJ	50S ribosomal protein L10
24,3683	24,5949	24,5371	24,5283	23,7664	24,2116	23,8968	23,308	24,5401	24,3189		3	3	3	26,4	26,4	26,4	19,182	4,96E+08	18	24,207	24,359	24,0551	10	5	5	0,280627	-0,303915	A0A132Z6F8	A0A132Z6F8	atpF	ATP synthase subunit b
26,8719	26,9716	26,7818	26,9575	26,8342	26,6562	26,5463	26,7125	26,9504	26,2203		6	6	6	24,3	24,3	24,3	38,385	1,53E+09	20	26,7503	26,8834	26,6171	10	5	5	0,0650299	-0,266249	A0A132P6P6	A0A132P6P6	lplA	Lipoate--protein ligase
26,9807	26,8078	26,9524	26,972	26,9763	26,4321	26,4682	26,9591	26,9131	26,6123		10	10	10	41,2	41,2	41,2	35,516	1,72E+09	46	26,8074	26,9378	26,677	10	5	5	0,0530852	-0,260865	A0A133CSV6	A0A133CSV6	pdhB	2-oxoisovalerate dehydrogenase
22,2329	22,295	22,4728	22,4879	22,369	22,3864	21,8092	21,876	22,3289	22,166		3	3	3	17,7	17,7	17,7	31,102	1,19E+08	12	22,2424	22,3715	22,1133	10	5	5	0,0760729	-0,25822	A0A132ZCS1	A0A132ZCS1	degV	DegV family EDD domain-containing protein
25,1541	24,961	25,0069	24,8929	24,9188	24,8608	24,9121	25,0301	24,01	24,8619		11	11	11	15,2	15,2	15,2	95,874	9,00E+08	41	24,8609	24,9867	24,735	10	5	5	0,220716	-0,251766	A0A132Z6H7	A0A132Z6H7	secA	Protein translocase subunit SecA
26,7468	26,3971	26,5618	26,5761	26,2254	26,3472	26,3637	26,3046	26,3537	25,8946		2	2	2	39,4	39,4	39,4	7,1988	1,83E+09	80	26,3771	26,5014	26,2528	10	5	5	0,0844208	-0,24869	A0A132Z8Y7	A0A132Z8Y7	cspE_1	Cold shock domain-containing protein
27,353	27,0453	27,588	27,3691	27,3482	26,8727	27,0285	27,0984	27,5204	26,9664		8	8	8	49,2	49,2	49,2	12,468	6,11E+09	115	27,219	27,3407	27,0973	10	5	5	0,123746	-0,243462	A0A133CSE1	A0A133CSE1	rplL	50S ribosomal protein L7/L12
25,9756	25,7971	26,1254	25,7565	26,0564	25,8204	25,5971	25,8567	25,9531	25,3371		3	3	3	20,4	20,4	20,4	15,707	1,60E+09	31	25,8275	25,9422	25,7129	10	5	5	0,120241	-0,229332	A0A133CT22	A0A133CT22	B1P95_13505	Alpha crystallin family heat shock protein
27,6505	28,0363	28,1613	28,1593	28,1235	27,9185	27,7341	27,9853	27,6627	27,707		10	10	10	48,2	48,2	48,2	25,955	9,99E+09	109	27,9139	28,0262	27,8015	10	5	5	0,0876885	-0,224658	A0A132ZJA4	A0A132ZJA4	gpmA_2	2,3-bisphosphoglycerate-dependent phosphoglycerate mutase
25,4589	25,5614	25,6196	25,4781	25,5734	24,9347	25,2058	25,4663	25,5879	25,3935		15	15	15	48,9	48,9	48,9	46,174	3,27E+09	96	25,428	25,5383	25,3176	10	5	5	0,0983488	-0,220643	A0A133CQ31	A0A133CQ31	arcA	Arginine deiminase
26,9201	26,268	26,8753	26,6506	26,9494	26,6709	26,2955	26,416	26,8421	26,3541		3	3	3	62,1	62,1	62,1	9,9508	1,63E+09	27	26,6242	26,7327	26,5157	10	5	5	0,223332	-0,216953	A0A133N5W3	A0A133N5W3	rpmE2	50S ribosomal protein L31 type B
22,2545	22,337	22,468	22,7687	22,3454	22,5364	22,193	22,2499	22,0934	22,0217		3	3	3	14,3	14,3	14,3	31,058	1,27E+08	12	22,3268	22,4347	22,2189	10	5	5	0,126186	-0,215851	A0A133CPM0	A0A133CPM0	B1P95_11620	DegV family EDD domain-containing protein
26,1219	25,9097	26,0211	25,9767	26,0366	25,9756	25,6659	25,7664	26,0437	25,6078		10	10	10	46,9	46,9	46,9	26,362	1,65E+09	70	25,9125	26,0132	25,8119	10	5	5	0,060568	-0,201314	A0A133MWA8	A0A133MWA8	arlR_2	DNA-binding response regulator
24,1967	24,1273	24,4323	24,3523	24,4353	23,8744	24,1285	24,159	24,1973	24,1864		6	6	6	18,8	18,8	18,8	44,769	3,99E+08	21	24,2089	24,3088	24,1091	10	5	5	0,0502592	-0,199664	A0A133CK16	A0A133CK16	glyA	Serine hydroxymethyltransferase
24,0142	23,7607	24,0523	23,8726	24,0136	23,471	23,7255	23,9102	24,0737	23,5445		4	4	4	23,5	23,5	23,5	20,811	4,45E+08	21	23,8438	23,9427	23,745	10	5	5	0,151686	-0,197682	Q842S4	Q842S4	def_2	Peptide deformylase
23,2267	24,071	24,1319	24,0988	24,3612	23,7502	23,8302	23,8149	24,1262	23,3898		6	6	6	17,8	17,8	17,8	38,118	5,16E+08	28	23,8801	23,9779	23,7823	10	5	5	0,414745	-0,195664	A0A133CIV5	A0A133CIV5	gfo	Gfo/Idh/MocA family oxidoreductase
27,3961	27,0092	27,4672	27,3747	27,48	26,9189	26,8984	27,2912	27,5816	27,1003		7	7	7	48,6	48,6	48,6	20,491	2,52E+09	32	27,2518	27,3454	27,1581	10	5	5	0,258522	-0,187347	A0A132PA28	A0A132PA28	gls20	Alkaline shock protein 23 family protein
24,4177	24,5482	24,6509	24,4328	24,5216	24,1758	24,6209	24,5	24,3141	24,1051		4	4	4	20,1	20,1	20,1	31,911	6,43E+08	28	24,4287	24,5142	24,3432	10	5	5	0,143984	-0,171061	A0A133CIA9	A0A133CIA9	rmlA	Glucose-1-phosphate thymidylyltransferase
25,8062	26,6338	26,75	26,573	26,6102	26,2232	26,5085	26,3574	26,5892	25,8495		3	3	3	22,5	22,5	22,5	13,736	1,77E+09	38	26,3901	26,4746	26,3056	10	5	5	0,452065	-0,169071	A0A0M1PG85	A0A0M1PG85	rpsK	30S ribosomal protein S11
26,3938	26,5477	26,4135	26,3467	26,3385	26,0013	26,0011	26,4197	26,4344	26,3452		16	16	16	38,2	38,2	38,2	47,983	2,99E+09	94	26,3242	26,4081	26,2404	10	5	5	0,151365	-0,167696	A0A132YYN1	A0A132YYN1	tig	Trigger factor
26,9577	26,7537	26,8051	26,7667	26,7253	26,6693	26,7165	26,8213	26,5018	26,4702		18	18	18	43,3	43,3	43,3	49,753	3,71E+09	110	26,7188	26,8017	26,6358	10	5	5	0,0655873	-0,165909	A0A132Z0X6	A0A132Z0X6	asnC	Asparagine--tRNA ligase
26,2199	26,0385	26,5342	26,0577	25,9411	25,9273	25,7852	26,0147	26,4799	25,7689		7	7	7	56,5	56,5	56,5	10,521	2,55E+09	54	26,0767	26,1583	25,9952	10	5	5	0,354898	-0,163127	A0A0M1PG69	A0A0M1PG69	rpsS	30S ribosomal protein S19
23,3386	23,5353	23,6081	23,7525	23,4959	23,2771	23,2447	23,5955	23,4677	23,3389		6	6	6	24,7	24,7	24,7	21,267	3,98E+08	25	23,4654	23,5461	23,3848	10	5	5	0,124496	-0,161296	A0A132P3Y6	A0A132P3Y6	ppi_2	Peptidyl-prolyl cis-trans isomerase
23,9826	24,0531	24,036	23,9941	24,1079	23,787	23,8405	23,987	24,0648	23,7036		4	4	4	28,6	28,6	28,6	15,561	4,74E+08	29	23,9557	24,0347	23,8766	10	5	5	0,0527497	-0,15817	A0A133CTS0	A0A133CTS0	atpC	ATP synthase epsilon chain
24,9054	24,973	25,2159	25,0523	24,8341	24,8235	24,9323	24,9309	24,778	24,7576		7	7	7	44,1	44,1	44,1	27,977	1,02E+09	26	24,9203	24,9962	24,8445	10	5	5	0,0794853	-0,151694	A0A133CIA7	A0A133CIA7	map	Methionine aminopeptidase
25,2804	25,0039	24,8778	25,0578	24,9724	24,7003	25,2402	25,0136	24,7824	24,7704		8	8	8	27,4	27,4	27,4	39,392	9,75E+08	24	24,9699	25,0385	24,9014	10	5	5	0,287337	-0,137105	A0A132Z5G9	A0A132Z5G9	pheS	Phenylalanine--tRNA ligase alpha subunit
25,7166	25,6012	25,6367	25,4928	25,6683	25,3538	25,4466	25,6789	25,5228	25,4451		12	12	12	29,5	29,5	29,5	60,372	1,95E+09	65	25,5563	25,6231	25,4894	10	5	5	0,0781311	-0,133681	A0A133CZU5	A0A133CZU5	yloV	DAK2 domain fusion protein YloV
23,9964	23,645	23,8859	23,8044	24,3335	23,8907	23,7586	23,7936	23,8023	23,7609		4	4	4	29,4	29,4	29,4	10,203	3,89E+08	13	23,8671	23,933	23,8012	10	5	5	0,2957	-0,131802	A0A132P9R4	A0A132P9R4	AWT83_0951	Uncharacterized protein
24,6282	24,3926	24,435	24,424	24,2329	24,3639	24,2509	24,394	24,3385	24,2101		4	4	4	38,3	38,3	38,3	20,89	4,77E+08	17	24,367	24,4225	24,3115	10	5	5	0,161431	-0,111018	A0A133CTP9	A0A133CTP9	atpH	ATP synthase subunit delta
27,4375	27,6953	27,838	27,6529	27,7361	27,5035	27,546	27,7798	27,5654	27,4681		21	21	21	68,6	68,6	68,6	32,243	1,07E+10	214	27,6223	27,672	27,5726	10	5	5	0,279562	-0,0994072	A0A132Z3T0	A0A132Z3T0	tsf	Elongation factor Ts
24,8431	24,8853	24,7901	24,7871	24,8721	24,8712	24,7155	24,7972	24,5588	24,7444		8	8	8	14,9	14,9	14,9	83,832	1,03E+09	44	24,7865	24,8355	24,7374	10	5	5	0,116606	-0,0981075	A0A132P2N7	A0A132P2N7	pflB	Formate acetyltransferase
25,0911	25,0328	25,0831	25,0618	25,0362	24,9393	24,804	25,0687	25,0583	24,971		11	11	11	47,7	47,7	47,7	31,998	2,15E+09	55	25,0146	25,061	24,9683	10	5	5	0,0973858	-0,0927422	A0A133CTS4	A0A133CTS4	yvgN	2,5-diketo-D-gluconic acid reductase
24,4067	24,4	24,6108	24,636	24,7033	24,3668	24,4136	24,5407	24,6108	24,4168		7	7	7	39,9	39,9	39,9	19,934	5,60E+08	16	24,5105	24,5513	24,4697	10	5	5	0,321199	-0,0816082	A0A5P3FRK9	A0A5P3FRK9	F6440_05160	DsbA family protein
27,5095	27,6301	27,6162	27,6212	27,5478	27,4233	27,3619	27,648	27,7117	27,4105		15	15	15	54,8	54,8	54,8	34,701	6,77E+09	156	27,548	27,5849	27,5111	10	5	5	0,349372	-0,0738766	A0A133CN32	A0A133CN32	galU	UTP--glucose-1-phosphate uridylyltransferase
25,7577	25,0659	25,6178	25,3267	25,5191	25,5871	25,3603	25,5078	25,6623	24,9122		5	5	5	33,3	33,3	33,3	12,185	1,28E+09	26	25,4317	25,4575	25,4059	10	5	5	0,781491	-0,0515129	E9KIK4	E9KIK4	AWT83_0044	Uncharacterized protein
22,9543	23,0966	23,1631	22,9201	22,8896	22,9553	22,6528	22,9731	23,3074	22,9372		3	3	3	14,9	14,9	14,9	29,602	2,14E+08	12	22,985	23,0047	22,9652	10	5	5	0,743156	-0,039576	A0A132Z6H6	A0A132Z6H6	yidA_1	Cof-type HAD-IIB family hydrolase
30,6504	30,4566	30,5635	30,392	30,51	30,3173	30,316	30,6229	30,5892	30,5498		32	32	32	63,4	63,4	63,4	57,184	3,62E+10	771	30,4968	30,5145	30,479	10	5	5	0,671243	-0,0354851	A0A132Z3A5	A0A132Z3A5	groL	60 kDa chaperonin
23,4306	23,3732	23,4354	23,378	23,442	23,3331	23,2984	23,3788	23,5906	23,2905		6	6	6	26,3	26,3	26,3	30,632	4,42E+08	37	23,395	23,4118	23,3783	10	5	5	0,574051	-0,0335751	A0A132Z952	A0A132Z952	nadE	NH(3)-dependent NAD(+) synthetase
23,6922	23,5253	23,4164	23,4083	23,647	23,5029	23,55	23,5157	23,7794	23,1961		9	9	9	27,6	27,6	27,6	44,284	3,77E+08	26	23,5233	23,5378	23,5088	10	5	5	0,797878	-0,0290154	A0A132P9H5	A0A132P9H5	nusA	Transcription termination/antitermination protein NusA
23,9013	23,7833	24,1944	23,9697	24,257	23,9678	23,9012	23,9883	24,3465	23,7683		7	7	7	54,7	54,7	54,7	16,808	6,64E+08	27	24,0078	24,0211	23,9944	10	5	5	0,843644	-0,0267113	A0A132Z291	A0A132Z291	A5810_00212	Aspartyl-tRNA amidotransferase
22,7789	22,8048	22,8721	22,7436	22,7827	22,797	22,6236	22,6446	23,1249	22,6689		5	5	5	27,5	27,5	27,5	29,453	2,17E+08	10	22,7841	22,7964	22,7718	10	5	5	0,803615	-0,0245995	A0A132P424	A0A132P424	ycfH_1	Hydrolase TatD
28,8627	29,1146	29,19	29,075	28,6641	28,9992	28,7562	28,9015	29,4371	28,715		9	9	9	68,9	68,9	68,9	14,84	1,15E+10	159	28,9715	28,9813	28,9618	10	5	5	0,906551	-0,0195152	A0A0M1PG81	A0A0M1PG81	rpsH	30S ribosomal protein S8
24,258	24,2655	24,4026	24,3987	24,258	24,3923	24,2199	24,4283	24,3024	24,144		7	7	7	18,8	18,8	18,8	45,094	4,28E+08	14	24,307	24,3165	24,2974	10	5	5	0,768772	-0,0191528	A0A133CDW4	A0A133CDW4	telA	Tellurite resistance protein
28,4423	28,2668	28,469	28,3626	28,2884	28,2664	28,3559	28,3924	28,5866	28,1479		14	14	14	50,8	50,8	50,8	29,33	1,37E+10	199	28,3578	28,3658	28,3498	10	5	5	0,852113	-0,0159908	A0A132P9G3	A0A132P9G3	rpsB	30S ribosomal protein S2
26,0282	26,1387	26,2183	26,0776	25,9918	26,1601	25,9981	26,1282	26,1086	26,0378		10	10	10	42,6	42,6	42,6	36,452	2,38E+09	58	26,0888	26,0909	26,0866	10	5	5	0,933289	-0,00433044	A0A133CMF3	A0A133CMF3	ccpA	Catabolite control protein A
25,5372	25,4742	25,7751	25,7333	25,3538	25,4368	25,7423	25,7806	25,7147	25,2468		5	5	5	36,6	36,6	36,6	22,892	8,19E+08	22	25,5795	25,5747	25,5842	10	5	5	0,943837	0,00950279	A0A133CIE4	A0A133CIE4	sodA	Superoxide dismutase
25,284	25,3597	25,251	25,1201	25,1702	25,2112	25,3092	25,3435	25,2216	25,1528		17	17	17	30,8	30,8	30,8	76,114	2,04E+09	109	25,2423	25,237	25,2477	10	5	5	0,849901	0,0106678	A0A133CLD1	A0A133CLD1	metG	Methionine--tRNA ligase
23,3986	24,1926	24,4328	24,126	23,6832	24,2309	23,9381	24,284	23,7678	23,6909		4	4	4	42,9	42,9	42,9	17,243	3,60E+08	26	23,9745	23,9666	23,9824	10	5	5	0,945203	0,0157181	A0A133CVR7	A0A133CVR7	AWT83_0226	Universal stress protein
24,4916	24,4183	24,5929	24,4609	24,9105	24,3878	24,5827	24,4459	25,1003	24,4451		6	6	6	33	33	33	21,107	7,01E+08	24	24,5836	24,5748	24,5924	10	5	5	0,914553	0,0175179	A0A132Z772	A0A132Z772	AWT83_0885	General stress protein
27,8931	28,0342	28,1269	28,0602	27,8879	28,0104	28,0397	28,0193	28,0885	27,9668		14	14	14	30	30	30	52,59	3,80E+09	69	28,0127	28,0005	28,0249	10	5	5	0,646771	0,0244545	A0A133CPD2	A0A133CPD2	hslU	ATP-dependent protease ATPase subunit HslU
23,6311	23,6009	23,6966	23,6798	23,7801	23,791	23,3483	23,9408	24,0727	23,477		6	6	6	15,9	15,9	15,9	52,51	2,71E+08	19	23,7018	23,6777	23,726	10	5	5	0,739821	0,0482693	A0A132P8P7	A0A132P8P7	ffh	Signal recognition particle protein
27,2248	26,8972	27,0275	26,8686	27,0759	27,0789	26,9385	27,1637	27,4003	26,7979		8	8	8	53,5	53,5	53,5	17,893	3,98E+09	83	27,0473	27,0188	27,0759	10	5	5	0,649505	0,0570637	A0A133CUD3	A0A133CUD3	dps	DNA protection during starvation protein 1
26,6714	26,798	26,6039	26,5729	26,4989	26,8959	26,6234	26,6741	26,6818	26,5596		16	16	16	43,8	43,8	43,8	43,325	3,56E+09	106	26,658	26,629	26,687	10	5	5	0,467074	0,0579273	A0A132Z3Y1	A0A132Z3Y1	metK	S-adenosylmethionine synthase
26,3133	26,1521	26,3431	26,3459	26,4368	26,448	26,1998	26,3666	26,7033	26,1738		4	4	4	46,4	46,4	46,4	10,427	2,85E+09	50	26,3483	26,3182	26,3783	10	5	5	0,588404	0,0601028	A0A132ZFM0	A0A132ZFM0	rpmA	50S ribosomal protein L27
26,8377	27,2055	26,8969	26,6998	26,8625	26,9973	26,9733	26,9554	27,1181	26,7774		9	9	9	25,6	25,6	25,6	50,6	1,73E+09	33	26,9324	26,9005	26,9643	10	5	5	0,539553	0,0638462	A0A133N4C0	A0A133N4C0	glnA	Glutamine synthetase
28,6835	28,6517	28,7829	28,6095	28,6495	28,8332	28,6485	28,9322	28,7717	28,5322		14	14	14	73,7	73,7	73,7	17,812	1,88E+10	192	28,7095	28,6754	28,7436	10	5	5	0,395668	0,0681396	A0A132ZIY7	A0A132ZIY7	rpsG_2	30S ribosomal protein S7
25,2647	25,3245	25,3511	25,1976	25,0634	25,3625	25,4427	25,3622	25,1562	25,2379		15	15	15	34,9	34,9	34,9	53,617	2,25E+09	103	25,2763	25,2403	25,3123	10	5	5	0,349366	0,0719975	A0A133CN79	A0A133CN79	gatB	Aspartyl/glutamyl-tRNA(Asn/Gln) amidotransferase subunit B
32,2532	33,6211	33,6902	33,5596	33,5335	33,6512	33,534	33,4955	32,9698	33,4314		14	14	14	70,9	70,9	70,9	20,189	1,23E+11	374	33,3739	33,3315	33,4164	10	5	5	0,781044	0,0848724	A0A133N7E0	A0A133N7E0	rplE	50S ribosomal protein L5
26,921	26,1248	26,8376	26,5783	26,5865	26,8743	26,5639	26,682	26,9807	26,395		7	7	7	75,5	75,5	75,5	10,004	3,79E+09	77	26,6544	26,6097	26,6992	10	5	5	0,62104	0,089534	Q5QHP6	Q5QHP6	groES	10 kDa chaperonin
24,022	23,9298	23,6305	23,8573	22,8607	23,5867	23,6826	23,9606	24,0004	23,5929		5	5	5	11,2	11,2	11,2	68,647	3,84E+08	18	23,7123	23,66	23,7646	10	5	5	0,659352	0,104586	A0A132ZIQ4	A0A132ZIQ4	pepF1	M3 family oligoendopeptidase
30,4645	30,5247	30,6698	30,4397	30,48	30,4741	30,5493	30,7183	30,6864	30,6867		24	21	21	64,6	58,5	58,5	43,216	6,47E+10	297	30,5694	30,5157	30,623	10	5	5	0,124862	0,10723	A0A133CT44	A0A133CT44	tuf	Elongation factor Tu
26,5179	26,2979	26,5483	26,191	26,6442	26,5648	26,5605	26,5973	26,7068	26,6029		10	10	10	20,9	20,9	20,9	59,817	1,92E+09	62	26,5231	26,4398	26,6064	10	5	5	0,0956647	0,166603	A0A132P6J2	A0A132P6J2	pyrG	CTP synthase
25,9999	25,6981	25,76	25,6098	25,9255	26,1659	25,8403	25,6884	25,9291	26,2044		6	6	6	17,2	17,2	17,2	49,272	1,06E+09	28	25,8821	25,7986	25,9656	10	5	5	0,206364	0,166977	A0A132P2R8	A0A132P2R8	engA	GTPase Der
24,2419	23,7958	24,5015	24,3435	24,3581	24,2502	24,2378	24,6657	24,5889	24,3418		3	3	3	12,7	12,7	12,7	45,764	3,63E+08	18	24,3325	24,2482	24,4169	10	5	5	0,291784	0,168722	A0A133CR01	A0A133CR01	pepT	Peptidase T

25,1381	25,2568	25,7199	25,3997	25,2166	25,5984	25,4228	25,7838	25,5827	25,3776			5	5	5	34	34	34	16,145	1,61E+09	66	25,4496	25,3462	25,5531	10	5	5	0,137651	0,206864	D8V1G9	D8V1G9	rplP	50S ribosomal protein L16
26,097	26,0076	26,076	26,4326	26,3097	26,6232	26,1641	26,1611	26,6622	26,3718			5	5	5	29,2	29,2	29,2	25,957	1,27E+09	28	26,2905	26,1846	26,3965	10	5	5	0,152876	0,211906	A0A133CQN2	A0A133CQN2	pyrH_1	Uridylate kinase
27,0385	27,1176	26,9254	26,6514	26,958	27,0213	27,2258	27,3558	26,8569	27,2949			33	33	33	33,3	33,3	33,3	135,14	8,27E+09	239	27,0446	26,9382	27,1509	10	5	5	0,118826	0,212737	A0A132ZDF9	A0A132ZDF9	rpoB	DNA-directed RNA polymerase subunit beta
26,5713	26,2047	26,5013	26,1348	25,6108	26,413	26,6281	26,0557	26,7269	26,2877			5	5	5	37,5	37,5	37,5	10,252	3,18E+09	43	26,3134	26,2046	26,4223	10	5	5	0,326454	0,21771	A0A133CTX7	A0A133CTX7	rpsQ	30S ribosomal protein S17
24,5946	24,713	24,8701	24,7769	24,896	24,9018	25,3723	25,2378	24,3549	25,1248			5	5	5	20,3	20,3	20,3	35,1	6,69E+08	28	24,8842	24,7701	24,9983	10	5	5	0,25619	0,228199	A0A132Z234	A0A132Z234	glyQ	Glycine--tRNA ligase alpha subunit
23,9471	23,9223	23,9601	23,9164	23,7348	24,3361	24,2507	24,4306	23,4782	24,1314			7	7	7	23,5	23,5	23,5	36,633	4,23E+08	26	24,0108	23,8961	24,1254	10	5	5	0,22433	0,229236	A0A132Z7I3	A0A132Z7I3	ftsY	Signal recognition particle receptor FtsY
24,6649	24,7725	24,7814	24,7648	24,7396	25,4067	25,4899	24,8	24,7269	24,5457			5	5	5	17,3	17,3	17,3	41,88	5,73E+08	17	24,8692	24,7447	24,9938	10	5	5	0,229905	0,249173	A0A133CT66	A0A133CT66	sigA	RNA polymerase sigma factor SigA
26,7212	25,808	26,7664	26,3823	26,6305	26,869	26,8346	26,7889	26,5812	26,5078			7	7	7	75,8	75,8	75,8	11,611	1,77E+09	29	26,589	26,4617	26,7163	10	5	5	0,218279	0,254652	A0A132Z8H0	A0A132Z8H0	rpsF	30S ribosomal protein S6
25,4593	25,5869	25,5433	25,5595	25,5929	25,825	25,9536	26,3478	25,7193	25,2042			6	6	6	24,4	24,4	24,4	35,812	7,26E+08	39	25,6792	25,5484	25,81	10	5	5	0,198696	0,261572	A0A132Z4R6	A0A132Z4R6	prsA	Putative ribose-phosphate pyrophosphokinase
27,1034	27,0279	27,0404	27,1253	26,8699	27,6	27,1105	27,0644	27,6845	27,0953			6	6	6	39,1	39,1	39,1	20,491	1,78E+09	51	27,1721	27,0334	27,3109	10	5	5	0,0888145	0,277534	A0A132Z1L9	A0A132Z1L9	pyrR	Bifunctional protein PyrR
24,3182	24,3259	24,2139	23,967	24,1287	24,2249	24,3799	24,1297	24,7373	24,9866			4	4	4	8,7	8,7	8,7	48,252	6,30E+08	18	24,3412	24,1908	24,4917	10	5	5	0,122886	0,300931	A0A133CKV6	A0A133CKV6	serS2	Serine--tRNA ligase
25,2766	25,405	25,4831	25,4589	25,2407	25,3626	25,4578	26,3334	25,7666	25,4755			8	8	8	23	23	23	57,973	1,08E+09	26	25,526	25,3729	25,6792	10	5	5	0,133671	0,306327	A0A133CSD0	A0A133CSD0	pdhC	Dihydrolipoamide acetyltransferase component of pyruvate dehydrogenase complex
23,4855	24,1787	24,8277	24,2276	24,3473	24,8239	24,6227	24,7299	23,8857	24,6715			3	3	3	15,2	15,2	15,2	21,678	4,56E+08	18	24,3801	24,2134	24,5467	10	5	5	0,257522	0,333341	A0A133CH35	A0A133CH35	clpP	ATP-dependent Clp protease proteolytic subunit
27,8813	28,1304	28,2254	27,4091	27,9177	28,4243	28,1083	28,2054	28,6652	28,0859			6	6	6	36,1	36,1	36,1	21,108	4,74E+09	73	28,1053	27,9128	28,2978	10	5	5	0,0635666	0,385029	A0A132P8A1	A0A132P8A1	raiA	Ribosome hibernation promoting factor
NaN	NaN	NaN	21,4643	NaN	22,7008	22,6985	22,5669	NaN	23,2281			2	2	2	6,3	6,3	6,3	43,302	6,08E+07	6	22,5317	21,4643	22,7986	5	1	4	1	1,33424	A0A133CVV6	A0A133CVV6	tgt	Queuine tRNA-ribosyltransferase
23,7676	23,2148	23,6017	23,4376	NaN	23,5558	NaN	23,7473	23,7358	23,5188			2	2	2	26,2	26,2	26,2	8,8893	1,99E+08	12	23,5724	23,5054	23,6394	8	4	4	0,349642	0,134023	A0A1A7TX61	A0A1A7TX61	B1P95_02905	Uncharacterized protein
22,1806	22,1028	22,2773	NaN	22,5097	22,3881	22,4864	NaN	22,6165	22,1936			2	2	2	8,4	8,4	8,4	31,037	8,94E+07	9	22,3444	22,2676	22,4212	8	4	4	0,266746	0,153526	A0A132Z0R8	A0A132Z0R8	pstS2	Phosphate-binding protein
24,4364	24,1442	24,1297	24,0079	24,2544	23,6918	23,5424	23,4715	23,7921	NaN	+		4	4	4	13,9	13,9	13,9	35,583	4,53E+08	20	23,9412	24,1945	23,6244	9	5	4	0,000895742	-0,570085	A0A132Z9P5	A0A132Z9P5	gmuF	Mannose-6-phosphate isomerase
22,5318	22,5852	22,6669	22,6692	22,6645	22,0922	22,0037	22,2328	22,2002	NaN	+		5	5	5	9,6	9,6	9,6	64,833	1,67E+08	9	22,4051	22,6235	22,1322	9	5	4	4,91E-05	-0,491292	A0A132Z2J1	A0A132Z2J1	sph	Myosin-cross-reactive antigen-like protein
22,8951	22,8406	22,9057	22,8364	22,906	22,2389	22,6591	22,2745	NaN	22,4082	+		2	2	2	7	7	7	41,281	1,12E+08	5	22,6627	22,8768	22,3952	9	5	4	0,000790495	-0,481599	A0A132ZFY3	A0A132ZFY3	yqeH	Ribosome biogenesis GTPase YqeH
23,0164	22,9461	22,9711	23,1363	23,0718	22,6005	22,6587	22,5961	22,4129	NaN	+		8	8	8	23,6	23,6	23,6	49,974	2,50E+08	29	22,8233	23,0283	22,567	9	5	4	0,000130142	-0,46129	A0A132ZGY6	A0A132ZGY6	gdhA	Glutamate dehydrogenase
22,1077	22,0759	22,3735	22,1558	22,4053	21,6894	21,9132	21,8805	NaN	21,9701	+		5	5	5	20,1	20,1	20,1	35,881	1,34E+08	12	22,0635	22,2236	21,8633	9	5	4	0,00670615	-0,360332	A0A133N3Y6	A0A133N3Y6	ybeZ	AAA family ATPase
22,9886	22,5794	22,8628	22,8358	23,0759	22,3587	22,3909	22,7036	22,753	NaN	+		2	2	2	6,8	6,8	6,8	37,234	1,79E+08	17	22,7276	22,8685	22,5515	9	5	4	0,0467262	-0,316954	A0A132Z5A5	A0A132Z5A5	trpS	Tryptophan--tRNA ligase
23,8087	23,5305	23,6398	23,7676	23,8776	23,3872	NaN	23,3133	23,5161	23,497	+		5	5	5	18,4	18,4	18,4	38,817	3,39E+08	14	23,5931	23,7248	23,4284	9	5	4	0,00855525	-0,296461	A0A132P540	A0A132P540	queA	S-adenosylmethionine:tRNA ribosyltransferase-isomerase
22,7713	22,5995	22,7535	22,7105	22,8678	22,5151	NaN	22,5166	22,6617	22,4635	+		4	4	4	14,3	14,3	14,3	43,473	1,65E+08	13	22,6511	22,7405	22,5392	9	5	4	0,0142098	-0,201284	A0A132Z4I6	A0A132Z4I6	DTPHA_6021	Hydroxymethylglutaryl-CoA synthase
23,4586	23,3641	23,5088	23,3865	23,6411	23,1474	23,2754	23,3406	NaN	23,4139	+		3	3	3	17,2	17,2	17,2	24,86	2,82E+08	11	23,3929	23,4718	23,2943	9	5	4	0,0497693	-0,177533	A0A133CEM5	A0A133CEM5	DTPHA_6023	Tyrosine-protein kinase CpsD
26,6722	26,6444	26,6834	26,3475	26,7801	27,0986	26,9012	26,7724	NaN	26,8486	+		8	8	8	18,7	18,7	18,7	67,753	1,12E+09	23	26,7498	26,6255	26,9052	9	5	4	0,030072	0,279681	A0A132Z8R4	A0A132Z8R4	ezrA_2	Septation ring formation regulator EzrA
23,2212	23,216	23,2037	23,2747	23,4029	23,4862	23,5826	23,434	23,7547	NaN	+		4	4	4	23,8	23,8	23,8	23,094	2,46E+08	15	23,3973	23,2637	23,5644	9	5	4	0,00503323	0,30068	A0A132Z003	A0A132Z003	pcp	Pyrrolidone-carboxylate peptidase
22,8182	22,4824	22,6762	22,5612	22,219	23,4319	23,5573	22,7244	NaN	22,8615	+		5	5	5	18,3	18,3	18,3	34,698	1,47E+08	15	22,8147	22,5514	23,1438	9	5	4	0,0277177	0,592369	A0A132Z8J5	A0A132Z8J5	ansA	Asparaginase
25,0363	25,0461	24,5344	24,2978	24,3847	NaN	26,1939	25,9193	25,7078	24,636	+		3	3	3	33,9	33,9	33,9	12,98	7,31E+08	32	25,084	24,6598	25,6142	9	5	4	0,0295543	0,9544	A0A132P5G1	A0A132P5G1	rplR	50S ribosomal protein L18
25,0552	24,9434	24,7074	24,7873	25,049	25,9415	25,9387	26,6952	27,0066	NaN	+		3	3	3	6	6	6	77,094	8,88E+08	6	25,5694	24,9085	26,3955	9	5	4	0,000577087	1,48705	A0A132Z5C0	A0A132Z5C0	ulaC_2	BglG family transcription antiterminator
23,4371	22,7546	23,1367	22,9112	22,6891	25,598	25,2613	24,7794	NaN	24,1747	+		2	2	2	60,5	60,5	60,5	4,4274	4,51E+08	13	23,8602	22,9857	24,9534	9	5	4	0,000406639	1,96761	A0A0M1PGZ2	A0A0M1PGZ2	rpmJ	50S ribosomal protein L36
24,0448	24,1714	24,3371	24,1872	24,1319	23,8867	23,6959	NaN	24,2508	23,7863			3	3	3	23,2	23,2	23,2	20,349	2,85E+08	15	24,0547	24,1745	23,9049	9	5	4	0,0590371	-0,269577	A0A132P685	A0A132P685	hpt	Hypoxanthine phosphoribosyltransferase
22,6811	22,9289	22,9433	22,7889	22,9901	22,4311	NaN	22,5555	22,8741	22,6916			5	5	5	17,1	17,1	17,1	41,142	1,94E+08	7	22,765	22,8665	22,6381	9	5	4	0,0674647	-0,228389	A0A133CHL8	A0A133CHL8	CUM68_0842	NADH-dependent flavin oxidoreductase
22,4539	22,5409	22,5827	22,3418	22,3377	22,5721	22,3775	21,6131	NaN	22,3677			2	2	2	16,6	16,6	16,6	18,566	9,00E+07	8	22,3542	22,4514	22,2326	9	5	4	0,297685	-0,218818	A0A132Z169	A0A132Z169	trpF	UPF0234 protein AS238_02080
22,5867	23,3969	23,4778	23,1674	23,5144	23,2105	23,125	23,2168	22,7785	NaN			3	3	3	11,1	11,1	11,1	33,227	1,71E+08	14	23,1638	23,2286	23,0827	9	5	4	0,518353	-0,14594	A0A132Z9U7	A0A132Z9U7	catE	Extradiol dioxygenase
23,1517	23,421	23,8671	23,3635	23,6471	23,2758	NaN	23,6333	23,3861	23,2012			8	8	8	16,6	16,6	16,6	62,602	2,83E+08	15	23,4385	23,4901	23,3741	9	5	4	0,497741	-0,115949	A0A132Z0C0	A0A132Z0C0	rnjB	Ribonuclease J
23,694	23,581	23,7871	23,5209	23,5608	23,7317	23,7184	23,8011	NaN	23,4133			3	3	3	27,6	27,6	27,6	20,26	2,58E+08	7	23,6454	23,6287	23,6661	9	5	4	0,702364	0,0373767	A0A132ZE39	A0A132ZE39	B1P95_06845	DUF664 domain-containing protein
22,7612	22,6145	22,5951	22,3691	22,8351	22,6957	22,2883	22,9115	22,8371	NaN			3	3	3	6,6	6,6	6,6	58,018	1,16E+08	10	22,6564	22,635	22,6831	9	5	4	0,761102	0,0481308	A0A133CPX4	A0A133CPX4	rny	Ribonuclease Y
NaN	NaN	NaN	NaN	NaN	NaN	24,9244	24,8027	25,0035	NaN			3	3	3	5,8	5,8	5,8	78,961	2,44E+08	6	24,9102	NaN	24,9102	3	0	3	1	NaN	A0A133CPM9	A0A133CPM9	topA	DNA topoisomerase 1
22,6296	22,6023	22,2769	22,1553	NaN	NaN	21,8751	NaN	21,8923	21,9745	+		3	3	3	4,9	4,9	4,9	70,054	8,41E+07	11	22,2008	22,416	21,914	7	4	3	0,0167261	-0,502052	A0A132ZA35	A0A132ZA35	gidA2	tRNA uridine 5-carboxymethylaminomethyl modification enzyme MnmG
23,6128	23,6987	23,5838	23,8714	NaN	23,6095	22,8116	NaN	23,5475	NaN			2	2	2	8,6	8,6	8,6	25,197	1,30E+08	7	23,5336	23,6917	23,3229	7	4	3	0,16614	-0,368811	A0A133CVB0	A0A133CVB0	CUM68_1491	DUF975 domain-containing protein
21,9096	21,9424	NaN	22,0709	22,3754	NaN	NaN	21,7185	21,9345	21,7846			3	3	3	13,6	13,6	13,6	33,178	7,21E+07	10	21,9623	22,0746	21,8125	7	4	3	0,112923	-0,262077	A0A133CKY6	A0A133CKY6	rsmA	Ribosomal RNA small subunit methyltransferase A
22,3446	NaN	22,6125	22,5574	22,7842	22,327	22,4814	NaN	NaN	22,3658			2	2	2	8,4	8,4	8,4	29,262	7,97E+07	9	22,4961	22,5747	22,3914	7	4	3	0,168887	-0,18329	A0A133CMR0	A0A133CMR0	xth	Exodeoxyribonuclease III
23,2767	23,5523	23,2657	23,4891	23,5905	NaN	NaN	22,9334	22,9532	22,8578	+		5	5	5	10,7	10,7	10,7	49,464	2,03E+08	14	23,2398	23,4348	22,9148	8	5	3	0,00147909	-0,520055	A0A132Z956	A0A132Z956	gor	Glutathione reductase
22,4677	22,2595	22,3425	22,4838	22,4878	21,5476	22,1988	22,162	NaN	NaN	+		8	8	8	12,8	12,8	12,8	81,636	1,81E+08	16	22,2437	22,4083	21,9695	8	5	3	0,0382936	-0,438799	A0A132P9P4	A0A132P9P4	yhgF	RNA binding protein S1
23,8606	23,7742	23,7355	23,6264	23,7614	23,2933	23,4391	23,2467	NaN	NaN	+		7	7	7	18,9	18,9	18,9	49,209	4,19E+08	19	23,5922	23,7516	23,3264	8	5	3	0,000645427	-0,425255	A0A132ZB48	A0A132ZB48	nox_3	NADH dehydrogenase
22,6084	22,5823	22,5805	22,7146	22,9809	NaN	22,5703	22,2383	NaN	22,2009	+		4	4	4	19,5	19,5	19,5	24,828	1,57E+08	14	22,5595	22,6934	22,3365	8	5	3	0,0360857	-0,356873	A0A132Z723	A0A132Z723	cmk	Cytidylate kinase
24,5489	24,4765	24,6222	24,8791	24,99	24,3647	24,3142	24,3754	NaN	NaN	+		4	4	4	9,7	9,7	9,7	48,718	5,30E+08	15	24,5714	24,7033	24,3514	8	5	3	0,0375847	-0,351904	A0A132P3S7	A0A132P3S7	tcyP	Cation:dicarboxylase symporter family transporter
23,6003	23,6532	23,6629	23,5706	23,9213	NaN	23,4144	23,5499	NaN	23,1178	+		6	6	6	16,8	16,8	16,8	33,15	3,65E+08	14	23,5613	23,6816	23,3607	8	5	3	0,0422645	-0,320922	A0A132YYN3	A0A132YYN3	lacC	Tagatose-6-phosphate kinase
23,4009	23,2597	23,1965	23,3983	23,2027	22,9842	NaN	22,8002	23,2001	NaN	+		4	4	4	15	15	15	49,254	2,16E+08	11	23,1803	23,2916	22,9948	8	5	3	0,0289439	-0,296799	A0A133CSE7	A0A133CSE7	lpdA	Dihydrolipoyl dehydrogenase
23,5157	23,5616	23,4701	23,402	23,5882	NaN	23,1633	23,4396	23,168	NaN	+		6	6	6	17,1	17,1	17,1	56,747	3,02E+08	18	23,4136	23,5075	23,257	8	5	3	0,0203283	-0,250545	A0A133CSR4	A0A133CSR4	dltA	D-alanine--D-alanyl carrier protein ligase
24,2196	23,8899	23,8155	24,0325	24,0244	24,2717	NaN	24,3554	NaN	24,8726	+		4	4	4	5,2	5,2	5,2	84,864	3,38E+08	15	24,1852	23,9964	24,4999	8	5	3	0,0226844	0,503534	A0A132Z463	A0A132Z463	pcrA_3	ATP-dependent DNA helicase
22,7252	22,7282	22,7049	22,571	22,2567	22,4459	22,7353	NaN	22,0903	NaN			4	4	4	16,7	16,7	16,7	32,192	1,55E+08	10	22,5322	22,5972	22,4238	8	5	3	0,376347	-0,173352	A0A133CLT6	A0A133CLT6	B1P95_11230	DegV family EDD domain-containing protein
22,2011	22,43	22,5294	22,4812	22,4004	22,0831	22,6137	NaN	NaN	22,2318			4	4	4	11,1	11,1	11,1	45,637	1,13E+08	16	22,3714	22,4084	22,3095	8	5	3	0,499576	-0,098896	A0A132Z318	A0A132Z318	proA	Gamma-glutamyl phosphate reductase
22,8782	22,6643	22,9878	22,7951	22,7627	NaN	NaN	22,7061	22,8617	22,5944			2	2	2	15,9	15,9	15,9	21,954	1,04E+08	4	22,7813	22,8176	22,7207	8	5	3	0,334102	-0,0968664	A0A132P541	A0A132P541	pheT_1	DUF4479 domain-containing protein
23,3347	23,6607	23,6817	22,7382	23,7514	23,2187	23,5243	NaN	23,69	NaN			5	5	5	34,3	34,3	34,3	22,378	2,74E+08	14	23,45	23,4333	23,4777	8	5	3	0,875164	0,044311	A0A2D0NJN9	A0A2D0NJN9	DTPHA_6006	Nitroreductase family protein
24,3182	24,2694	23,9951	24,2536	24,4631	24,2134	NaN	NaN	24,604	24,3781			3	3	3	17,4	17,4	17,4	26,728	3,30E+08	15	24,3119	24,2599	24,3985	8	5	3	0,329263	0,13864	A0A133CJ65	A0A133CJ65	divIVA	Cell division protein DivIVA
25,4819	25,0224	25,0313	24,9744	24,8338	NaN	25,4335	25,1571	NaN	25,0365			4	4	4	12,6	12,6	12,6	41,564	6,50E+08	25	25,1214	25,0688	25,2091	8	5	3	0,438126	0,140292	A0A133CLY6	A0A133CLY6	mnmA	tRNA-specific 2-thiouridylase MnmA
23,5273	23,4416	23,4942	23,4234	23,7325	24,173	23,3828	NaN	23,4448	NaN			5	5	5	14,6	14,6	14,6	43,666	3,64E+08	21	23,5775	23,5238	23,6669	8	5	3	0,500113	0,143066	A0A132PAL9	A0A132PAL9	aspB	Aminotransferase
22,9965	23,221	23,3061	NaN	NaN	NaN	NaN	NaN	23,6846	23,4671			2	2	2	7,5	7,5	7,5	26,585	1,66E+08	5	23,3351	23,1745	23,5759	5	3	2	0,0687372	0,401361	A0A132P689	A0A132P689	AWT83_0461	Probable transcriptional regulatory protein AS238_03140
NaN	23,2707	23,5009	23,4073	23,3384	NaN	23,6681	23,847	NaN	NaN	+		2	2	2	35,5	35,5	35,5	8,9019	1,37E+08	4	23,5054	23,3793	23,7575	6	4	2	0,0146746	0,378199	A0A132ZG80	A0A132ZG80	ykuJ	DUF1797 domain-containing protein
NaN	22,9759	23,098	22,7235	22,7356	23,2182	NaN	NaN	NaN	23,3669	+		3	3	3	12,4	12,4	12,4	41,466	1,32E+08	10	23,0197	22,8832	23,2926	6	4	2	0,0482632	0,409343	A0A133CNA8	A0A133CNA8	dapE	Probable succinyl-diaminopimelate desuccinylase
NaN	23,8265	24,2283	24,0898	24,057	24,0855	NaN	24,0465	NaN	NaN			5	5	5	24,5	24,5	24,5	28,125	3,75E+08	8	24,0556	24,0504	24,066	6	4	2	0,907231	0,0155869	A0A132ZGF6	A0A132ZGF6	gdh	Glucose 1-dehydrogenase
21,9989	21,9175	21,8243	22,0107	NaN	21,9729	NaN	NaN	22,0545	NaN			2	2	2	9,7	9,7	9,7	26,758	5,18E+07	5	21,9631	21,9378	22,0137	6	4	2	0,3358	0,0758367	A0A132Z712	A0A132Z712	rluB_2	Pseudouridine synthase
23,6282	23,1914	23,4303	23,5732	23,2479	NaN	NaN	21,9544	22,9971	NaN	+		2	2	2	7,7	7,7	7,7	34,061	1,49E+08	8	23,1461	23,4142	22,4758	7	5	2	0,0296054	-0,938455	A0A132PAB2	A0A132PAB2	AWT83_1247	Cation diffusion facilitator family transporter
23,6601	23,8681	23,9198	23,8963	23,9024	NaN	NaN	NaN	23,6432	23,0697	+		2	2	2	7,3	7,3	7,3	34,545	1,99E+08	15	23,7085	23,8494	23,3565	7	5	2	0,0349594	-0,492903	A0A133CNC0	A0A133CNC0	panE2	2-dehydropantoate 2-reductase
24,4384	24,2311	24,4206	24,3952	24,5958	24,1272	24,0421	NaN	NaN	NaN	+		2	2	2	4,3	4,3	4,3	54,621	2,36E+08	15	24,3215	24,4162	24,0846	7	5	2	0,0209216	-0,331601	A0A132Z8S3	A0A132Z8S3	A5810_00004	Arginine:ornithine antiporter
22,5161	22,3642	22,472	22,546	22,238	NaN	NaN	22,1597	22,0362	NaN	+		4	4	4	12,2	12,2	12,2	58,389	1,17E+08	8	22,3332	22,4273	22,098	7	5	2	0,0216394	-0,329274	A0A132ZFD2	A0A132ZFD2	AWT83_1288	Metal-dependent carboxypeptidase
23,9689	23,8904	24,206	24,1524	24,2015	24,7865	NaN	NaN	24,9057	NaN	+		3	3	3	10	10	10	35,983	2,41E+08	16	24,3016	24,0838	24,8461	7	5	2	0,00108631	0,762283	A0A133CNW7	A0A133CNW7	nrnA_2	Bifunctional oligoribonuclease/PAP phosphatase NrnA
22,2508	22,1579	22,0358	22,3342	21,9847	NaN	21,7694	21,9828	NaN	NaN			3	3	3	7,5	7,5	7,5	47,788	9,91E+07	9	22,0737	22,1527	21,8761	7	5	2	0,0736292	-0,276559	A0A132Z4S1	A0A132Z4S1	sufD_1	Fe-S cluster assembly protein SufD
23,396	23,2956	23,4237	23,1368	23,1209	NaN	23,056	23,0134	NaN	NaN			3	3	3	13,6	13,6	13,6	36,653	1,46E+08	8	23,2061	23,2746	23,0347	7	5	2	0,0739545	-0,239852	A0A133CL03	A0A133CL03	gpsA	Glycerol-3-phosphate dehydrogenase [NAD(P)+]
23,0327	22,7025	22,9736	23,0245	23,111	NaN	22,7022	22,826	NaN	NaN			4	4	4	21	21	21	32,874	2,01E+08	12	22,9104	22,9689	22,7641	7	5	2	0,153632	-0,204756	A0A133CMF1	A0A133CMF1	nfo	Probable endonuclease 4
24,2814	24,1638	24,2765	24,1716	24,6475	23,9876	NaN	NaN	24,2909	NaN			2	2	2	22	22	22	10,355	2,18E+08	6	24,2599	24,3082	24,1393	7	5	2	0,36171	-0,168896	A0A132P9T6	A0A132P9T6	rpsP	30S ribosomal protein S16
22,6742	22,7254	23,0004	22,4716	22,5343	NaN	NaN	22,6223	22,4468	NaN			3	3	3	12,5	12,5	12,5	30,303	1,05E+08	3	22,6393	22,6812	22,5345	7	5	2	0,403617	-0,146695	A0A132YZI9	A0A132YZI9	purR	Pur operon repressor
22,631	22,7298	22,695	22,5382	22,7697	NaN	NaN	22,4313	22,6556	NaN			3	3	3	18,9	18,9	18,9	19,603	1,28E+08	9	22,6358	22,6727	22,5435	7	5	2	0,211401	-0,129273	A0A132P612	A0A132P612	dut	dUTP diphosphatase
22,6658	22,4764	22,4731	22,4498	22,6904	22,4441	22,4577	NaN	NaN	NaN			6	6	6	9,3	9,3	9,3	100,47	1,80E+08	15	22,5225	22,5511	22,4509	7	5	2	0,303494	-0,100236	A0A5P3FTI1	A0A5P3FTI1	polA	DNA polymerase I
23,4203	23,5352	23,4875	23,5785	23,2483	NaN	NaN	23,2667	23,4985	NaN			2	2	2	18,3	18,3	18,3	11,87	1,32E+08	9	23,4336	23,454	23,3826	7	5	2	0,560272	-0,0713566	A0A132P6N2	A0A132P6N2	A5810_00035	Protein from nitrogen regulatory protein P-II (GLNB) family
23,0857	22,794	23,0477	23,019	23,0913	22,8081	NaN	NaN	23,0906	NaN			4	4	4	13,4	13,4	13,4	28,846	1,64E+08	14	22,9909	23,0075	22,9493	7	5	2	0,644106	-0,0582243	A0A133CK67	A0A133CK67	AWT83_1299	SDR family NAD(P)-dependent oxidoreductase
23,0061	22,7287	22,8296	22,8947	23,2697	NaN	NaN	NaN	23,112	22,6669			2	2	2	44,1	44,1	44,1	6,928	1,36E+08	8	22,9297	22,9458	22,8894	7	5	2	0,783986	-0,0563261	A0A133N173	A0A133N173	B1P95_03955	Primosomal protein N
22,5385	22,6852	22,735	22,656	22,5318	NaN	22,5357	22,6241	NaN	NaN			3	3	3	12,6	12,6	12,6	35,619	1,32E+08	13	22,6152	22,6293	22,5799	7	5	2	0,521089	-0,0494179	A0A133CMU6	A0A133CMU6	pta	Phosphate acetyltransferase
23,3099	23,4221	23,2949	23,3492	23,4409	NaN	23,6406	23,0855	NaN	NaN			3	3	3	4,6	4,6	4,6	88,508	2,45E+08	17	23,3633	23,3634	23,3631	7	5	2	0,998286	-0,000349617	A0A133CMT2	A0A133CMT2	mutS2	Endonuclease MutS2
22,8269	23,0892	23,3732	23,1331	23,0915	NaN	NaN	22,9736	23,3314	NaN			5	5	5	24,1	24,1	24,1	31,777	1,43E+08	5	23,117	23,1028	23,1525	7	5	2	0,785649	0,0497313	A0A133CHH4	A0A133CHH4	glxR	2-hydroxy-3-oxopropionate reductase
23,3477	23,2277	23,2753	23,3187	23,3162	23,4799	NaN	23,2992	NaN	NaN			7	7	7	24,1	24,1	24,1	38,161	3,09E+08	11	23,3235	23,2971	23,3896	7	5	2	0,178925	0,0924368	A0A132Z2S8	A0A132Z2S8	AWT83_0423	Zinc-type alcohol dehydrogenase-like protein
24,5863	24,3123	24,5322	24,5932	24,5028	NaN	NaN	24,4545	24,8508	NaN			2	2	2	18,7	18,7	18,7	14,014	3,17E+08	21	24,5474	24,5054	24,6526	7	5	2	0,326073	0,147273	A0A133CMR3	A0A133CMR3	gloA3	Lactoylglutathione lyase
NaN	22,1077	22,2279	22,1963	NaN	NaN	NaN	NaN	21,5325	NaN			2	2	2	6,3	6,3	6,3	35,074	5,20E+07	8	22,0161	22,1773	21,5325	4	3	1	1	-0,64483	A0A132Z6Q5	A0A132Z6Q5	gspA_1	Glycosyl transferase
NaN	23,331	NaN	23,3629	23,5035	NaN	NaN	23,0671	NaN	NaN			2	2	2	5,8	5,8	5,8	47,529	1,23E+08	10	23,3161	23,3991	23,0671	4	3	1	1	-0,332055	A0A133CIX4	A0A133CIX4	secY	Protein translocase subunit SecY
NaN	22,9529	23,0693	22,8049	NaN	NaN	22,7812	NaN	NaN	NaN			2	2	2	14,7	14,7	14,7	18,713	9,00E+07	7	22,9021	22,9423	22,7812	4	3	1	1	-0,161133	A0A132P295	A0A132P295	apt	Adenine phosphoribosyltransferase
NaN	21,2289	20,7803	21,2134	NaN	NaN	NaN	21,5567	NaN	NaN			3	3	3	6,8	6,8	6,8	50,773	4,99E+07	4	21,1948	21,0742	21,5567	4	3	1	1	0,482505	A0A133CMJ6	A0A133CMJ6	dnaB	Replicative DNA helicase
NaN	22,6285	22,9461	22,9511	23,203	NaN	22,9225	NaN	NaN	NaN			2	2	2	28,6	28,6	28,6	9,1033	1,07E+08	14	22,9303	22,9322	22,9225	5	4	1	1	-0,00964022	A0A132Z627	A0A132Z627	AWT83_0868	DUF2969 domain-containing protein
21,6223	NaN	22,0229	21,9129	21,6918	NaN	NaN	NaN	21,8163	NaN			3	3	3	20,1	20,1	20,1	16,974	5,83E+07	4	21,8132	21,8125	21,8163	5	4	1	1	0,00382137	A0A132P3H6	A0A132P3H6	nusB	Transcription antitermination protein NusB
NaN	22,8064	22,4975	22,6608	22,3858	22,6581	NaN	NaN	NaN	NaN			4	4	4	12,2	12,2	12,2	37,797	8,76E+07	16	22,6017	22,5876	22,6581	5	4	1	1	0,0704455	A0A132ZIZ3	A0A132ZIZ3	tmpC	ABC superfamily ATP binding cassette transporter, binding protein
26,153	25,9678	25,9944	26,2853	26,3401	NaN	NaN	NaN	NaN	25,6161			3	3	3	7,4	7,4	7,4	48,05	1,02E+09	16	26,0595	26,1481	25,6161	6	5	1	1	-0,532004	A0A133CQC5	A0A133CQC5	cypC	Cytochrome P450
23,6624	23,2961	23,4377	23,5116	23,5015	23,0576	NaN	NaN	NaN	NaN			4	4	4	17,1	17,1	17,1	31,774	2,58E+08	24	23,4112	23,4819	23,0576	6	5	1	1	-0,424333	A0A133CPY5	A0A133CPY5	rlmB	23S rRNA (Guanosine(2251)-2-O)-methyltransferase RlmB
22,9696	23,0062	23,0953	23,0362	23,04	NaN	NaN	22,6565	NaN	NaN			3	3	3	14,8	14,8	14,8	26,353	1,36E+08	3	22,9673	23,0295	22,6565	6	5	1	1	-0,372994	A0A133CHK7	A0A133CHK7	B1P95_00185	B3/4 domain-containing protein
23,0973	22,9772	23,3744	23,1405	23,2476	NaN	22,9021	NaN	NaN	NaN			3	3	3	51,5	51,5	51,5	11,657	1,53E+08	6	23,1232	23,1674	22,9021	6	5	1	1	-0,265266	A0A132ZKD5	A0A132ZKD5	rpoZ	DNA-directed RNA polymerase subunit omega
22,4527	22,4341	22,4311	22,3394	22,5499	NaN	NaN	22,2268	NaN	NaN			5	5	5	15,9	15,9	15,9	48,393	1,51E+08	16	22,4056	22,4414	22,2268	6	5	1	1	-0,214589	A0A132Z0D5	A0A132Z0D5	B1P95_03860	Insulinase family protein
22,8505	22,732	23,1756	23,0378	23,1022	22,7668	NaN	NaN	NaN	NaN			2	2	2	10,3	10,3	10,3	34,193	1,31E+08	9	22,9442	22,9796	22,7668	6	5	1	1	-0,212769	A0A132YYH0	A0A132YYH0	murB	UDP-N-acetylenolpyruvoylglucosamine reductase
22,9913	23,1215	23,4012	22,9548	23,3547	22,978	NaN	NaN	NaN	NaN			3	3	3	10,2	10,2	10,2	27,547	1,21E+08	9	23,1336	23,1647	22,978	6	5	1	1	-0,186674	A0A133CTF0	A0A133CTF0	BU187_05270	MBL fold metallo-hydrolase
22,3741	22,4745	22,6708	22,4818	22,3727	NaN	NaN	22,3355	NaN	NaN			4	4	4	24,3	24,3	24,3	29,085	1,17E+08	11	22,4516	22,4748	22,3355	6	5	1	1	-0,139257	A0A132Z0Y0	A0A132Z0Y0	codY	GTP-sensing transcriptional pleiotropic repressor CodY
22,3575	22,3224	22,066	22,1859	21,9961	NaN	22,0713	NaN	NaN	NaN			2	2	2	13	13	13	21,207	5,98E+07	6	22,1665	22,1856	22,0713	6	5	1	1	-0,11423	A0A132Z122	A0A132Z122	xpt	Xanthine phosphoribosyltransferase
23,8013	23,6855	23,9482	23,7969	23,9955	NaN	NaN	NaN	23,8206	NaN			5	5	5	21,8	21,8	21,8	29,305	2,64E+08	12	23,8413	23,8455	23,8206	6	5	1	1	-0,0249405	A0A133CHQ3	A0A133CHQ3	accA	Acetyl-coenzyme A carboxylase carboxyl transferase subunit alpha
22,9006	23,1122	22,8704	22,8029	22,9404	NaN	NaN	NaN	22,9004	NaN			3	3	3	12,7	12,7	12,7	32,984	1,06E+08	9	22,9211	22,9253	22,9004	6	5	1	1	-0,0248951	A0A133CPL6	A0A133CPL6	galM4	Aldose 1-epimerase
21,7936	21,7005	21,8216	21,5558	21,9141	NaN	NaN	NaN	21,7407	NaN			2	2	2	8,9	8,9	8,9	26,854	4,24E+07	4	21,7544	21,7571	21,7407	6	5	1	1	-0,016394	A0A132Z967	A0A132Z967	rsmG	Ribosomal RNA small subunit methyltransferase G
23,6392	23,8038	23,6512	23,9352	23,9429	23,8105	NaN	NaN	NaN	NaN			3	3	3	11,2	11,2	11,2	37,368	2,34E+08	5	23,7971	23,7945	23,8105	6	5	1	1	0,0160385	A0A132Z3D8	A0A132Z3D8	aroF	3-deoxy-7-phosphoheptulonate synthase
22,1837	22,2156	22,3759	22,0904	21,988	NaN	NaN	NaN	22,2337	NaN			2	2	2	14,5	14,5	14,5	19,532	6,43E+07	12	22,1812	22,1707	22,2337	6	5	1	1	0,0630146	A0A132Z561	A0A132Z561	AWT83_1305	Phosphoesterase
23,1699	22,9544	22,7773	22,9094	23,13	NaN	NaN	NaN	23,105	NaN			4	4	4	22,9	22,9	22,9	36,872	1,38E+08	5	23,0077	22,9882	23,105	6	5	1	1	0,116835	A0A132Z550	A0A132Z550	prsA3	Foldase protein PrsA
22,6822	22,7307	22,454	22,5262	23,0323	22,9129	NaN	NaN	NaN	NaN			3	3	3	14,4	14,4	14,4	20,932	1,01E+08	11	22,723	22,6851	22,9129	6	5	1	1	0,227794	A0A133CK50	A0A133CK50	grpE	Protein GrpE
NaN	22,8069	22,9002	22,8448	NaN	NaN	NaN	NaN	NaN	NaN			2	2	2	13,2	13,2	13,2	16,496	6,78E+07	9	22,8507	22,8507	NaN	3	3	0	1	NaN	A0A132P374	A0A132P374	AWT83_1484	Acetyltransferase, GNAT family
NaN	21,6192	21,5657	21,7402	NaN	NaN	NaN	NaN	NaN	NaN			2	2	2	12,1	12,1	12,1	19,446	3,27E+07	3	21,6417	21,6417	NaN	3	3	0	1	NaN	A0A132P8Q4	A0A132P8Q4	rimM	Ribosome maturation factor RimM
22,222	NaN	22,9251	22,4233	NaN	NaN	NaN	NaN	NaN	NaN			3	3	3	10	10	10	49,319	9,92E+07	5	22,5235	22,5235	NaN	3	3	0	1	NaN	A0A132YZ03	A0A132YZ03	AWT83_0442	Insulinase family protein
NaN	NaN	22,1763	22,2011	22,4616	NaN	NaN	NaN	NaN	NaN			2	2	2	5,8	5,8	5,8	42,478	5,28E+07	7	22,2797	22,2797	NaN	3	3	0	1	NaN	A0A133CGP1	A0A133CGP1	iscS2	Aminotransferase V
NaN	NaN	23,1192	22,7269	23,0405	NaN	NaN	NaN	NaN	NaN			4	4	4	9,7	9,7	9,7	60,803	7,96E+07	4	22,9622	22,9622	NaN	3	3	0	1	NaN	A0A133CIQ3	A0A133CIQ3	metW	Class I SAM-dependent methyltransferase
23,0633	22,8063	NaN	NaN	22,9147	NaN	NaN	NaN	NaN	NaN			3	3	3	29,5	29,5	29,5	17,165	1,13E+08	4	22,9281	22,9281	NaN	3	3	0	1	NaN	A0A133CIW5	A0A133CIW5	argR2	Arginine repressor
22,3423	22,2229	22,4974	NaN	NaN	NaN	NaN	NaN	NaN	NaN			2	2	2	7,6	7,6	7,6	38,507	6,58E+07	3	22,3542	22,3542	NaN	3	3	0	1	NaN	A0A133CN70	A0A133CN70	aroB	3-dehydroquinate synthase
NaN	22,7087	22,7045	22,6692	22,8005	NaN	NaN	NaN	NaN	NaN			3	3	3	16,2	16,2	16,2	25,699	8,89E+07	5	22,7207	22,7207	NaN	4	4	0	1	NaN	A0A132P271	A0A132P271	ftsE	Cell division ATP-binding protein FtsE
21,0886	21,2939	21,0071	21,186	NaN	NaN	NaN	NaN	NaN	NaN			2	2	2	5,9	5,9	5,9	50,779	2,85E+07	3	21,1439	21,1439	NaN	4	4	0	1	NaN	A0A132Z0Q7	A0A132Z0Q7	trmFO	Methylenetetrahydrofolate--tRNA-(uracil-5-)-methyltransferase TrmFO
NaN	23,5053	23,9002	23,8781	23,8491	NaN	NaN	NaN	NaN	NaN			2	2	2	17,4	17,4	17,4	13,221	1,43E+08	6	23,7832	23,7832	NaN	4	4	0	1	NaN	A0A132Z496	A0A132Z496	rbfA	Ribosome-binding factor A
21,7829	21,626	21,6658	21,6457	NaN	NaN	NaN	NaN	NaN	NaN			2	2	2	10,7	10,7	10,7	27,989	3,69E+07	3	21,6801	21,6801	NaN	4	4	0	1	NaN	A0A132ZFC6	A0A132ZFC6	aroD	3-dehydroquinate dehydratase
NaN	22,6096	22,9672	22,747	22,8726	NaN	NaN	NaN	NaN	NaN			2	2	2	13,9	13,9	13,9	23,329	1,31E+08	6	22,7991	22,7991	NaN	4	4	0	1	NaN	A0A132ZIT7	A0A132ZIT7	rpe	Ribulose-phosphate 3-epimerase
NaN	24,0565	23,9042	24,0957	23,9891	NaN	NaN	NaN	NaN	NaN			4	4	4	10	10	10	50,342	1,99E+08	10	24,0114	24,0114	NaN	4	4	0	1	NaN	A0A133CGR0	A0A133CGR0	pepC	Aminopeptidase
NaN	23,4823	23,5591	23,71	23,8621	NaN	NaN	NaN	NaN	NaN			4	4	4	18,2	18,2	18,2	18,452	2,38E+08	8	23,6534	23,6534	NaN	4	4	0	1	NaN	A0A133CLV7	A0A133CLV7	ssb_1	Single-stranded DNA-binding protein
23,9071	23,7531	23,7686	23,7667	NaN	NaN	NaN	NaN	NaN	NaN			2	2	2	7,1	7,1	7,1	40,919	1,94E+08	7	23,7989	23,7989	NaN	4	4	0	1	NaN	A0A133CMG3	A0A133CMG3	CUM68_0608	Aminopeptidase P family protein
22,2211	22,1437	22,5123	NaN	22,1855	NaN	NaN	NaN	NaN	NaN			3	3	3	27	27	27	16,838	7,02E+07	5	22,2656	22,2656	NaN	4	4	0	1	NaN	A0A133CQI2	A0A133CQI2	msrB	Peptide methionine sulfoxide reductase MsrB
21,3024	21,1381	21,275	21,1276	NaN	NaN	NaN	NaN	NaN	NaN			2	2	2	15	15	15	19,232	2,83E+07	3	21,2108	21,2108	NaN	4	4	0	1	NaN	A0A133CU61	A0A133CU61	nadR	DNA-binding protein
22,019	22,0121	21,9956	22,0461	NaN	NaN	NaN	NaN	NaN	NaN			2	2	2	2,2	2,2	2,2	85,468	5,72E+07	4	22,0182	22,0182	NaN	4	4	0	1	NaN	A0A133CZI8	A0A133CZI8	macB_3	ABC transporter ATP-binding protein/permease
22,0773	22,0684	22,0919	21,9307	NaN	NaN	NaN	NaN	NaN	NaN			4	4	4	4,4	4,4	4,4	136,11	6,13E+07	9	22,0421	22,0421	NaN	4	4	0	1	NaN	A0A133CZT7	A0A133CZT7	smc	Chromosome partition protein Smc
23,6007	23,4008	23,58	23,4741	24,0983	NaN	NaN	NaN	NaN	NaN			3	3	3	28,8	28,8	28,8	15,398	1,82E+08	5	23,6308	23,6308	NaN	5	5	0	1	NaN	A0A132P4N4	A0A132P4N4	spxA	Global transcriptional regulator Spx

22,1358	22,131	22,1526	22,1965	22,0757	NaN	NaN	NaN	NaN	NaN		3	3	3	11,7	11,7	11,7	34,056	7,33E+07	8	22,1383	22,1383	NaN	5	5	0	1	NaN	A0A132P8E4	A0A132P8E4	era	GTPase Era
25,6929	25,309	25,3625	25,3839	25,6791	NaN	NaN	NaN	NaN	NaN		4	4	4	14,5	14,5	14,5	36,631	5,63E+08	11	25,4855	25,4855	NaN	5	5	0	1	NaN	A0A132P8Q3	A0A132P8Q3	cbh_3	Choloylglycine hydrolase
22,5727	22,5652	22,7009	22,6917	22,5293	NaN	NaN	NaN	NaN	NaN		5	5	5	20	20	20	34,058	1,28E+08	9	22,612	22,612	NaN	5	5	0	1	NaN	A0A132Z012	A0A132Z012	qor	Alcohol dehydrogenase
22,4728	22,2882	22,5636	22,0668	22,4795	NaN	NaN	NaN	NaN	NaN		3	3	3	7,9	7,9	7,9	45,605	7,03E+07	10	22,3742	22,3742	NaN	5	5	0	1	NaN	A0A132Z8A9	A0A132Z8A9	thiI_2	Probable tRNA sulfurtransferase
22,0732	22,284	22,0141	22,1263	21,9529	NaN	NaN	NaN	NaN	NaN		4	4	4	7,6	7,6	7,6	71,957	5,68E+07	10	22,0901	22,0901	NaN	5	5	0	1	NaN	A0A132Z8K2	A0A132Z8K2	yjlD	NAD(P)/FAD-dependent oxidoreductase
21,7773	21,8023	21,773	21,948	21,8537	NaN	NaN	NaN	NaN	NaN		3	3	3	6,1	6,1	6,1	64,63	7,11E+07	8	21,8308	21,8308	NaN	5	5	0	1	NaN	A0A132ZC57	A0A132ZC57	dxs	1-deoxy-D-xylulose-5-phosphate synthase
21,9962	21,7524	22,0091	21,9106	21,8445	NaN	NaN	NaN	NaN	NaN		2	2	2	10,7	10,7	10,7	27,445	5,50E+07	7	21,9026	21,9026	NaN	5	5	0	1	NaN	A0A132ZCC2	A0A132ZCC2	ntcA	Crp/Fnr family transcriptional regulator
22,5121	22,4388	22,339	22,4403	23,2367	NaN	NaN	NaN	NaN	NaN		4	4	4	22,3	22,3	22,3	15,801	1,28E+08	14	22,5934	22,5934	NaN	5	5	0	1	NaN	A0A132ZF95	A0A132ZF95	divIVA2	Cell cycle protein GpsB
22,6821	22,3014	22,639	22,5187	22,436	NaN	NaN	NaN	NaN	NaN		2	2	2	16,9	16,9	16,9	14,573	6,40E+07	9	22,5154	22,5154	NaN	5	5	0	1	NaN	A0A132ZHZ0	A0A132ZHZ0	osmC	Ohr family peroxiredoxin
22,027	22,0474	22,0333	21,815	21,9397	NaN	NaN	NaN	NaN	NaN		2	2	2	9,5	9,5	9,5	31,692	5,92E+07	5	21,9725	21,9725	NaN	5	5	0	1	NaN	A0A132ZJ42	A0A132ZJ42	hslO	33 kDa chaperonin
22,628	22,6056	22,6483	22,5158	22,4346	NaN	NaN	NaN	NaN	NaN		2	2	2	5,2	5,2	5,2	46,104	6,98E+07	4	22,5665	22,5665	NaN	5	5	0	1	NaN	A0A133CIA3	A0A133CIA3	tagH	ABC transporter ATP-binding protein
21,7097	21,5848	21,8519	21,6243	21,6571	NaN	NaN	NaN	NaN	NaN		3	3	3	27	27	27	17,582	4,54E+07	4	21,6856	21,6856	NaN	5	5	0	1	NaN	A0A133CID4	A0A133CID4	greA	Transcription elongation factor GreA
21,2019	21,2412	21,2842	20,9293	21,0211	NaN	NaN	NaN	NaN	NaN		2	2	2	5,6	5,6	5,6	50,262	2,78E+07	5	21,1355	21,1355	NaN	5	5	0	1	NaN	A0A133CJY8	A0A133CJY8	murE	Lipid II isoglutaminyl synthase (glutamine-hydrolyzing) subunit MurT
23,979	23,5366	23,6974	23,5762	23,7585	NaN	NaN	NaN	NaN	NaN		3	3	3	15,6	15,6	15,6	33,448	2,41E+08	9	23,7095	23,7095	NaN	5	5	0	1	NaN	A0A133CKG8	A0A133CKG8	rapZ	Nucleotide-binding protein AS238_11640
22,1476	21,7948	21,966	21,8404	21,8198	NaN	NaN	NaN	NaN	NaN		4	4	4	15,9	15,9	15,9	37,718	9,43E+07	8	21,9137	21,9137	NaN	5	5	0	1	NaN	A0A133CP22	A0A133CP22	B1P95_03395	3-carboxymuconate cyclase
22,9369	23,0611	22,923	22,9263	23,0812	NaN	NaN	NaN	NaN	NaN		3	3	3	10,9	10,9	10,9	43,993	1,08E+08	11	22,9857	22,9857	NaN	5	5	0	1	NaN	A0A133CQB8	A0A133CQB8	ackA_4	Acetate kinase
22,657	22,5901	22,9149	22,5418	22,8483	NaN	NaN	NaN	NaN	NaN		5	5	5	4,6	4,6	4,6	127,39	1,84E+08	19	22,7104	22,7104	NaN	5	5	0	1	NaN	A0A133CSN6	A0A133CSN6	cfiB_1	Pyruvate carboxylase
22,8352	22,4755	22,5309	22,326	22,1523	NaN	NaN	NaN	NaN	NaN		5	5	5	14,9	14,9	14,9	49,052	1,20E+08	9	22,464	22,464	NaN	5	5	0	1	NaN	A0A133CUU0	A0A133CUU0	murF	UDP-N-acetylmuramoyl-tripeptide--D-alanyl-D-alanine ligase
22,8717	22,914	23,2523	23,046	23,2261	NaN	NaN	NaN	NaN	NaN		2	2	2	10	10	10	12,887	1,22E+08	9	23,062	23,062	NaN	5	5	0	1	NaN	A0A133CZC6	A0A133CZC6	asp2	Alkaline-shock protein
22,4017	22,2451	22,5082	22,3448	22,3475	NaN	NaN	NaN	NaN	NaN		4	4	4	12,2	12,2	12,2	38,181	9,60E+07	14	22,3695	22,3695	NaN	5	5	0	1	NaN	A0A133MSW5	A0A133MSW5	metN2	Methionine import ATP-binding protein MetN
23,668	23,0746	23,1855	23,1532	23,3162	NaN	NaN	NaN	NaN	NaN		4	4	4	7	7	7	86,873	2,86E+08	9	23,2795	23,2795	NaN	5	5	0	1	NaN	A0A1A7S9W6	A0A1A7S9W6	gshAB	Glutathione biosynthesis bifunctional protein GshAB
